# Isolation of halogen-substituted silylium ions

**DOI:** 10.1038/s41557-025-01880-2

**Published:** 2025-07-31

**Authors:** Tobias Randt, Morten Lehmann, Elisabeth Irran, Martin Kaupp, Hendrik F. T. Klare, Martin Oestreich

**Affiliations:** Institut für Chemie, Technische Universität Berlin, Berlin, Germany

**Keywords:** Inorganic chemistry, Organic chemistry

## Abstract

The existence and intermediacy of halogen-substituted silylium ions have been the subject of speculation for decades. These elusive reactive intermediates are synthetically attractive because of their computationally predicted super Lewis acidity and their relevance in several synthetic transformations such as recycling of waste from the Müller–Rochow process and hydrodefluorination of per- and polyfluoroalkyl substances. Here we report the generation and characterization of all halogen-substituted silylium ions of type [Alk_2_XSi(HCB_11_H_5_Br_6_)] (X = F, Cl, Br or I; Alk = Me, Et, *i*Pr or *t*Bu). While the established Corey hydride transfer reaction fails to make such ions in the condensed phase, the protolysis of the halosilanes Alk_2_XSi‒LG (LG = H or Ph) using Reed’s superacidic benzenium ion [H(C_6_H_6_)]^+^[HCB_11_H_5_Br_6_]^−^ provides practical access. The full series of counteranion-stabilized *i*Pr_2_XSi^+^ cations is isolated and crystallographically characterized. The obtained halogen-substituted silylium ions are more Lewis acidic than their known trialkyl- and hydrogen-substituted congeners, as verified by quantitative assessment of their fluoride ion affinities using density functional theory calculations.

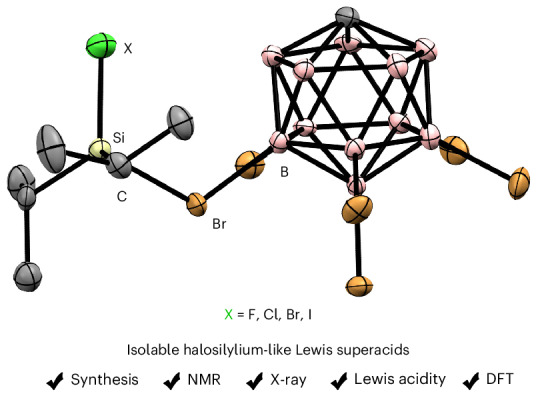

## Abstract (web summary)

Halogen-substituted silylium ions are compounds for which Lewis superacidity has been predicted, but their synthesis has proven challenging. Now a series of ionic halosilylium Lewis superacids have been prepared in the condensed phase, opening the door to their application in synthetic processes.

## Main

**Fig. 1:**
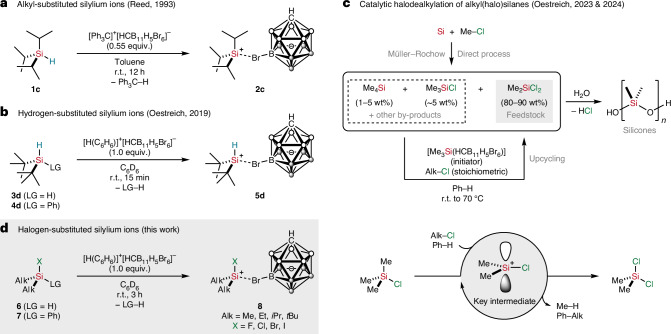
Synthetic routes to silylium ions. **a**, Preparation of alkyl-substituted silylium carborates by hydride abstraction^7^. **b**, Preparation of hydrogen-substituted silylium carborates by dehydrogenative or dephenylative protolysis^15^. **c**, Chlorine-substituted silylium ions as key intermediates in the catalytic halodealkylation of alkyl(halo)silanes with relevance to the Müller–Rochow direct process^16^^,^^17^. **d**, Protolysis strategy to access halogen-substituted, counteranion-stabilized silylium ions. Alk, alkyl substituent; r.t., room temperature. White circles indicate B–H, and grey circles indicate B–Br.

The debate on the existence of silylium ions (R_3_Si^+^) in the condensed phase, the so-called silylium ion problem^1^, was finally resolved with the isolation and characterization of the tricoordinate (free) trimesitylsilylium ion (Mes_3_Si^+^) (ref. ^2^). Despite these controversial beginnings^3^, silylium ions have nowadays emerged as potent reagents and catalysts in synthetic chemistry^4,5^. In catalytic transformations, tetracoordinate trialkyl-substituted silylium ions stabilized by a donor molecule such as the solvent^6^ or counteranion^7^ are typically used as initiators, and the catalytic cycle is then maintained by the self-regeneration of the silylium ion^5^. The traditional way to make these reactive intermediates is the Corey reaction, that is, the hydride abstraction from the corresponding hydrosilane with the trityl (Ph_3_C^+^) salt of a weakly coordinating anion^8^. Using halogenated 1-carba-*closo*-dodecaborates, Reed and co-workers succeeded in the synthesis and solid-state characterization of counteranion-stabilized trialkylsilylium ions such as [*i*Pr_3_Si(HCB_11_H_5_Br_6_)] (refs. ^7,9–11^) (**1c** → **2c**; Fig. 1a). Although the silyl cation is coordinated by one halogen atom of the carborate cluster, this interaction is energetically weak^9,10^, and the carborate salts show silylium-ion-like reactivity in solution^5^.

Our laboratory introduced a general approach for the rapid access of carborate-stabilized silylium ions based on the chemoselective protolysis of various tetraorganosilanes using Reed’s superacidic benzenium salt^12^ [H(C_6_H_6_)]^+^[HCB_11_H_5_Br_6_]^−^ (refs. ^13,14^). Following this strategy, we achieved the synthesis of the full series of hydrogen-substituted silylium ions either by dehydrogenation (leaving group (LG) = H) or dephenylation (LG = Ph), as exemplarily shown for the generation of the secondary silylium ion [*t*Bu_2_HSi(HCB_11_H_5_Br_6_)] (ref. ^15^) (**3d** or **4d** → **5d**; Fig. 1b).

Recently, we disclosed a protocol for the catalytic halodealkylation of fully alkylated silanes^16^ and proposed halogen-substituted silylium ions of the type [R_2_XSi(HCB_11_H_5_Br_6_)] as the key intermediates in the corresponding twofold halodealkylation process^17^ (Fig. 1c). This method enables the upcycling of Me_4_Si and Me_3_SiCl, accumulating as by-products from the Müller–Rochow direct process, into higher-value Me_2_SiCl_2_, which serves as feedstock monomer for the silicone production^18,19^. Within the catalytic cycle, an arenium ion is supposed to cleave the C(*sp*^3^)‒Si bond in trialkylhalosilanes, giving rise to hitherto unknown halosilylium ions^17^. Of note, Dolgov, Voronkov and Borisov have initially postulated the intermediacy of chlorosilylium ions in the substituent redistribution reaction of dialkylchlorosilanes promoted by catalytic amounts of AlCl_3_ (ref. ^20^). However, experimental evidence of these reactive intermediates is still lacking. While both inter- and intramolecular halogen stabilization of silylium ions have been investigated systematically^21,22^, work on halogen-substituted silylium ions has been limited to theoretical^23^ and gas-phase studies^24^.

The intermolecular interaction between silylium ions and halides is also the critical step of one of the most prominent applications of trialkylsilylium ions^5^, that is, the catalytic hydrodefluorination of aliphatic C(*sp*^3^)–F bonds in fluorocarbons^25^. In the search for even more fluoridophilic silylium ions, which even enable the hydrodefluorination of environmentally harmful per- and polyfluoroalkyl substances (PFAS), Gusev and Ozerov identified chlorine-substituted silylium ions as promising candidates by calculating the hydride ion affinity and fluoride ion affinity (FIA) of various silylium ions^26^. Compared with trialkyl- and hydrogen-substituted silylium ions, these cations are expected to be more electrophilic, as evident by the increasing FIAs in the order Me_3_Si^+^ < Me_2_HSi^+^ < Me_2_ClSi^+^.

Given the importance and relevance of halogen-substituted silylium ions, we envisaged their independent preparation and characterization in the condensed phase. Here, we report the synthesis and solid-state structures of halosilylium ions of type [Alk_2_XSi(HCB_11_H_5_Br_6_)] (X = F, Cl, Br or I; Alk = Me, Et, *i*Pr or *t*Bu). The key to accessing these long-sought reactive intermediates is the implementation of the protolysis approach by dehydrogenation (LG = H) or dephenylation (LG = Ph) of the corresponding hydro- or phenylsilanes using Reed’s superacidic benzenium ion [H(C_6_H_6_)]^+^[HCB_11_H_5_Br_6_]^−^ (**6** or **7** → **8**; Fig. 1d).

## Results and discussion

### Synthesis

**Fig. 2:**
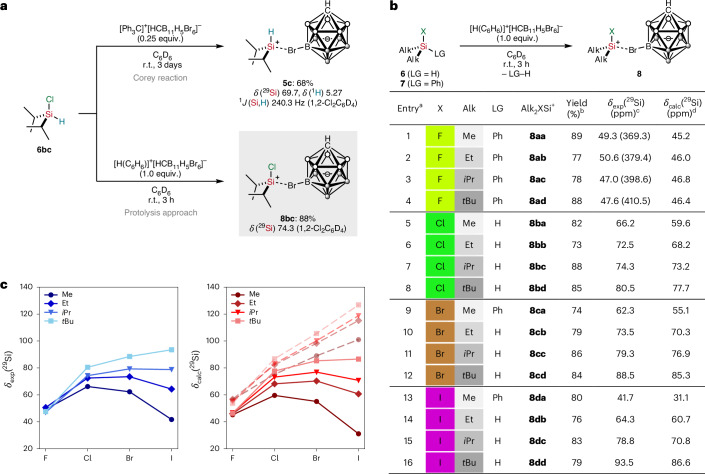
Synthesis and NMR spectroscopic characterization of halogen-substituted silylium ions. **a**, Attempts to generate a chlorine-substituted silylium ion by classical hydride abstraction (top) and successful synthesis by our protolysis approach (bottom). **b**, Synthesis of the full series of halogen-substituted silylium carborates. For the preparation of the individual silanes as silylium ion precursors, see Supplementary Figs. 1–4 in Supplementary Information Section 3. **c**, Plots of the experimental (left) and calculated (right) ^29^Si NMR chemical shifts of the halosilylium carborates. The computed ^29^Si chemical shifts (in ppm relative to tetramethylsilane) were obtained at the X2C-PBE0/x2c-TZVPall-2c level of theory, comparing one-component (1c) calculations with only scalar-relativistic effects (values on the dashed lines) and two-component (2c) calculations including also spin–orbit effects (values on the solid lines). ^a^All reactions were performed at ca. 0.02 mmol scale in C_6_D_6_ (0.2 ml, 0.1 M) in a glovebox under an argon atmosphere. ^b^Isolated yields. ^c^Experimental ^29^Si NMR chemical shifts determined by ^1^H,^29^Si HMQC NMR spectroscopy (500/99 MHz, 298 K, optimized for *J* = 7 Hz) in 1,2-Cl_2_C_6_D_4_. ^1^*J*(Si,F) coupling constant in Hz in parentheses. ^d^Computed ^29^Si NMR chemical shifts at the ‘exact two-component’ (X2C) spin–orbit PBE0/x2c-TZVPall-2c level.

In our initial attempt to generate a halogen-substituted silylium ion, we tested the classical Corey hydride abstraction from a dialkyl-substituted halosilane and chose chlorodiisopropylsilane (**6bc**) as a test substrate, as we knew from our previous work that the bulky isopropyl group prevents any redistribution of substituents^27,28^ (Fig. 2a, top). When the trityl salt [Ph_3_C]^+^[HCB_11_H_5_Br_6_]^−^ was treated with an excess of *i*Pr_2_SiHCl (**6bc**) in benzene at room temperature, a slow reaction occurred, as indicated by a gradual fading of the yellow reaction mixture. However, nuclear magnetic resonance (NMR) spectroscopic and X-ray crystallographic analysis revealed the exclusive formation of hydrogen-substituted silylium carborate [*i*Pr_2_HSi(HCB_11_H_5_Br_6_)] (**5c**), which is characterized by a ^29^Si NMR chemical shift of *δ* = 69.7 ppm and a diagnostic signal in the ^1^H NMR spectrum for the intact Si–H bond at *δ* = 5.27 ppm with a ^1^*J*(Si,H) coupling constant of 240 Hz. The same reaction outcome was also obtained starting from the corresponding iodosilane *i*Pr_2_SiHI (**6dc**), and reducing the silane equivalents to stoichiometric quantities led only to a slower reaction rate. In all cases, no formation of the desired halogen-substituted silylium ion was detected. This result was unexpected, as the trityl cation should not be able to heterolytically cleave silicon‒halogen bonds owing to its substantially lower FIA compared with secondary silylium ions^26^. We assumed that initial hydride transfer generates a halosilylium intermediate that immediately abstracts the halide from the starting silane to eventually arrive at the observed hydrogen-substituted silylium carborate **5c**. This hypothesis was later corroborated by an independent control experiment (see ‘Reactivity‘ section).

In another effort to access a halogen-substituted silylium ion, we tested our protolysis approach, which already enabled the synthesis of the hydrogen-substituted congeners^15^. Following this strategy, a pale yellow suspension of Reed’s benzenium salt [H(C_6_H_6_)]^+^[HCB_11_H_5_Br_6_]^−^ in benzene was treated with equimolar amounts of *i*Pr_2_SiHCl (**6cb**), resulting in immediate gas evolution and the formation of an off-white solid (Fig. 2a, bottom). NMR spectroscopic analysis now indicated successful Si–H bond heterolysis, as apparent by the disappearance of the signal for the Si–H bond in the ^1^H NMR spectrum. The measured ^29^Si NMR chemical shift of *δ* = 74.3 ppm was shifted downfield relative to the hydrogen-substituted silylium ion **5c** (Δ*δ* = 4.6 ppm). Because the silylium carborate salts are not very soluble in benzene, the NMR spectra were recorded in deuterated *ortho*-dichlorobenzene. Unambiguous evidence for the formation of the chlorine-substituted, counteranion-stabilized silylium ion [*i*Pr_2_ClSi(HCB_11_H_5_Br_6_)] (**8bc**) was eventually provided by X-ray diffraction analysis (see ‘Crystallographic characterization’ section).

We next embarked on a systematic investigation of halogen-substituted silylium ions by variation of the halogen substituent (X = F, Cl, Br or I) and alkyl substituent (Alk = Me, Et, *i*Pr or *t*Bu) in [Alk_2_XSi(HCB_11_H_5_Br_6_)] (Fig. 2b). Using equimolar amounts of benzenium salt [H(C_6_H_6_)]^+^[HCB_11_H_5_Br_6_]^−^, *i*Pr_2_SiHBr (**6cc**) and *i*Pr_2_SiHI (**6dc**) were equally converted in high yields to the corresponding halosilylium ions **8cc** and **8dc**, respectively (table in Fig. 2b, entries 11 and 15). Conversely, the protolysis of *i*Pr_2_SiHF (**6ac**) led to a 1:1 mixture of [*i*Pr_2_HSi(HCB_11_H_5_Br_6_)] (**5c**) and [*i*Pr_2_FSi(HCB_11_H_5_Br_6_)] (**8ac**). The observed reaction outcome was unclear at this stage, but we had already speculated that fluoride abstraction from *i*Pr_2_SiHF (**6ac**) by the transiently formed fluorosilylium ion *i*Pr_2_FSi^+^ (**8ac**) competes with the dehydrogenative protolysis. We therefore used phenyl as the leaving group (LG = Ph) because dearylation (protodesilylation) proved to be a more facile and faster process than the dehydrogenation (LG = H) in our previous study^15^. Indeed, selective formation of [*i*Pr_2_FSi(HCB_11_H_5_Br_6_)] (**8ac**) was now achieved by dephenylation of *i*Pr_2_PhSiF (**7ac**) under the same reaction set-up (table in Fig. 2b, entry 3). In addition to the isopropyl group, methyl, ethyl and *tert*-butyl substituents were also tolerated in this reaction (Fig. 2b). It is worth mentioning that, unlike hydrogen-substituted silylium ions **5**, the halogen-substituted silylium ions **8** were obtained without any detectable substituent redistribution, even when decorated with small methyl or ethyl groups at the silicon atom that are prone to intermolecular exchange reactions^27,28^.

### NMR spectroscopic characterization

The measured solution ^29^Si NMR chemical shifts of the halogen-substituted silylium carborates **8** span a relatively wide spectral range (*δ*(^29^Si) = 94–41 ppm; Fig. 2b) compared with the purely alkyl-substituted tertiary silylium carborates **2** (*δ*(^29^Si) = 113–93 ppm)^5^. Replacement of one alkyl group in [Alk_3_Si(HCB_11_H_5_Br_6_)] (**2**) by a halogen atom generally leads to a considerable high-field shift of the ^29^Si NMR resonance. Although the ^29^Si NMR chemical shift of classical trialkylsilylium ions is rather insensitive to the alkyl substituent^9^, an increasing low-field shift is seen in the order Me < Et < *i*Pr < *t*Bu (refs. ^13,28^). This trend is also evident for the halogen-substituted silylium ions **8** (except for X = F) and becomes increasingly pronounced in the order Cl < Br < I (Fig. 2c, left). While in the iodosilylium ion series, the ^29^Si NMR chemical shifts cover the broadest spectral range (*δ*(^29^Si) = 94–41 ppm), this alkyl effect is not reflected for the fluorine-substituted silylium ions, and all ^29^Si NMR chemical shifts are essentially identical (Δ*δ*(^29^Si) < 4 ppm). However, a correlation is again observable for the ^1^*J*(Si,F) coupling constant, which increases steadily in the order Me < Et < *i*Pr < *t*Bu (table in Fig. 2b, entries 1–4). The large ^1^*J*(Si,F) values between 369 Hz and 411 Hz are markedly higher than those in neutral fluorosilanes, which typically fall in the range of 275–290 Hz (for example, ^1^*J*(Si,F) = 275 Hz for Me_3_SiF)^29^. The ^1^*J*(Si,F) coupling constant of zwitterionic [*t*Bu_2_FSi(HCB_11_H_5_Br_6_)] (**8ad**) also exceeds that of *t*Bu_2_FSiBr by 65 Hz (^1^*J*(Si,F) = 410.5 versus 345.6 Hz)^30^. The ^19^F NMR chemical shifts are also more shielded in the same order (*δ*(^19^F) = −116.7 ppm for **8aa**, −129.0 ppm for **8ab**, −137.0 ppm for **8ac** and −142.0 ppm for **8ad**).

The experimental ^29^Si NMR chemical shifts were further corroborated by density functional theory (DFT) calculations (table in Fig. 2b and Fig. 2c, right). To understand the larger dependence on the alkyl substituent for the heavier halogen-substituted silylium ions, the chemical shifts were computed at adequate, that is, relativistic quantum-chemical levels (see Supplementary Information for the computational details). As expected^15^, computations on the free gas-phase cations resulted in far too large shifts, and inclusion of the interaction with the carborate counteranion is crucial to obtain shifts in the right range^31^ (Extended Data Table 1). It should be mentioned here that the silylium ion can exhibit different binding modes with the carborate cluster^28,32^ (for an in-depth analysis, see Supplementary Information Section 7). Moreover, neglecting the effects of spin–orbit coupling (SOC) at scalar-relativistic one-component (1c) levels leads to an overestimation of the shifts. In particular, the 1c calculations fail to properly describe the trends observed when going to the heavier halogen substituents (Fig. 2c, right, and Extended Data Table 2). Moderate SOC effects for X = F and Cl can be attributed mostly to the interaction of the silicon centre with the bromine atom of the carborate anion, while the larger SOC effects for the heavier halogen substituents align closely with the known SOC shielding effects of covalently bound halogen substituents in many other systems, the so-called normal halogen dependence^33,34^.

Importantly, the increasing curvature of the experimental shift plot towards X = Br and I with decreasing electron-donating ability of the alkyl substituent is reproduced only when SOC effects are included (Fig. 2c). It is known that such SOC NMR shifts arise through a Fermi-contact mechanism that transfers SOC-induced spin polarization from the heavy substituent responsible for SOC to the respective NMR nucleus^33,34^. Obviously, the SOC effects increase with the atomic number (that is, nuclear charge) of the halogen. In addition, a better electron-donating ability of the alkyl substituent with Me < Et < *i*Pr < *t*Bu strengthens the covalency of the Si–C bonds and, thus, diminishes the covalency of the Si–X bond. This is clearly evident from the natural population analysis (NPA) charges and from the composition of the relevant natural localized molecular orbitals (Extended Data Table 3), where along this series of alkyl substituents a given Si–X bond becomes increasingly polarized towards the halogen atom. While this effect looks rather moderate, such small changes are known to be sufficient to reduce the effectiveness of the mentioned Fermi-contact mechanism of the SOC NMR shifts^34^. As a result, the shielding SOC effects for a given halogen tend to be largest for Alk = Me and smallest for Alk = *t*Bu (for example, Δ*δ*(^29^Si) = −70 ppm for Me_2_ISi^+^ and Δ*δ*(^29^Si) = −40 ppm for *t*Bu_2_ISi^+^; Fig. 2c, right, and Extended Data Table 2). The known dependence of the SOC effects on the bond covalency together with the interplay between Si–C and Si–X bond covalency thus largely explain the interesting experimental findings.

The monotonous increase of the computed ^29^Si chemical shifts in the absence of spin–orbit contributions (see values on the dashed lines in Fig. 2c, right) correlates with decreasing HOMO–LUMO gaps (the energy difference between the highest occupied and lowest unoccupied molecular orbitals; Extended Data Table 4) and may reflect, to some extent, decreasing halogen–silicon π-bonding along this series. The more electron-donating alkyl substituents decrease the gaps and therefore contribute to larger shifts at the scalar relativistic level, that is, without spin–orbit effects.

### Crystallographic characterization

**Fig. 3:**
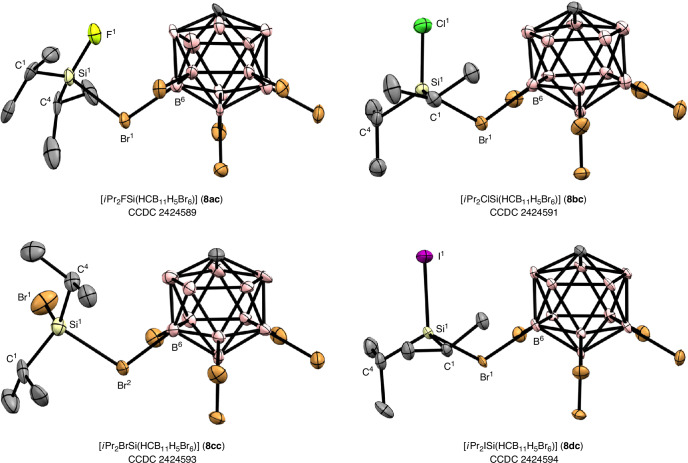
Solid-state molecular structures of halogen-substituted silylium carborates. Thermal ellipsoids are shown at the 50% probability level. All hydrogen atoms are omitted for clarity.

For the isopropyl substituent, we succeeded in the crystallographic characterization of [*i*Pr_2_XSi(HCB_11_H_5_Br_6_)] for all four halogen atoms (X = F, Cl, Br and I). Single crystals suitable for X-ray diffraction analysis were obtained from a solution of the corresponding silylium salt in *ortho*-difluorobenzene (for X = F, Br and I) or *ortho*-dichlorobenzene (for X = Cl) by vapour diffusion with *n*-pentane and *n*-hexane, respectively. All molecular structures show the same general features as the reported tertiary trialkyl-^7,9,28^ and secondary hydrogen-substituted^15^ silylium carborates (Fig. 3). The positively charged silicon atom is coordinated by one of the bromine atoms from the pentagonal belt of the icosahedral carborate cluster. This interaction leads to a distorted tetrahedral coordination environment at the silicon atom. The key bond lengths and angles of the halosilylium ions **8ac**–**dc** are summarized in Table 1. For comparison, the geometric parameters for [*i*Pr_2_HSi(HCB_11_H_5_Br_6_)] (**5c**) and [*i*Pr_3_Si(HCB_11_H_5_Br_6_)] (**2c**)^7,9^ are also listed. As expected, the Si–X bond length increases in the order F < Cl < Br < I. The Si–Br bond distance to the coordinating carborate anion decreases from **8ac** (2.439 Å) to **8bc** (2.417 Å) and **8cc** (2.393 Å), but is longer again for **8dc** (2.436 Å). Although all Si–Br bonds are shorter than in trialkyl-substituted silylium carborate **2c**, they are still substantially elongated compared with neutral bromosilane Me_3_SiBr (2.235 Å)^35^ and dibromosilane (F_5_C_2_)_2_SiBr_2_ (2.154 Å)^36^. The bromosilylium salt [*i*Pr_2_BrSi(HCB_11_H_5_Br_6_)] (**8cc**) serves as a good reference for assessing the nature of the different Si–Br bonds, as the silicon atom combines both a coordinating and a bound bromine atom. While the former Si–Br bond length is 7% longer, the latter is 3% shorter than in Me_3_SiBr. This reflects the weakly coordinating nature of the anion and the cationic character of the silylium ion. In addition to the shortened Si–Br bond to the counteranion, the halosilylium ions **8ac**–**dc** exhibit an increasing degree of pyramidalization compared with trialkylsilylium carborate **2c**, which is indicative of a higher bromonium ion character arising from the electron-withdrawing effect of the halogen substituent. It should be noted, however, that the Si–Br bond lengths to the carborate anion (2.393–2.439 Å) as well as the sum of the three angles around the silicon atom as a measure of pyramidalization (∑R–Si–R = 344–346°) fall within a relatively narrow range and cannot be correlated with the Lewis acidity of the individual halosilylium ion (see ‘Lewis acidity’ section). A trend is seen only for each Si–C bond to the isopropyl substituent, which lengthens steadily from fluorine to iodine within the halosilylium ion series.

**Table 1:** Comparison of selected bond lengths and angles of silylium carborates [*i*Pr_2_XSi(HCB_11_H_5_Br_6_)]

	2c (X = *i*Pr)^a^	5c (X = H)	8ac (X = F)	8bc (X = Cl)	8cc (X = Br)	8dc (X = I)
Si–X (Å)	–	1.81(5)	1.60(1)	2.033(2)	2.179(3)	2.398(2)
Si–C_*i*Pr_ (Å)	1.799(35) 1.860(27) 1.908(27)	1.850(4) 1.863(6)	1.84(2) 1.85(2)	1.843(5) 1.850(5)	1.861(9) 1.865(8)	1.871(8) 1.871(9)
Si–Br_carborate_ (Å)	2.479(9)	2.432(1)	2.439(6)	2.417(1)	2.393(3)	2.436(2)
B–Br_coord_ (Å)	2.05(3)	2.013(4)	2.02(2)	2.009(5)	2.015(8)	2.020(6)
Si–Br–B (°)	114.7(7)	106.6(1)	109.4(7)	115.6(1)	111.2(3)	116.7(2)
C_*i*Pr_–Si–X (°)	–	111(2) 112(2)	109.9(9) 112.4(9)	112.2(2) 112.3(2)	110.8(3) 112.4(3)	112.5(2) 113.8(2)
C_*i*Pr_–Si–C_*i*Pr_ (°)	111.2(14) 119.6(13) 120.2(12)	121.2(2)	124(1)	120.0(2)	122.7(4)	118.1(3)
∑R–Si–R (°)	351	344	346	345	346	344

^a^The data for [*i*Pr_3_Si(HCB_11_H_5_Br_6_)] (**2c**) are taken from refs. ^7,9^.

### Lewis acidity

**Fig. 4:**
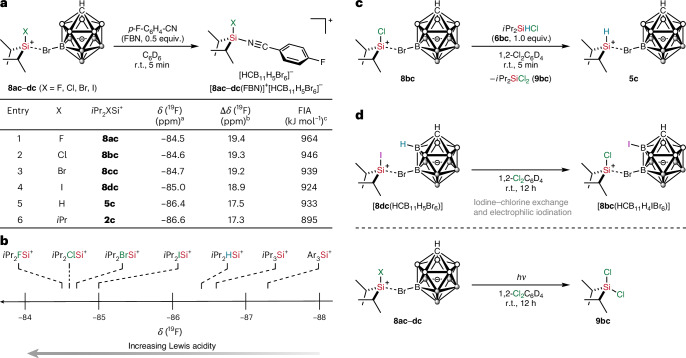
Lewis acidity and reactivity of halogen-substituted silylium ions. **a**, Experimental and theoretical evaluation of the Lewis acidity based on Müller’s FBN scale and calculated fluoride ion affinities. **b**, The FBN acidity scale for different silylium ions in benzene. The ^19^F NMR chemical shift for Ar_3_Si^+^ (Ar = pentamethylphenyl) is taken from ref. ^22^. **c**, Chloride abstraction from a chlorosilane by a halogen-substituted silylium ion provides access to hydrogen-substituted silylium ions. **d**, Decomposition pathways of the halosilylium carborates. ^a19^F NMR chemical shifts of the silylnitrilium carborates [*i*Pr_2_XSi(FBN)]^+^[HCB_11_H_5_Br_6_]^−^ determined from the ^19^F NMR spectrum (471 MHz, 298 K) in C_6_D_6_. ^b^Deshielding of the ^19^F NMR chemical shift relative to free FBN (*δ*(^19^F) = −103.9 ppm). ^c^Directly computed gas-phase fluoride ion affinities at the LH20t-D4/def2-TZVPD//BP86-D4/def2-TZVP level. For a comprehensive list and a comparison with the anchor-point approach using OCF_2_, see Extended Data Table 5.

In an attempt to experimentally correlate the relative Lewis acidities of the halogen-substituted silylium ions, we initially chose the established Gutmann–Beckett method using triethylphosphine oxide^37^. However, it turned out that this analysis is not compatible with the exceptional Lewis acidity of the halosilylium ions, and multiple signals were observed in the ^31^P{^1^H} NMR spectrum. We therefore turned to the Lewis acidity scale introduced by Müller and co-workers using *para*-fluorobenzonitrile (FBN) as Lewis base^38^. This NMR probe indeed proved to be suitable, and immediate Lewis adduct formation with the silylium ion led to clean generation of the corresponding silylnitrilium carborate (Fig. 4a). The addition of substoichiometric amounts of FBN ensured that the formation of any pentacoordinate silicon complexes (silanium ions) by double nitrile coordination was prevented. All silylnitrilium ions were fully characterized by multinuclear NMR spectroscopy (Supplementary Table 1 in Supplementary Information Section 5). The ^19^F NMR chemical shifts are summarized in the table of Fig. 4a. The degree of deshielding of the ^19^F NMR chemical shift relative to free FBN (*δ*(^19^F) = −103.9 ppm in C_6_D_6_) is a measure for scaling the relative Lewis acidities of the corresponding silylium ions. The results depicted in Fig. 4b rank the halosilylium ions **8ac**–**dc** among the strongest isolable silicon Lewis superacids so far^39^. They are stronger Lewis acids than the corresponding hydrogen-, trialkyl- and triaryl-substituted congeners. The decreasing Lewis acidity from the fluorine to iodine substituent in *i*Pr_2_XSi^+^ is in line with the observed trend of intramolecularly halo-stabilized silyl cations reported by Müller and co-workers^22^.

The Lewis acidity ranking of the different silylium ions established with the FBN scale was further supported by DFT calculations of the FIAs, a generally accepted more direct thermodynamic index for Lewis acidity^39^. The FIA is defined as the negative of the enthalpy of the reaction LA + F^−^ → LA–F^−^. Some of us have recently shown that the widely used anchor-point methods, which are based on isodesmic reactions for Lewis acids with a known FIA (for example, of F_2_CO) to avoid the calculation of free fluoride, are an improvement only when inadequate density functionals and/or insufficient basis sets lacking diffuse functions are used^40^. This is also corroborated for the present silylium ions, as supported by a comparison of directly computed and anchor-based FIAs for the full set of silylium ions (Extended Data Table 5). The directly computed FIAs for the different isopropyl-substituted silylium ions are summarized within the table of Fig. 4a. As expected, substitution of hydrogen by fluorine or chlorine enhances the Lewis acidity for a given silylium ion, while bromine or iodine substitution has a much smaller effect. In general, the electron-donating ability of the hydrogen and alkyl substituents increases along the series H < Me < Et < *i*Pr < *t*Bu, and this trend is also reflected in the computed FIAs (as well as in the NPA charges; Extended Data Table 3). Strikingly, Me_2_FSi^+^ has the largest FIA only just behind H_3_Si^+^ (Extended Data Table 5). Overall, the data clearly confirm that such silyl cations are among the strongest known Lewis acids.

### Reactivity

The relative order of Lewis acidity was further tested experimentally by a competition experiment (Fig. 4c). Treatment of chlorosilylium carborate **8bc** with equimolar amounts of *i*Pr_2_SiHCl (**6bc**) resulted in immediate chloride abstraction from **6bc** and the formation of hydrogen-substituted silylium salt [*i*Pr_2_HSi(HCB_11_H_5_Br_6_)] (**5c**) along with *i*Pr_2_SiCl_2_ (**9bc**), as monitored by NMR spectroscopy. The reaction outcome can be rationalized by the higher chloride ion affinity of *i*Pr_2_ClSi^+^ compared with *i*Pr_2_HSi^+^. At the same time, this result also provides an explanation why the established Corey reaction cannot be used to prepare halogen-substituted silylium carborates (Fig. 2a, top). As hydride abstraction from *i*Pr_2_SiHCl (**6bc**) by the trityl cation is slow, the incipiently generated chlorosilylium ion *i*Pr_2_ClSi^+^ immediately abstracts a chloride from the starting silane **6bc**, eventually leading to the selective formation of [*i*Pr_2_HSi(HCB_11_H_5_Br_6_)] (**5c**). All halosilylium carborates **8** are crystalline salts stable under inert atmosphere for several weeks at −30 °C, but slow decomposition of any halogen-substituted silylium ion is observed in *ortho*-dichlorobenzene solution (Fig. 4d, top; for an analysis of this decomposition pathway, see Supplementary Fig. 5). Exposure to sunlight led to even faster decomposition of the halosilylium ions in solution, now resulting in the formation of the corresponding dichlorosilane (Fig. 4d, bottom).

## Conclusions

Previously elusive halogen-substituted silylium ions are not only exceptionally strong Lewis acids of academic interest, but also highly relevant in synthetic chemistry. Examples include their intermediacy in the halodealkylation of waste material from the Müller–Rochow process^16,17^ and their long-sought role as reagents in the hydrodefluorination of PFAS, exploiting their enormous fluoridophilicity^25,26^. The present work discloses a reliable synthetic access to the whole family of these superreactive cations in the form of their counteranion-stabilized carborate salts [Alk_2_XSi(HCB_11_H_5_Br_6_)] (X = F, Cl, Br or I). This was achieved by our dehydrogenative (LG = H) or dephenylative (LG = Ph) protolysis^13,15^ of the corresponding halosilanes R_2_XSi–LG using Reed’s superacidic benzenium salt^12^ [H(C_6_H_6_)]^+^[HCB_11_H_5_Br_6_]^−^. The molecular structures have been characterized by X-ray diffraction analysis with the fluorine congener being particularly fragile and delicate to handle. DFT calculations lend support to the understanding of the stabilization of these superacids, and the agreement between the measured and the computed ^29^Si NMR chemical shifts is excellent when SOC effects are taken into consideration. An assessment of the Lewis acidity using Müller’s FBN method^38^ and calculated FIAs^40^ confirm that halogen-substituted silylium ions are among the strongest Lewis acids ever isolated. This work opens the door to unexplored reactivity in catalysis with superelectrophilic silicon reagents.

## Methods

### General procedure for the generation of halogen-substituted silylium carborates

In a high-quality glovebox (O_2_, H_2_O < 1.0 ppm), the indicated hydrosilane **6** or phenylsilane **7** (1.0 equiv.) was added to a suspension of [H(C_6_H_6_)]^+^[HCB_11_H_5_Br_6_]^−^ (1.0 equiv.) in C_6_D_6_ (0.2 ml). After stirring the reaction mixture for 2–3 h at room temperature, *n*-hexane (0.4 ml) was added. The suspension was filtered, and the residue was washed with additional *n*-hexane (3 × 0.2 ml) and dried for 10 min under high vacuum (~10^−3^ mbar). The halogen-substituted silylium carborates **8** were obtained as white solids, which can be stored for several weeks at −30 °C in the glovebox.

## Online content

Any methods, additional references, Nature Portfolio reporting summaries, source data, extended data, supplementary information, acknowledgements, peer review information; details of author contributions and competing interests; and statements of data and code availability are available at 10.1038/s41557-025-01880-2.

## Supplementary information

Supplementary Information Supplementary Figs. 1–221, Tables 1–8, experimental procedures, characterization data, crystallographic data and computational details.

Supplementary Data Cartesian coordinates for all calculated structures.

## Data Availability

The data supporting the findings of this study are available within the Article and its Supplementary Information and are also available from the corresponding authors upon request. Crystallographic data for the structures reported in this Article have been deposited at the Cambridge Crystallographic Data Centre, under deposition numbers CCDC 2424587 (**5c**), 2424589 (**8ac**), 2424591 (**8bc**), 2424593 (**8cc**), 2424594 (**8dc**) and 2424600 (**8ad**). Copies of the data can be obtained free of charge via https://www.ccdc.cam.ac.uk/structures/.

## Extended data

**Extended Data Table 1:** Comparison of computed ^29^Si chemical shifts for free gas-phase halosilylium ions and their adducts with the counteranion [HCB_11_H_5_Br_6_]^−^ at different levels of theory.^a^

	1c/PBE0	4c/PBE0	1c/PBE0	2c/PBE0	4c/PBE0	4c/PBE0^c^	exp.
coord.^b^	−	−	A^−^	A^−^	A^−^	A^−^	
*i*Pr_2_FSi^+^	178.1	173.4	76.6	67.4	67.7	47.0	47.0
*i*Pr_2_ClSi^+^	263.2	253.8	105.5	93.1	94.2	73.4	74.3
*i*Pr_2_BrSi^+^	304.0	272.2	122.6	99.8	100.0	79.3	79.3
*i*Pr_2_ISi^+^	358.2	286.8	141.9	102.4	102.6	81.9	78.8

^a^Chemical shifts in ppm relative to tetramethylsilane.

^b^Gas-phase vs. adduct with the carborate counteranion (conformer **A** was used; see Supplementary Fig. 12).

^c^Referenced to tetramethylsilane indirectly via the secondary standard *i*Pr_2_FSi^+^ to correct for systematic errors. This leads to a constant low-frequency shift by ca. –20.7 ppm.

**Extended Data Table 2:** Comparison of computed ^29^Si chemical shifts for silylium carborates [Alk_2_XSi(HCB_11_H_5_Br_6_)] at the scalar relativistic (1c) and at the two-component spin-orbit (2c) PBE0/x2c-TZVPall-2c level of theory.^a,b^

	1c	2c	exp.		1c	2c	exp.
Me_2_FSi^+^	56.3	45.2	49.3	Et_2_FSi^+^	56.8	46.0	50.6
Me_2_ClSi^+^	75.3	59.6	66.2	Et_2_ClSi^+^	82.3	68.2	72.5
Me_2_BrSi^+^	89.0	55.1	62.3	Et_2_BrSi^+^	98.3	70.3	73.5
Me_2_ISi^+^	101.0	31.1	41.7	Et_2_ISi^+^	115.2	60.7	64.3
*i*Pr_2_FSi^+^	55.0	46.8	47.0	*t*Bu_2_FSi^+^	53.7	46.4	47.6
*i*Pr_2_ClSi^+^	83.2	73.2	74.3	*t*Bu_2_ClSi^+^	86.8	77.7	80.5
*i*Pr_2_BrSi^+^	100.2	76.9	79.3	*t*Bu_2_BrSi^+^	105.6	85.3	88.5
*i*Pr_2_ISi^+^	118.8	70.8	78.8	*t*Bu_2_ISi^+^	126.8	86.6	93.5

^a^Chemical shifts in ppm relative to tetramethylsilane.

^b^Conformer **B** of the adduct with the carborate counteranion was used (see Supplementary Fig. 12).

**Extended Data Table 3:** NPA charges and atomic contributions (in %) to the Si–X bond of silylium carborates [Alk_2_XSi(HCB_11_H_5_Br_6_)] based on an NLMO analysis (LH20t/def2-TZVPD).^a^

	NPA charge		atomic contribution to Si–X NLMO	
	Si	X	Si	X
Me_2_FSi^+^	1.89	−0.64	12.0	87.4
Me_2_ClSi^+^	1.49	−0.31	26.3	73.0
Me_2_BrSi^+^	1.39	−0.22	30.9	68.3
Me_2_ISi^+^	1.29	−0.11	36.7	62.4
Et_2_FSi^+^	1.92	−0.64	11.4	87.7
Et_2_ClSi^+^	1.51	−0.32	25.8	73.5
Et_2_BrSi^+^	1.42	−0.23	30.3	68.9
Et_2_ISi^+^	1.32	−0.11	35.9	63.0
*i*Pr_2_FSi^+^	1.97	−0.65	11.1	88.0
*i*Pr_2_ClSi^+^	1.56	−0.32	25.4	73.8
*i*Pr_2_BrSi^+^	1.46	−0.22	29.9	69.2
*i*Pr_2_ISi^+^	1.37	−0.10	35.7	63.1
*t*Bu_2_FSi^+^	2.13	−0.68	9.3	89.6
*t*Bu_2_ClSi^+^	1.69	−0.34	23.0	75.9
*t*Bu_2_BrSi^+^	1.60	−0.24	27.3	71.5
*t*Bu_2_ISi^+^	1.49	−0.10	33.1	65.3

^a^Conformer **B** of the adduct with the carborate counteranion was used (see Supplementary Fig. 12).

**Extended Data Table 4:** HOMO–LUMO gaps (in eV) for free halosilylium ions and their adducts with the counteranion [HCB_11_H_5_Br_6_]^−^ at the one-component X2C-PBE0/x2c-TZVPall-2c level of theory.^a^

	free silylium ion	carborate adduct
Me_2_FSi^+^	7.77	5.56
Me_2_ClSi^+^	6.84	5.53
Me_2_BrSi^+^	6.16	5.42
Me_2_ISi^+^	5.23	5.17
Et_2_FSi^+^	6.44	5.56
Et_2_ClSi^+^	6.03	5.50
Et_2_BrSi^+^	5.78	5.39
Et_2_ISi^+^	5.13	5.15
*i*Pr_2_FSi^+^	6.10	5.48
*i*Pr_2_ClSi^+^	5.67	5.42
*i*Pr_2_BrSi^+^	5.48	5.36
*i*Pr_2_ISi^+^	5.02	5.16
*t*Bu_2_FSi^+^	5.92	5.42
*t*Bu_2_ClSi^+^	5.48	5.34
*t*Bu_2_BrSi^+^	5.33	5.28
*t*Bu_2_ISi^+^	4.97	5.13

^a^Conformer **B** of the adduct with the carborate counteranion was used (see Supplementary Fig. 12).

**Extended Data Table 5:** Computed gas-phase fluoride ion affinities (in kJ mol^−1^) for various silylium ions at the LH20t-D4/def2-TZVPD//BP86-D4/def2-TZVP level of theory calculated directly (FIA^direct^) and with the anchor-point approach using OCF_2_ (FIA^anchored^)

	FIA^direct^	FIA^anchored^
Me_2_HSi^+^	976	972
Me_2_FSi^+^	1004	999
Me_2_ClSi^+^	981	977
Me_2_BrSi^+^	972	968
Me_2_ISi^+^	956	952
Et_2_HSi^+^	958	954
Et_2_FSi^+^	983	979
Et_2_ClSi^+^	965	961
Et_2_BrSi^+^	958	954
Et_2_ISi^+^	940	936
*i*Pr_2_HSi^+^	933	929
*i*Pr_2_FSi^+^	964	960
*i*Pr_2_ClSi^+^	946	941
*i*Pr_2_BrSi^+^	939	934
*i*Pr_2_ISi^+^	924	920
*t*Bu_2_HSi^+^	923	918
*t*Bu_2_FSi^+^	951	947
*t*Bu_2_ClSi^+^	934	930
*t*Bu_2_BrSi^+^	927	923
*t*Bu_2_ISi^+^	914	910
*t*Bu_3_Si^+^	889	885
*t*Bu_2_HSi^+^	923	918
*t*BuH_2_Si^+^	972	968
H_3_Si^+^	1079	1075
Me_3_Si^+^	938	933
Et_3_Si^+^	916	912
*i*Pr_3_Si^+^	895	890
*t*Bu_3_Si^+^	889	885
