# Supplementary material for: Isolation of halogen-substituted silylium ions
Source: Nat Chem. 2025 Jul 31;17(11):1666–72. doi: 10.1038/s41557-025-01880-2 (PMC12580324; doi:10.1038/s41557-025-01880-2)
Supplement: Supplementary file 1 — Supplementary Figs. 1–221, Tables 1–8, experimental procedures, characterization data, crystallographic data and computational details. [file 41557_2025_1880_MOESM1_ESM.pdf]

nature chemistry

Supplementary information

https://doi.org/10.1038/s41557-025-01880-2

Isolation of halogen-substituted silylium ions

In the format provided by the authors and unedited

Table of Contents

**1**	**General information**	**S3**
**2**	**Synthesis of Reed's Ion-Like Carborate Salts**	**S5**
2.1	Trityl carborate $[Ph_3C]^+[HCB_{11}H_5Br_6]^-$	S5
2.2	Benzenium carborate $[H(C_6H_6)]^+[HCB_{11}H_5Br_6]^-$	S6
**3**	**Synthesis of silanes**	**S7**
3.1	Fluorodimethyl(phenyl)silane (**7aa**)	S8
3.2	Bromodimethyl(phenyl)silane (**7ca**)	S9
3.3	Iododimethyl(phenyl)silane (**7da**)	S10
3.4	Diethylfluoro(phenyl)silane (**7ab**) and ethyldifluoro(phenyl)silane (**S3**)	S11
3.5	Chlorodiethylsilane (**6bb**)	S13
3.6	Bromodiethylsilane (**6cb**)	S14
3.7	Diethyliodosilane (**6db**)	S15
3.8	Chlorodiisopropyl(phenyl)silane (**7bc**)	S16
3.9	Fluorodiisopropyl(phenyl)silane (**7ac**)	S17
3.10	Chlorodiisopropylsilane (**6bc**)	S18
3.11	Fluorodiisopropylsilane (**6ac**)	S19
3.12	Diisopropylsilane (**3c**)	S20
3.13	Bromodiisopropylsilane (**6cc**)	S21
3.14	Diisopropyliodosilane (**6dc**)	S22
3.15	Trifluoro(phenyl)silane (**S8**)	S23
3.16	Di-*tert*-butylfluoro(phenyl)silane (**7ad**)	S24
3.17	Di-*tert*-butylchlorosilane (**6bd**)	S25
3.18	Bromodi-*tert*-butylsilane (**6cd**)	S26
3.19	Di-*tert*-butyliodosilane (**6dd**)	S27
3.20	Difluoromethyl(phenyl)silane (**S11**)	S28
3.21	Dichloroisopropylsilane (**S12**)	S29
3.22	*tert*-Butyldichloro(phenyl)silane (**S13**)	S30
3.23	*tert*-Butyldifluoro(phenyl)silane (**S14**)	S31
**4**	**Synthesis of silylium ions**	**S32**
4.1	[*i*$Pr_2HSi(HCB_{11}H_5Br_6)$] (**5c**)	S32
4.2	General procedure for the generation of halogen-substituted silylium carborates (**GP 1**)	S33
4.2.1	[$Me_2FSi(HCB_{11}H_5Br_6)$] (**8aa**)	S34
4.2.2	[$Et_2FSi(HCB_{11}H_5Br_6)$] (**8ab**)	S35

4.2.3 [*i*$Pr_2FSi(HCB_{11}H_5Br_6)$] (**8ac**) S36
4.2.4 [*t*$Bu_2FSi(HCB_{11}H_5Br_6)$] (**8ad**) S37
4.2.5 [$Me_2ClSi(HCB_{11}H_5Br_6)$] (**8ba**) S38
4.2.6 [$Et_2ClSi(HCB_{11}H_5Br_6)$] (**8bb**) S39
4.2.7 [*i*$Pr_2ClSi(HCB_{11}H_5Br_6)$] (**8bc**) S40
4.2.8 [*t*$Bu_2ClSi(HCB_{11}H_5Br_6)$] (**8bd**) S41
4.2.9 [$Me_2BrSi(HCB_{11}H_5Br_6)$] (**8ca**) S42
4.2.10 [$Et_2BrSi(HCB_{11}H_5Br_6)$] (**8cb**) S43
4.2.11 [*i*$Pr_2BrSi(HCB_{11}H_5Br_6)$] (**8cc**) S44
4.2.12 [*t*$Bu_2BrSi(HCB_{11}H_5Br_6)$] (**8cd**) S45
4.2.13 [$Me_2ISi(HCB_{11}H_5Br_6)$] (**8da**) S46
4.2.14 [$Et_2ISi(HCB_{11}H_5Br_6)$] (**8db**) S47
4.2.15 [*i*$Pr_2ISi(HCB_{11}H_5Br_6)$] (**8dc**) S48
4.2.16 [*t*$Bu_2ISi(HCB_{11}H_5Br_6)$] (**8dd**) S50

5 Determination of the Lewis acidity using Müller's FBN method S51
5.1 General procedure for the generation of silylnitrilium carborates with *p*-fluorobenzonitrile (**GP 2**) S51
5.1.1 [*i*$Pr_2FSi(FBN)]^+[HCB_{11}H_5Br_6]^-$ ([**8ac**$(FBN)]^+[HCB_{11}H_5Br_6]^-$) S52
5.1.2 [*i*$Pr_2ClSi(FBN)]^+[HCB_{11}H_5Br_6]^-$ ([**8bc**$(FBN)]^+[HCB_{11}H_5Br_6]^-$) S53
5.1.3 [*i*$Pr_2BrSi(FBN)]^+[HCB_{11}H_5Br_6]^-$ ([**8cc**$(FBN)]^+[HCB_{11}H_5Br_6]^-$) S54
5.1.4 [*i*$Pr_2ISi(FBN)]^+[HCB_{11}H_5Br_6]^-$ ([**8dc**$(FBN)]^+[HCB_{11}H_5Br_6]^-$) S55
5.1.5 [*i*$Pr_2HSi(FBN)]^+[HCB_{11}H_5Br_6]^-$ ([**5c**$(FBN)]^+[HCB_{11}H_5Br_6]^-$) S56
5.1.6 [*i*$Pr_3Si(FBN)]^+[HCB_{11}H_5Br_6]^-$ ([**2c**$(FBN)]^+[HCB_{11}H_5Br_6]^-$) S57

6 Crystallographic data S58
6.1 Molecular structure of [*i*$Pr_2HSi(HCB_{11}H_5Br_6)$] (**5c**) S58
6.2 Molecular structure of [*i*$Pr_2FSi(HCB_{11}H_5Br_6)$] (**8ac**) S59
6.3 Molecular structure of [*t*$Bu_2FSi(HCB_{11}H_5Br_6)$] (**8ad**) S60
6.4 Molecular structure of [*i*$Pr_2ClSi(HCB_{11}H_5Br_6)$] (**8bc**) S61
6.5 Molecular structure of [*i*$Pr_2BrSi(HCB_{11}H_5Br_6)$] (**8cc**) S62
6.6 Molecular structure of [*i*$Pr_2ISi(HCB_{11}H_5Br_6)$] (**8dc**) S63

7 Computational details S64

8 Analytical spectra S67

9 References S275

1 General information

All reactions were performed in flame-dried glassware using an MBraun glove box (argon atmosphere, O_2 < 1.0 ppm, H_2O < 1.0 ppm) or conventional Schlenk techniques under a static pressure of nitrogen gas unless otherwise stated. All given temperatures refer to external bath temperatures. Standard solvents and reagents were obtained from commercial suppliers and used as received unless otherwise stated. Liquids and solutions were transferred via syringes. Dry *n*-pentane, *n*-hexane, CH_2Cl_2, and benzene were obtained from an MBraun solvent purification system (SPS-800), degassed by three freeze-pump-thaw cycles, and stored in a glovebox over thermally activated 4 Å molecular sieves. Technical grade solvents for extraction or chromatography (CH_2Cl_2, cyclohexane, and *n*-pentane) were distilled prior to use. Et_2O and THF were dried over potassium/benzophenone and freshly distilled prior to use. Benzene-d_6 (C_6D_6), *ortho*-dichlorobenzene-d_4 (1,2-$C_6D_4Cl_2$), and dichloromethane-d_2 (CD_2Cl_2) were degassed by three freeze-pump-thaw cycles and stored in a glovebox over thermally activated 4 Å molecular sieves. $CDCl_3$ was obtained from commercial suppliers and used as received. Trityl carborate $[Ph_3C]^+[HCB_{11}H_5Br_6]^-$ and benzenium carborate $[H(C_6H_6)]^+[HCB_{11}H_5Br_6]^-$ were prepared according to reported procedures (see Section 2).[11,12] Chlorodimethyl(phenyl)silane, dichlorodiisopropylsilane, diethylsilane, di-*tert*-butylsilane, and lithium aluminium hydride were obtained from ABCR and used as received. Ethylmagnesium bromide, magnesium, potassium fluoride, and *tert*-butyllithium were obtained from Acros Organics and used as received. Dimethyl(phenyl)silane was obtained from BLDpharm and used as received. Bromine, bromoethane, 2-chloropropane, dichloromethyl(phenyl)silane, iodine, phenyllithium, and trichlorosilane were obtained from Sigma-Aldrich and used as received. Trichloro(phenyl)-silane was obtained from TCI and used as received. Potassium bifluoride was obtained from Thermo Fisher Scientific and used as received. Infrared (IR) spectra were recorded on an Agilent Technologies Cary 630 FT-IR spectrometer equipped with an ATR unit, and the signals are reported in wavenumbers (cm^{-1}). The ATR crystal was directly coated with the substance prior to the measurement and the absorption intensity is indicated by the following abbreviations: vs = very strong, s = strong, m = medium, w = weak, br = broad. ^1H, ^2H, ^{11}B, ^{13}C, ^{19}F, and ^{29}Si NMR spectra were recorded in C_6D_6, 1,2-$C_6D_4Cl_2$, CD_2Cl_2, or $CDCl_3$ on Bruker AV500 and Bruker AV700 instruments, respectively. For silylium carborates, the J Young NMR tube was covered with aluminum foil to protect the sample from light until it was subjected to the NMR spectrometer. Chemical shifts are reported in parts per million (ppm) and are referenced to the residual solvent resonance as the internal standard (C_6D_5H: δ = 7.16 ppm for ^1H and ^2H NMR and C_6D_6: δ = 128.06 ppm for ^{13}C NMR; $CHCl_3$: δ = 7.28 ppm for ^1H NMR and $CDCl_3$: δ = 77.00 ppm for ^{13}C NMR; $CDHCl_2$: δ = 5.32 ppm for ^1H NMR and CD_2Cl_2: δ = 53.84 ppm for ^{13}C NMR; 1,2-$C_6D_3HCl_2$: δ = 6.94 and 7.20 ppm for ^1H NMR and 1,2-$C_6D_4Cl_2$: δ = 127.1, 130.1, and 132.5 ppm for ^{13}C NMR). ^{11}B, ^{19}F, and ^{29}Si NMR spectra are referenced

in compliance with the unified scale for NMR chemical shifts as recommended by the IUPAC stating the chemical shift relative to BF_3•Et_2O, CCl_3F, or Me_4Si respectively.[41] Data are reported as follows: chemical shift, multiplicity (s = singlet, d = doublet, t = triplet, q = quartet, quint = quintet, sext = sextet, sept = septet, oct = octet, non = nonet, m = multiplet, m_c = centrosymmetric multiplet), coupling constants (Hz) and integration. High resolution mass spectrometry (HRMS) was conducted by the Center of Mass Spectrometry at the Institut für Chemie, Technische Universität Berlin, using an LTQ Orbitrap XL mass analyzer by Thermo Fisher Scientific. Gas-liquid chromatography mass spectrometry (GLC-MS) measurements were conducted on an Agilent Technologies 5975C TAD – GC/MSD-System with electron impact ionization (EI) connected to a fused silica HP-5ms capillary column (length: 30 m, inner diameter: 0.25 mm, thickness of the stationary phase: 0.25 µm). Measurements were performed using the following protocol: Carrier gas: He, injector temperature: 280 °C, detector temperature: 280 °C, flow rate: 4 mL/min, temperature program: starting temperature: 40 °C, heating rate: 10 °C/min, final temperature: 280 °C for 10 min. Data for the single-crystal structure determination were collected with an Agilent SuperNova diffractometer equipped with a CCD area Atlas detector and a mirror monochromator by utilizing Cu-Kα radiation (λ = 1.5418 Å). The following software packages were used: CrysAlisPro for data collection, cell refinement, and data reduction,[42] SHELXS-2018 for structure solution,[43] SHELXL-2018 for structure refinement,[44] and Mercury for graphics.[45]

2 Synthesis of Reed's Ion-Like Carborate Salts

2.1 Trityl carborate $[Ph_3C]^+[HCB_{11}H_5Br_6]^-$

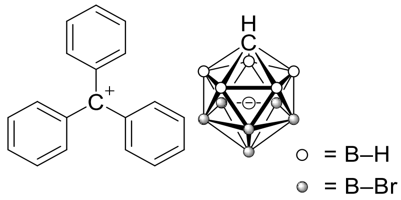

$C_{20}H_{21}B_{11}Br_6$
M = 859.73 g/mol

According to a modified reported procedure,[11] $Cs^+[HCB_{11}H_5Br_6]^-$ (4.10 g, 5.47 mmol, 1.00 equiv) was dissolved in water (270 mL) and the solution was heated to 140 °C. HNO_3 (65%, two drops) was added to achieve a slightly acidic solution. The flask was wrapped in aluminum foil to ensure a low ambient light level. Silver(I) nitrate (1.02 g, 6.00 mmol, 1.10 equiv) was added, and the reaction mixture was stirred for 15 min at 140 °C. The solution was allowed to cool to room temperature, and the resulting white suspension was filtered. The residue was washed with water (3 × 5 mL) and dried under high vacuum (~10^{-3} mbar) for 13 h at 130 °C. The white residue was then transferred into a glovebox, and trityl bromide (2.10 g, 6.50 mmol, 1.19 equiv) and toluene (30 mL) were added. The solution was then again transferred out of the glovebox. Acetonitrile (20 mL) was added, and the reaction mixture was stirred for 67 h at room temperature. The solution was filtered under Schlenk conditions, and the residue was washed with acetonitrile (5 × 5 mL). The solvent was removed under reduced pressure, and the residue was dried under high vacuum (~10^{-3} mbar) for 17 h. The crude product was washed with *n*-hexane (5 × 10 mL) and dried under high vacuum (~10^{-3} mbar) for 48 h at 80 °C. $[Ph_3C]^+[HCB_{11}H_5Br_6]^-$ (4.40 g, 5.12 mmol, 94%) was obtained as a yellow crystalline solid and stored in a glove box.

^{1}H NMR (500 MHz, CD_2Cl_2, 298 K): δ/ppm = 8.30 (t, $^3J_{H,H}$ = 7.4 Hz, 3H, *p*-C*H*-Ph), 7.93 (t, $^3J_{H,H}$ = 7.8 Hz, 6H, *m*-C*H*-Ph), 7.70 (d, $^3J_{H,H}$ = 7.6 Hz, 6H, *o*-C*H*-Ph), 2.56 (s br, 1H, $[\mathit{H}CB_{11}H_5Br_6]^-$), 2.33 (q, $^1J_{H,B}$ = 164.0 Hz, 5H, $[HCB_{11}\mathit{H}_5Br_6]^-$).

^{11}B NMR (160 MHz, CD_2Cl_2, 298 K): δ/ppm = –1.8 (s, 1B), –9.9 (s, 5B), –20.2 (d, $^1J_{B,H}$ = 172.9 Hz, 5B).

^{13}C{^{1}H} NMR (126 MHz, CD_2Cl_2, 298 K): δ/ppm = 211.2 ($Ph_3\mathit{C}^+$), 144.1 (*p*-*C*H-Ph), 143.3 (*o*-*C*H-Ph), 140.4 (*i*-*C*-Ph), 131.2 (*m*-*C*H-Ph), 41.7 ($[H\mathit{C}B_{11}H_5Br_6]^-$).

2.2 Benzenium carborate $[H(C_6H_6)]^+[HCB_{11}H_5Br_6]^-$

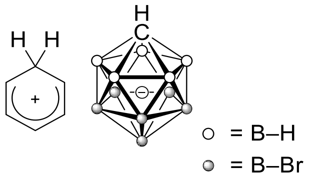

$C_7H_{13}B_{11}Br_6$
M = 695.52 g/mol

According to a reported procedure,[12] $[Ph_3C]^+[HCB_{11}H_5Br_6]^-$ (200 mg, 233 µmol, 1.00 equiv) was suspended in benzene (0.5 mL) in a high-quality glovebox (O_2, H_2O < 1.0 ppm), and triethylsilane (75.0 µL, 54.6 mg, 470 µmol, 2.02 equiv) was added. After stirring the reaction mixture at room temperature for 48 h, *n*-hexane (0.5 mL) was added to the resulting suspension. The precipitate was collected by filtration, washed with *n*-hexane (3 × 0.5 mL) and *n*-pentane (0.5 mL), and dried for 10 min under high vacuum (~10^{-3} mbar). The solid was suspended in benzene (0.5 mL), and triflic acid (20.5 µL, 35.1 mg, 234 µmol, 1.00 equiv) was added. The reaction mixture was vigorously stirred at room temperature for 5 min, and the precipitate was collected by filtration and washed with *n*-pentane (3 × 0.5 mL) and dried for 10 min under high vacuum (~10^{-3} mbar). $[H(C_6H_6)]^+[HCB_{11}H_5Br_6]^-$ (141.6 mg, 204 µmol, 88%) was obtained as a pale yellow solid.

IR (ATR): $\tilde{\nu}$/cm^{-1} = 3094, 3052, 2912, 2804, 2710, 2603, 2593, 1599, 1540, 1481, 1446, 1402, 1326, 1204, 1177, 1158, 1130, 1034, 1000, 989, 950, 931, 858, 810, 748, 718, 678, 637, 614, 609, 579.

^{11}B NMR (160 MHz, C_6D_6, 298 K): δ/ppm = –1.5 (s, 1B), –9.5 (s, 5B), –19.9 (s, 5B). Deuterium incorporation into the carborate counteranion and benzenium ion is observed using deuterated benzene as solvent. NMR spectra in CD_2Cl_2 and 1,2-$Cl_2C_6D_4$ could not be obtained due to the decomposition of $[H(C_6H_6)]^+[HCB_{11}H_5Br_6]^-$ in these solvents.

3 Synthesis of silanes

The precursors for the halosilylium carborates were prepared from commercially available chloro- and hydrosilanes by nucleophilic substitution with suitable organometallic reagents and halogenation using potassium bifluoride, bromine and iodine, respectively.

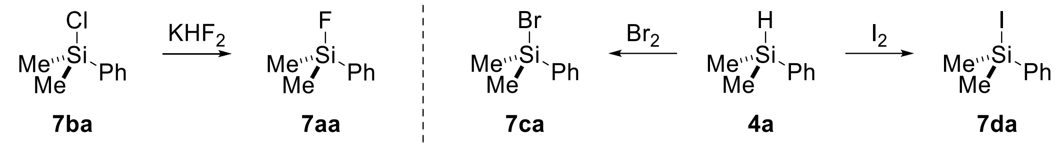

Supplementary Fig. 1. Synthesis of methyl-substituted halosilanes.

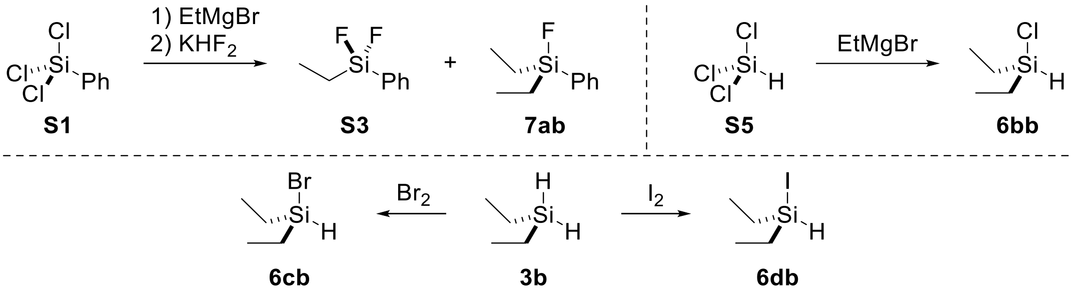

Supplementary Fig. 2. Synthesis of ethyl-substituted halosilanes.

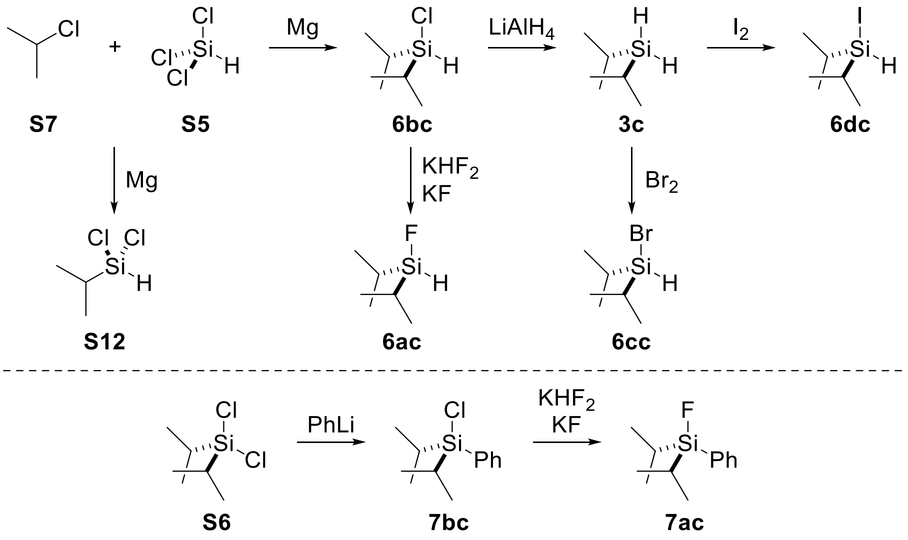

Supplementary Fig. 3. Synthesis of isopropyl-substituted halosilanes.

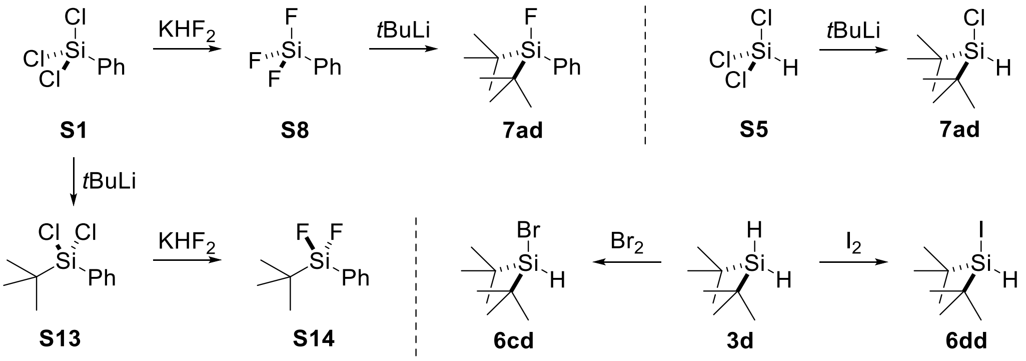

Supplementary Fig. 4. Synthesis of *tert*-butyl-substituted halosilanes.

3.1 Fluorodimethyl(phenyl)silane (**7aa**)

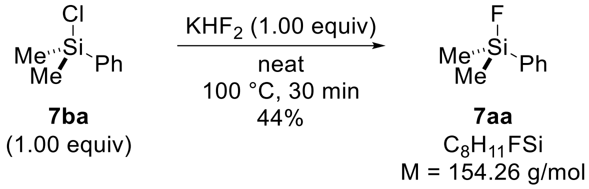

Potassium bifluoride (2.36 g, 30.2 mmol, 1.00 equiv) was added to chlorodimethyl(phenyl)-silane (**7ba**, 5.00 mL, 5.16 g, 30.2 mmol, 1.00 equiv), and the resulting reaction mixture was vigorously stirred for 30 min at 100 °C. Fluorodimethyl(phenyl)silane (**7aa**, 2.04 g, 13.2 mmol, 44%) was obtained by distillation under atmospheric pressure (b.p. 158 °C) as a colorless oil, which was stored in a glovebox after degassing by three freeze-pump-thaw cycles.

^{1}H NMR (500 MHz, C_6D_6, 298 K): δ/ppm = 7.45 (m_c, 2H, *o*-C*H*-Ph), 7.18–7.11 (m, 3H, *m*-C*H*-Ph, *p*-C*H*-Ph), 0.23 (d, $^{3}J_{H,F}$ = 7.5 Hz, 6H, SiC*H*$_3$).

^{13}C{^{1}H} NMR (126 MHz, C_6D_6, 298 K): δ/ppm = 136.5 (d, $^{2}J_{C,F}$ = 15.2 Hz, *i*-C-Ph), 133.4 (d, $^{3}J_{C,F}$ = 2.3 Hz, *o*-*C*H-Ph), 130.5 (*p*-*C*H-Ph), 128.3 (*m*-*C*H-Ph), –1.2 (d, $^{2}J_{C,F}$ = 16.3 Hz, Si*C*H$_3$).

^{19}F NMR (471 MHz, C_6D_6, 298 K): δ/ppm = –161.5 (sept, $^{3}J_{F,H}$ = 7.2 Hz, $^{1}J_{F,Si}$ = 278.2 Hz).

^{29}Si{^{1}H} DEPT NMR (99 MHz, C_6D_6, 298 K, optimized for $J_{H,Si}$ = 7 Hz, 24.1°): δ/ppm = 20.0 (d, $^{1}J_{Si,F}$ = 278.5 Hz).

IR (ATR): $\tilde{\nu}$/cm^{-1} = 3072 (w), 2963 (w), 1592 (w), 1428 (m), 1256 (s), 1122 (s), 868 (s), 831 (vs), 791 (vs), 766 (m), 727 (s), 696 (vs).

GLC-MS (EI): *m*/*z* = 154.0 (M^+), 139.0 (100%, $[M–Me]^+$), 91.0, 86.9, 77.0, 64.9, 62.9, 50.9.

HRMS (LIFDI): *m*/*z* calculated for $C_8H_{11}FSi^+$ $[M]^+$: 154.0614; found: 154.0609.

3.2 Bromodimethyl(phenyl)silane (**7ca**)

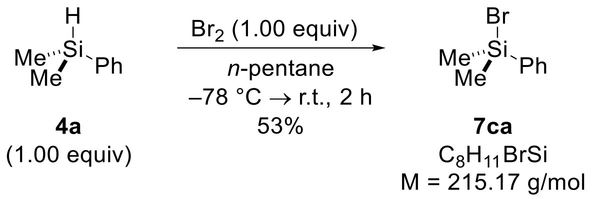

To a solution of dimethyl(phenyl)silane (**4a**, 6.00 mL, 5.33 g, 39.1 mmol, 1.00 equiv) in *n*-pentane (50 mL), bromine (2.00 mL, 6.24 g, 39.1 mmol, 1.00 equiv) was added dropwise at –78 °C. The reaction mixture was allowed to gradually warm to room temperature and stirred for additional 2 h. The solvent was removed under reduced pressure, and the crude product was purified by vacuum distillation (b.p. 76.5 °C at 7 mbar). Bromodimethyl(phenyl)silane (**7ca**, 4.50 g, 20.9 mmol, 53%) was obtained as a colorless oil, which was stored in a glovebox after degassing by three freeze-pump-thaw cycles.

Caution: Slow addition of bromine at –78 °C is advisable to avoid abrupt HBr evolution. The product was found to decompose rapidly and therby act corrosive to metals when handled outside of the glovebox due to release of HBr by hydrolysis.

^{1}H NMR (500 MHz, C_6D_6, 298 K): δ/ppm = 7.52–7.46 (m, 2H, *o*-C*H*-Ph), 7.15–7.11 (m, 3H, *m*-C*H*-Ph, *p*-C*H*-Ph), 0.55 (s, 6H, $^{1}J_{H,C}$ = 119.2 Hz, SiCH_3).

^{13}C{^{1}H} NMR (126 MHz, C_6D_6, 298 K): δ/ppm = 136.1 (*i*-C-Ph), 133.6 (*o*-*C*H-Ph), 130.6 (*p*-*C*H-Ph), 128.3 (*m*-*C*H-Ph), 2.8 ($^{1}J_{C,Si}$ = 58.4 Hz, Si*C*H_3).

^{29}Si{^{1}H} DEPT NMR (99 MHz, C_6D_6, 298 K, optimized for $J_{H,Si}$ = 7 Hz, 20.7°): δ/ppm = 16.4 ($^{1}J_{Si,C}$ = 58.2 Hz).

HRMS (LIFDI): *m*/*z* calculated for $C_8H_{11}Si^+$ [M–Br]$^+$: 135.0625; found: 135.0623.

3.3 Iododimethyl(phenyl)silane (**7da**)

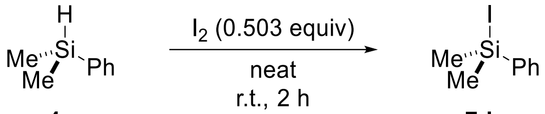

4a
(1.00 equiv)

7da
$C_8H_{11}ISi$
M = 262.17 g/mol

Iodine (5.00 g, 19.7 mmol, 0.503 equiv) was added portionwise to dimethyl(phenyl)silane (**4a**, 6.00 mL, 5.33 g, 39.1 mmol, 1.00 equiv) at 0 °C, and the reaction mixture was vigorously sitirred for 2 h at room temperature. Residual iodine was removed under reduced pressure (50–55 °C at 7 mbar). Iododimethyl(phenyl)silane (**7da**, 1.42 g, 5.42 mmol, 14%) was obtained by vacuum distillation (b.p. 76 °C at 7 mbar) as a colorless oil, which was stored in a glovebox after degassing by three freeze-pump-thaw cycles.

Caution: Slow addition of iodine at 0 °C is advisable to avoid abrupt HI evolution. The product was found to decompose rapidly and therby act corrosive to metals when handled outside of the glovebox due to release of HI by hydrolysis.

^{1}H NMR (500 MHz, C_6D_6, 298 K): δ/ppm = 7.49–7.43 (m, 2H, *o*-C*H*-Ph), 7.11 (m_c, 3H, *m*-C*H*-Ph, *p*-C*H*-Ph), 0.74 (s, 6H, $^1J_{H,C}$ = 120.8 Hz, SiCH_3).

^{13}C{^{1}H} NMR (126 MHz, C_6D_6, 298 K): δ/ppm = 135.9 (*i*-C-Ph), 133.7 (*o*-*C*H-Ph), 130.6 (*p*-*C*H-Ph), 128.3 (*m*-*C*H-Ph), 4.2 ($^1J_{C,Si}$ = 56.1 Hz, Si*C*H_3).

^{29}Si{^{1}H} DEPT NMR (99 MHz, C_6D_6, 298 K, optimized for $J_{H,Si}$ = 7 Hz, 20.7°): δ/ppm = –0.3.

HRMS (APCI): *m*/*z* calculated for $C_8H_{11}Si^+$ $[M–I]^+$: 135.0625; found: 135.0624.

3.4 Diethylfluoro(phenyl)silane (**7ab**) and ethyldifluoro(phenyl)silane (**S3**)

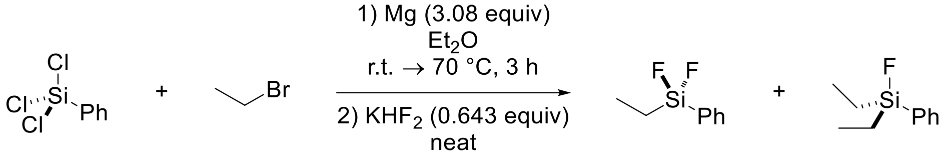

S1 (1.00 equiv) **S2** (2.15 equiv) **S3**: 8% $C_8H_{10}F_2Si$ M = 172.25 g/mol **7ab**: 2% $C_{10}H_{15}FSi$ M = 182.31 g/mol

Magnesium turnings (7.00 g, 288 mmol, 3.08 equiv) were heated with vigorous stirring under vacuum (2 times) and suspended in diethyl ether (250 mL). 1,2-Bromoethane (**S2**, 15.0 mL, 21.9 g, 201 mmol, 2.15 equiv) was added dropwise over 30 min to maintain reflux. The reaction mixture was stirred for 1 h at 70 °C and allowed to warm to room temperature. The reaction mixture was then added dropwise to a solution of trichloro(phenyl)silane (**S1**, 15.0 mL, 19.8 g, 93.7 mmol, 1.00 equiv) in diethyl ether (250 mL), and the reaction mixture was stirred for additional 2 h at 70 °C. The mixture was filtered, the residue was washed with *n*-pentane (3 × 100 mL), and the solvent was removed under reduced pressure. Vacuum distillation afforded a mixture of $Et_2PhSiCl$ (δ(^{29}Si) = 24.0 ppm) and $EtPhSiCl_2$ (δ(^{29}Si) = 20.5 ppm) as a colorless oil (b.p. 98 °C at 10 mbar, 11.6 g), which was used in the next step without further purification. Potassium bifluoride (4.70 g, 60.2 mmol, 0.643 equiv) was added, and the reaction mixture was vigorously stirred for 30 min at 100 °C. Fractional vacuum distillation afforded ethyldifluoro(phenyl)silane (**S3**, 1.30 g, 7.55 mmol, 8%, b.p. 63 °C at 36 mbar) and diethylfluoro(phenyl)silane (**7ab**, 363 mg, 1.99 mmol, 2%, b.p. 83 °C at 30 mbar) as colorless oils, which were stored in a glovebox after degassing by three freeze-pump-thaw cycles.

Spectroscopic data for ethyldifluoro(phenyl)silane (**S3**):

^{1}H NMR (500 MHz, C_6D_6, 298 K): δ/ppm = 7.49 (dd, $^3J_{H,H}$ = 8.0 Hz, $^4J_{H,H}$ = 1.3 Hz, 2H, *o*-C*H*-Ph), 7.15 (tt, $^3J_{H,H}$ = 7.4 Hz, $^4J_{H,H}$ = 1.4 Hz, 1H, *p*-C*H*-Ph), 7.07 (t, $^3J_{H,H}$ = 7.5 Hz, 2H, *m*-C*H*-Ph), 0.86 (t, $^3J_{H,H}$ = 7.9 Hz, 3H, $SiCH_2CH_3$), 0.72–0.64 (m, 2H, $SiCH_2CH_3$).

^{13}C{^{1}H} NMR (126 MHz, C_6D_6, 298 K): δ/ppm = 133.9 (*o*-*C*H-Ph), 131.9 (*p*-*C*H-Ph), 129.4 (t, $^2J_{C,F}$ = 18.7 Hz, *i*-C-Ph), 128.5 (*m*-*C*H-Ph), 5.2 ($SiCH_2CH_3$), 4.1 (t, $^2J_{C,F}$ = 16.3 Hz, $SiCH_2CH_3$).

^{19}F NMR (471 MHz, C_6D_6, 298 K): δ/ppm = –142.6 (t, $^3J_{F,H}$ = 5.0 Hz, $^1J_{F,Si}$ = 299.0 Hz).

^{29}Si{^{1}H} DEPT NMR (99 MHz, C_6D_6, 298 K, optimized for $J_{H,Si}$ = 7 Hz, 22.2°): δ/ppm = –14.1 (t, $^1J_{Si,F}$ = 299.1 Hz).

IR (ATR): $\tilde{\nu}$/cm^{-1} = 3076 (w), 2968 (w), 2885 (w), 1593 (w), 1461 (w), 1430 (m), 1250 (w), 1128 (s), 1010 (m), 964 (m), 891 (s), 851 (vs), 736 (vs), 697 (vs), 659 (s).

GLC-MS (EI): *m*/*z* = 172.0 (M^+), 152.9 ($[M–F]^+$), 143.0 (100%, $[M–Et]^+$), 91.0, 78.0, 77.0, 51.0.

HRMS (LIFDI): *m*/*z* calculated for $C_{10}H_{15}F_2Si^+$ $[M]^+$: 172.0514; found: 172.0514.

Spectroscopic data for diethylfluoro(phenyl)silane (**7ab**):

^{1}H NMR (500 MHz, C_6D_6, 298 K): δ/ppm = 7.55–7.50 (m, 2H, *o*-C*H*-Ph), 7.21–7.16 (m, 3H, *m*-C*H*-Ph, *p*-C*H*-Ph), 0.94 (t, $^3J_{H,H}$ = 7.9 Hz, 6H, $SiCH_2CH_3$), 0.80–0.70 (m, 4H, $SiCH_2CH_3$).

^{13}C{^{1}H} NMR (126 MHz, C_6D_6, 298 K): δ/ppm = 134.8 (d, $^2J_{C,F}$ = 15.9 Hz, *i*-C-Ph), 133.8 (d, $^3J_{C,F}$ = 2.8 Hz, *o*-CH-Ph), 130.4 (*p*-*C*H-Ph), 128.3 (*m*-*C*H-Ph), 6.3 ($SiCH_2CH_3$), 6.0 (d, $^2J_{C,F}$ = 14.0 Hz, $SiCH_2CH_3$).

^{19}F NMR (471 MHz, C_6D_6, 298 K): δ/ppm = –175.8 (quint, $^3J_{F,H}$ = 6.4 Hz, $^1J_{F,Si}$ = 287.5 Hz).

^{29}Si{^{1}H} DEPT NMR (99 MHz, C_6D_6, 298 K, optimized for $J_{H,Si}$ = 7 Hz, 16.8°): δ/ppm = 19.6 (d, $^1J_{Si,F}$ = 287.0 Hz).

IR (ATR): $\tilde{\nu}$/cm^{-1} = 3071 (w), 2960 (m), 2879 (m), 1591 (w), 1460 (m), 1427 (m), 1240 (m), 1117 (s), 1008 (m), 965 (m), 831 (vs), 717 (vs), 695 (vs).

GLC-MS (EI): *m*/*z* = 182.0 (M^+), 153.0 ($[M–Et]^+$), 125.0 (100%), 98.9, 91.0, 77.0, 65.0, 50.9.

HRMS (LIFDI): *m*/*z* calculated for $C_{10}H_{15}Si^+$ $[M–F]^+$: 163.0938; found: 163.0934.

3.5 Chlorodiethylsilane (**6bb**)

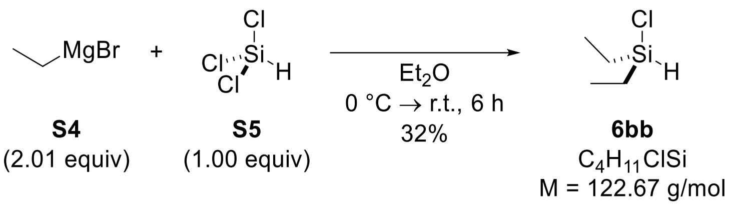

Ethylmagnesium bromide (**S4**, 3.00 M in Et_2O, 43.5 mL, 131 mmol, 2.01 equiv) was added dropwise to a solution of trichlorosilane (**S5**, 6.60 mL, 8.84 g, 65.3 mmol, 1.00 equiv) in Et_2O (150 mL) at 0 °C, and the reaction mixture was stirred for 6 h at room temperature. The mixture was filtered, and the residue was washed with *n*-pentane (3 × 50 mL). The solvent was removed under reduced pressure, and the crude product was purified by distillation at atmospheric pressure (b.p. 100 °C). Chlorodiethylsilane (**6bb**, 2.54 g, 20.7 mmol, 32%) was obtained as a colorless oil.

^{1}H NMR (500 MHz, C_6D_6, 298 K): δ/ppm = 4.68 (s, $^{1}J_{H,Si}$ = 217.1 Hz, 1H, Si*H*), 0.87 (t, $^{3}J_{H,H}$ = 7.9 Hz, 6H, $SiCH_2CH_3$), 0.57 (q, $^{3}J_{H,H}$ = 7.8 Hz, 4H, $SiCH_2CH_3$).

^{13}C{^{1}H} NMR (126 MHz, C_6D_6, 298 K): δ/ppm = 7.6 ($SiCH_2CH_3$), 6.7 $(SiCH_2CH_3)_2$.

^{29}Si{^{1}H} DEPT NMR (99 MHz, C_6D_6, 298 K, optimized for $J_{H,Si}$ = 7 Hz, 18.4°): δ/ppm = 18.7.

HRMS (APCI): *m*/*z* calculated for $C_4H_{10}ClSi^+$ $[M–H]^+$: 121.0235; found: 121.0234.

3.6 Bromodiethylsilane (**6cb**)

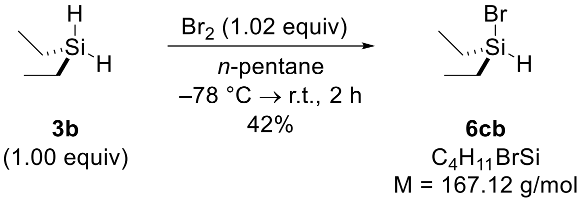

To a solution of diethylsilane (**3b**, 8.68 mL, 5.92 g, 67.1 mmol, 1.00 equiv) in *n*-pentane (45 mL), bromine (3.50 mL, 10.9 g, 68.3 mmol, 1.02 equiv) was added dropwise at –78 °C. The reaction mixture was allowed to warm to room temperature and stirred for additional 1 h. The solvent was removed under reduced pressure, and the crude product was purified by distillation at atmospheric pressure (b.p. 121 °C). Bromodiethylsilane (**6cb**, 4.76 g, 28.5 mmol, 42%) was obtained as a colorless oil, which was stored in a glovebox after degassing by three freeze-pump-thaw cycles.

Caution: Slow addition of bromine at –78 °C is advisable to avoid abrupt HBr evolution. The product was found to decompose rapidly and therby act corrosive to metals when handled outside of the glovebox due to release of HBr by hydrolysis.

^{1}H NMR (500 MHz, C_6D_6, 298 K): δ/ppm = 4.60 (quint, $^{3}J_{H,H}$ = 2.6 Hz, $^{1}J_{H,Si}$ = 218.4 Hz, 1H, Si*H*), 0.86 (t, $^{3}J_{H,H}$ = 7.8 Hz, 6H, SiCH$_2$C*H*$_3$), 0.65 (m$_c$, 4H, SiC*H*$_2$CH$_3$).

^{13}C{^{1}H} NMR (126 MHz, C_6D_6, 298 K): δ/ppm = 7.6 (Si*C*H$_2$CH$_3$), 7.3 (SiCH$_2$*C*H$_3$).

^{29}Si{^{1}H} DEPT NMR (99 MHz, C_6D_6, 298 K, optimized for $J_{H,Si}$ = 200 Hz, 90.0°): δ/ppm = 15.1.

IR (ATR): $\tilde{\nu}$/cm^{-1} = 2960 (m), 2878 (m), 2149 (m, Si–H), 1458 (m), 1408 (w), 1379 (w), 1232 (w), 1006 (m), 973 (m), 810 (s), 776 (vs), 716 (s), 690 (s).

HRMS (APCI): *m*/*z* calculated for $C_4H_{11}Si^+$ [M–Br]$^+$: 87.0625; found: 87.0622.

3.7 Diethyliodosilane (**6db**)

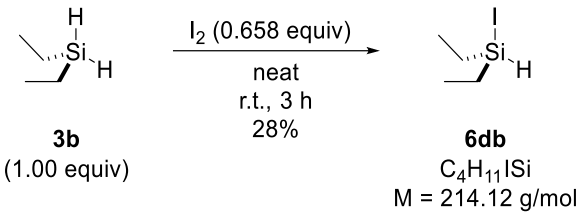

Iodine (4.21 g, 16.6 mmol, 0.658 equiv) was added to diethylsilane (**3b**, 3.26 mL, 2.22 g, 25.2 mmol, 1.00 equiv), and the reaction mixture was vigorously sitirred for 3 h at room temperature. Diethyliodosilane (**6db**, 1.51 g, 7.05 mmol, 28%) was obtained by vacuum distillation (b.p. 65 °C at 60 mbar) as a colorless oil, which was stored in a glovebox after degassing by three freeze-pump-thaw cycles.

Caution: An ice bath sould be kept at hand during this reaction to counteract abrupt HI evolution. The product was found to decompose rapidly and therby act corrosive to metals when handled outside of the glovebox due to release of HI by hydrolysis.

^{1}H NMR (500 MHz, C_6D_6, 298 K): δ/ppm = 4.50 (s, $^{1}J_{H,Si}$ = 217.0 Hz, 1H, Si*H*), 0.83 (t, $^{3}J_{H,H}$ = 7.5 Hz, 6H, $SiCH_2CH_3$), 0.76 (m_c, 4H, $SiCH_2CH_3$).

^{13}C{^{1}H} NMR (126 MHz, C_6D_6, 298 K): δ/ppm = 8.4 ($SiCH_2CH_3$), 7.3 ($SiCH_2CH_3$).

^{29}Si{^{1}H} DEPT NMR (99 MHz, C_6D_6, 298 K, optimized for $J_{H,Si}$ = 7 Hz, 18.4°): δ/ppm = –1.5.

HRMS (APCI): *m*/*z* calculated for $C_4H_{10}ISi^+$ [M–H]$^+$: 212.9591; found: 212.9593.

3.8 Chlorodiisopropyl(phenyl)silane (**7bc**)

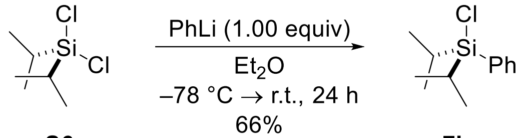

S6
(1.01 equiv)

7bc
$C_{12}H_{19}ClSi$
M = 226.82 g/mol

A solution of dichlorodiisopropylsilane (**S6**, 10.0 mL, 10.3 g, 55.4 mmol, 1.01 equiv) in Et_2O (120 mL) was cooled to –78 °C, and phenyllithium (1.90 M in Bu_2O, 29.0 mL, 55.1 mmol, 1.00 equiv) was added dropwise. The reaction mixture was stirred for 10 min at –78 °C, gradually warmed to room temperature and stirred for additional 24 h. The reaction mixture was filtered and concentrated under reduced pressure. Residual Bu_2O was removed by distillation at atmospheric pressure (b.p. 142 °C), and the crude product was purified by vacuum distillation (b.p. 124 °C at 10 mbar). Chlorodiisopropyl(phenyl)silane (**7bc**, 8.25 g, 36.4 mmol, 66%) was obtained as a colorless oil, which was stored in a glovebox after degassing by three freeze-pump-thaw cycles.

^{1}H NMR (500 MHz, CD_2Cl_2, 298 K): δ/ppm = 7.64–7.59 (m, 2H, *o*-C*H*-Ph), 7.47–7.38 (m, 3H, *m*-C*H*-Ph, *p*-C*H*-Ph), 1.43 (sept, $^3J_{H,H}$ = 7.4 Hz, 2H, SiC*H*(CH_3)$_2$), 1.09 (d, $^3J_{H,H}$ = 7.3 Hz, 6H, SiCH(CH_3)$_2$), 1.01 (d, $^3J_{H,H}$ = 7.5 Hz, 6H, SiCH(CH_3)$_2$).

^{13}C{^{1}H} NMR (126 MHz, CD_2Cl_2, 298 K): δ/ppm = 134.8 (*o*-*C*H-Ph), 132.6 (*i*-C-Ph), 130.4 (*p*-*C*H-Ph), 128.2 (*m*-*C*H-Ph), 17.2 (SiCH(*C*H_3)$_2$), 16.9 (SiCH(*C*H_3)$_2$), 14.2 (Si*C*H(CH_3)$_2$).

^{29}Si{^{1}H} DEPT NMR (99 MHz, CD_2Cl_2, 298 K, optimized for $J_{H,Si}$ = 7 Hz, 14.5°): δ/ppm = 27.1.

IR (ATR): $\tilde{\nu}$/cm^{-1} = 2947 (m), 2866 (m), 1462 (m), 1428 (m), 1384 (w), 1111 (s), 993 (m), 921 (w), 881 (s), 738 (s), 697 (vs), 668 (vs).

HRMS (APCI): *m*/*z* calculated for $C_{12}H_{19}Si^+$ $[M–Cl]^+$: 191.1251; found: 191.1250.

3.9 Fluorodiisopropyl(phenyl)silane (**7ac**)

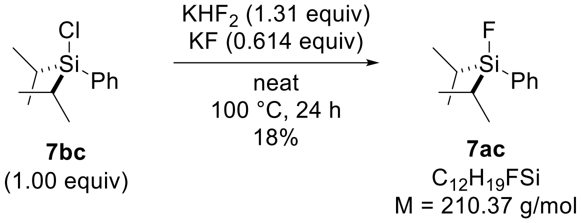

Potassium bifluoride (3.27 g, 41.9 mmol, 1.31 equiv) and potassium fluoride (1.14 g, 19.7 mmol, 0.614 equiv) were added to chlorodiisopropyl(phenyl)silane (**7bc**, 7.66 mL, 7.28 g, 32.1 mmol, 1.00 equiv), and the reaction mixture was vigorously stirred for 24 h at 100 °C. Fluorodiisopropyl(phenyl)silane (**7ac**, 1.22 g, 5.80 mmol, 18%) was obtained by vacucum distillation (b.p. 94 °C at 10 mbar) as a colorless oil, which was stored in a glovebox after degassing by three freeze-pump-thaw cycles.

^{1}H NMR (500 MHz, CD_2Cl_2, 298 K): δ/ppm = 7.57–7.53 (m, 2H, *o*-C*H*-Ph), 7.46–7.37 (m, 3H, *m*-C*H*-Ph, *p*-C*H*-Ph), 1.29 (oct, $^{3}J_{H,H}$ = $^{3}J_{H,F}$ = 7.3 Hz, 2H, SiC*H*(CH_3)$_2$), 1.08 (d, $^{3}J_{H,H}$ = 7.3 Hz, 6H, SiCH(CH_3)$_2$), 1.01 (d, $^{3}J_{H,H}$ = 7.5 Hz, 6H, SiCH(CH_3)$_2$).

^{13}C{^{1}H} NMR (126 MHz, CD_2Cl_2, 298 K): δ/ppm = 134.3 (d, $^{3}J_{C,F}$ = 3.0 Hz, *o*-*C*H-Ph), 133.3 (d, $^{2}J_{C,F}$ = 14.9 Hz, *i*-C-Ph), 130.3 (*p*-*C*H-Ph), 128.2 (*m*-*C*H-Ph), 16.9 (SiCH(*C*H_3)$_2$), 16.7 (SiCH(*C*H_3)$_2$), 12.6 (d, $^{2}J_{C,F}$ = 13.1 Hz, Si*C*H(CH_3)$_2$).

^{19}F NMR (471 MHz, CD_2Cl_2, 298 K): δ/ppm = –187.4 (t, $^{3}J_{F,H}$ = 6.0 Hz, $^{1}J_{F,Si}$ = 293.3 Hz).

^{29}Si{^{1}H} DEPT NMR (99 MHz, CD_2Cl_2, 298 K, optimized for $J_{H,Si}$ = 7 Hz, 14.5°): δ/ppm = 17.7 (d, $^{1}J_{Si,F}$ = 292.9 Hz).

IR (ATR): $\tilde{\nu}$/cm^{-1} = 3071 (w), 2945 (m), 2867 (m), 1462 (m), 1428 (m), 1385 (w), 1245 (w), 1117 (s), 1064 (w), 994 (m), 920 (w), 880 (s), 827 (vs), 738 (m), 712 (s), 697 (vs), 674 (vs).

GLC-MS (EI): *m*/*z* = 210.1 (M^+), 167.0 ([M–*i*Pr]$^+$), 139.0 (100%), 125.0, 91.0, 77.0, 63.0.

HRMS (APCI): *m*/*z* calculated for $C_{12}H_{19}Si^+$ [M–F]$^+$: 191.1251; found: 191.1251.

3.10 Chlorodiisopropylsilane (**6bc**)

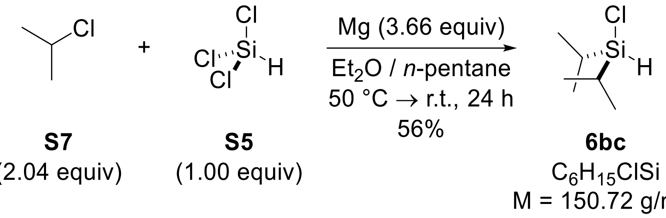

S7 (2.04 equiv) **S5** (1.00 equiv) **6bc** $C_6H_{15}ClSi$ M = 150.72 g/mol

Magnesium turnings (11.0 g, 452 mmol, 3.66 equiv) were heated with vigorous stirring under vacuum (2 times) and suspended in diethyl ether (150 mL). 2-Chloropropane (**S7**, 23.0 mL, 19.8 g, 252 mmol, 2.04 equiv) was added dropwise over 30 min to maintain reflux. The reaction mixture was stirred for 30 min at 50 °C and allowed to warm to room temperature. The reaction mixture was then added dropwise to a solution of trichlorosilane (**S5**, 12.5 mL, 16.8 g, 124 mmol, 1.00 equiv) in *n*-pentane (300 mL), and the mixture was stirred for additional 18 h at room temperature. The reaction mixture was filtered, and the residue was washed with *n*-pentane (3 × 100 mL). The solvent was removed under reduced pressure, and the crude product was purified by distillation at atmospheric pressure (b.p. 137 °C). Chlorodiisopropylsilane (**6bc**, 10.4 g, 69.0 mmol, 56%) was obtained as a colorless oil.

^{1}H NMR (500 MHz, C_6D_6, 298 K): δ/ppm = 4.49 (t, $^3J_{H,H}$ = 1.7 Hz, $^1J_{H,Si}$ = 213.6 Hz, 1H, Si*H*), 1.01–0.85 (m, 14H, SiC*H*(C*H*$_3$)$_2$).

^{13}C{^{1}H} NMR (126 MHz, C_6D_6, 298 K): δ/ppm = 17.3 (SiCH(*C*H$_3$)$_2$), 16.9 (SiCH(*C*H$_3$)$_2$), 13.7 (Si*C*H(CH$_3$)$_2$).

^{29}Si{^{1}H} DEPT NMR (99 MHz, C_6D_6, 298 K, optimized for $J_{H,Si}$ = 200 Hz, 90.0°): δ/ppm = 24.3.

IR (ATR): $\tilde{\nu}$/cm^{-1} = 2948 (m), 2867 (m), 2140 (m, Si–H), 1462 (m), 1386 (w), 1241 (w), 1067 (w), 1006 (m), 921 (w), 880 (m), 781 (s), 667 (m).

HRMS (APCI): *m*/*z* calculated for $C_6H_{14}ClSi^+$ [M–H]$^+$: 149.0548; found: 149.0546.

3.11 Fluorodiisopropylsilane (**6ac**)

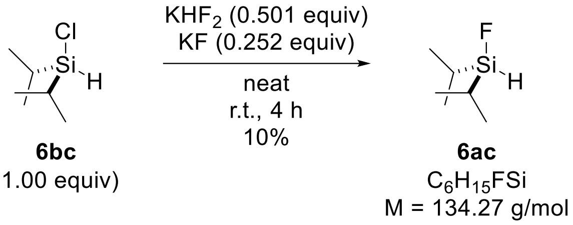

Potassium bifluoride (1.32 g, 16.9 mmol, 0.501 equiv) and potassium fluoride (493 mg, 8.49 mmol, 0.252 equiv) were added to chlorodiisopropylsilane (**6bc**, 5.83 mL, 5.08 g, 33.7 mmol, 1.00 equiv), and the reaction mixture was vigorously stirred for 4 h at room temperature. Fluorodiisopropylsilane (**6ac**, 470 mg, 3.50 mmol, 10%) was obtained by distillation at atmospheric pressure (b.p. 96 °C) as a colorless oil, which was stored in a glovebox after degassing by three freeze-pump-thaw cycles.

<u>*Note*</u>: Significant formation of *i*Pr_2SiF_2 (δ(^{29}Si) = –3.3 ppm) was observed when using 1.00 equiv KHF_2, which could not be separated by distillation.

^{1}H NMR (700 MHz, C_6D_6, 298 K): δ/ppm = 4.51 (dt, $^2J_{H,F}$ = 51.3 Hz, $^3J_{H,H}$ = 2.0 Hz, $^1J_{H,Si}$ = 208.2 Hz, 1H, Si*H*), 0.99 (d, $^3J_{H,H}$ = 7.4 Hz, 6H, SiCH(C*H*$_3$)$_2$), 0.93 (d, $^3J_{H,H}$ = 7.4 Hz, 6H, SiCH(C*H*$_3$)$_2$), 0.88–0.80 (m, 2H, SiC*H*(CH$_3$)$_2$).

^{13}C{^{1}H} NMR (126 MHz, C_6D_6, 298 K): δ/ppm = 16.5 (SiCH(*C*H$_3$)$_2$), 12.4 (d, $^2J_{C,F}$ = 12.2 Hz, Si*C*H(CH$_3$)$_2$).

^{19}F NMR (659 MHz, C_6D_6, 298 K): δ/ppm = –187.6 (dt, $^2J_{F,H}$ = 51.4 Hz, $^3J_{F,H}$ = 4.8 Hz, $^1J_{F,Si}$ = 299.0 Hz).

^{29}Si{^{1}H} DEPT NMR (99 MHz, C_6D_6, 298 K, optimized for $J_{H,Si}$ = 7 Hz, 15.5°): δ/ppm = 24.1 (d, $^1J_{Si,F}$ = 299.2 Hz).

IR (ATR): $\tilde{\nu}$/cm^{-1} = 2947 (m), 2868 (m), 2134 (m, Si–H), 1462 (m), 1387 (w), 1367 (w), 1247 (w), 1068 (w), 1003 (m), 921 (w), 880 (s), 846 (vs), 805 (vs), 674 (m).

HRMS (APCI): *m*/*z* calculated for $C_6H_{14}FSi^+$ [M–H]$^+$: 133.0843; found: 133.0842.

3.12 Diisopropylsilane (**3c**)

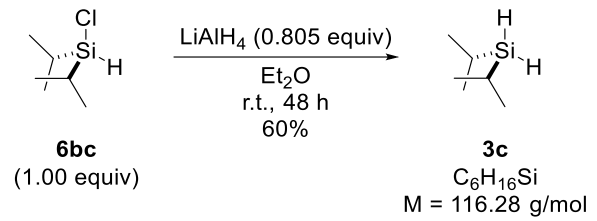

Lithium aluminium hydride (1.75 g, 46.1 mmol, 0.805 equiv) was suspended in diethyl ether (100 mL), and chlorodiisopropylsilane (**6bc**, 12.0 mL, 8.64 g, 57.3 mmol, 1.00 equiv) was added. The reaction mixture was stirred for 48 h at room temperature. The mixture was filtered, the residue was washed with *n*-pentane (2 × 50 mL), and the solvent was removed under reduced pressure. The crude product was purified by distillation at atmospheric pressure (b.p. 98.5 °C), affording diisopropylsilane (**3c**, 5.72 g, 34.4 mmol, 60%) as a colorless oil.

^{1}H NMR (500 MHz, C_6D_6, 298 K): δ/ppm = 3.71 (t, $^3J_{H,H}$ = 2.9 Hz, $^1J_{H,Si}$ = 181.3 Hz, 2H, Si*H*), 1.02 (d, $^3J_{H,H}$ = 6.9 Hz, 12H, SiCH(C*H*$_3$)$_2$), 0.98–0.87 (m, 2H, SiC*H*(CH$_3$)$_2$).

^{13}C{^{1}H} NMR (126 MHz, C_6D_6, 298 K): δ/ppm = 19.6 (SiCH(*C*H$_3$)$_2$), 9.6 (Si*C*H(CH$_3$)$_2$).

^{29}Si{^{1}H} DEPT NMR (99 MHz, C_6D_6, 298 K, optimized for $J_{H,Si}$ = 200 Hz, 45.0°): δ/ppm = –8.3.

IR (ATR): $\tilde{\nu}$/cm^{-1} = 2940 (m), 2863 (m), 2113 (s, Si–H), 1461 (m), 1003 (m), 934 (s), 880 (m), 827 (s).

HRMS (APCI): *m*/*z* calculated for $C_6H_{15}Si^+$ [M–H]$^+$: 115.0938; found: 115.0937.

3.13 Bromodiisopropylsilane (**6cc**)

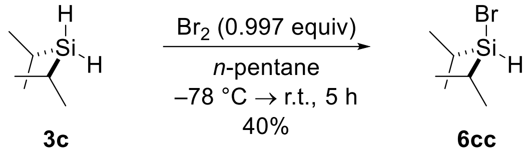

3c
(1.00 equiv)

6cc
$C_6H_{15}BrSi$
M = 195.18 g/mol

To a solution of diisopropylsilane (**3c**, 5.96 mL, 4.10 g, 35.2 mmol, 1.00 equiv) in *n*-pentane (50 mL), bromine (1.80 mL, 5.62 g, 35.1 mmol, 0.997 equiv) was added dropwise at –78 °C. The reaction mixture was stirred for 4 h at –78 °C, gradually warmed to room temperature and stirred for additional 1 h. The solvent was removed under reduced pressure, and the crude product was purified by vacuum distillation (b.p. 69 °C at 43 mbar). Bromodiisopropylsilane (**6cc**, 2.75 g, 14.1 mmol, 40%) was obtained as a colorless oil, which was stored in a glovebox after degassing by three freeze-pump-thaw cycles.

Caution: Slow addition of bromine at –78 °C is advisable to avoid abrupt HBr evolution. The product was found to decompose rapidly and therby act corrosive to metals when handled outside of the glovebox due to release of HBr by hydrolysis.

^{1}H NMR (500 MHz, C_6D_6, 298 K): δ/ppm = 4.44 (s, $^1J_{H,Si}$ = 214.1 Hz, 1H, Si*H*), 1.12–0.79 (m, 14H, SiC*H*(C*H*$_3$)$_2$).

^{13}C{^{1}H} NMR (126 MHz, C_6D_6, 298 K): δ/ppm = 17.7 (SiCH(*C*H$_3$)$_2$), 17.4 (SiCH(*C*H$_3$)$_2$), 13.7 (Si*C*H(CH$_3$)$_2$).

^{29}Si{^{1}H} DEPT NMR (99 MHz, C_6D_6, 298 K, optimized for $J_{H,Si}$ = 7 Hz, 15.5°): δ/ppm = 23.4.

IR (ATR): $\tilde{\nu}$/cm^{-1} = 2947 (m), 2866 (m), 2139 (m, Si–H), 1461 (m), 1385 (w), 1368 (w), 1241 (w), 1066 (w), 1007 (m), 921 (w), 879 (m), 808 (m), 789 (s), 761 (vs), 663 (m).

HRMS (APCI): *m*/*z* calculated for $C_6H_{15}Si^+$ [M–Br]$^+$: 115.0938; found: 115.0935.

3.14 Diisopropyliodosilane (**6dc**)

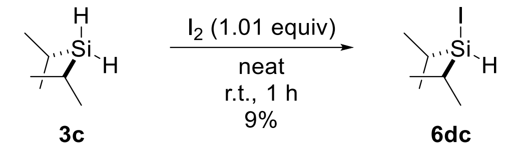

3c (1.00 equiv) → **6dc** $C_6H_{15}ISi$, M = 242.18 g/mol

Iodine (6.18 g, 24.4 mmol, 1.01 equiv) was added to diisopropylsilane (**3c**, 3.90 mL, 2.80 g, 24.1 mmol, 1.00 equiv), and the reaction mixture was vigorously sitirred for 1 h at room temperature. Diisopropyliodosilane (**6dc**, 528 mg, 2.18 mmol, 9%) was obtained by vacuum distillation (b.p. 62 °C at 15 mbar) as a colorless oil, which was stored in a glovebox after degassing by three freeze-pump-thaw cycles.

<u>Caution</u>: *An ice bath sould be kept at hand during this reaction to counteract abrupt HI evolution. The product was found to decompose rapidly and therby act corrosive to metals when handled outside of the glovebox due to release of HI by hydrolysis.*

^{1}H NMR (500 MHz, C_6D_6, 298 K): δ/ppm = 4.39 (s, $^{1}J_{H,Si}$ = 212.1 Hz, 1H, Si*H*), 1.13–0.76 (m, 14H, SiC*H*(C*H*$_3$)$_2$).

^{13}C{^{1}H} NMR (126 MHz, C_6D_6, 298 K): δ/ppm = 18.42 (SiCH(*C*H$_3$)$_2$), 18.40 (SiCH(*C*H$_3$)$_2$), 13.1 (Si*C*H(CH$_3$)$_2$).

^{29}Si{^{1}H} DEPT NMR (99 MHz, C_6D_6, 298 K, optimized for $J_{H,Si}$ = 7 Hz, 15.5°): δ/ppm = 13.1.

HRMS (APCI): *m*/*z* calculated for $C_6H_{14}ISi^+$ [M–H]$^+$: 240.9904; found: 240.9901.

3.15 Trifluoro(phenyl)silane (**S8**)

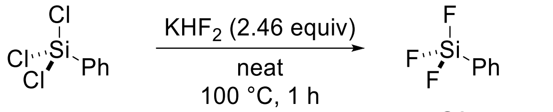

S1 (1.00 equiv) → **S8** $C_6H_5F_3Si$ M = 162.19 g/mol

Potassium bifluoride (6.00 g, 7.68 mmol, 2.46 equiv) was added to trichloro(phenyl)silane (**S1**, 5.00 mL, 6.61 g, 31.2 mmol, 1.00 equiv), and the reaction mixture was vigorously stirred for 1 h at 100 °C. Trifluoro(phenyl)silane (**S8**, 1.20 g, 7.40 mmol, 24%) was obtained by distillation at atmospheric pressure (b.p. 95 °C) as a colorless oil, which was stored in a glovebox after degassing by three freeze-pump-thaw cycles.

^{1}H NMR (500 MHz, C_6D_6, 298 K): δ/ppm = 7.29 (d, $^3J_{H,H}$ = 6.9 Hz, 2H, *o*-C*H*-Ph), 7.03 (t, $^3J_{H,H}$ = 7.6 Hz, 1H, *p*-C*H*-Ph), 6.88 (t, $^3J_{H,H}$ = 7.6 Hz, 2H, *m*-C*H*-Ph).

^{13}C{^{1}H} NMR (126 MHz, CD_2Cl_2, 298 K): δ/ppm = 135.2 (*o*-*C*H-Ph), 134.2 (*p*-*C*H-Ph), 129.4 (*m*-*C*H-Ph), 120.9 (q, $^2J_{C,F}$ = 25.7 Hz, *i*-C-Ph).

^{19}F NMR (471 MHz, C_6D_6, 298 K): δ/ppm = –140.8 (s, $^1J_{F,Si}$ = 268.9 Hz).

^{29}Si{^{1}H} IG NMR (99 MHz, CD_2Cl_2, 298 K): δ/ppm = –72.7 (q, $^1J_{Si,F}$ = 267.3 Hz).

IR (ATR): $\tilde{\nu}$/cm^{-1} = 3082 (w), 1596 (m), 1433 (m), 1140 (s), 943 (vs), 854 (vs), 740 (s), 695 (s).

HRMS (APCI): *m*/*z* calculated for $C_6H_5F_3Si^+$ $[M]^+$: 162.0107; found: 162.0108.

3.16 Di-*tert*-butylfluoro(phenyl)silane (**7ad**)

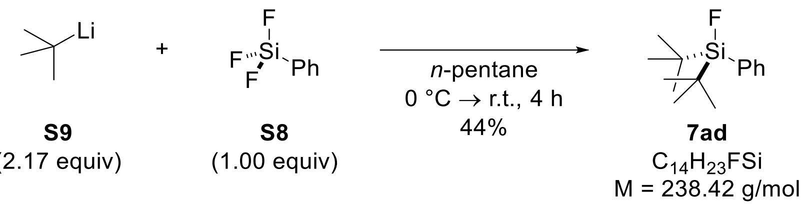

tert-Butyllithium (**S9**, 1.90 M in *n*-pentane, 37.0 mL, 70.3 mmol, 2.17 equiv) was added dropwise to a solution of trifluoro(phenyl)silane (**S8**, 5.25 g, 32.4 mmol, 1.00 equiv) in *n*-pentane (50 mL) at 0 °C, and the reaction mixture was stirred for 4 h at room temperature. The reaction mixture was filtered, and the residue was washed with *n*-pentane (3 × 50 mL). The solvent was removed under reduced pressure, and the crude product was purified by vacuum distillation (b.p. 102 °C at 7 mbar). Di-*tert*-butylfluoro(phenyl)silane (**7ad**, 3.44 g, 14.4 mmol, 44%) was obtained as a colorless oil, which was stored in a glovebox after degassing by three freeze-pump-thaw cycles.

^{1}H NMR (500 MHz, C_6D_6, 298 K): δ/ppm = 7.68–7.63 (m, 2H, *o*-C*H*-Ph), 7.20–7.17 (m, 3H, *m*-C*H*-Ph, *p*-C*H*-Ph), 1.06 (d, $^{4}J_{H,F}$ = 1.2 Hz, 18H, SiC(CH_3)$_3$).

^{13}C{^{1}H} NMR (126 MHz, C_6D_6, 298 K): δ/ppm = 134.3 (d, $^{3}J_{C,F}$ = 4.6 Hz, *o*-*C*H-Ph), 134.0 (d, $^{2}J_{C,F}$ = 14.0 Hz, *i*-C-Ph), 129.9 (*p*-*C*H-Ph), 128.1 (*m*-*C*H-Ph), 27.5 (SiC(*C*H_3)$_3$), 20.4 (d, $^{2}J_{C,F}$ = 12.8 Hz, Si*C*(CH_3)$_3$).

^{19}F NMR (471 MHz, C_6D_6, 298 K): δ/ppm = –188.3 (s, $^{1}J_{F,Si}$ = 299.0 Hz).

^{29}Si{^{1}H} DEPT NMR (99 MHz, C_6D_6, 298 K, optimized for $J_{H,Si}$ = 7 Hz, 12.9°): δ/ppm = 13.4 (d, $^{1}J_{Si,F}$ = 297.9 Hz).

IR (ATR): $\tilde{\nu}$/cm^{-1} = 3072 (w), 2960 (m), 2933 (m), 2890 (m), 2859 (m), 1469 (s), 1428 (m), 1390 (m), 1364 (m), 1112 (s), 1012 (w), 936 (w), 835 (s), 824 (vs), 813 (vs), 738 (m), 700 (vs).

GLC-MS (EI): *m*/*z* = 238.1 (M^+), 181.0 ([M–*t*Bu]$^+$), 139.0 (100%), 125.0, 91.0, 77.0, 56.0.

HRMS (LIFDI): *m*/*z* calculated for $C_{14}H_{23}FSi^+$ [M]$^+$: 238.1548; found: 238.1547.

3.17 Di-*tert*-butylchlorosilane (**6bd**)

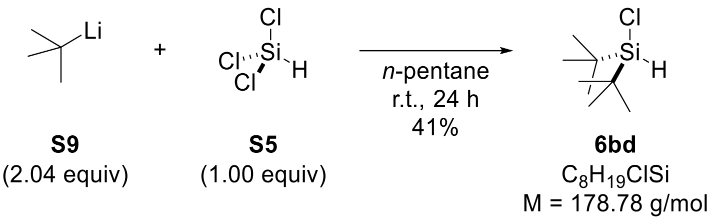

tert-Butyllithium (**S9**, 1.90 M in *n*-pentane, 27.5 mL, 52.3 mmol, 2.04 equiv) was added dropwise to a solution of trichlorosilane (**S5**, 2.60 mL, 3.48 g, 25.7 mmol, 1.00 equiv) in *n*-pentane (50 mL), and the reaction mixture was stirred for 18 h at room temperature. The reaction mixture was filtered, and the residue was washed with *n*-pentane (2 × 50 mL). The solvent was removed under reduced pressure, and the crude product was purified by distillation at atmospheric pressure (b.p. 167.5 °C). Di-*tert*-butylchlorosilane (**6bd**, 1.87 g, 10.5 mmol, 41%) was obtained as a colorless oil, which was stored in a glovebox after degassing by three freeze-pump-thaw cycles.

^{1}H NMR (500 MHz, C_6D_6, 298 K): δ/ppm = 4.35 (s, $^{1}J_{H,Si}$ = 211.8 Hz, 1H, Si*H*), 0.99 (s, $^{1}J_{H,Si}$ = 7.0 Hz, 18H, SiC(C*H*$_3$)$_3$).

^{13}C{^{1}H} NMR (126 MHz, C_6D_6, 298 K): δ/ppm = 27.1 (SiC(*C*H$_3$)$_3$), 20.7 (Si*C*(CH$_3$)$_3$).

^{29}Si{^{1}H} DEPT NMR (99 MHz, C_6D_6, 298 K, optimized for $J_{H,Si}$ = 200 Hz, 90.0°): δ/ppm = 27.3.

IR (ATR): $\tilde{\nu}$/cm^{-1} = 2933 (m), 2860 (m), 2133 (m, Si–H), 1468 (m), 1365 (w), 1011 (w), 938 (w), 817 (s), 777 (s).

HRMS (APCI): *m*/*z* calculated for $C_8H_{19}Si^+$ [M–Cl]$^+$: 143.1251; found: 143.1248.

3.18 Bromodi-*tert*-butylsilane (**6cd**)

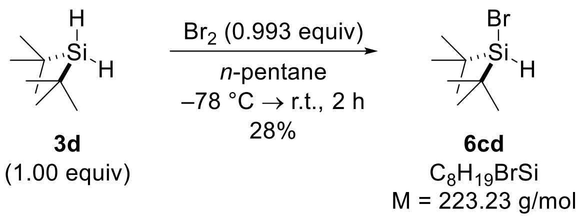

To a solution of di-*tert*-butylsilane (**3d**, 5.75 mL, 4.26 g, 29.5 mmol, 1.00 equiv) in *n*-pentane (45 mL), bromine (1.50 mL, 4.68 g, 29.3 mmol, 0.993 equiv) was added dropwise at –78 °C. The reaction mixture was allowed to warm to room temperature and stirred for additional 2 h. The solvent was removed under reduced pressure, and the crude product was purified by vacuum distillation (b.p. 82 °C at 30 mbar). Bromodi-*tert*-butylsilane (**6cd**, 1.85 g, 8.29 mmol, 28%) was obtained as a colorless oil, which was stored in a glovebox after degassing by three freeze-pump-thaw cycles.

Caution: Slow addition of bromine at –78 °C is advisable to avoid abrupt HBr evolution. The product was found to decompose rapidly and therby act corrosive to metals when handled outside of the glovebox due to release of HBr by hydrolysis.

^{1}H NMR (500 MHz, C_6D_6, 298 K): δ/ppm = 4.34 (s, $^{1}J_{H,Si}$ = 211.9 Hz, 1H, Si*H*), 1.00 (s, 18H, SiC(C*H*$_3$)$_3$).

^{13}C{^{1}H} NMR (126 MHz, C_6D_6, 298 K): δ/ppm = 27.4 (SiC(*C*H$_3$)$_3$), 20.7 (Si*C*(CH$_3$)$_3$).

^{29}Si{^{1}H} DEPT NMR (99 MHz, C_6D_6, 298 K, optimized for $J_{H,Si}$ = 7 Hz, 13.6°): δ/ppm = 28.3.

IR (ATR): $\tilde{\nu}$/cm^{-1} = 2964 (m), 2933 (m), 2892 (m), 2860 (m), 2132 (m, Si–H), 1467 (m), 1390 (w), 1365 (m), 1189 (w), 1010 (m), 938 (w), 811 (s), 758 (s).

HRMS (APCI): *m*/*z* calculated for $C_8H_{18}BrSi^+$ [M–H]$^+$: 221.0356; found: 221.0358.

3.19 Di-*tert*-butyliodosilane (**6dd**)

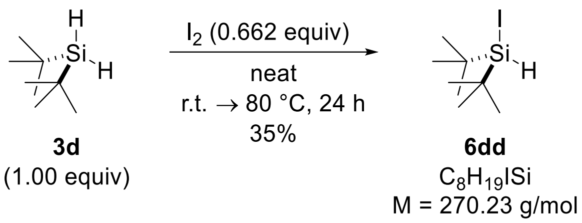

Iodine (5.59 g, 22.0 mmol, 0.662 equiv) was added to di-*tert*-butylsilane (**3d**, 6.50 mL, 4.80 g, 33.3 mmol, 1.00 equiv), and the reaction mixture was vigorously sitirred for 25 h at room temperature and then 1 h at 80 °C. Di-*tert*-butyliodosilane (**6dd**, 3.16 g, 11.7 mmol, 35%) was obtained by vacuum distillation (b.p. 73 °C at 8 mbar) as a colorless oil, which was stored in a glovebox after degassing by three freeze-pump-thaw cycles.

Caution: An ice bath sould be kept at hand during this reaction to counteract abrupt HI evolution. The product was found to decompose rapidly and therby act corrosive to metals when handled outside of the glovebox due to release of HI by hydrolysis.

^{1}H NMR (500 MHz, C_6D_6, 298 K): δ/ppm = 4.37 (s, $^1J_{H,Si}$ = 209.5 Hz, 1H, Si*H*), 1.02 (s, 18H, SiC(C*H*$_3$)$_3$).

^{13}C{^{1}H} NMR (126 MHz, C_6D_6, 298 K): δ/ppm = 28.0 (SiC(*C*H$_3$)$_3$), 20.1 (Si*C*(CH$_3$)$_3$).

^{29}Si{^{1}H} DEPT NMR (99 MHz, C_6D_6, 298 K, optimized for $J_{H,Si}$ = 7 Hz, 13.6°): δ/ppm = 21.7.

HRMS (APCI): *m*/*z* calculated for $C_8H_{18}ISi^+$ [M–H]$^+$: 269.0217; found: 269.0216.

3.20 Difluoromethyl(phenyl)silane (**S11**)

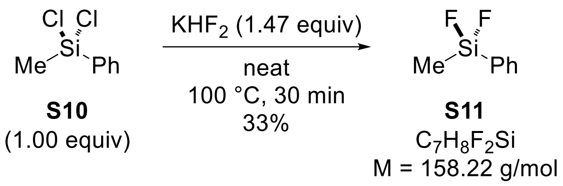

Potassium bifluoride (5.00 g, 64.0 mmol, 1.47 equiv) was added to dichloromethyl(phenyl)-silane (**S10**, 7.00 mL, 8.31 g, 43.5 mmol, 1.00 equiv) and the reaction mixture was vigorously stirred for 30 min at 100 °C. Difluoromethyl(phenyl)silane (**S11**, 2.24 g, 14.2 mmol, 33%) was obtained by distillation at atmospheric pressure (b.p. 130 °C) as a colorless oil, which was stored in a glovebox after degassing by three freeze-pump-thaw cycles.

^{1}H NMR (500 MHz, C_6D_6, 298 K): δ/ppm = 7.44 (m_c, 2H, *o*-C*H*-Ph), 7.14 (m_c, 1H, *p*-C*H*-Ph), 7.06 (m_c, 2H, *m*-C*H*-Ph), 0.16 (t, $^{3}J_{H,F}$ = 6.0 Hz, 3H, SiC*H*$_3$).

^{13}C{^{1}H} NMR (126 MHz, C_6D_6, 298 K): δ/ppm = 133.7 (*o*-*C*H-Ph), 131.9 (*p*-*C*H-Ph), 130.2 (t, $^{2}J_{C,F}$ = 19.4 Hz, *i*-C-Ph), 128.5 (*m*-*C*H-Ph), –5.2 (t, $^{2}J_{C,F}$ = 17.1 Hz, Si*C*H$_3$).

^{19}F NMR (471 MHz, C_6D_6, 298 K): δ/ppm = –136.3 (q, $^{3}J_{F,H}$ = 6.0 Hz, $^{1}J_{F,Si}$ = 290.8 Hz).

^{29}Si{^{1}H} IG NMR (99 MHz, C_6D_6, 298 K): δ/ppm = –12.0 (t, $^{1}J_{Si,F}$ = 291.0 Hz).

IR (ATR): $\tilde{\nu}$/cm^{-1} = 3076 (w), 1594 (w), 1431 (m), 1405 (w), 1268 (m), 1128 (s), 906 (s), 866 (vs), 791 (vs), 727 (vs), 695 (vs), 671 (m).

GLC-MS (EI): *m*/*z* = 158.0 (M^+), 143.0 ([M–Me]$^+$, 100%), 91.0, 81.0, 77.0, 51.0.

HRMS (LIFDI): *m*/*z* calculated for $C_7H_8F_2Si^+$ [M]$^+$: 158.0358; found: 158.0358.

3.21 Dichloroisopropylsilane (**S12**)

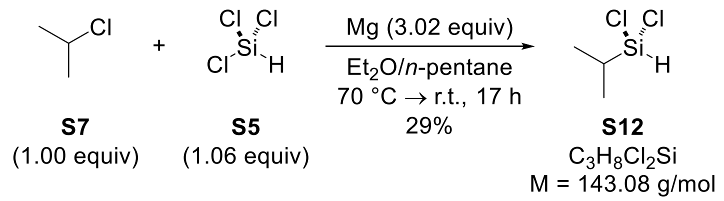

Magnesium turnings (14.4 g, 594 mmol, 3.02 equiv) were heated with vigorous stirring under vacuum (2 times) and suspended in diethyl ether (150 mL). 2-Chloropropane (**S7**, 18.0 mL, 15.5 g, 197 mmol, 1.00 equiv) was added dropwise over 30 min to maintain reflux. The reaction mixture was stirred for 30 min at 70 °C and allowed to warm room temperature. The reaction mixture was then added dropwise to a solution of trichlorosilane (**S5**, 21.0 mL, 28.1 g, 208 mmol, 1.06 equiv) in *n*-pentane (350 mL), and the mixture was stirred for additional 14 h at room temperature. The reaction mixture was filtered, and the residue was washed with *n*-pentane (3 × 100 mL). The solvent was removed under reduced pressure, and the crude product was purified by distillation at atmospheric pressure (b.p. 96 °C). Dichloroisopropyl-silane (**S12**, 8.05 g, 56.3 mmol, 29%) was obtained as a colorless oil and stored in a glovebox after degassing by three freeze-pump-thaw cycles.

^{1}H NMR (500 MHz, C_6D_6, 298 K): δ/ppm = 5.16 (s, $^1J_{H,Si}$ = 273.4 Hz, 1H, Si*H*), 0.79 (s, 7H, SiC*H*(C*H*$_3$)$_2$).

^{13}C{^{1}H} NMR (126 MHz, C_6D_6, 298 K): δ/ppm = 18.3 (Si*C*H(CH$_3$)$_2$), 15.0 (SiCH(*C*H$_3$)$_2$).

^{29}Si{^{1}H} DEPT NMR (99 MHz, C_6D_6, 298 K, optimized for $J_{H,Si}$ = 7 Hz, 22.2°): δ/ppm = 13.7.

IR (ATR): $\tilde{\nu}$/cm^{-1} = 2956 (m), 2871 (m), 2199 (m, Si–H), 1464 (m), 1389 (w), 1240 (w), 1072 (w), 1005 (m), 924 (w), 879 (m), 788 (vs), 670 (m).

HRMS (LIFDI): *m*/*z* calculated for $C_3H_{10}ClOSi^+$ [M–Cl+H_2O]$^+$: 125.0179; found: 125.0182.

3.22 *tert*-Butyldichloro(phenyl)silane (**S13**)

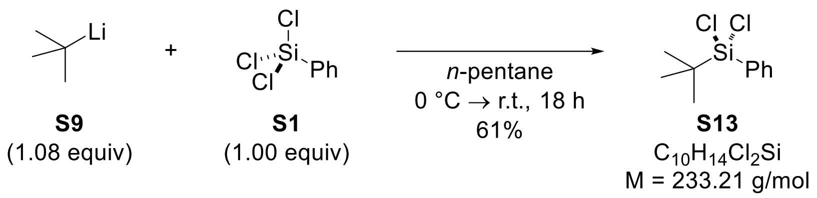

A solution of trichloro(phenyl)silane (**S1**, 24.0 mL, 31.7 g, 150 mmol, 1.00 equiv) in *n*-pentane (200 mL) was cooled to 0 °C, and *tert*-butyllithium (**S9**, 1.90 M in *n*-pentane, 85.0 mL, 162 mmol, 1.08 equiv) was added dropwise. The reaction mixture was allowed to warm to room temperature and stirred for additional 18 h. The reaction mixture was filtered and concentrated under reduced pressure. *tert*-Butyldichloro(phenyl)silane (**S13**, 21.3 g, 91.3 mmol, 61%) was obtained by vacuum distillation (b.p. 103 °C at 8 mbar) as a colorless oil, which was stored in a glovebox after degassing by three freeze-pump-thaw cycles.

^{1}H NMR (500 MHz, C_6D_6, 298 K): δ/ppm = 7.68 (m_c, 2H, *o*-C*H*-Ph), 7.14–7.04 (m, 3H, *m*-C*H*-Ph, *p*-C*H*-Ph), 0.97 (s, 9H, SiC(CH_3)$_3$).

^{13}C{^{1}H} NMR (126 MHz, C_6D_6, 298 K): δ/ppm = 134.8 (*o*-*C*H-Ph), 131.6 (*p*-*C*H-Ph), 130.9 (*i*-C-Ph), 128.3 (*m*-*C*H-Ph), 24.9 (SiC(*C*H_3)$_3$), 23.2 (Si*C*(CH_3)$_3$).

^{29}Si{^{1}H} DEPT NMR (99 MHz, C_6D_6, 298 K, optimized for $J_{H,Si}$ = 7 Hz, 17.5°): δ/ppm = 23.0.

IR (ATR): $\tilde{\nu}$/cm^{-1} = 3074 (w), 2960 (m), 2936 (m), 2896 (w), 2863 (m), 1590 (w), 1462 (m), 1428 (m), 1394 (w), 1364 (m), 1191 (w), 1111 (vs), 1007 (m), 940 (w), 819 (s), 739 (s), 708 (vs), 693 (vs).

HRMS (APCI): *m*/*z* calculated for $C_{10}H_{14}ClSi^+$ [M–Cl]$^+$: 197.0548; found: 197.0549.

3.23 *tert*-Butyldifluoro(phenyl)silane (**S14**)

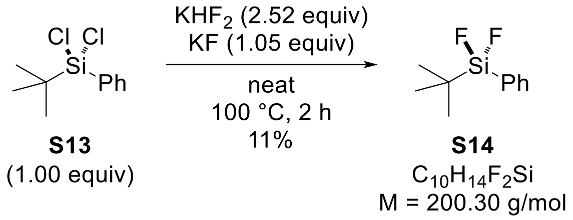

Potassium bifluoride (4.20 g, 53.8 mmol, 2.52 equiv) and potassium fluoride (1.30 g, 22.4 mmol, 1.05 equiv) were added to *tert*-butyldichloro(phenyl)silane (**S13**, 4.50 mL, 4.97 g, 21.3 mmol, 1.00 equiv), and the reaction mixture was vigorously stirred for 2 h at 100 °C. *tert*-Butyldifluoro(phenyl)silane (**S14**, 460 mg, 2.30 mmol, 11%) was obtained by vacuum distillation (b.p. 67 °C at 13 mbar) as a colorless oil, which was stored in a glovebox after degassing by three freeze-pump-thaw cycles.

^{1}H NMR (500 MHz, CD_2Cl_2, 298 K): δ/ppm = 7.68 (d, $^{3}J_{H,H}$ = 7.1 Hz, 2H, *o*-C*H*-Ph), 7.55 (t, $^{3}J_{H,H}$ = 7.5 Hz, 1H, *p*-C*H*-Ph), 7.46 (t, $^{3}J_{H,H}$ = 7.4 Hz, 2H, *m*-C*H*-Ph), 1.08 (s, 9H, SiC(CH_3)$_3$).

^{13}C{^{1}H} NMR (126 MHz, CD_2Cl_2, 298 K): δ/ppm = 134.7 (*o*-*C*H-Ph), 132.2 (*p*-*C*H-Ph), 128.6 (*m*-*C*H-Ph), 127.8 (t, $^{2}J_{C,F}$ = 18.2 Hz, *i*-C-Ph), 24.9 (SiC(*C*H_3)$_3$), 18.1 (t, $^{2}J_{C,F}$ = 15.2 Hz, Si*C*(CH_3)$_3$).

^{19}F NMR (471 MHz, CD_2Cl_2, 298 K): δ/ppm = –152.5 (s, $^{1}J_{F,Si}$ = 313.7 Hz).

^{29}Si{^{1}H} DEPT NMR (99 MHz, CD_2Cl_2, 298 K, optimized for $J_{H,Si}$ = 7 Hz, 17.5°): δ/ppm = –18.3 (t, $^{1}J_{Si,F}$ = 313.1 Hz).

IR (ATR): $\tilde{\nu}$/cm^{-1} = 2937 (w), 2864 (w), 1593 (w), 1472 (w), 1430 (w), 1366 (w), 1126 (s), 1009 (w), 900 (s), 850 (vs), 819 (s), 740 (m), 710 (s), 697 (s).

GLC-MS (EI): *m*/*z* = 200.0 (M^+), 184.9 ([M–Me]$^+$), 142.9 ([M–*t*Bu]$^+$), 121.9, 117.0, 91.0, 80.9, 77.0, 57.0 ([M–SiF_2Ph]$^+$), 56.0 (100%), 51.0.

HRMS (APCI): *m*/*z* calculated for $C_4H_9F_2Si^+$ [M–Ph]$^+$: 123.0436; found: 123.0437.

4 Synthesis of silylium ions

4.1 [*i*Pr$_2$HSi(HCB$_{11}$H$_5$Br$_6$)] (**5c**)

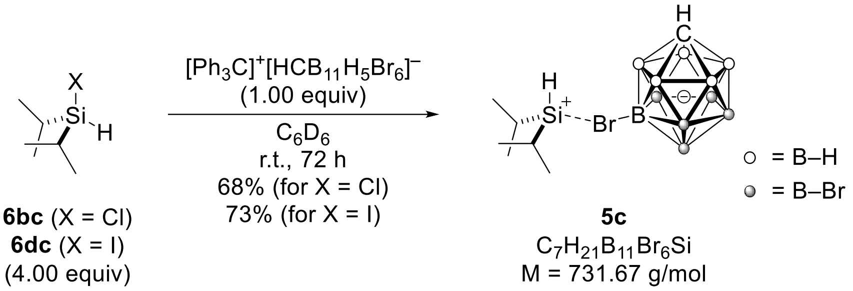

6bc (X = Cl)
6dc (X = I)
(4.00 equiv)

5c
C$_7$H$_{21}$B$_{11}$Br$_6$Si
M = 731.67 g/mol

In a high-quality glovebox (O$_2$, H$_2$O < 1.0 ppm), chlorodiisopropylsilane (**6bc**, 12.0 µL, 10.5 mg, 69.4 µmol, 3.99 equiv) or iododiisopropylsilane (**6dc**, 12.8 µL, 16.8 mg, 69.4 µmol, 3.99 equiv) was added to a suspension of [Ph$_3$C]$^+$[HCB$_{11}$H$_5$Br$_6$]$^-$ (15.0 mg, 17.4 µmol, 1.00 equiv) in C$_6$D$_6$ (0.2 mL). After stirring the reaction mixture for 72 h at room temperature, *n*-hexane (0.4 mL) was added. The suspension was filtered, and the residue was washed with *n*-hexane (3 × 0.2 mL) and dried for 10 min under high vacuum (~10^{-3} mbar). [*i*Pr$_2$HSi(HCB$_{11}$H$_5$Br$_6$)] (**5c**, 68% for X = Cl; 73% for X = I) was obtained as an off-white solid independent of the starting halosilane.

^{1}H NMR (500 MHz, 1,2-C$_6$D$_4$Cl$_2$, 298 K): δ/ppm = 5.27 (t, $^3J_{H,H}$ = 2.1 Hz, $^1J_{H,Si}$ = 240.3 Hz, 1H, Si*H*), 3.24–2.00 (m, 5H, [HCB$_{11}$*H*$_5$Br$_6$]$^-$), 2.65 (s br, 1H, [*H*CB$_{11}$H$_5$Br$_6$]$^-$), 1.42 (sept d, $^3J_{H,H}$ = 7.5 Hz, $^3J_{H,H}$ = 2.1 Hz, 2H, SiC*H*(CH$_3$)$_2$), 1.04 (d, $^3J_{H,H}$ = 7.5 Hz, 12H, SiCH(C*H*$_3$)$_2$).

^{11}B NMR (160 MHz, 1,2-C$_6$D$_4$Cl$_2$, 298 K): δ/ppm = –1.6 (s), –9.1 (s br), –20.1 (d br, $^1J_{B,H}$ = 132.6 Hz).

^{13}C{^{1}H} NMR (126 MHz, 1,2-C$_6$D$_4$Cl$_2$, 298 K): δ/ppm = 41.5 ([H*C*B$_{11}$H$_5$Br$_6$]$^-$), 16.7 (SiCH(*C*H$_3$)$_2$), 14.9 (Si*C*H(CH$_3$)$_2$).

^{29}Si{^{1}H} DEPT NMR (99 MHz, 1,2-C$_6$D$_4$Cl$_2$, 298 K, optimized for $J_{H,Si}$ = 240 Hz, 90.0°): δ/ppm = 69.7.

Single crystals of [*i*Pr$_2$HSi(HCB$_{11}$H$_5$Br$_6$)] (**5c**) suitable for X-ray diffraction analysis were obtained from a solution of silylium carborate **5c** in 1,2-C$_6$H$_4$Cl$_2$ by vapor diffusion with *n*-hexane at room temperature over 4 days (Supplementary Fig. 6 in Section 6.1). CCDC 2424587 contains the supplementary crystallographic data. These data are provided free of charge by The Cambridge Crystallographic Data Centre.

4.2 General procedure for the generation of halogen-substituted silylium carborates (**GP 1**)

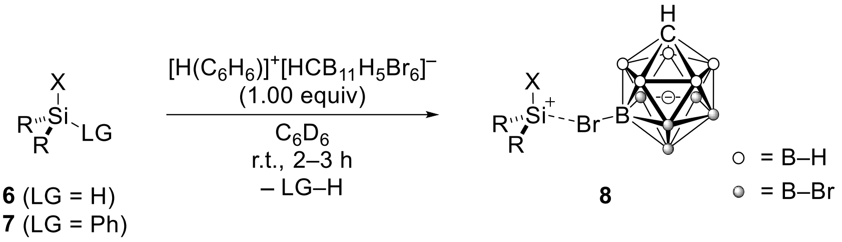

In a high-quality glovebox (O_2, H_2O < 1.0 ppm), the indicated hydrosilane **6** or phenylsilane **7** (1.00 equiv) was added to a suspension of $[H(C_6H_6)]^+[HCB_{11}H_5Br_6]^-$ (1.00 equiv) in C_6D_6 (0.2 mL). After stirring the reaction mixture for 2–3 h at room temperature, *n*-hexane (0.4 mL) was added. The suspension was filtered, and the residue was washed with additional *n*-hexane (3 × 0.2 mL) and dried for 10 min under high vacuum (~10^{-3} mbar). The halogen-substituted silylium carborates **8** were obtained as white solids, which can be stored for several weeks at –30 °C in the glovebox.

4.2.1 [$Me_2FSi(HCB_{11}H_5Br_6)$] (**8aa**)

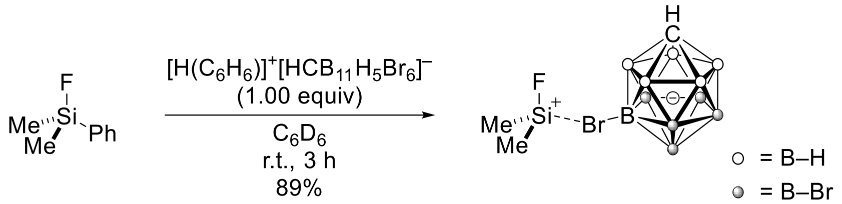

7aa
(1.02 equiv)

8aa
$C_3H_{12}B_{11}Br_6FSi$
M = 693.56 g/mol

Fluorosilylium carborate **8aa** was prepared according to **GP 1** from fluorodimethyl(phenyl)-silane (**7aa**, 3.75 µL, 3.53 mg, 22.9 µmol, 1.02 equiv) and $[H(C_6H_6)]^+[HCB_{11}H_5Br_6]^-$ (15.6 mg, 22.4 µmol, 1.00 equiv). [$Me_2FSi(HCB_{11}H_5Br_6)$] (**8aa**, 13.8 mg, 19.9 µmol, 89%) was obtained as an off-white solid.

^{1}H NMR (500 MHz, 1,2-$C_6D_4Cl_2$, 298 K): δ/ppm = 2.65 (m_c br, 5H, $[HCB_{11}\mathit{H}_5Br_6]^-$), 2.63 (s br, 1H, $[\mathit{H}CB_{11}H_5Br_6]^-$), 1.05 (d, $^3J_{H,F}$ = 6.6 Hz, 6H, $SiC\mathit{H}_3$).

^{11}B NMR (160 MHz, 1,2-$C_6D_4Cl_2$, 298 K): δ/ppm = –1.4 (s), –9.2 (s br), –20.3 (s br).

^{13}C{^{1}H} NMR (126 MHz, 1,2-$C_6D_4Cl_2$, 298 K): δ/ppm = 41.4 ($[H\mathit{C}B_{11}H_5Br_6]^-$), 3.9 (d, $^2J_{C,F}$ = 8.6 Hz, $Si\mathit{C}H_3$).

^{19}F NMR (471 MHz, 1,2-$C_6D_4Cl_2$, 298 K): δ/ppm = –116.7 (s br, $^1J_{F,Si}$ = 369.3 Hz).

^{1}H,^{29}Si HMQC NMR (500/99 MHz, 1,2-$C_6D_4Cl_2$, 298 K, optimized for J = 7 Hz): δ/ppm = 1.05/49.3 (d, $^1J_{Si,F}$ = 369.3 Hz).

4.2.2 [$Et_2FSi(HCB_{11}H_5Br_6)$] (**8ab**)

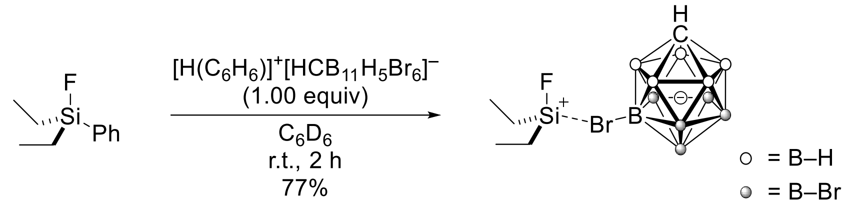

7ab
(1.00 equiv)

8ab
$C_5H_{16}B_{11}Br_6FSi$
M = 721.61 g/mol

Fluorosilylium carborate **8ab** was prepared according to **GP 1** from diethylfluoro(phenyl)silane (**7ab**, 3.00 µL, 2.95 mg, 16.2 µmol, 1.00 equiv) and $[H(C_6H_6)]^+[HCB_{11}H_5Br_6]^-$ (11.3 mg, 16.2 µmol, 1.00 equiv). [$Et_2FSi(HCB_{11}H_5Br_6)$] (**8ab**, 9.00 mg, 12.5 µmol, 77%) was obtained as an off-white solid.

^{1}H NMR (500 MHz, 1,2-$C_6D_4Cl_2$, 298 K): δ/ppm = 2.64 (s br, 1H, $[HCB_{11}H_5Br_6]^-$), 1.36 (qd, $^3J_{H,H}$ = 7.9 Hz, $^3J_{H,F}$ = 6.2 Hz, 4H, $SiCH_2CH_3$), 1.00 (t, $^3J_{H,H}$ = 7.9 Hz, 6H, $SiCH_2CH_3$). Deuterium incorporation into the carborate counteranion and the formation of [$Et_2FSi(HCB_{11}D_5Br_6)$] was observed in 1,2-$C_6D_4Cl_2$.[46]

^{11}B NMR (160 MHz, 1,2-$C_6D_4Cl_2$, 298 K): δ/ppm = –1.5 (s), –9.2 (s br), –20.2 (s br).

^{13}C{^{1}H} NMR (126 MHz, 1,2-$C_6D_4Cl_2$, 298 K): δ/ppm = 41.2 ($[HCB_{11}H_5Br_6]^-$), 10.2 (d, $^2J_{C,F}$ = 7.6 Hz, $SiCH_2CH_3$), 4.8 ($SiCH_2CH_3$).

^{19}F NMR (471 MHz, 1,2-$C_6D_4Cl_2$, 298 K): δ/ppm = –129.0 (s br, $^1J_{F,Si}$ = 379.4 Hz).

^{1}H,^{29}Si HMQC NMR (500/99 MHz, 1,2-$C_6D_4Cl_2$, 298 K, optimized for J = 7 Hz): δ/ppm = 1.36/50.6, 1.00/50.6 (d, $^1J_{Si,F}$ = 379.4 Hz).

A solution of [$Et_2FSi(HCB_{11}H_5Br_6)$] (**8ab**) in 1,2-$C_6D_4Cl_2$ was found to be stable for severals days at room temperature when protected from light. Exposure to sunlight resulted in decomposition to [$Et_2ClSi(HCB_{11}H_5Br_6)$] (**8bb**), as indicated by a shift of the ^{29}Si NMR signal to δ(^{29}Si) = 72.6 ppm. The decomposition was accompanied by a color change from yellow to orange to dark red/brown.

4.2.3 [iPr$_2$FSi(HCB$_{11}$H$_5$Br$_6$)] (**8ac**)

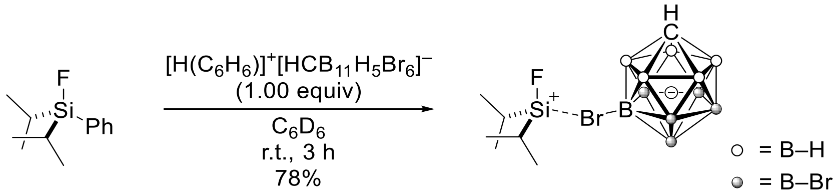

7ac (0.993 equiv)

8ac $C_7H_{20}B_{11}Br_6FSi$ M = 749.66 g/mol

Fluorosilylium carborate **8ac** was prepared according to **GP 1** from fluorodiisopropyl(phenyl)-silane (**7ac**, 6.25 µL, 5.81 mg, 27.6 µmol, 0.993 equiv) and $[H(C_6H_6)]^+[HCB_{11}H_5Br_6]^-$ (19.3 mg, 27.8 µmol, 1.00 equiv). [iPr$_2$FSi(HCB$_{11}$H$_5$Br$_6$)] (**8ac**, 16.2 mg, 21.6 µmol, 78%) was obtained as an off-white solid.

^{1}H NMR (500 MHz, C_6D_6, 298 K): δ/ppm = 2.64 (s br, 1H, $[HCB_{11}H_5Br_6]^-$), 1.65–1.53 (m, 2H, SiCH(CH$_3$)$_2$), 1.06 (d, $^3J_{H,H}$ = 7.5 Hz, 12H, SiCH(CH_3)$_2$). Deuterium incorporation into the carborate counteranion and the formation of [iPr$_2$FSi(HCB$_{11}$D$_5$Br$_6$)] was observed in 1,2-$C_6D_4Cl_2$.[46]

^{11}B NMR (160 MHz, 1,2-$C_6D_4Cl_2$, 298 K): δ/ppm = –1.3 (s), –8.8 (s br), –19.9 (s br).

^{13}C{^{1}H} NMR (126 MHz, 1,2-$C_6D_4Cl_2$, 298 K): δ/ppm = 41.1 ($[HCB_{11}H_5Br_6]^-$), 17.2 (d, $^2J_{C,F}$ = 9.4 Hz, SiCH(CH$_3$)$_2$), 15.2 (SiCH(CH$_3$)$_2$).

^{19}F NMR (471 MHz, 1,2-$C_6D_4Cl_2$, 298 K): δ/ppm = –137.0 (s br, $^1J_{F,Si}$ = 398.6 Hz).

^{1}H,^{29}Si HMQC NMR (500/99 MHz, 1,2-$C_6D_4Cl_2$, 298 K, optimized for J = 7 Hz): δ/ppm = 1.06/47.0 (d, $^1J_{Si,F}$ = 398.6 Hz).

Single crystals of [iPr$_2$FSi(HCB$_{11}$H$_5$Br$_6$)] (**8ac**) suitable for X-ray diffraction analysis were obtained from a solution of silylium carborate **8ac** in 1,2-$C_6H_4F_2$ by vapor diffusion with n-pentane at –30 °C over 4 days (Supplementary Fig. 7 in Section 6.2). CCDC 2424589 contains the supplementary crystallographic data. These data are provided free of charge by The Cambridge Crystallographic Data Centre.

A solution of [iPr$_2$FSi(HCB$_{11}$H$_5$Br$_6$)] (**8ac**) in 1,2-$C_6D_4Cl_2$ was found to be stable for severals days at room temperature when protected from light. Exposure to sunlight resulted in slow decomposition to iPr$_2$SiCl$_2$, as indicated by a shift of the ^{29}Si NMR signal to δ(^{29}Si) = 38.3 ppm. The decomposition was accompanied by a color change from yellow to orange to dark red/brown.

4.2.4 [tBu$_2$FSi(HCB$_{11}$H$_5$Br$_6$)] (**8ad**)

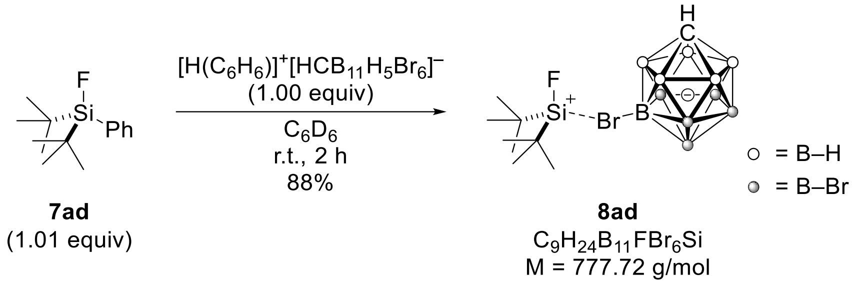

Fluorosilylium carborate **8ad** was prepared according to **GP 1** from di-*tert*-butylfluoro(phenyl)-silane (**7ad**, 3.75 µL, 3.52 mg, 14.8 µmol, 1.01 equiv) and $[H(C_6H_6)]^+[HCB_{11}H_5Br_6]^-$ (10.2 mg, 14.7 µmol, 1.00 equiv). [tBu$_2$FSi(HCB$_{11}$H$_5$Br$_6$)] (**8ad**, 10.1 mg, 13.0 µmol, 88%) was obtained as an off-white solid.

^{1}H NMR (500 MHz, 1,2-$C_6D_4Cl_2$, 298 K): δ/ppm = 2.66 (s br, 1H, $[HCB_{11}H_5Br_6]^-$), 1.07 (s, 18H, SiC(CH_3)$_3$). Deuterium incorporation into the carborate counteranion and the formation of [tBu$_2$FSi(HCB$_{11}$D$_5$Br$_6$)] was observed in 1,2-$C_6D_4Cl_2$.[46]

^{11}B NMR (160 MHz, 1,2-$C_6D_4Cl_2$, 298 K): δ/ppm = –1.7 (s), –9.4 (s br), –20.1 (s br). Anion decomposition was found after several hours in solution, as indicated by the appearance of additional signals in the ^{11}B NMR spectrum.

^{13}C{^{1}H} NMR (126 MHz, 1,2-$C_6D_4Cl_2$, 298 K): δ/ppm = 25.6 (d, $^2J_{C,F}$ = 5.9 Hz, Si*C*(CH$_3$)$_3$), 25.4 (SiC(*C*H$_3$)$_3$).

^{19}F{^{1}H} NMR (471 MHz, 1,2-$C_6D_4Cl_2$, 298 K): δ/ppm = –142.0 (s br, $^1J_{F,Si}$ = 410.5 Hz).

^{29}Si{^{1}H} DEPT NMR (99 MHz, 1,2-$C_6D_4Cl_2$, 298 K, optimized for $J_{H,Si}$ = 7 Hz, 13.6°): δ/ppm = 47.6 (d, $^1J_{Si,F}$ = 410.5 Hz).

Single crystals of [tBu$_2$FSi(HCB$_{11}$H$_5$Br$_6$)] (**8ad**) suitable for X-ray diffraction analysis were obtained from a solution of silylium carborate **8ad** in 1,2-$C_6H_4F_2$ by vapor diffusion with *n*-pentane at room temperature over 4 days (Supplementary Fig. 8 in Section 6.3). CCDC 2424600 contains the supplementary crystallographic data. These data are provided free of charge by The Cambridge Crystallographic Data Centre.

A solution of [tBu$_2$FSi(HCB$_{11}$H$_5$Br$_6$)] (**8ad**) in 1,2-$C_6D_4Cl_2$ was found to be stable for severals days at room temperature when protected from light. Exposure to sunlight resulted in decomposition to [tBu$_2$ClSi(HCB$_{11}$H$_5$Br$_6$)] (**8bd**), tBu$_2$SiCl$_2$, tBu$_2$SiFCl, and tBu$_2$SiF$_2$, as indicated by ^{29}Si NMR spectroscopy. The decomposition was accompanied by a color change from yellow to orange to dark red/brown.

4.2.5 [$Me_2ClSi(HCB_{11}H_5Br_6)$] (**8ba**)

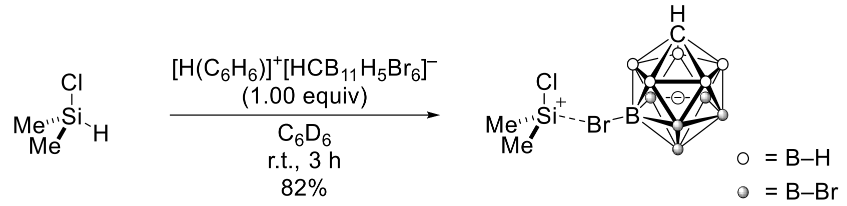

6ba (1.00 equiv)

8ba $C_3H_{12}B_{11}Br_6ClSi$ M = 710.01 g/mol

Chlorosilylium carborate **8ba** was prepared according to **GP 1** from chlorodimethylsilane (**6ba**, 3.25 µL, 2.77 mg, 29.3 µmol, 1.00 equiv) and $[H(C_6H_6)]^+[HCB_{11}H_5Br_6]^-$ (20.4 mg, 29.3 µmol, 1.00 equiv). [$Me_2ClSi(HCB_{11}H_5Br_6)$] (**8ba**, 17.0 mg, 23.9 µmol, 82%) was obtained as an off-white solid.

^{1}H NMR (500 MHz, 1,2-$C_6D_4Cl_2$, 298 K): δ/ppm = 2.63 (s br, 1H, $[HCB_{11}H_5Br_6]^-$), 1.22 (s, 6H, SiCH_3). Deuterium incorporation into the carborate counteranion and the formation of [$Me_2ClSi(HCB_{11}D_5Br_6)$] was observed in 1,2-$C_6D_4Cl_2$.[46]

^{11}B NMR (160 MHz, 1,2-$C_6D_4Cl_2$, 298 K): δ/ppm = –1.3 (s), –9.2 (s br), –20.1 (s br).

^{13}C{^{1}H} NMR (126 MHz, 1,2-$C_6D_4Cl_2$, 298 K): δ/ppm = 41.4 ($[HCB_{11}H_5Br_6]^-$), 7.8 (SiCH_3).

^{29}Si{^{1}H} DEPT NMR (99 MHz, 1,2-$C_6D_4Cl_2$, 298 K, optimized for $J_{H,Si}$ = 7 Hz, 13.6°): δ/ppm = 66.2.

A solution of [$Me_2ClSi(HCB_{11}H_5Br_6)$] (**8ba**) in 1,2-$C_6D_4Cl_2$ was found to be stable for severals days at room temperature when protected from light. Exposure to sunlight resulted in slow decomposition to Me_2SiCl_2, as indicated by a shift of the ^{29}Si NMR signal to δ(^{29}Si) = 32.2 ppm. The decomposition was accompanied by a color change from yellow to orange to dark red/brown.

4.2.6 [$Et_2ClSi(HCB_{11}H_5Br_6)$] (**8bb**)

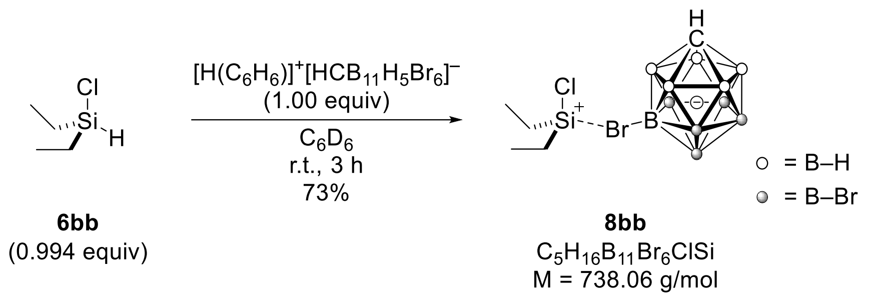

Chlorosilylium carborate **8bb** was prepared according to **GP 1** from chlorodiethylsilane (**6bb**, 4.00 µL, 3.56 mg, 29.0 µmol, 0.994 equiv) and $[H(C_6H_6)]^+[HCB_{11}H_5Br_6]^-$ (20.3 mg, 29.2 µmol, 1.00 equiv). [$Et_2ClSi(HCB_{11}H_5Br_6)$] (**8bb**, 15.7 mg, 21.3 µmol, 73%) was obtained as an off-white solid.

^{1}H NMR (500 MHz, 1,2-$C_6D_4Cl_2$, 298 K): δ/ppm = 2.63 (s br, 1H, $[HCB_{11}H_5Br_6]^-$), 1.48 (q, $^3J_{H,H}$ = 7.4 Hz, 4H, $SiCH_2CH_3$), 1.02 (t, $^3J_{H,H}$ = 7.2 Hz, 6H, $SiCH_2CH_3$). Deuterium incorporation into the carborate counteranion and the formation of [$Et_2ClSi(HCB_{11}D_5Br_6)$] was observed in 1,2-$C_6D_4Cl_2$.[46]

^{11}B NMR (160 MHz, 1,2-$C_6D_4Cl_2$, 298 K): δ/ppm = –1.4 (s), –9.2 (s br), –20.2 (s br).

^{13}C{^{1}H} NMR (126 MHz, 1,2-$C_6D_4Cl_2$, 298 K): δ/ppm = 41.2 ($[HCB_{11}H_5Br_6]^-$), 13.4 ($SiCH_2CH_3$), 5.8 ($SiCH_2CH_3$).

^{1}H,^{29}Si HMQC NMR (500/99 MHz, 1,2-$C_6D_4Cl_2$, 298 K, optimized for J = 7 Hz): δ/ppm = 1.48/72.5, 1.02/72.5.

4.2.7 [*i*$Pr_2ClSi(HCB_{11}H_5Br_6)$] (**8bc**)

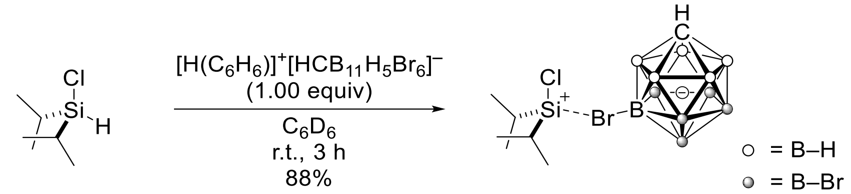

6bc (1.01 equiv) **8bc** $C_7H_{20}B_{11}Br_6ClSi$ M = 766.12 g/mol

Chlorosilylium carborate **8bc** was prepared according to **GP 1** from chlorodiisopropylsilane (**6bc**, 6.00 µL, 5.23 mg, 34.7 µmol, 1.01 equiv) and $[H(C_6H_6)]^+[HCB_{11}H_5Br_6]^-$ (23.9 mg, 34.4 µmol, 1.00 equiv). [*i*$Pr_2ClSi(HCB_{11}H_5Br_6)$] (**8bc**, 23.2 mg, 30.3 µmol, 88%) was obtained as an off-white solid.

^{1}H NMR (500 MHz, C_6D_6, 298 K): δ/ppm = 2.63 (s br, 1H, $[HCB_{11}H_5Br_6]^-$), 1.74 (sept, $^3J_{H,H}$ = 7.3 Hz, 2H, SiC*H*(CH_3)$_2$), 1.08 (d, $^3J_{H,H}$ = 7.4 Hz, 12H, SiCH(C*H*$_3$)$_2$). Deuterium incorporation into the carborate counteranion and the formation of [*i*$Pr_2ClSi(HCB_{11}D_5Br_6)$] was observed in 1,2-$C_6D_4Cl_2$.[46]

^{11}B NMR (160 MHz, 1,2-$C_6D_4Cl_2$, 298 K): δ/ppm = –1.4 (s), –9.2 (s br), –20.4 (s br).

^{13}C{^{1}H} NMR (126 MHz, 1,2-$C_6D_4Cl_2$, 298 K): δ/ppm = 41.1 ([H*C*$B_{11}H_5Br_6$]$^-$), 20.3 (Si*C*H(CH_3)$_2$), 16.0 (SiCH(*C*H_3)$_2$).

^{1}H,^{29}Si HMQC NMR (500/99 MHz, 1,2-$C_6D_4Cl_2$, 298 K, optimized for *J* = 7 Hz): δ/ppm = 1.08/74.3.

Single crystals of [*i*$Pr_2ClSi(HCB_{11}H_5Br_6)$] (**8bc**) suitable for X-ray diffraction analysis were obtained from a solution of silylium carborate **8bc** in 1,2-$C_6H_4Cl_2$ by vapor diffusion with *n*-hexane at room temperature over 4 days (Supplementary Fig. 9 in Section 6.4). CCDC 2424591 contains the supplementary crystallographic data. These data are provided free of charge by The Cambridge Crystallographic Data Centre.

A solution of [*i*$Pr_2ClSi(HCB_{11}H_5Br_6)$] (**8bc**) in 1,2-$C_6D_4Cl_2$ was found to be stable for severals days at room temperature when protected from light. Exposure to sunlight resulted in immediate decomposition to *i*Pr_2SiCl_2, as indicated by a shift of the ^{29}Si NMR signal to δ(^{29}Si) = 37.9 ppm. The decomposition was accompanied by a color change from yellow to orange to dark red/brown.

4.2.8 [tBu$_2$ClSi(HCB$_{11}$H$_5$Br$_6$)] (**8bd**)

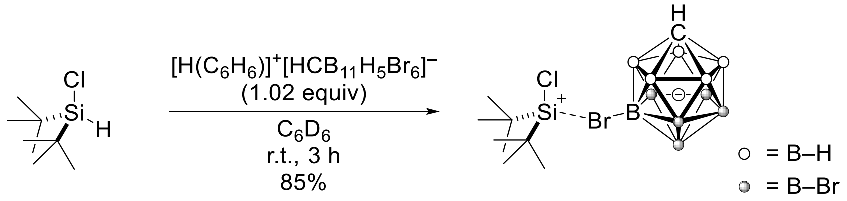

6bd (1.00 equiv) **8bd** $C_9H_{24}B_{11}Br_6ClSi$ M = 794.17 g/mol

Chlorosilylium carborate **8bd** was prepared according to **GP 1** from di-*tert*-butylchlorosilane (**6bd**, 7.50 µL, 6.63 mg, 37.1 µmol, 1.00 equiv) and $[H(C_6H_6)]^+[HCB_{11}H_5Br_6]^-$ (26.3 mg, 37.8 µmol, 1.02 equiv). [tBu$_2$ClSi(HCB$_{11}$H$_5$Br$_6$)] (**8bd**, 25.1 mg, 31.6 µmol, 85%) was obtained as an off-white solid.

^{1}H NMR (500 MHz, 1,2-$C_6D_4Cl_2$, 298 K): δ/ppm = 2.66 (s br, 1H, [HCB$_{11}$H$_5$Br$_6$]$^-$), 1.07 (s, 18H, SiC(CH_3)$_3$). Deuterium incorporation into the carborate counteranion and the formation of [tBu$_2$ClSi(HCB$_{11}$D$_5$Br$_6$)] was observed in 1,2-$C_6D_4Cl_2$.[46]

^{11}B NMR (160 MHz, 1,2-$C_6D_4Cl_2$, 298 K): δ/ppm = –1.2 (s), –9.4 (s br), –19.8 (s br).

^{13}C{^{1}H} NMR (126 MHz, 1,2-$C_6D_4Cl_2$, 298 K): δ/ppm = 28.9 (SiC(CH$_3$)$_3$), 26.0 (SiC(CH$_3$)$_3$).

^{29}Si{^{1}H} DEPT NMR (99 MHz, 1,2-$C_6D_4Cl_2$, 298 K, optimized for $J_{H,Si}$ = 7 Hz, 13.6°): δ/ppm = 80.5.

A solution of [tBu$_2$ClSi(HCB$_{11}$H$_5$Br$_6$)] (**8bd**) in 1,2-$C_6D_4Cl_2$ was found to be stable for severals days at room temperature when protected from light. Exposure to sunlight resulted in immediate decomposition to tBu$_2$SiCl$_2$, as indicated by a shift of the ^{29}Si NMR signal to δ(^{29}Si) = 39.0 ppm. The decomposition was accompanied by a color change from yellow to orange to dark red/brown.

4.2.9 [$Me_2BrSi(HCB_{11}H_5Br_6)$] (**8ca**)

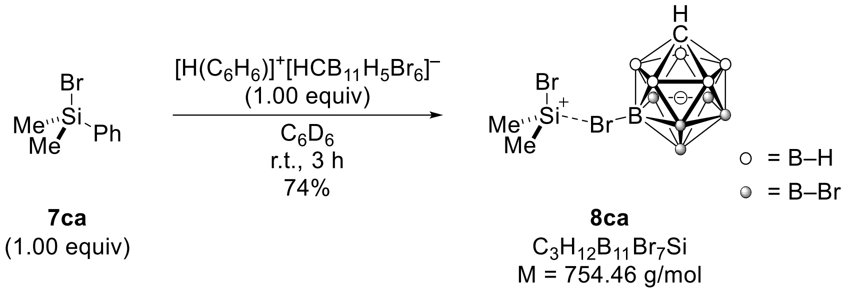

Bromosilylium carborate **8ca** was prepared according to **GP 1** from bromodimethyl(phenyl)-silane (**7ca**, 3.00 µL, 3.76 mg, 17.5 µmol, 1.00 equiv) and $[H(C_6H_6)]^+[HCB_{11}H_5Br_6]^-$ (12.2 mg, 17.5 µmol, 1.00 equiv). [$Me_2BrSi(HCB_{11}H_5Br_6)$] (**8ca**, 9.80 mg, 13.0 µmol, 74%) was obtained as an off-white solid.

^{1}H NMR (500 MHz, 1,2-$C_6D_4Cl_2$, 298 K): δ/ppm = 2.64 (s br, 1H, $[HCB_{11}H_5Br_6]^-$), 2.60 (m_c br, 5H, $[HCB_{11}H_5Br_6]^-$), 1.38 (s, $^1J_{H,C}$ = 126.0 Hz, 6H, $SiCH_3$).

^{11}B NMR (160 MHz, 1,2-$C_6D_4Cl_2$, 298 K): δ/ppm = –1.3 (s), –9.1 (s br), –20.1 (d br, $^1J_{BH}$ = 120.0 Hz).

^{13}C{^{1}H} NMR (126 MHz, 1,2-$C_6D_4Cl_2$, 298 K): δ/ppm = 41.5 ($[HCB_{11}H_5Br_6]^-$), 9.2 ($SiCH_3$).

^{29}Si{^{1}H} DEPT NMR (99 MHz, 1,2-$C_6D_4Cl_2$, 298 K, optimized for $J_{H,Si}$ = 7 Hz, 24.1°): δ/ppm = 62.3.

4.2.10 $[Et_2BrSi(HCB_{11}H_5Br_6)]$ (**8cb**)

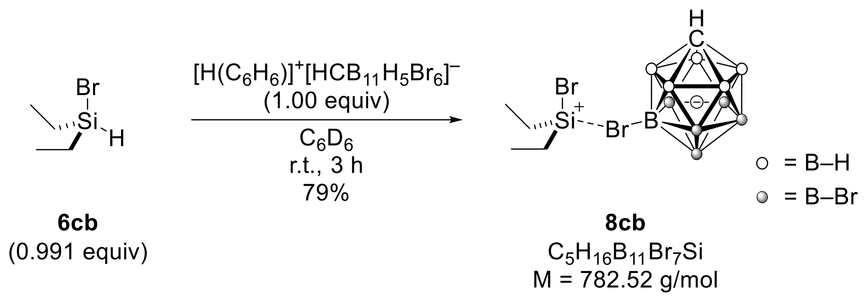

Bromosilylium carborate **8cb** was prepared according to **GP 1** from bromodiethylsilane (**6cb**, 4.00 µL, 4.76 mg, 28.5 µmol, 0.991 equiv) and $[H(C_6H_6)]^+[HCB_{11}H_5Br_6]^-$ (20.0 mg, 28.8 µmol, 1.00 equiv). $[Et_2BrSi(HCB_{11}H_5Br_6)]$ (**8cb**, 17.7 mg, 22.6 µmol, 79%) was obtained as an off-white solid.

^{1}H NMR (500 MHz, 1,2-$C_6D_4Cl_2$, 298 K): δ/ppm = 2.63 (s br, 1H, $[\mathit{H}CB_{11}H_5Br_6]^-$), 1.62–1.51 (m, 4H, $SiC\mathit{H}_2CH_3$), 1.05–0.95 (m, 6H, $SiCH_2C\mathit{H}_3$). Deuterium incorporation into the carborate counteranion and the formation of $[Et_2BrSi(HCB_{11}D_5Br_6)]$ was observed in 1,2-$C_6D_4Cl_2$.[46]

^{11}B NMR (160 MHz, 1,2-$C_6D_4Cl_2$, 298 K): δ/ppm = –1.2 (s), –8.9 (s br), –20.1 (s br).

^{13}C{^{1}H} NMR (126 MHz, 1,2-$C_6D_4Cl_2$, 298 K): δ/ppm = 41.2 ($[H\mathit{C}B_{11}H_5Br_6]^-$), 14.4 ($Si\mathit{C}H_2CH_3$), 6.4 ($SiCH_2\mathit{C}H_3$).

^{1}H,^{29}Si HMQC NMR (500/99 MHz, 1,2-$C_6D_4Cl_2$, 298 K, optimized for J = 7 Hz): δ/ppm = 1.58/73.5, 1.02/73.5.

4.2.11 [iPr$_2$BrSi(HCB$_{11}$H$_5$Br$_6$)] (**8cc**)

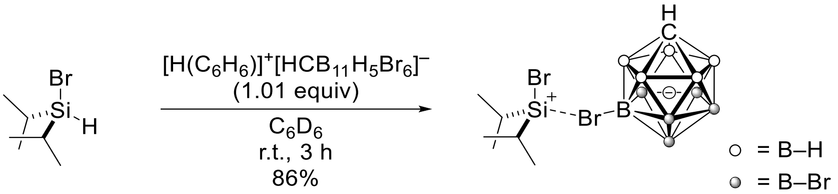

6cc
(1.00 equiv)

8cc
$C_7H_{20}B_{11}Br_7Si$
M = 810.57 g/mol

Bromosilylium carborate **8cc** was prepared according to **GP 1** from bromodiisopropylsilane (**6cc**, 10.0 µL, 11.1 mg, 56.8 µmol, 1.00 equiv) and [H(C$_6$H$_6$)]$^+$[HCB$_{11}$H$_5$Br$_6$]$^-$ (40.0 mg, 57.5 µmol, 1.01 equiv). [iPr$_2$BrSi(HCB$_{11}$H$_5$Br$_6$)] (**8cc**, 39.7 mg, 49.0 µmol, 86%) was obtained as an off-white solid.

^{1}H NMR (500 MHz, 1,2-C$_6$D$_4$Cl$_2$, 298 K): δ/ppm = 2.63 (s br, 1H, [HCB$_{11}$H$_5$Br$_6$]$^-$), 1.84 (sept, $^3J_{H,H}$ = 7.4 Hz, 2H, SiCH(CH$_3$)$_2$), 1.07 (d, $^3J_{H,H}$ = 7.4 Hz, 12H, SiCH(CH_3)$_2$). Deuterium incorporation into the carborate counteranion and the formation of [iPr$_2$BrSi(HCB$_{11}$D$_5$Br$_6$)] was observed in 1,2-C$_6$D$_4$Cl$_2$.[46]

^{11}B NMR (160 MHz, 1,2-C$_6$D$_4$Cl$_2$, 298 K): δ/ppm = –1.4 (s), –9.4 (s br), –20.7 (s br).

^{13}C{^{1}H} NMR (126 MHz, 1,2-C$_6$D$_4$Cl$_2$, 298 K): δ/ppm = 41.1 ([HCB$_{11}$H$_5$Br$_6$]$^-$), 21.1 (SiCH(CH$_3$)$_2$), 16.5 (SiCH(CH$_3$)$_2$).

^{1}H,^{29}Si HMQC NMR (500/99 MHz, 1,2-C$_6$D$_4$Cl$_2$, 298 K, optimized for J = 7 Hz): δ/ppm = 1.84/79.3, 1.07/79.3.

Single crystals of [iPr$_2$BrSi(HCB$_{11}$H$_5$Br$_6$)] (**8cc**) suitable for X-ray diffraction analysis were obtained from a solution of silylium carborate **8cc** in 1,2-C$_6$H$_4$F$_2$ by vapor diffusion with n-pentane at –30 °C over 4 days (Supplementary Fig. 10 in Section 6.5). CCDC 2424593 contains the supplementary crystallographic data. These data are provided free of charge by The Cambridge Crystallographic Data Centre.

A solution of [iPr$_2$BrSi(HCB$_{11}$H$_5$Br$_6$)] (**8cc**) in 1,2-C$_6$D$_4$Cl$_2$ was found to be stable for severals days at room temperature when protected from light. Exposure to sunlight resulted in immediate decomposition to iPr$_2$SiCl$_2$, as indicated by a shift of the ^{29}Si NMR signal to δ(^{29}Si) = 37.7 ppm. The decomposition was accompanied by a color change from yellow to orange to dark red/brown.

4.2.12 [*t*$Bu_2BrSi(HCB_{11}H_5Br_6)$] (**8cd**)

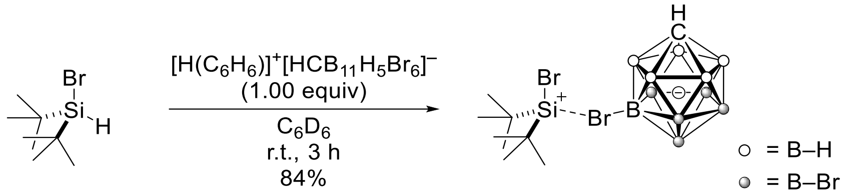

6cd
(1.00 equiv)

8cd
$C_9H_{24}B_{11}Br_7Si$
M = 838.62 g/mol

Bromosilylium carborate **8cd** was prepared according to **GP 1** from bromodi-*tert*-butylsilane (**6cd**, 6.00 µL, 6.42 mg, 28.8 µmol, 1.00 equiv) and $[H(C_6H_6)]^+[HCB_{11}H_5Br_6]^-$ (20.1 mg, 28.9 µmol, 1.00 equiv). [*t*$Bu_2BrSi(HCB_{11}H_5Br_6)$] (**8cd**, 20.2 mg, 24.1 µmol, 84%) was obtained as an off-white solid.

^{1}H NMR (500 MHz, 1,2-$C_6D_4Cl_2$, 298 K): δ/ppm = 2.67 (s br, 1H, [*H*$CB_{11}H_5Br_6]^-$), 1.09 (s, 18H, SiC(C*H*$_3$)$_3$). Deuterium incorporation into the carborate counteranion and the formation of [*t*$Bu_2BrSi(HCB_{11}D_5Br_6)$] was observed in 1,2-$C_6D_4Cl_2$.[46]

^{11}B NMR (160 MHz, 1,2-$C_6D_4Cl_2$, 298 K): δ/ppm = –1.1 (s), –8.4 (s br), –19.8 (s br). Anion decomposition was found after several hours in solution, as indicated by the appearance of additional signals in the ^{11}B NMR spectrum.

^{13}C{^{1}H} NMR (126 MHz, 1,2-$C_6D_4Cl_2$, 298 K): δ/ppm = 40.9 ([H*C*$B_{11}H_5Br_6]^-$), 30.0 (Si*C*$(CH_3)_3$), 26.4 (SiC(*C*$H_3)_3$).

^{29}Si{^{1}H} DEPT NMR (99 MHz, 1,2-$C_6D_4Cl_2$, 298 K, optimized for $J_{H,Si}$ = 7 Hz, 13.6°): δ/ppm = 88.5.

A solution of [*t*$Bu_2BrSi(HCB_{11}H_5Br_6)$] (**8cd**) in 1,2-$C_6D_4Cl_2$ was found to be stable for severals days at room temperature when protected from light. Exposure to sunlight resulted in decomposition to *t*Bu_2SiCl_2, *t*$Bu_2SiBrCl$, and *t*Bu_2SiBr_2, as indicated by ^{29}Si NMR spectroscopy. The decomposition was accompanied by a color change from yellow to orange to dark red/brown.

4.2.13 $[Me_2ISi(HCB_{11}H_5Br_6)]$ (**8da**)

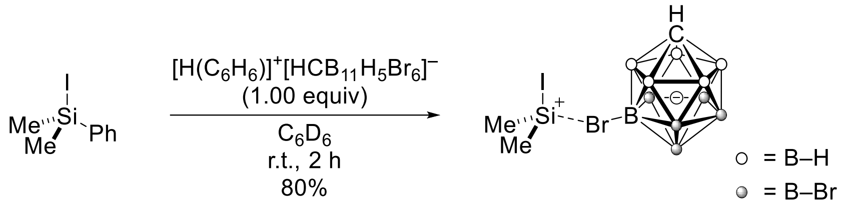

7da (1.00 equiv)

8da $C_3H_{12}B_{11}Br_6ISi$ M = 801.46 g/mol

Iodosilylium carborate **8da** was prepared according to **GP 1** from iododimethyl(phenyl)silane (**7da**, 3.00 µL, 4.37 mg, 16.7 µmol, 1.00 equiv) and $[H(C_6H_6)]^+[HCB_{11}H_5Br_6]^-$ (11.6 mg, 16.7 µmol, 1.00 equiv). $[Me_2ISi(HCB_{11}H_5Br_6)]$ (**8da**, 10.7 mg, 13.4 µmol, 80%) was obtained as an off-white solid.

^{1}**H NMR** (500 MHz, 1,2-$C_6D_4Cl_2$, 298 K): δ/ppm = 2.64 (s br, 1H, $[HCB_{11}H_5Br_6]^-$), 1.61 (s, 6H, $^1J_{H,C}$ = 127.3 Hz, $SiCH_3$). Deuterium incorporation into the carborate counteranion and the formation of $[Me_2ISi(HCB_{11}D_5Br_6)]$ was observed in 1,2-$C_6D_4Cl_2$.[46]

^{11}**B NMR** (160 MHz, 1,2-$C_6D_4Cl_2$, 298 K): δ/ppm = –1.3 (s), –9.1 (s br), –20.4 (s br).

^{13}**C{^{1}H} NMR** (126 MHz, 1,2-$C_6D_4Cl_2$, 298 K): δ/ppm = 41.4 ($[HCB_{11}H_5Br_6]^-$), 11.3 ($SiCH_3$).

^{1}**H,^{29}Si HMQC NMR** (500/99 MHz, 1,2-$C_6D_4Cl_2$, 298 K, optimized for J = 7 Hz): δ/ppm = 1.61/41.7.

A solution of $[Me_2ISi(HCB_{11}H_5Br_6)]$ (**8da**) in 1,2-$C_6D_4Cl_2$ was found to be stable for severals hours at room temperature when protected from light. Exposure to sunlight resulted in slow decomposition to $[Me_2BrSi(HCB_{11}H_5Br_6)]$ (**8ca**, δ(^{29}Si) = 62.3 ppm) and $[Me_2ClSi(HCB_{11}H_5Br_6)]$ (**8ba**, δ(^{29}Si) = 66.4 ppm), as indicated by ^{29}Si NMR spectroscopy. The decomposition was accompanied by a color change from yellow to orange to dark red/brown.

4.2.14 $[Et_2ISi(HCB_{11}H_5Br_6)]$ (**8db**)

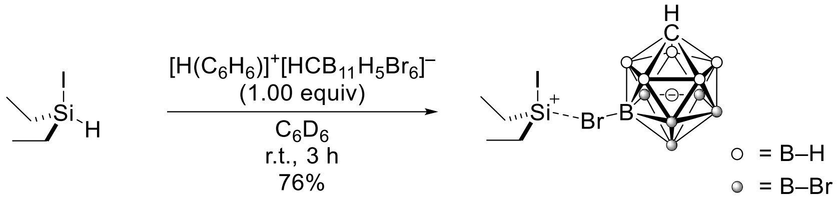

Iodosilylium carborate **8db** was prepared according to **GP 1** from diethyliodosilane (**6db**, 4.25 µL, 6.02 mg, 28.1 µmol, 0.992 equiv) and $[H(C_6H_6)]^+[HCB_{11}H_5Br_6]^-$ (19.7 mg, 28.3 µmol, 1.00 equiv). $[Et_2ISi(HCB_{11}H_5Br_6)]$ (**8db**, 17.8 mg, 21.5 µmol, 76%) was obtained as an off-white solid.

^{1}H NMR (500 MHz, 1,2-$C_6D_4Cl_2$, 298 K): δ/ppm = 2.64 (s br, 1H, $[HCB_{11}H_5Br_6]^-$), 1.71 (q, $^3J_{H,H}$ = 7.5 Hz, 4H, $SiCH_2CH_3$), 0.99 (t, $^3J_{H,H}$ = 7.7 Hz, 6H, $SiCH_2CH_3$). Deuterium incorporation into the carborate counteranion and the formation of $[Et_2ISi(HCB_{11}D_5Br_6)]$ was observed in 1,2-$C_6D_4Cl_2$.[46]

^{11}B NMR (160 MHz, 1,2-$C_6D_4Cl_2$, 298 K): δ/ppm = –1.3 (s), –9.1 (s br), –20.3 (s br).

^{13}C{^{1}H} NMR (126 MHz, 1,2-$C_6D_4Cl_2$, 298 K): δ/ppm = 41.2 ($[HCB_{11}H_5Br_6]^-$), 15.6 ($SiCH_2CH_3$), 7.7 ($SiCH_2CH_3$).

^{1}H,^{29}Si HMQC NMR (500/99 MHz, 1,2-$C_6D_4Cl_2$, 298 K, optimized for J = 7 Hz): δ/ppm = 1.71/64.3, 0.99/64.3.

A solution of $[Et_2ISi(HCB_{11}H_5Br_6)]$ (**8db**) in 1,2-$C_6D_4Cl_2$ was found to be stable for few hours at room temperature when protected from light, followed by complete conversion to $[Et_2ClSi(HCB_{11}H_5Br_6)]$ (**8bb**, δ(^{29}Si) = 73.6 ppm), as indicated by ^{29}Si NMR spectroscopy. Exposure of this solution to sunlight resulted in slow decomposition to Et_2SiCl_2, as indicated by a shift of the ^{29}Si NMR signal to δ(^{29}Si) = 36.3 ppm. The decomposition was accompanied by a color change from yellow to orange to dark red/brown and by decomposition of the carborate counteranion, as indicated by ^{11}B NMR spectroscopy.

4.2.15 [*i*$Pr_2ISi(HCB_{11}H_5Br_6)$] (**8dc**)

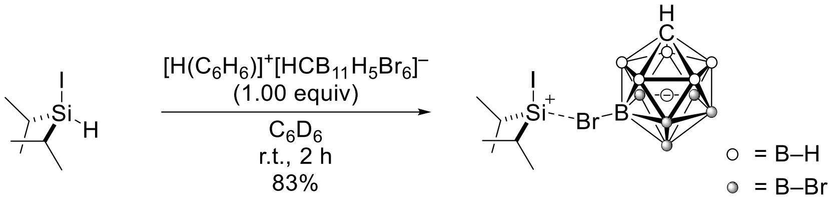

6dc (1.00 equiv)

8dc $C_7H_{20}B_{11}Br_6ISi$ M = 857.57 g/mol

Iodosilylium carborate **8dc** was prepared according to **GP 1** from diisopropyliodosilane (**6dc**, 7.75 µL, 10.2 mg, 42.1 µmol, 1.00 equiv) and $[H(C_6H_6)]^+[HCB_{11}H_5Br_6]^-$ (29.2 mg, 42.0 µmol, 1.00 equiv). [*i*$Pr_2ISi(HCB_{11}H_5Br_6)$] (**8dc**, 29.9 mg, 34.9 µmol, 83%) was obtained as an off-white solid.

^{1}H NMR (500 MHz, 1,2-$C_6D_4Cl_2$, 298 K): δ/ppm = 2.65 (s br, 1H, $[HCB_{11}H_5Br_6]^-$), 1.93 (sept, $^3J_{H,H}$ = 7.2 Hz, 2H, SiC*H*$(CH_3)_2$), 1.04 (d, $^3J_{H,H}$ = 7.2 Hz, 12H, SiCH(C*H*$_3)_2$). Deuterium incorporation into the carborate counteranion and the formation of [*i*$Pr_2ISi(HCB_{11}D_5Br_6)$] was observed in 1,2-$C_6D_4Cl_2$.[46]

^{11}B NMR (160 MHz, 1,2-$C_6D_4Cl_2$, 298 K): δ/ppm = –1.4 (s), –9.3 (s br), –20.5 (s br).

^{13}C{^{1}H} NMR (126 MHz, 1,2-$C_6D_4Cl_2$, 298 K): δ/ppm = 41.1 ([H*C*$B_{11}H_5Br_6]^-$), 21.7 (Si*C*H$(CH_3)_2$), 17.3 (s br, SiCH(*C*$H_3)_2$).

^{1}H,^{29}Si HMQC NMR (500/99 MHz, 1,2-$C_6D_4Cl_2$, 298 K, optimized for *J* = 7 Hz): δ/ppm = 1.93/78.8, 1.04/78.8.

Single crystals of [*i*$Pr_2ISi(HCB_{11}H_5Br_6)$] (**8dc**) suitable for X-ray diffraction analysis were obtained from a solution of silylium carborate **8dc** in 1,2-$C_6H_4F_2$ by vapor diffusion with *n*-pentane at –30 °C over 4 days (Supplementary Fig. 11 in Section 6.6). CCDC 2424594 contains the supplementary crystallographic data. These data are provided free of charge by The Cambridge Crystallographic Data Centre.

A solution of [*i*$Pr_2ISi(HCB_{11}H_5Br_6)$] (**8dc**) in 1,2-$C_6D_4Cl_2$ was found to decompose over the course of several hours to silylium ion *i*Pr_2ClSi^+, as indicated by a shift of the ^{29}Si NMR signal to δ(^{29}Si) = 74.6 ppm. Concommitant anion decomposition was observed in the ^{11}B NMR spectrum. Exposure to sunlight resulted in further decomposition to *i*Pr_2SiCl_2, as indicated by a shift of the ^{29}Si NMR signal to δ(^{29}Si) = 37.7 ppm. The decomposition was accompanied by a color change from yellow to orange to dark red/brown.

HRMS analysis (negative mode) of [*i*$Pr_2ISi(HCB_{11}H_5Br_6)$] (**8dc**) in 1,2-$C_6H_4Cl_2$ after 24 h revealed that $[HCB_{11}H_4IBr_6]^-$ (m/z = 742.5569) and $[HCB_{11}H_3I_2Br_6]^-$ (m/z = 868.4536) were the main decomposition products (Supplementary Fig. 5). Subsequent GLC-MS analysis revealed the presence of chlorobenzene. While the deuterated *ortho*-dichlorobenzene solvent serves as the chloride source, the iodosilylium ion apparently acts as an electrophilic iodinating agent (I^+ source), resulting in partial iodination of the B–H bonds of the *σ*-aromatic carborate anion by electrophilic aromatic substitution (S_EAr). The exact mechanism of this transformation remains unclear at this stage, but the existence of a silylene intermediate can be speculated.

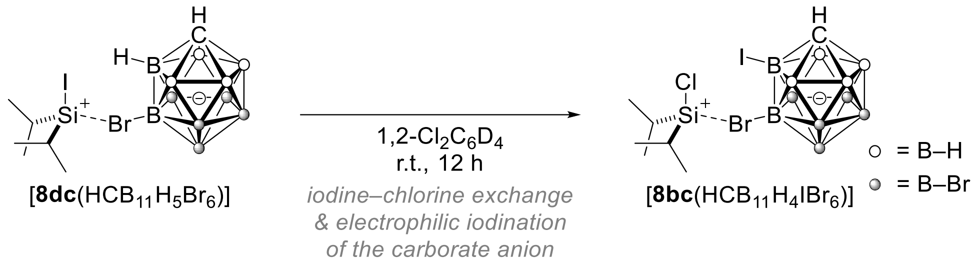

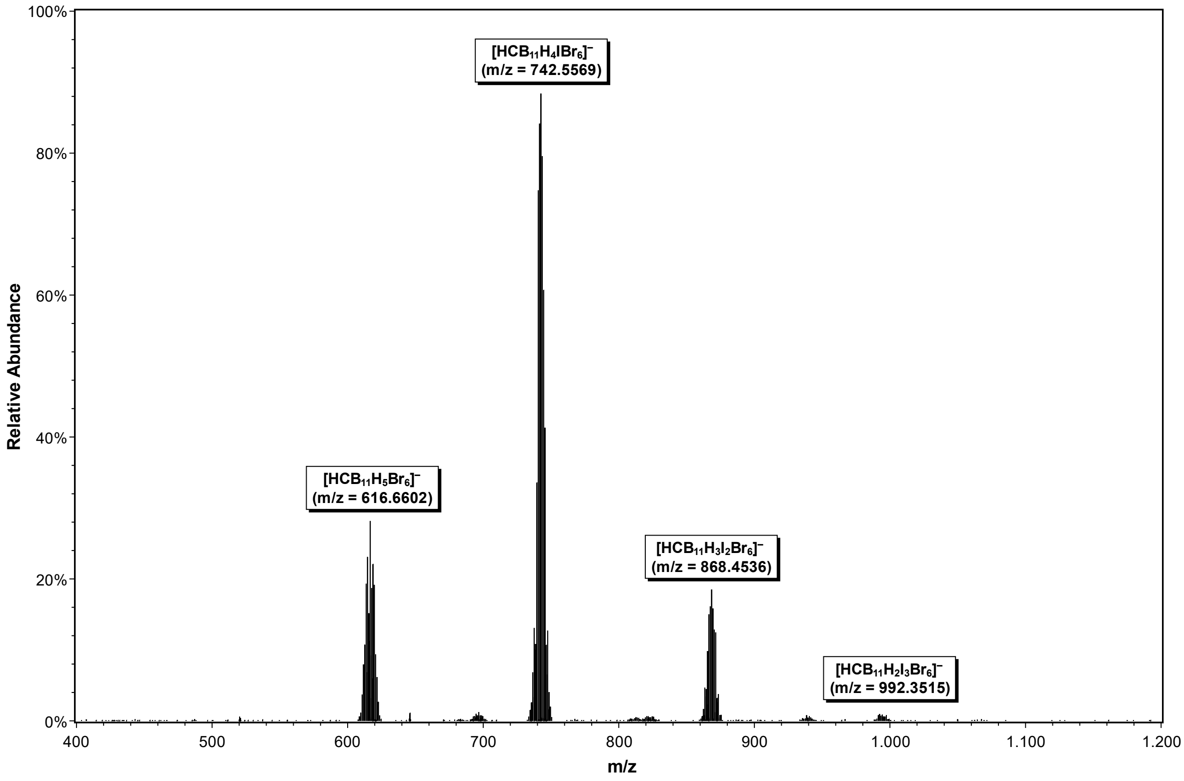

Supplementary Fig. 5. Decomposition of [*i*$Pr_2ISi(HCB_{11}H_5Br_6)$] (**8dc**) in 1,2-$C_6H_4Cl_2$ by iodine–chlorine exchange and electrophilic iodination of the carborate counteranion (top) and the high resolution mass spectrum (ESI, negative ion mode) of [*i*$Pr_2ISi(HCB_{11}H_5Br_6)$] (**8dc**) in 1,2-$C_6H_4Cl_2$ after 24 h (bottom).

4.2.16 [*t*$Bu_2ISi(HCB_{11}H_5Br_6)$] (**8dd**)

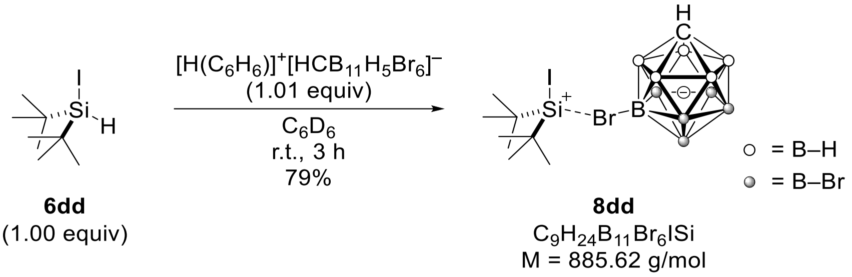

Iodosilylium carborate **8dd** was prepared according to **GP 1** from di-*tert*-butyliodosilane (**6dd**, 6.00 µL, 7.50 mg, 27.7 µmol, 1.00 equiv) and $[H(C_6H_6)]^+[HCB_{11}H_5Br_6]^-$ (19.5 mg, 28.0 µmol, 1.01 equiv). [*t*$Bu_2ISi(HCB_{11}H_5Br_6)$] (**8dd**, 19.3 mg, 21.8 µmol, 79%) was obtained as an off-white solid.

^{1}H NMR (500 MHz, 1,2-$C_6D_4Cl_2$, 298 K): δ/ppm = 2.68 (s br, 1H, $[HCB_{11}H_5Br_6]^-$), 1.09 (s, 18H, SiC(C*H*$_3$)$_3$). Deuterium incorporation into the carborate counteranion and the formation of [*t*$Bu_2ISi(HCB_{11}D_5Br_6)$] was observed in 1,2-$C_6D_4Cl_2$.[46]

^{11}B NMR (160 MHz, 1,2-$C_6D_4Cl_2$, 298 K): δ/ppm = –1.1 (s), –8.5 (s br), –19.9 (s br). Anion decomposition was found after several hours in solution, as indicated by the appearance of additional signals in the ^{11}B NMR spectrum.

^{13}C{^{1}H} NMR (126 MHz, 1,2-$C_6D_4Cl_2$, 298 K): δ/ppm = 40.8 ($[HCB_{11}H_5Br_6]^-$), 30.9 (Si*C*(CH$_3$)$_3$), 26.7 (SiC(*C*H$_3$)$_3$).

^{29}Si{^{1}H} DEPT NMR (99 MHz, 1,2-$C_6D_4Cl_2$, 298 K, optimized for $J_{H,Si}$ = 7 Hz, 13.6°): δ/ppm = 93.5.

A solution of [*t*$Bu_2ISi(HCB_{11}H_5Br_6)$] (**8dd**) in 1,2-$C_6D_4Cl_2$ was found to be stable for severals hours at room temperature when protected from light, followed by partial conversion to [*t*$Bu_2ClSi(HCB_{11}H_5Br_6)$] (**8bd**), as indicated by the appearance of an additional NMR signal at δ(^{29}Si) = 80.4 ppm. Exposure to sunlight resulted in slow decomposition to *t*Bu_2SiCl_2, as indicated by a shift of the ^{29}Si NMR signal to δ(^{29}Si) = 38.7 ppm, and further decomposition of the anion, as indicated by the appearance of several additional signals in the ^{11}B NMR spectrum. The decomposition was accompanied by a color change from yellow to orange to dark red/brown.

5 Determination of the Lewis acidity using Müller's FBN method

The Lewis acidity of the halogen-substituted silylium ions was experimentally verified by the NMR-based Lewis acidity scale introduced by Müller using *p*-fluorobenzonitrile (FBN) as an NMR probe.[38] Lewis adduct formation with the silylium ion leads to the generation of a silylnitrilium ion. The change of the ^{19}F NMR chemical shift (Δδ(^{19}F)) is used for scaling the different Lewis acidities of the corresponding silylium ions.

5.1 General procedure for the generation of silylnitrilium carborates with *p*-fluorobenzonitrile (**GP 2**)

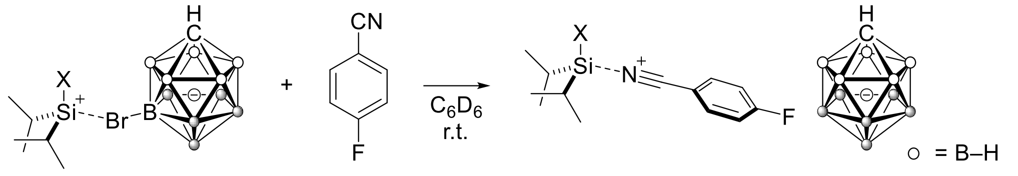

2c (X = H) **5c** (X = *i*Pr) **8ac–8dc** (X = F, Cl, Br, I) — **FBN** (0.50 equiv) — [**2c**(FBN)]$^+$[$HCB_{11}H_5Br_6$]$^-$ [**5c**(FBN)]$^+$[$HCB_{11}H_5Br_6$]$^-$ [**8ac–dc**(FBN)]$^+$[$HCB_{11}H_5Br_6$]$^-$

In a high-quality glovebox (O_2, H_2O < 1.0 ppm), the indicated silylium carborate (1.00 equiv) was suspended in C_6D_6 (0.7 mL) at room temperature and *p*-fluorobenzonitrile (**FBN**, 0.728 M in C_6D_6, 0.500 equiv) was added. The resulting solution was transferred into a J Young NMR tube and directly subjected to NMR spectroscopic analysis.

Supplementary Table 1. Selected NMR spectroscopic data of silylnitrilium carborates [iPr$_2$XSi(FBN)]$^+$ [$HCB_{11}H_5Br_6$]$^-$.

Entry	X	*i*Pr$_2$XSi$^+$	δ(^{19}F) [ppm][a]	Δδ(^{19}F) [ppm][b]	$^1J_{C,F}$ [Hz][a]	δ(^{29}Si) [ppm][c]
1	F	**8ac**	–84.5	19.4	271.1	11.2 (340.3 Hz)
2	Cl	**8bc**	–84.6	19.3	270.6	28.5
3	Br	**8cc**	–84.7	19.2	271.1	30.1
4	I	**8dc**	–85.0	18.9	271.4	25.1
5	H	**5c**	–86.4	17.5	268.8	18.2
6	*i*Pr	**2c**	–86.6	17.3	270.0	35.8

[a] Determined from the ^{19}F NMR spectrum (471 MHz, 298 K) in C_6D_6. [b] Deshielding of the ^{19}F NMR chemical shift relative to free and *p*-fluorobenzonitrile (FBN, δ(^{19}F) = –103.9 ppm). [c] Determined from the ^{29}Si{^{1}H} DEPT NMR spectrum (99 MHz, 298 K, optimized for $J_{H,Si}$ = 7 Hz, 15.5°) in C_6D_6. $^1J_{Si,F}$ coupling constant in Hz in parentheses.

5.1.1 [iPr$_2$FSi(FBN)]$^+$[HCB$_{11}$H$_5$Br$_6$]$^-$ ([**8ac**(FBN)]$^+$[HCB$_{11}$H$_5$Br$_6$]$^-$)

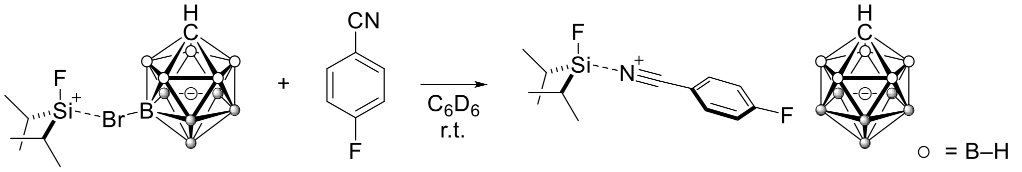

8ac (1.00 equiv) | **FBN** (0.510 equiv) | [**8ac**(FBN)]$^+$[HCB$_{11}$H$_5$Br$_6$]$^-$

Fluorosilylnitrilium carborate [**8ac**(FBN)]$^+$[HCB$_{11}$H$_5$Br$_6$]$^-$ was prepared according to **GP 2** from fluorosilylium carborate [iPr$_2$FSi(HCB$_{11}$H$_5$Br$_6$)] (**8ac**, 7.50 mg, 10.0 µmol, 1.00 equiv) and p-fluorobenzonitrile (**FBN**, 0.728 M in C$_6$D$_6$, 7.00 µL, 5.10 µmol, 0.510 equiv).

^{1}H NMR (500 MHz, C$_6$D$_6$, 298 K): δ/ppm = 7.68 (m$_c$, 2H, o-CH-Ar), 6.64 (t, $^3J_{H,H}$ = $^3J_{H,F}$ = 8.5 Hz, 2H, m-CH-Ar), 3.45–2.10 (m, 5H, [HCB$_{11}$$H_5Br_6$]$^-$), 1.86 (s br, 1H, [$HCB_{11}H_5Br_6$]$^-$), 1.54 (d sept, $^3J_{H,H}$ = $^3J_{H,F}$ = 7.6 Hz, 2H, SiCH(CH$_3$)$_2$), 0.98 (d, $^3J_{H,H}$ = 7.3 Hz, 6H, SiCH(CH_3)$_2$), 0.88 (d, $^3J_{H,H}$ = 7.5 Hz, 6H, SiCH(CH_3)$_2$).

^{11}B NMR (160 MHz, C$_6$D$_6$, 298 K): δ/ppm = –1.2 (s), –9.1 (s), –19.9 (s br).

^{13}C{^{1}H} NMR (126 MHz, C$_6$D$_6$, 298 K): δ/ppm = 169.8 (d, $^1J_{C,F}$ = 271.1 Hz, p-CF-Ar), 141.5 (d, $^3J_{C,F}$ = 11.7 Hz, o-CH-Ar), 126.2 (C≡N), 119.0 (d, $^2J_{C,F}$ = 23.5 Hz, m-CH-Ar), 95.4 (d, $^4J_{C,F}$ = 2.3 Hz, i-C-Ar), 41.6 ([HCB$_{11}$H$_5$Br$_6$]$^-$), 15.9 (SiCH(CH$_3$)$_2$), 15.3 (SiCH(CH$_3$)$_2$), 12.5 (d, $^2J_{C,F}$ = 10.6 Hz, SiCH(CH$_3$)$_2$).

^{19}F NMR (471 MHz, C$_6$D$_6$, 298 K): δ/ppm = –84.5 (m$_c$, 1F, p-CF-Ar), –161.7 (t, $^3J_{F,H}$ = 7.9 Hz, $^1J_{F,Si}$ = 340.3 Hz, 1F, Si–F).

^{29}Si{^{1}H} DEPT NMR (99 MHz, C$_6$D$_6$, 298 K, optimized for $J_{H,Si}$ = 7 Hz, 15.5°): δ/ppm = 11.3 (d, $^1J_{Si,F}$ = 340.3 Hz).

5.1.2 $[iPr_2ClSi(FBN)]^+[HCB_{11}H_5Br_6]^-$ ($[$**8bc**$(FBN)]^+[HCB_{11}H_5Br_6]^-$)

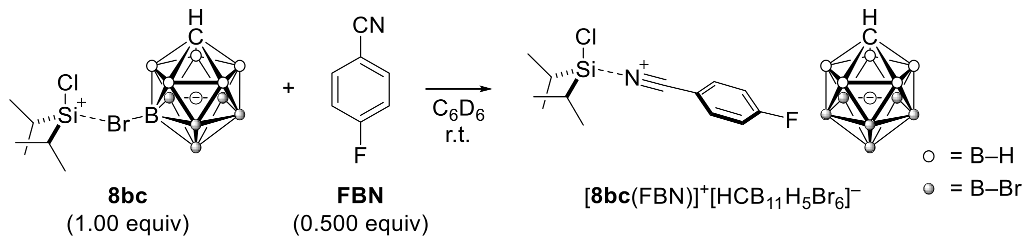

Chlorosilylnitrilium carborate $[$**8bc**$(FBN)]^+[HCB_{11}H_5Br_6]^-$ was prepared according to **GP 2** from chlorosilylium carborate $[iPr_2ClSi(HCB_{11}H_5Br_6)]$ (**8bc**, 7.80 mg, 10.2 µmol, 1.00 equiv) and *p*-fluorobenzonitrile (**FBN**, 0.728 M in C_6D_6, 7.00 µL, 5.10 µmol, 0.500 equiv).

^{1}H NMR (500 MHz, C_6D_6, 298 K): δ/ppm = 7.69 (m_c, 2H, *o*-C*H*-Ar), 6.64 (t, $^3J_{H,H}$ = $^3J_{H,F}$ = 8.4 Hz, 2H, *m*-C*H*-Ar), 3.45–2.10 (m, 5H, $[HCB_{11}H_5Br_6]^-$), 1.90 (s br, 1H, $[HCB_{11}H_5Br_6]^-$), 1.73 (sept, $^3J_{H,H}$ = 7.4 Hz, 2H, SiC*H*($CH_3)_2$), 1.01 (d, $^3J_{H,H}$ = 7.5 Hz, 6H, SiCH(C*H*$_3)_2$), 0.87 (d, $^3J_{H,H}$ = 7.6 Hz, 6H, SiCH(C*H*$_3)_2$).

^{11}B NMR (160 MHz, C_6D_6, 298 K): δ/ppm = –1.2 (s), –9.2 (s), –19.9 (s br).

^{13}C{^{1}H} NMR (176 MHz, C_6D_6, 298 K): δ/ppm = 169.7 (d, $^1J_{C,F}$ = 270.6 Hz, *p*-*C*F-Ar)), 141.4 (d, $^3J_{C,F}$ = 11.4 Hz, *o*-*C*H-Ar), 126.6 (*C*≡N), 119.1 (d, $^2J_{C,F}$ = 23.7 Hz, *m*-*C*H-Ar), 95.6 (d, $^4J_{C,F}$ = 3.3 Hz, *i*-*C*-Ar), 41.7 ($[HCB_{11}H_5Br_6]^-$), 16.6 (SiCH(*C*H$_3)_2$), 15.6 (SiCH(*C*H$_3)_2$), 15.2 (Si*C*H($CH_3)_2$).

^{19}F NMR (471 MHz, C_6D_6, 298 K): δ/ppm = –84.6 (m_c, *p*-C*F*-Ar).

^{29}Si{^{1}H} DEPT NMR (99 MHz, C_6D_6, 298 K, optimized for $J_{H,Si}$ = 7 Hz, 15.5°): δ/ppm = 28.5.

5.1.3 [iPr$_2$BrSi(FBN)]$^+$[HCB$_{11}$H$_5$Br$_6$]$^-$ ([**8cc**(FBN)]$^+$[HCB$_{11}$H$_5$Br$_6$]$^-$)

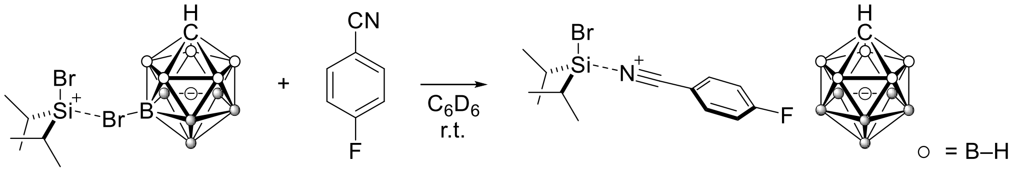

8cc (1.00 equiv) **FBN** (0.501 equiv) [**8cc**(FBN)]$^+$[HCB$_{11}$H$_5$Br$_6$]$^-$

Bromosilylnitrilium carborate [**8cc**(FBN)]$^+$[HCB$_{11}$H$_5$Br$_6$]$^-$ was prepared according to **GP 2** from bromosilylium carborate [iPr$_2$BrSi(HCB$_{11}$H$_5$Br$_6$)] (**8cc**, 5.00 mg, 6.17 µmol, 1.00 equiv) and *p*-fluorobenzonitrile (**FBN**, 0.728 M in C$_6$D$_6$, 4.25 µL, 3.09 µmol, 0.501 equiv).

^{1}H NMR (500 MHz, C$_6$D$_6$, 298 K): δ/ppm = 7.59 (m$_c$, 2H, *o*-C*H*-Ar), 6.57 (t, $^3J_{H,H}$ = $^3J_{H,F}$ = 8.4 Hz, 2H, *m*-C*H*-Ar), 3.45–2.10 (m, 5H, [HCB$_{11}$*H*$_5$Br$_6$]$^-$), 1.87 (s br, 1H, [*H*CB$_{11}$H$_5$Br$_6$]$^-$), 1.85 (sept, $^3J_{H,H}$ = 7.3 Hz, 2H, SiC*H*(CH$_3$)$_2$), 0.98 (d, $^3J_{H,H}$ = 7.3 Hz, 6H, SiCH(C*H*$_3$)$_2$), 0.82 (d, $^3J_{H,H}$ = 7.5 Hz, 6H, SiCH(C*H*$_3$)$_2$).

^{11}B NMR (160 MHz, C$_6$D$_6$, 298 K): δ/ppm = –1.1 (s), –9.2 (s), –19.9 (s br).

^{13}C{^{1}H} NMR (126 MHz, C$_6$D$_6$, 298 K): δ/ppm = 169.7 (d, $^1J_{C,F}$ = 271.1 Hz, *p*-*C*F-Ar), 141.2 (d, $^3J_{C,F}$ = 11.7 Hz, *o*-*C*H-Ar), 126.9 (*C*≡N), 119.0 (d, $^2J_{C,F}$ = 23.5 Hz, *m*-*C*H-Ar), 95.7 (d, $^4J_{C,F}$ = 3.5 Hz, *i*-*C*-Ar), 41.7 ([H*C*B$_{11}$H$_5$Br$_6$]$^-$), 17.1 (SiCH(*C*H$_3$)$_2$), 15.8 (Si*C*H(CH$_3$)$_2$), 15.8 (SiCH(*C*H$_3$)$_2$).

^{19}F NMR (471 MHz, C$_6$D$_6$, 298 K): δ/ppm = –84.7 (m$_c$, *p*-C*F*-Ar).

^{29}Si{^{1}H} DEPT NMR (99 MHz, C$_6$D$_6$, 298 K, optimized for $J_{H,Si}$ = 7 Hz, 15.5°): δ/ppm = 30.1.

5.1.4 [iPr$_2$ISi(FBN)]$^+$[HCB$_{11}$H$_5$Br$_6$]$^-$ ([**8dc**(FBN)]$^+$[HCB$_{11}$H$_5$Br$_6$]$^-$)

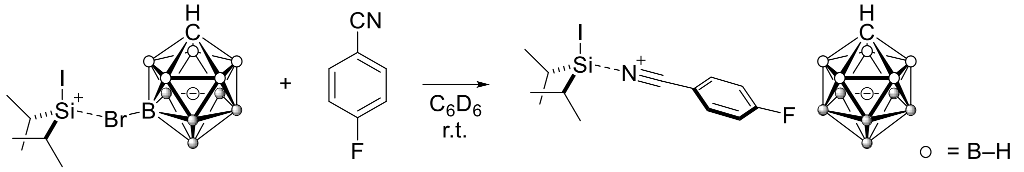

8dc (1.00 equiv) **FBN** (0.520 equiv) [**8dc**(FBN)]$^+$[HCB$_{11}$H$_5$Br$_6$]$^-$

Iodosilylnitrilium carborate [**8dc**(FBN)]$^+$[HCB$_{11}$H$_5$Br$_6$]$^-$ was prepared according to **GP 2** from iodosilylium carborate [iPr$_2$ISi(HCB$_{11}$H$_5$Br$_6$)] (**8dc**, 4.20 mg, 4.90 µmol, 1.00 equiv) and p-fluorobenzonitrile (**FBN**, 0.728 M in C_6D_6, 3.50 µL, 2.55 µmol, 0.520 equiv).

^{1}H NMR (500 MHz, C_6D_6, 298 K): δ/ppm = 7.55 (m$_c$, 2H, o-CH-Ar), 6.55 (t, $^3J_{H,H}$ = $^3J_{H,F}$ = 8.0 Hz, 2H, m-CH-Ar), 3.45–2.10 (m, 5H, [HCB$_{11}$$H_5Br_6$]$^-$), 1.94–1.81 (m, 3H, SiC$H$(CH$_3$)$_2$, [$HCB_{11}H_5Br_6$]$^-$), 0.94 (d, $^3J_{H,H}$ = 7.0 Hz, 6H, SiCH(CH_3)$_2$), 0.77 (d, $^3J_{H,H}$ = 7.5 Hz, 6H, SiCH(CH_3)$_2$).

^{11}B NMR (160 MHz, C_6D_6, 298 K): δ/ppm = –1.1 (s), –9.2 (s), –20.1 (s br).

^{13}C{^{1}H} NMR (176 MHz, C_6D_6, 298 K): δ/ppm = 169.6 (d, $^1J_{C,F}$ = 271.4 Hz, p-CF-Ar), 141.0 (d, $^3J_{C,F}$ = 11.4 Hz, o-CH-Ar), 127.3 (C≡N), 118.9 (d, $^2J_{C,F}$ = 23.7 Hz, m-CH-Ar), 95.9 (d, $^4J_{C,F}$ = 2.5 Hz, i-C-Ar), 41.7 ([HCB$_{11}$H$_5$Br$_6$]$^-$), 18.0 (SiCH(CH$_3$)$_2$), 16.5 (SiCH(CH$_3$)$_2$), 16.4 (SiCH(CH$_3$)$_2$).

^{19}F NMR (471 MHz, C_6D_6, 298 K): δ/ppm = –85.0 (m$_c$, 1F, p-CF-Ar).

^{1}H,^{29}Si HMQC NMR (500/99 MHz, C_6D_6, 298 K, optimized for J = 7 Hz): δ/ppm = 0.94/25.1, 0.77/25.1.

5.1.5 [*i*Pr_2HSi(FBN)]$^+$[$HCB_{11}H_5Br_6$]$^-$ ([**5c**(FBN)]$^+$[$HCB_{11}H_5Br_6$]$^-$)

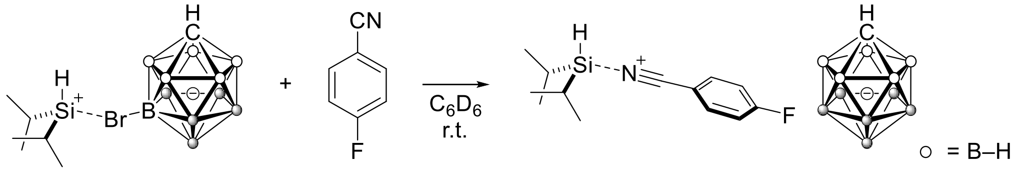

5c (1.00 equiv) **FBN** (0.507 equiv) [**5c**(FBN)]$^+$[$HCB_{11}H_5Br_6$]$^-$

Hydrosilylnitrilium carborate [**5c**(FBN)]$^+$[$HCB_{11}H_5Br_6$]$^-$ was prepared according to **GP 2** from hydrosilylium carborate [*i*Pr_2HSi($HCB_{11}H_5Br_6$)] (**5c**, 5.00 mg, 6.83 µmol, 1.00 equiv) and *p*-fluorobenzonitrile (**FBN**, 0.728 M in C_6D_6, 4.75 µL, 3.46 µmol, 0.507 equiv).

^{1}H NMR (500 MHz, C_6D_6, 298 K): δ/ppm = 7.79 (m$_c$, 2H, *o*-C*H*-Ar), 6.65 (t, $^3J_{H,H}$ = $^3J_{H,F}$ = 8.5 Hz, 2H, *m*-C*H*-Ar), 4.40 (t, $^3J_{H,H}$ = 3.0 Hz, $^1J_{H,Si}$ = 234.7 Hz, 1H, Si*H*), 3.45–2.10 (m, 5H, [HCB_{11}*H*$_5Br_6$]$^-$), 1.86 (s br, 1H, [*H*$CB_{11}H_5Br_6$]$^-$), 1.30 (sept d, $^3J_{H,H}$ = 7.5 Hz, $^3J_{H,H}$ = 3.0 Hz, 2H, SiC*H*(CH_3)$_2$), 0.89 (d, $^3J_{H,H}$ = 7.4 Hz, 6H, SiCH(C*H*$_3$)$_2$), 0.81 (d, $^3J_{H,H}$ = 7.4 Hz, 6H, SiCH(C*H*$_3$)$_2$).

^{11}B NMR (160 MHz, C_6D_6, 298 K): δ/ppm = –1.2 (s), –9.2 (s), –19.8 (d br, $^1J_{B,H}$ = 154.8 Hz).

^{13}C{^{1}H} NMR (126 MHz, C_6D_6, 298 K): δ/ppm = 169.3 (d, $^1J_{C,F}$ = 268.8 Hz, *p*-*C*F-Ar), 140.9 (d, $^3J_{C,F}$ = 11.7 Hz, *o*-*C*H-Ar), 126.1 (*C*≡N), 118.8 (d, $^2J_{C,F}$ = 23.5 Hz, *m*-*C*H-Ar), 96.8 (d, $^4J_{C,F}$ = 3.5 Hz, *i*-*C*-Ar), 42.2 ([H*C*$B_{11}H_5Br_6$]$^-$), 17.3 (SiCH(*C*H_3)$_2$), 16.8 (SiCH(*C*H_3)$_2$), 11.1 (Si*C*H(CH_3)$_2$).

^{19}F NMR (471 MHz, C_6D_6, 298 K): δ/ppm = –86.4 (m$_c$, 1F, *p*-C*F*-Ar).

^{29}Si{^{1}H} DEPT NMR (99 MHz, C_6D_6, 298 K, optimized for $J_{H,Si}$ = 7 Hz, 15.5°): δ/ppm = 18.2.

5.1.6 [iPr$_3$Si(FBN)]$^+$[HCB$_{11}$H$_5$Br$_6$]$^-$ ([**2c**(FBN)]$^+$[HCB$_{11}$H$_5$Br$_6$]$^-$)

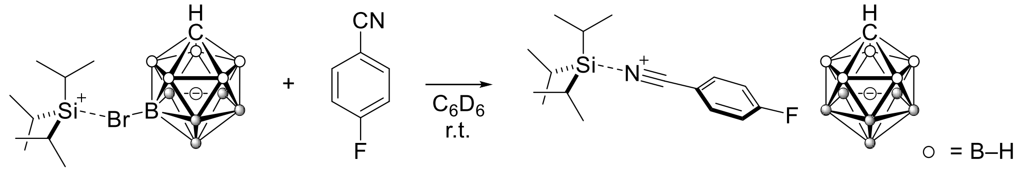

2c (1.00 equiv) **FBN** (0.516 equiv) [**2c**(FBN)]$^+$[HCB$_{11}$H$_5$Br$_6$]$^-$

Silylnitrilium carborate [**2c**(FBN)]$^+$[HCB$_{11}$H$_5$Br$_6$]$^-$ was prepared according to **GP 2** from silylium carborate [iPr$_3$Si(HCB$_{11}$H$_5$Br$_6$)] (**2c**, 6.00 mg, 7.75 µmol, 1.00 equiv) and *p*-fluorobenzonitrile (**FBN**, 0.728 M in C_6D_6, 5.50 µL, 4.00 µmol, 0.516 equiv).

^{1}H NMR (500 MHz, C_6D_6, 298 K): δ/ppm = 7.81 (m$_c$, 2H, *o*-C*H*-Ar), 6.81 (t, $^3J_{H,H}$ = $^3J_{H,F}$ = 8.4 Hz, 2H, *m*-C*H*-Ar), 3.45–2.10 (m, 5H, [HCB$_{11}$*H*$_5$Br$_6$]$^-$), 1.90 (s br, 1H, [*H*CB$_{11}$H$_5$Br$_6$]$^-$), 1.26 (sept, $^3J_{H,H}$ = 7.5 Hz, 3H, SiC*H*(CH$_3$)$_2$), 0.89 (d, $^3J_{H,H}$ = 7.6 Hz, 18H, SiCH(C*H*$_3$)$_2$).

^{11}B NMR (160 MHz, C_6D_6, 298 K): δ/ppm = –1.2 (s), –9.1 (s), –19.4 (s br).

^{13}C{^{1}H} NMR (126 MHz, C_6D_6, 298 K): δ/ppm = 169.3 (d, $^1J_{C,F}$ = 270.0 Hz, *p*-*C*F-Ar), 140.6 (d, $^3J_{C,F}$ = 11.7 Hz, *o*-*C*H-Ar), 125.6 (*C*≡N), 119.2 (d, $^2J_{C,F}$ = 23.5 Hz, *m*-*C*H-Ar), 96.7 (d, $^4J_{C,F}$ = 2.3 Hz, *i*-*C*-Ar), 41.7 ([H*C*B$_{11}$H$_5$Br$_6$]$^-$), 17.3 (SiCH(*C*H$_3$)$_2$), 16.8 (SiCH(*C*H$_3$)$_2$), 11.1 (Si*C*H(CH$_3$)$_2$).

^{19}F NMR (471 MHz, C_6D_6, 298 K): δ/ppm = –86.6 (m$_c$, 1F, *p*-C*F*-Ar).

^{29}Si{^{1}H} DEPT NMR (99 MHz, C_6D_6, 298 K, optimized for $J_{H,Si}$ = 7 Hz, 12.6°): δ/ppm = 35.8.

6 Crystallographic data

6.1 Molecular structure of [*i*$Pr_2HSi(HCB_{11}H_5Br_6)$] (**5c**) [CCDC 2424587]

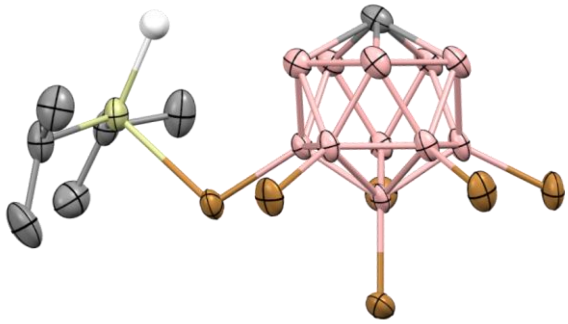

Supplementary Fig. 6. ORTEP view of the molecular structure of [*i*$Pr_2HSi(HCB_{11}H_5Br_6)$] (**5c**) with thermal ellipsoids shown at the 50% probability level. Hydrogen atoms except for Si–H are omitted for clarity. The hydrogen atom at the silicon atom was observable in the electron density map.

Supplementary Table 2. Crystal data and structure refinement for [*i*$Pr_2HSi(HCB_{11}H_5Br_6)$] (**5c**)

Empirical formula	$C_7H_{21}B_{11}Br_6Si$	
Formula weight	731.70	
Temperature	150.01(10) K	
Wavelength	1.54184 Å	
Crystal system	Triclinic	
Space group	P-1 (No. 2)	
Unit cell dimensions	a = 7.9052(5) Å	α = 86.319(4)°
	b = 10.4516(6) Å	β = 81.563(5)°
	c = 14.1493(8) Å	γ = 84.577(5)°
Volume	1149.71(12) $Å^3$	
Z	2	
Density (calculated)	2.114 Mg/m^3	
Absorption coefficient	13.024 mm^{-1}	
F(000) 684		
Crystal size	0.278 × 0.190 × 0.052 mm^3	
Theta range for data collection	3.162 to 72.246°	
Index ranges	–9<=h<=9, –11<=k<=12, –17<=l<=16	
Reflections collected	7420	
Independent reflections	4400 [R(int) = 0.0218]	
Completeness to theta = 67.684°	99.7%	
Absorption correction	Semi-empirical from equivalents	
Max. and min. transmission	1.00000 and 0.18275	
Refinement method	Full-matrix least-squares on F^2	
Data / restraints / parameters	4400 / 0 / 258	
Goodness-of-fit on F^2	1.072	
Final R indices [I>2sigma(I)]	R1 = 0.0361, wR2 = 0.0969	
R indices (all data)	R1 = 0.0380, wR2 = 0.0984	
Extinction coefficient	n/a	
Largest diff. peak and hole	1.198 and –1.096 e.$Å^{-3}$	

6.2 Molecular structure of [iPr$_2$FSi(HCB$_{11}$H$_5$Br$_6$)] (**8ac**) [CCDC 2424589]

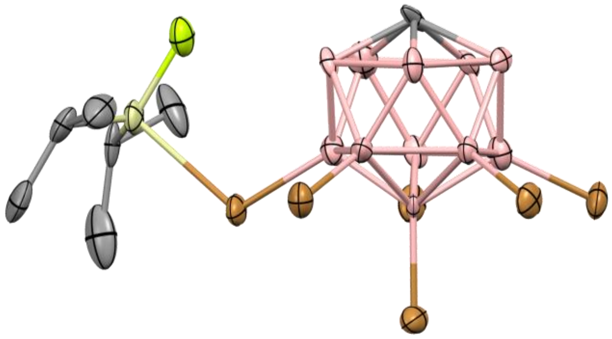

Supplementary Fig. 7. ORTEP view of the molecular structure of [iPr$_2$FSi(HCB$_{11}$H$_5$Br$_6$)] (**8ac**) with thermal ellipsoids shown at the 50% probability level. All hydrogen atoms are omitted for clarity. Note: Due to rapid decomposition during the measurement and the resulting low quality of the crystal, the checkCIF file containes A- and B-level alerts regarding the calculated residual electron density at the bromine atoms of the carborate cluster and the C–C bond precision, which is 0.04 Å. The anisotropic displacement parameters of the boron atoms were restrained.

Supplementary Table 3. Crystal data and structure refinement for [iPr$_2$FSi(HCB$_{11}$H$_5$Br$_6$)] (**8ac**)

Empirical formula	$C_7H_{20}B_{11}Br_6FSi$	
Formula weight	749.69	
Temperature	150.01(10) K	
Wavelength	1.54184 Å	
Crystal system	Triclinic	
Space group	P-1 (No. 2)	
Unit cell dimensions	a = 7.9237(11) Å	α = 86.500(11)°
	b = 10.6003(13) Å	β = 81.660(12)°
	c = 14.022(2) Å	γ = 83.617(11)°
Volume	1156.8(3) Å^3	
Z	2	
Density (calculated)	2.152 Mg/m^3	
Absorption coefficient	13.030 mm^{-1}	
F(000)	700	
Crystal size	0.178 × 0.154 × 0.094 mm^3	
Theta range for data collection	3.189 to 72.553°	
Index ranges	–9<=h<=9, –13<=k<=12, –17<=l<=17	
Reflections collected	7928	
Independent reflections	4444 [R(int) = 0.1501]	
Completeness to theta = 67.684°	99.8%	
Absorption correction	Semi-empirical from equivalents	
Max. and min. transmission	1.00000 and 0.07731	
Refinement method	Full-matrix least-squares on F^2	
Data / restraints / parameters	4444 / 66 / 239	
Goodness-of-fit on F^2	0.975	
Final R indices [I>2sigma(I)]	R1 = 0.1024, wR2 = 0.2400	
R indices (all data)	R1 = 0.1854, wR2 = 0.3335	
Extinction coefficient	n/a	
Largest diff. peak and hole	2.272 and –2.260 e.Å^{-3}	

6.3 Molecular structure of [tBu$_2$FSi(HCB$_{11}$H$_5$Br$_6$)] (**8ad**) [CCDC 2424600]

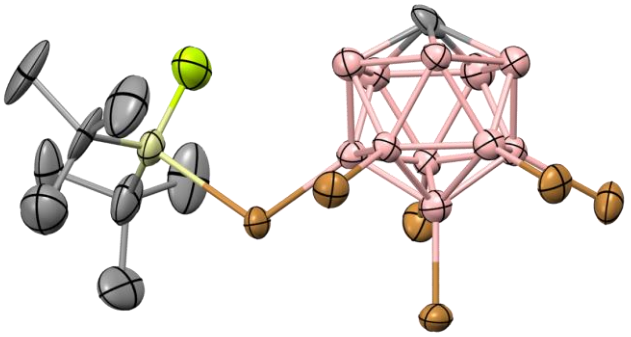

Supplementary Fig. 8. ORTEP view of the molecular structure of [tBu$_2$FSi(HCB$_{11}$H$_5$Br$_6$)] (**8ad**) with thermal ellipsoids shown at the 50% probability level. All hydrogen atoms are omitted for clarity. Note: Due to rapid decomposition during the measurement and the resulting low quality of the crystal, the checkCIF file containes A- and B-level alerts regarding the calculated residual electron density at one boron and carbon atom of the carborate cluster and the C–C bond precision, which is 0.04333 Å.

Supplementary Table 4. Crystal data and structure refinement for [tBu$_2$FSi(HCB$_{11}$H$_5$Br$_6$)] (**8ad**)

Empirical formula	$C_9H_{24}B_{11}Br_6FSi$	
Formula weight	777.74	
Temperature	150.01(10) K	
Wavelength	1.54184 Å	
Crystal system	Triclinic	
Space group	P-1 (No. 2)	
Unit cell dimensions	a = 8.0266(6) Å	α = 84.541(6)°
	b = 12.0074(9) Å	β = 80.991(6)°
	c = 13.8919(8) Å	γ = 77.860(7)°
Volume	1290.11(16) Å^3	
Z	2	
Density (calculated)	2.002 Mg/m^3	
Absorption coefficient	11.712 mm^{-1}	
F(000)	732	
Crystal size	0.212 × 0.084 × 0.030 mm^3	
Theta range for data collection	3.228 to 72.485°	
Index ranges	–9<=h<=9, –14<=k<=14, –14<=l<=17	
Reflections collected	9561	
Independent reflections	4978 [R(int) = 0.0906]	
Completeness to theta = 67.684°	99.8%	
Absorption correction	Semi-empirical from equivalents	
Max. and min. transmission	1.00000 and 0.05765	
Refinement method	Full-matrix least-squares on F^2	
Data / restraints / parameters	4978 / 67 / 259	
Goodness-of-fit on F^2	1.103	
Final R indices [I>2sigma(I)]	R1 = 0.1047, wR2 = 0.2829	
R indices (all data)	R1 = 0.1410, wR2 = 0.3047	
Extinction coefficient	n/a	
Largest diff. peak and hole	2.301 and –1.440 e.Å^{-3}	

6.4 Molecular structure of [iPr$_2$ClSi(HCB$_{11}$H$_5$Br$_6$)] (**8bc**) [CCDC 2424591]

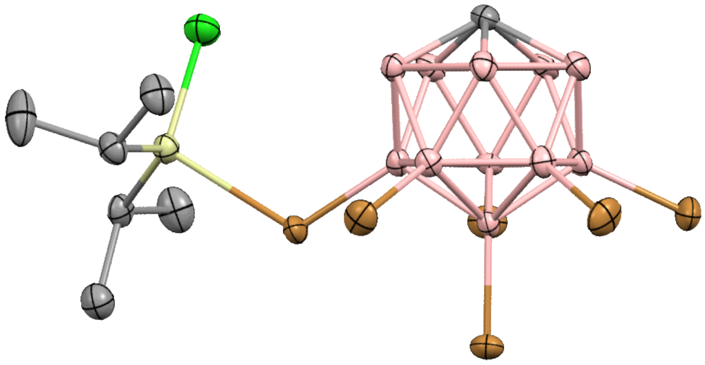

Supplementary Fig. 9. ORTEP view of the molecular structure of [iPr$_2$ClSi(HCB$_{11}$H$_5$Br$_6$)] (**8bc**) with thermal ellipsoids shown at the 50% probability level. All hydrogen atoms are omitted for clarity.

Supplementary Table 5. Crystal data and structure refinement for [iPr$_2$ClSi(HCB$_{11}$H$_5$Br$_6$)] (**8bc**)

Empirical formula	$C_7H_{20}B_{11}Br_6ClSi$	
Formula weight	766.14	
Temperature	150.01(10) K	
Wavelength	1.54184 Å	
Crystal system	Monoclinic	
Space group	$P2_1/c$ (No. 14)	
Unit cell dimensions	a = 7.65400(10) Å	α = 90°
	b = 16.3925(2) Å	β = 91.3720(10)°
	c = 37.9111(4) Å	γ = 90°
Volume	4755.27(10) Å^3	
Z	8	
Density (calculated)	2.140 Mg/m^3	
Absorption coefficient	13.645 mm^{-1}	
F(000)	2864	
Crystal size	0.280 × 0.168 × 0.076 mm^3	
Theta range for data collection	2.937 to 72.481°	
Index ranges	–9<=h<=7, –20<=k<=19, –46<=l<=46	
Reflections collected	19693	
Independent reflections	9200 [R(int) = 0.0339]	
Completeness to theta = 67.684°	99.7%	
Absorption correction	Semi-empirical from equivalents	
Max. and min. transmission	1.00000 and 0.13961	
Refinement method	Full-matrix least-squares on F^2	
Data / restraints / parameters	9200 / 0 / 524	
Goodness-of-fit on F^2	1.081	
Final R indices [I>2sigma(I)]	R1 = 0.0385, wR2 = 0.0972	
R indices (all data)	R1 = 0.0420, wR2 = 0.0996	
Extinction coefficient	n/a	
Largest diff. peak and hole	0.735 and –0.866 e.Å^{-3}	

6.5 Molecular structure of [iPr$_2$BrSi(HCB$_{11}$H$_5$Br$_6$)] (**8cc**) [CCDC 2424593]

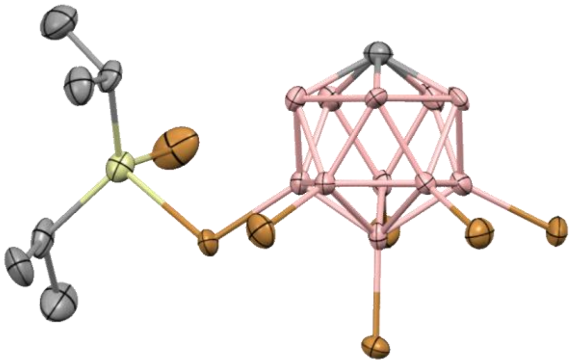

Supplementary Fig. 10. ORTEP view of the molecular structure of [iPr$_2$BrSi(HCB$_{11}$H$_5$Br$_6$)] (**8cc**) with thermal ellipsoids shown at the 50% probability level. All hydrogen atoms are omitted for clarity.

Supplementary Table 6. Crystal data and structure refinement for [iPr$_2$BrSi(HCB$_{11}$H$_5$Br$_6$)] (**8cc**)

Empirical formula	$C_7H_{20}B_{11}Br_7Si$	
Formula weight	810.60	
Temperature	150.01(10) K	
Wavelength	1.54184 Å	
Crystal system	Monoclinic	
Space group	C2/c (No. 15)	
Unit cell dimensions	a = 22.8103(6) Å	α = 90°
	b = 7.7152(2) Å	β = 103.689(3)°
	c = 28.3445(8) Å	γ = 90°
Volume	4846.5(2) Å^3	
Z	8	
Density (calculated)	2.222 Mg/m^3	
Absorption coefficient	14.306 mm^{-1}	
F(000)	3008	
Crystal size	0.180 × 0.072 × 0.042 mm^3	
Theta range for data collection	3.209 to 72.609°	
Index ranges	–25<=h<=27, –9<=k<=9, –34<=l<=35	
Reflections collected	16913	
Independent reflections	4744 [R(int) = 0.0980]	
Completeness to theta = 67.684°	99.9 %	
Absorption correction	Semi-empirical from equivalents	
Max. and min. transmission	1.00000 and 0.36664	
Refinement method	Full-matrix least-squares on F^2	
Data / restraints / parameters	4744 / 1 / 254	
Goodness-of-fit on F^2	1.054	
Final R indices [I>2sigma(I)]	R1 = 0.0609, wR2 = 0.1547	
R indices (all data)	R1 = 0.0774, wR2 = 0.1729	
Extinction coefficient	n/a	
Largest diff. peak and hole	1.193 and –1.949 e.Å^{-3}	

6.6 Molecular structure of [iPr$_2$ISi(HCB$_{11}$H$_5$Br$_6$)] (**8dc**) [CCDC 2424594]

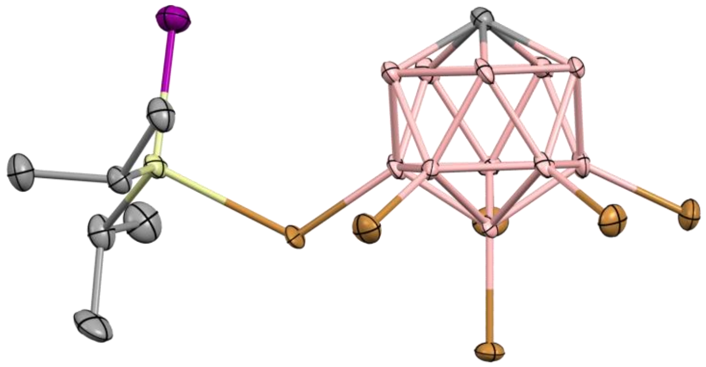

Supplementary Fig. 11. ORTEP view of the molecular structure of [iPr$_2$ISi(HCB$_{11}$H$_5$Br$_6$)] (**8dc**) with thermal ellipsoids shown at the 50% probability level. All hydrogen atoms are omitted for clarity.

Supplementary Table 7. Crystal data and structure refinement for [iPr$_2$ISi(HCB$_{11}$H$_5$Br$_6$)] (**8dc**)

Empirical formula	$C_7H_{20}B_{11}Br_6ISi$	
Formula weight	857.59	
Temperature	150.01(10) K	
Wavelength	1.54184 Å	
Crystal system	Triclinic	
Space group	P-1 (No. 2)	
Unit cell dimensions	a = 7.6822(3) Å	α = 75.732(4)°
	b = 12.0386(6) Å	β = 80.088(4)°
	c = 14.1744(7) Å	γ = 73.298(4)°
Volume	1209.59(10) Å^3	
Z	2	
Density (calculated)	2.355 Mg/m^3	
Absorption coefficient	22.416 mm^{-1}	
F(000)	788	
Crystal size	0.140 × 0.066 × 0.022 mm^3	
Theta range for data collection	3.236 to 72.642°	
Index ranges	–9<=h<=9, –11<=k<=14, –17<=l<=16	
Reflections collected	8437	
Independent reflections	4662 [R(int) = 0.0456]	
Completeness to theta = 67.684°	99.9 %	
Absorption correction	Semi-empirical from equivalents	
Max. and min. transmission	1.00000 and 0.00594	
Refinement method	Full-matrix least-squares on F^2	
Data / restraints / parameters	4662 / 0 / 242	
Goodness-of-fit on F^2	1.024	
Final R indices [I>2sigma(I)]	R1 = 0.0460, wR2 = 0.1181	
R indices (all data)	R1 = 0.0539, wR2 = 0.1247	
Extinction coefficient	n/a	
Largest diff. peak and hole	1.300 and –2.073 e.Å^{-3}	

7 Computational details

After conformational sampling with CREST 2.12[47] using the GFN2-xTB tight-binding method,[48] local structure optimizations were performed with a local version of Turbomole, based on release 7.9[49] at the DFT BP86[50,51,52] level together with a def2-TZVP[53] basis, D4 dispersion corrections[54] and convergence criteria of 10^{-7} Hartree for the energy and 10^{-4} for the Cartesian gradient norm. Harmonic vibrational frequencies were calculated to ensure that the structures converged into local minima and to obtain thermodynamic contributions for the calculation of FIAs. For all DFT single-point energy calculations, the energy convergence criterion was set to 10^{-9} Hartree ($scfconv 9), the density convergence to 10^{-7} ($denconv 1d-7). Single-point energy calculations of FIAs of the Lewis acids were performed with the local hybrid functional LH20t,[55] def2-TZVPD[53,56] basis sets, and D4 dispersion corrections.[54] All calculations employed a fine integration grid (gridsize 5) and the multipole-accelerated RI-J approximation[57] together with the corresponding auxiliary basis sets.[58]

Nuclear shieldings and the NMR chemical shifts have been calculated using x2c-TZVPall-2c[59] basis sets on all atoms with the DFT functional PBE0.[60] One-component (1c, $rx2c) as well as two-component (2c, $soghf) calculations in a Kramers-restricted formalism ($kramers) were carried out using Turbomole's mpshift module. Boettger's "screened nuclear spin-orbit" (SNSO) approach[61,62,63,64] ($snso) and a Gaussian finite nucleus model[64,65] ($finnuc) were applied.

Additional four-component matrix Dirac-Kohn-Sham shielding calculations were performed with the program ReSpect, release 5.3.0.[66] In this case basis sets suited for four-component calculations had to be used for the heavy atoms. upcS-3[67,68,69] basis sets were used for Si and its directly neighboring C atoms, dyall-vtz[70] basis sets were used for the halogen atoms directly bound to silicon. For the remaining atoms, upcS-2[67,68,71] basis sets (dyall-vdz[70] for Br) were employed to reduce the computational burden of the 4c calculations. Again, a Gaussian finite nucleus model was applied.

NBO program version 7.0[72] interfaced to Turbomole was used for investigating the electronic structure of the silylium ion pairs, at the LH20t/def2-TZVPD level.

The silylium carborate adducts can exhibit different binding modes in which the silyl cation can be coordinated by one bromine atom either from the pentagonal belt of the carborate cluster (7-position) or from the antipodal 12-position.[28,32] However, the energy differences are rather small, and it depends on the steric influence of the alkyl substituent at the silicon atom which isomer is energetically preferred.[28] It is assumed that crystal-packing effects are the reason why all silylium carborates only crystallize in form of their 7-isomer. During our calculations of the minimum-energy structures, we also noticed that the silyl moiety in the 7-isomer can adopt different conformations, in particular a conformer **A** with an additional coordination to the antipodal bromine atom of the carborate anion and a conformer **B** where this interaction is not present (Supplementary Fig. 12). The latter conformation resembles that of the molecular structures in the solid-state (see Section Crystallographic characterization in the main manuscript). Our computations energetically favor conformer **A** by about 10 kJ mol^{-1} for all alkyl substituents except for Alk = *t*Bu, where conformer **B** is preferred (Supplementary Table 8). The best computed shifts at the 2c level for conformer **B** are slightly below the experimental values and those for conformer **A** are somewhat above (Supplementary Fig. 13). We assume that fast conformational rotation will lead to an average value, providing even better agreement with the experimentally determined chemical shifts in solution.

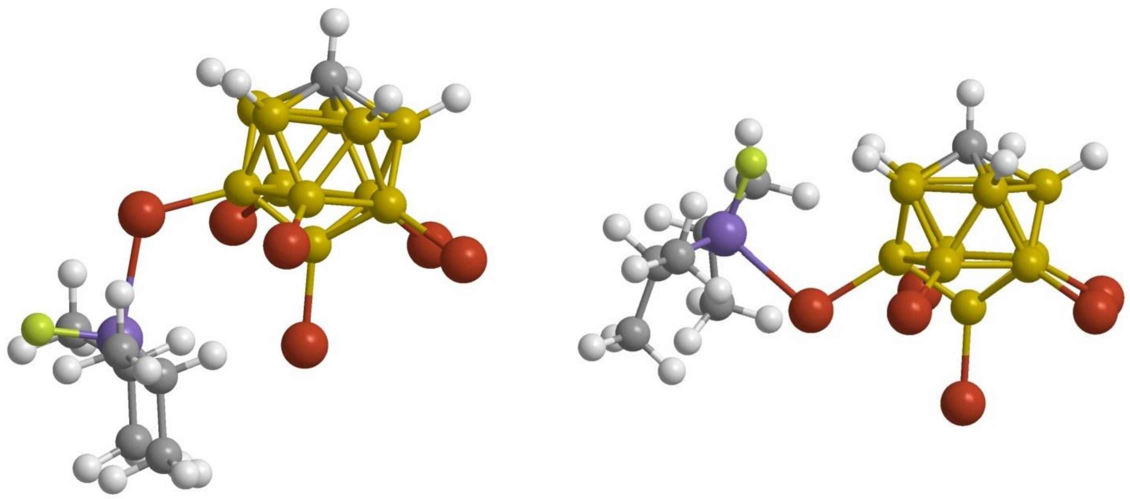

conformer **A** conformer **B**

Supplementary Fig. 12. Computed molecular structures of the two observed conformers exemplarily shown for silylium carborate [iPr$_2$FSi(HCB$_{11}$H$_5$Br$_6$)].

Supplementary Table 8. Computed ^{29}Si chemical shifts (in ppm relative to TMS) at the 2c/PBE0/x2c-TZVPall-2c level of theory for two conformers (**A** and **B**) of silylium carborates [$Alk_2XSi(HCB_{11}H_5Br_6)$] and the conformer energy differences $\Delta E = E_A - E_B$ (in kJ mol^{-1}) at the LH20t-D4/def2-TZVPD level of theory.[a,b]

	**A**	**B**	exp.	ΔE		**A**	**B**	exp.	ΔE
Me_2FSi^+	66.3	45.2	49.3	−12	Et_2FSi^+	66.8	46.0	50.6	−15
Me_2ClSi^+	77.8	59.6	66.2	−10	Et_2ClSi^+	80.7	68.2	72.5	−15
Me_2BrSi^+	73.2	55.1	62.3	−10	Et_2BrSi^+	78.4	70.3	73.5	−14
Me_2ISi^+	50.5	31.1	41.7	−10	Et_2ISi^+	66.4	60.7	64.3	−14
	**A**	**B**	exp.	ΔE		**A**	**B**	exp.	ΔE
$i Pr_2FSi^+$	67.4	46.8	47.0	−17	$t Bu_2FSi^+$	72.1	46.4	47.6	11
$i Pr_2ClSi^+$	93.1	73.2	74.3	−19	$t Bu_2ClSi^+$	102.3	77.7	80.5	11
$i Pr_2BrSi^+$	99.8	76.9	79.3	−21	$t Bu_2BrSi^+$	110.7	85.3	88.5	10
$i Pr_2ISi^+$	102.4	70.8	78.8	−23	$t Bu_2ISi^+$	117.3	86.6	93.5	9

[a]For the molecular structures of the two conformers **A** and **B**, see Supplementary Fig. 12. [b]A negative ΔE value means conformer **A** is more stable.

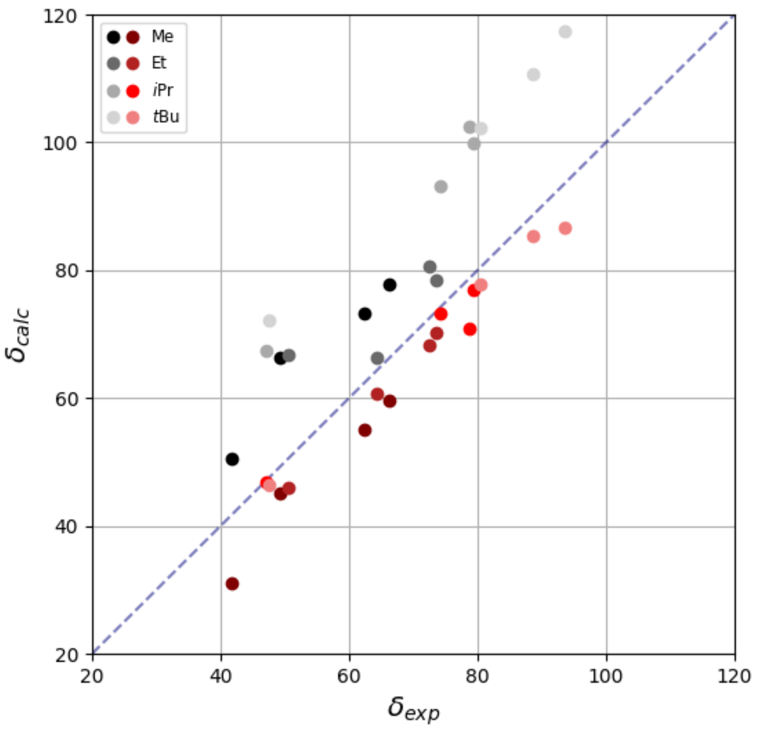

Supplementary Fig. 13. Comparison of the computed ^{29}Si chemical shifts (in ppm relative to TMS) at the PBE0 two-component level including spin–orbit coupling for the two different conformer types **A** and **B** (**A** in black and grey tones, **B** in red tones).[a] Linear regression for conformer type **A**: y = 1.12x + 8.16, R^2 = 0.88; linear regression for conformer type **B**: y = 1.00x – 4.68, R^2 = 0.97.

8 Analytical spectra

Supplementary Fig. 14. **¹H NMR** spectrum (500 MHz, C_6D_6, 298 K) of fluorodimethyl(phenyl)silane (**7aa**)

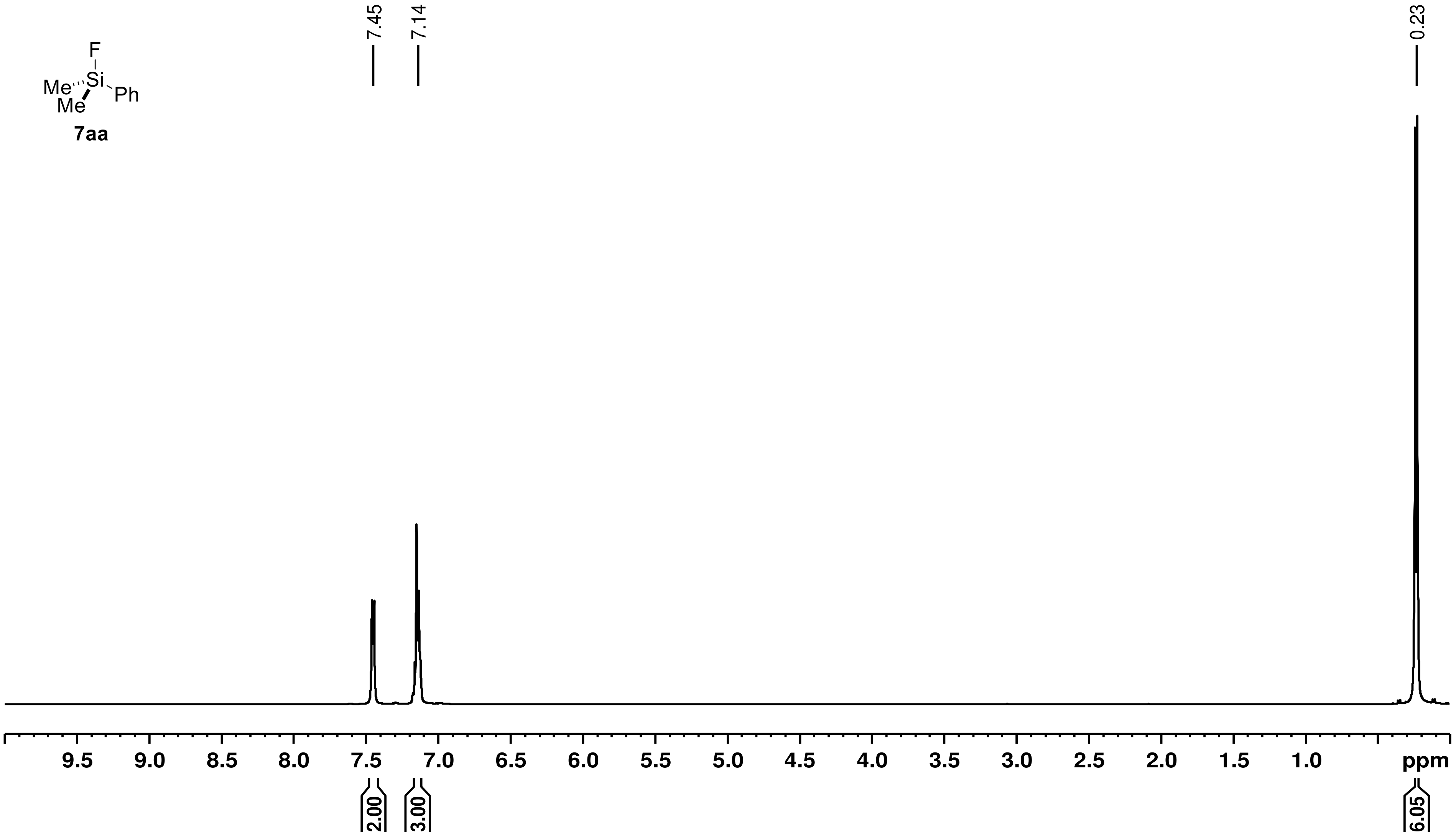

Supplementary Fig. 15. **$^{13}C\{^{1}H\}$ NMR** spectrum (126 MHz, C_6D_6, 298 K) of fluorodimethyl(phenyl)silane (**7aa**)

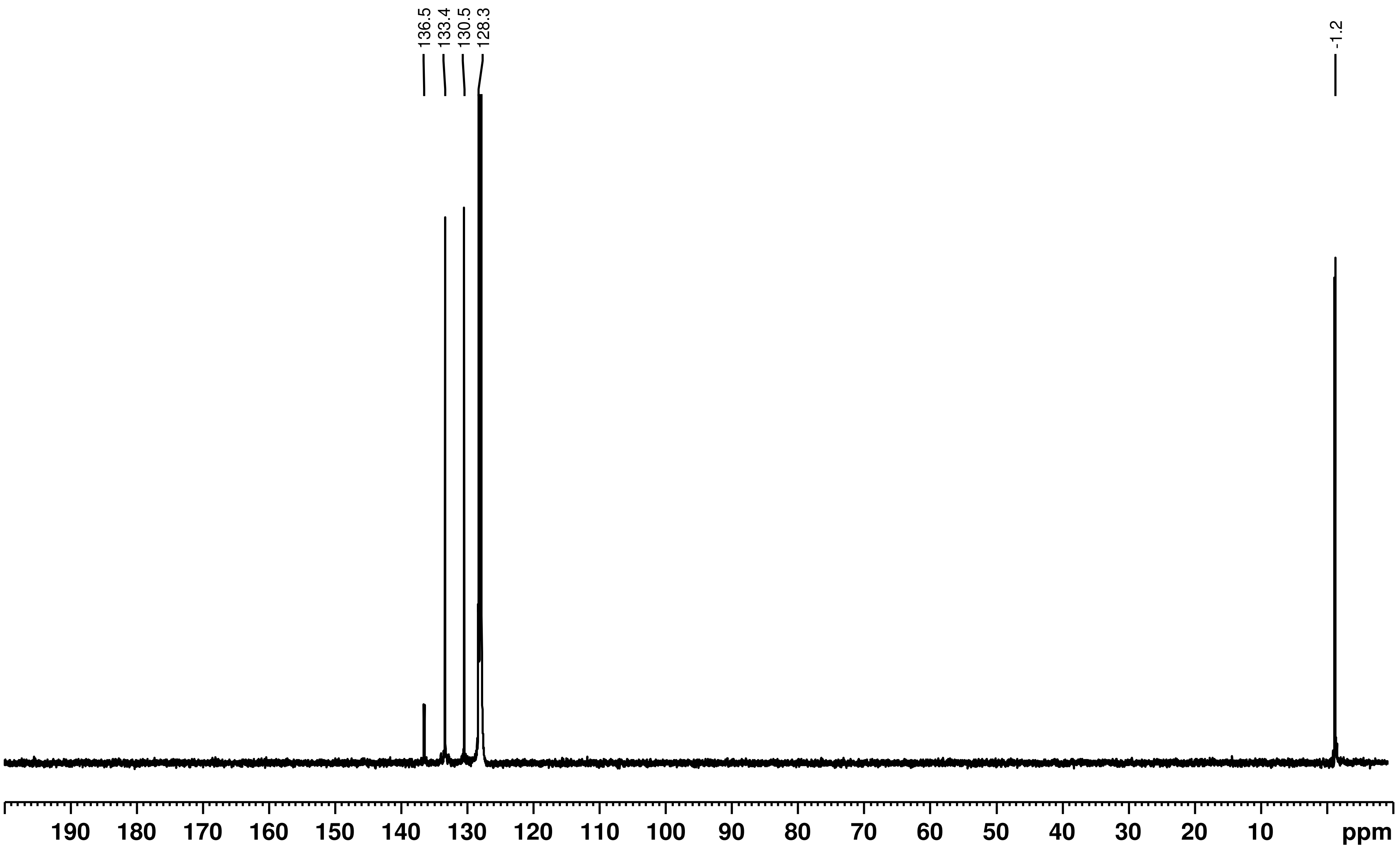

Supplementary Fig. 16. ^{19}**F NMR** spectrum (471 MHz, C_6D_6, 298 K) of fluorodimethyl(phenyl)silane (**7aa**)

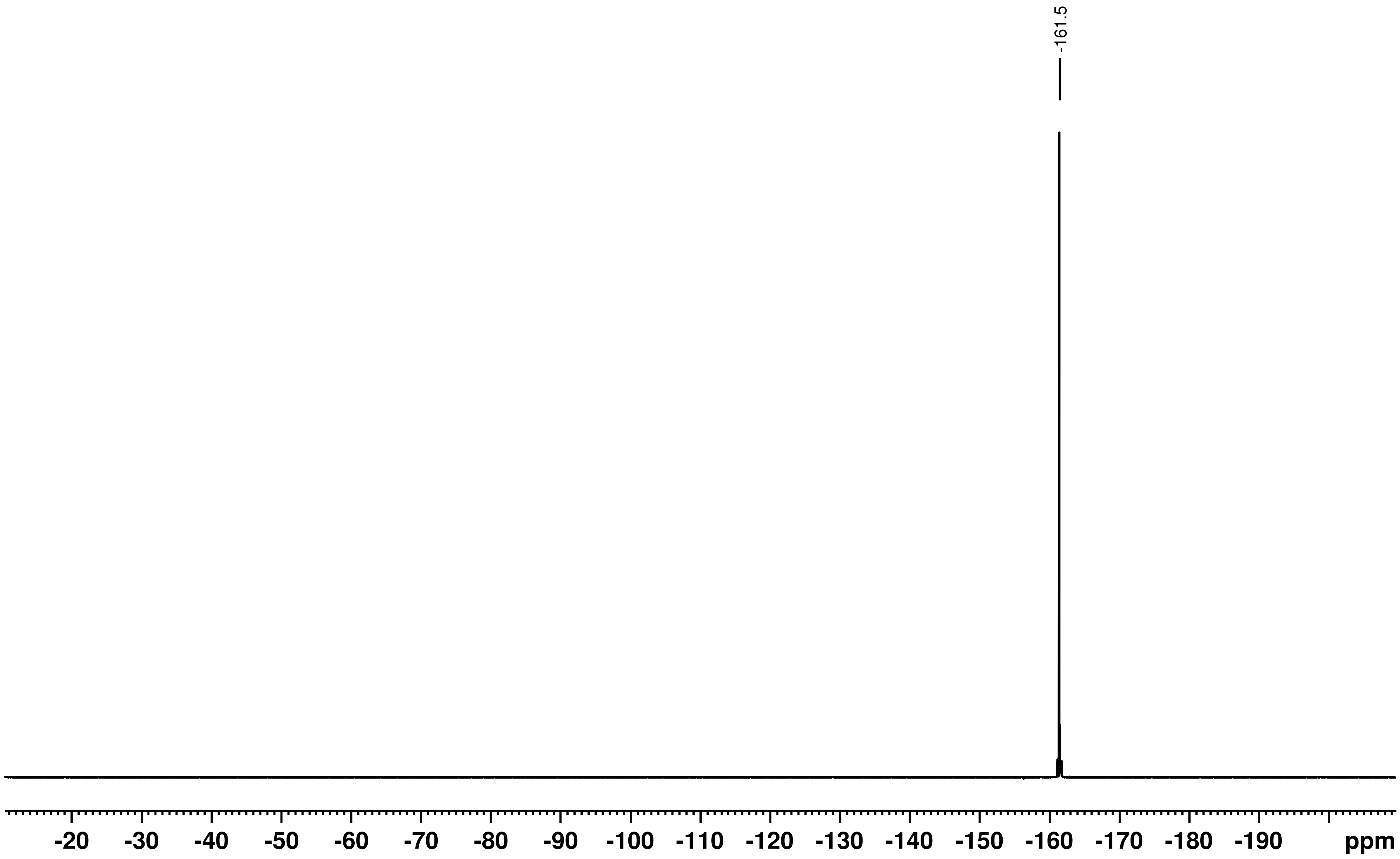

Supplementary Fig. 17. **^{29}Si{^{1}H} DEPT NMR** spectrum (99 MHz, C_6D_6, 298 K, optimized for $J_{H,Si}$ = 7 Hz, 24.1°) of fluorodimethyl(phenyl)silane (**7aa**)

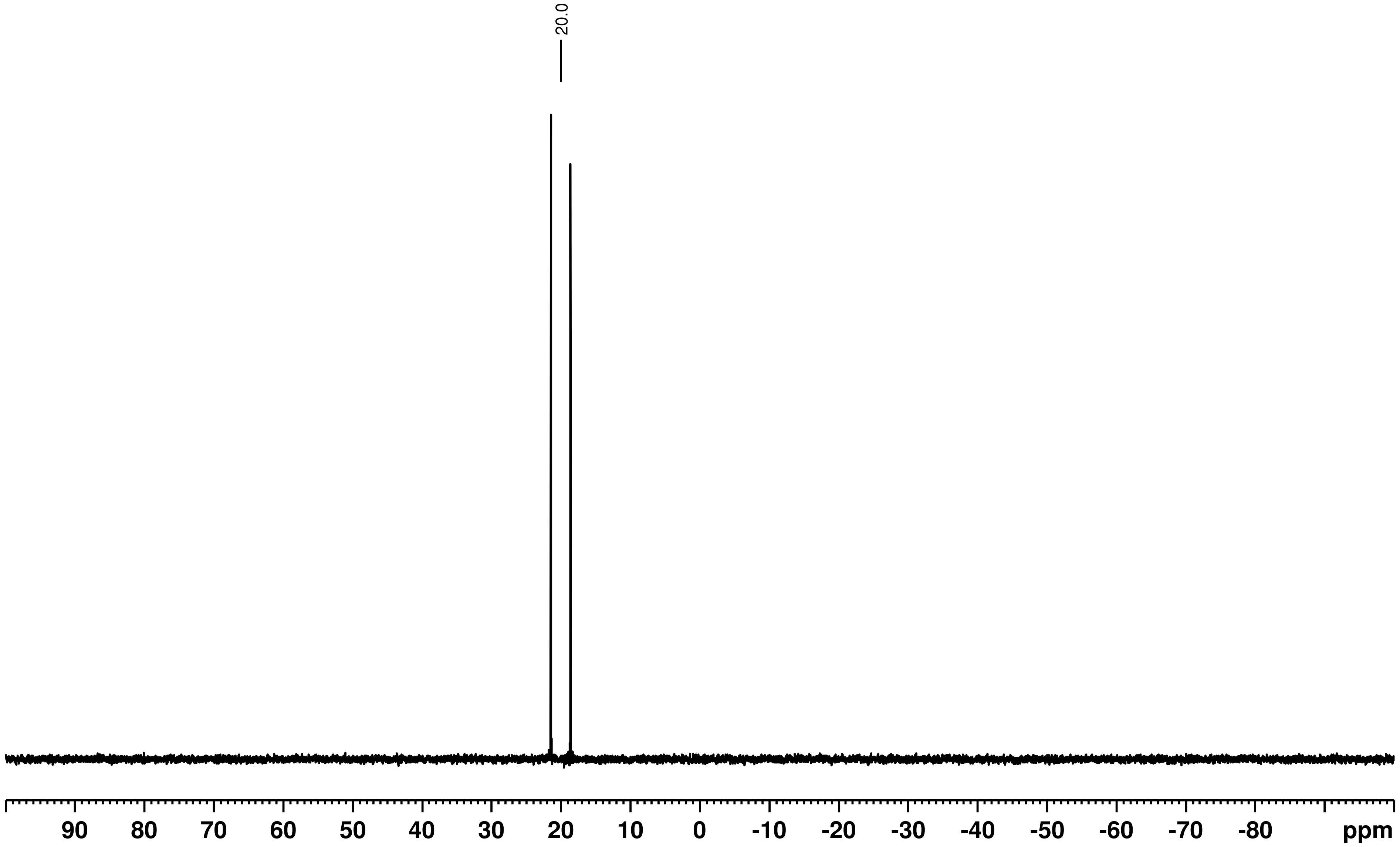

Supplementary Fig. 18. **IR** spectrum (ATR) of fluorodimethyl(phenyl)silane (**7aa**)

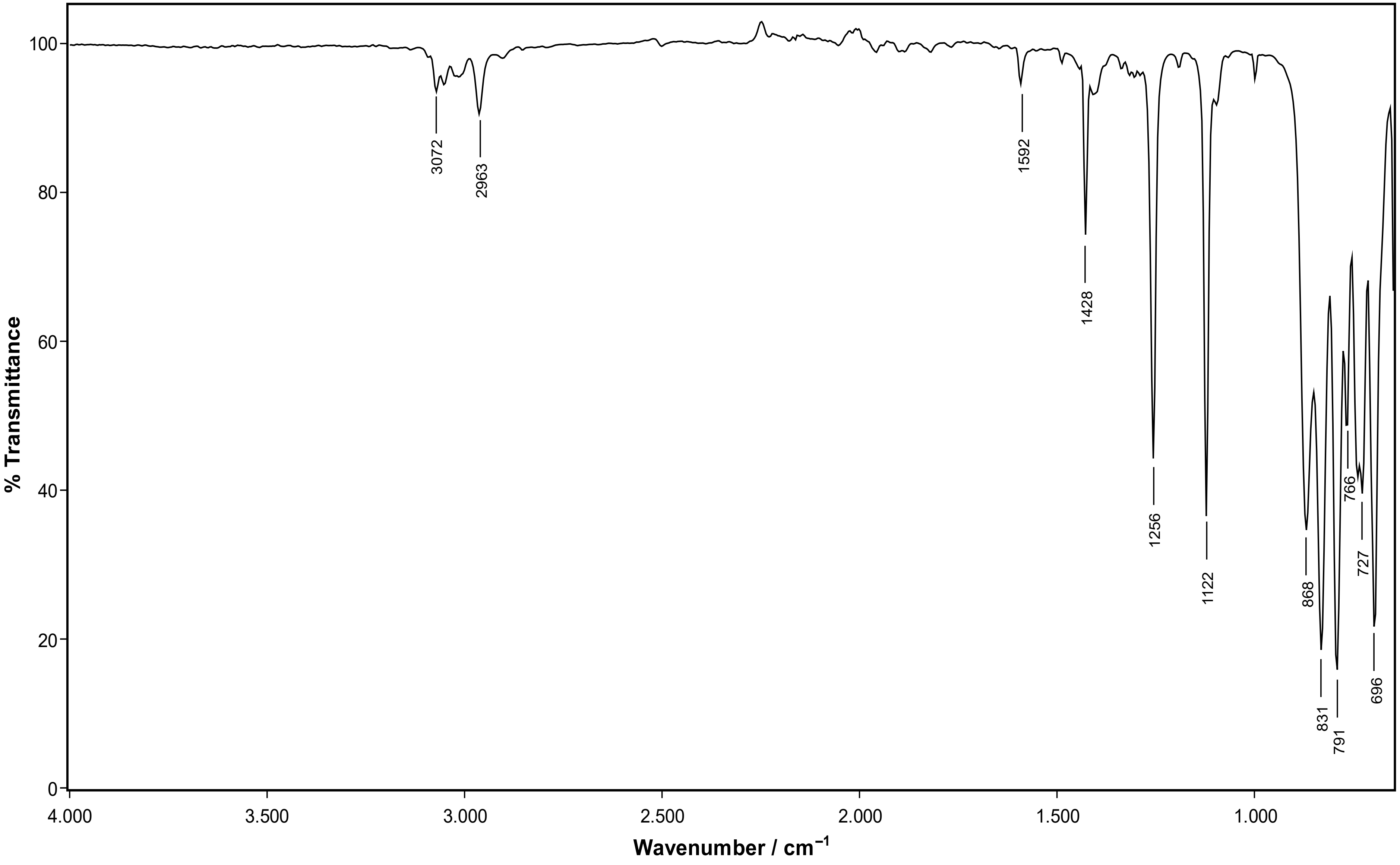

Supplementary Fig. 19. **GLC-MS** spectrum (EI) of fluorodimethyl(phenyl)silane (**7aa**)

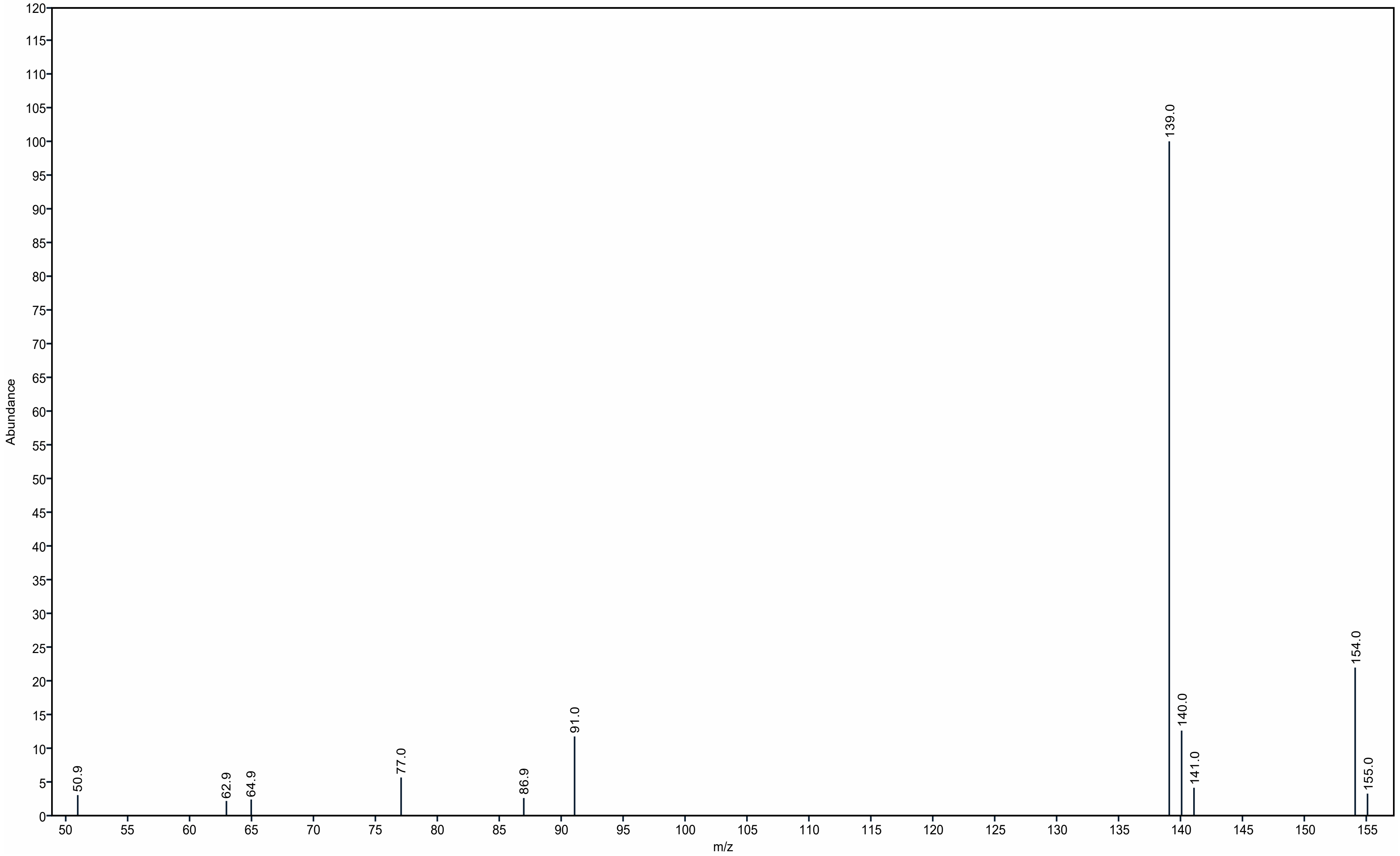

Supplementary Fig. 20. ^{1}**H NMR** spectrum (500 MHz, C_6D_6, 298 K) of bromodimethyl(phenyl)silane (**7ca**)

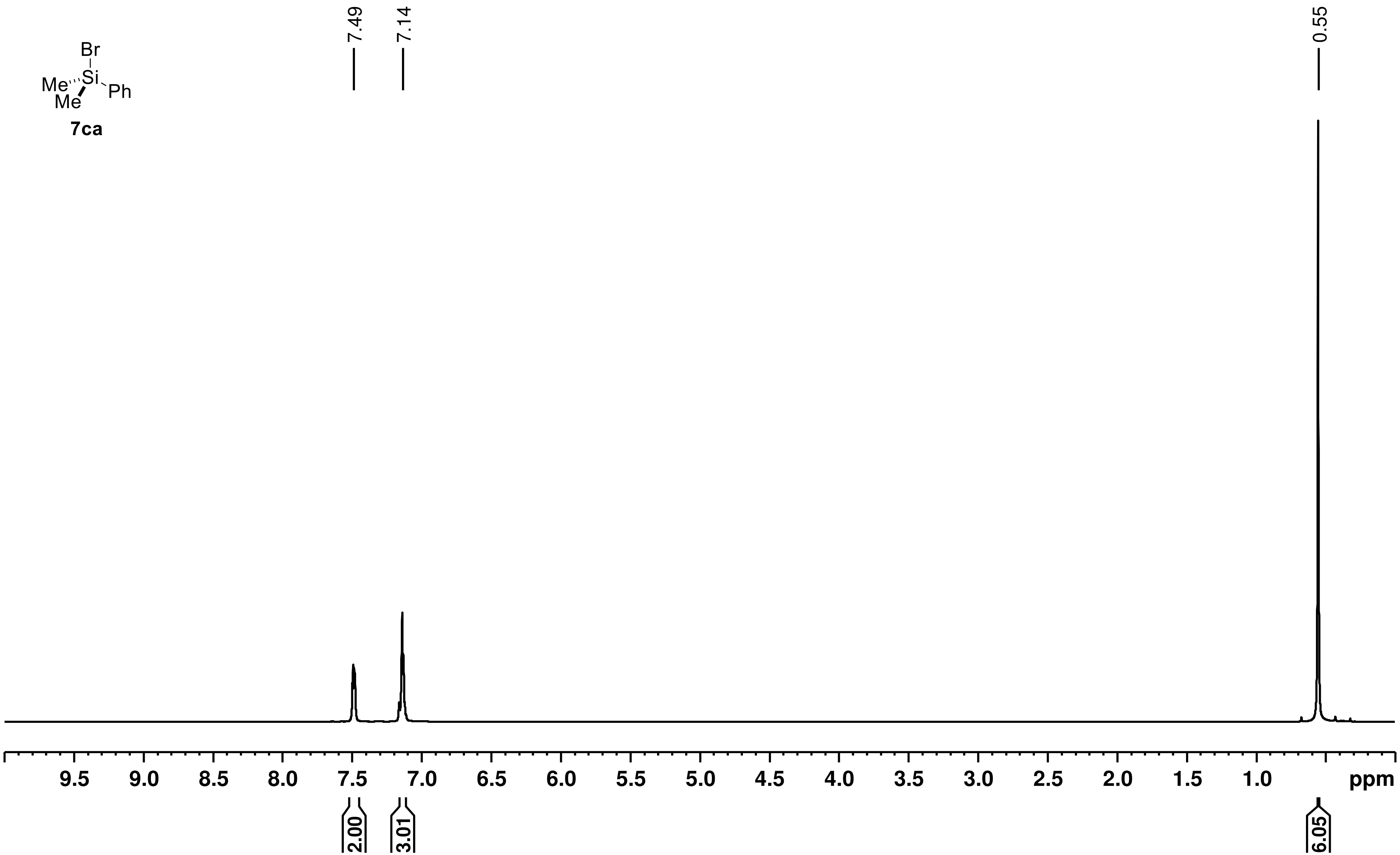

Supplementary Fig. 21. **^{13}C{^{1}H} NMR** spectrum (126 MHz, C_6D_6, 298 K) of bromodimethyl(phenyl)silane (**7ca**)

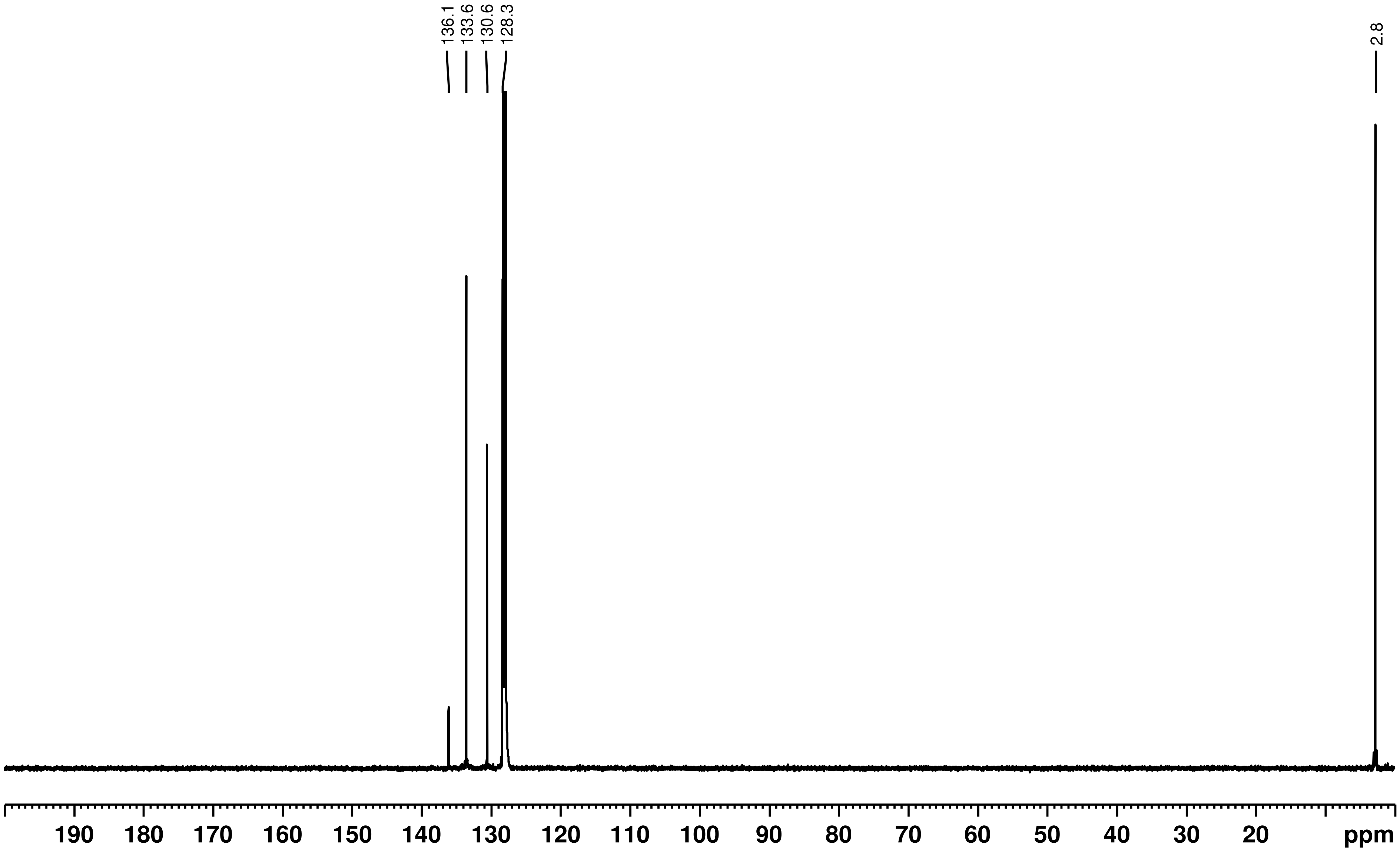

Supplementary Fig. 22. **^{29}Si{^{1}H} DEPT NMR** spectrum (99 MHz, C_6D_6, 298 K, optimized for $J_{H,Si}$ = 7 Hz, 20.7°) of bromodimethyl(phenyl)silane (**7ca**)

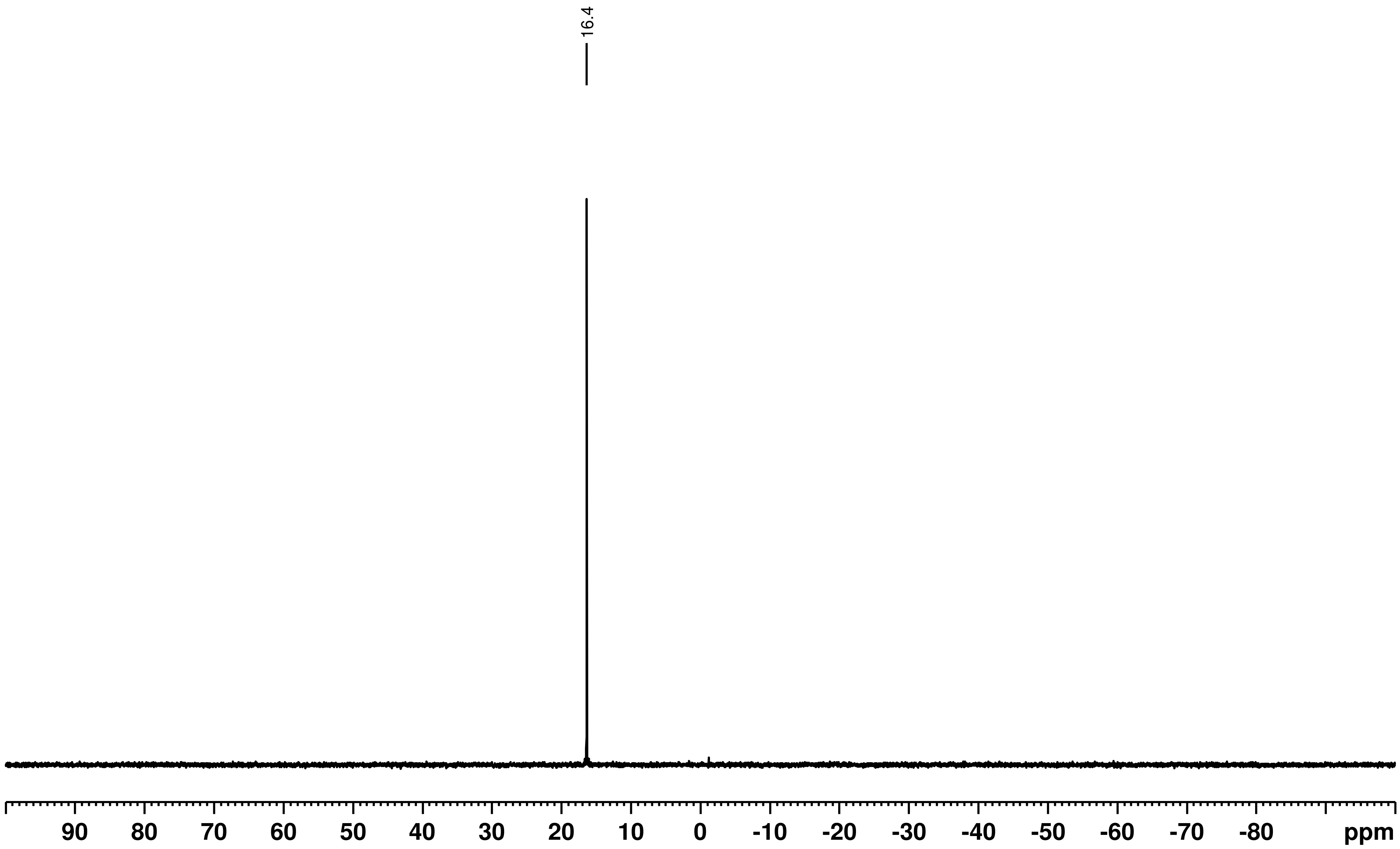

Supplementary Fig. 23. ^{1}**H NMR** spectrum (500 MHz, C_6D_6, 298 K) of iododimethyl(phenyl)silane (**7da**)

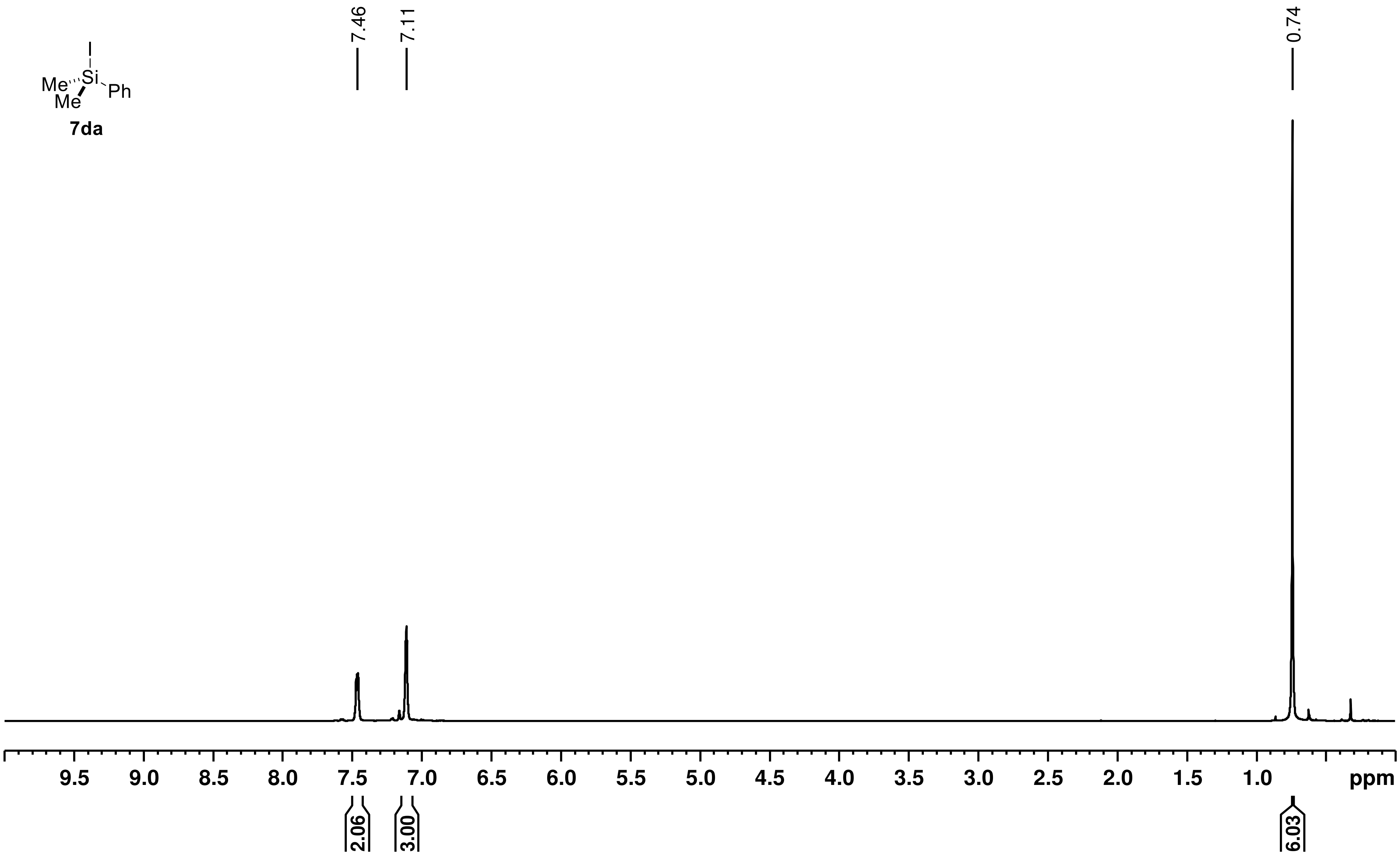

Supplementary Fig. 24. **^{13}C{^{1}H} NMR** spectrum (126 MHz, C_6D_6, 298 K) of iododimethyl(phenyl)silane (**7da**)

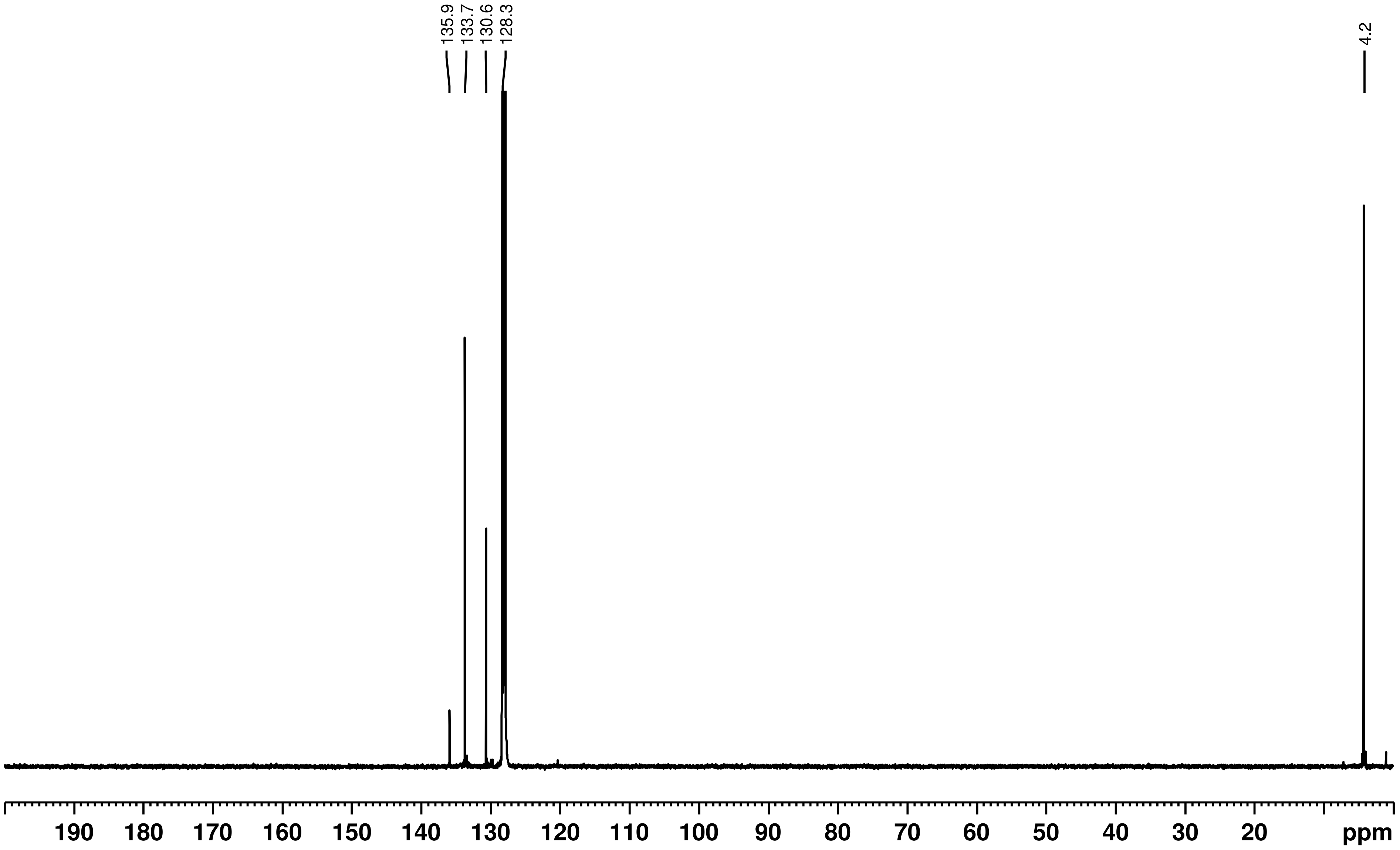

Supplementary Fig. 25. **^{29}Si{^{1}H} DEPT NMR** spectrum (99 MHz, C_6D_6, 298 K, optimized for $J_{H,Si}$ = 7 Hz, 20.7°) of iododimethyl(phenyl)silane (**7da**)

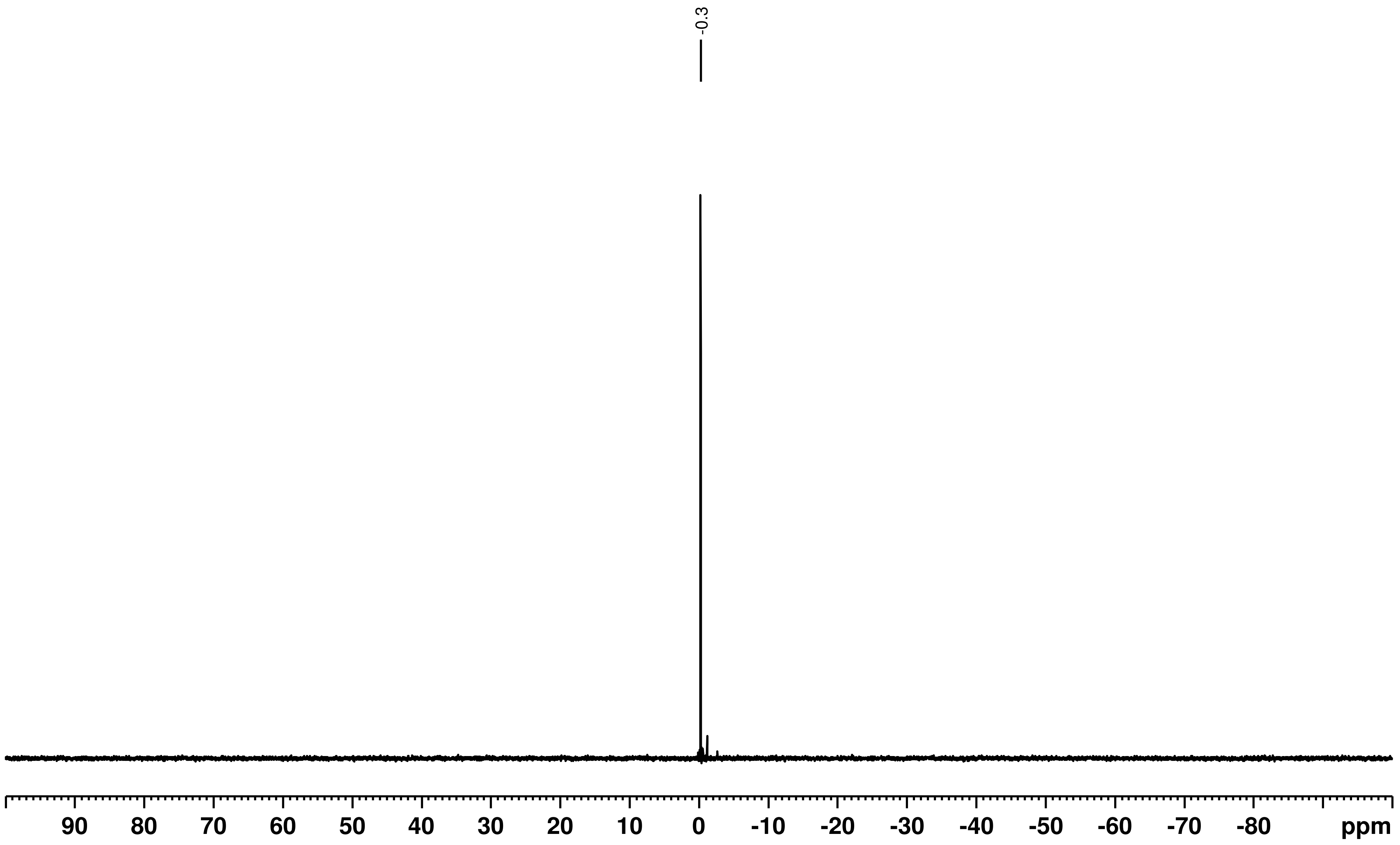

Supplementary Fig. 26. [1]**H NMR** spectrum (500 MHz, C_6D_6, 298 K) of ethyldifluoro(phenyl)silane (**S3**)

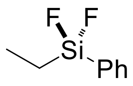

S3

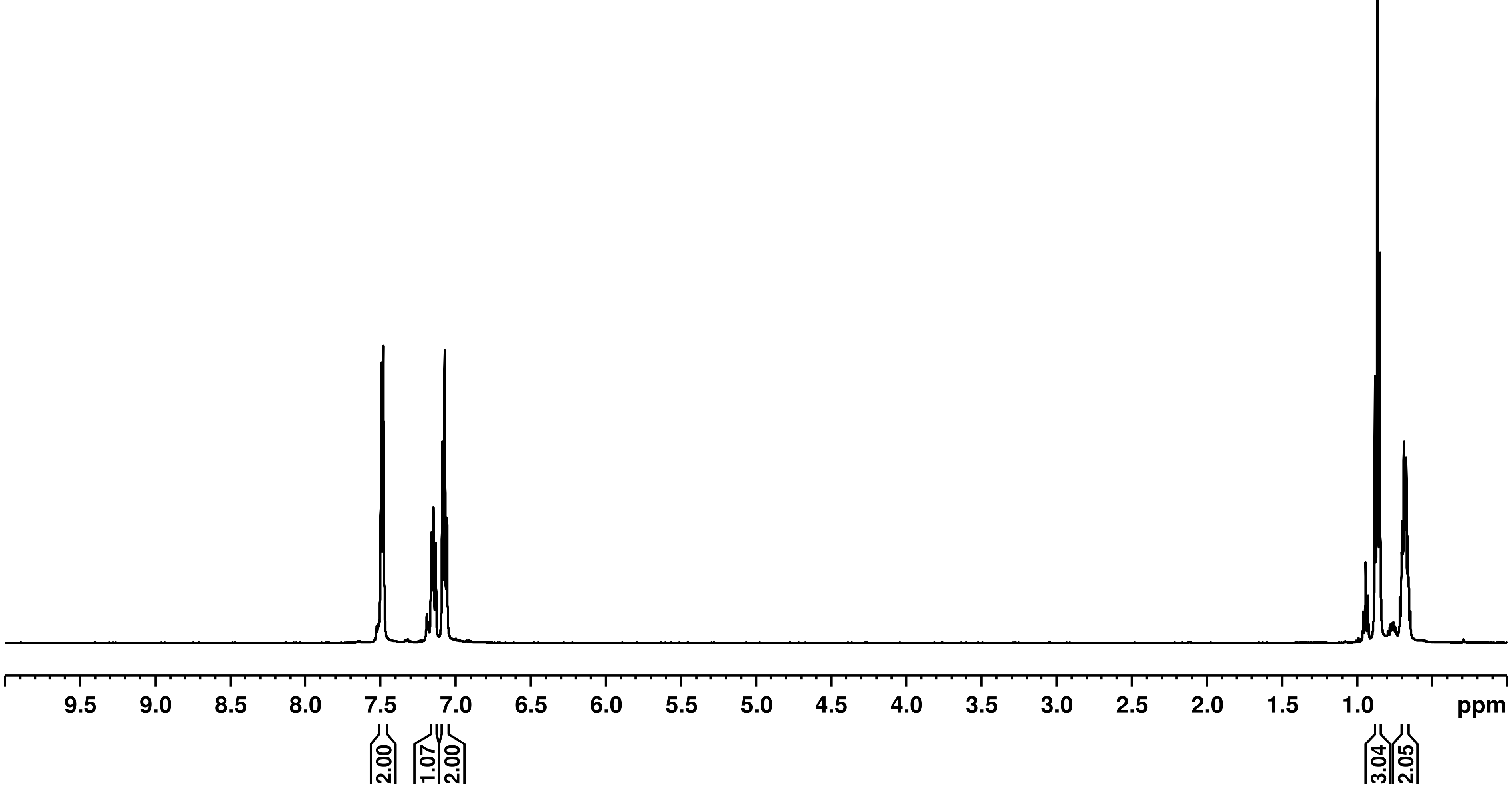

Supplementary Fig. 27. **^{13}C{^{1}H} NMR** spectrum (126 MHz, C_6D_6, 298 K) of ethyldifluoro(phenyl)silane (**S3**)

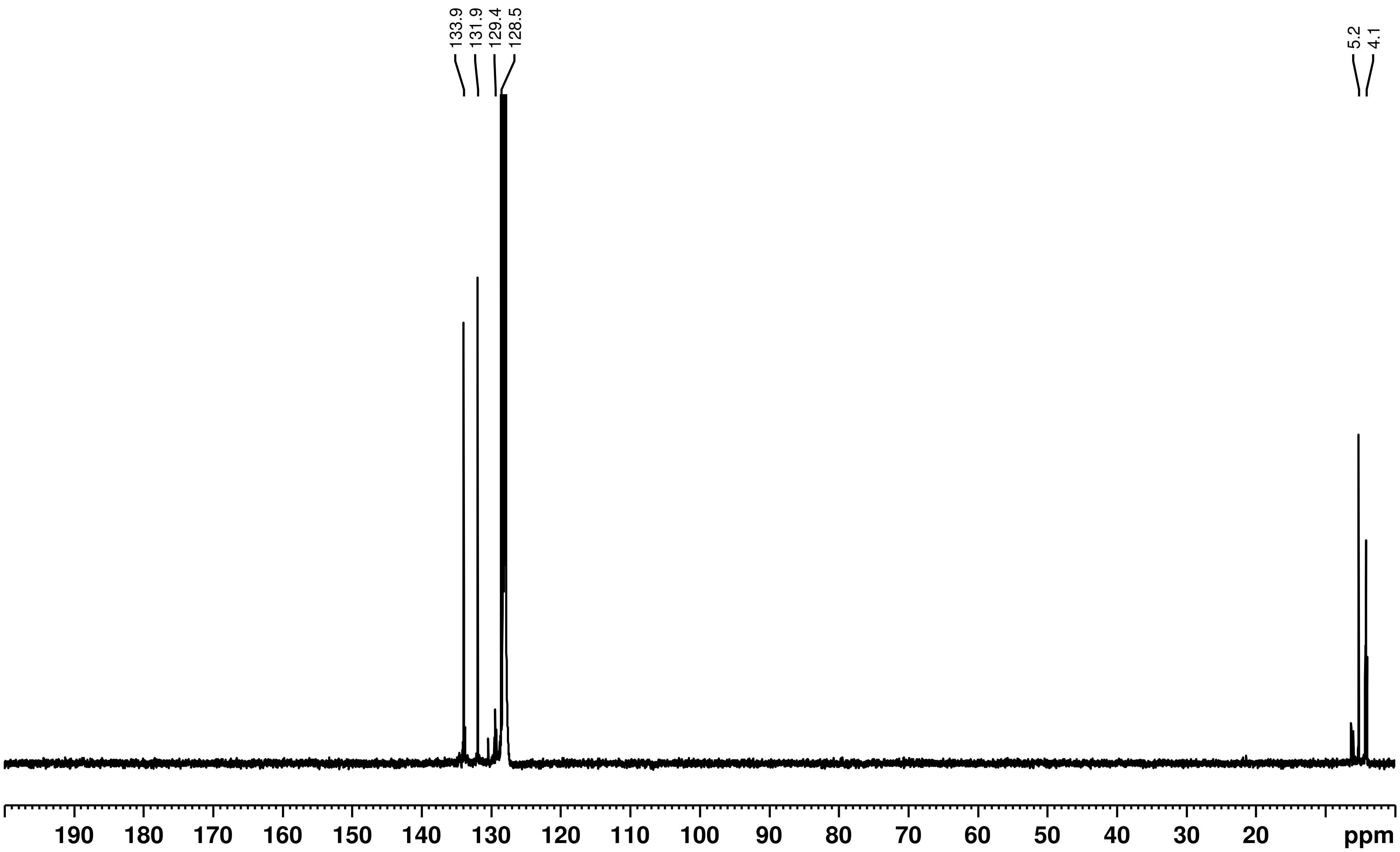

Supplementary Fig. 28. ^{19}**F NMR** spectrum (471 MHz, C_6D_6, 298 K) of ethyldifluoro(phenyl)silane (**S3**)

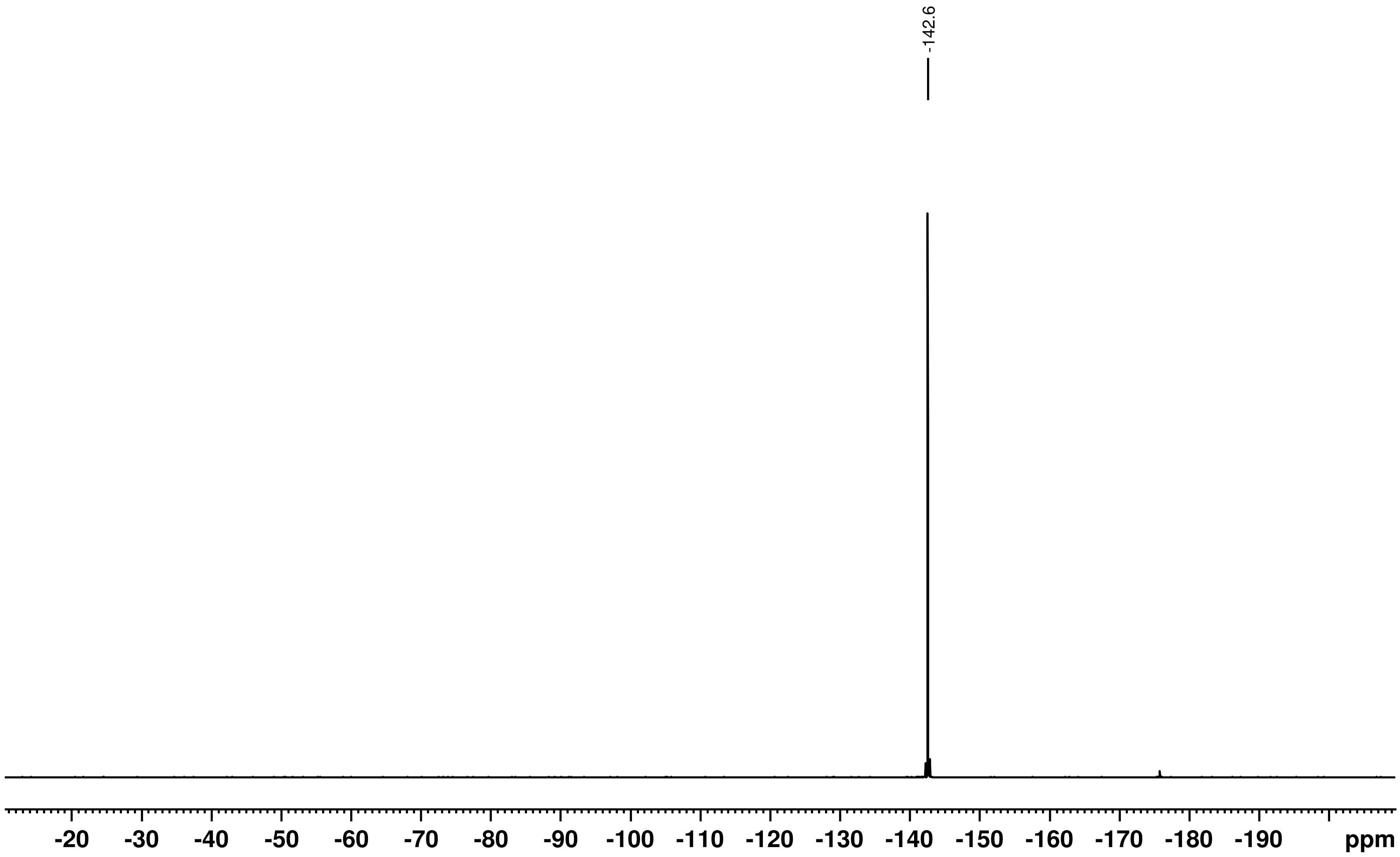

Supplementary Fig. 29. **^{29}Si{^{1}H} DEPT NMR** spectrum (99 MHz, C_6D_6, 298 K, optimized for $J_{H,Si}$ = 7 Hz, 22.2°) of ethyldifluoro(phenyl)silane (**S3**)

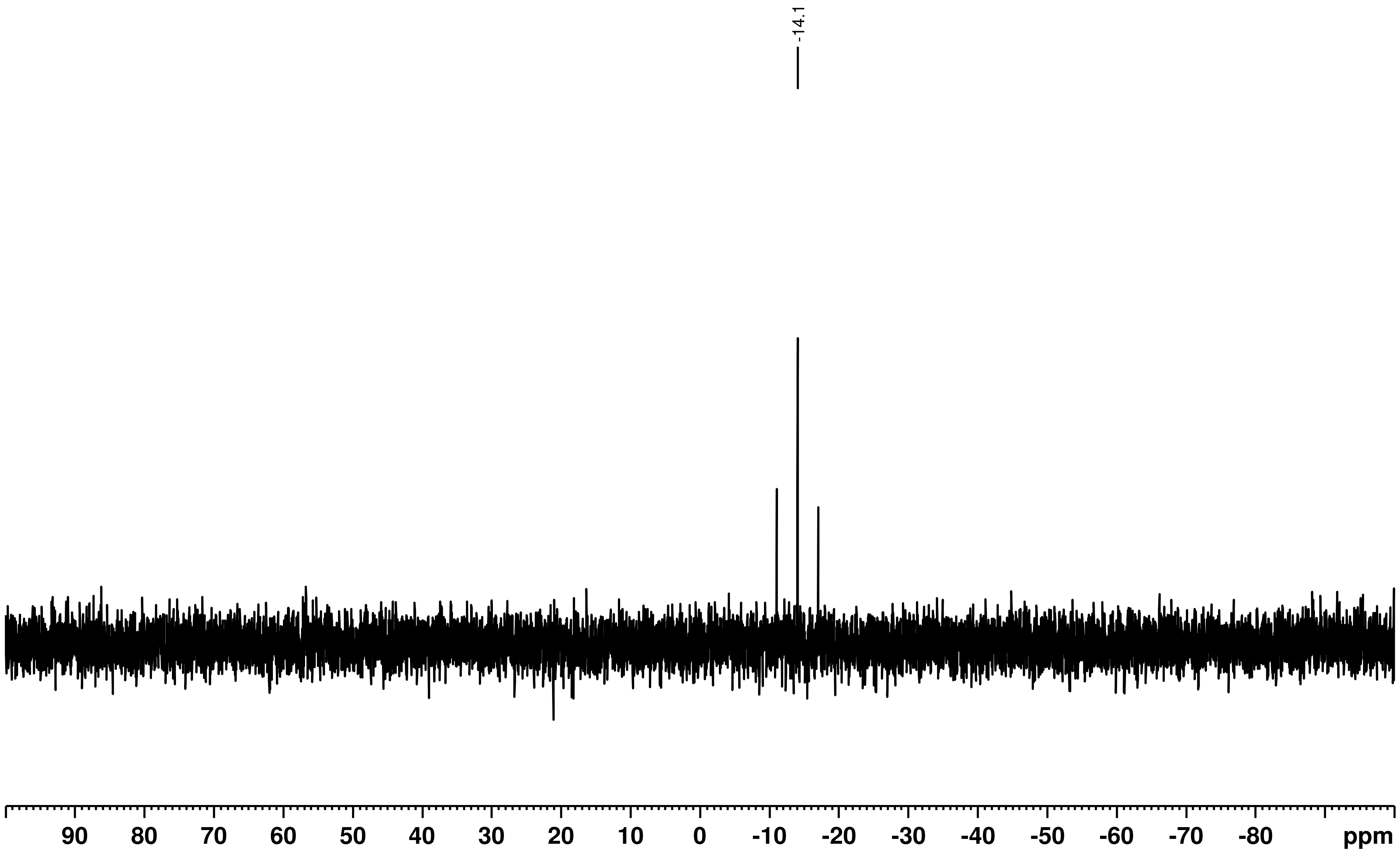

Supplementary Fig. 30. **IR** spectrum (ATR) of ethyldifluoro(phenyl)silane (**S3**)

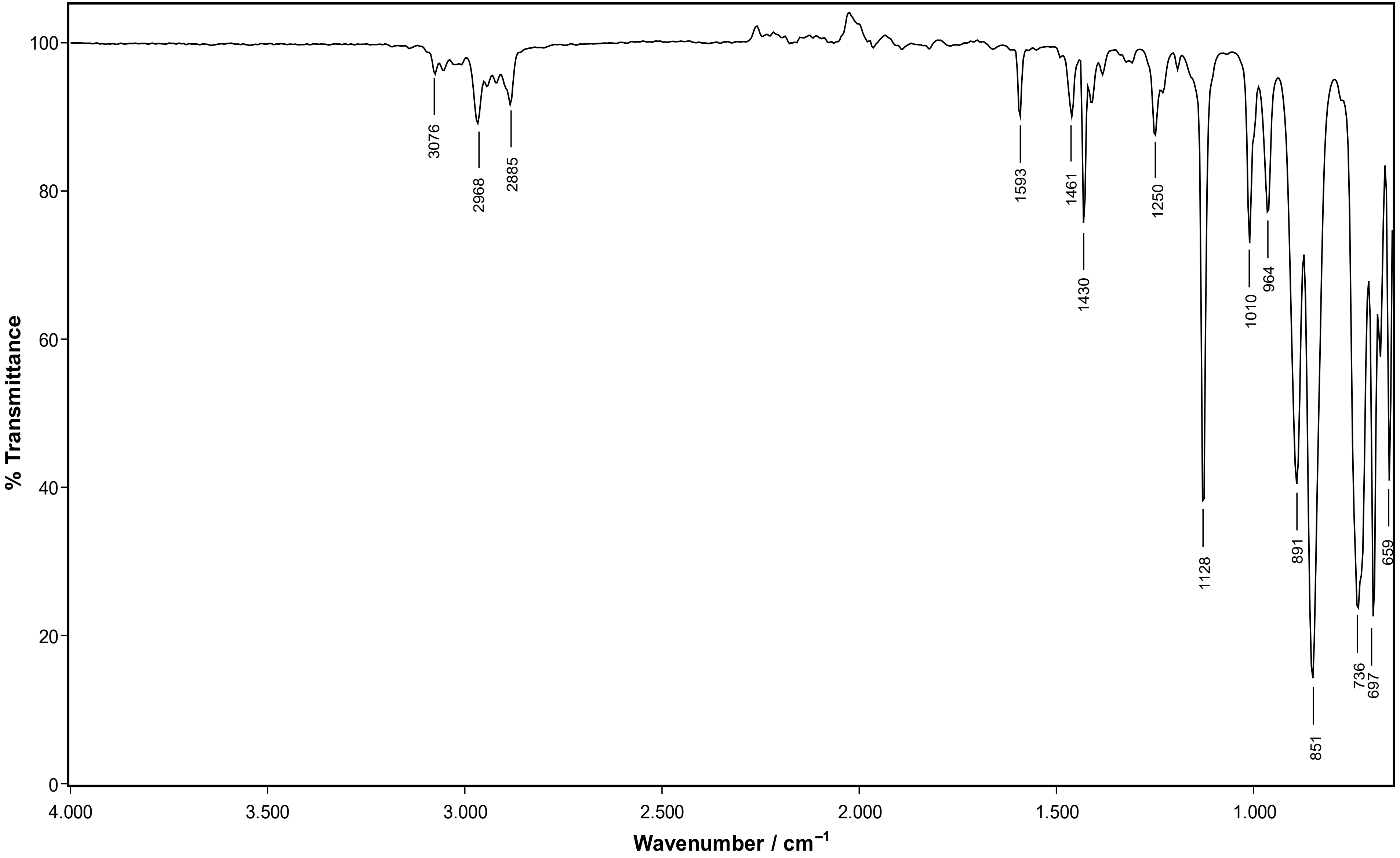

Supplementary Fig. 31. **GLC-MS** spectrum (EI) of ethyldifluoro(phenyl)silane (**S3**)

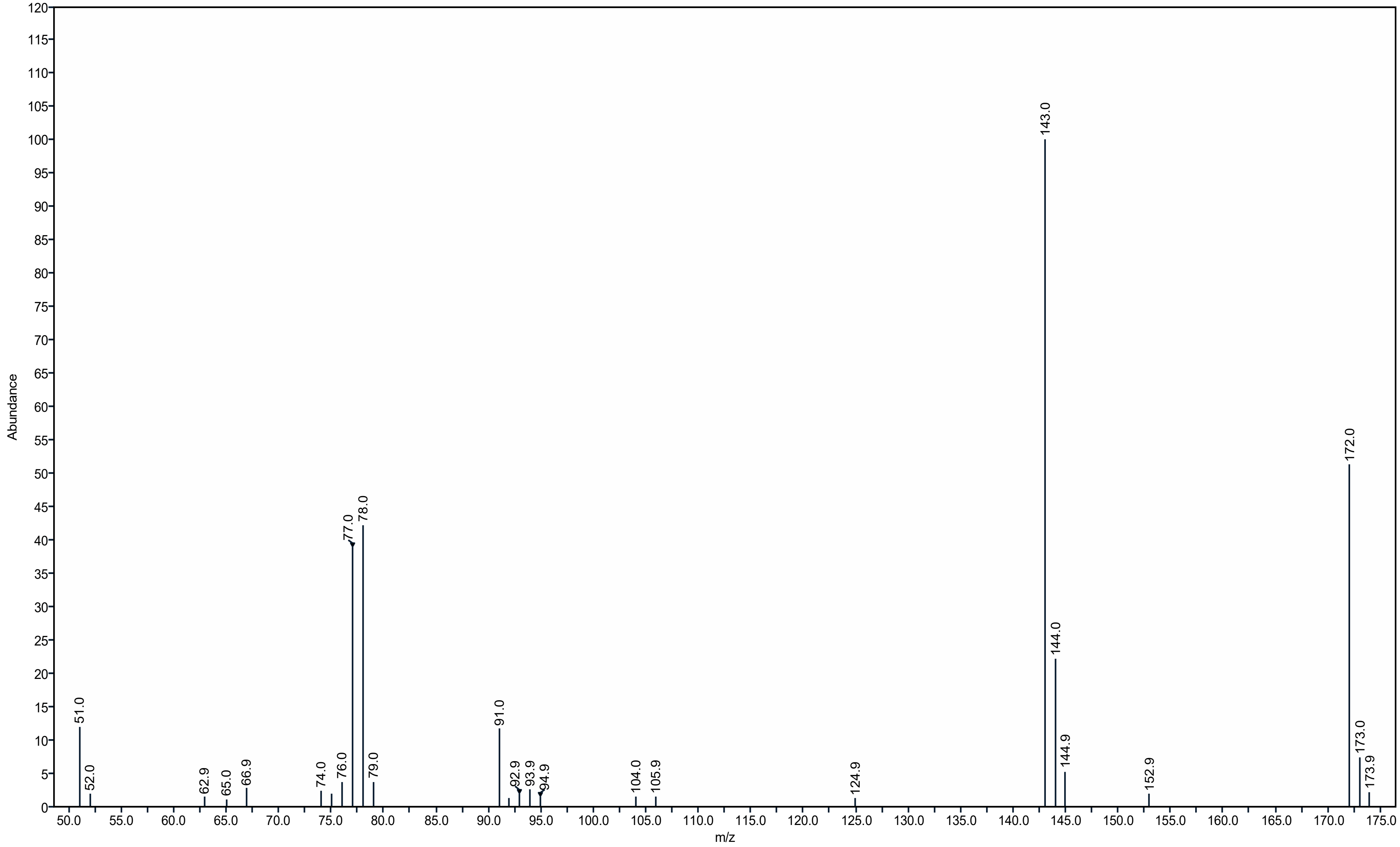

Supplementary Fig. 32. ^{1}**H NMR** spectrum (500 MHz, C_6D_6, 298 K) of diethylfluoro(phenyl)silane (**7ab**)

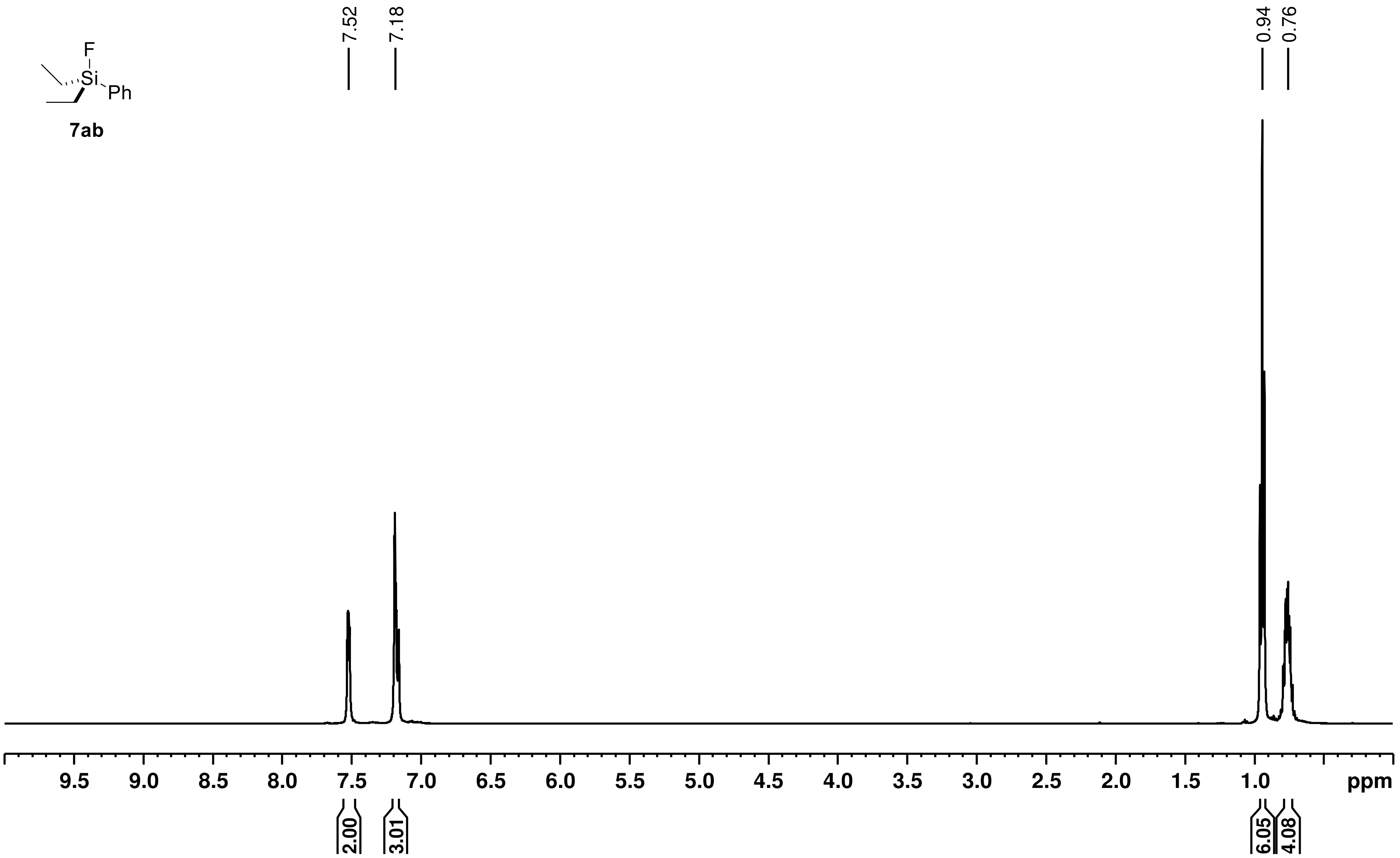

Supplementary Fig. 33. **$^{13}C\{^1H\}$ NMR** spectrum (126 MHz, C_6D_6, 298 K) of diethylfluoro(phenyl)silane (**7ab**)

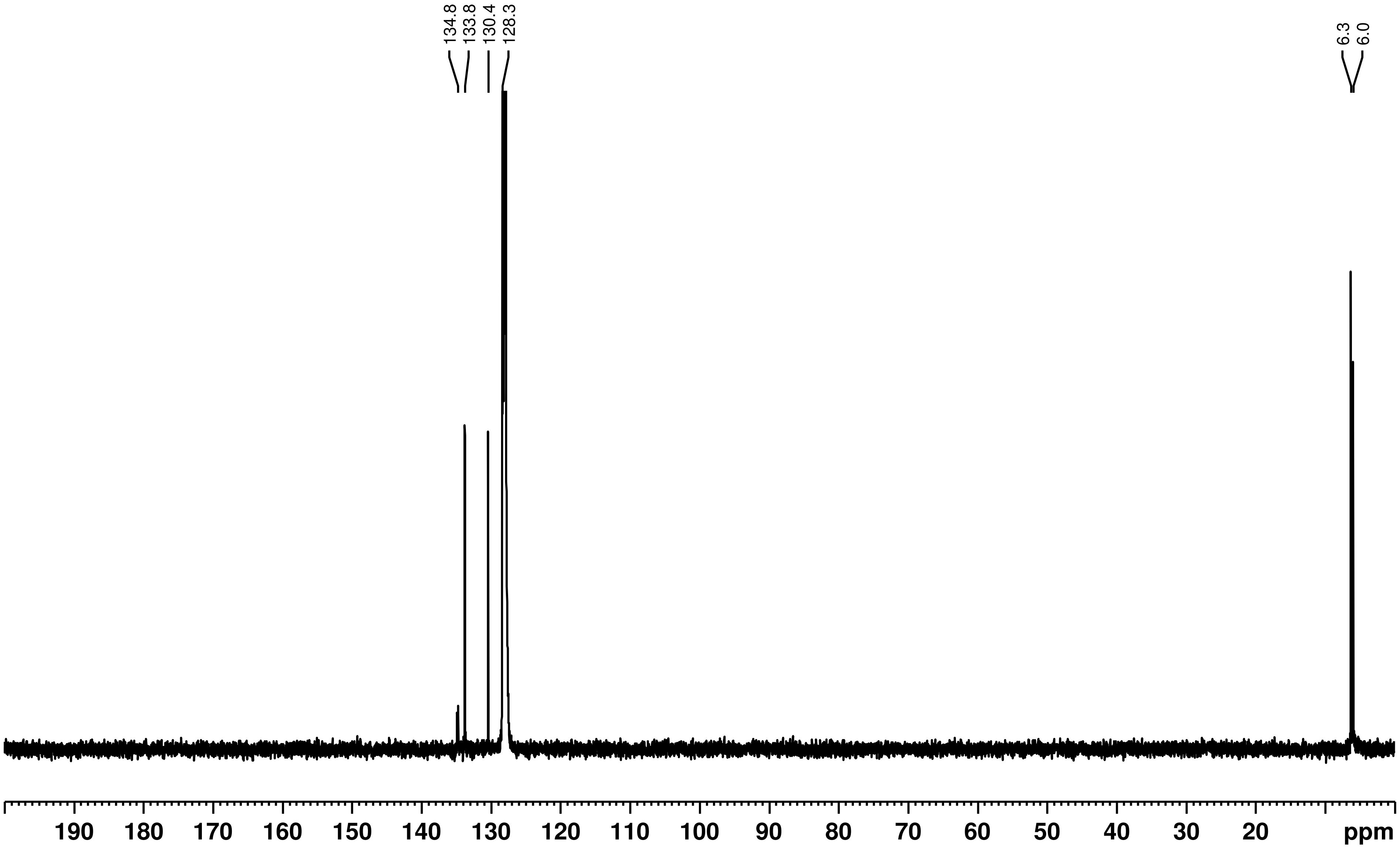

Supplementary Fig. 34. **^{19}F NMR** spectrum (471 MHz, C_6D_6, 298 K) of diethylfluoro(phenyl)silane (**7ab**)

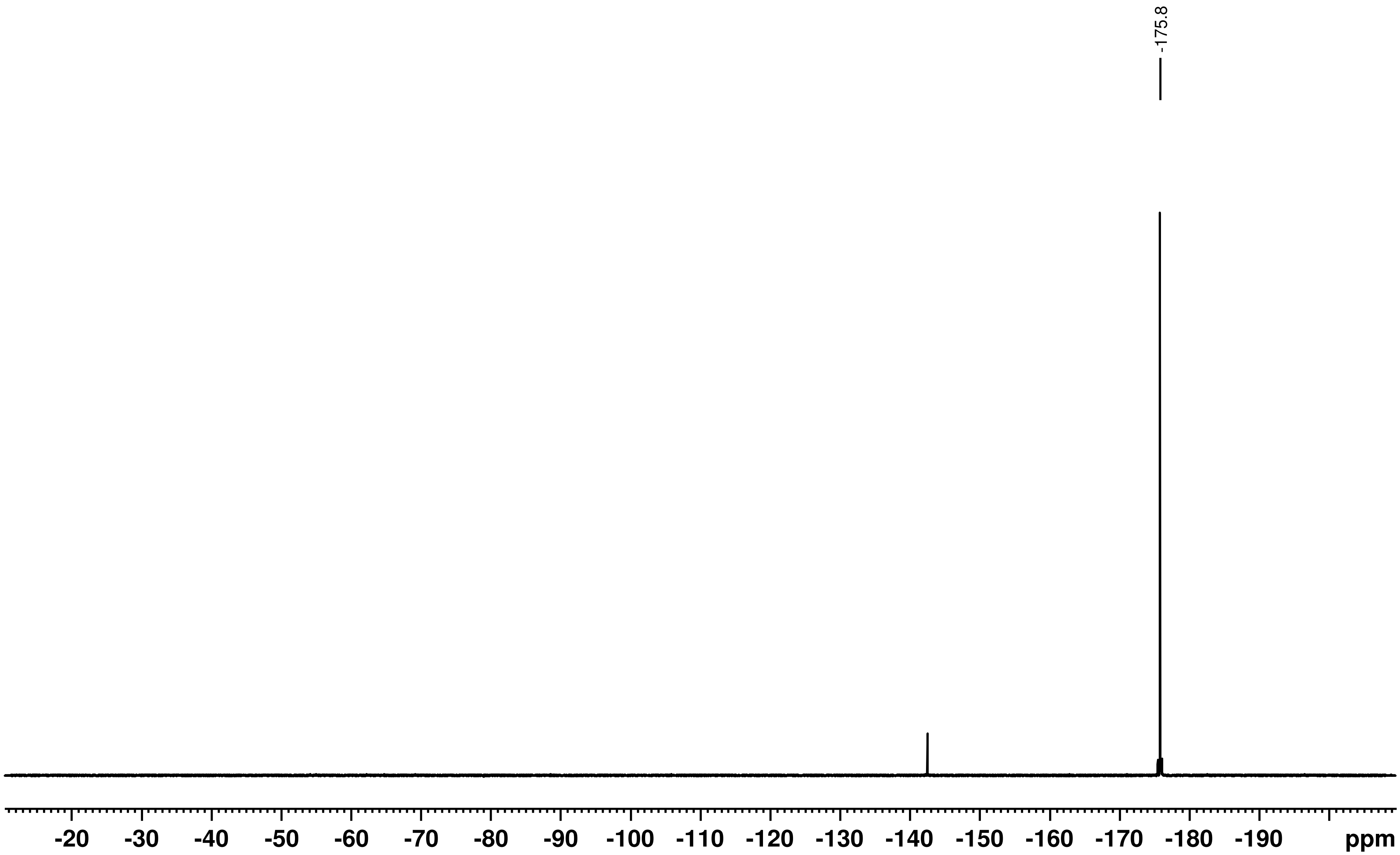

Supplementary Fig. 35. ^{29}**Si{^{1}H} DEPT NMR** spectrum (99 MHz, C_6D_6, 298 K, optimized for $J_{H,Si}$ = 7 Hz, 16.8°) of diethylfluoro(phenyl)silane (**7ab**)

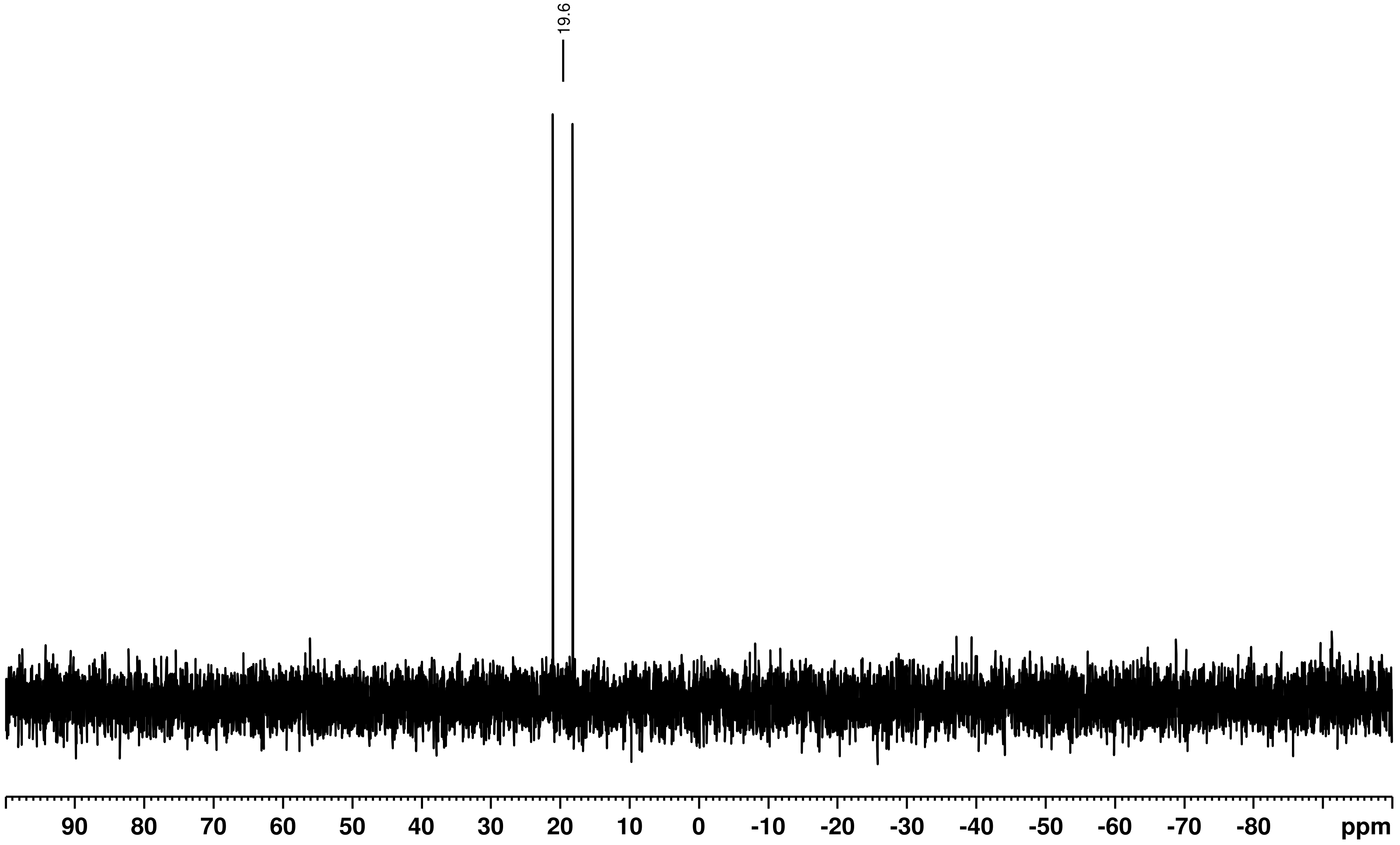

Supplementary Fig. 36. **IR** spectrum (ATR) of diethylfluoro(phenyl)silane (**7ab**)

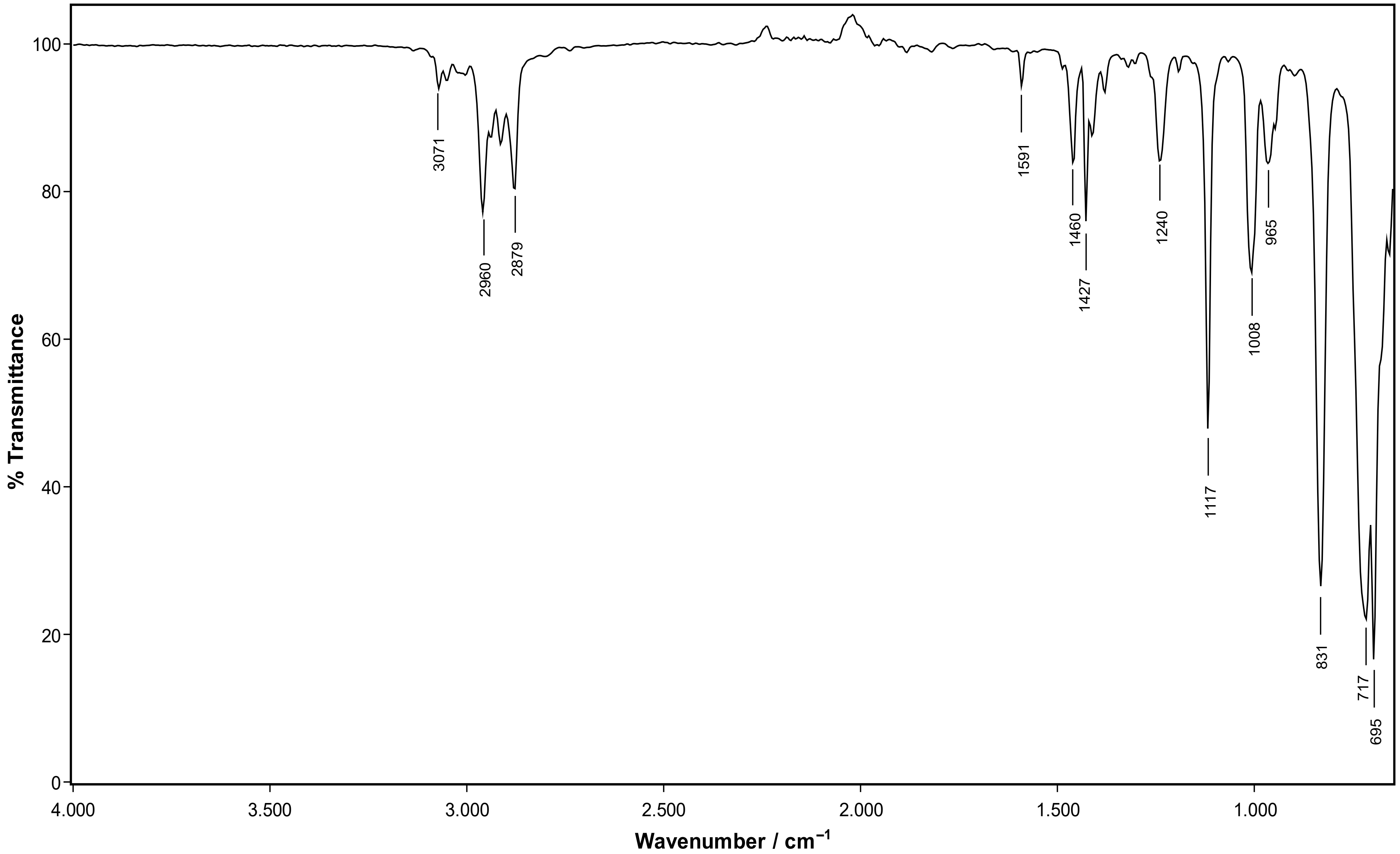

Supplementary Fig. 37. **GLC-MS** spectrum (EI) of diethylfluoro(phenyl)silane (**7ab**)

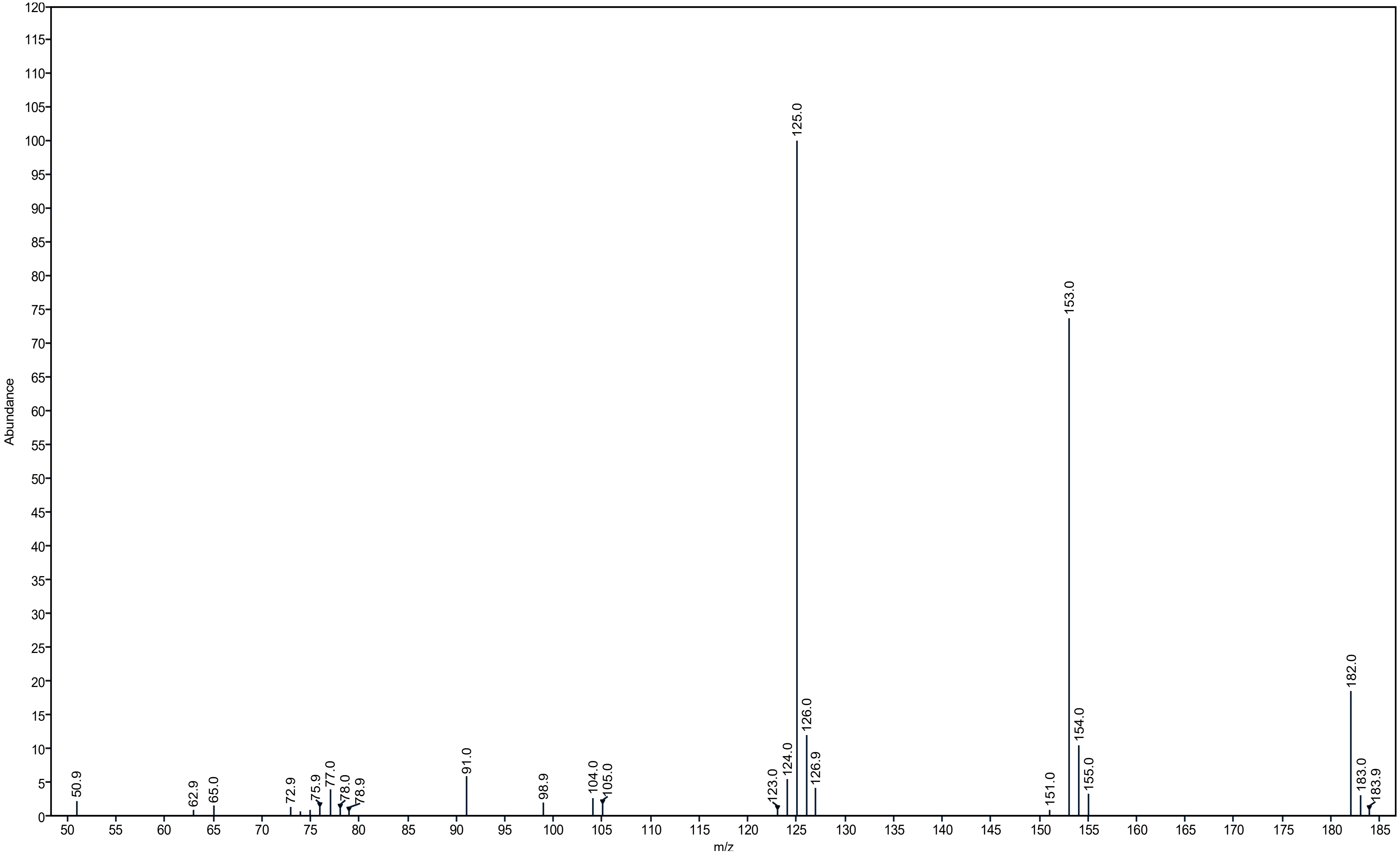

Supplementary Fig. 38. ^{1}**H NMR** spectrum (500 MHz, C_6D_6, 298 K) of chlorodiethylsilane (**6bb**) (* Et_3SiH)

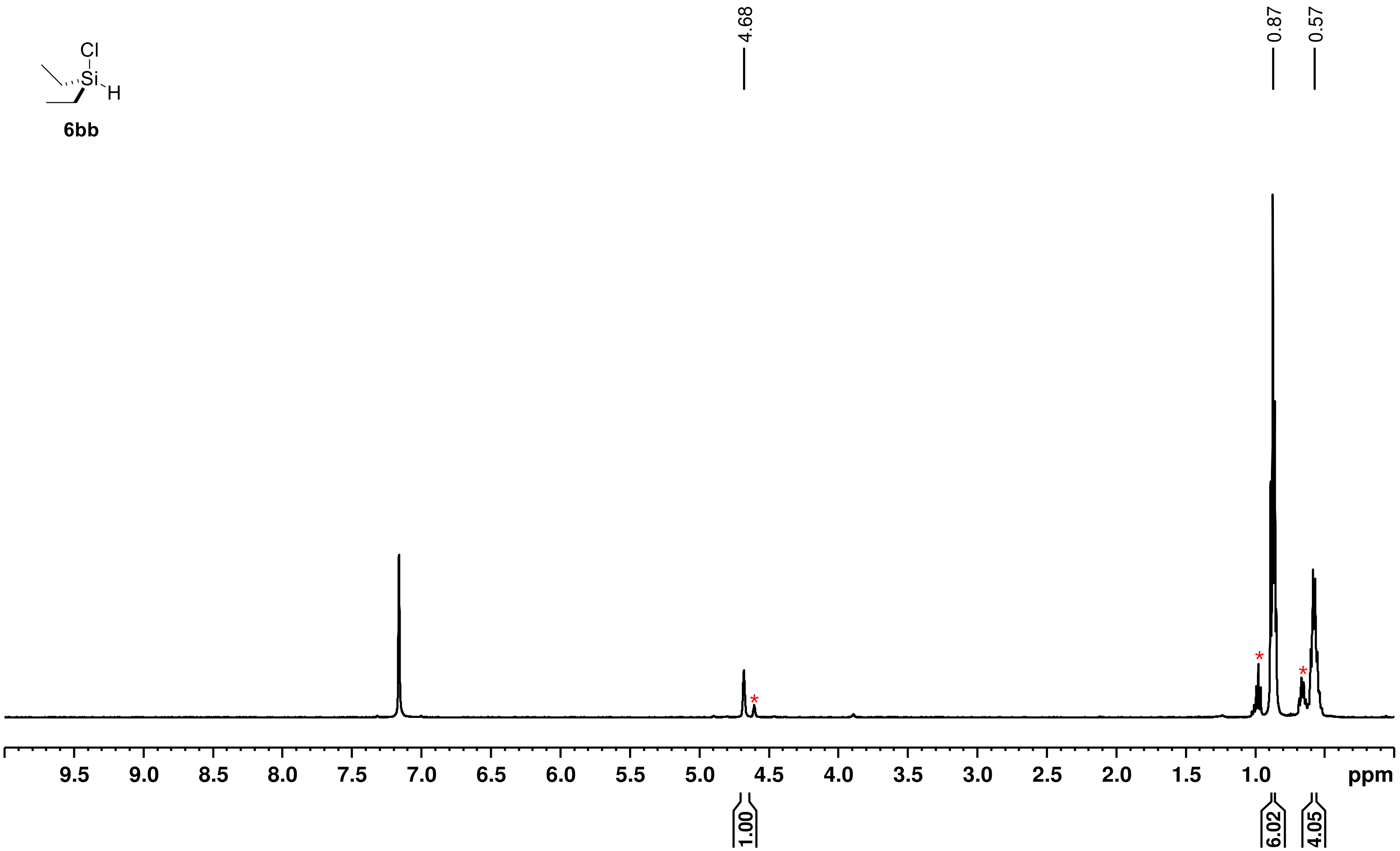

Supplementary Fig. 39. **^{13}C{^{1}H} NMR** spectrum (126 MHz, C_6D_6, 298 K) of chlorodiethylsilane (**6bb**)

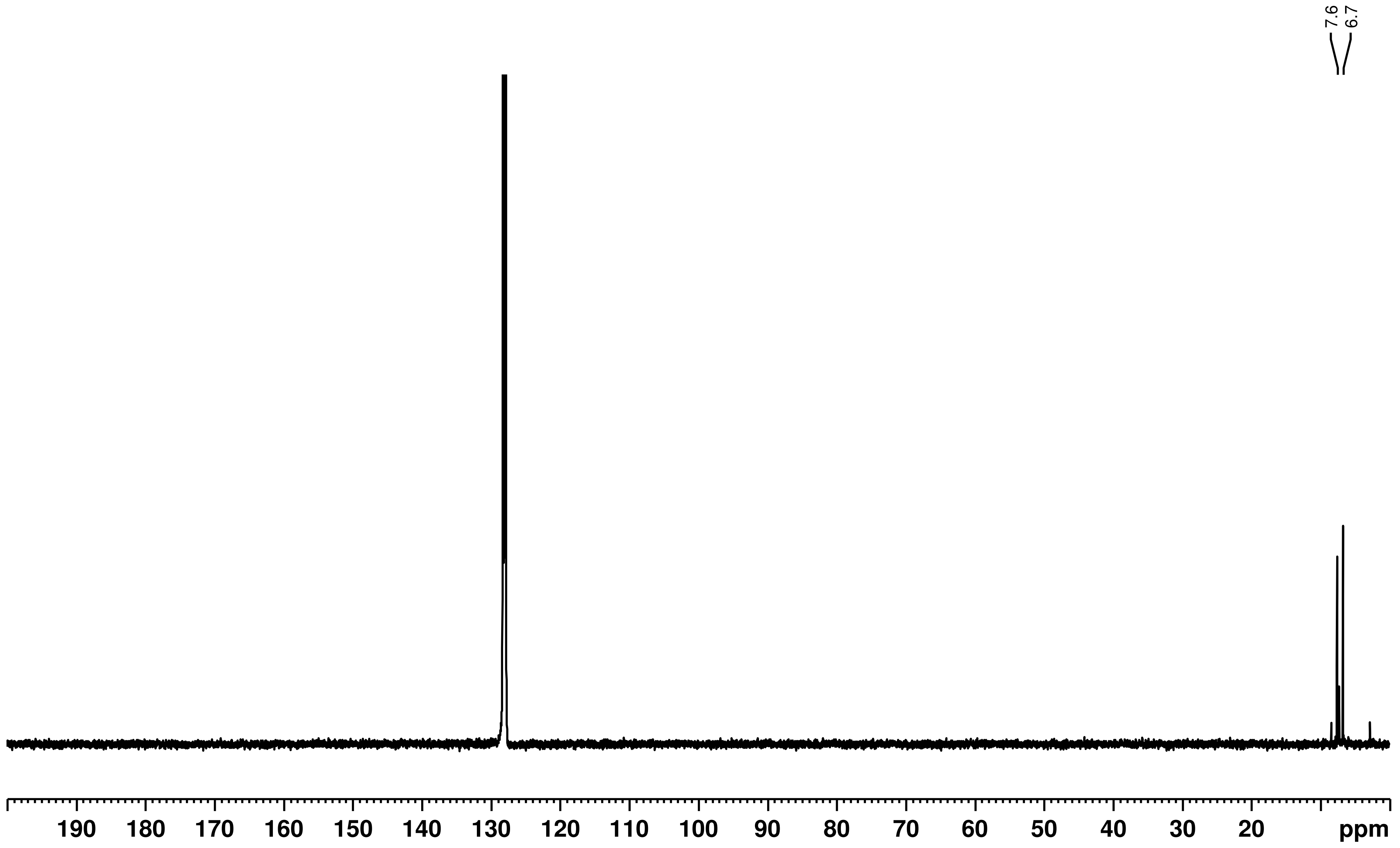

Supplementary Fig. 40. **^{29}Si{^{1}H} DEPT NMR** spectrum (99 MHz, C_6D_6, 298 K, optimized for $J_{H,Si}$ = 7 Hz, 18.4°) of chlorodiethylsilane (**6bb**)

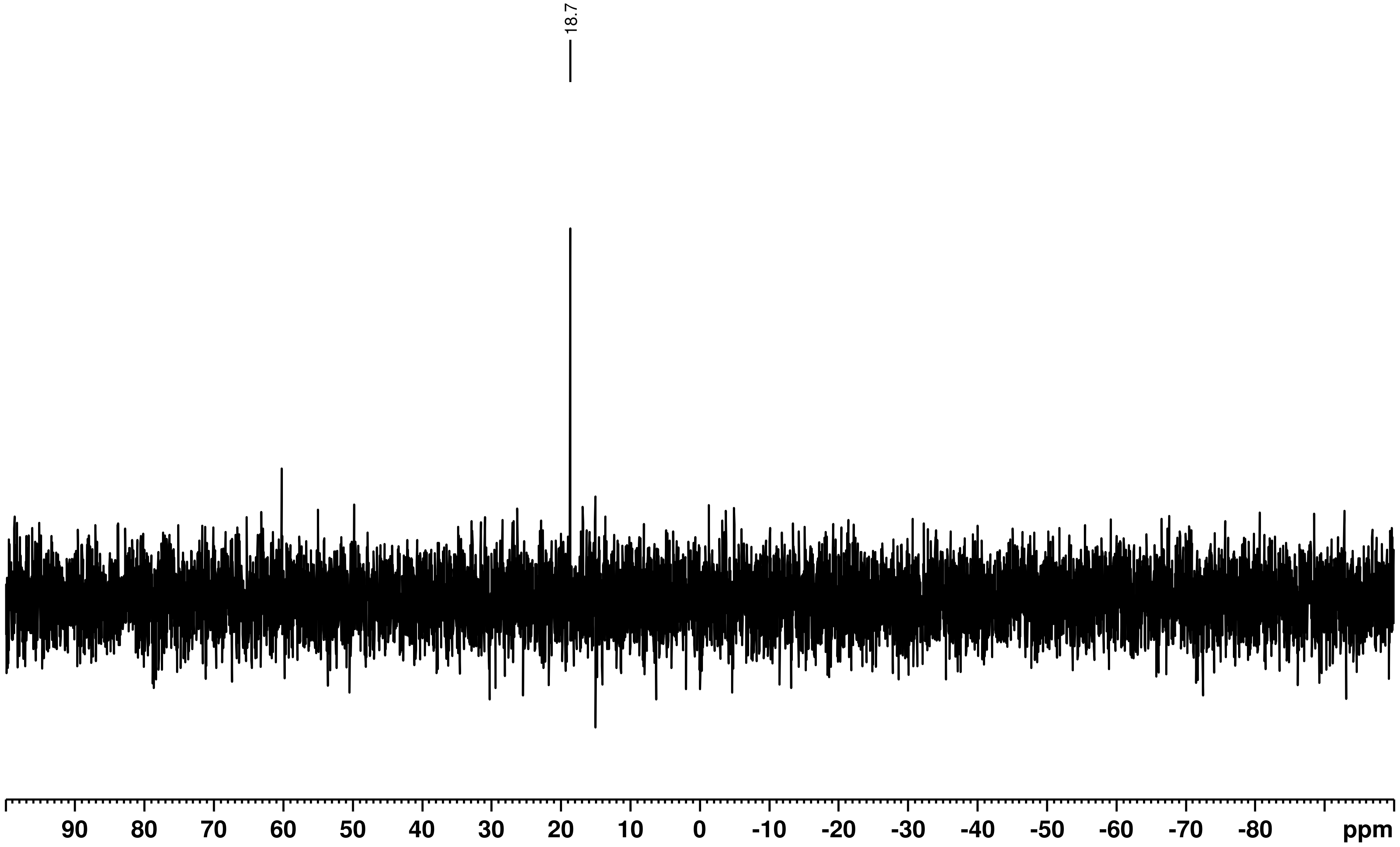

Supplementary Fig. 41. ^{1}**H NMR** spectrum (500 MHz, C_6D_6, 298 K) of bromodiethylsilane (**6cb**)

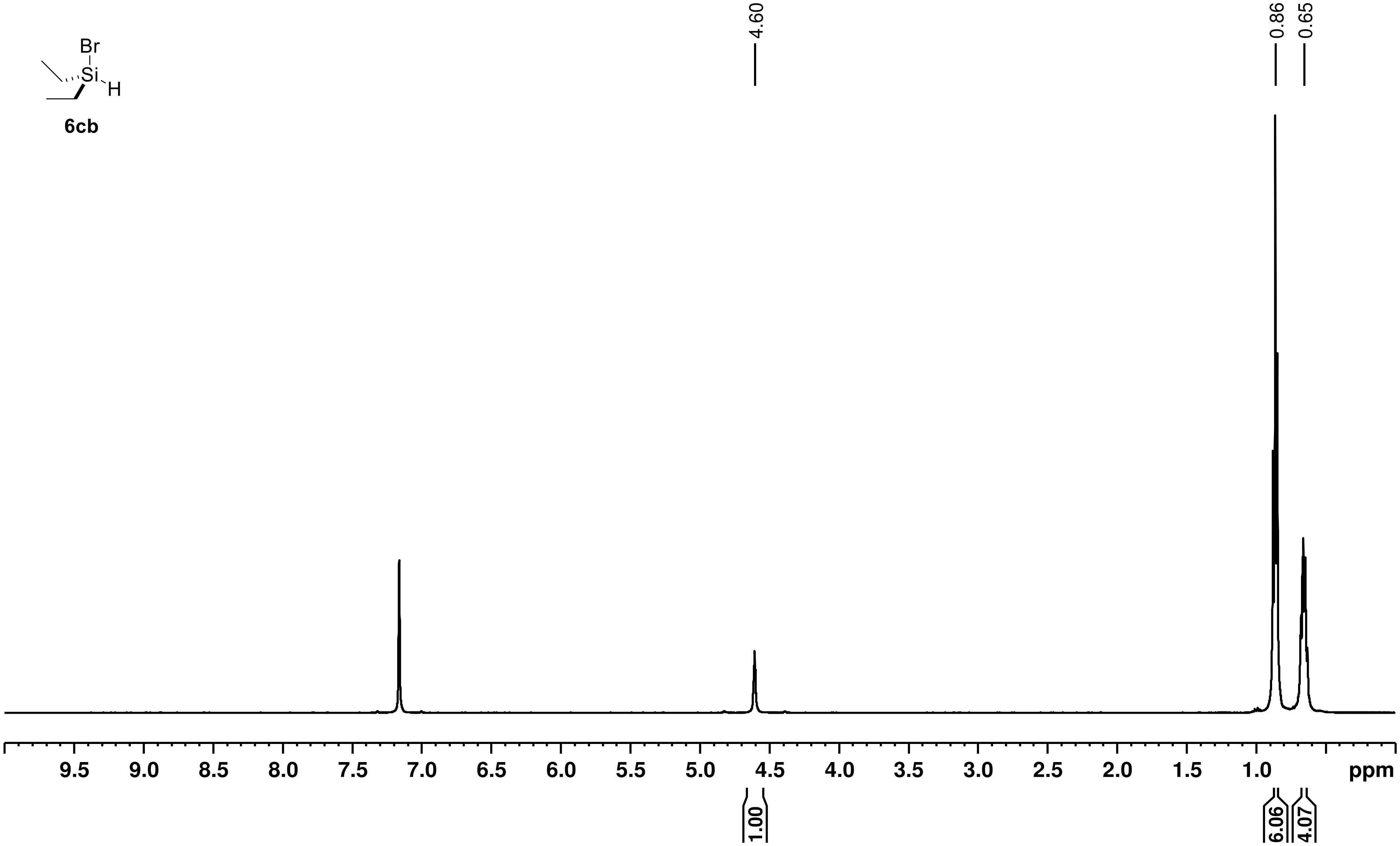

Supplementary Fig. 42. **^{13}C{^{1}H} NMR** spectrum (126 MHz, C_6D_6, 298 K) of bromodiethylsilane (**6cb**)

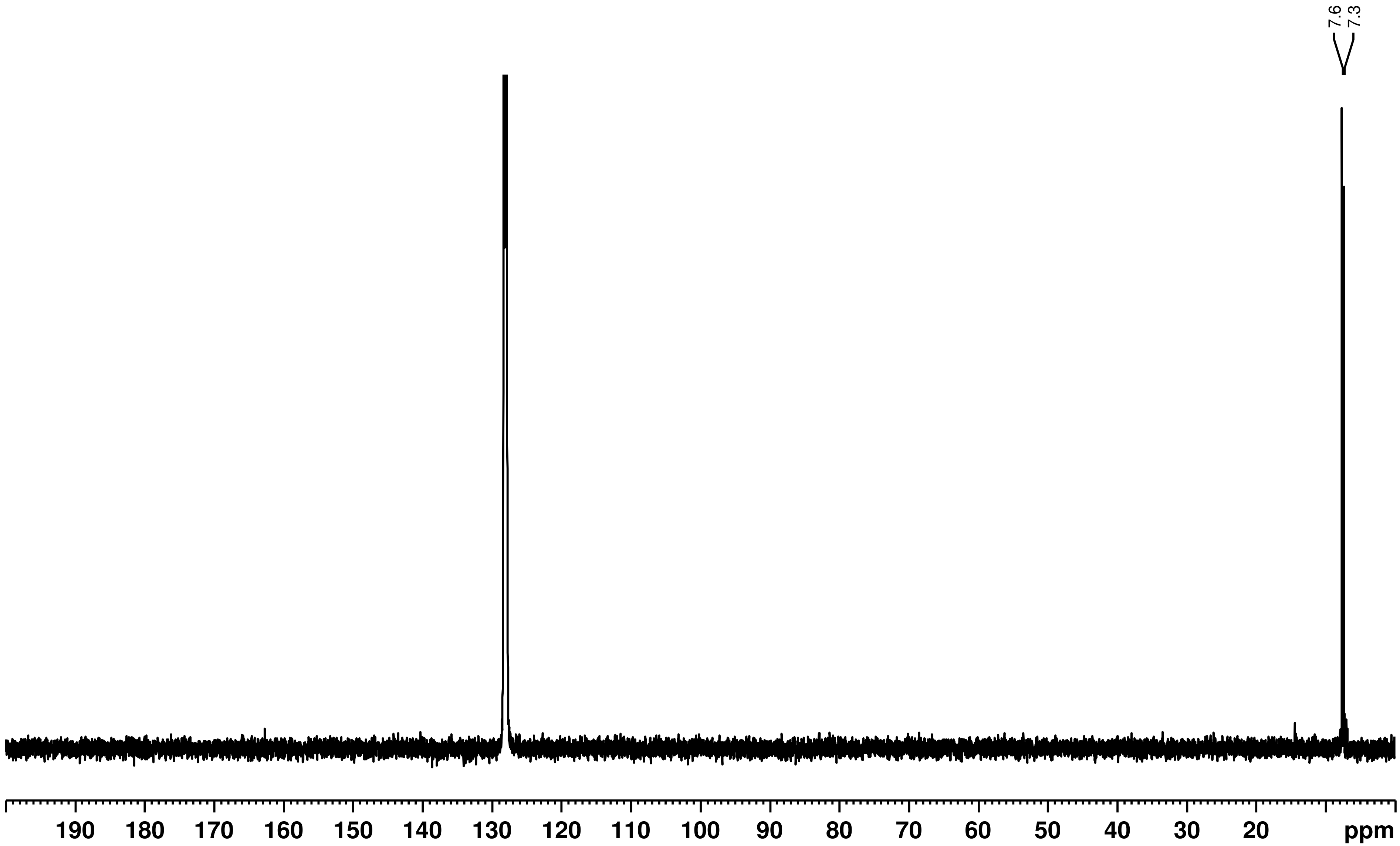

Supplementary Fig. 43. **^{29}Si{^{1}H} DEPT NMR** spectrum (99 MHz, C_6D_6, 298 K, optimized for $J_{H,Si}$ = 200 Hz, 90.0°) of bromodiethylsilane (**6cb**)

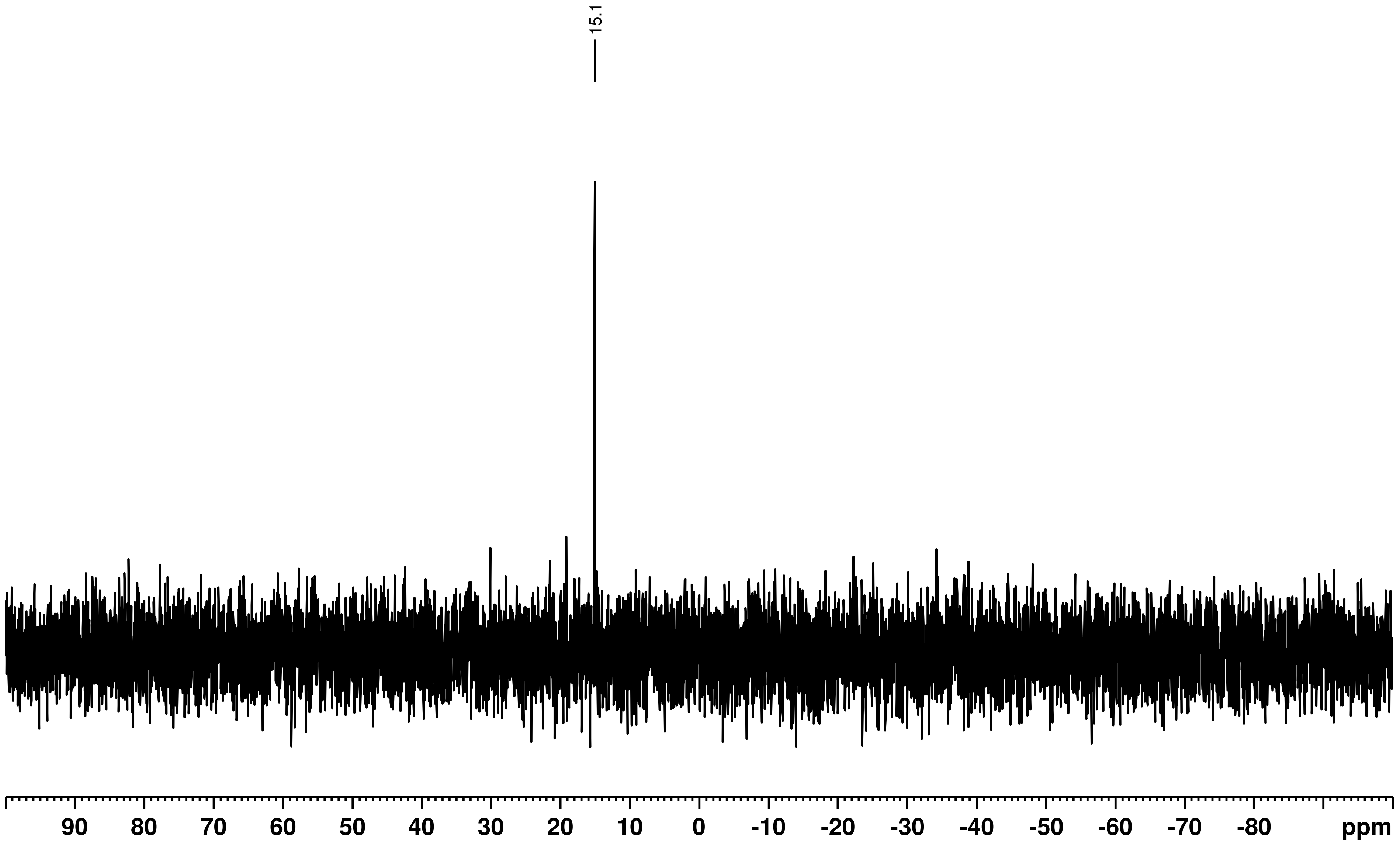

Supplementary Fig. 44. **IR** spectrum (ATR) of bromodiethylsilane (**6cb**)

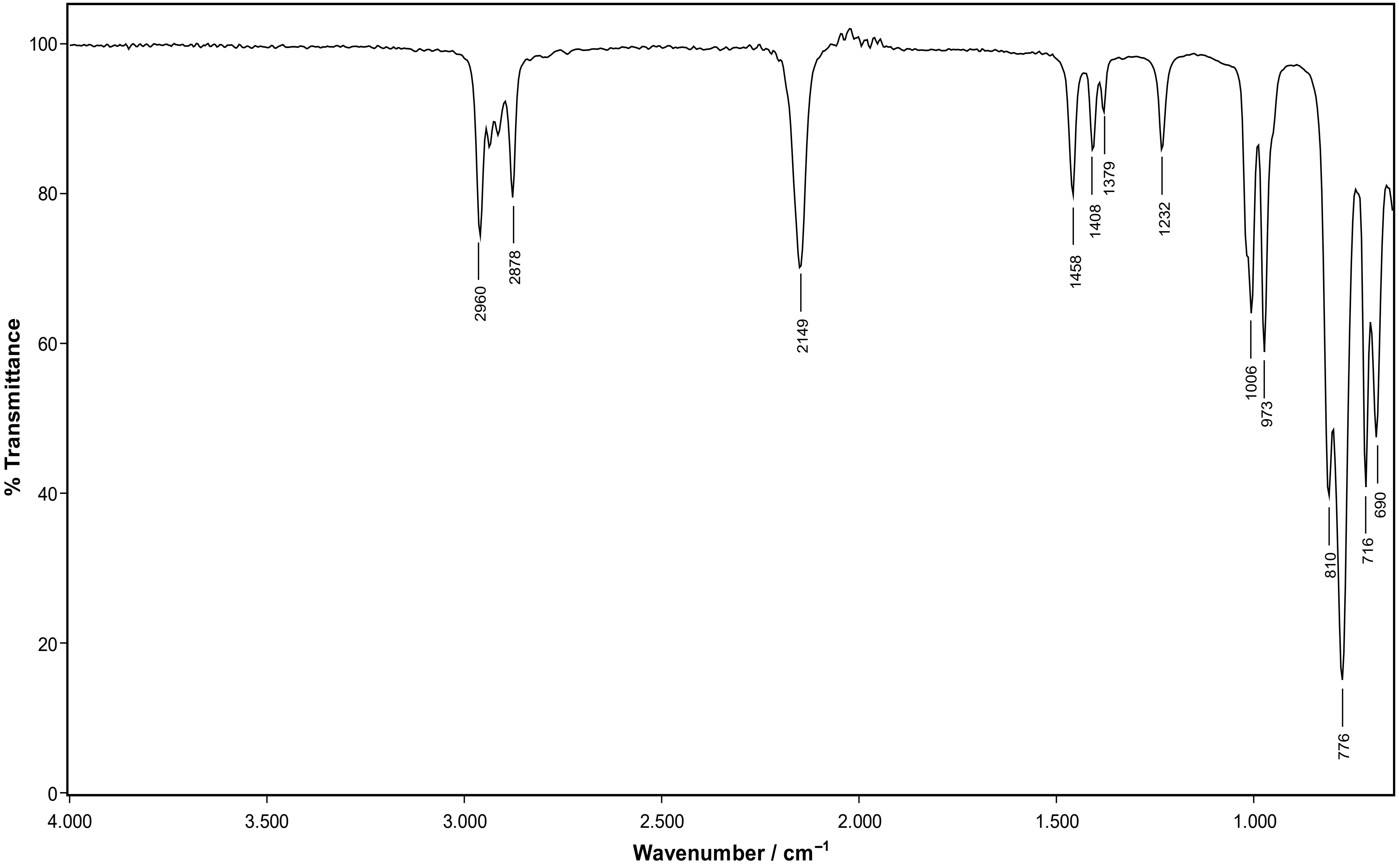

Supplementary Fig. 45. **^{1}H NMR** spectrum (500 MHz, C_6D_6, 298 K) of diethyliodosilane (**6db**)

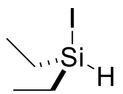

6db

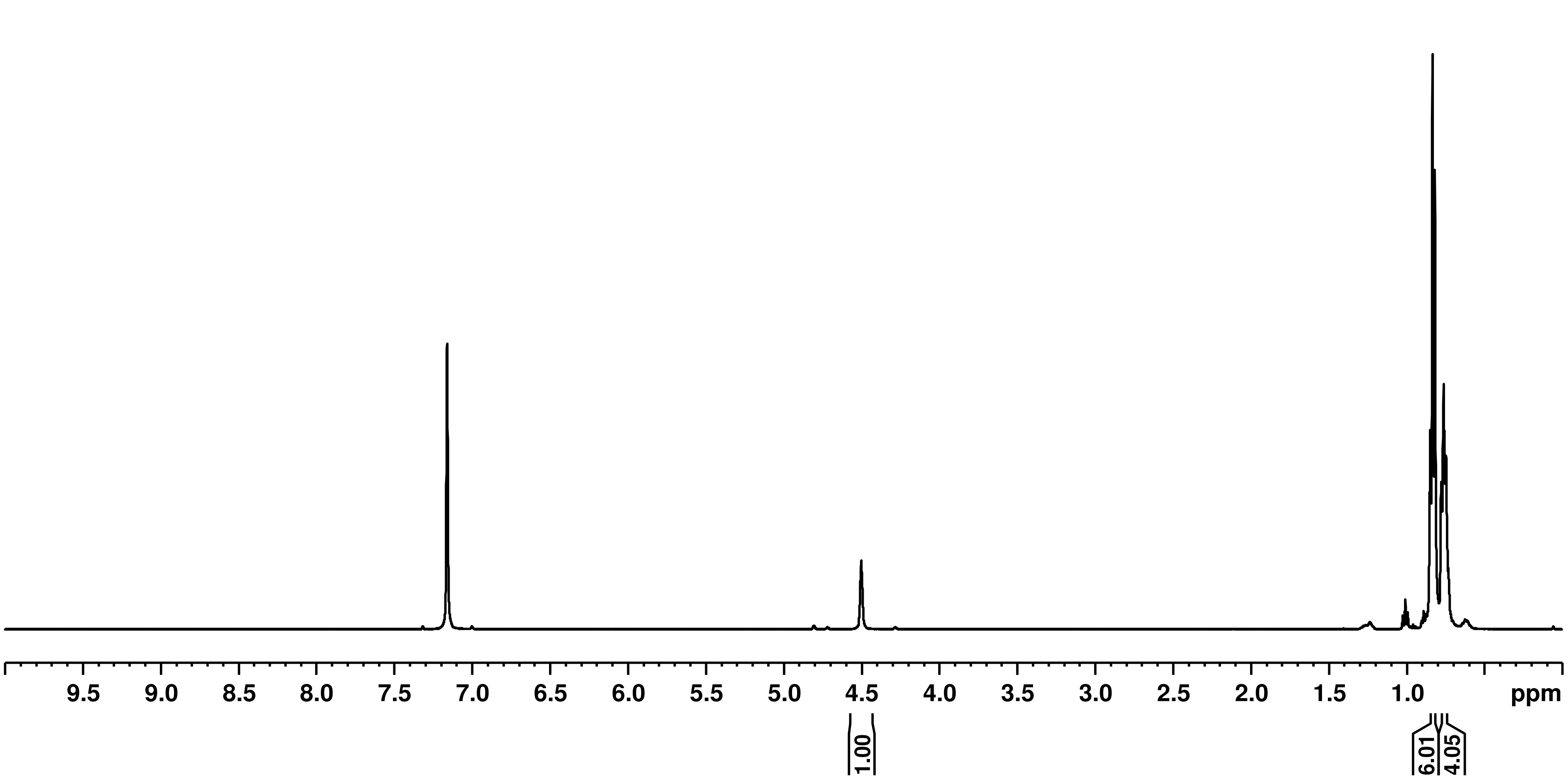

Supplementary Fig. 46. **^{13}C{^{1}H} NMR** spectrum (126 MHz, C_6D_6, 298 K) of diethyliodosilane (**6db**)

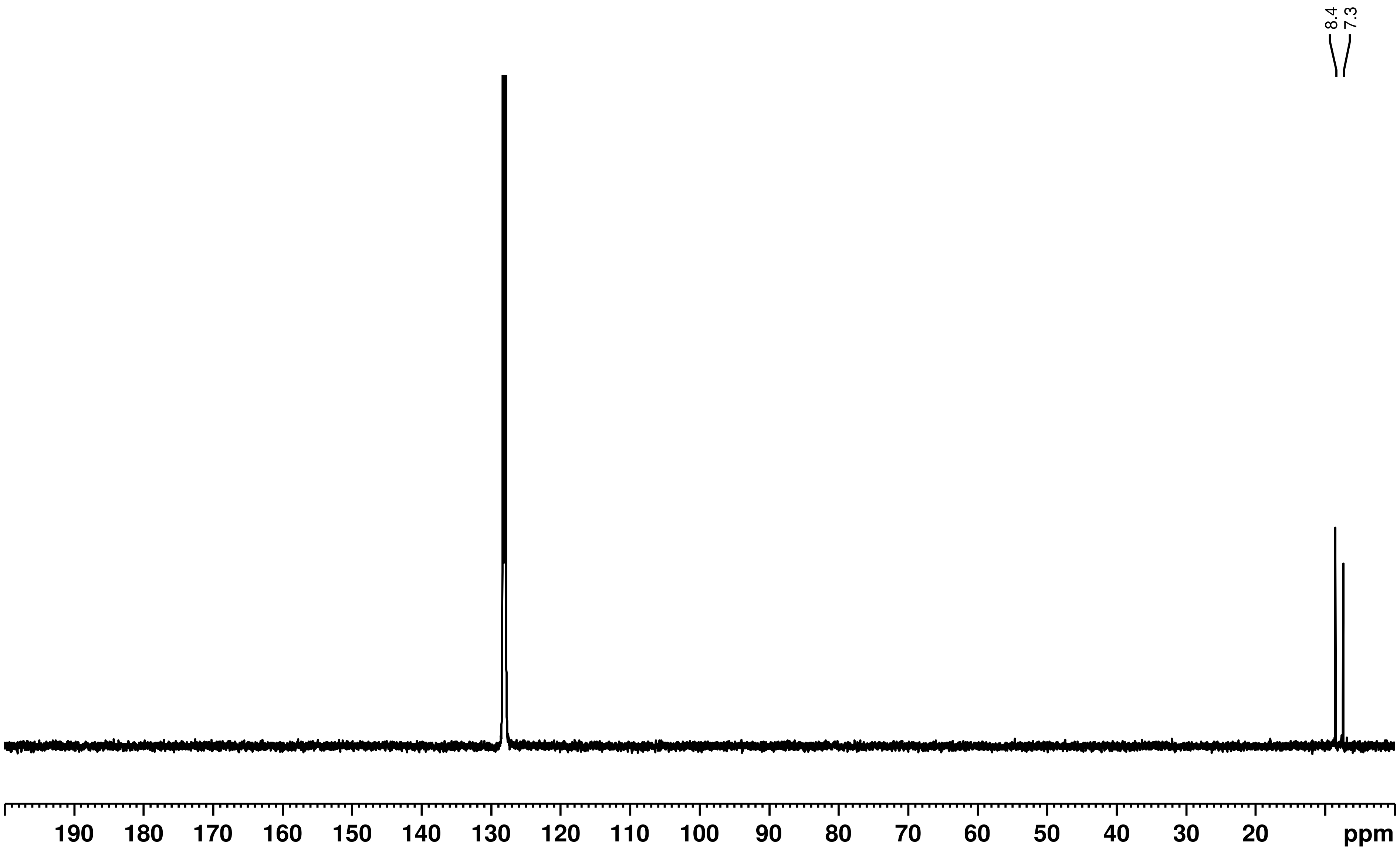

Supplementary Fig. 47. **$^{29}Si\{^{1}H\}$ DEPT NMR** spectrum (99 MHz, C_6D_6, 298 K, optimized for $J_{H,Si}$ = 7 Hz, 18.4°) of diethyliodosilane (**6db**)

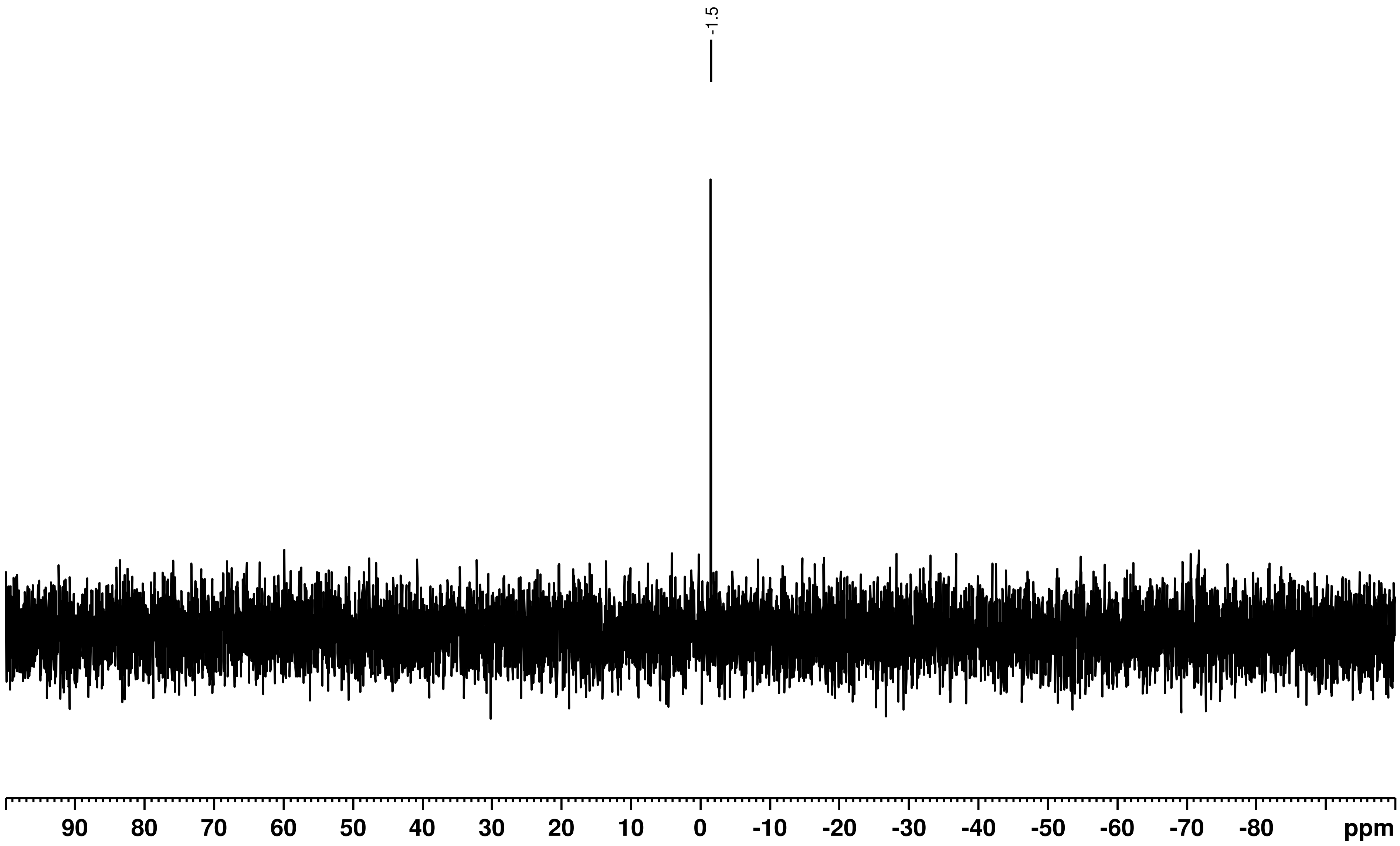

Supplementary Fig. 48. ^{1}**H NMR** spectrum (500 MHz, CD_2Cl_2, 298 K) of chlorodiisopropyl(phenyl)silane (**7bc**)

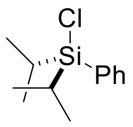

7bc

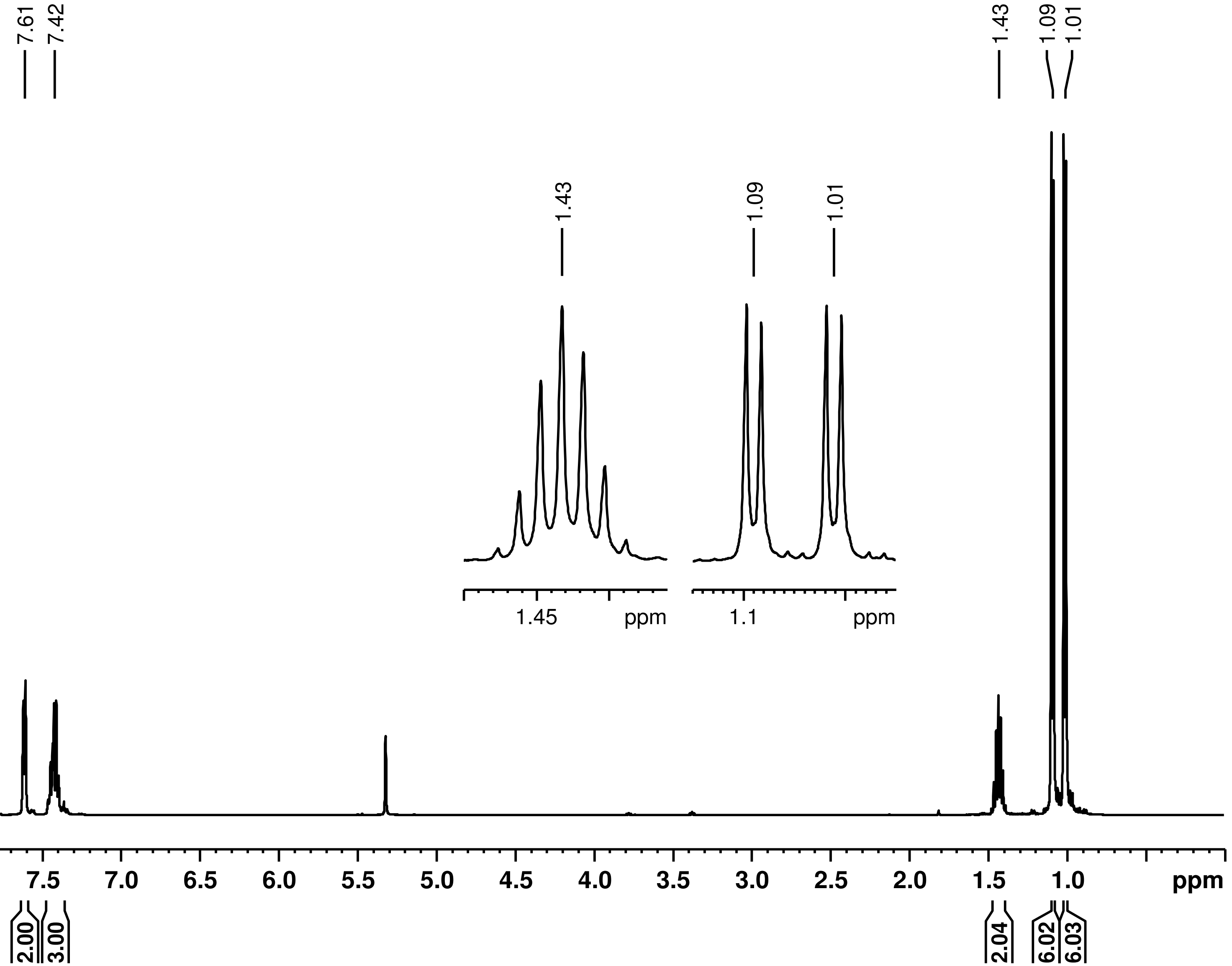

Supplementary Fig. 49. **^{13}C{^{1}H} NMR** spectrum (126 MHz, CD_2Cl_2, 298 K) of chlorodiisopropyl(phenyl)silane (**7bc**)

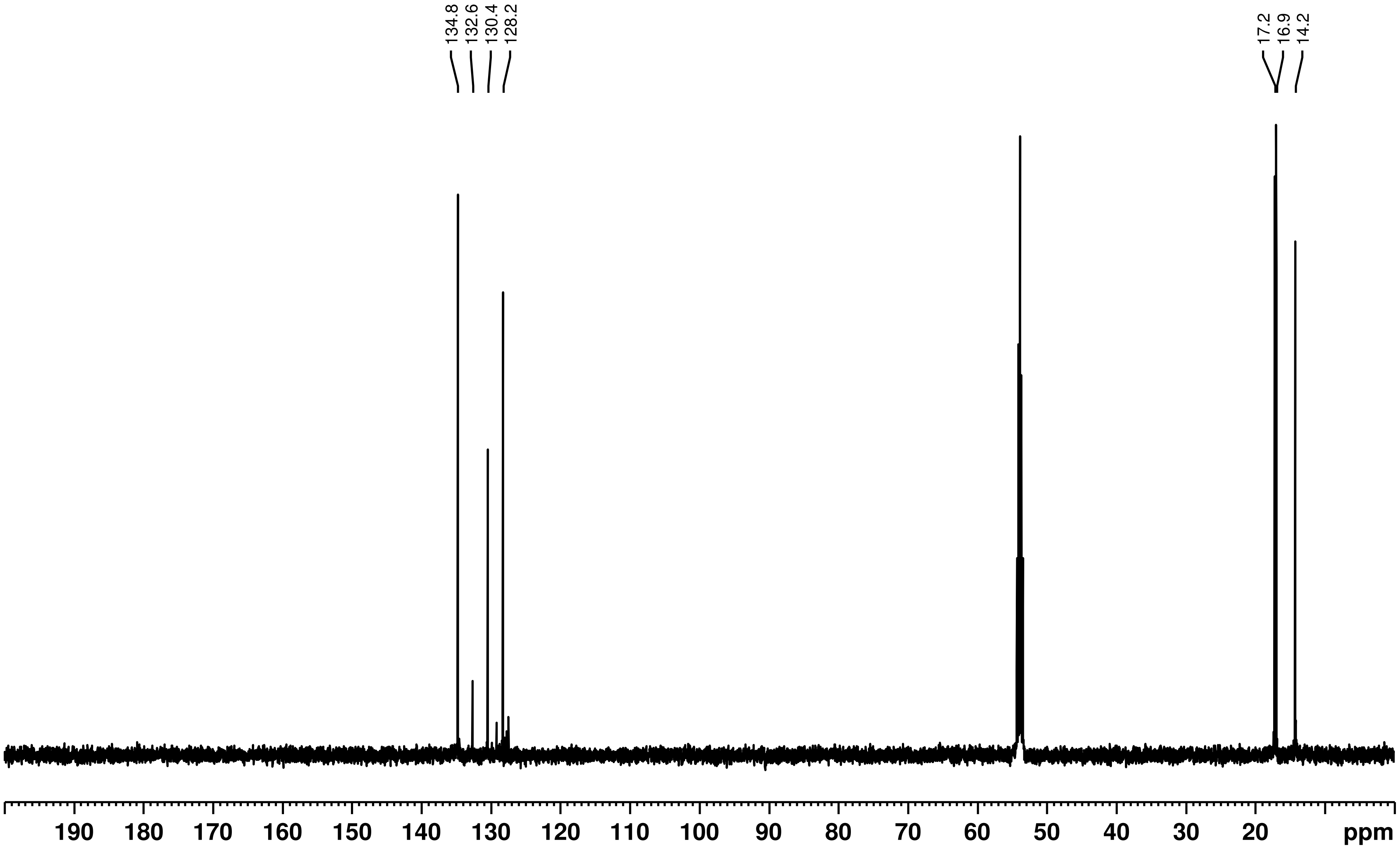

Supplementary Fig. 50. **^{29}Si{^{1}H} DEPT NMR** spectrum (99 MHz, CD_2Cl_2, 298 K, optimized for $J_{H,Si}$ = 7 Hz, 14.5°) of chlorodiisopropyl(phenyl)silane (**7bc**)

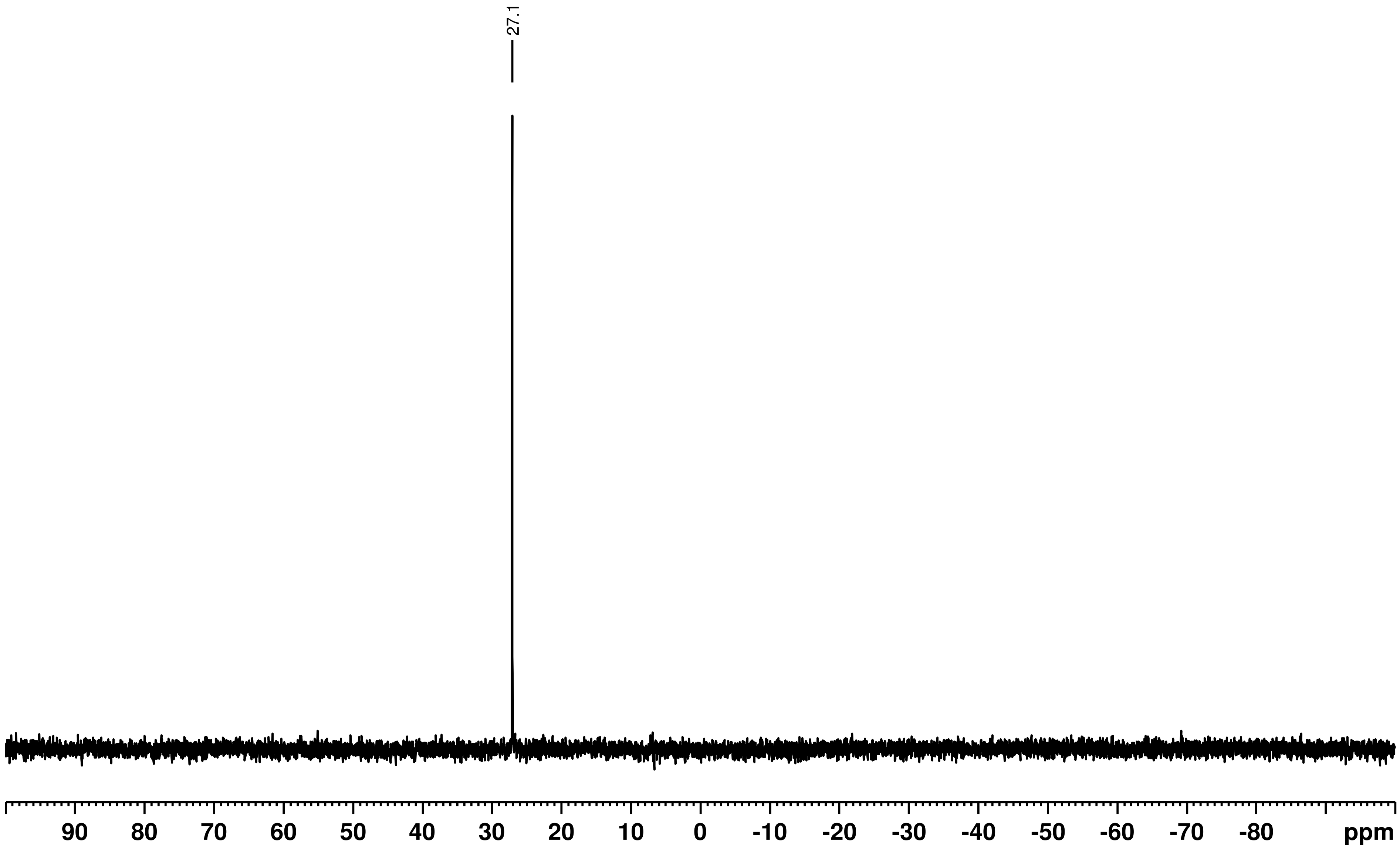

Supplementary Fig. 51. **IR** spectrum (ATR) of chlorodiisopropyl(phenyl)silane (**7bc**)

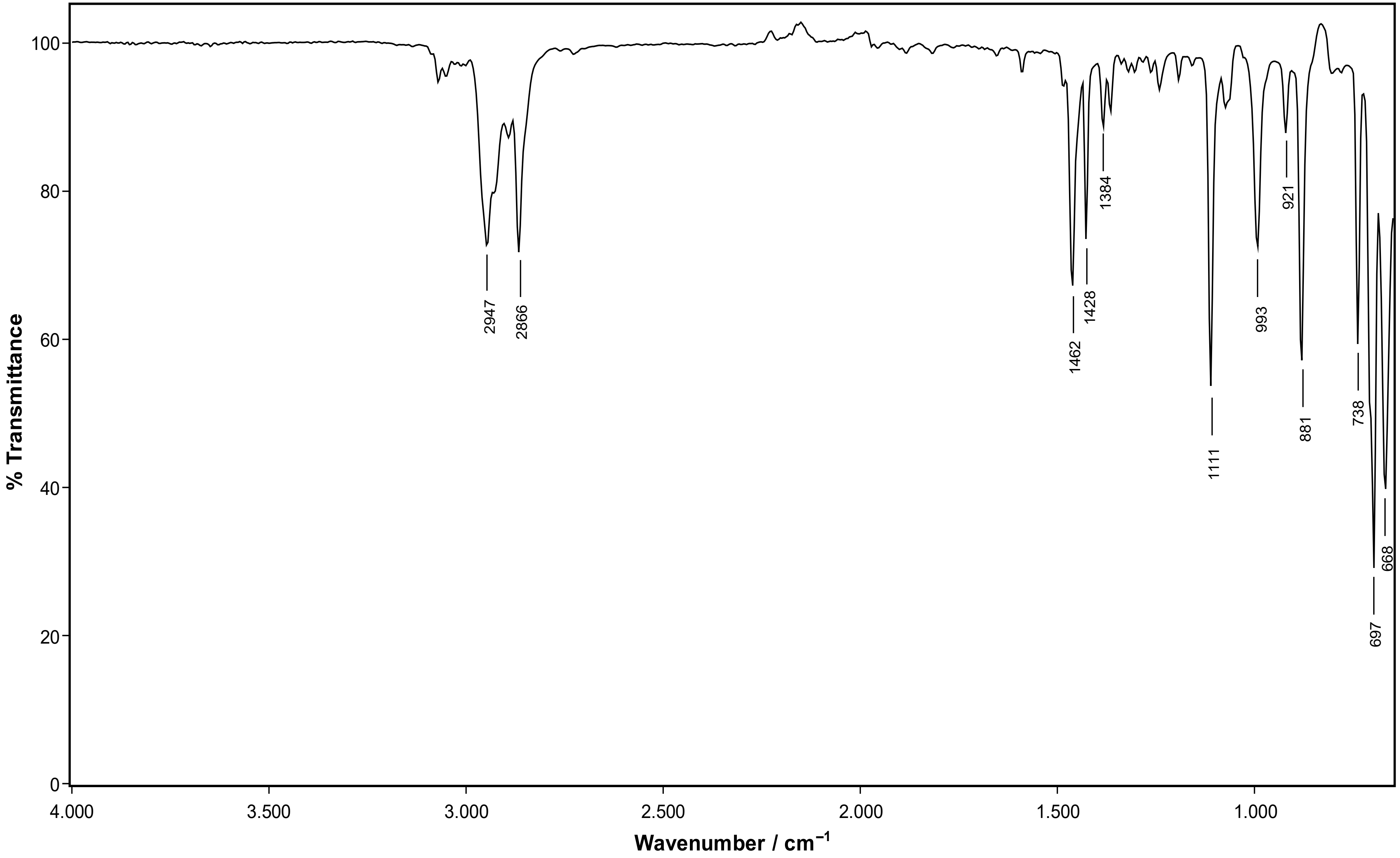

Supplementary Fig. 52. ^{1}**H NMR** spectrum (500 MHz, CD_2Cl_2, 298 K) of fluorodiisopropyl(phenyl)silane (**7ac**)

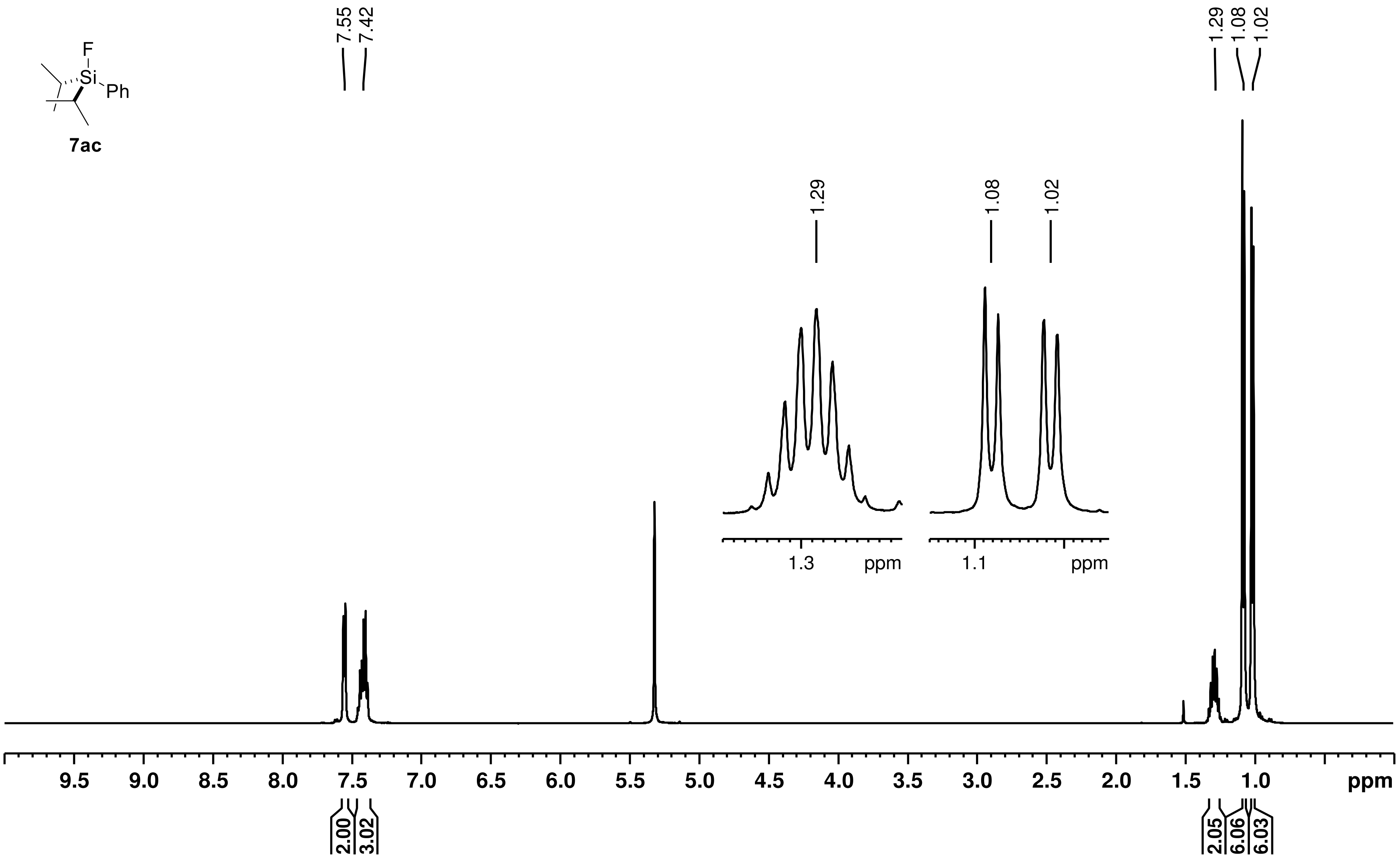

Supplementary Fig. 53. **^{13}C{^{1}H} NMR** spectrum (126 MHz, CD_2Cl_2, 298 K) of fluorodiisopropyl(phenyl)silane (**7ac**)

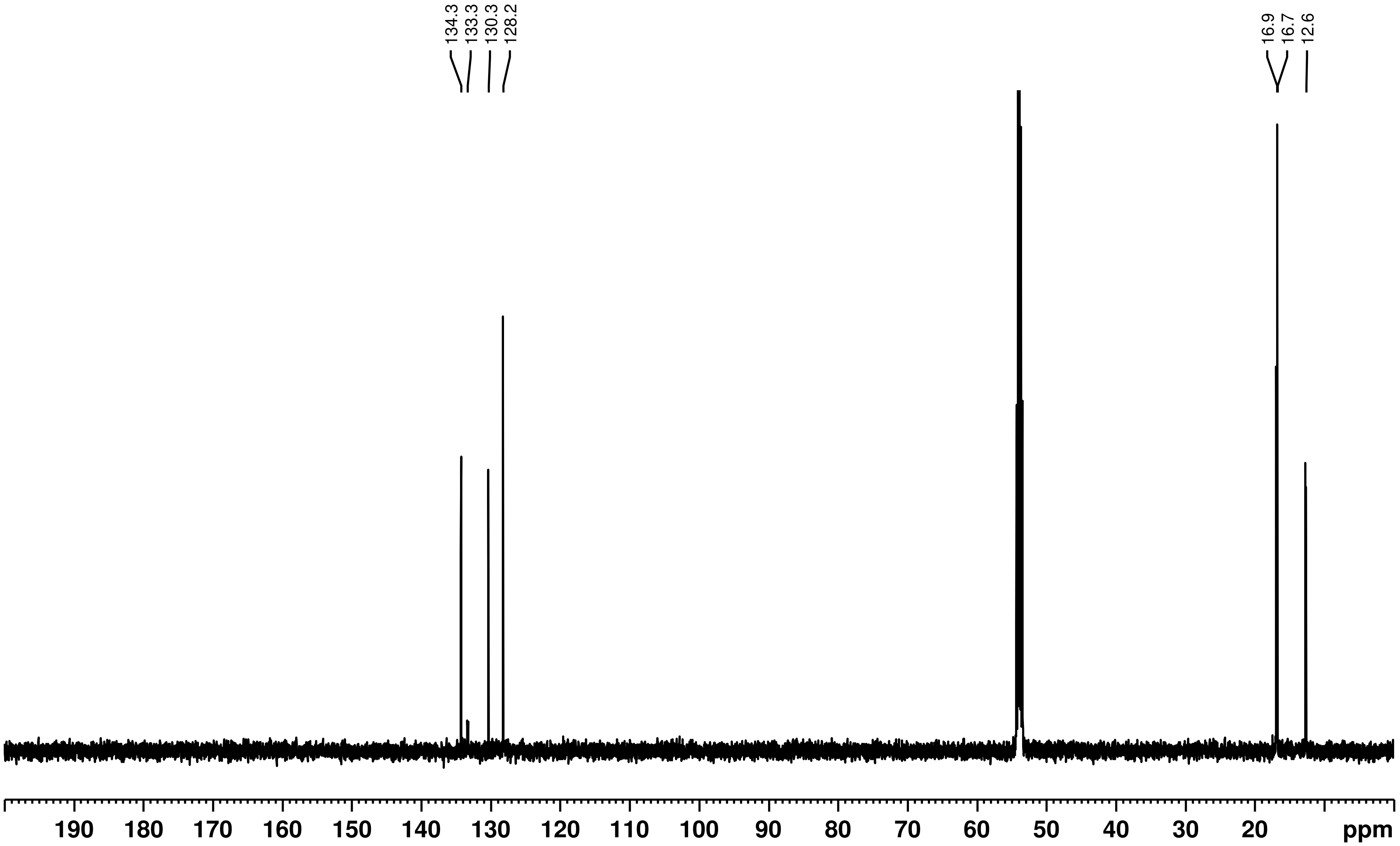

Supplementary Fig. 54. ^{19}**F NMR** spectrum (471 MHz, CD_2Cl_2, 298 K) of fluorodiisopropyl(phenyl)silane (**7ac**)

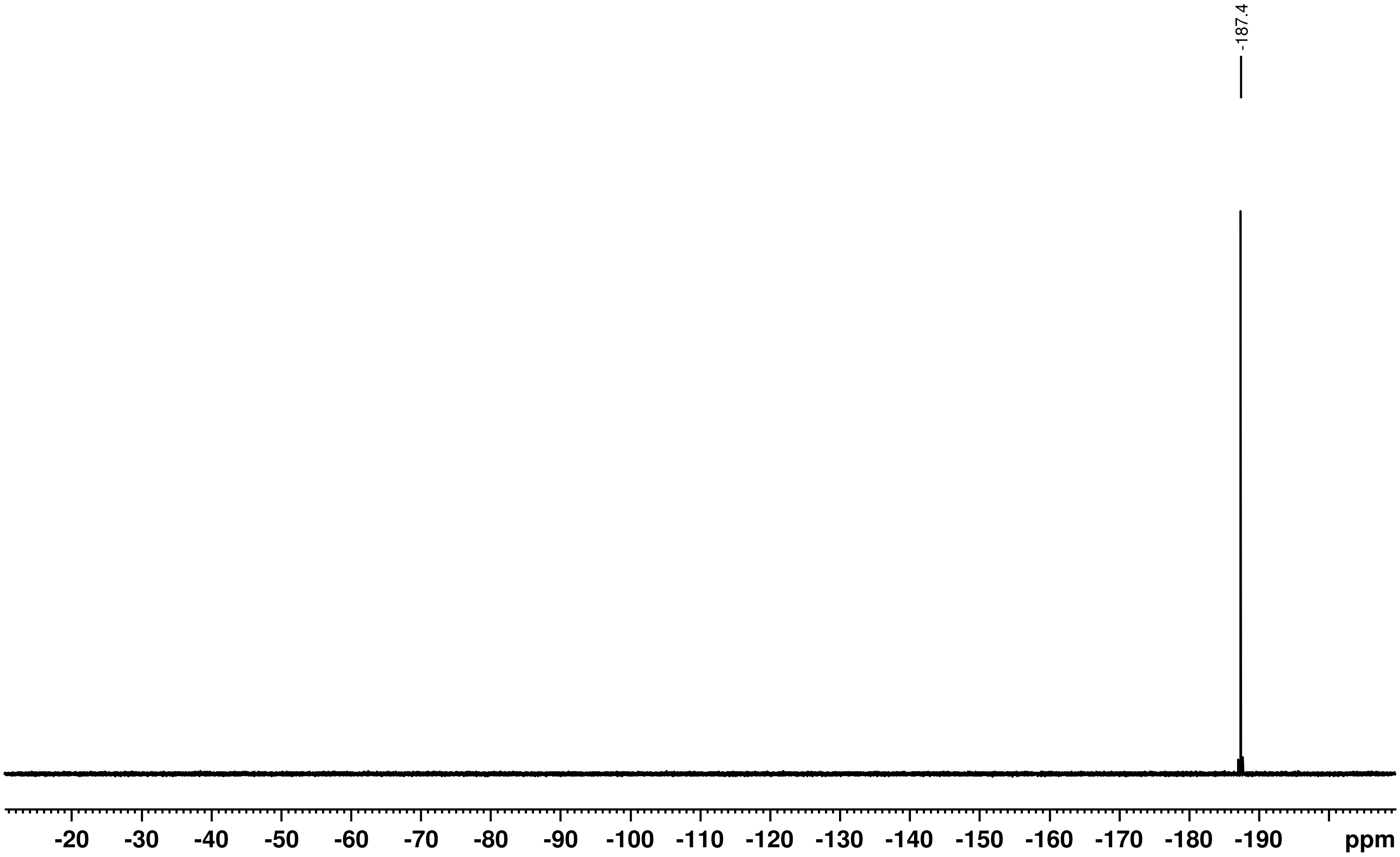

Supplementary Fig. 55. **^{29}Si{^{1}H} DEPT NMR** spectrum (99 MHz, CD_2Cl_2, 298 K, optimized for $J_{H,Si}$ = 7 Hz, 14.5°) of fluorodiisopropyl(phenyl)silane (**7ac**)

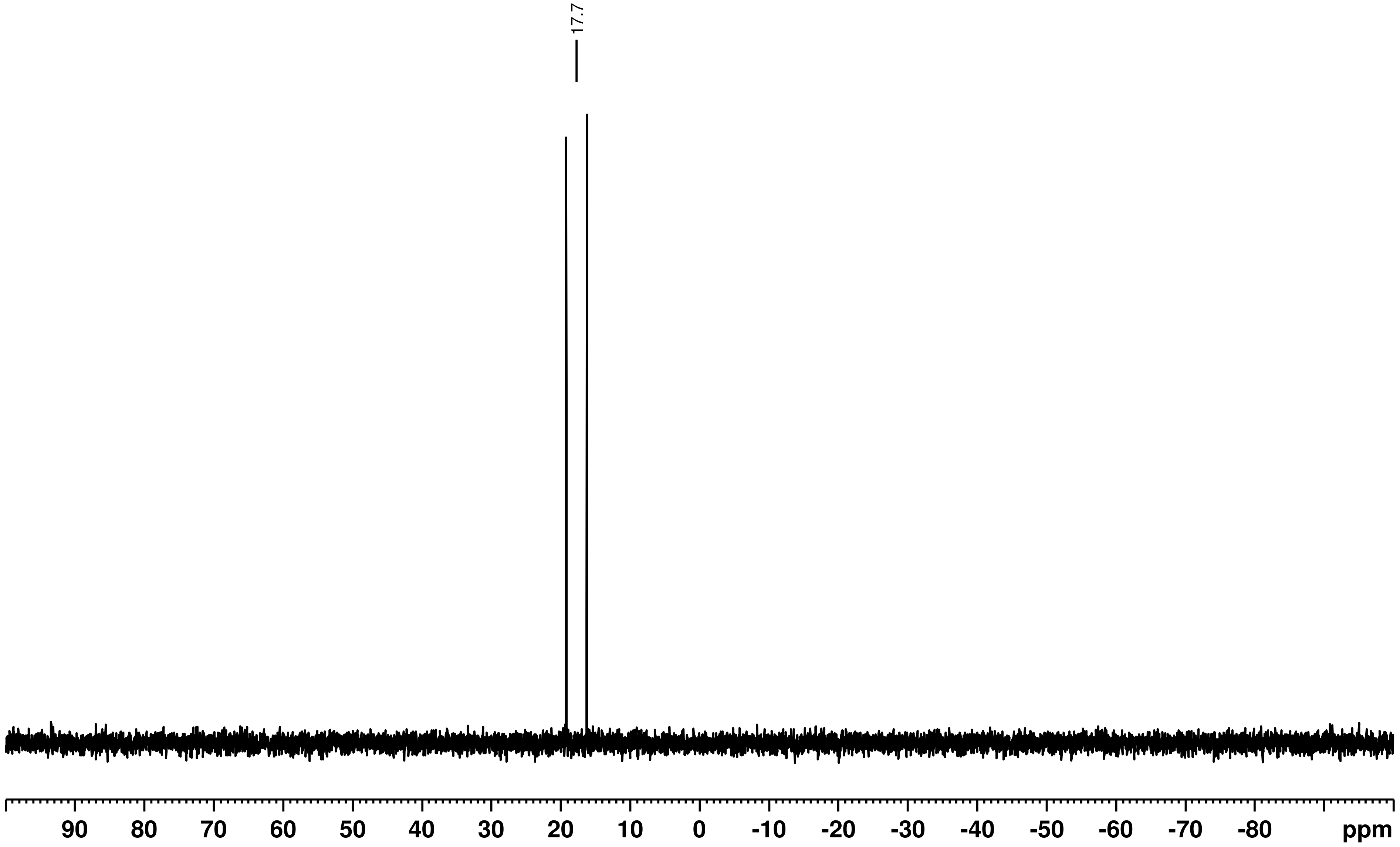

Supplementary Fig. 56. **IR** spectrum (ATR) of fluorodiisopropyl(phenyl)silane (**7ac**)

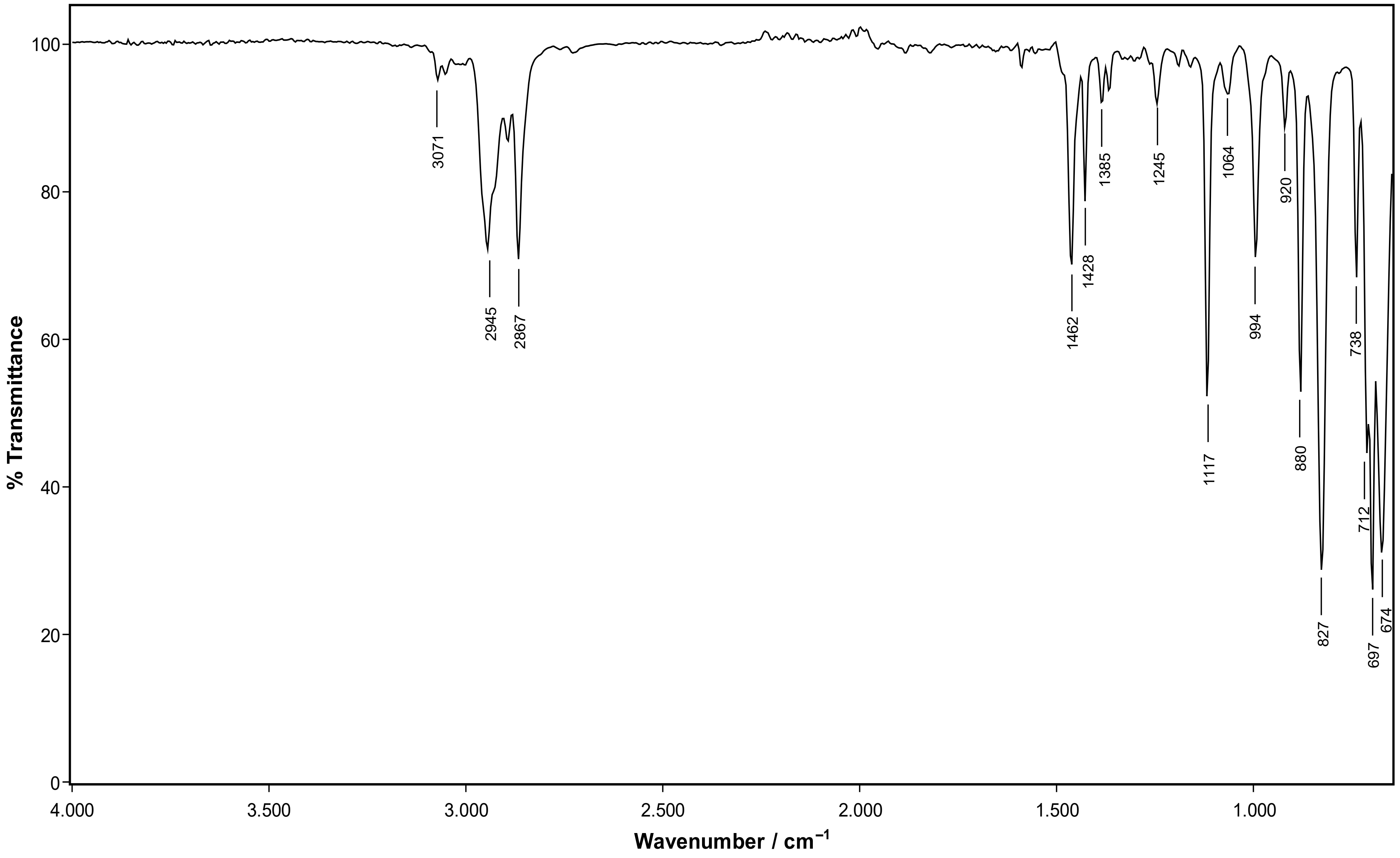

Supplementary Fig. 57. **GLC-MS** spectrum (EI) of fluorodiisopropyl(phenyl)silane (**7ac**)

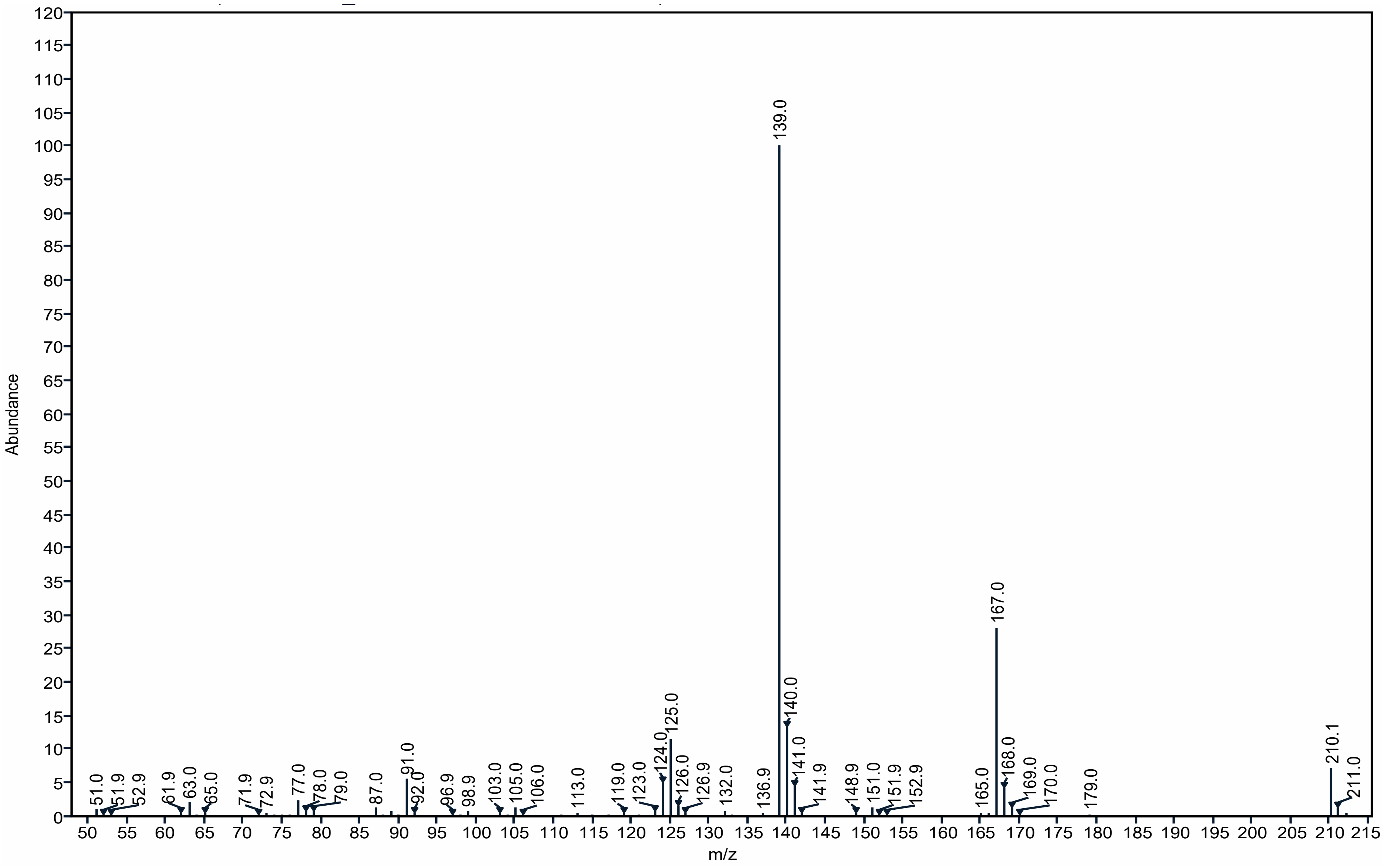

Supplementary Fig. 58. ^{1}**H NMR** spectrum (500 MHz, C_6D_6, 298 K) of chlorodiisopropylsilane (**6bc**)

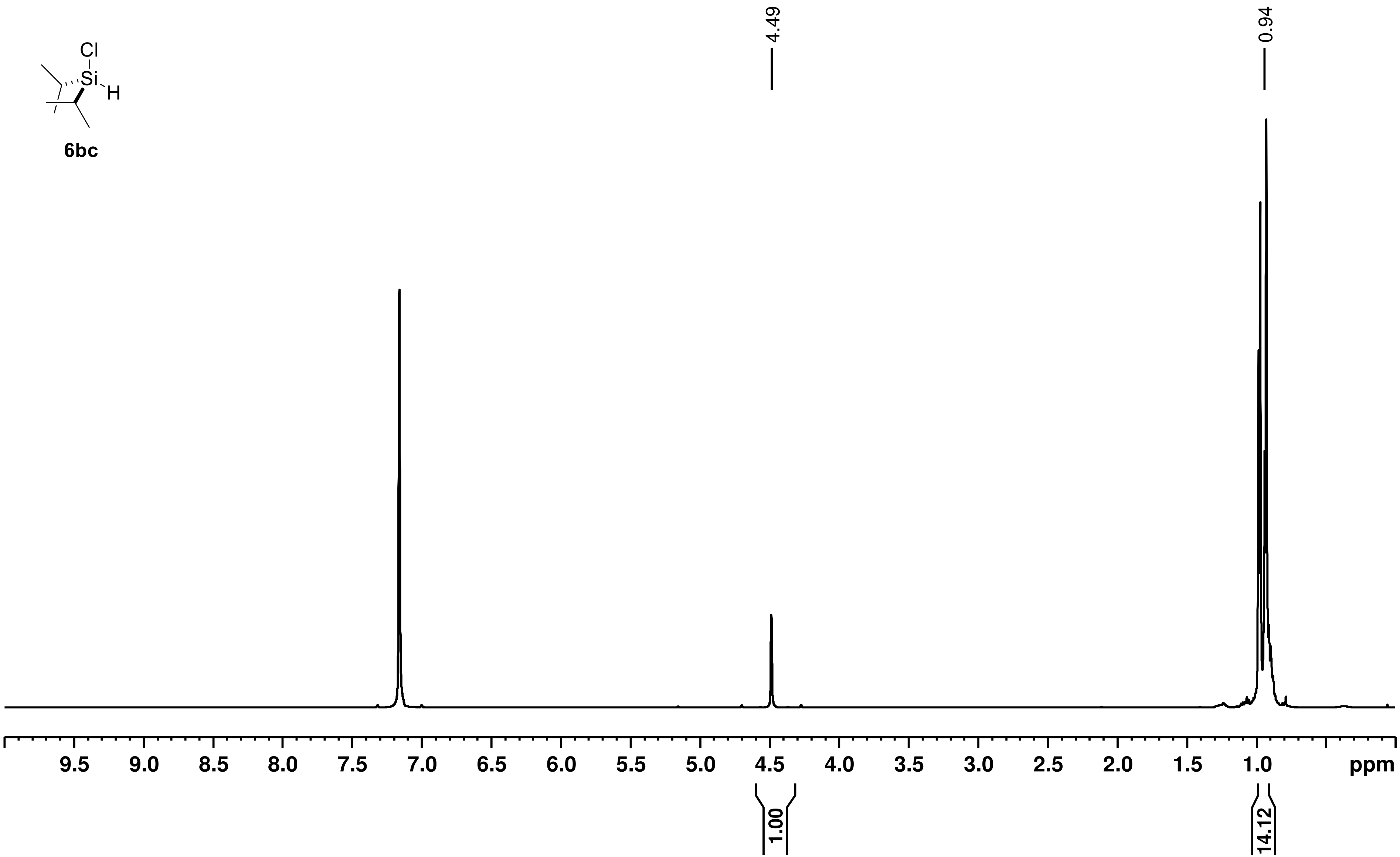

Supplementary Fig. 59. **^{13}C{^{1}H} NMR** spectrum (126 MHz, C_6D_6, 298 K) of chlorodiisopropylsilane (**6bc**)

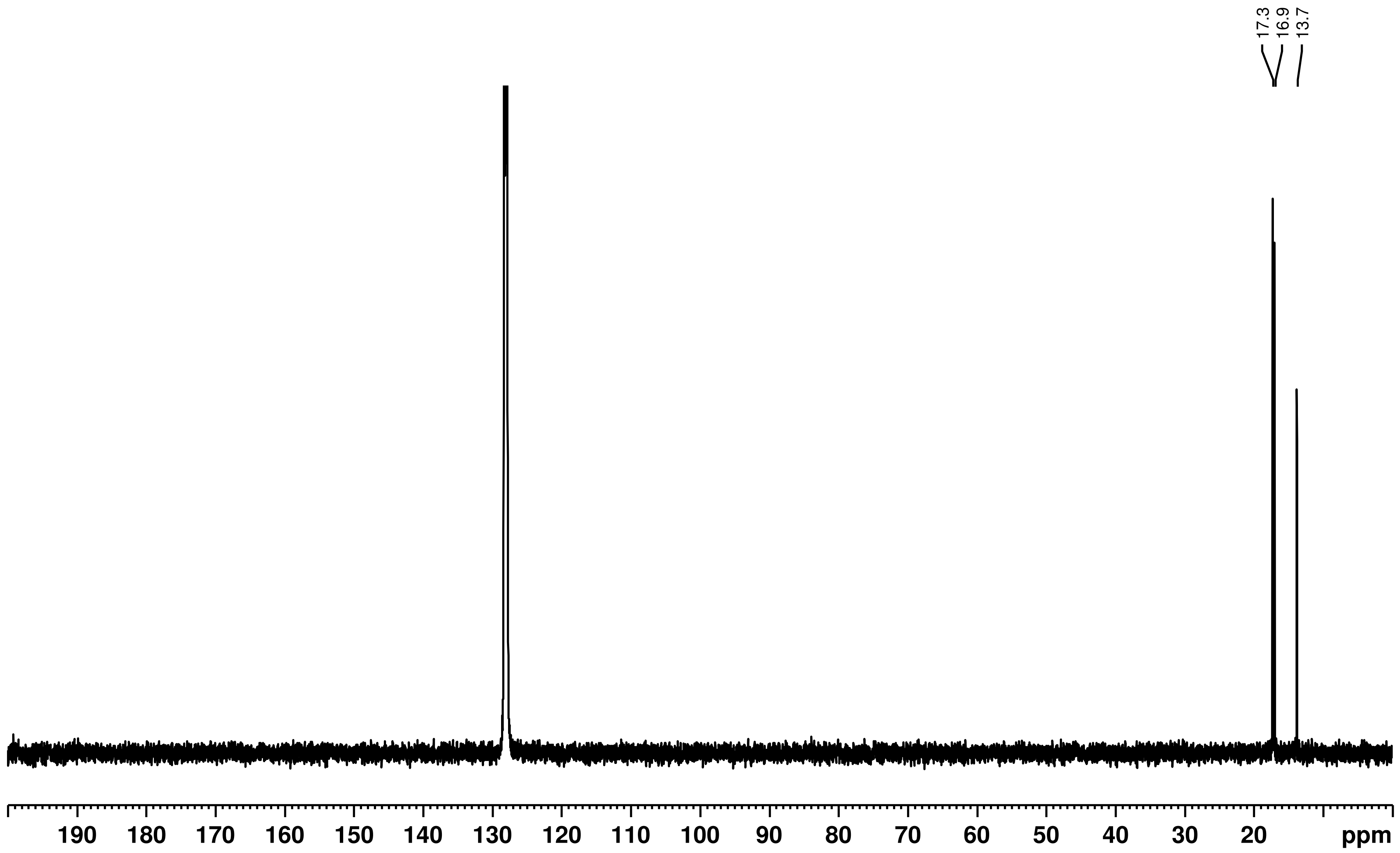

Supplementary Fig. 60. **^{29}Si{^{1}H} DEPT NMR** spectrum (99 MHz, C_6D_6, 298 K, optimized for $J_{H,Si}$ = 200 Hz, 90.0°) of chlorodiisopropylsilane (**6bc**)

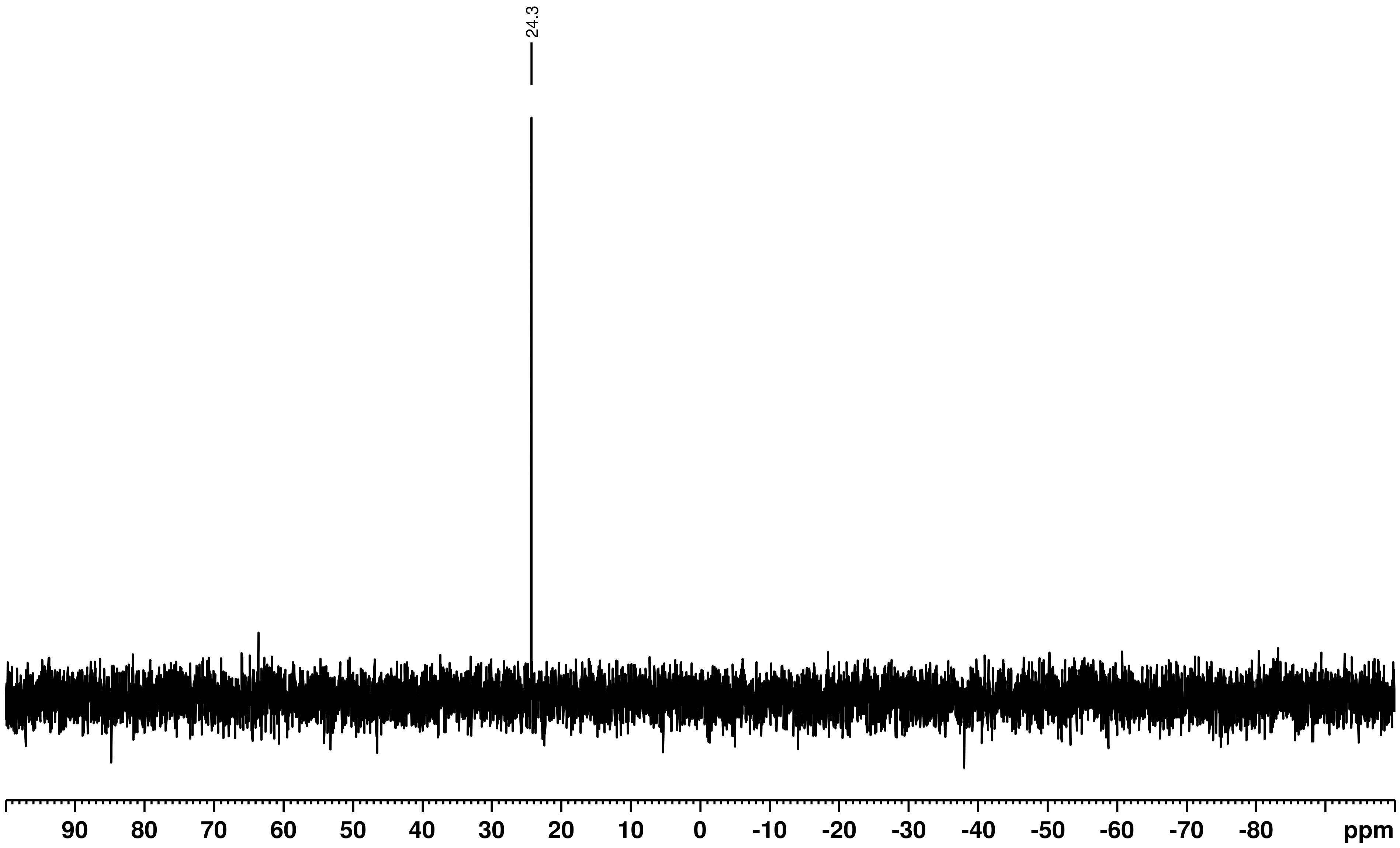

Supplementary Fig. 61. **IR** spectrum (ATR) of chlorodiisopropylsilane (**6bc**)

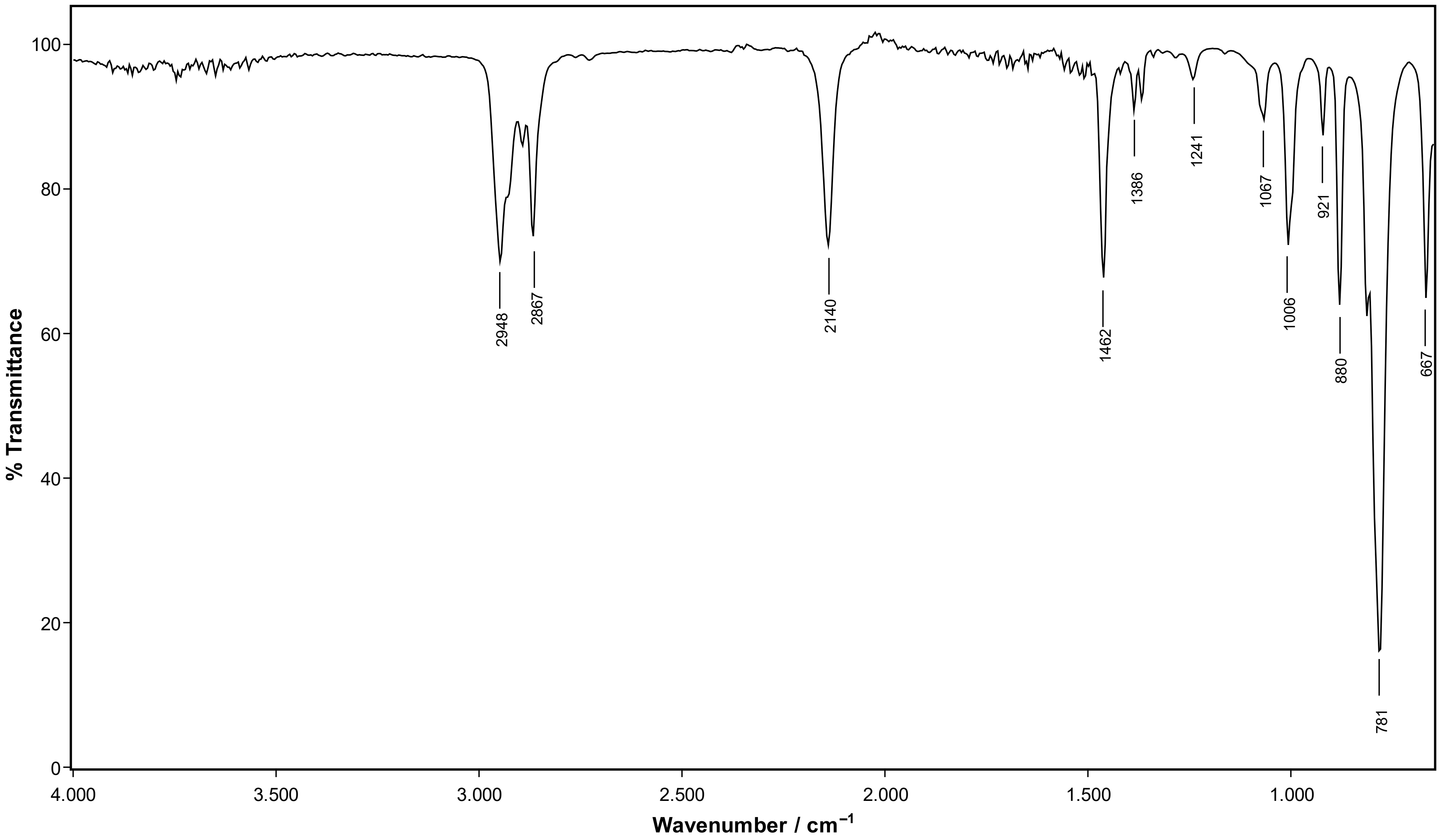

Supplementary Fig. 62. ^{1}**H NMR** spectrum (700 MHz, C_6D_6, 298 K) of fluorodiisopropylsilane (**6ac**)

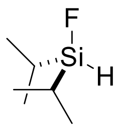

6ac

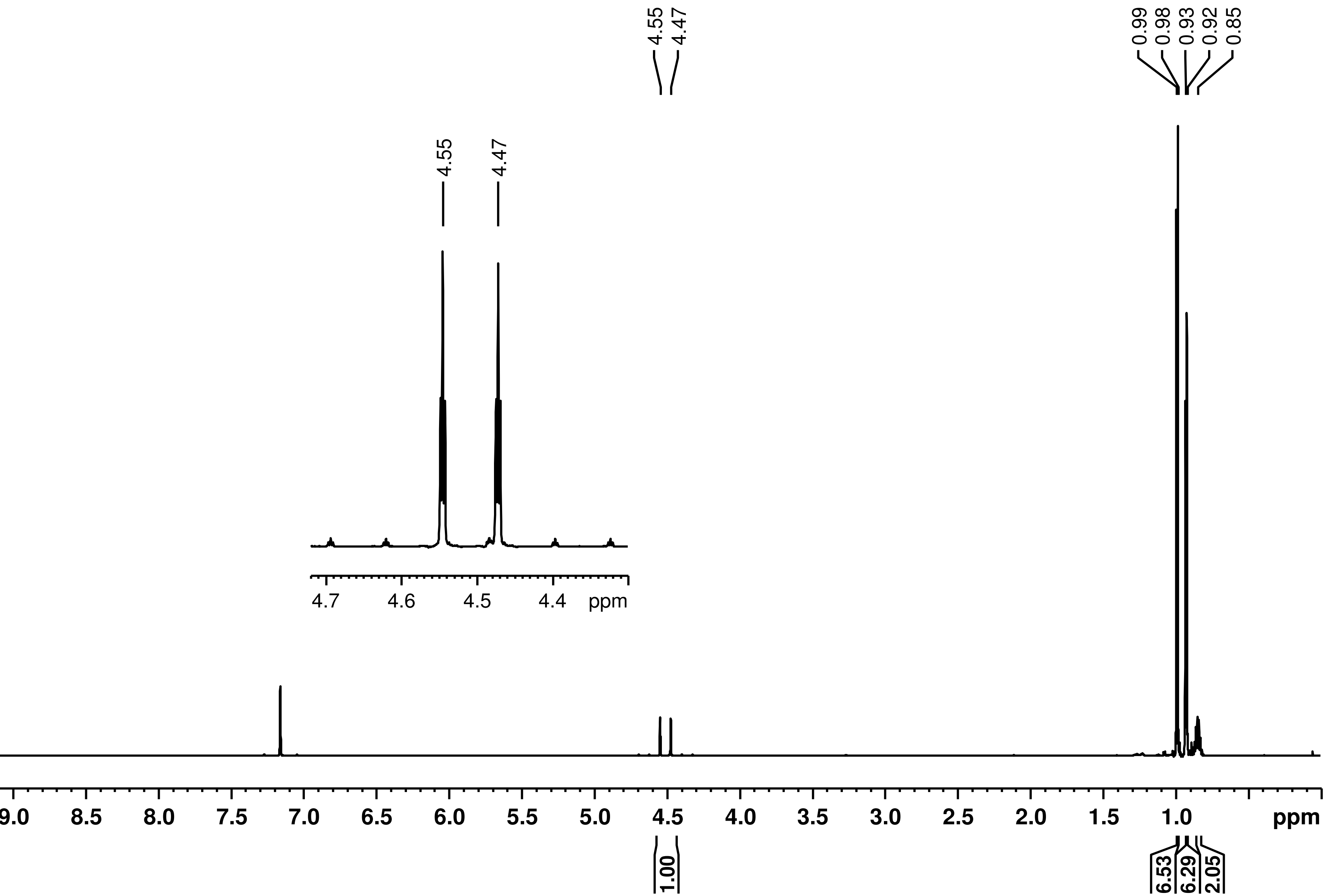

Supplementary Fig. 63. **^{13}C{^{1}H} NMR** spectrum (126 MHz, C_6D_6, 298 K) of fluorodiisopropylsilane (**6ac**)

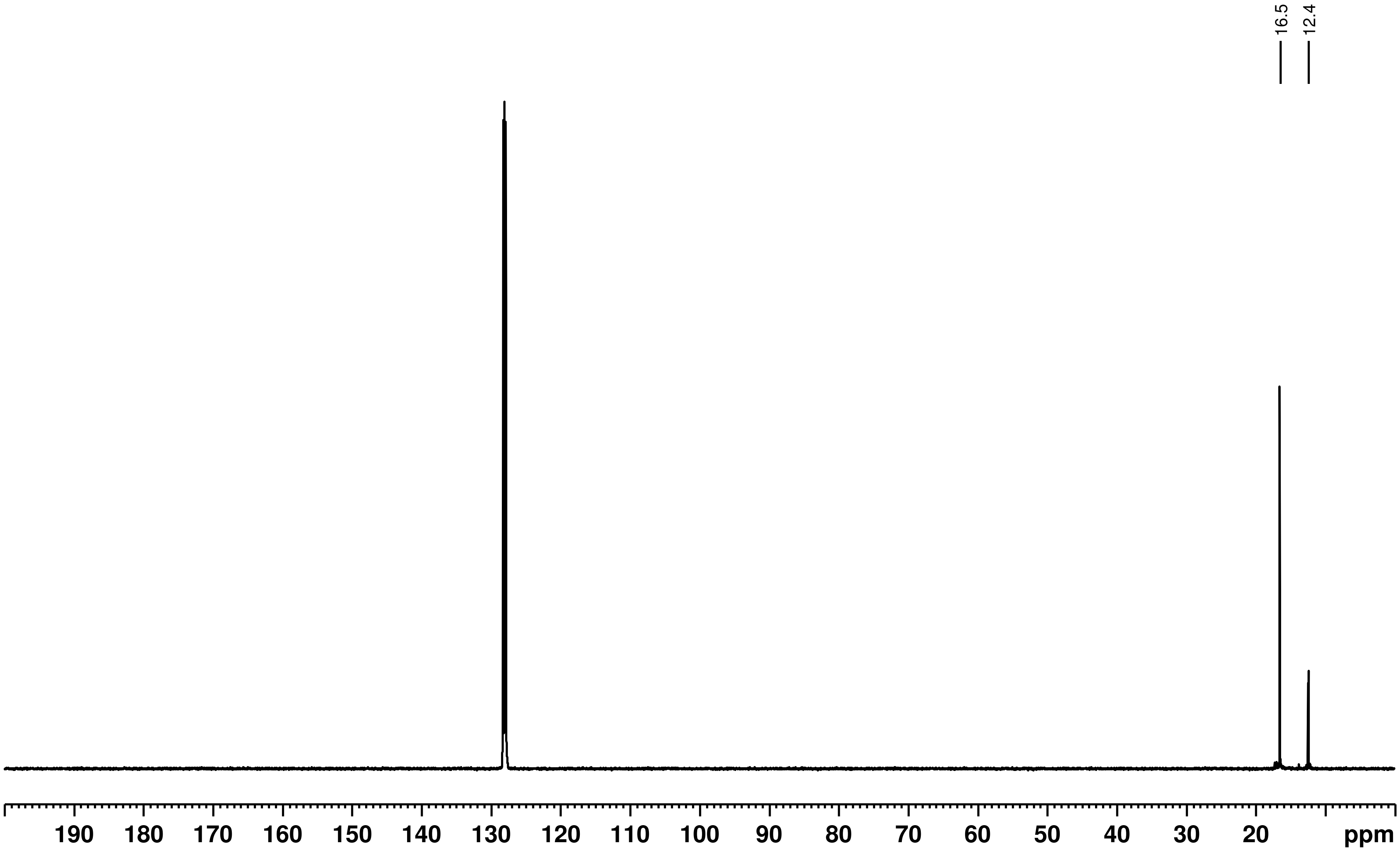

Supplementary Fig. 64. ^{19}**F NMR** spectrum (659 MHz, C_6D_6, 298 K) of fluorodiisopropylsilane (**6ac**)

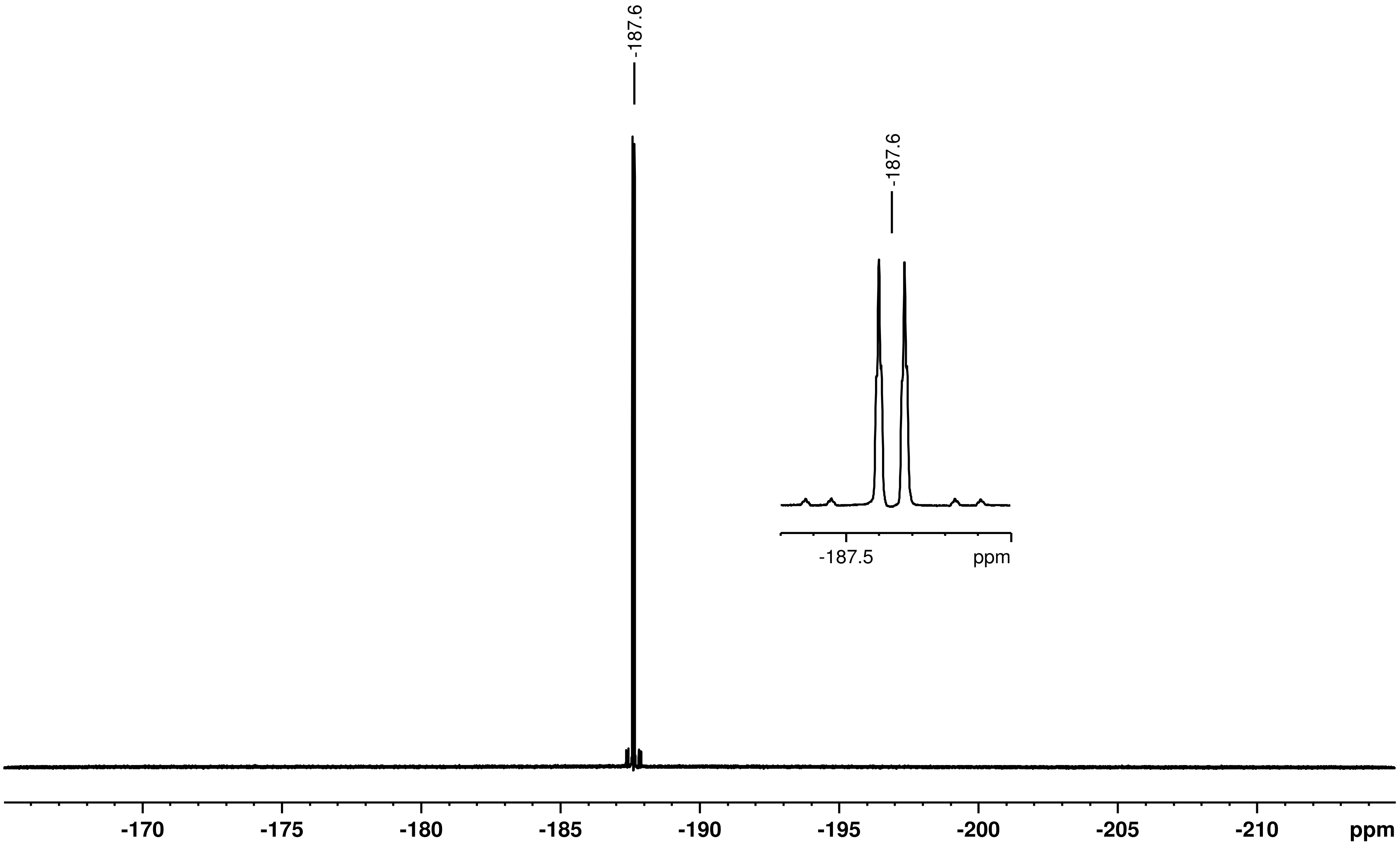

Supplementary Fig. 65. **^{29}Si{^{1}H} DEPT NMR** spectrum (99 MHz, C_6D_6, 298 K, optimized for $J_{H,Si}$ = 7 Hz, 15.5°) of fluorodiisopropylsilane (**6ac**)

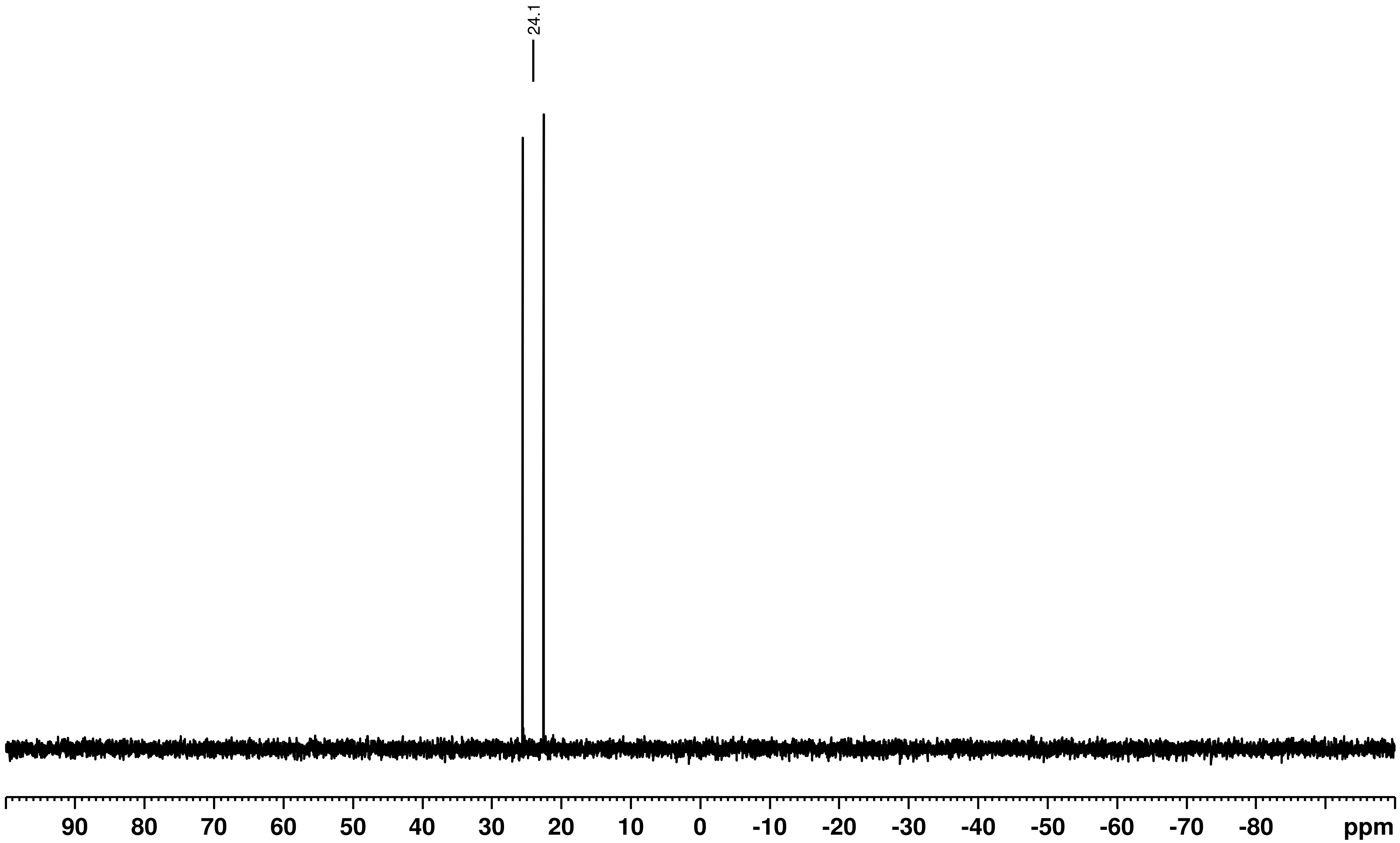

Supplementary Fig. 66. **IR** spectrum (ATR) of fluorodiisopropylsilane (**6ac**)

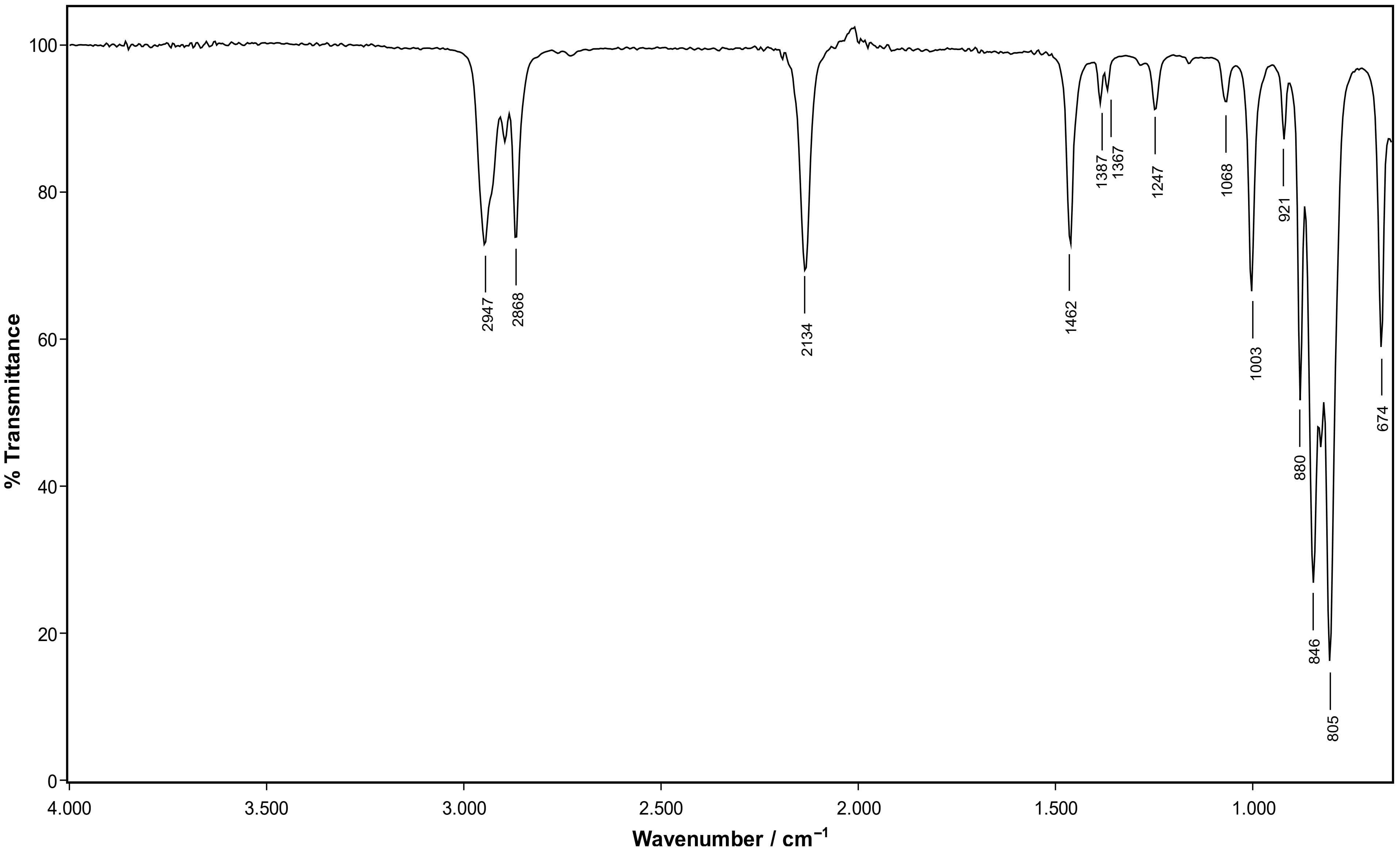

Supplementary Fig. 67. **^{1}H NMR** spectrum (500 MHz, C_6D_6, 298 K) of diisopropylsilane (**3c**)

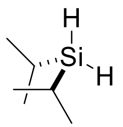

3c

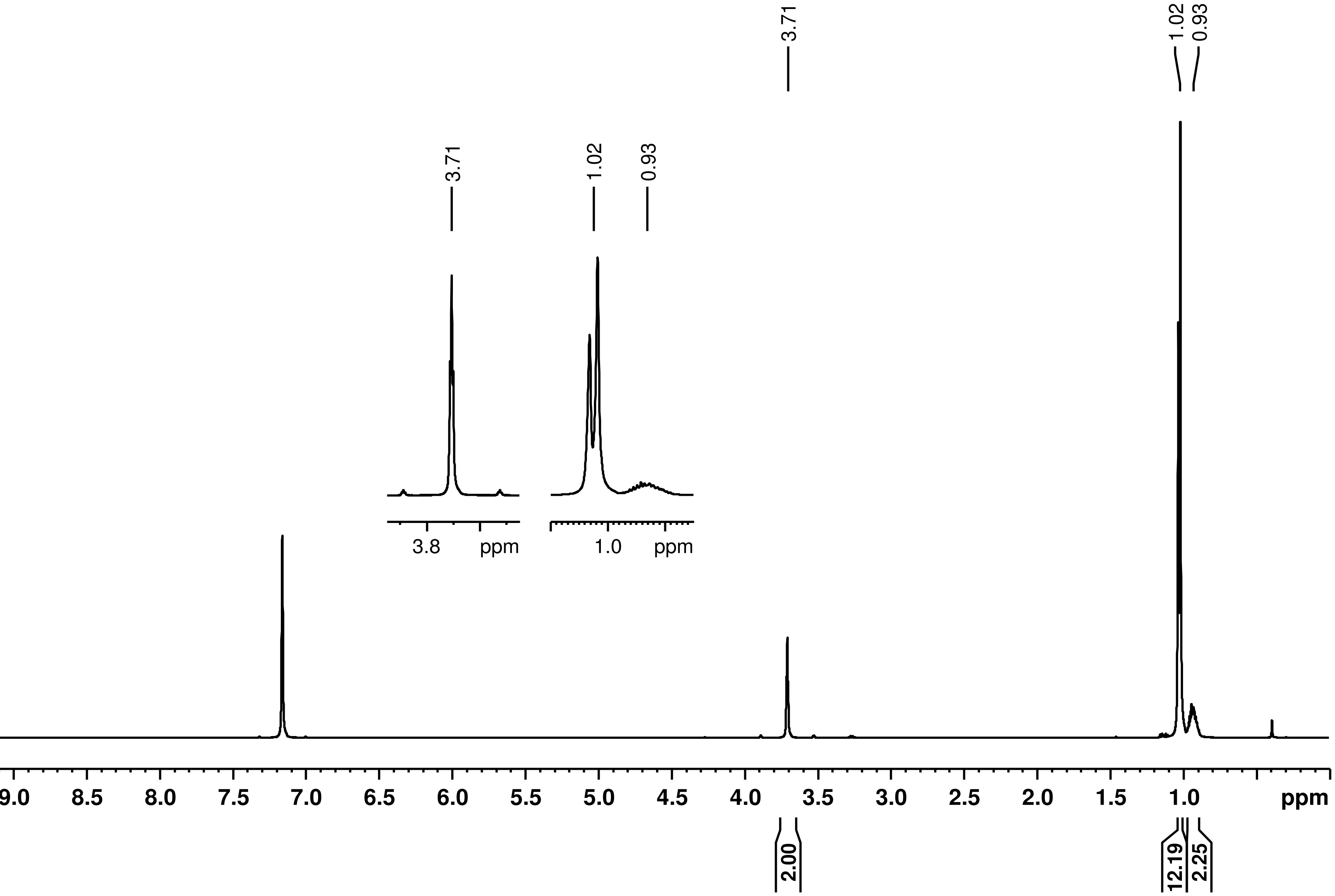

Supplementary Fig. 68. **^{13}C{^{1}H} NMR** spectrum (126 MHz, C_6D_6, 298 K) of diisopropylsilane (**3c**)

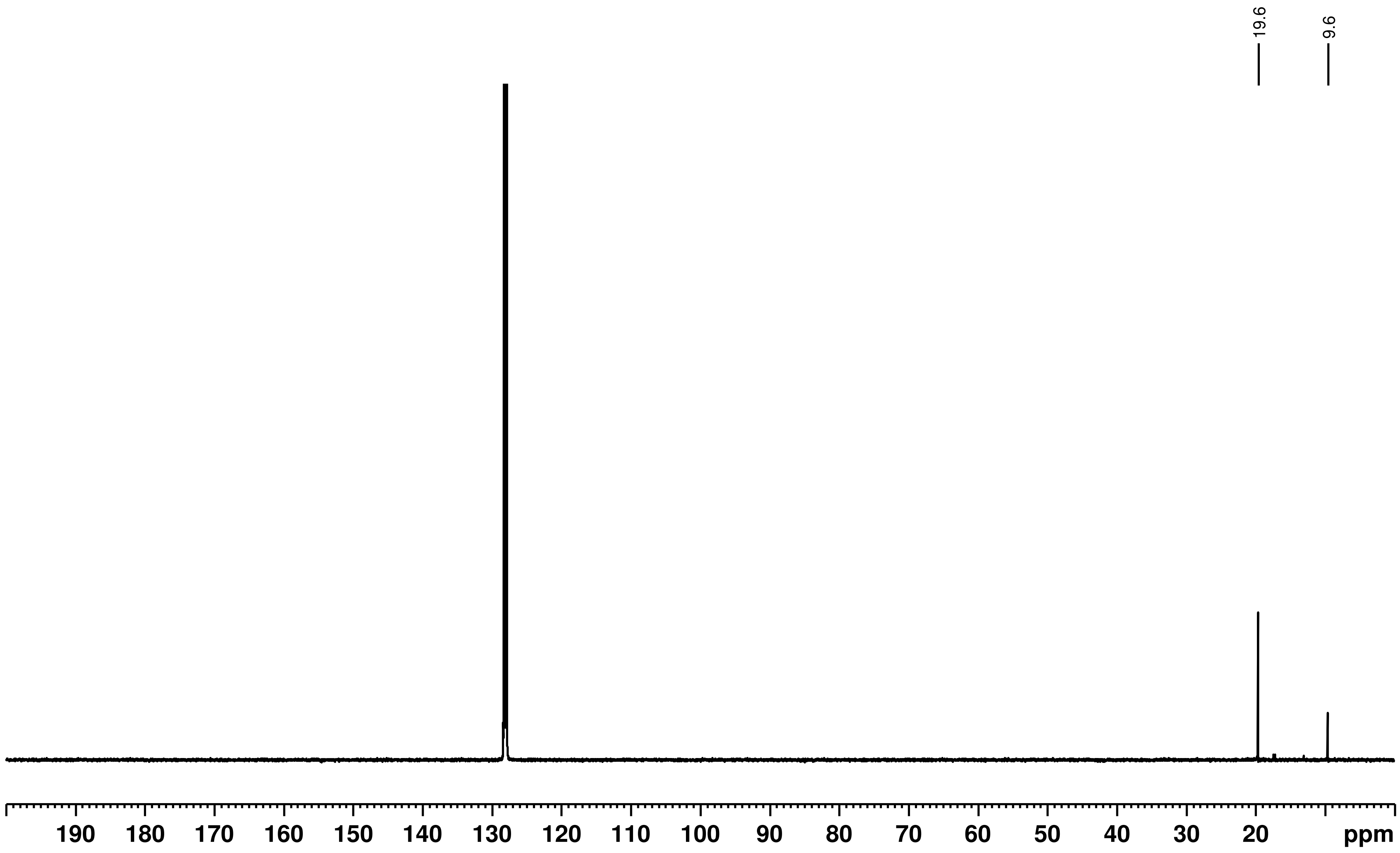

Supplementary Fig. 69. **^{29}Si{^{1}H} DEPT NMR** spectrum (99 MHz, C_6D_6, 298 K, optimized for $J_{H,Si}$ = 200 Hz, 45.0°) of diisopropylsilane (**3c**)

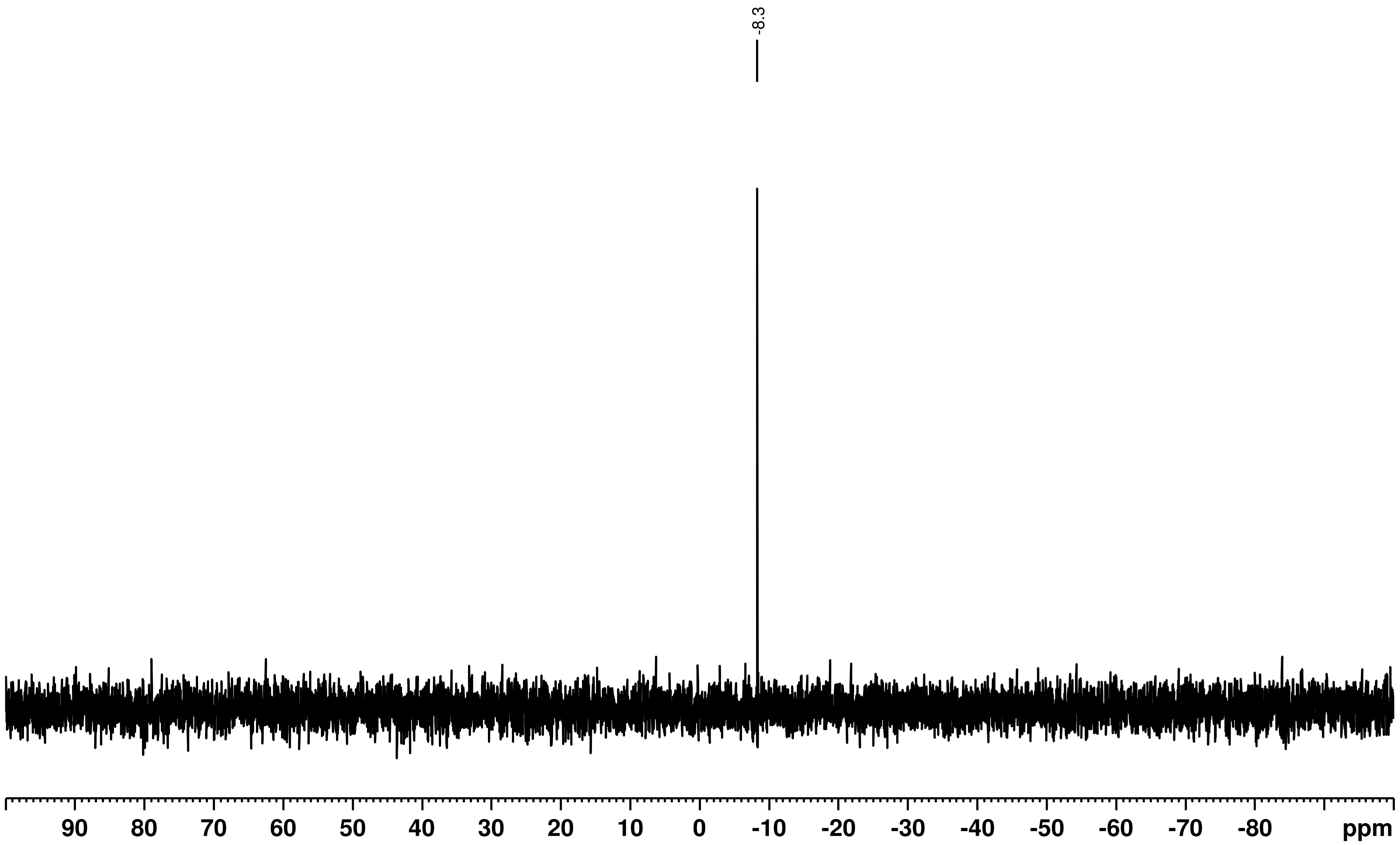

Supplementary Fig. 70. **IR** spectrum (ATR) of diisopropylsilane (**3c**)

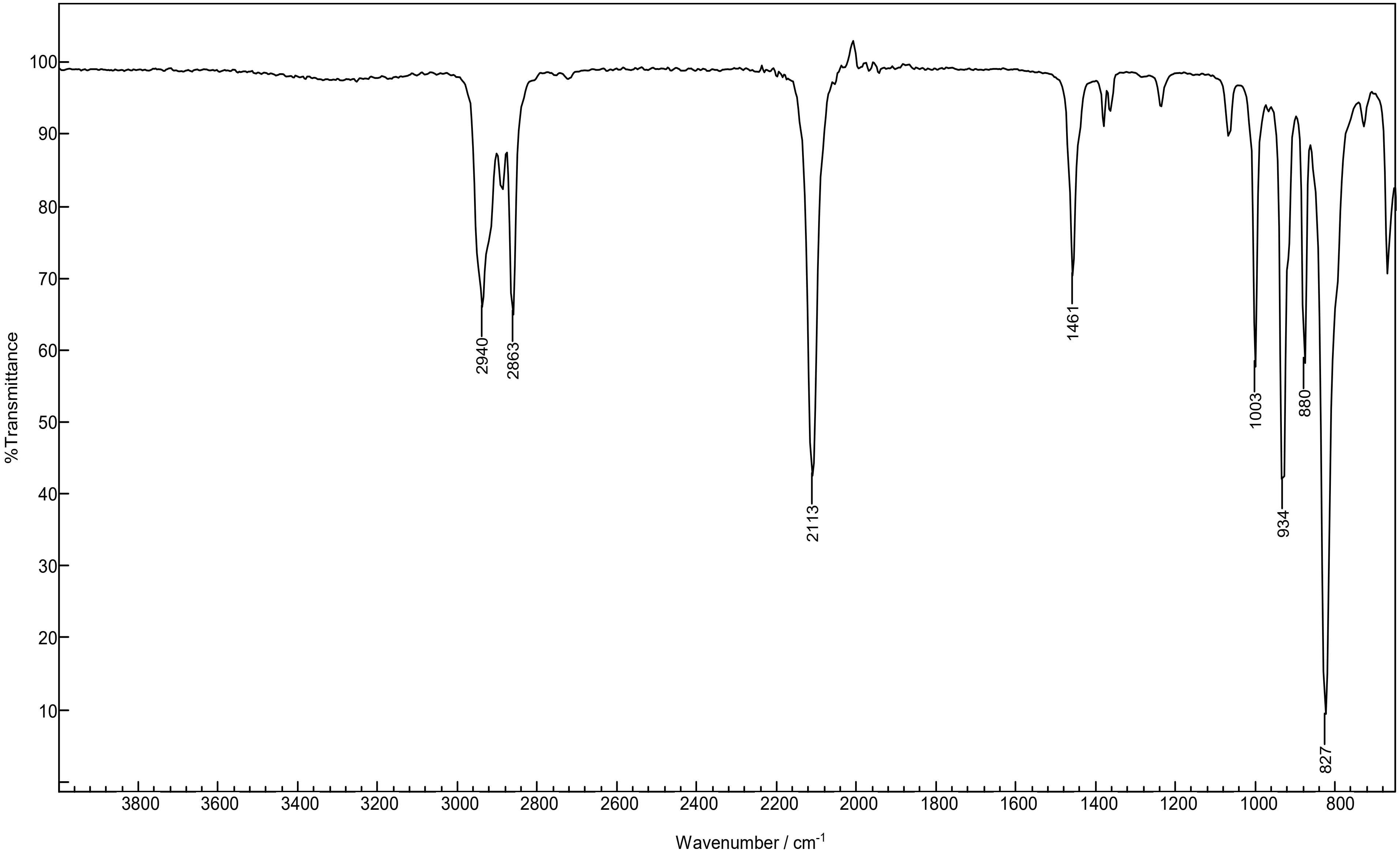

Supplementary Fig. 71. [1]**H NMR** spectrum (500 MHz, C_6D_6, 298 K) of bromodiisopropylsilane (**6cc**)

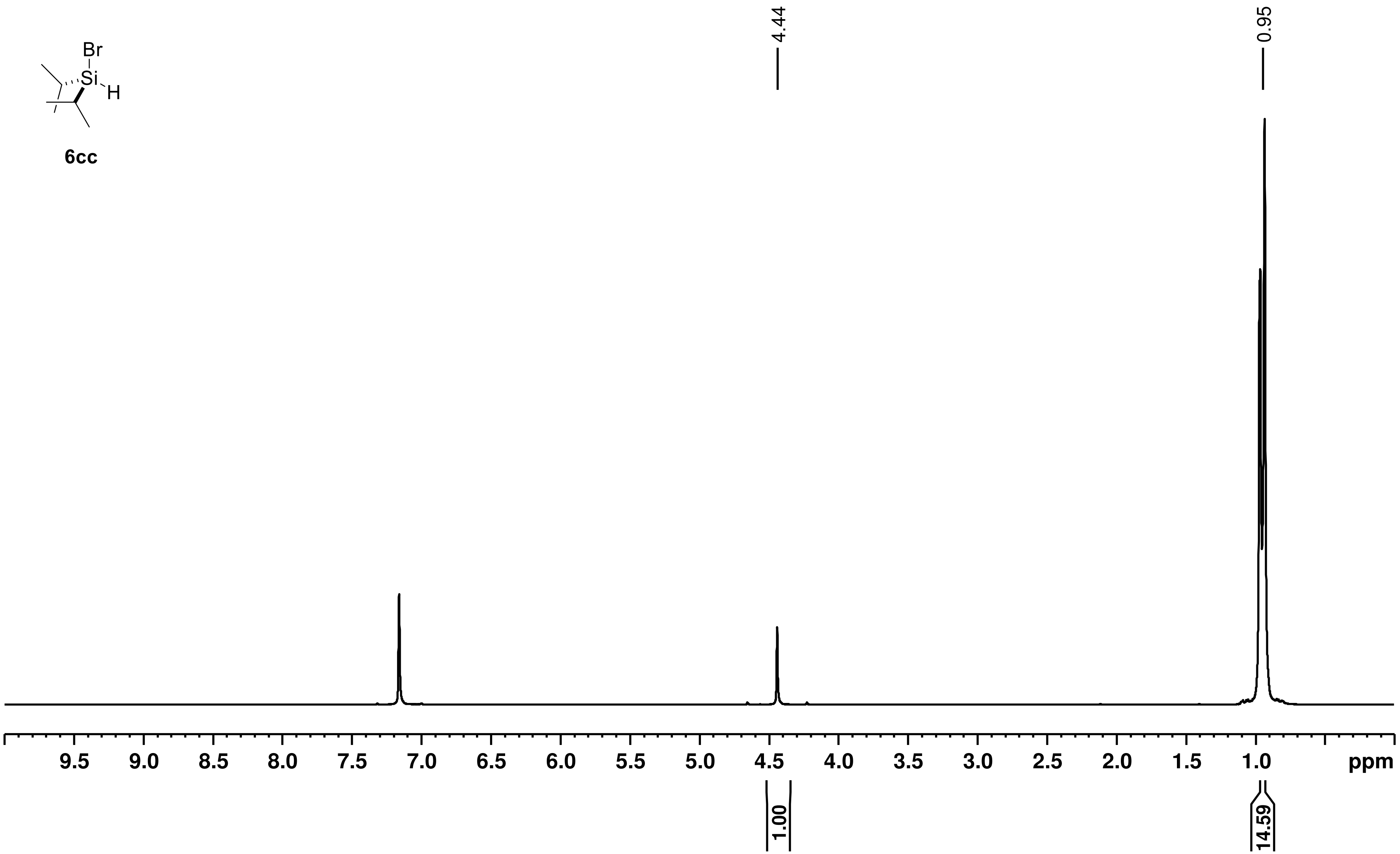

Supplementary Fig. 72. **^{13}C{^{1}H} NMR** spectrum (126 MHz, C_6D_6, 298 K) of bromodiisopropylsilane (**6cc**)

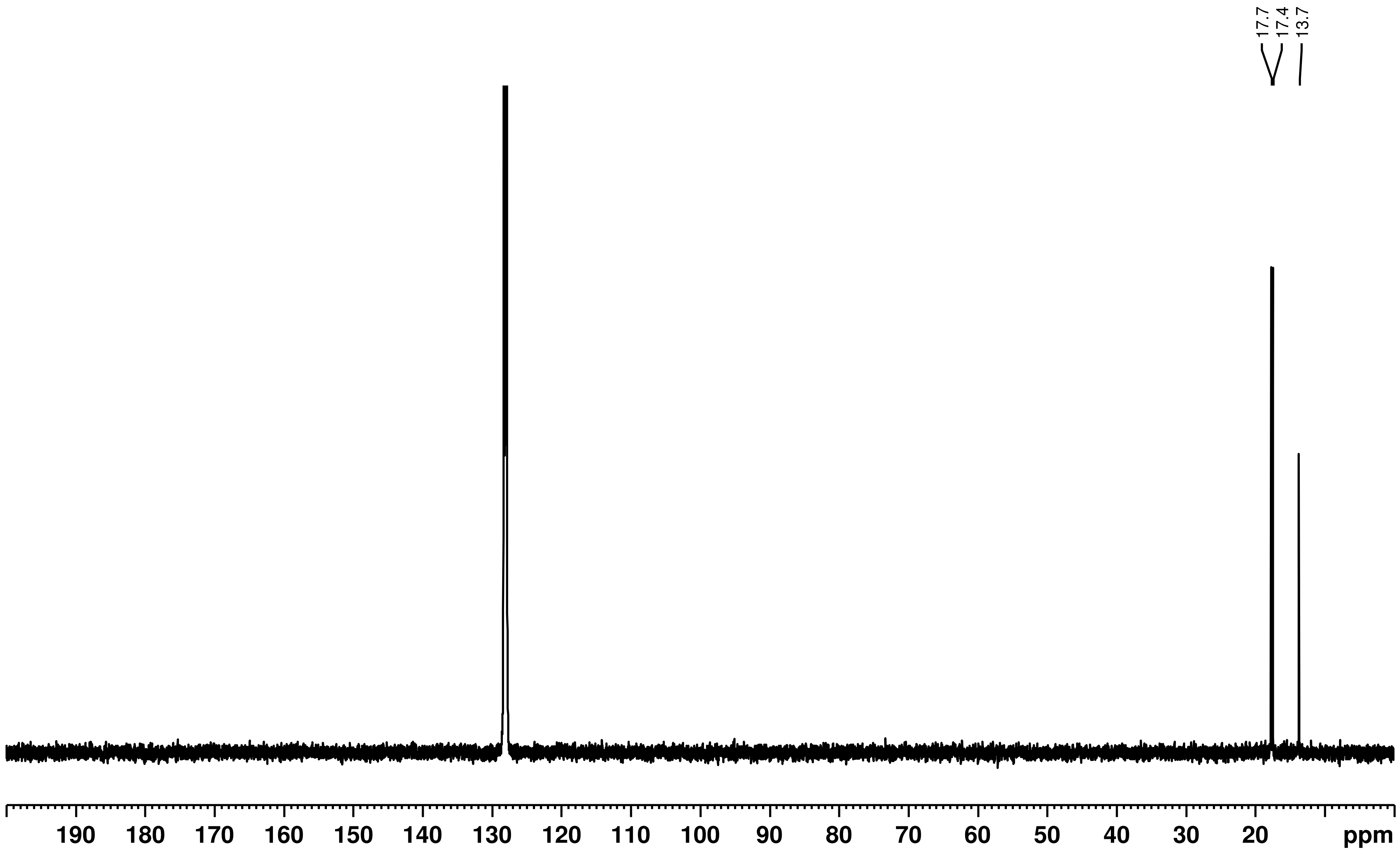

Supplementary Fig. 73. ^{29}**Si{^{1}H} DEPT NMR** spectrum (99 MHz, C_6D_6, 298 K, optimized for $J_{H,Si}$ = 7 Hz, 15.5°) of bromodiisopropylsilane (**6cc**)

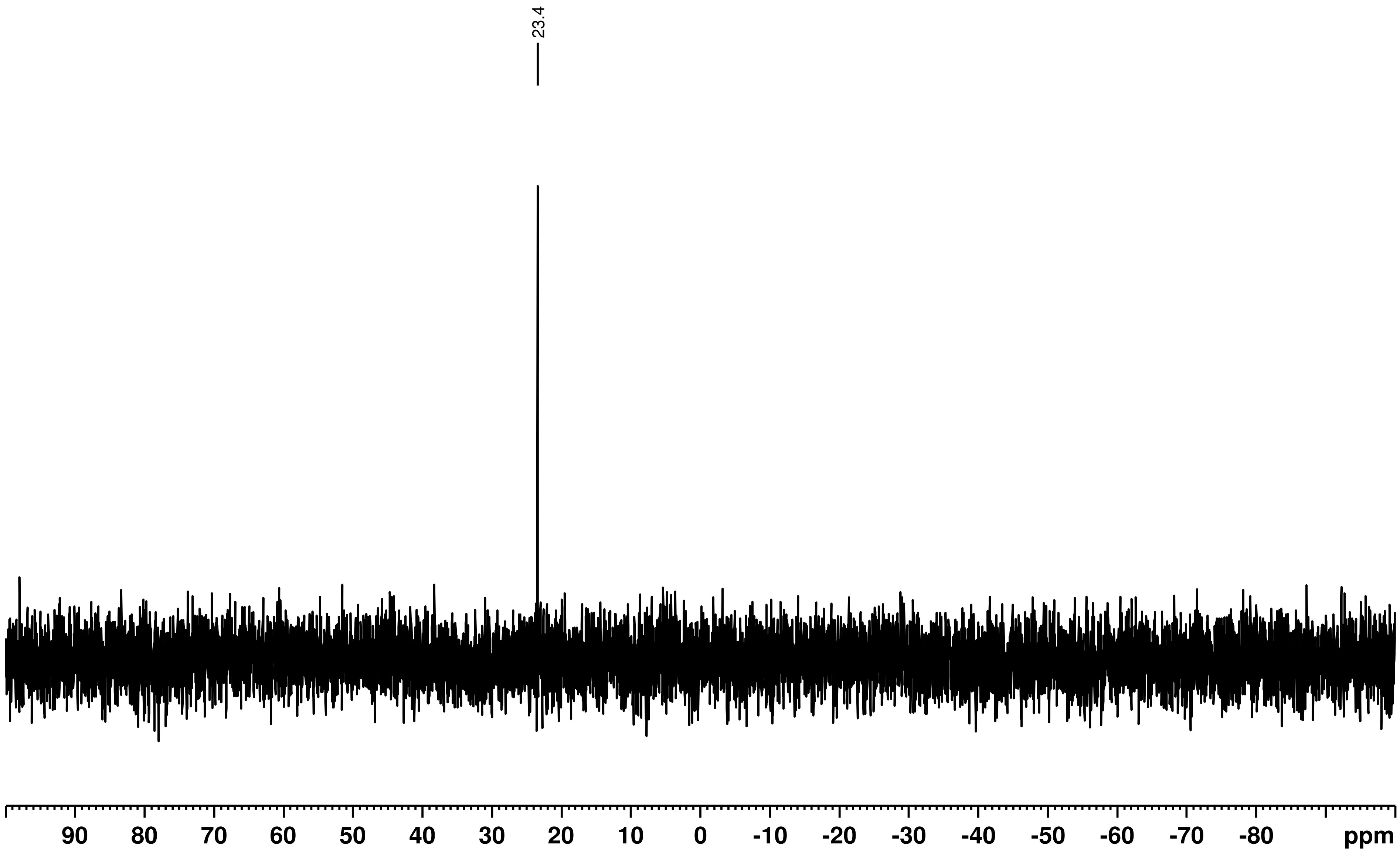

Supplementary Fig. 74. **IR** spectrum (ATR) of bromodiisopropylsilane (**6cc**)

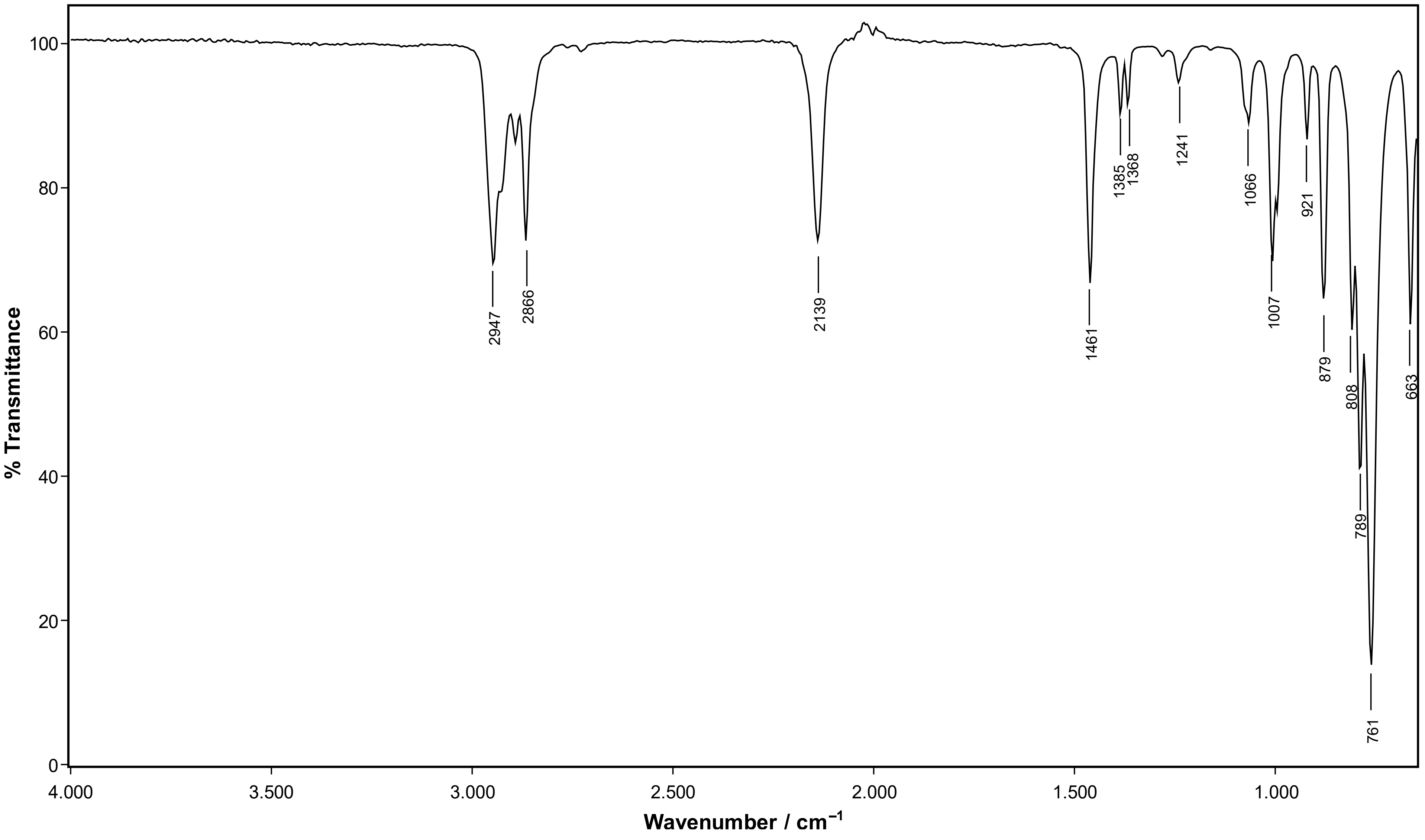

Supplementary Fig. 75. ^{1}**H NMR** spectrum (500 MHz, C_6D_6, 298 K) of diisopropyliodosilane (**6dc**)

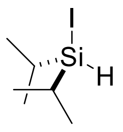

6dc

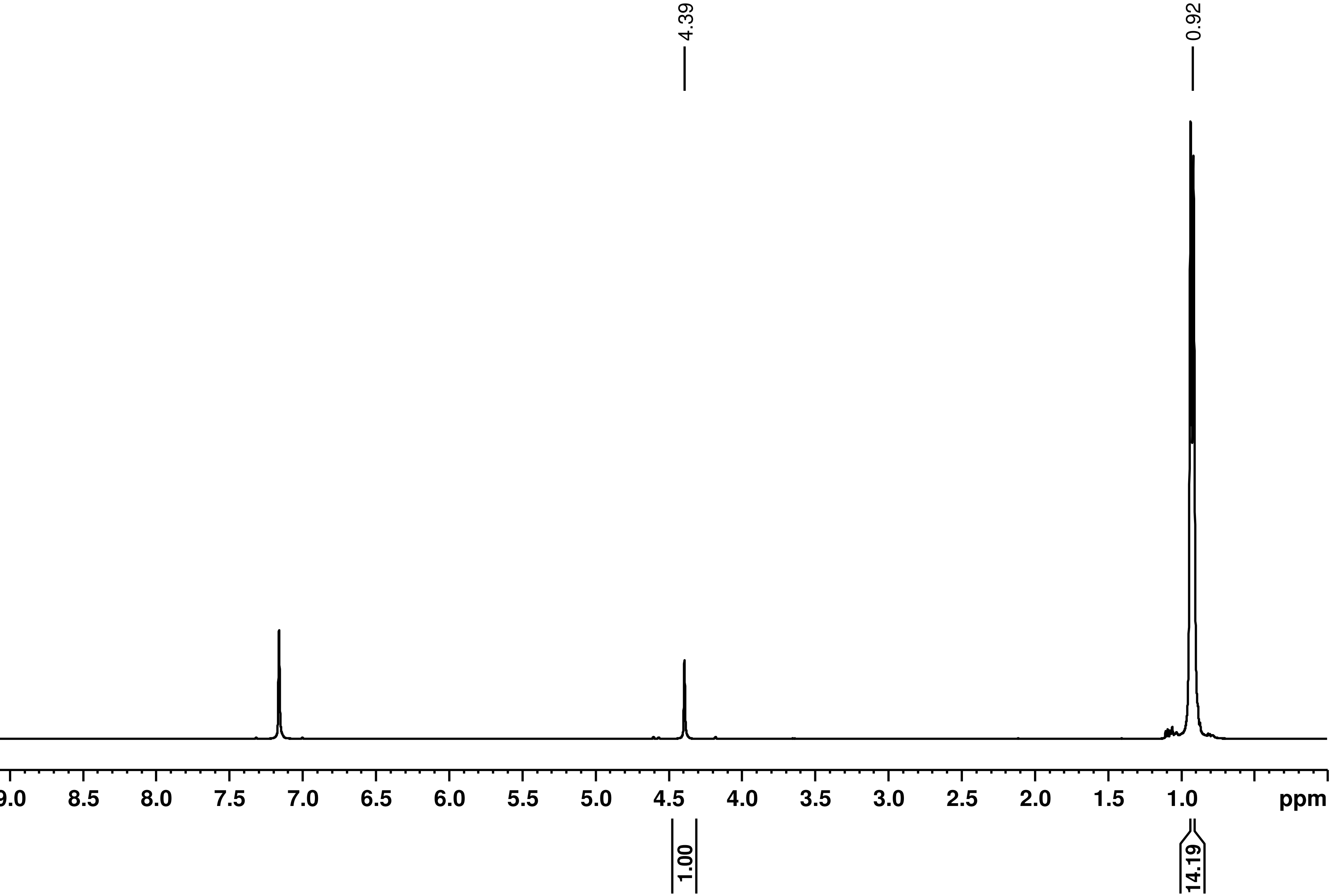

Supplementary Fig. 76. **^{13}C{^{1}H} NMR** spectrum (126 MHz, C_6D_6, 298 K) of diisopropyliodosilane (**6dc**)

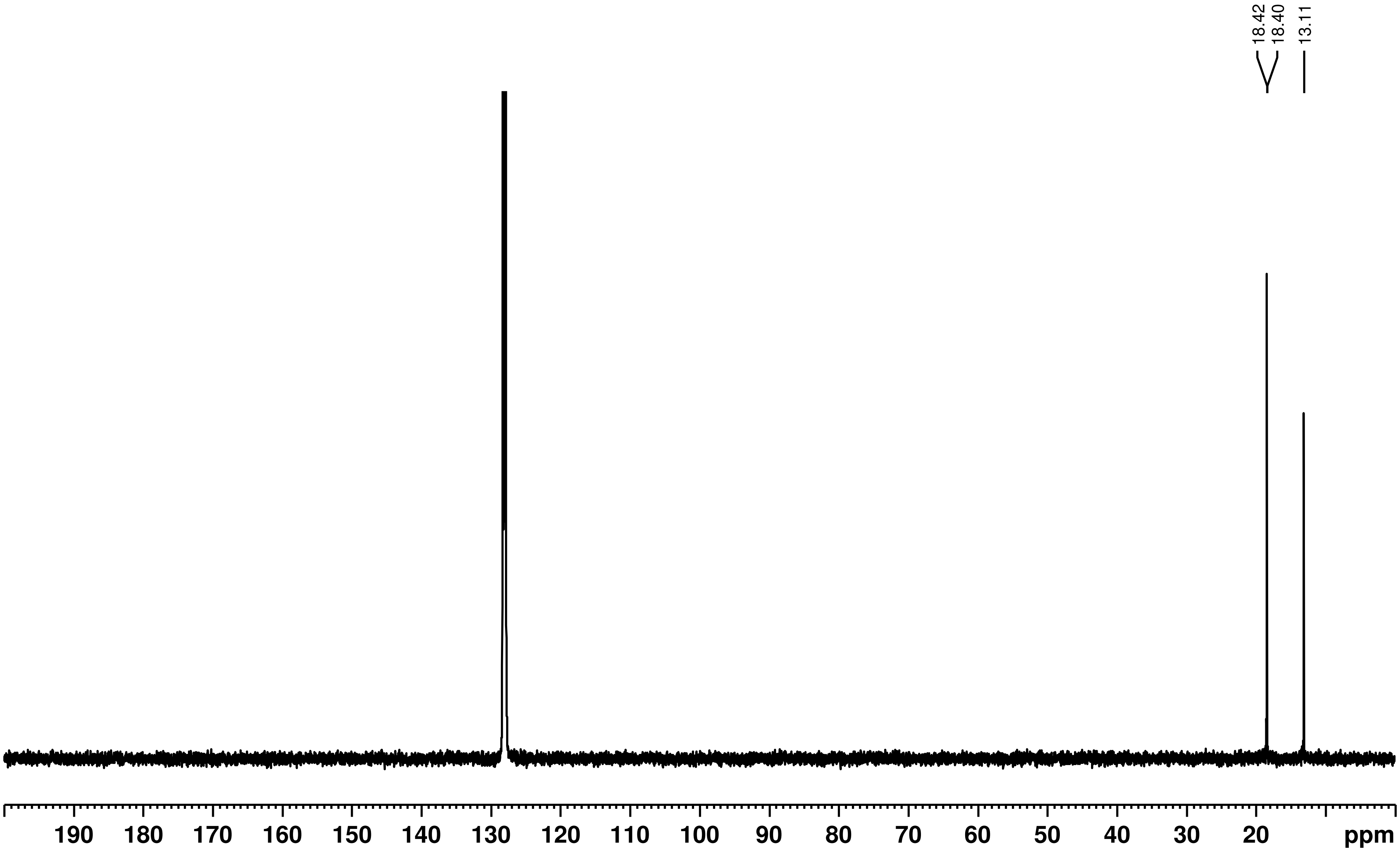

Supplementary Fig. 77. **^{29}Si{^{1}H} DEPT NMR** spectrum (99 MHz, C_6D_6, 298 K, optimized for $J_{H,Si}$ = 7 Hz, 15.5°) of diisopropyliodosilane (**6dc**)

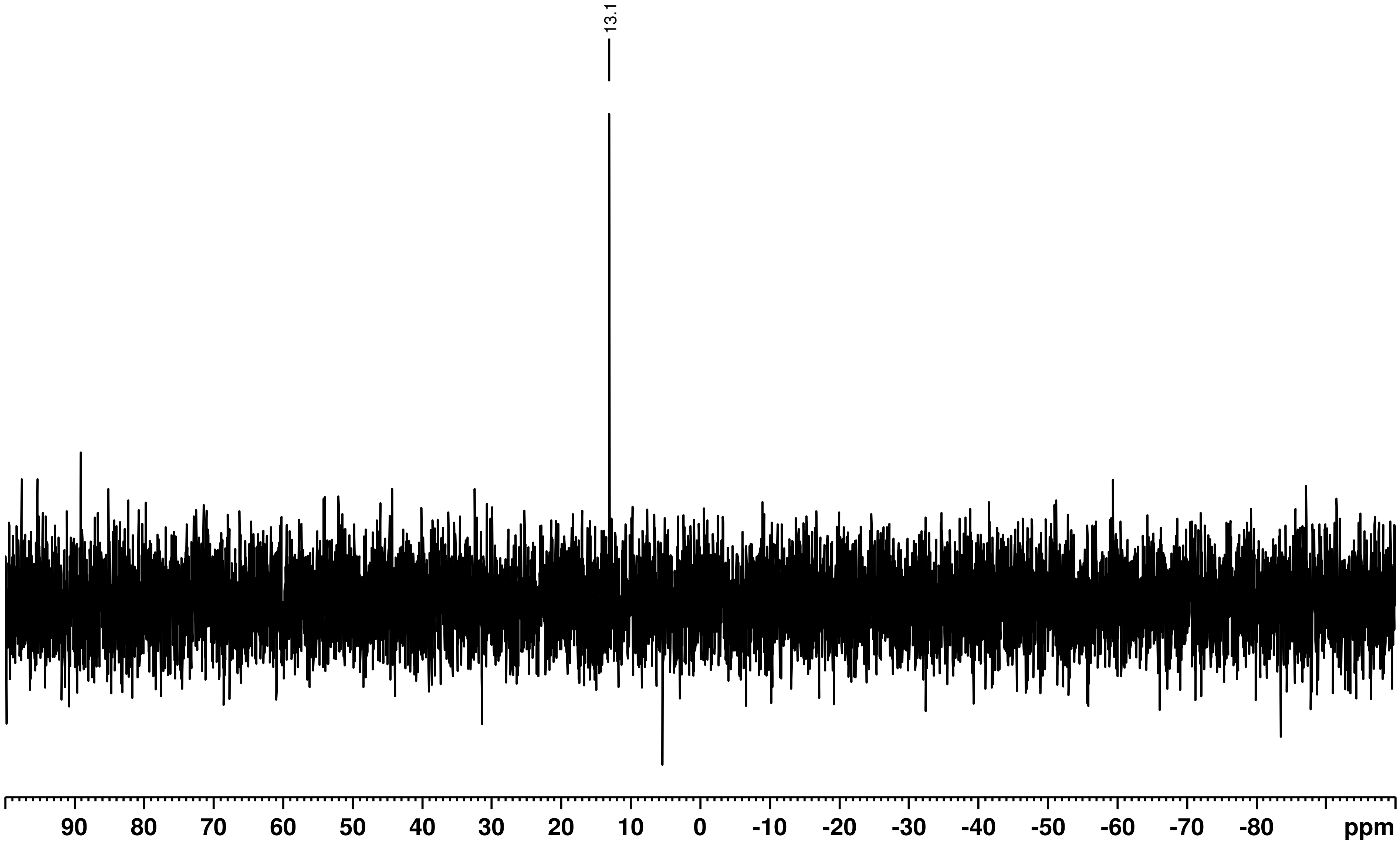

Supplementary Fig. 78. **^{1}H NMR** spectrum (500 MHz, C_6D_6, 298 K) of trifluoro(phenyl)silane (**S8**)

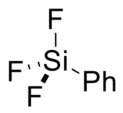

S8

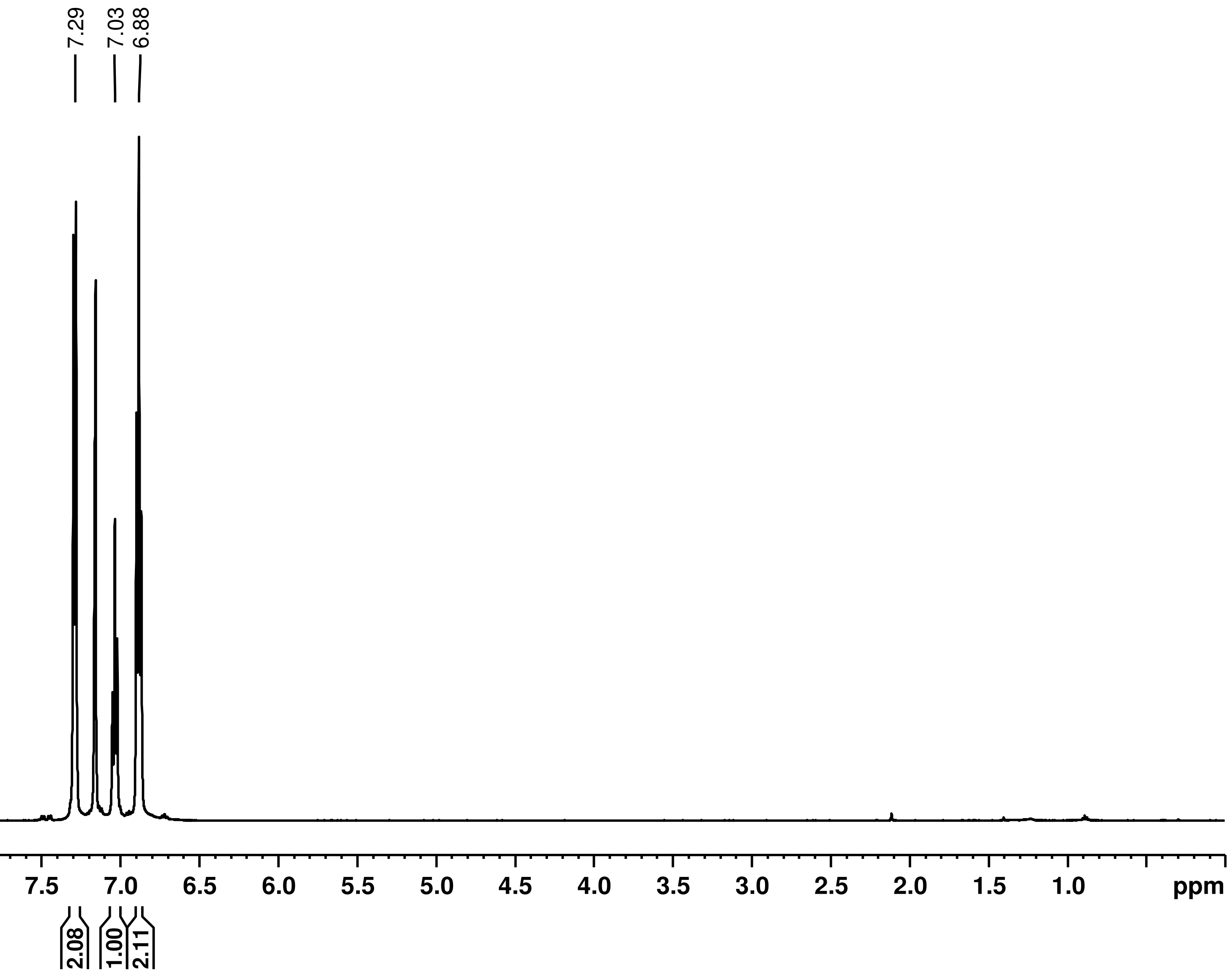

Supplementary Fig. 79. **^{13}C{^{1}H} NMR** spectrum (126 MHz, CD_2Cl_2, 298 K) of trifluoro(phenyl)silane (**7ab**)

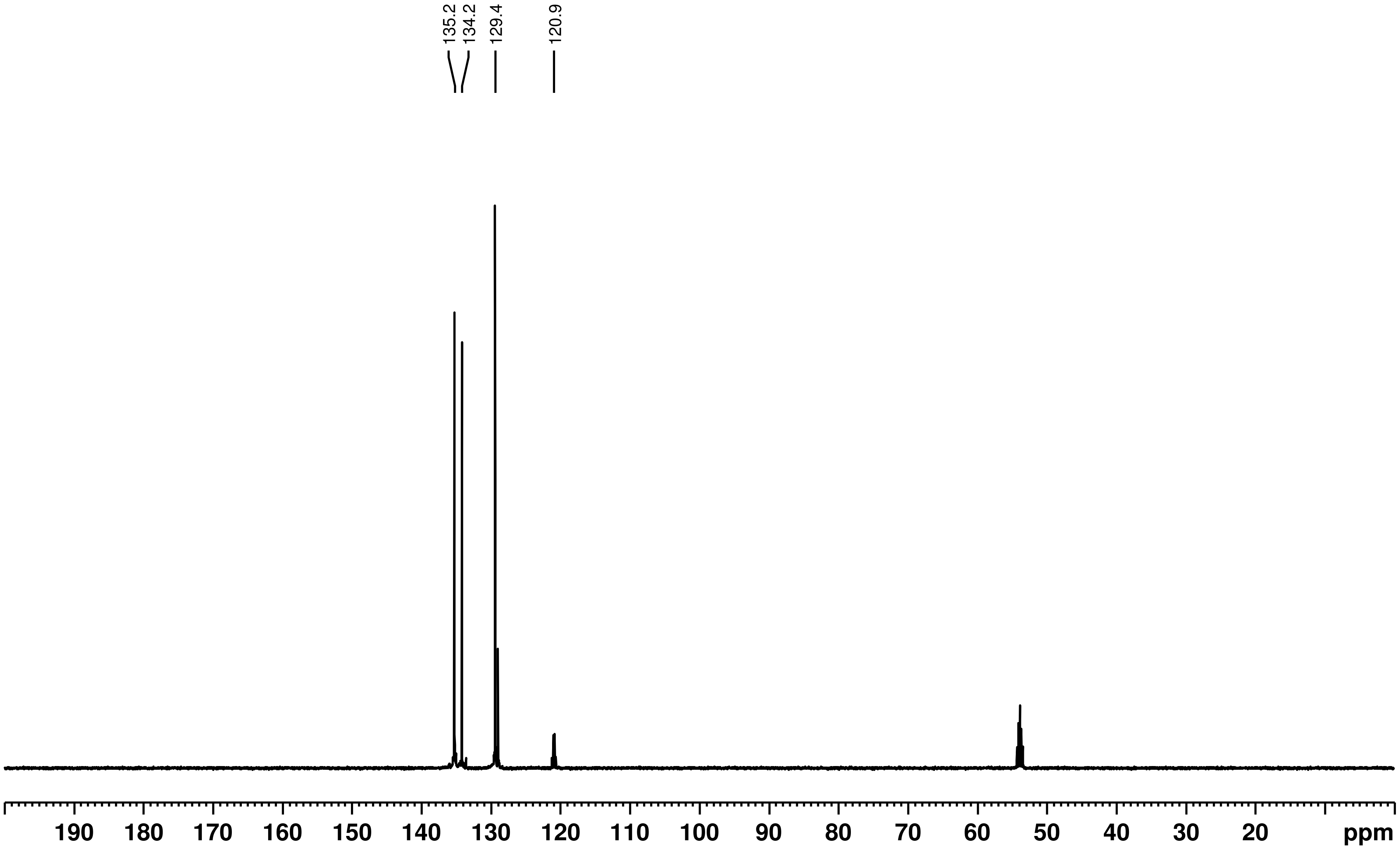

Supplementary Fig. 80. ^{19}**F NMR** spectrum (471 MHz, C_6D_6, 298 K) of trifluoro(phenyl)silane (**7ab**)

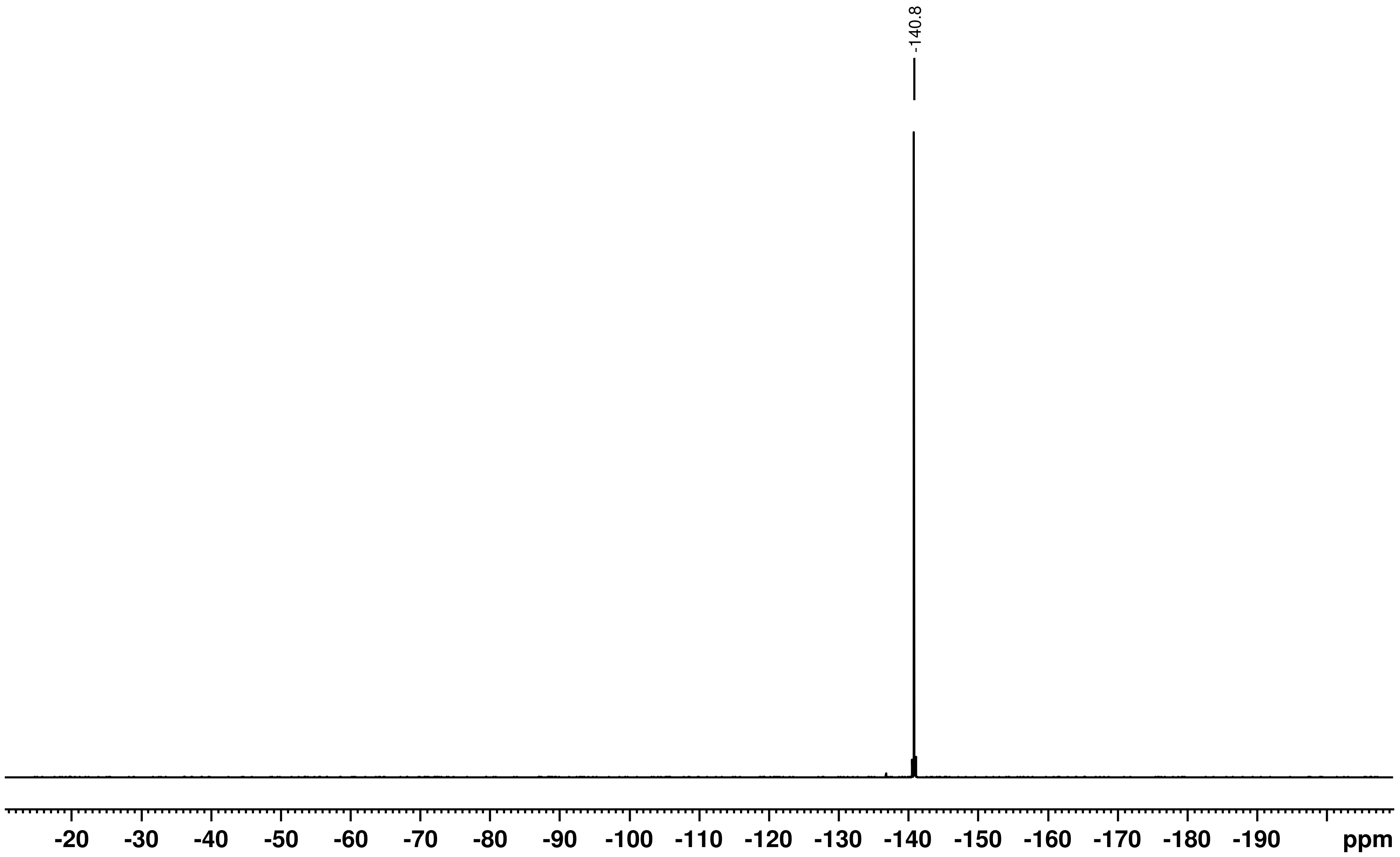

Supplementary Fig. 81. ^{29}**Si{^{1}H} IG NMR** spectrum (99 MHz, CD_2Cl_2, 298 K) of trifluoro(phenyl)silane (**7ab**)

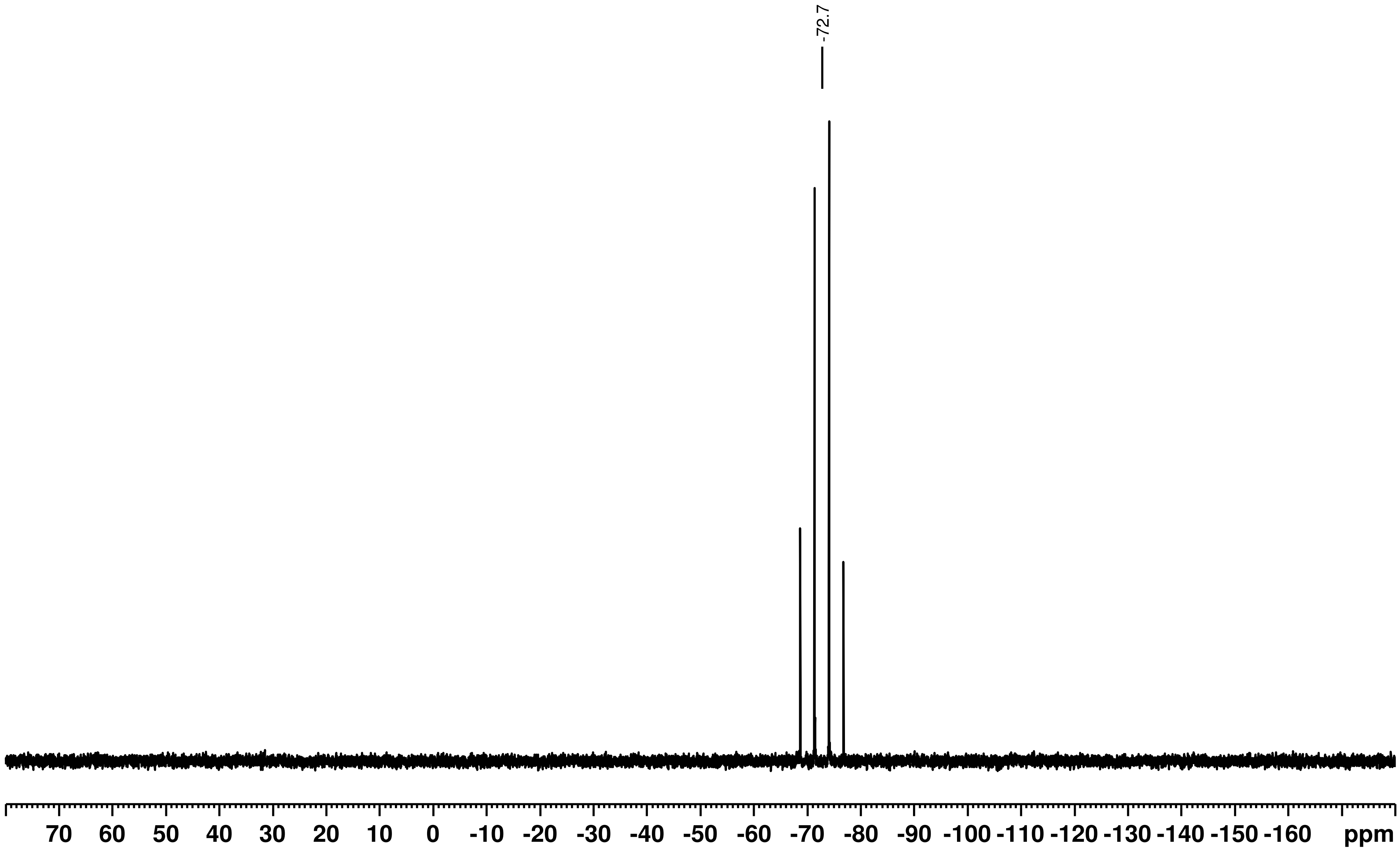

Supplementary Fig. 82. **IR** spectrum (ATR) of trifluoro(phenyl)silane (**7ab**)

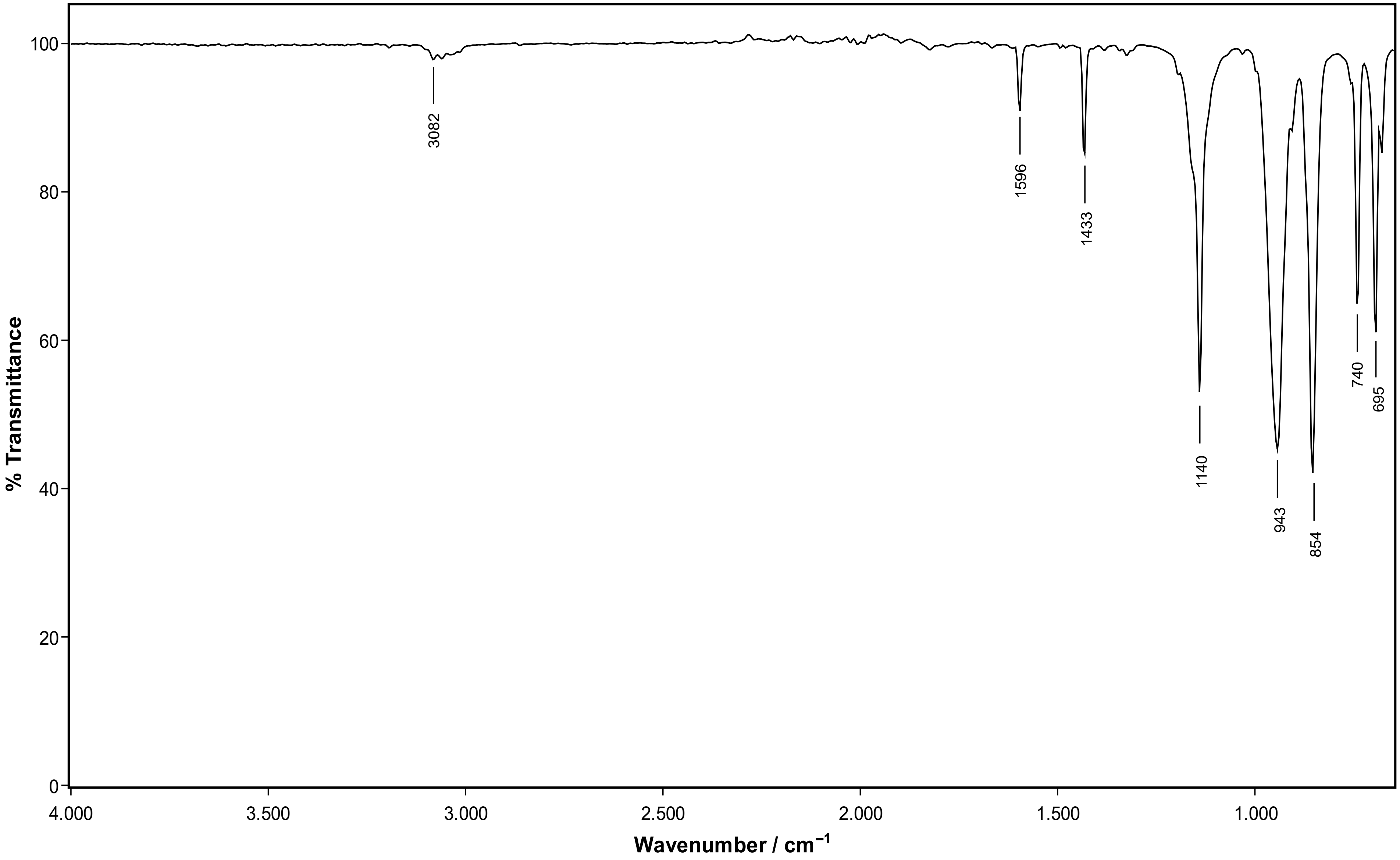

Supplementary Fig. 83. **^{1}H NMR** spectrum (500 MHz, C_6D_6, 298 K) of di-*tert*-butylfluoro(phenyl)silane (**7ad**)

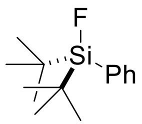

7ad

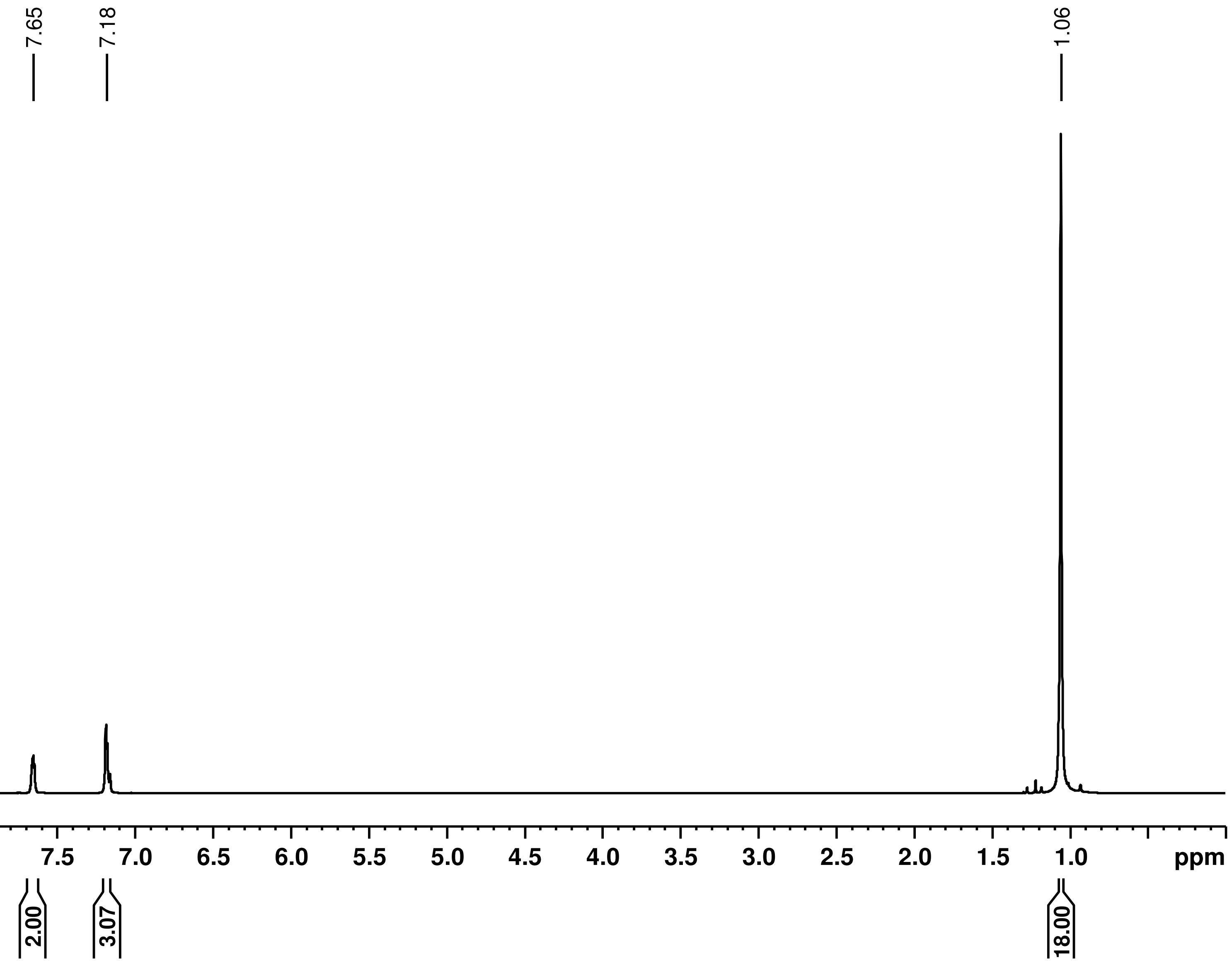

Supplementary Fig. 84. **^{13}C{^{1}H} NMR** spectrum (126 MHz, C_6D_6, 298 K) of di-*tert*-butylfluoro(phenyl)silane (**7ad**)

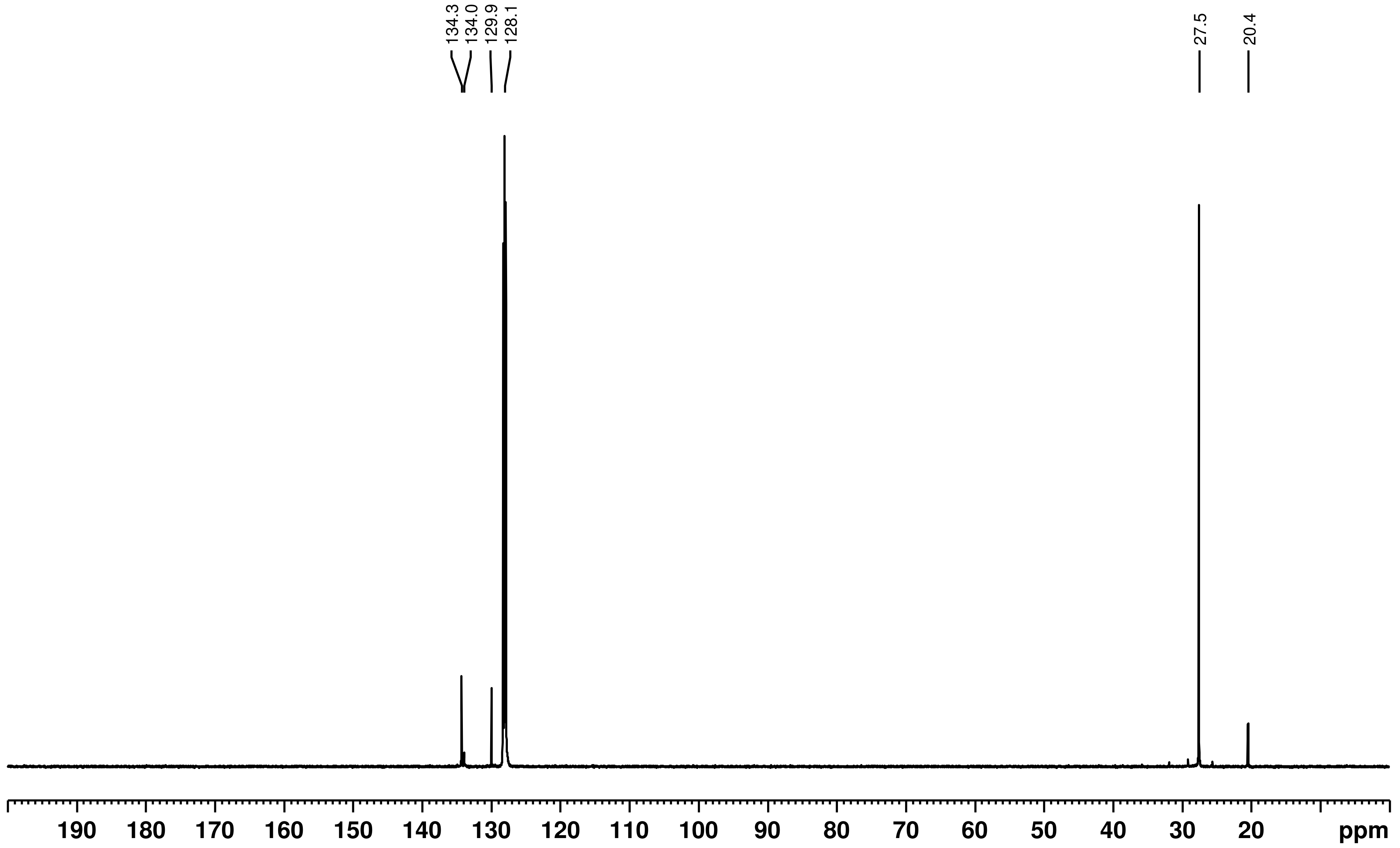

Supplementary Fig. 85. ^{19}**F NMR** spectrum (471 MHz, C_6D_6, 298 K) of di-*tert*-butylfluoro(phenyl)silane (**7ad**)

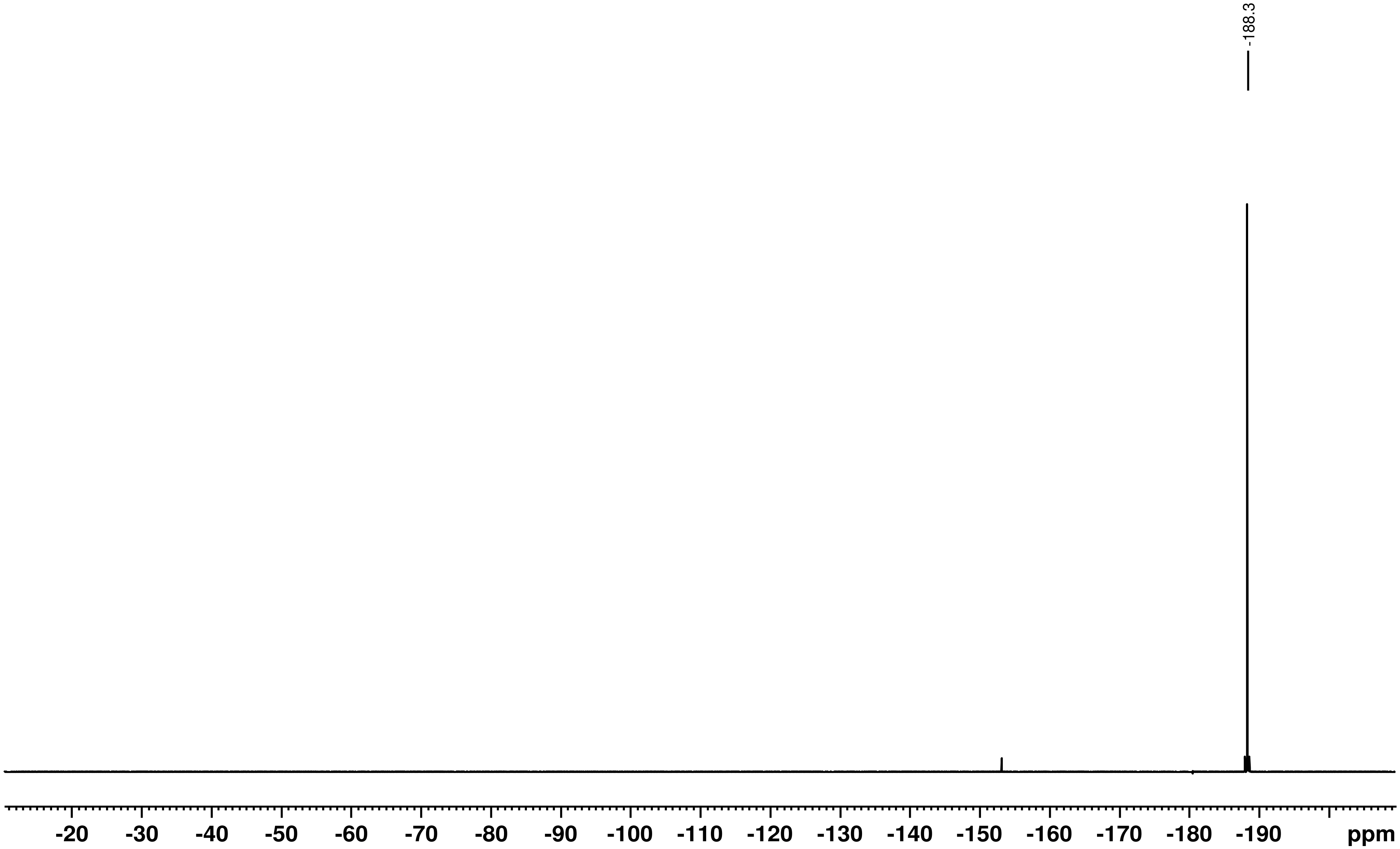

Supplementary Fig. 86. **^{29}Si{^{1}H} DEPT NMR** spectrum (99 MHz, C_6D_6, 298 K, optimized for $J_{H,Si}$ = 7 Hz, 12.9°) of di-*tert*-butylfluoro(phenyl)silane (**7ad**)

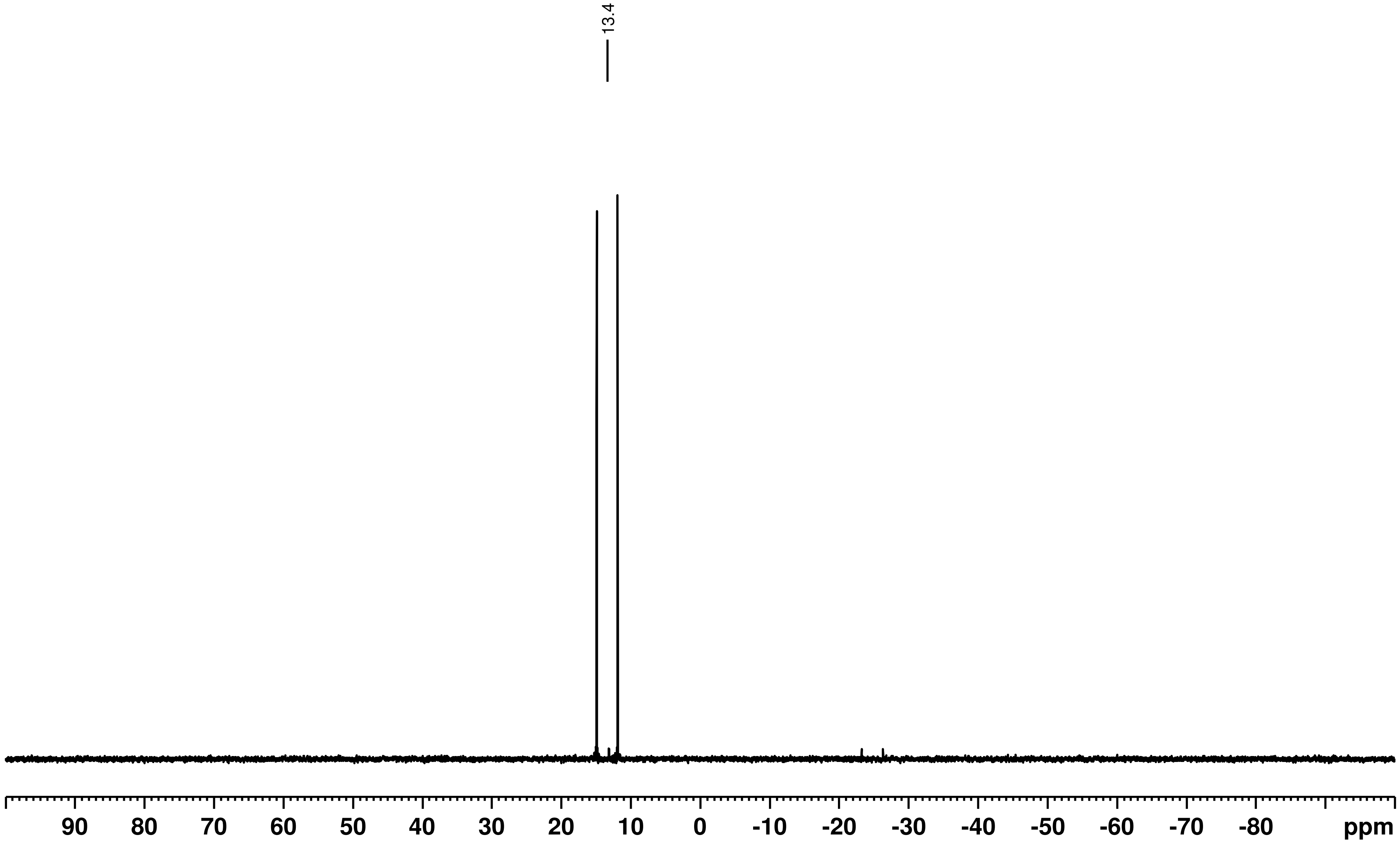

Supplementary Fig. 87. **IR** spectrum (ATR) of di-*tert*-butylfluoro(phenyl)silane (**7ad**)

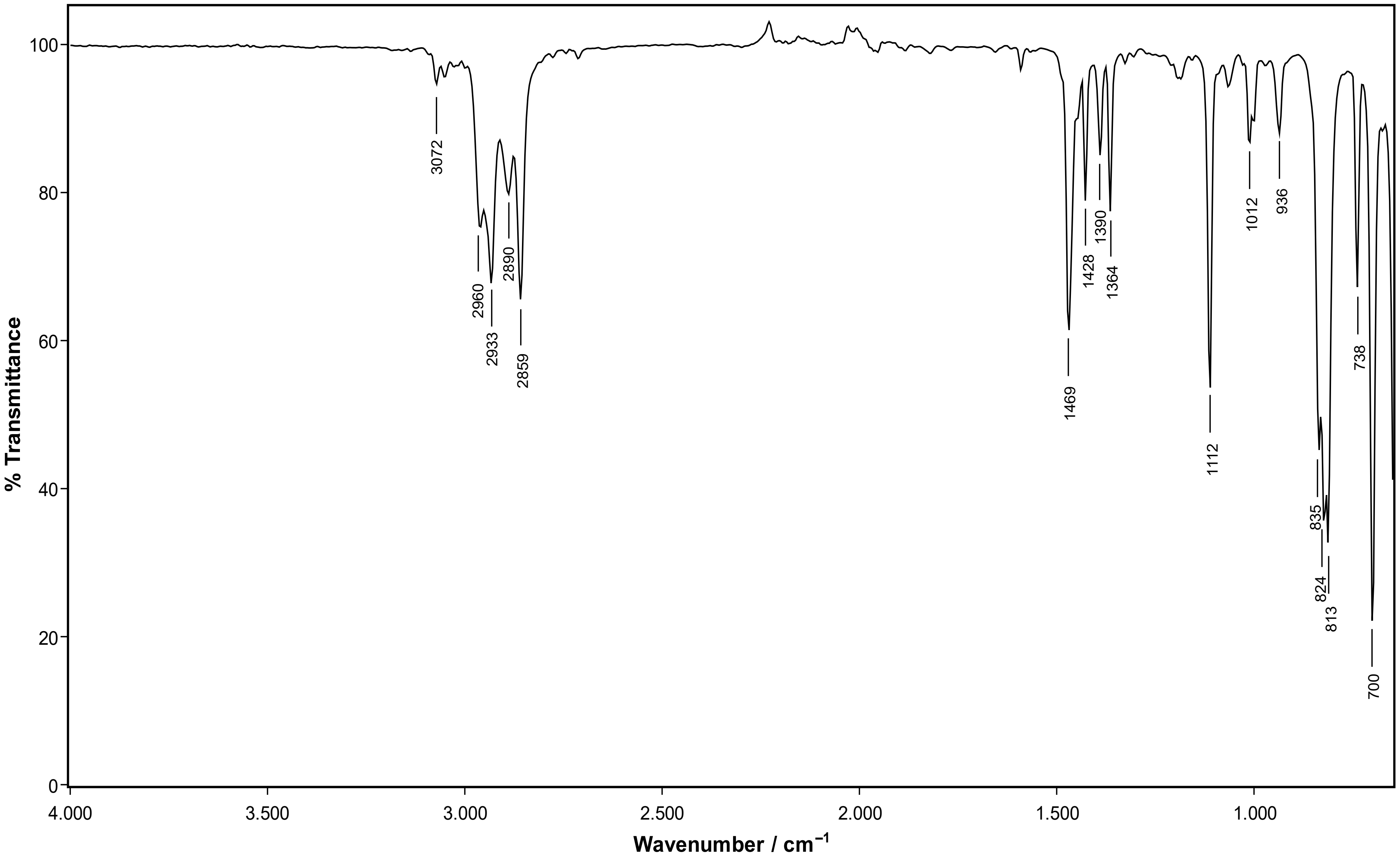

Supplementary Fig. 88. **GLC-MS** spectrum (EI) of di-*tert*-butylfluoro(phenyl)silane (**7ad**)

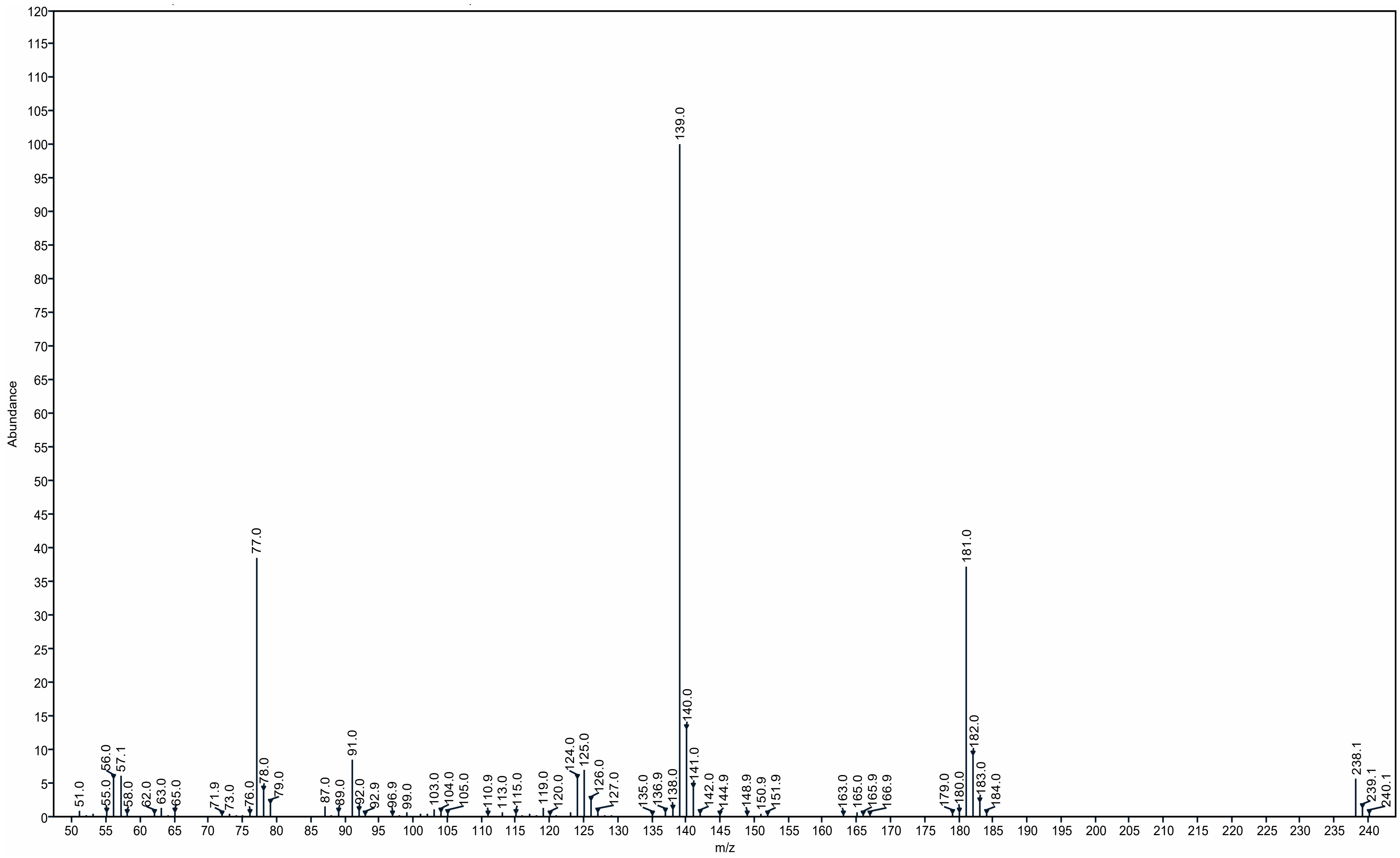

Supplementary Fig. 89. **^{1}H NMR** spectrum (500 MHz, C_6D_6, 298 K) of di-*tert*-butylchlorosilane (**6bd**)

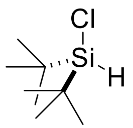

6bd

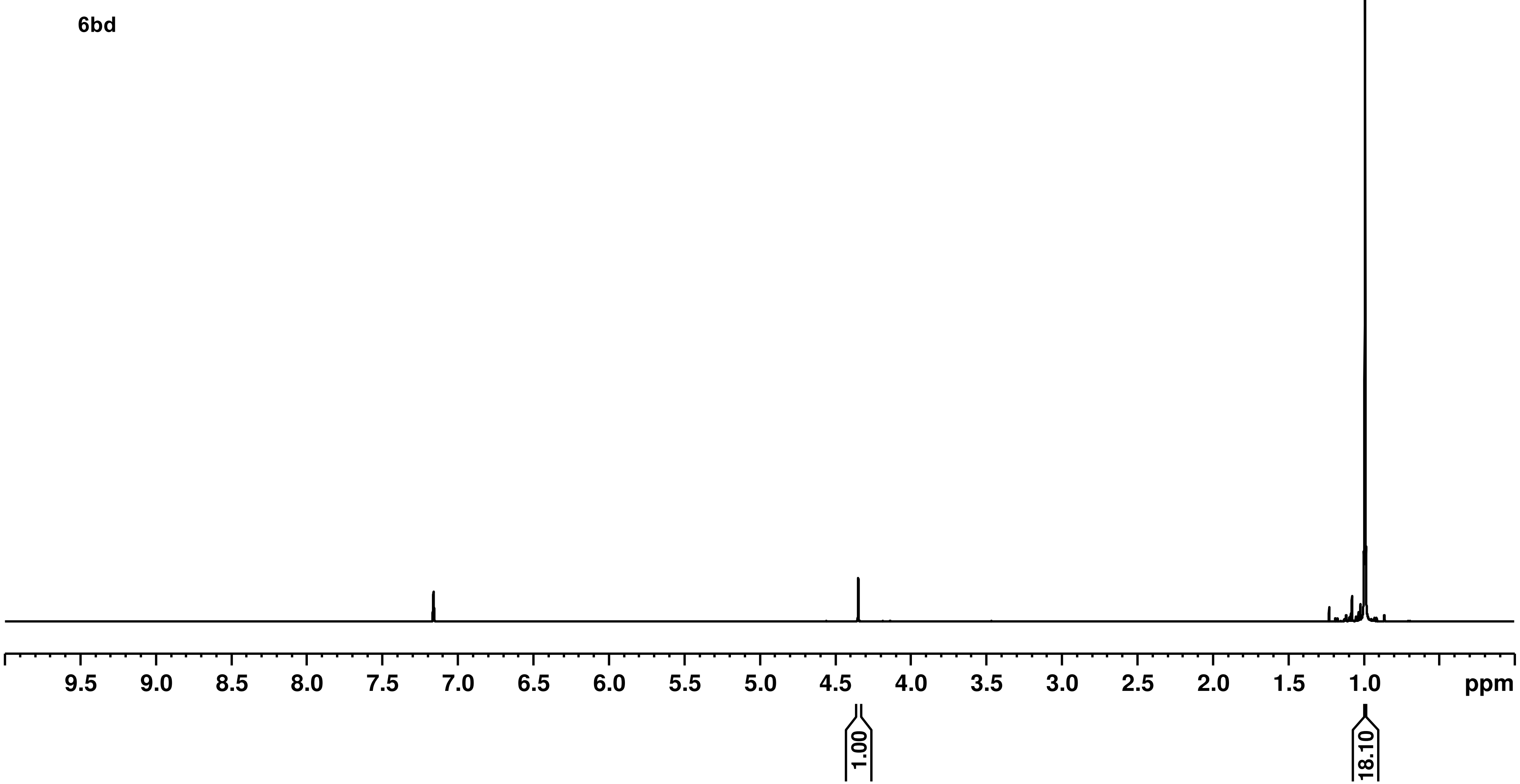

Supplementary Fig. 90. **^{13}C{^{1}H} NMR** spectrum (126 MHz, C_6D_6, 298 K) of di-*tert*-butylchlorosilane (**6bd**)

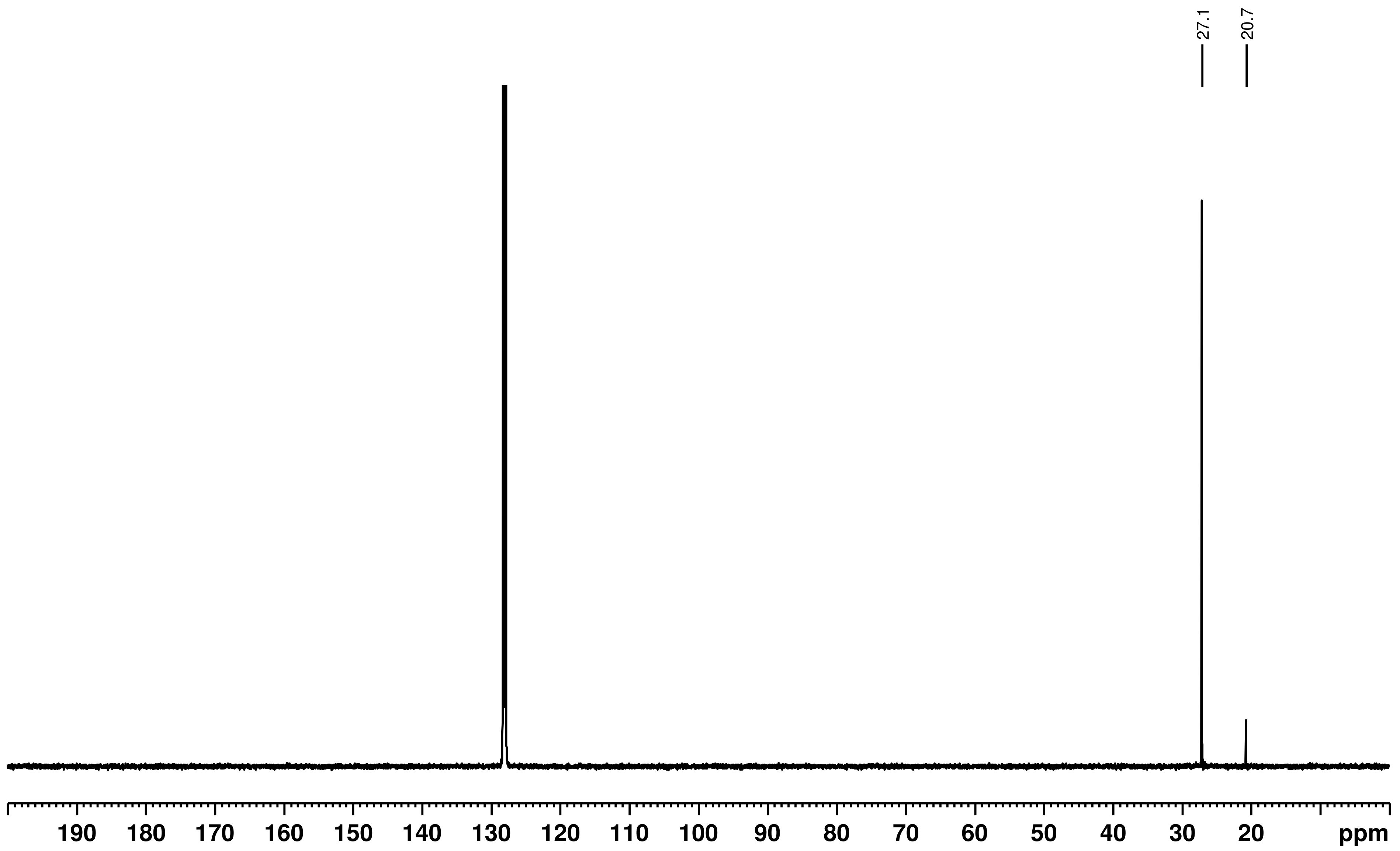

Supplementary Fig. 91. ^{29}**Si{^{1}H} DEPT NMR** spectrum (99 MHz, C_6D_6, 298 K, optimized for $J_{H,Si}$ = 200 Hz, 90.0°) of di-*tert*-butylchlorosilane (**6bd**)

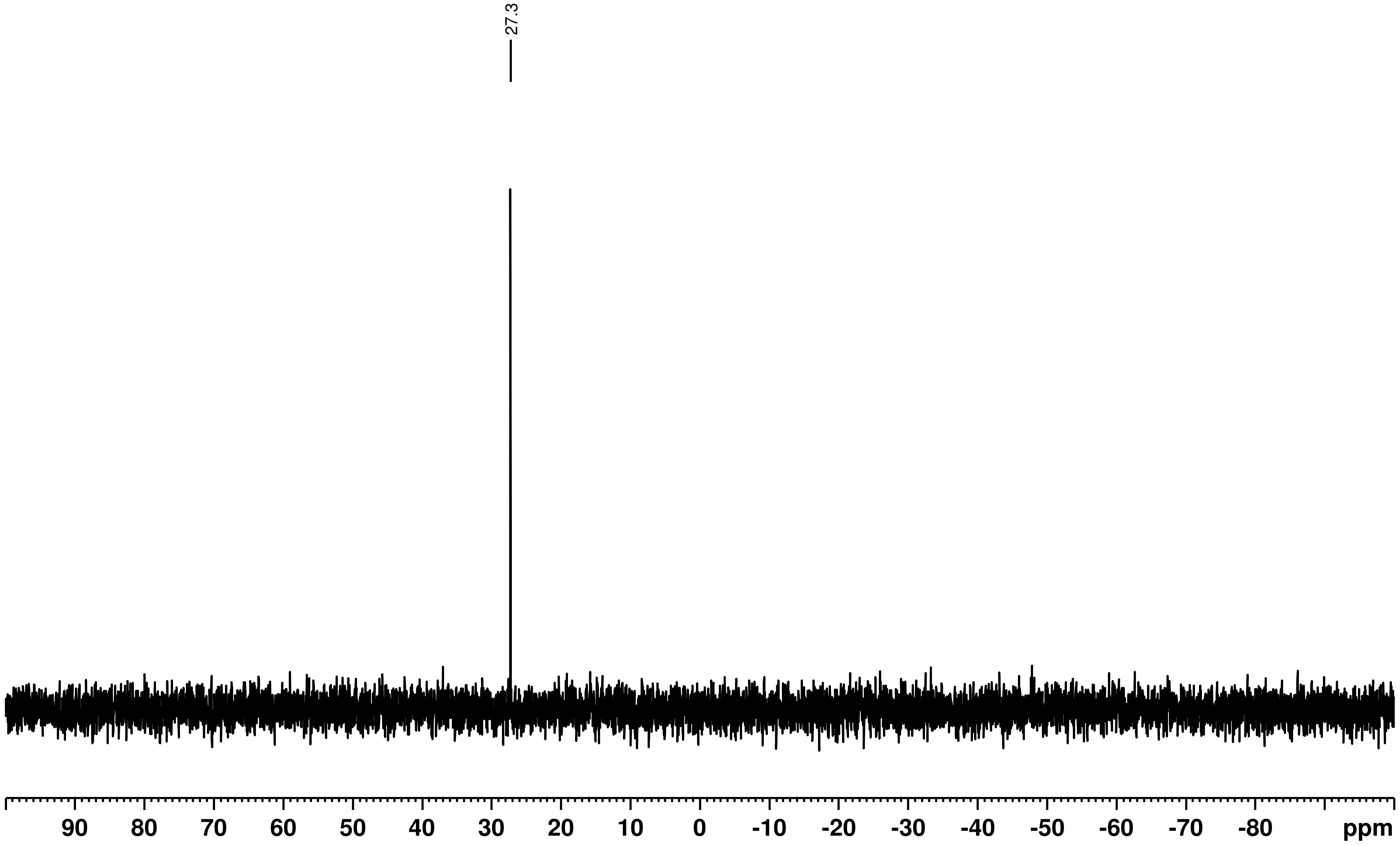

Supplementary Fig. 92. **IR** spectrum (ATR) of di-*tert*-butylchlorosilane (**6bd**)

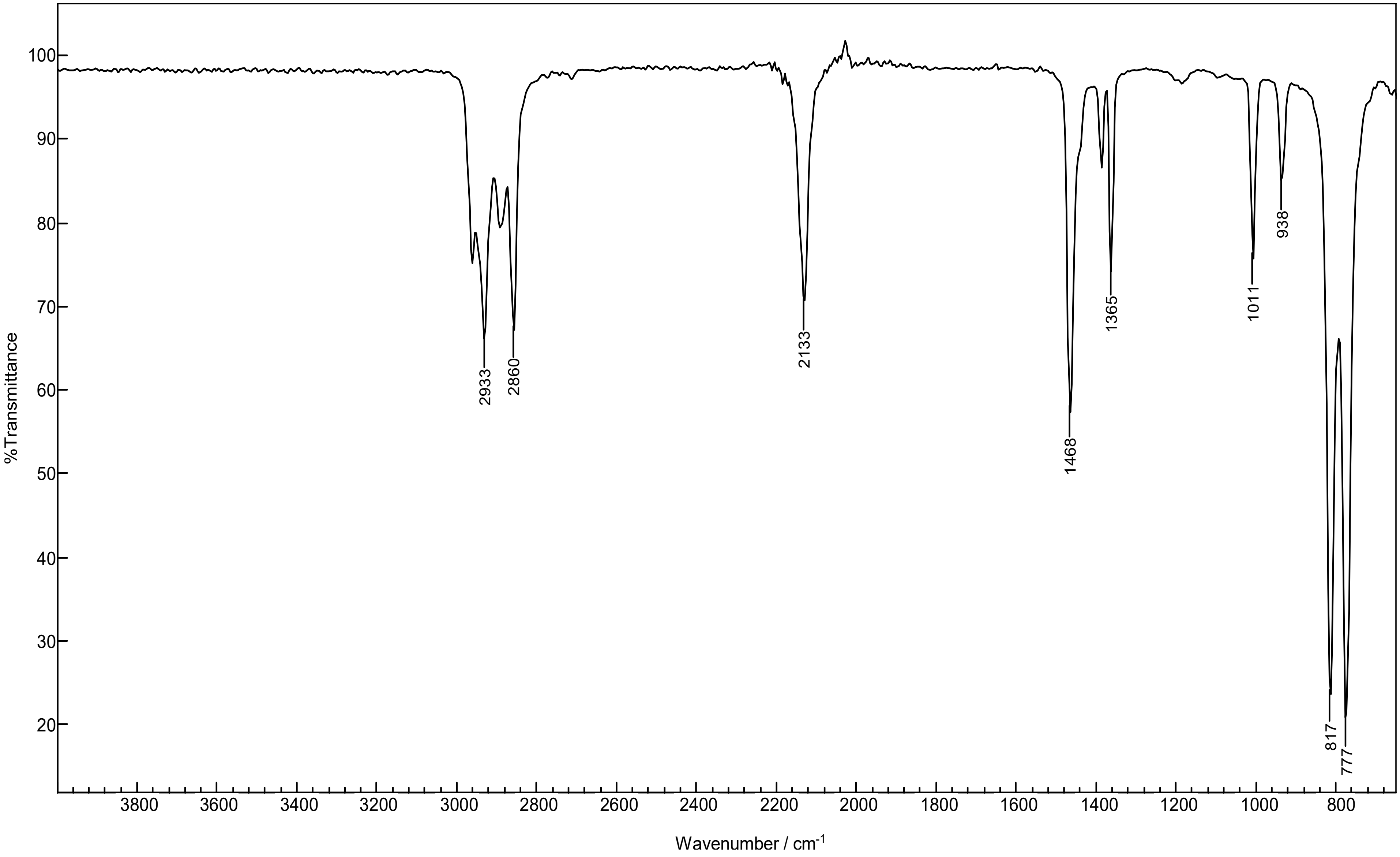

Supplementary Fig. 93. **^{1}H NMR** spectrum (500 MHz, C_6D_6, 298 K) of bromodi-*tert*-butylsilane (**6cd**)

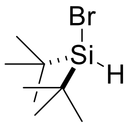

6cd

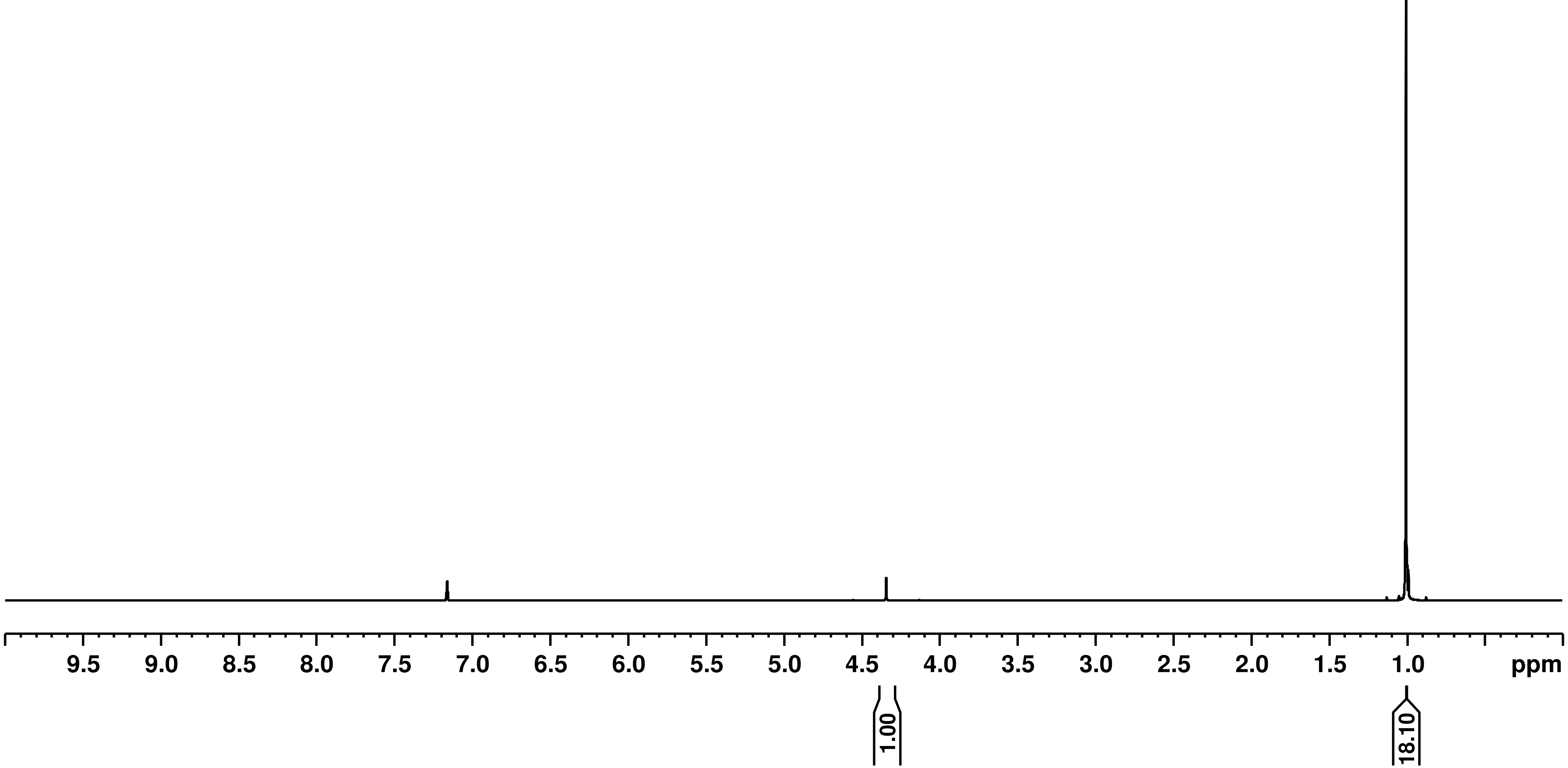

Supplementary Fig. 94. **^{13}C{^{1}H} NMR** spectrum (126 MHz, C_6D_6, 298 K) of bromodi-*tert*-butylsilane (**6cd**)

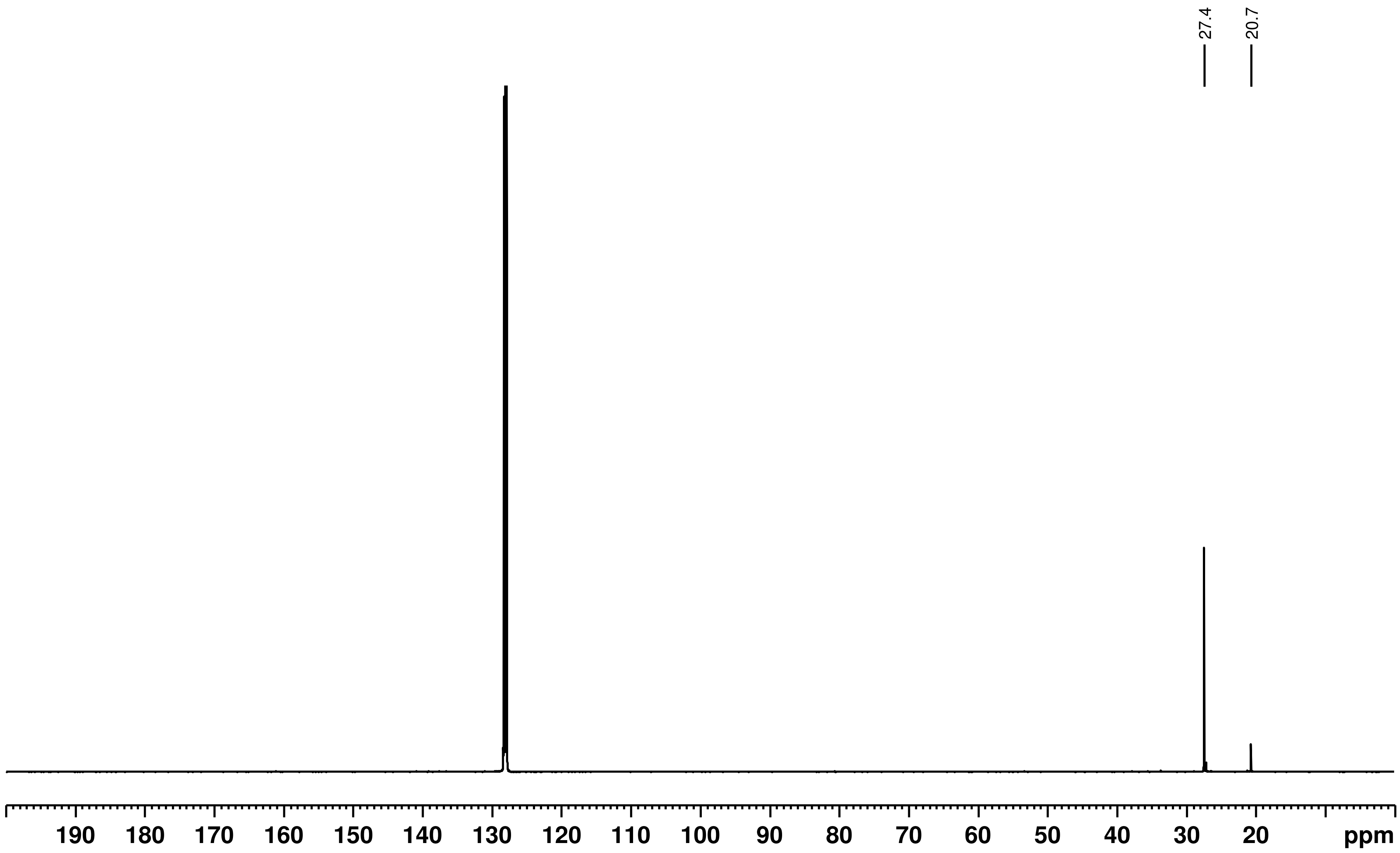

Supplementary Fig. 95. **^{29}Si{^{1}H} DEPT NMR** spectrum (99 MHz, C_6D_6, 298 K, optimized for $J_{H,Si}$ = 7 Hz, 13.6°) of bromodi-*tert*-butylsilane (**6cd**)

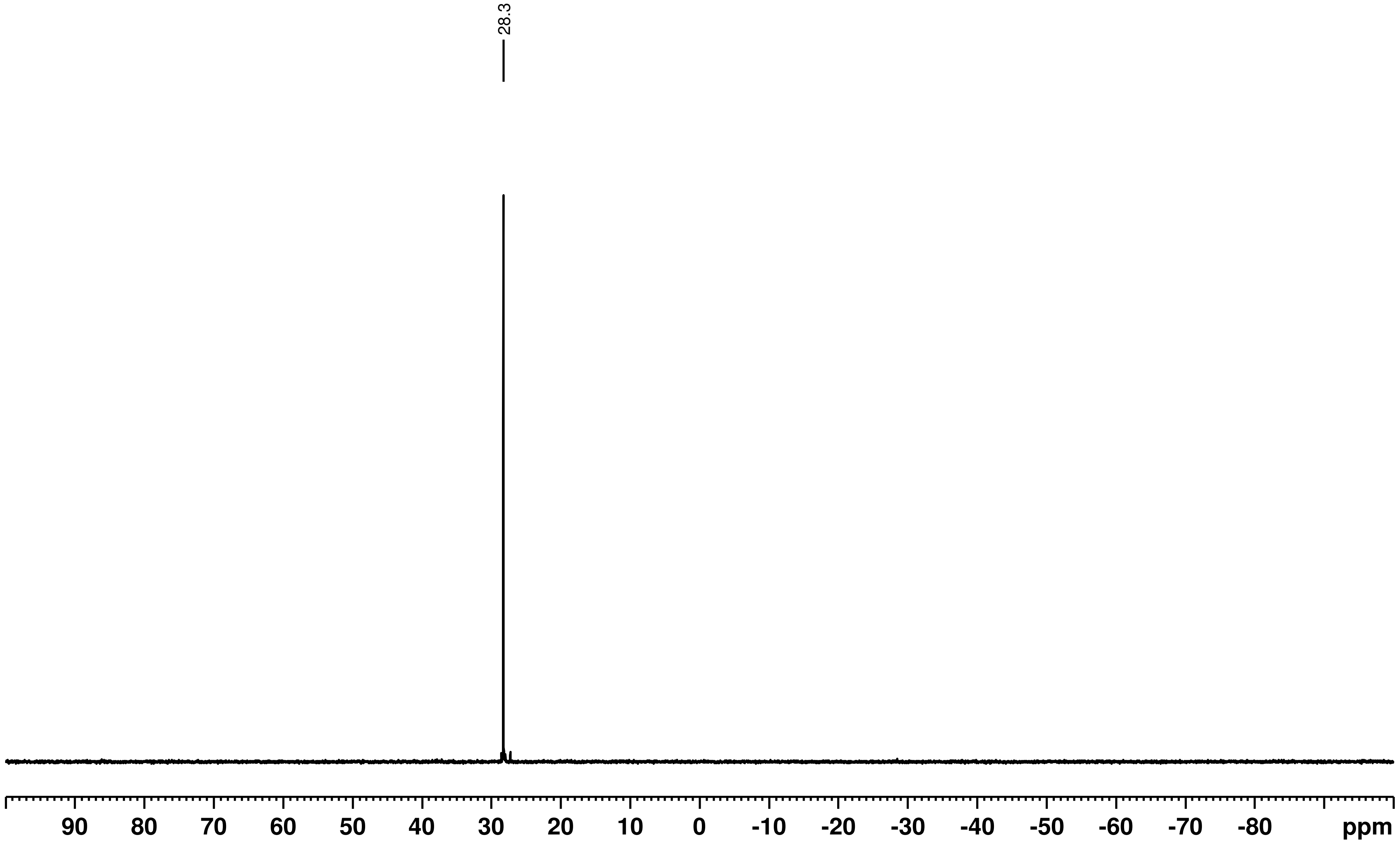

Supplementary Fig. 96. **IR** spectrum (ATR) of bromodi-*tert*-butylsilane (**6cd**)

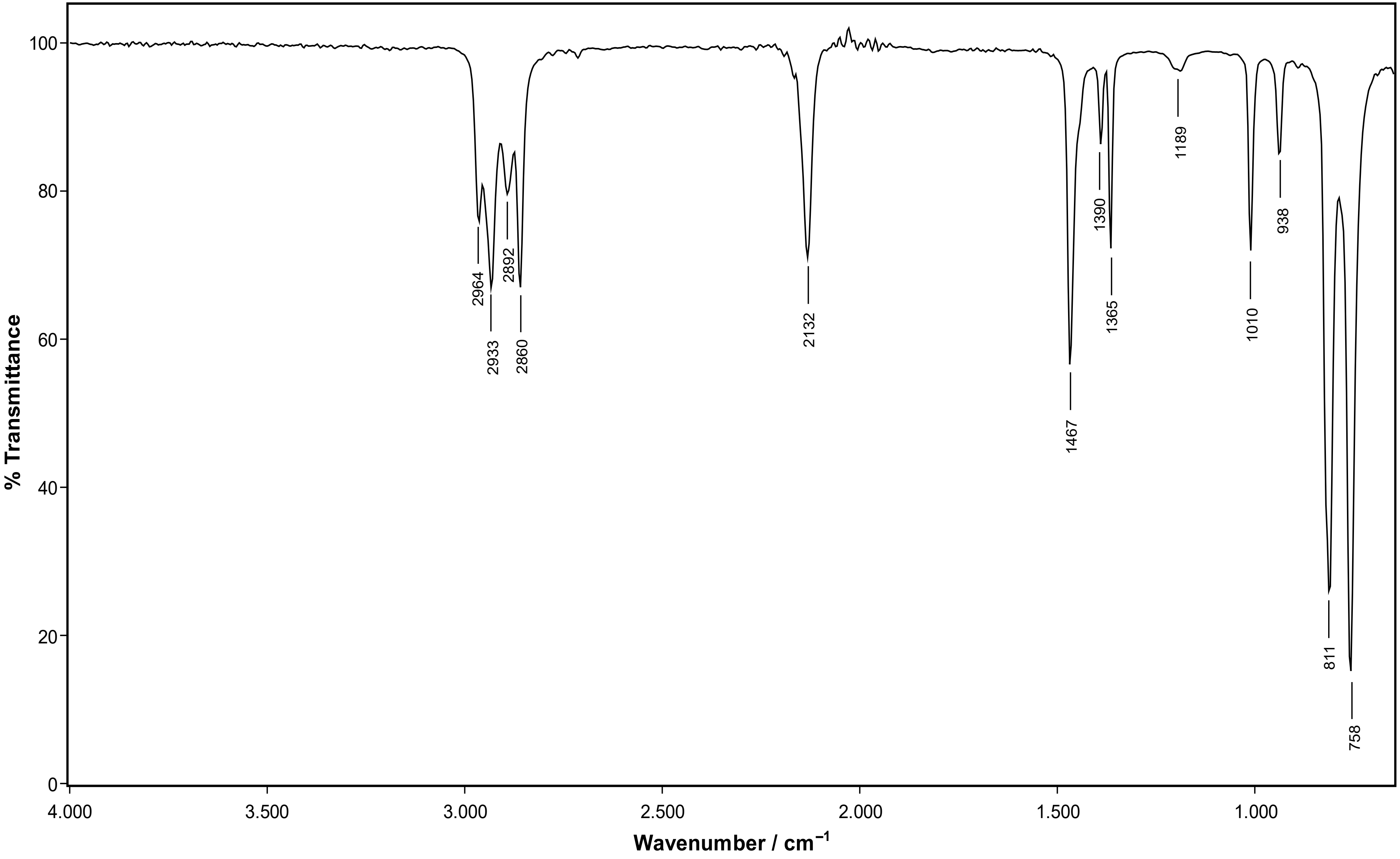

Supplementary Fig. 97. **^{1}H NMR** spectrum (500 MHz, C_6D_6, 298 K) of di-*tert*-butyliodosilane (**6dd**)

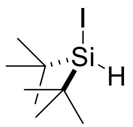

6dd

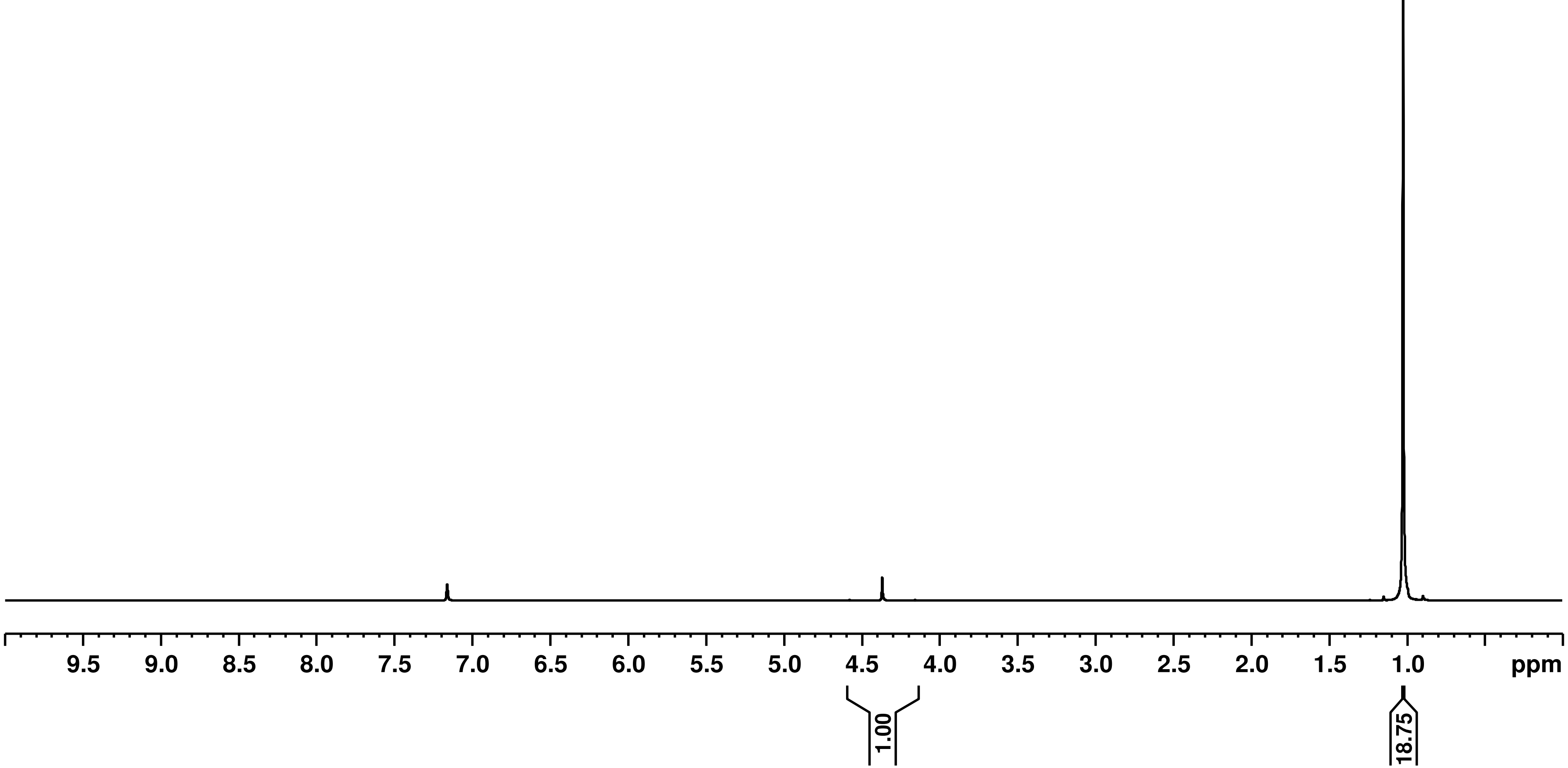

Supplementary Fig. 98. **^{13}C{^{1}H} NMR** spectrum (126 MHz, C_6D_6, 298 K) of di-*tert*-butyliodosilane (**6dd**)

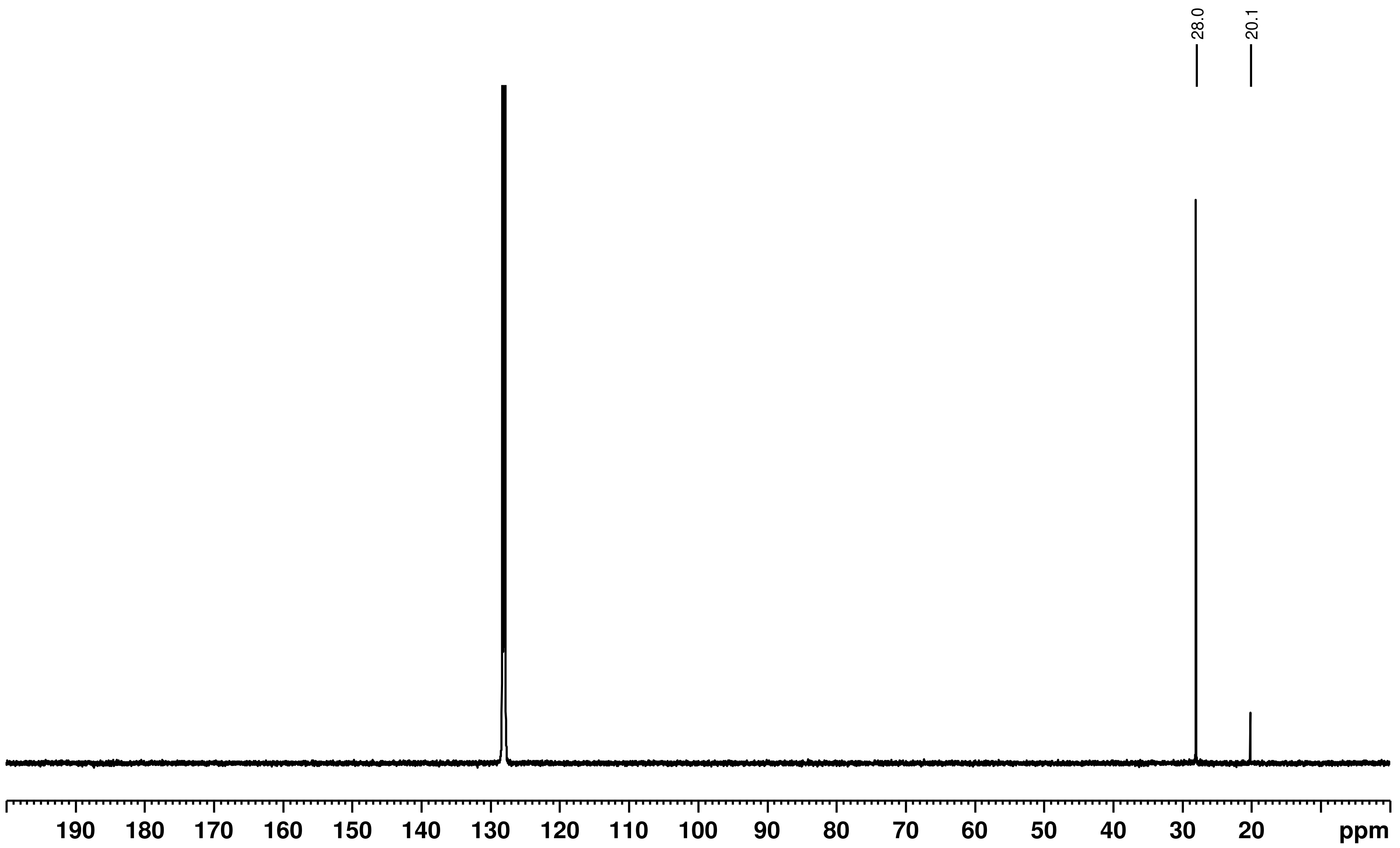

Supplementary Fig. 99. ^{29}**Si{^{1}H} DEPT NMR** spectrum (99 MHz, C_6D_6, 298 K, optimized for $J_{H,Si}$ = 7 Hz, 13.6°) of di-*tert*-butyliodosilane (**6dd**)

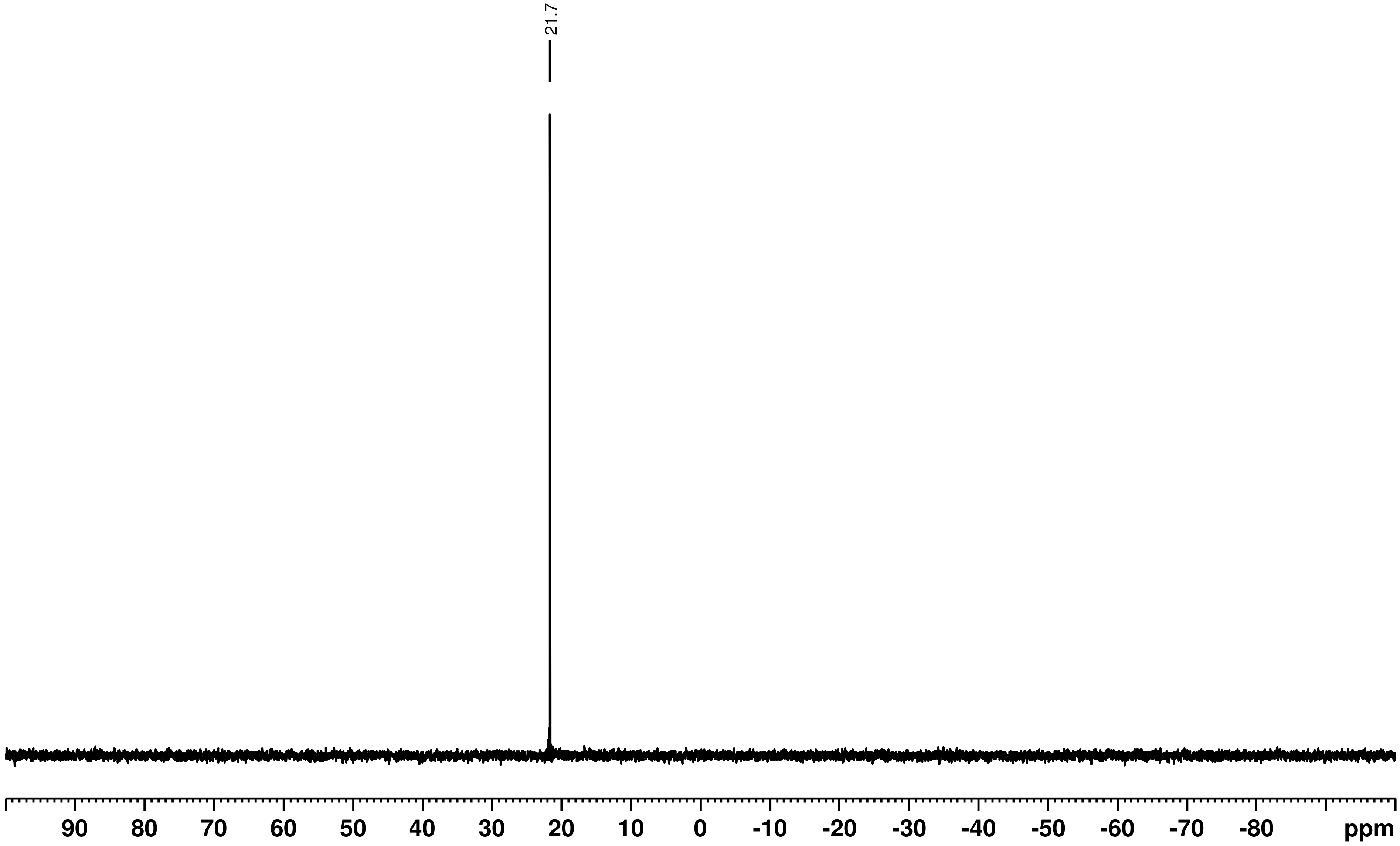

Supplementary Fig. 100. **^{1}H NMR** spectrum (500 MHz, C_6D_6, 298 K) of difluoromethyl(phenyl)silane (**S11**)

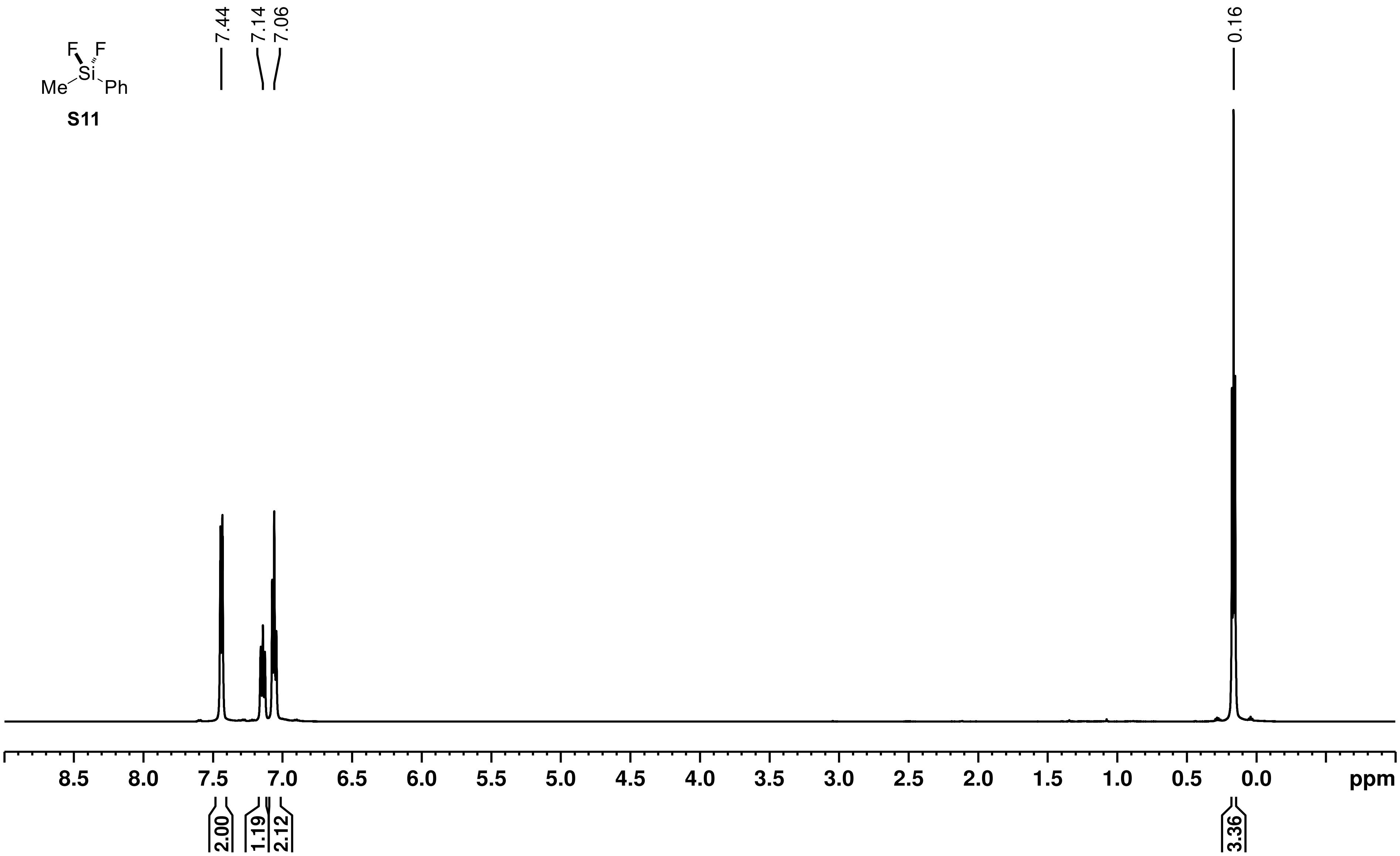

Supplementary Fig. 101. **^{13}C{^{1}H} NMR** spectrum (126 MHz, C_6D_6, 298 K) of difluoromethyl(phenyl)silane (**S11**)

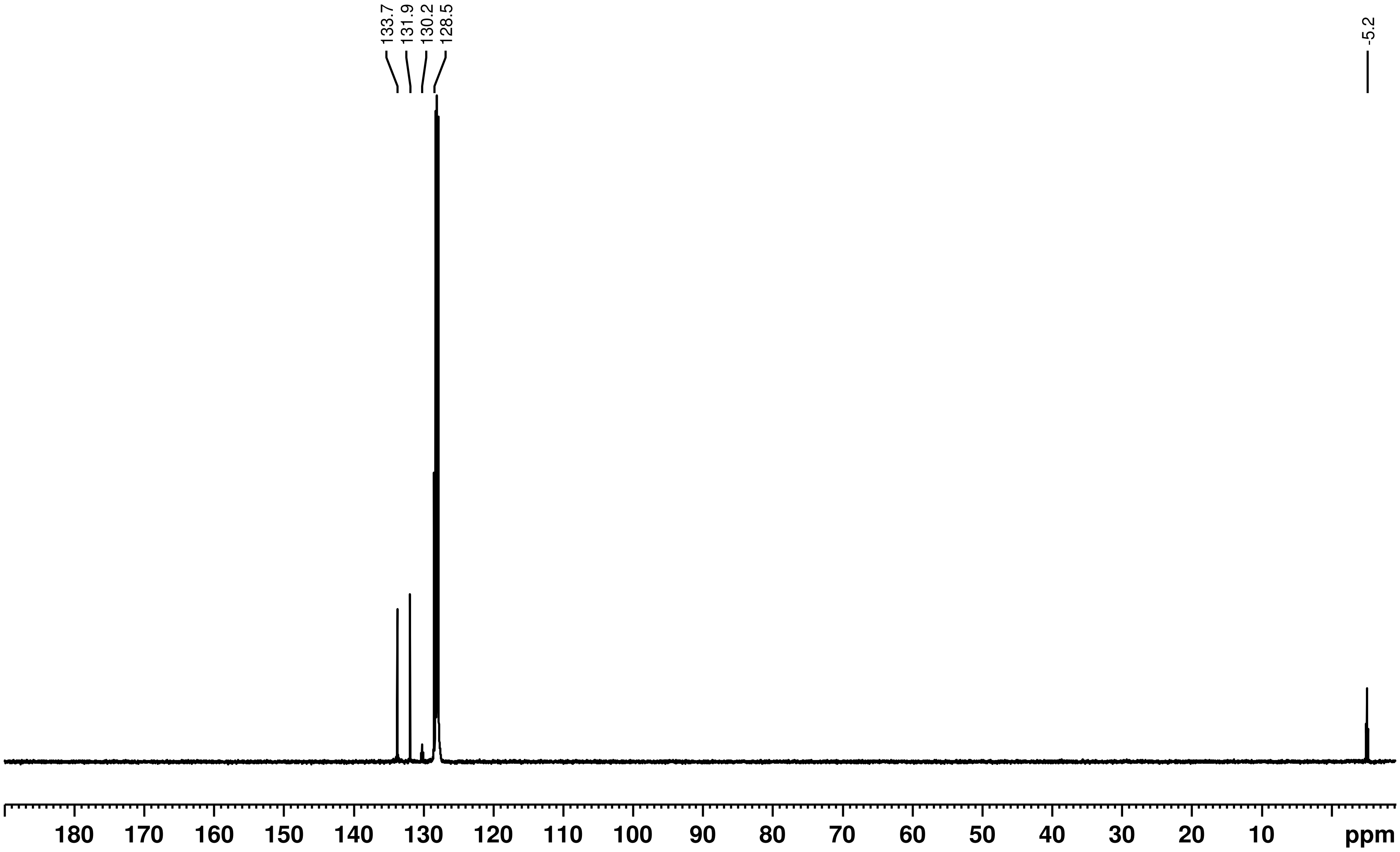

Supplementary Fig. 102. **^{19}F NMR** spectrum (471 MHz, C_6D_6, 298 K) of difluoromethyl(phenyl)silane (**S11**)

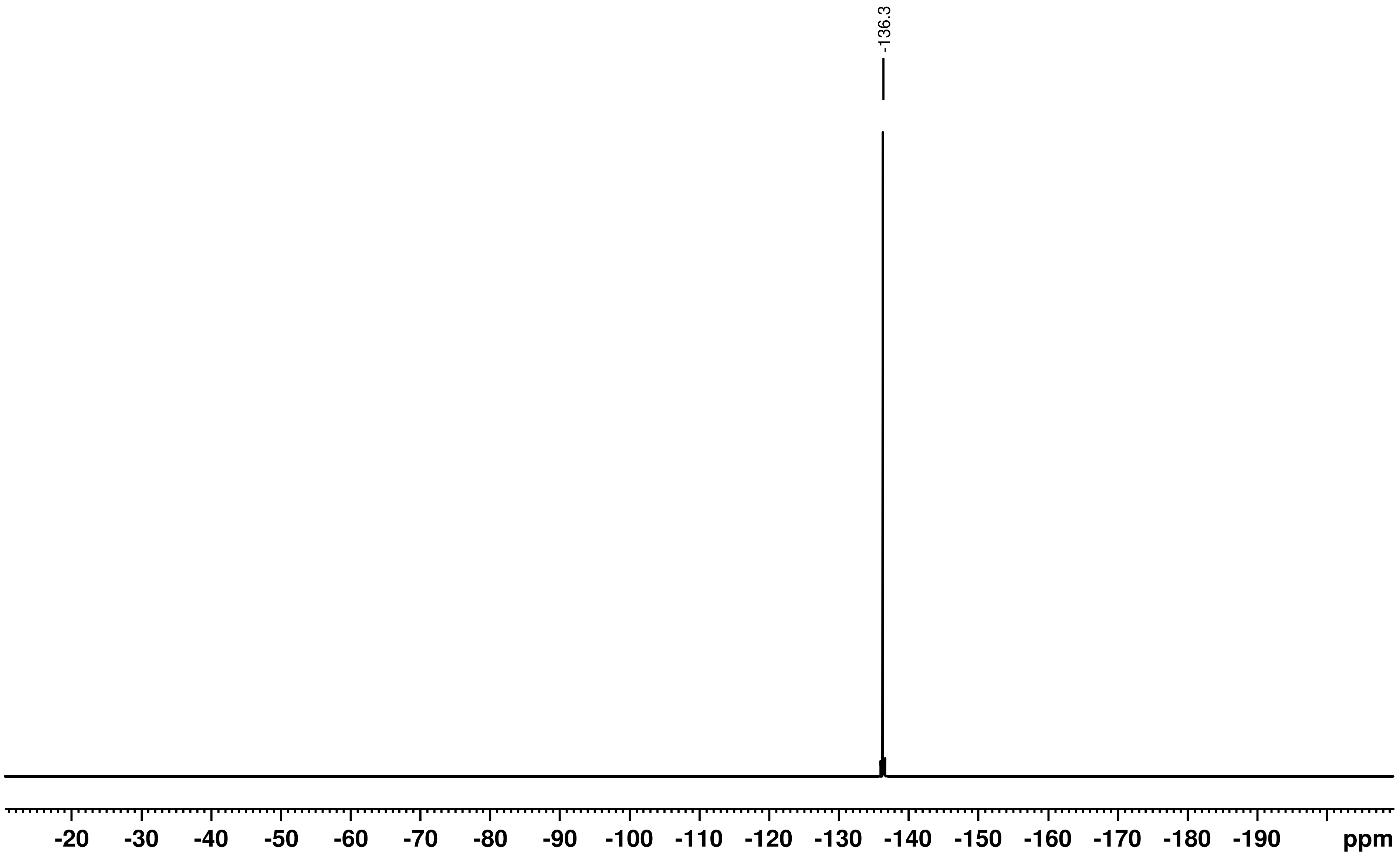

Supplementary Fig. 103. **^{29}Si{^{1}H} IG NMR** spectrum (99 MHz, C_6D_6, 298 K) of difluoromethyl(phenyl)silane (**S11**)

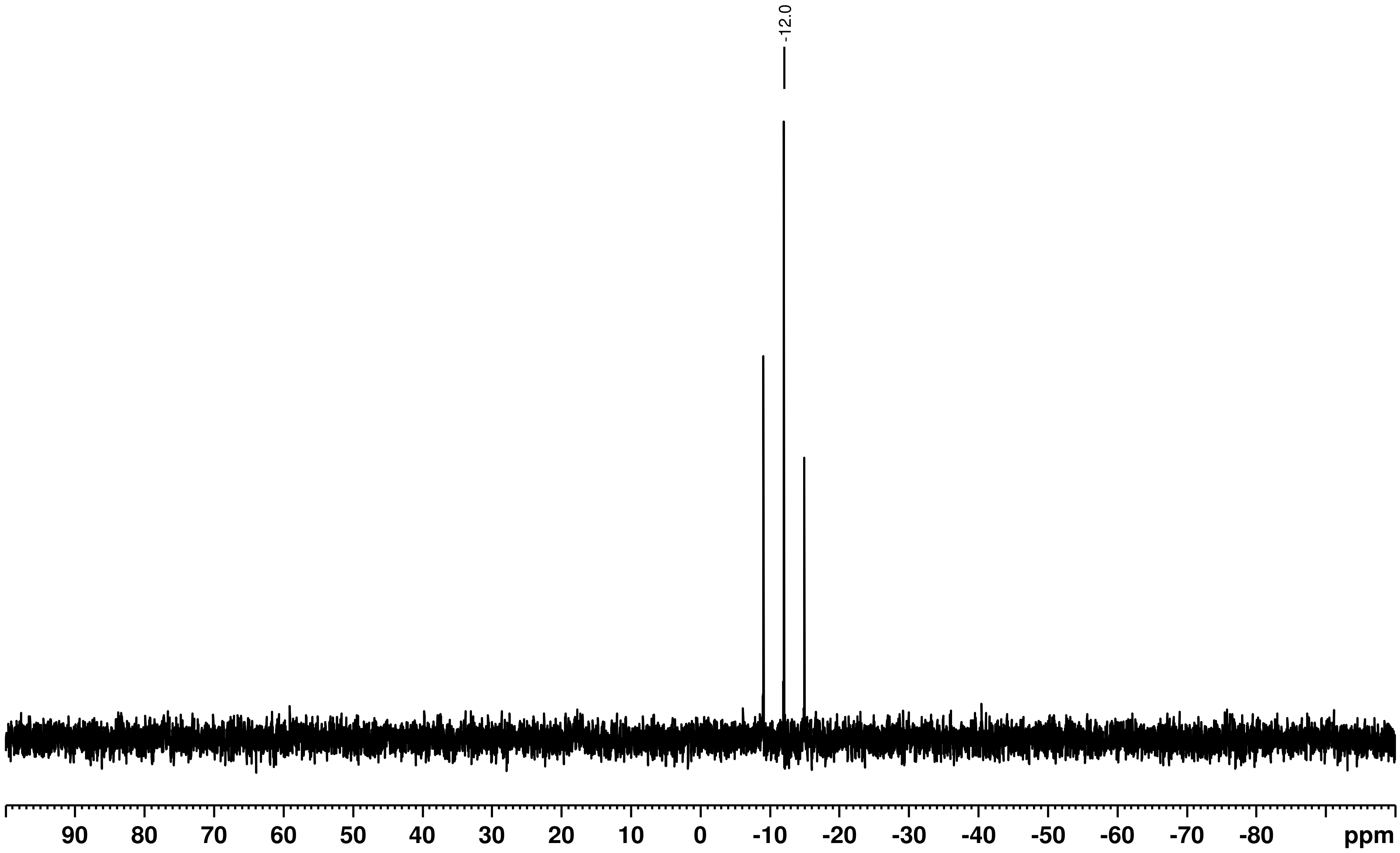

Supplementary Fig. 104. **IR** spectrum (ATR) of difluoromethyl(phenyl)silane (**S11**)

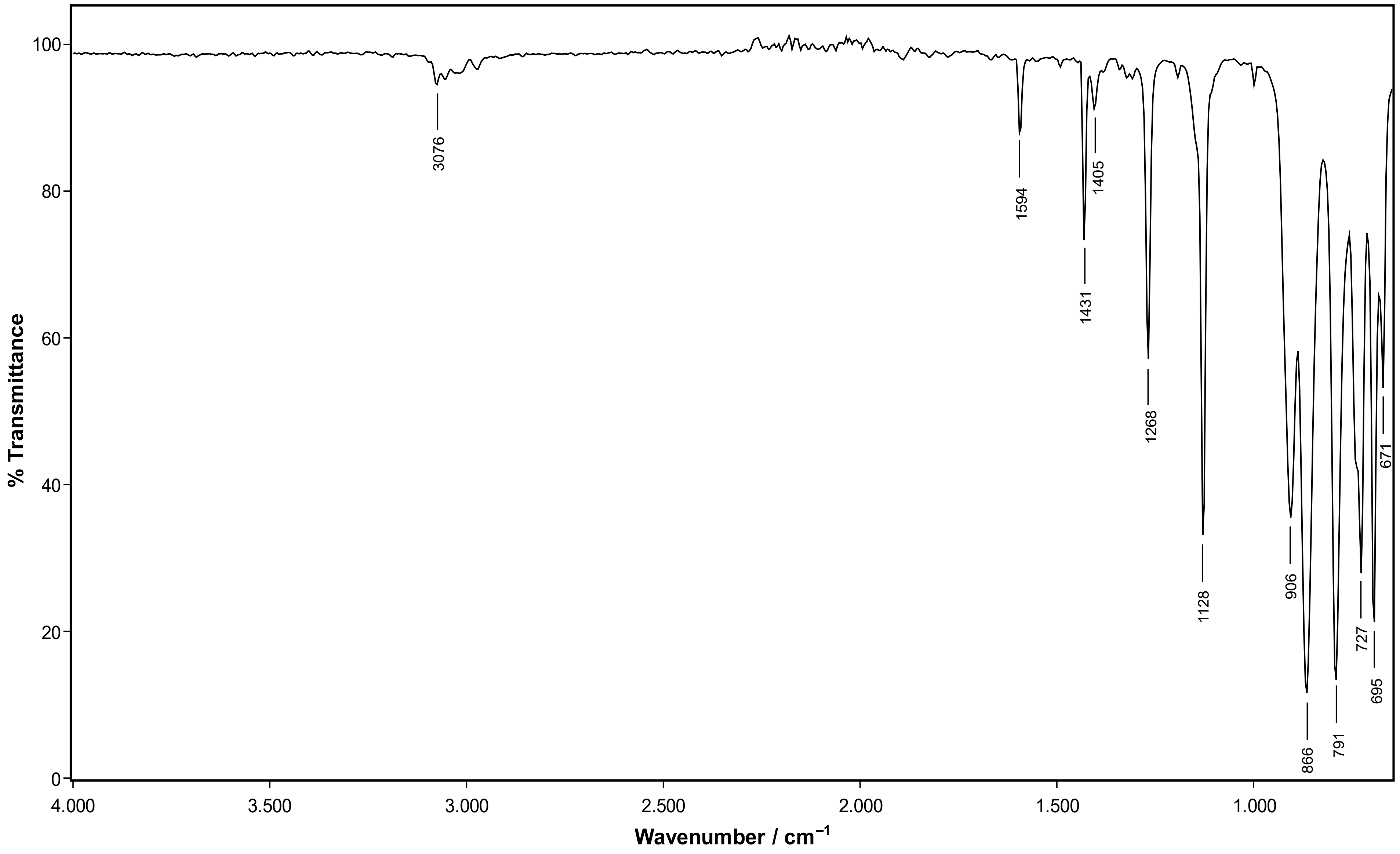

Supplementary Fig. 105. **GLC-MS** spectrum (EI) of difluoromethyl(phenyl)silane (**S11**)

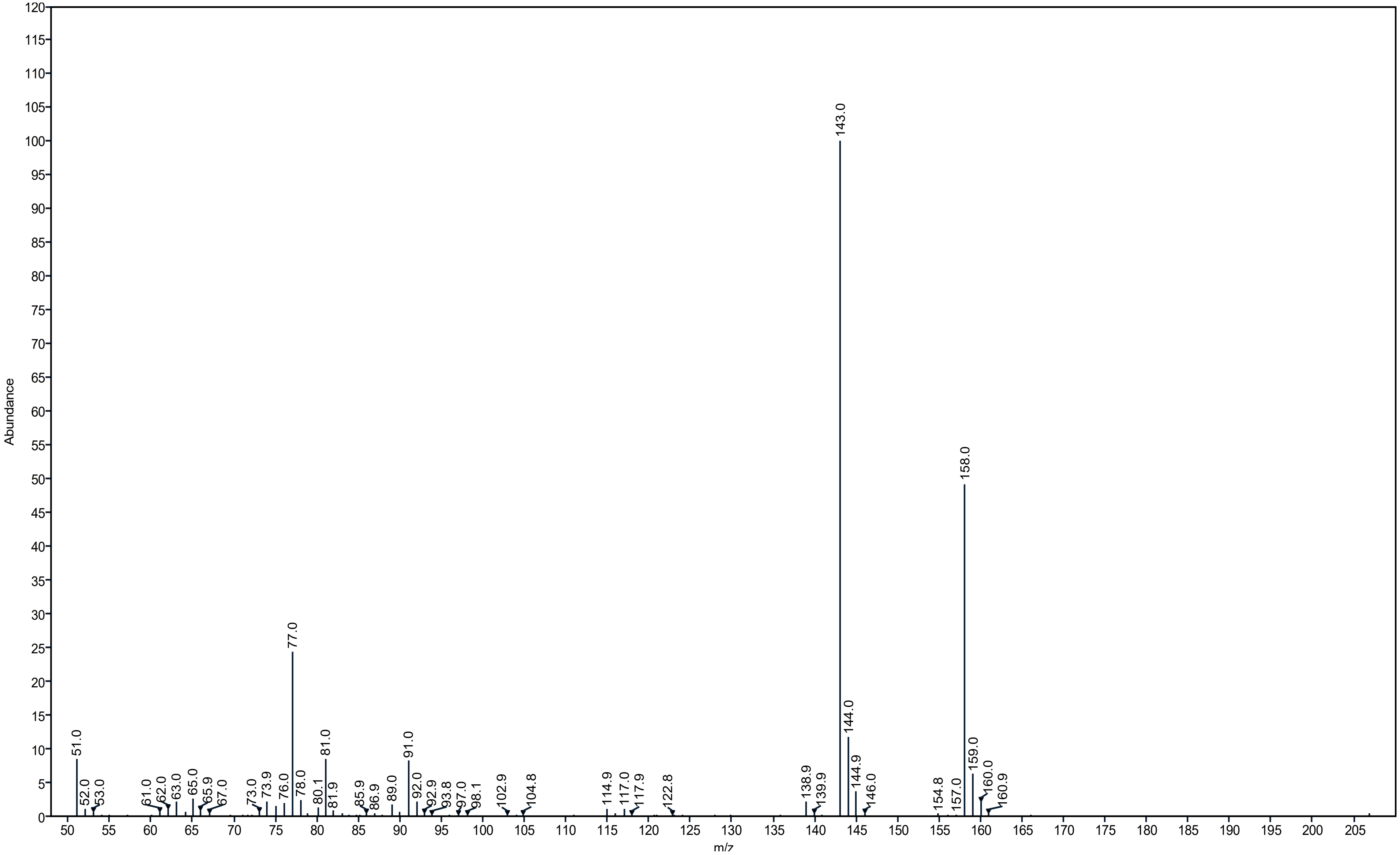

Supplementary Fig. 106. ^{1}**H NMR** spectrum (500 MHz, CD_2Cl_2, 298 K) of dichloroisopropylsilane (**S12**)

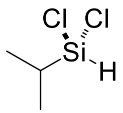

S12

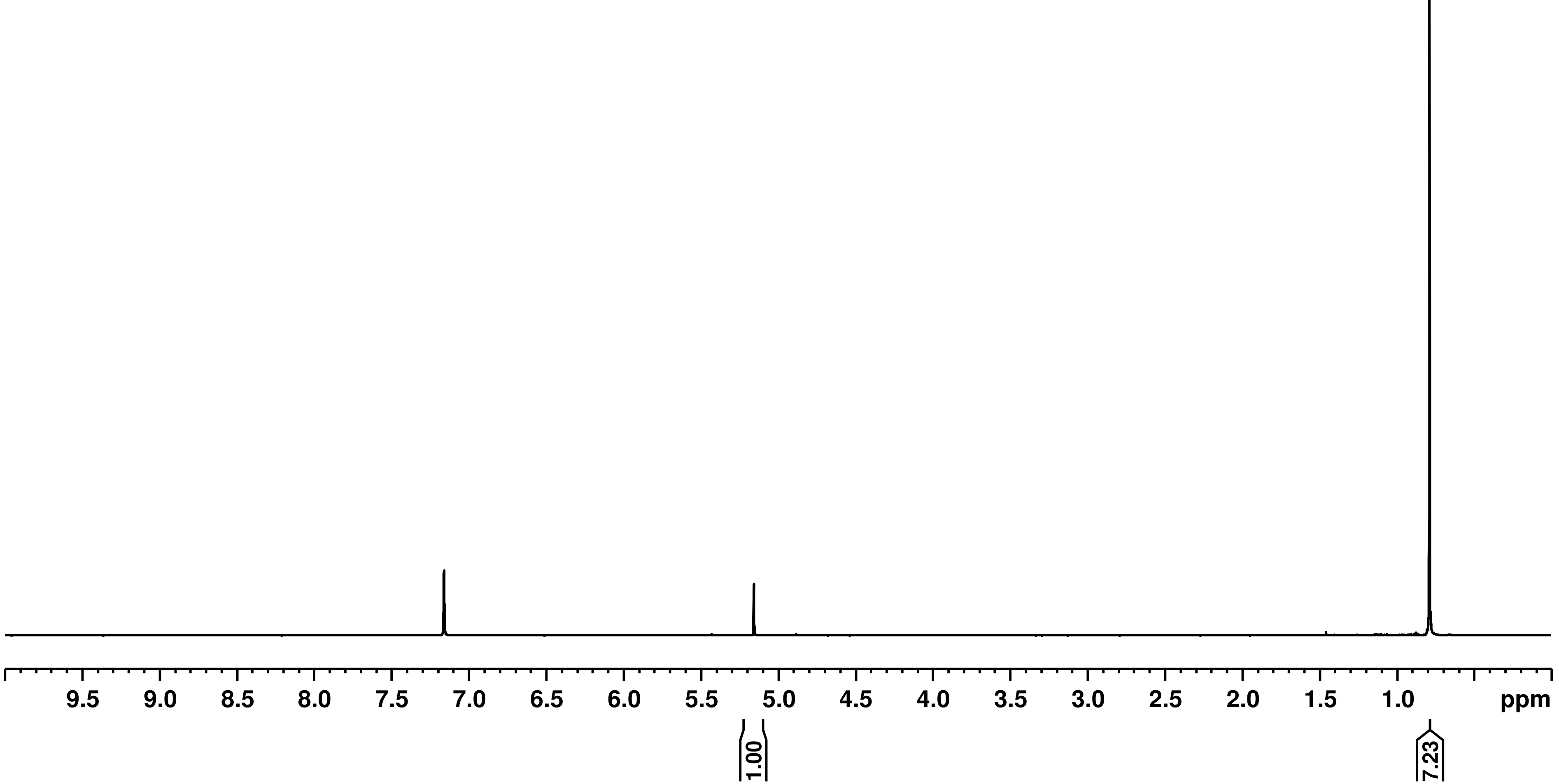

Supplementary Fig. 107. **$^{13}C\{^{1}H\}$ NMR** spectrum (126 MHz, C_6D_6, 298 K) of dichloroisopropylsilane (**S12**)

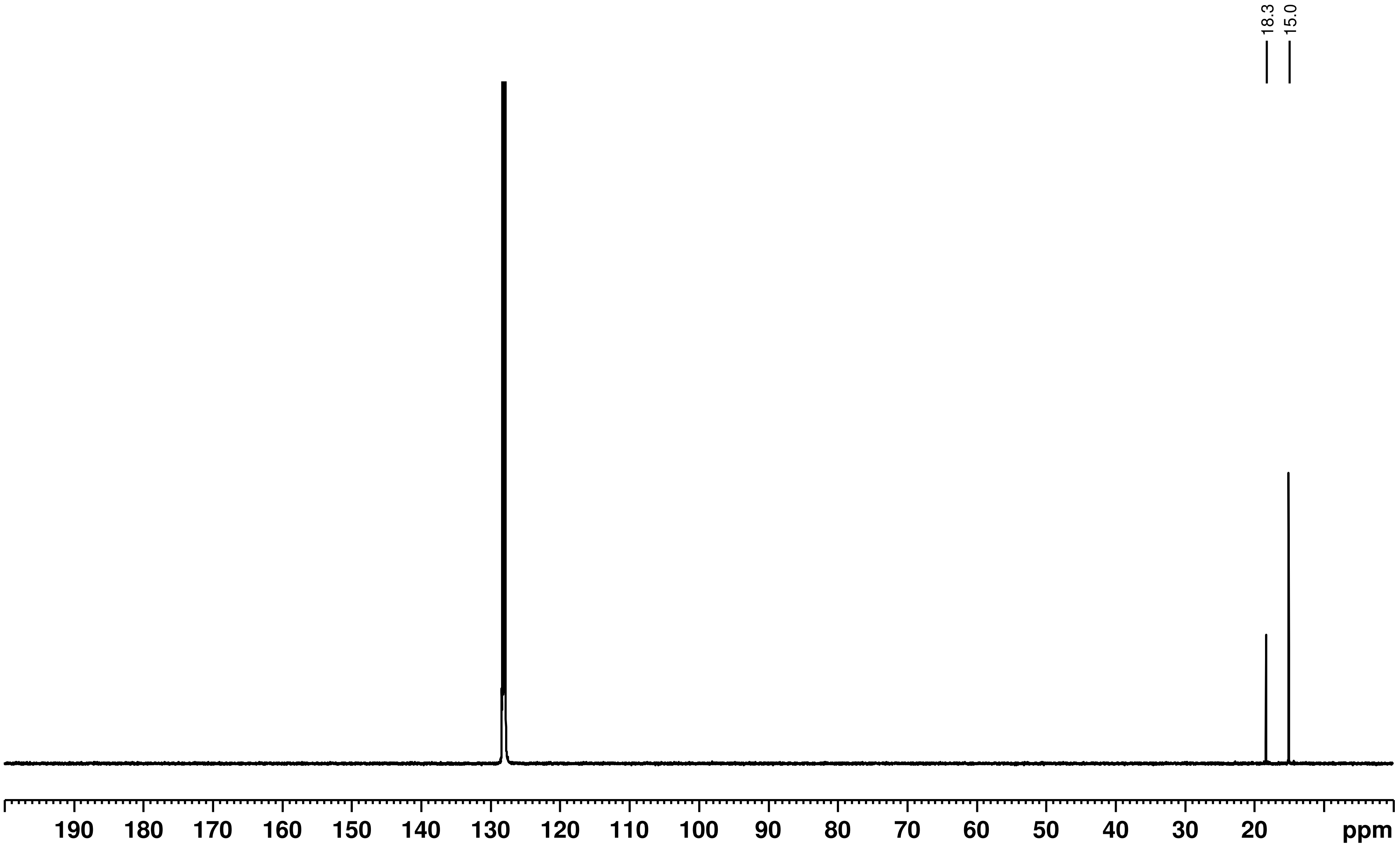

Supplementary Fig. 108. **$^{29}Si\{^{1}H\}$ DEPT NMR** spectrum (99 MHz, C_6D_6, 298 K, optimized for $J_{H,Si}$ = 7 Hz, 22.2°) of dichloroisopropylsilane (**S12**)

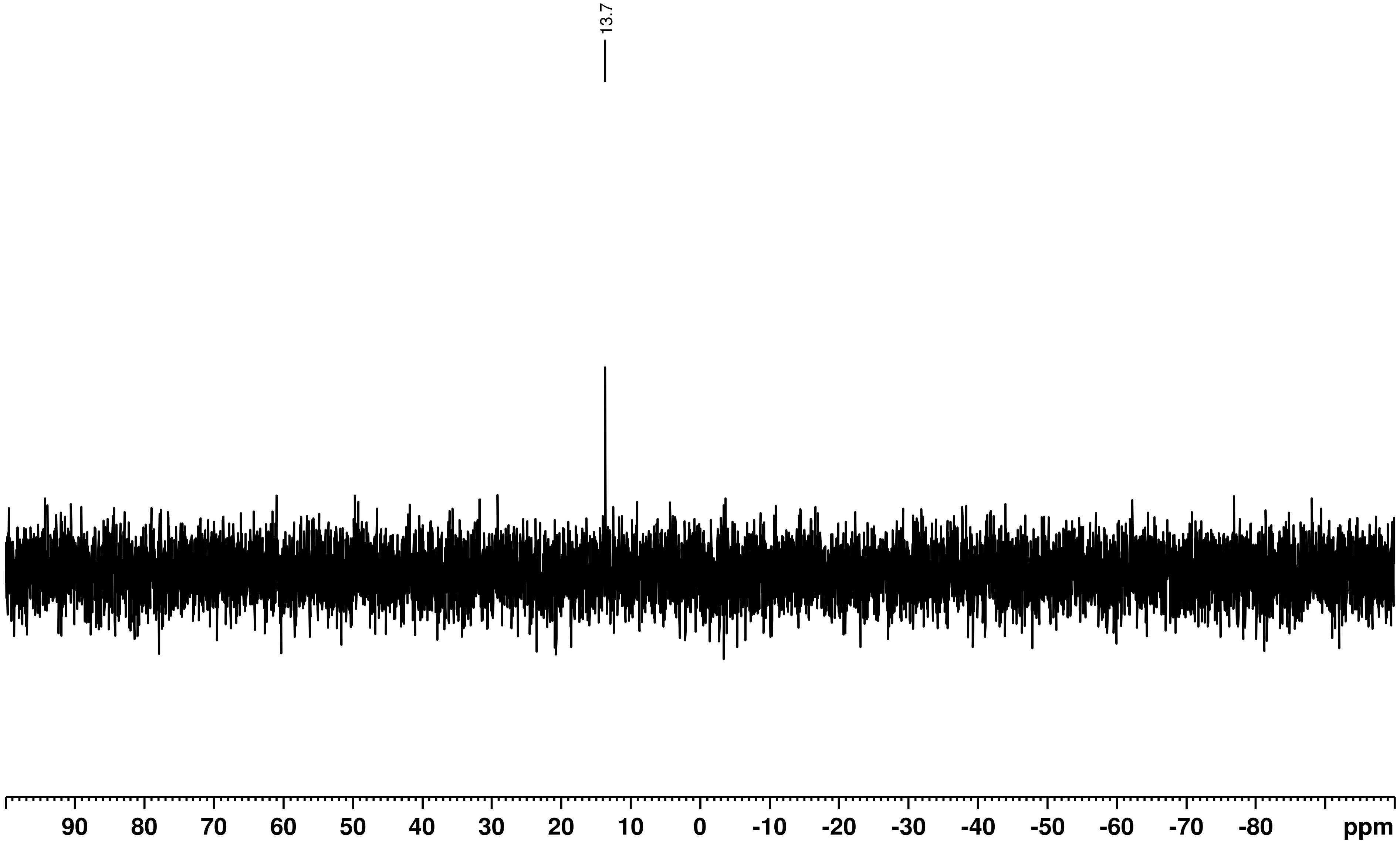

Supplementary Fig. 109. **IR** spectrum (ATR) of dichloroisopropylsilane (**S12**)

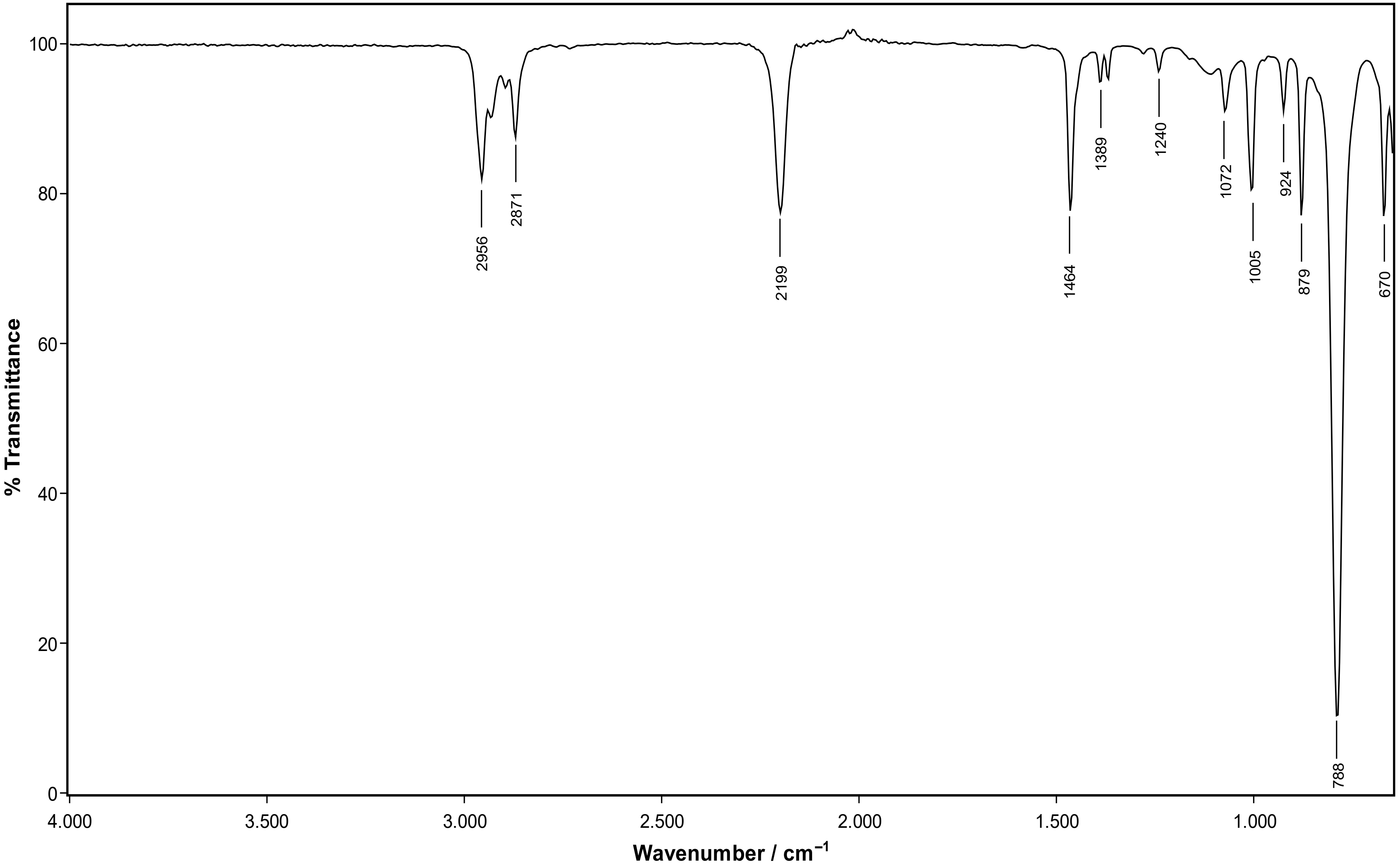

Supplementary Fig. 110. **^{1}H NMR** spectrum (500 MHz, C_6D_6, 298 K) of *tert*-butyldichloro(phenyl)silane (**S13**)

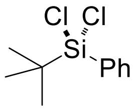

S13

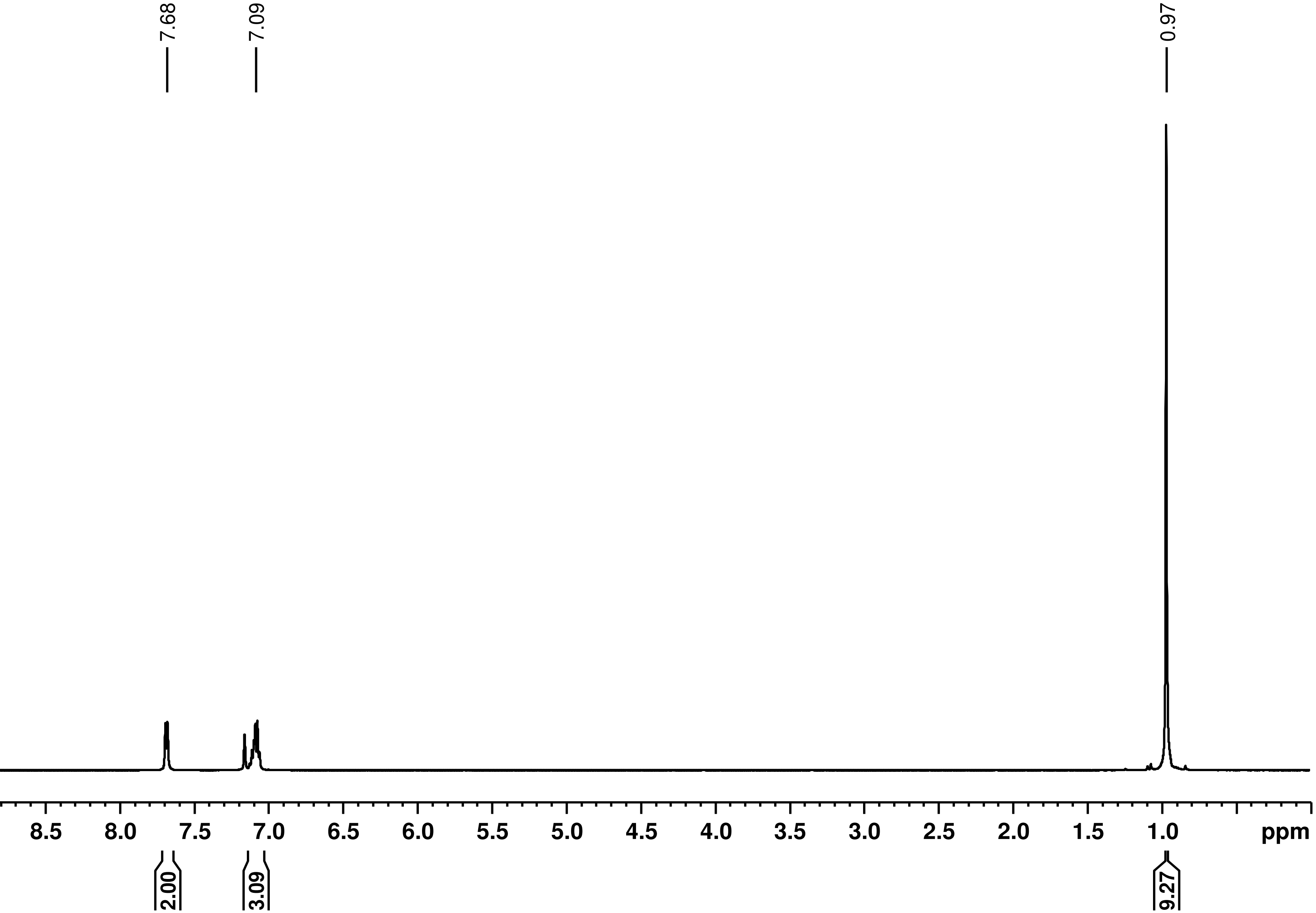

Supplementary Fig. 111. **^{13}C{^{1}H} NMR** spectrum (126 MHz, C_6D_6, 298 K) of *tert*-butyldichloro(phenyl)silane (**S13**)

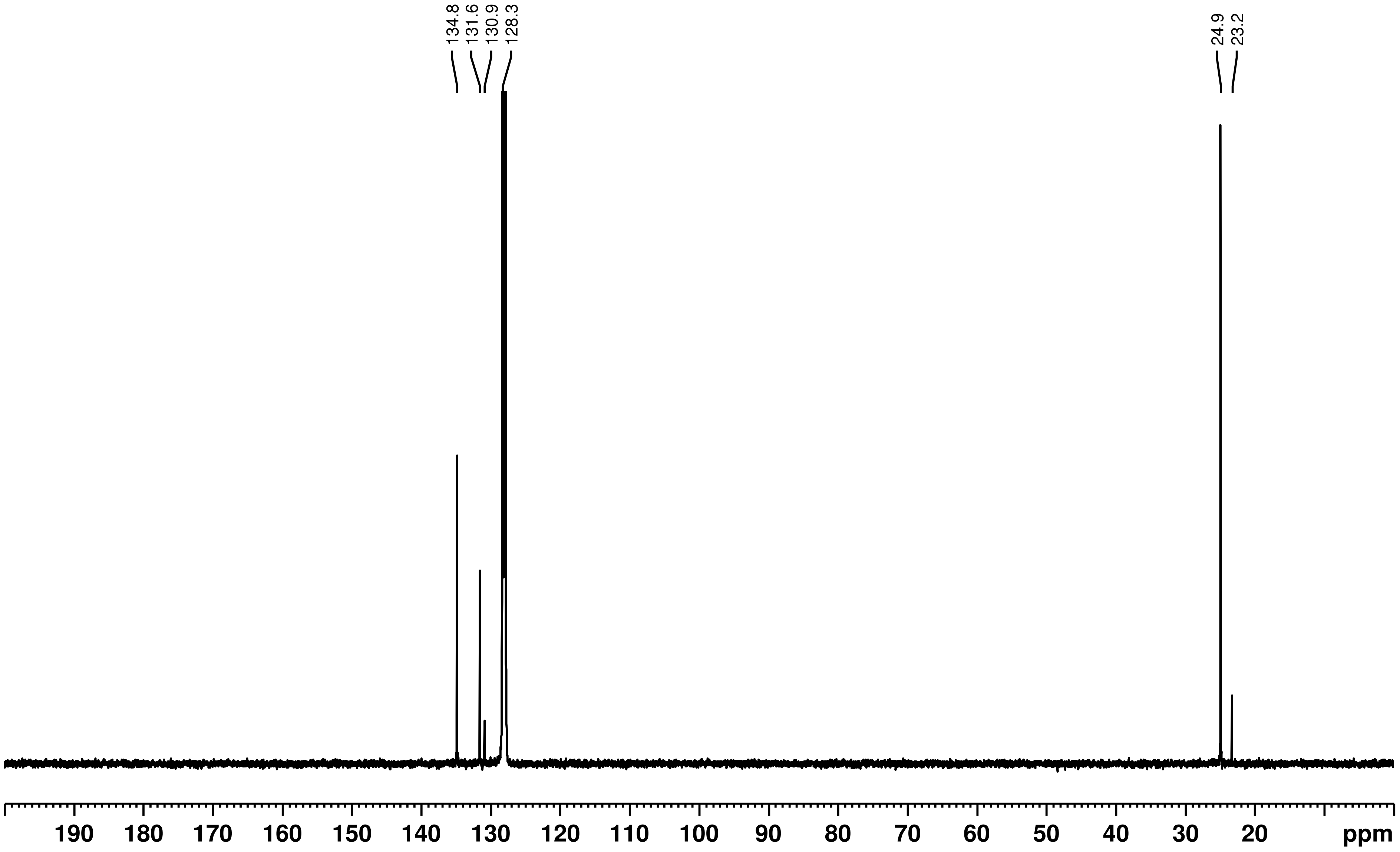

Supplementary Fig. 112. **^{29}Si{^{1}H} DEPT NMR** spectrum (99 MHz, C_6D_6, 298 K, optimized for $J_{H,Si}$ = 7 Hz, 17.5°) of *tert*-butyldichloro(phenyl)silane (**S13**)

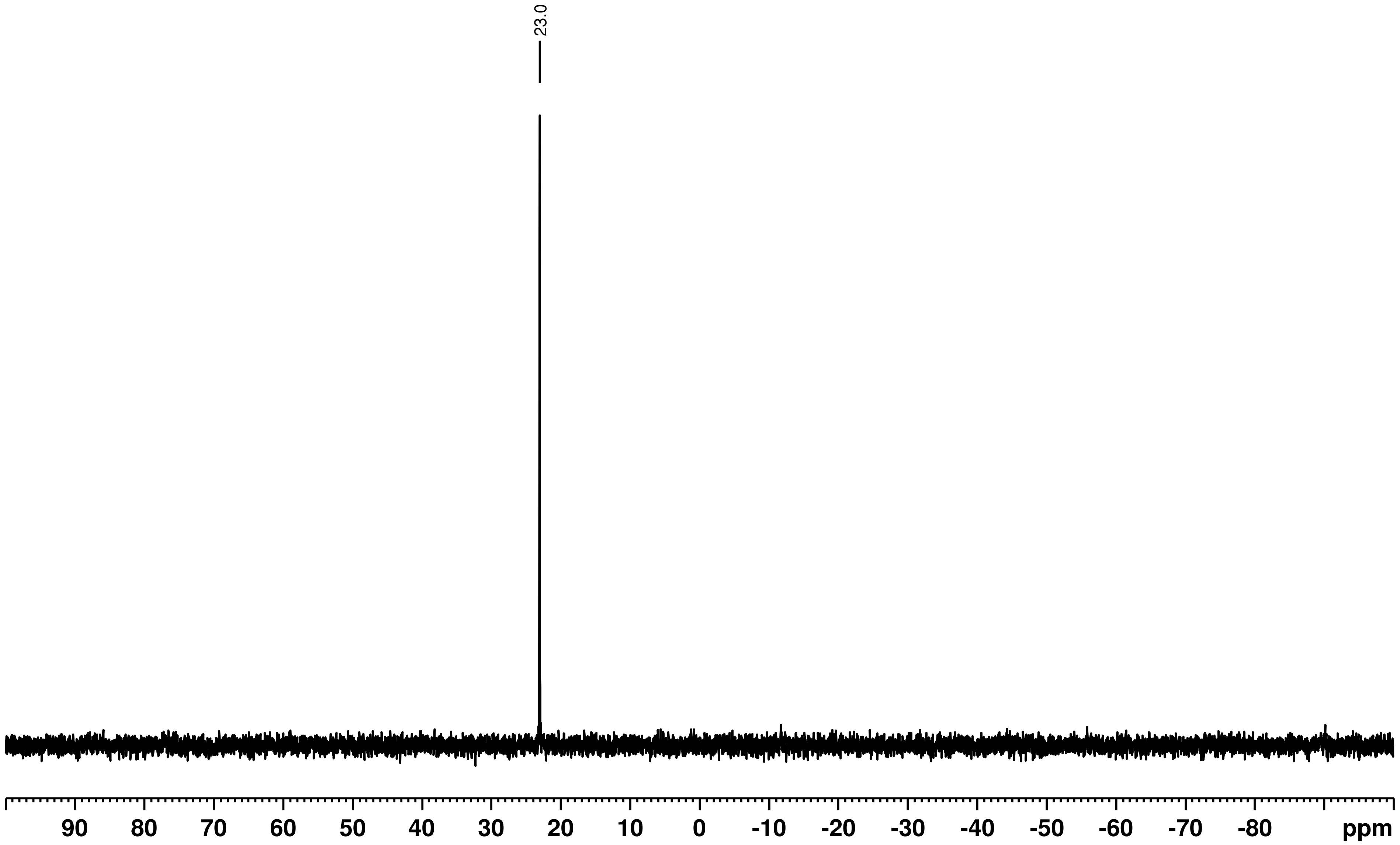

Supplementary Fig. 113. **IR** spectrum (ATR) of *tert*-butyldichlorophenylsilane (**S13**)

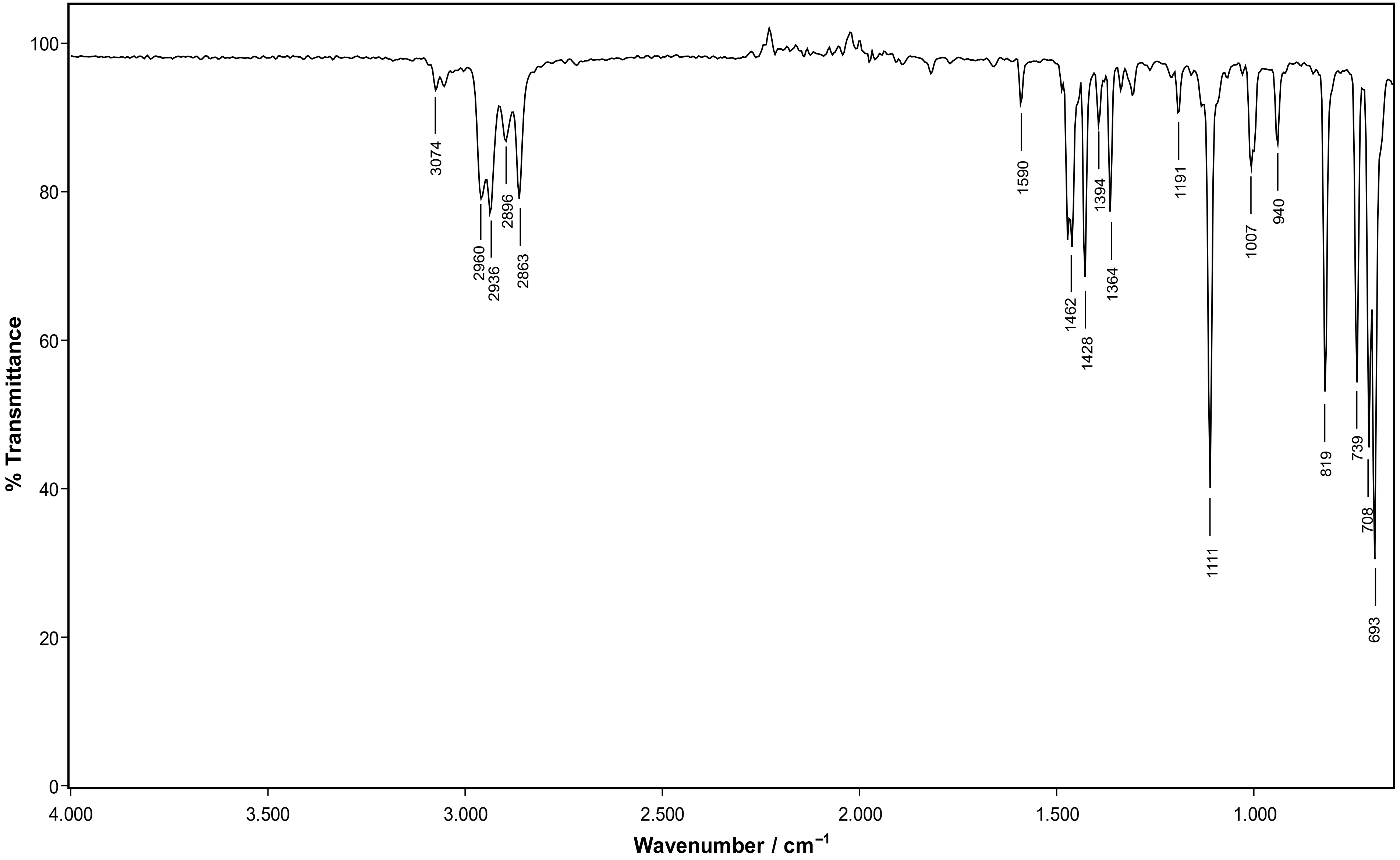

Supplementary Fig. 114. [1]**H NMR** spectrum (500 MHz, CD_2Cl_2, 298 K) of *tert*-butyldifluoro(phenyl)silane (**S14**)

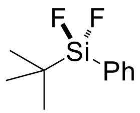

S14

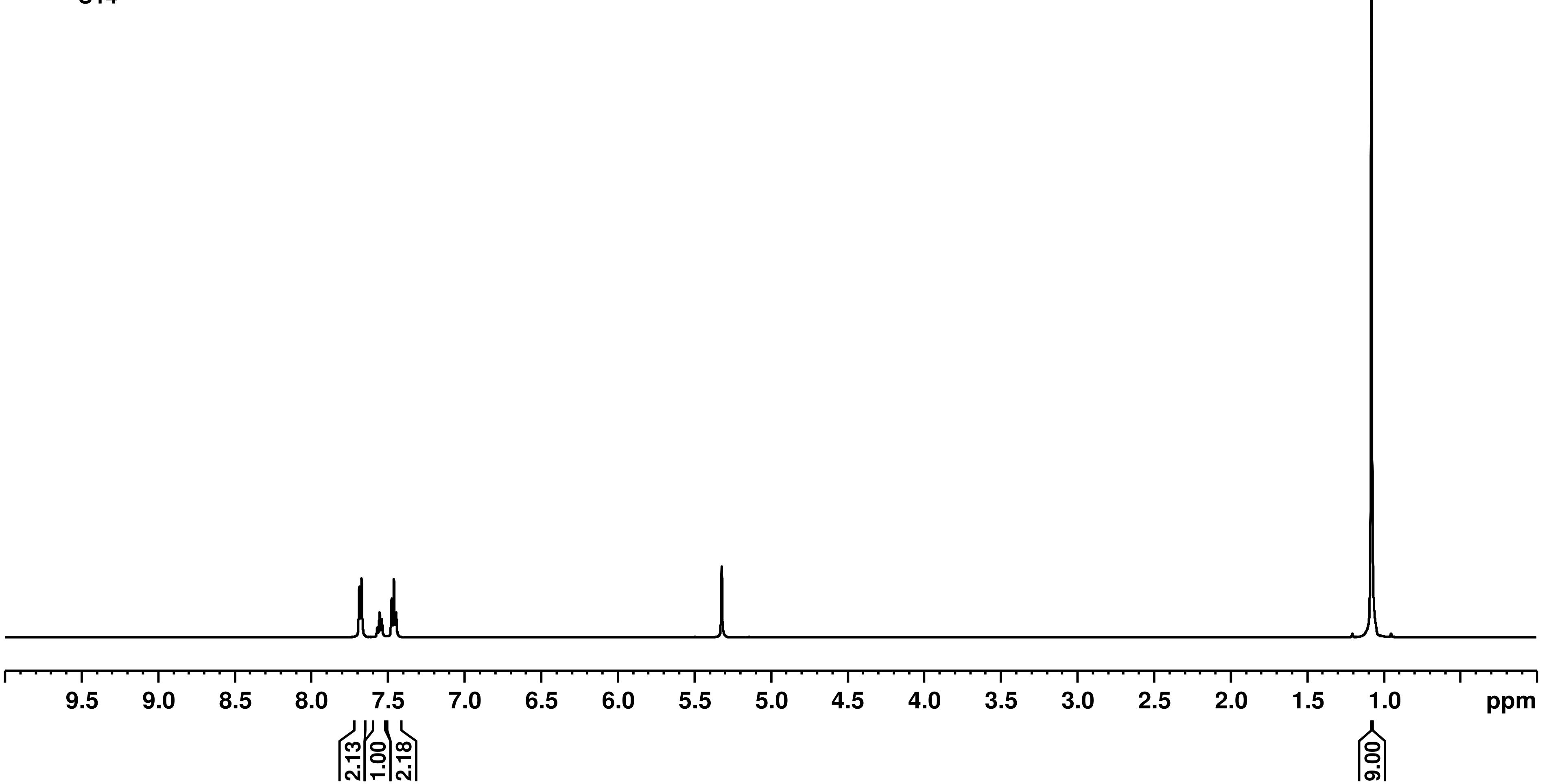

Supplementary Fig. 115. **^{13}C{^{1}H} NMR** spectrum (126 MHz, CD_2Cl_2, 298 K) of *tert*-butyldifluoro(phenyl)silane (**S14**)

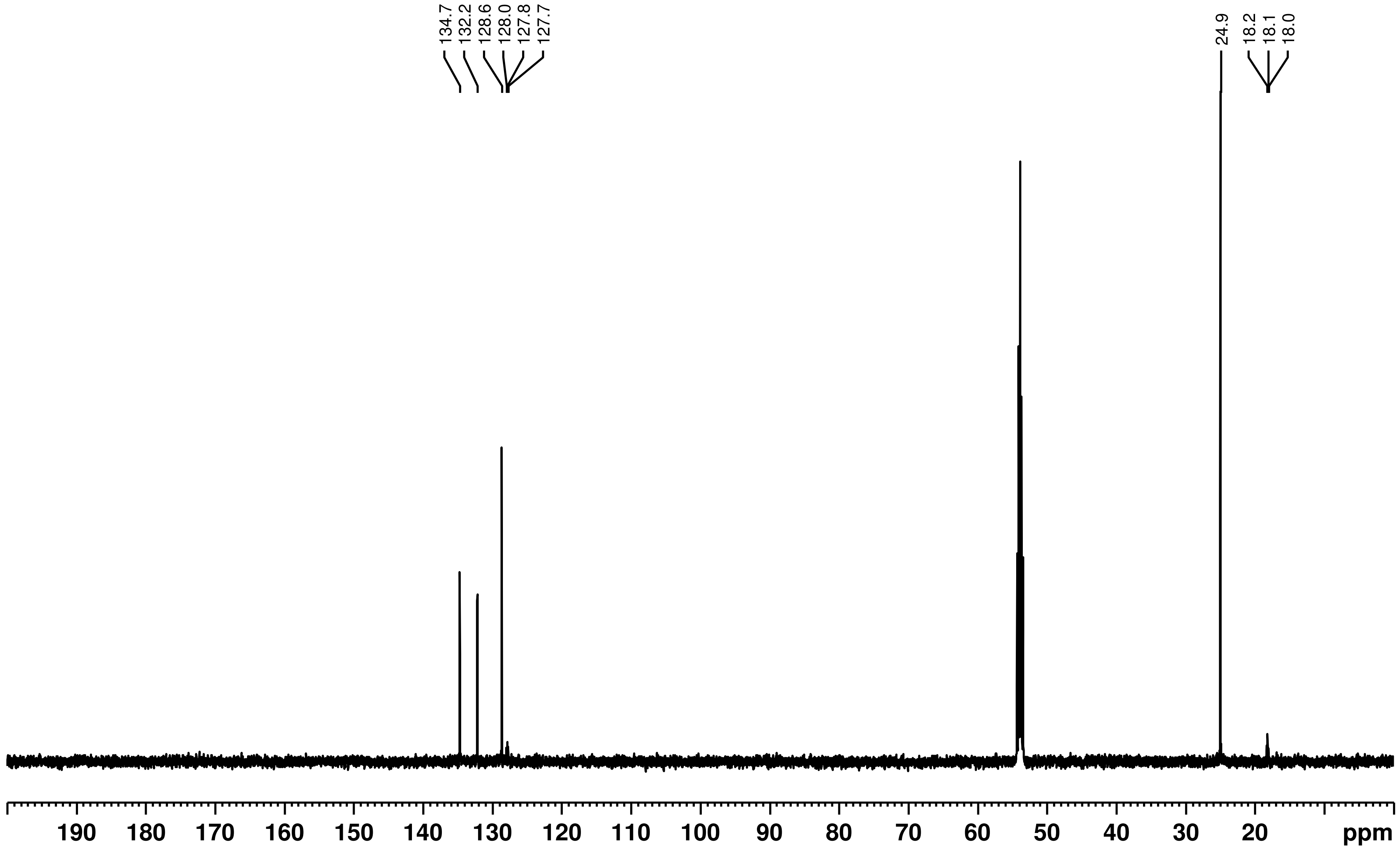

Supplementary Fig. 116. **^{19}F NMR** spectrum (471 MHz, CD_2Cl_2, 298 K) of *tert*-butyldifluoro(phenyl)silane (**S14**)

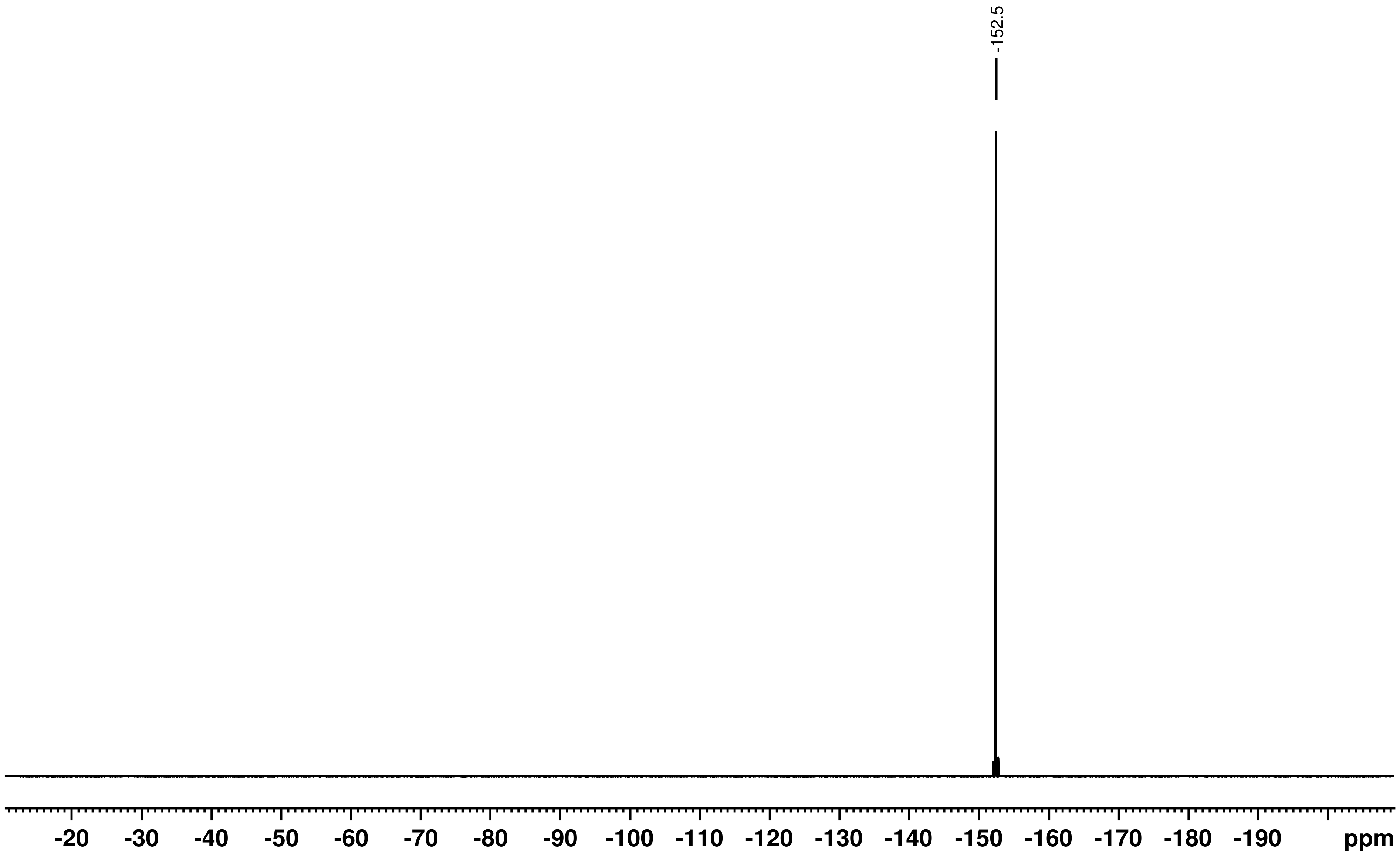

Supplementary Fig. 117. **^{29}Si{^{1}H} DEPT NMR** spectrum (99 MHz, CD_2Cl_2, 298 K, optimized for $J_{H,Si}$ = 7 Hz, 17.5°) of *tert*-butyldifluoro(phenyl)silane (**S14**)

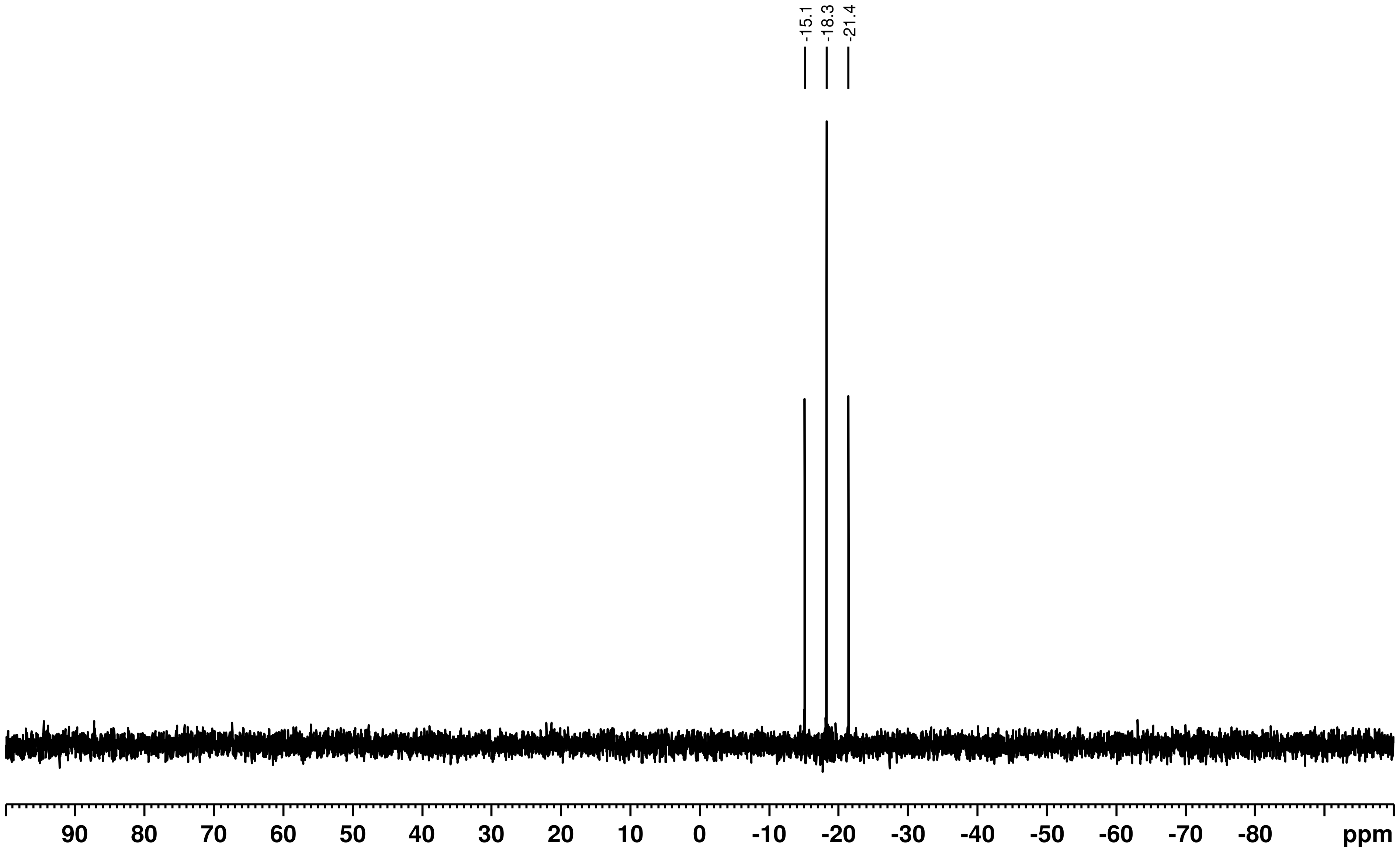

Supplementary Fig. 118. **IR** spectrum (ATR) of *tert*-butyldifluoro(phenyl)silane (**S14**)

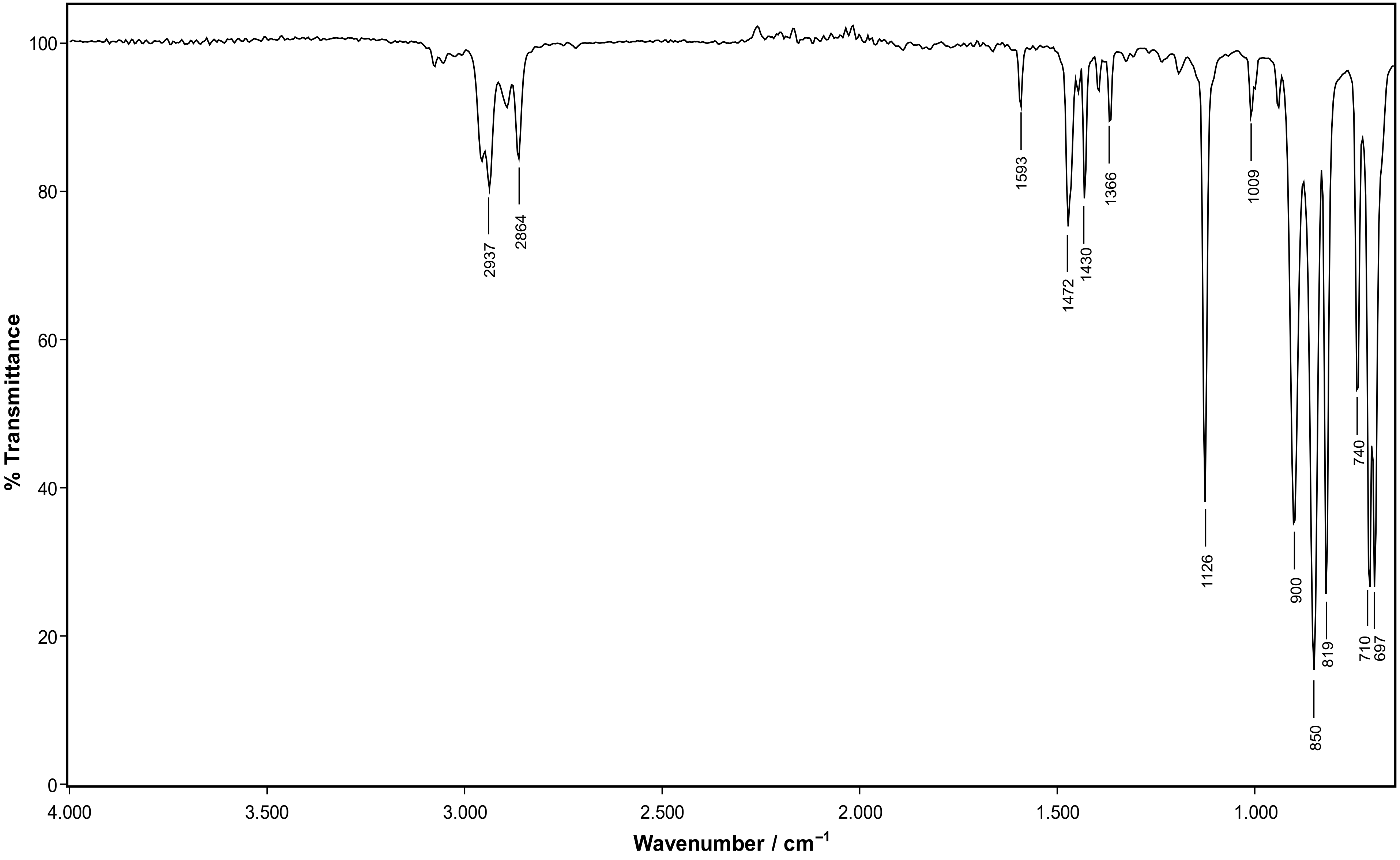

Supplementary Fig. 119. **GLC-MS** spectrum (EI) of *tert*-butyldifluoro(phenyl)silane (**S14**)

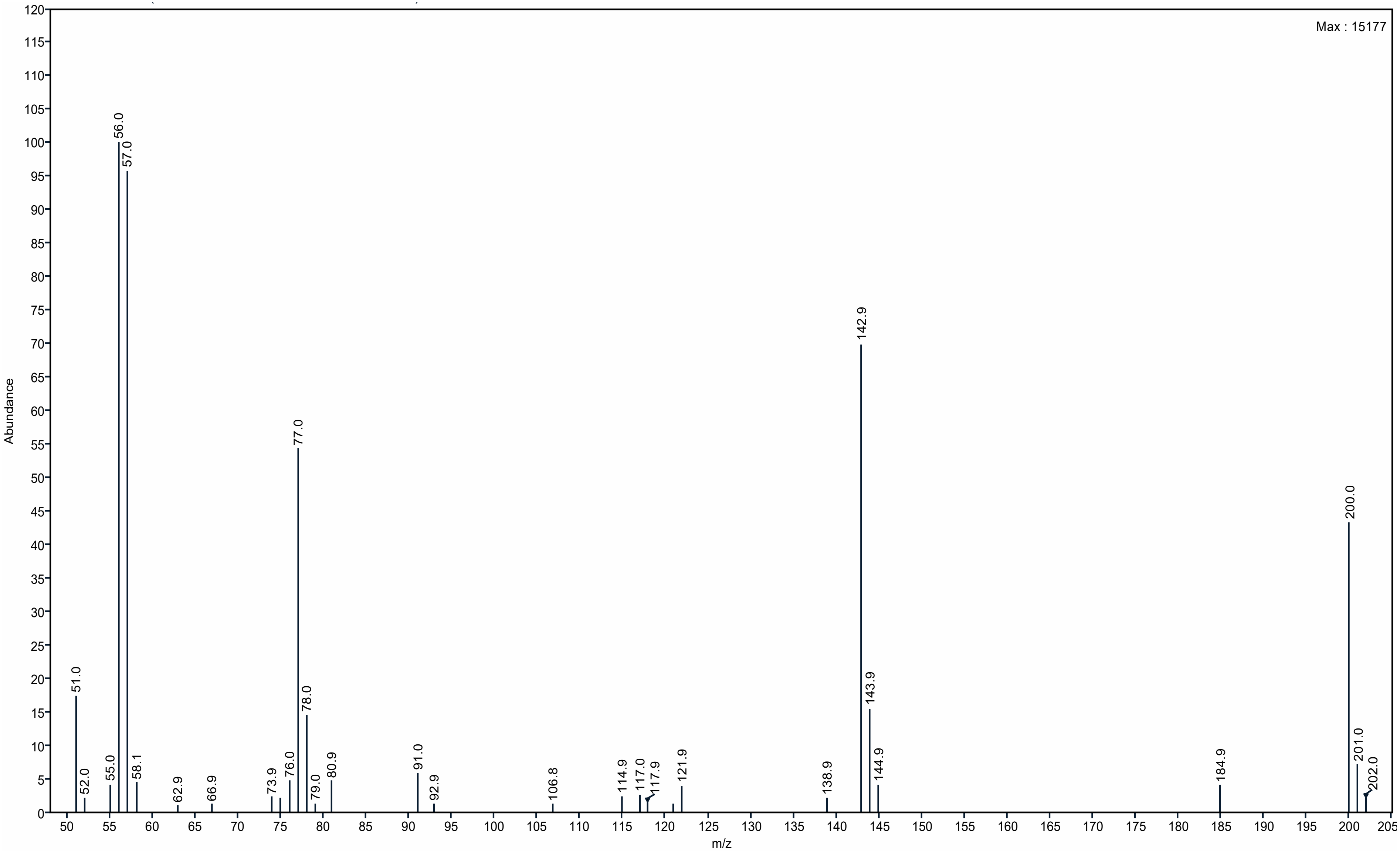

Supplementary Fig. 120. **^{1}H NMR** spectrum (500 MHz, 1,2-$C_6D_4Cl_2$, 298 K) of [*i*$Pr_2HSi(HCB_{11}H_5Br_6)$] (**5c**)

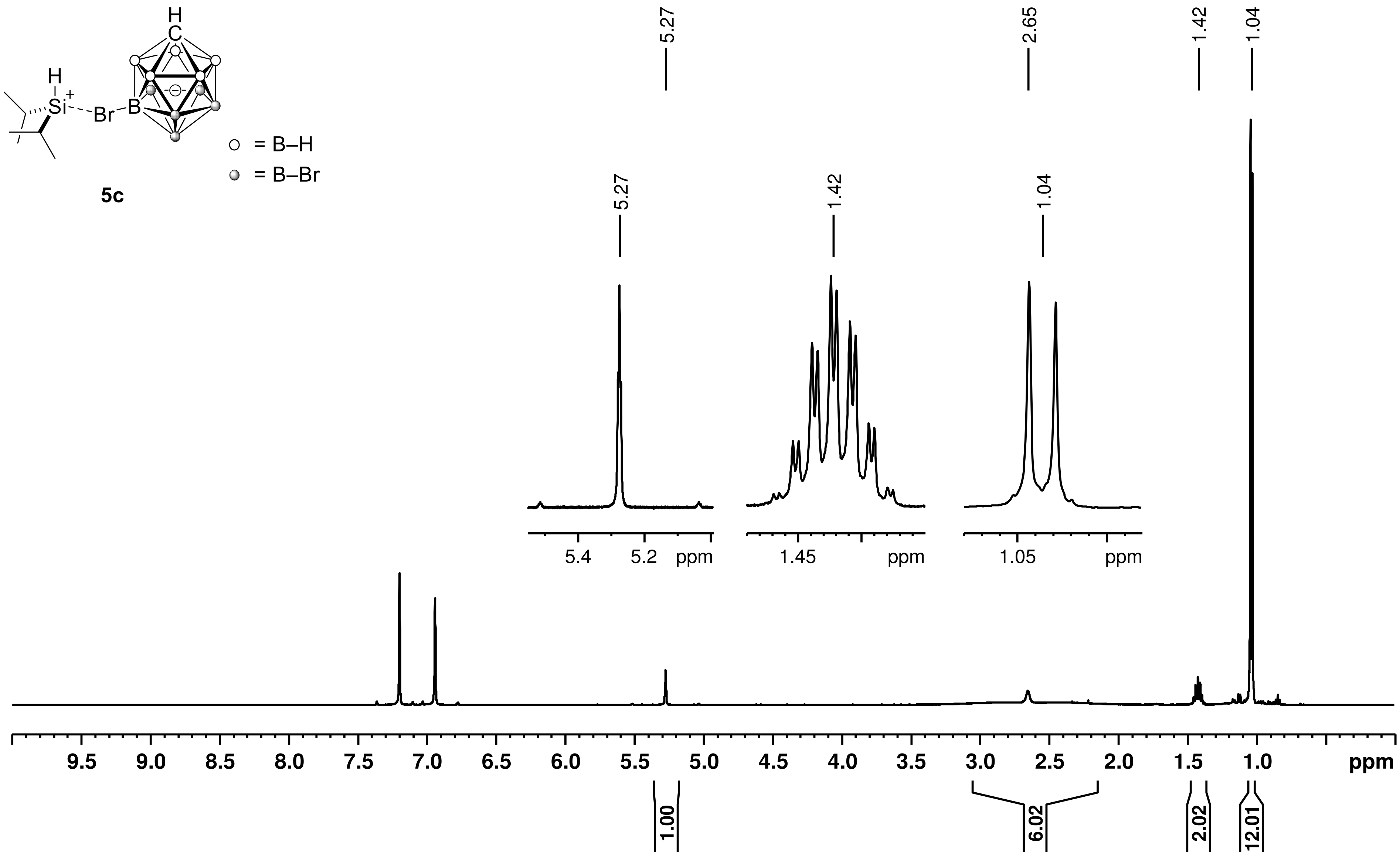

Supplementary Fig. 121. **^{11}B NMR** spectrum (160 MHz, 1,2-$C_6D_4Cl_2$, 298 K) of [*i*$Pr_2HSi(HCB_{11}H_5Br_6)$] (**5c**)

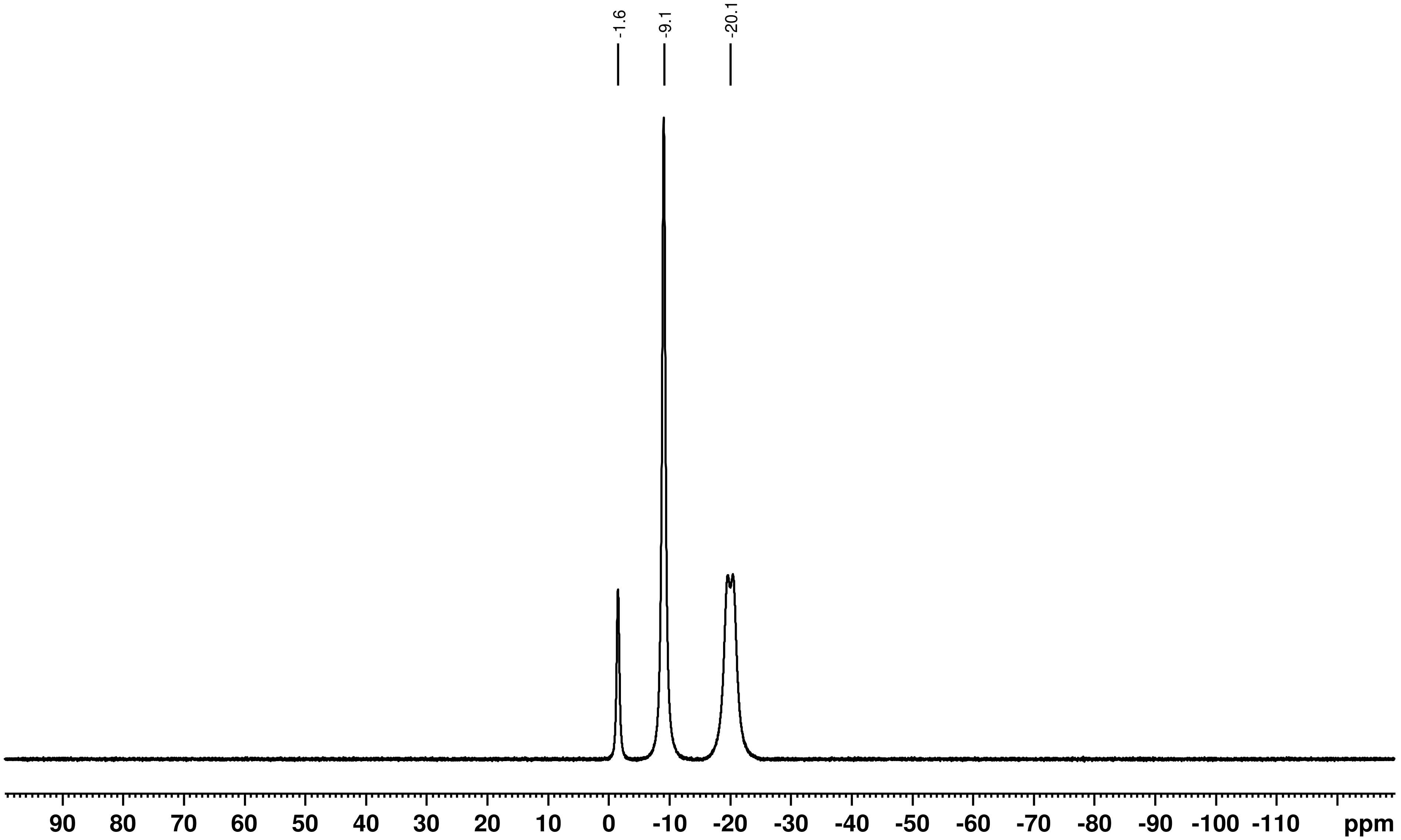

Supplementary Fig. 122. **$^{13}C\{^1H\}$ NMR** spectrum (126 MHz, 1,2-$C_6D_4Cl_2$, 298 K) of [*i*$Pr_2HSi(HCB_{11}H_5Br_6)$] (**5c**)

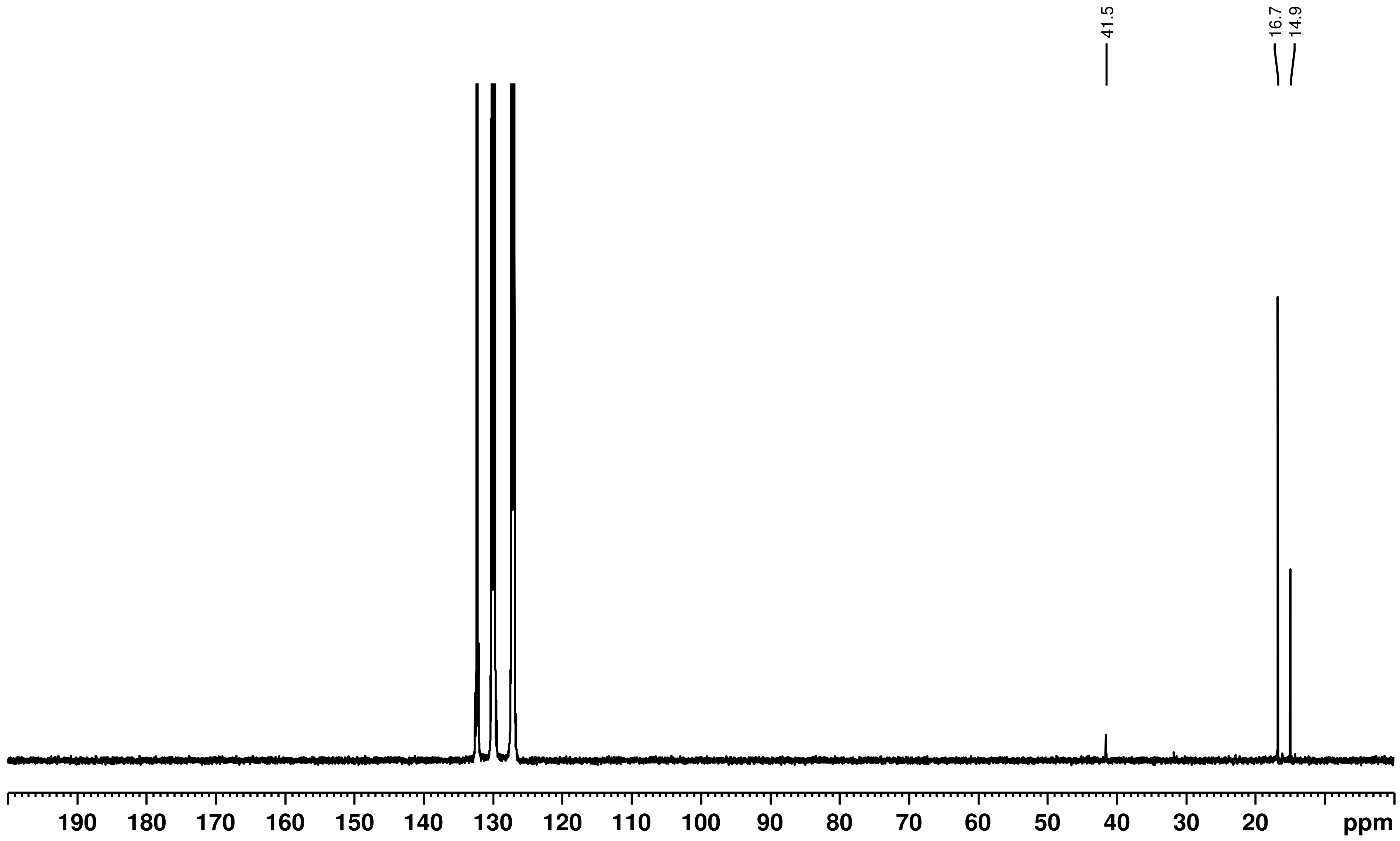

Supplementary Fig. 123. **^{29}Si{^{1}H} DEPT NMR** spectrum (99 MHz, 1,2-$C_6D_4Cl_2$, 298 K, optimized for $J_{H,Si}$ = 240 Hz, 90.0°) of [$i$$Pr_2$HSi($HCB_{11}H_5Br_6$)] (**5c**)

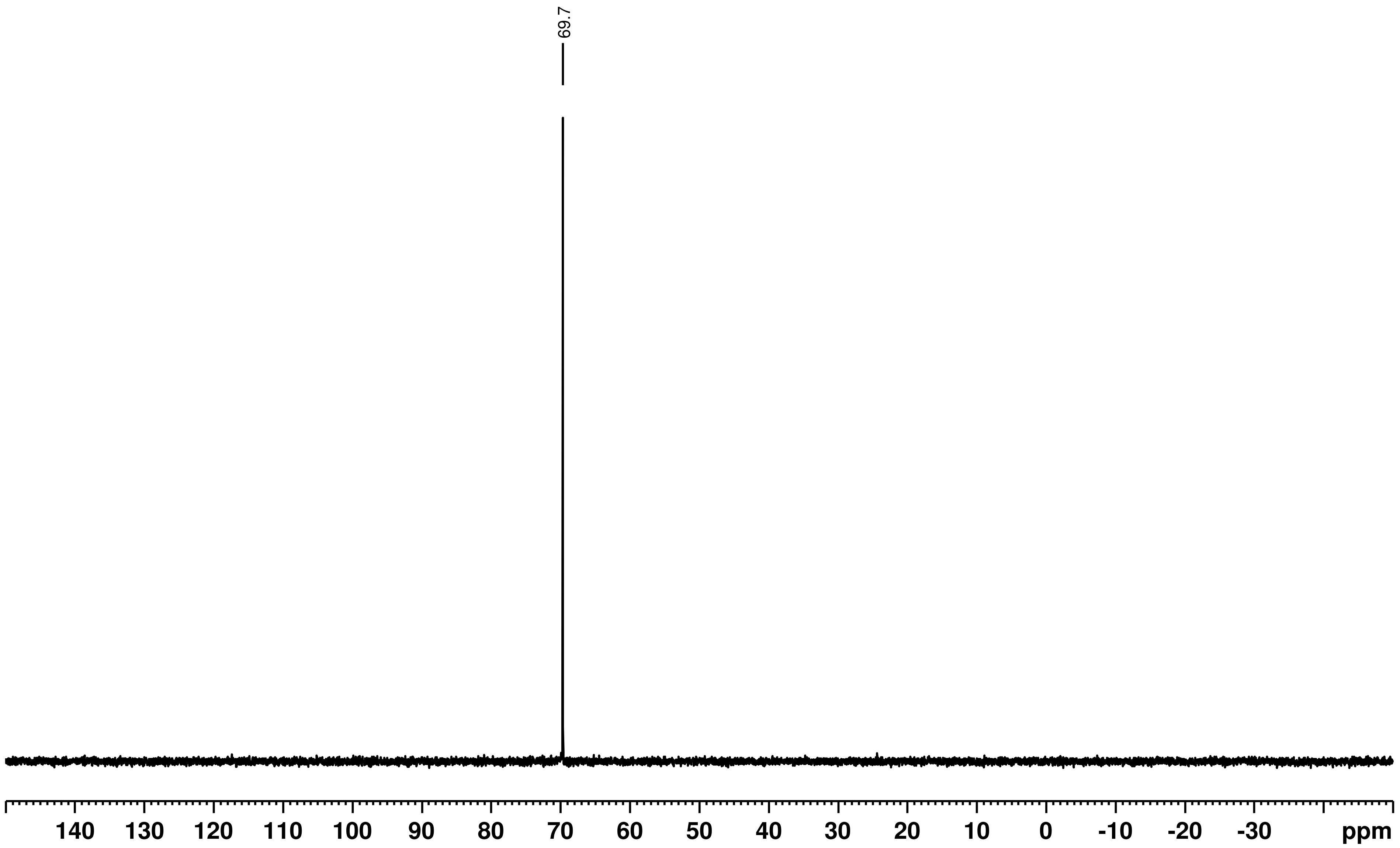

Supplementary Fig. 124. **^{1}H NMR** spectrum (500 MHz, 1,2-$C_6D_4Cl_2$, 298 K) of [$Me_2FSi(HCB_{11}H_5Br_6)$] (**8aa**)

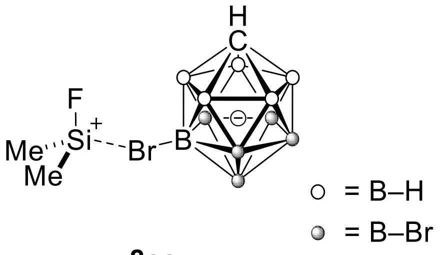

8aa

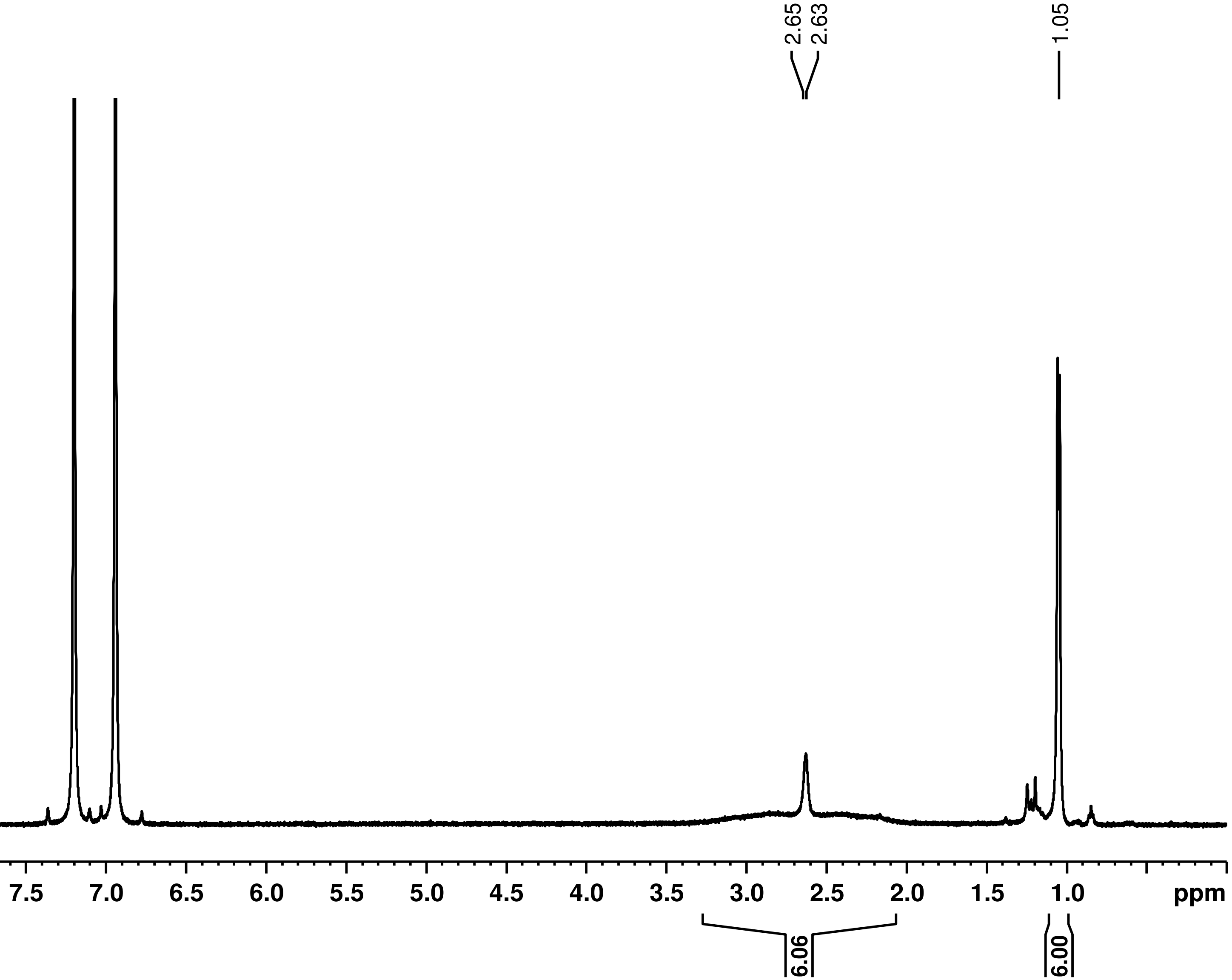

Supplementary Fig. 125. **^{11}B NMR** spectrum (160 MHz, 1,2-$C_6D_4Cl_2$, 298 K) of [$Me_2FSi(HCB_{11}H_5Br_6)$] (**8aa**)

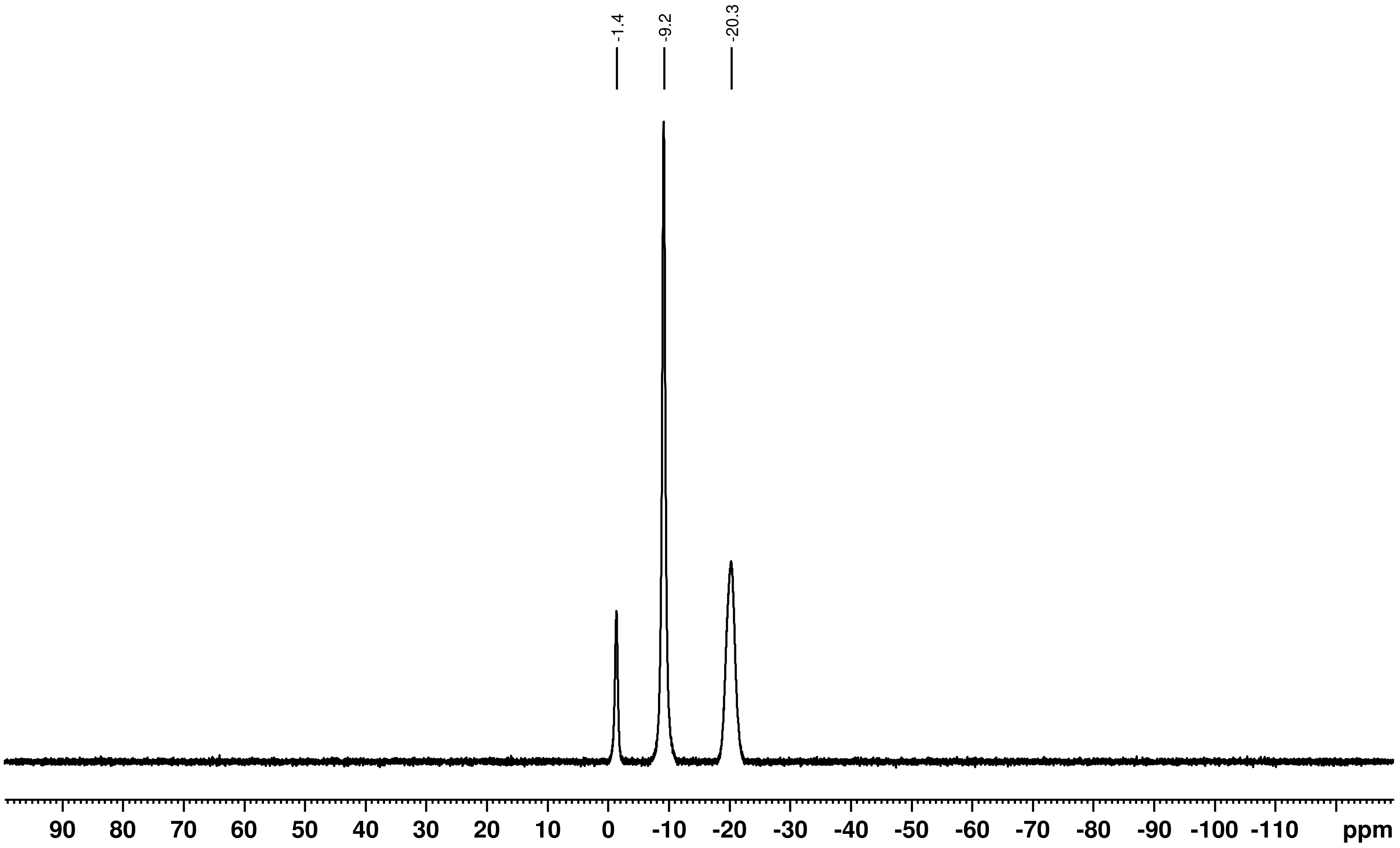

Supplementary Fig. 126. ^{13}**C{^{1}H} NMR** spectrum (126 MHz, 1,2-$C_6D_4Cl_2$, 298 K) of [$Me_2FSi(HCB_{11}H_5Br_6)$] (**8aa**)

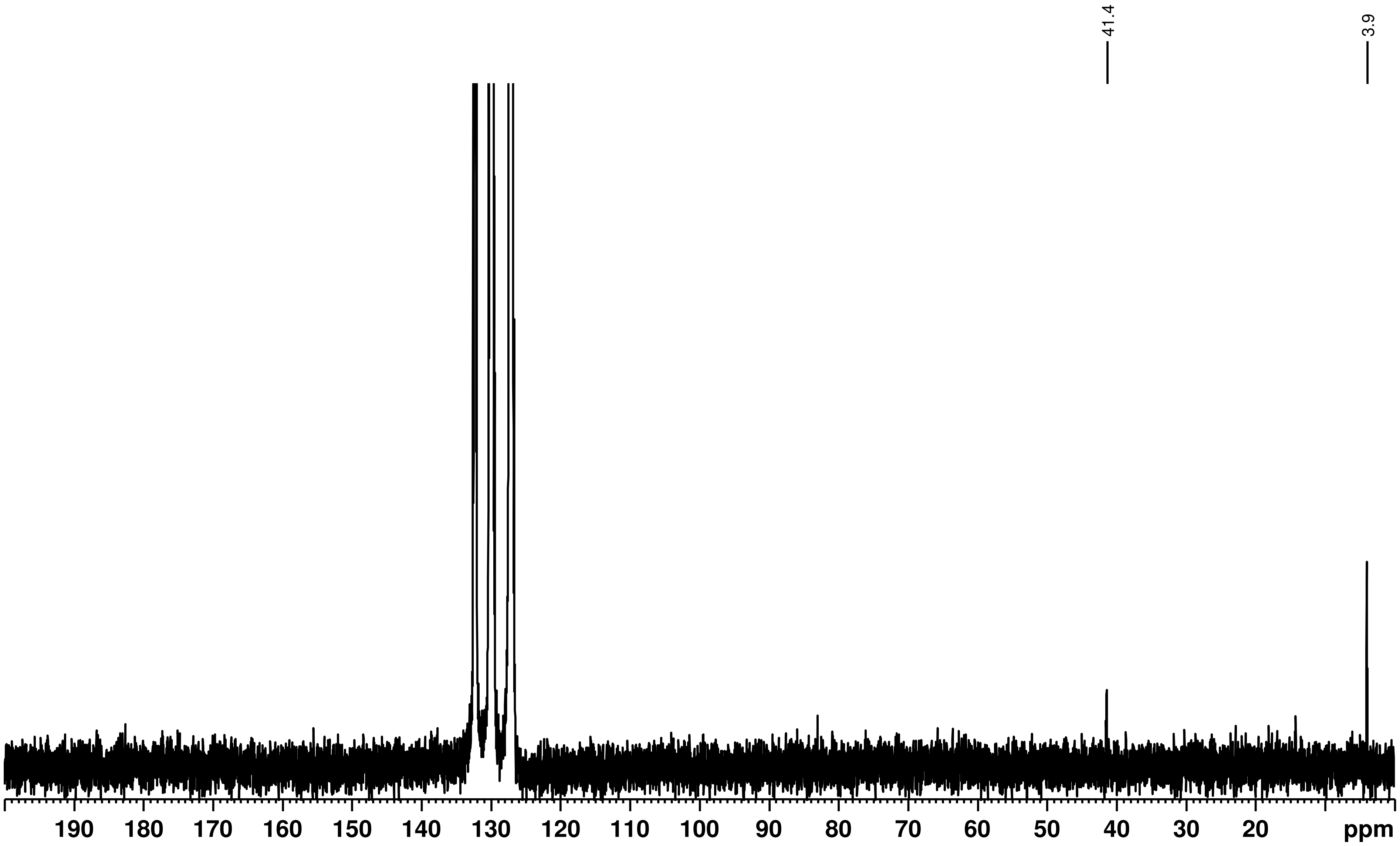

Supplementary Fig. 127. ^{19}**F NMR** spectrum (471 MHz, 1,2-$C_6D_4Cl_2$, 298 K) of [$Me_2FSi(HCB_{11}H_5Br_6)$] (**8aa**)

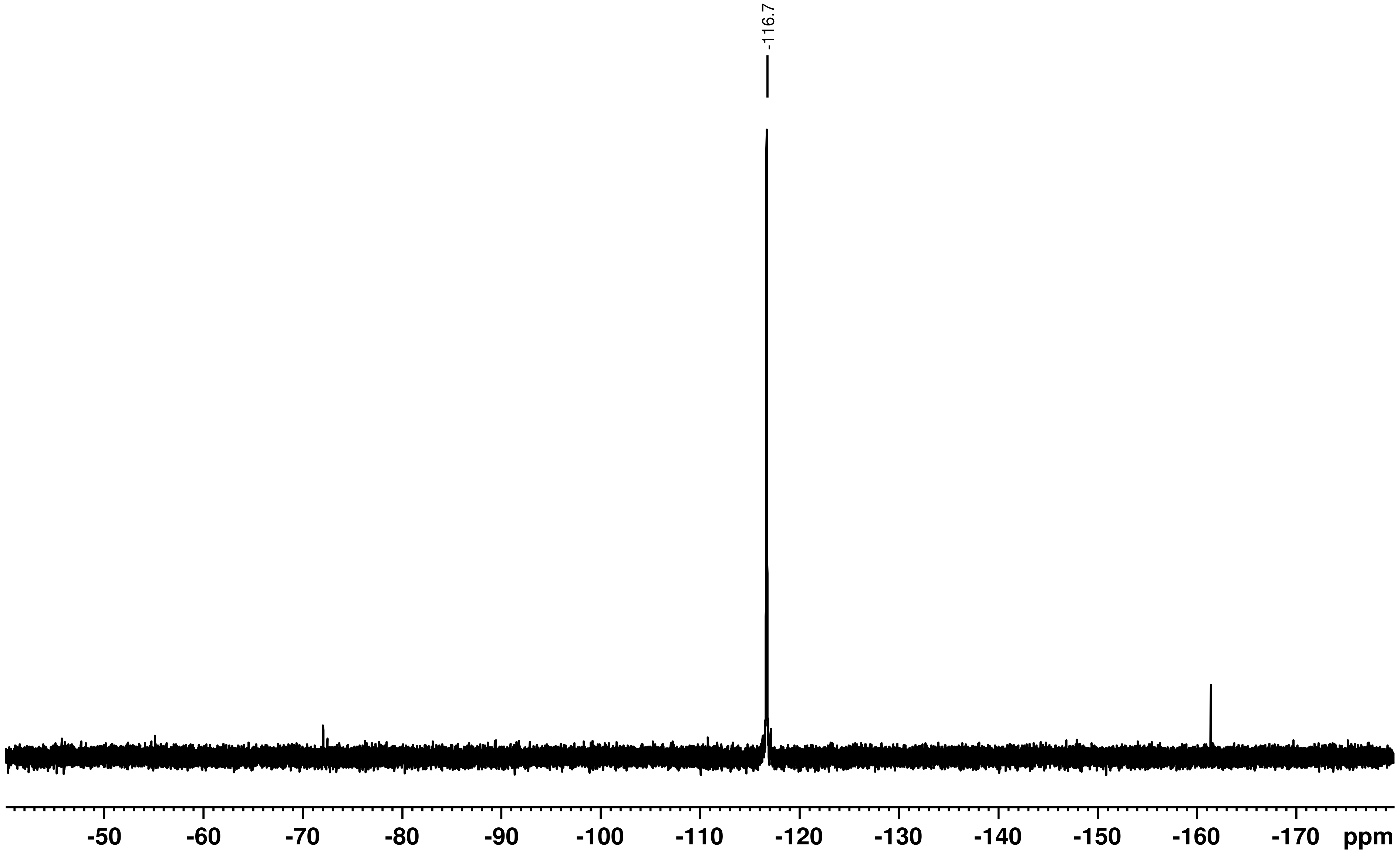

Supplementary Fig. 128. **^{1}H,^{29}Si HMQC NMR** (500/99 MHz, 1,2-$C_6D_4Cl_2$, 298 K, optimized for J = 7 Hz) of [$Me_2FSi(HCB_{11}H_5Br_6)$] (**8aa**)

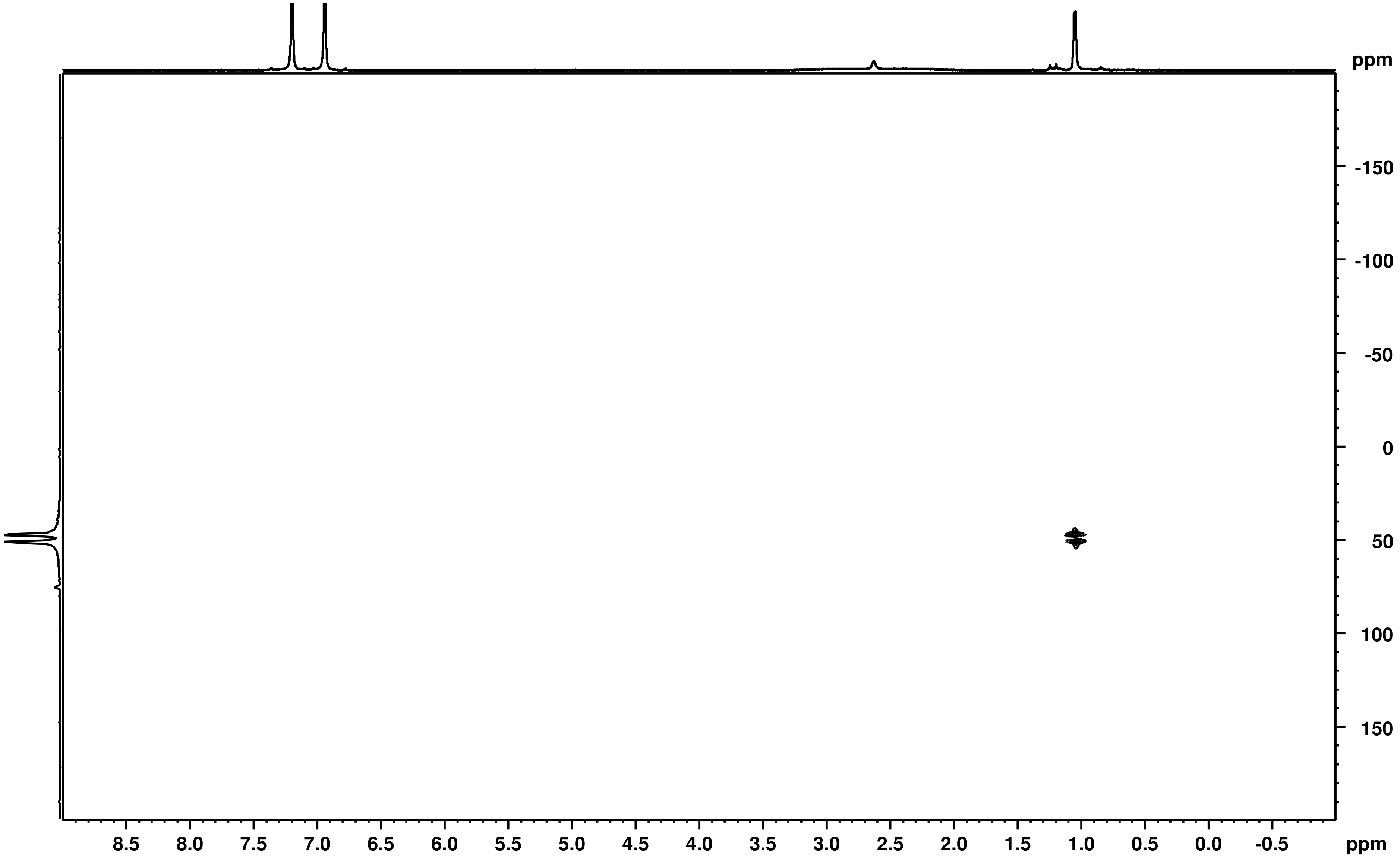

Supplementary Fig. 129. **^{1}H NMR** spectrum (500 MHz, 1,2-$C_6D_4Cl_2$, 298 K) of [$Et_2FSi(HCB_{11}H_5Br_6)$] (**8ab**)

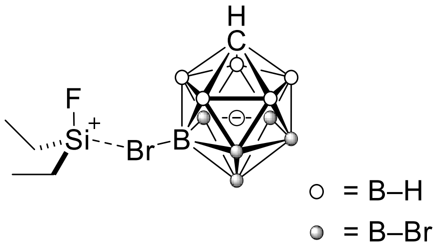

8ab

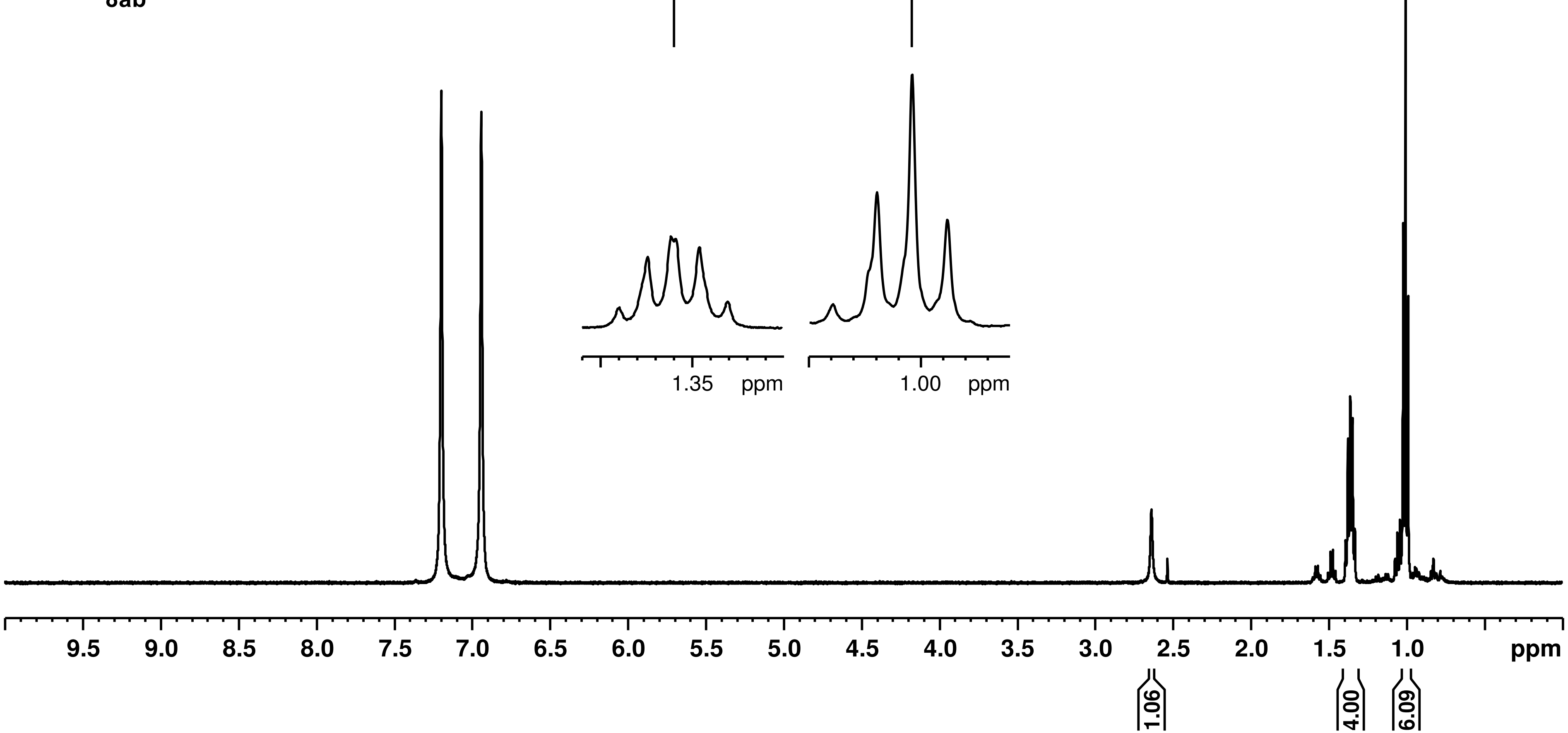

Supplementary Fig. 130. ^{11}**B NMR** spectrum (160 MHz, 1,2-$C_6D_4Cl_2$, 298 K) of [$Et_2FSi(HCB_{11}H_5Br_6)$] (**8ab**)

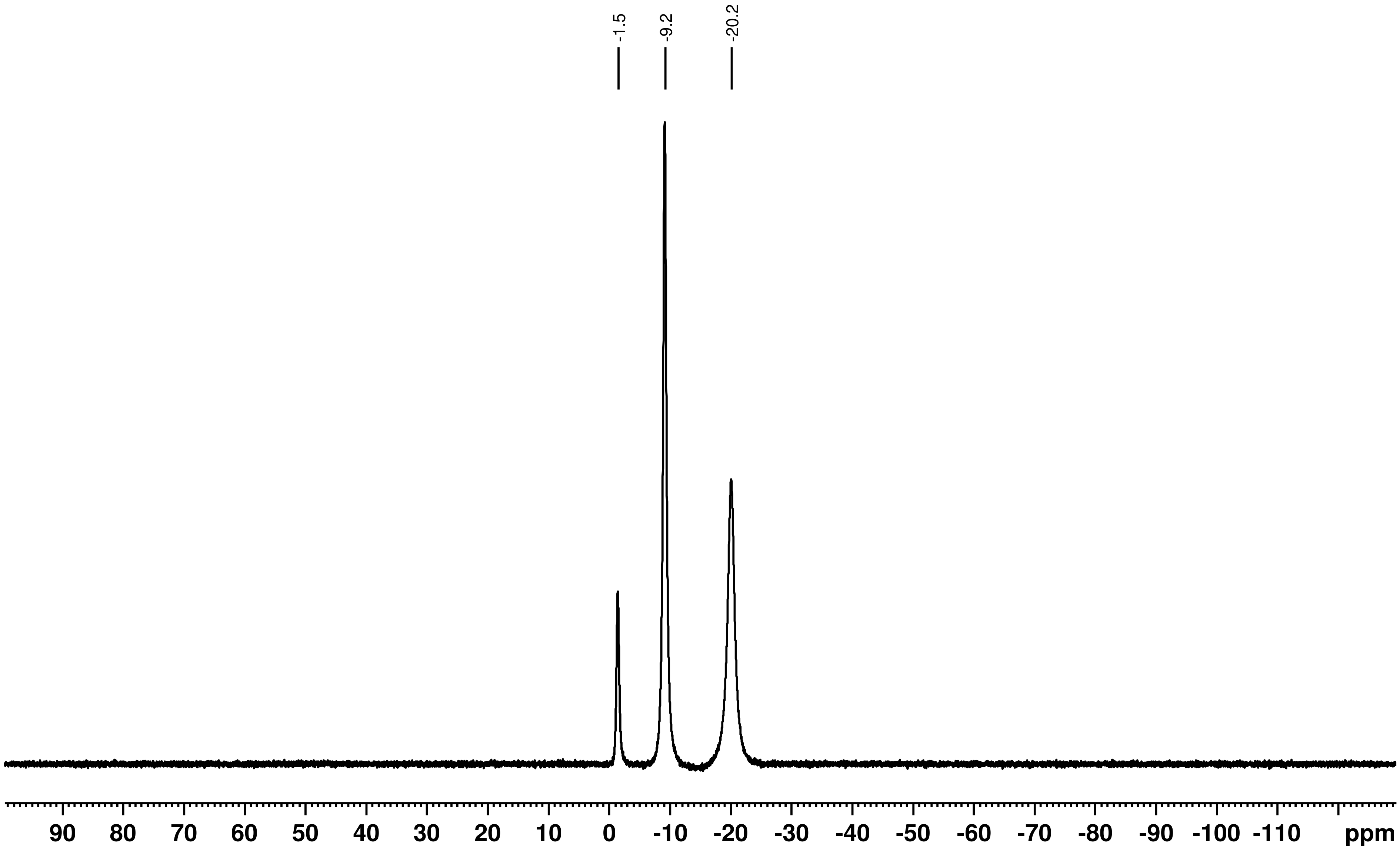

Supplementary Fig. 131. **^{13}C{^{1}H} NMR** spectrum (126 MHz, 1,2-$C_6D_4Cl_2$, 298 K) of [$Et_2FSi(HCB_{11}H_5Br_6)$] (**8ab**)

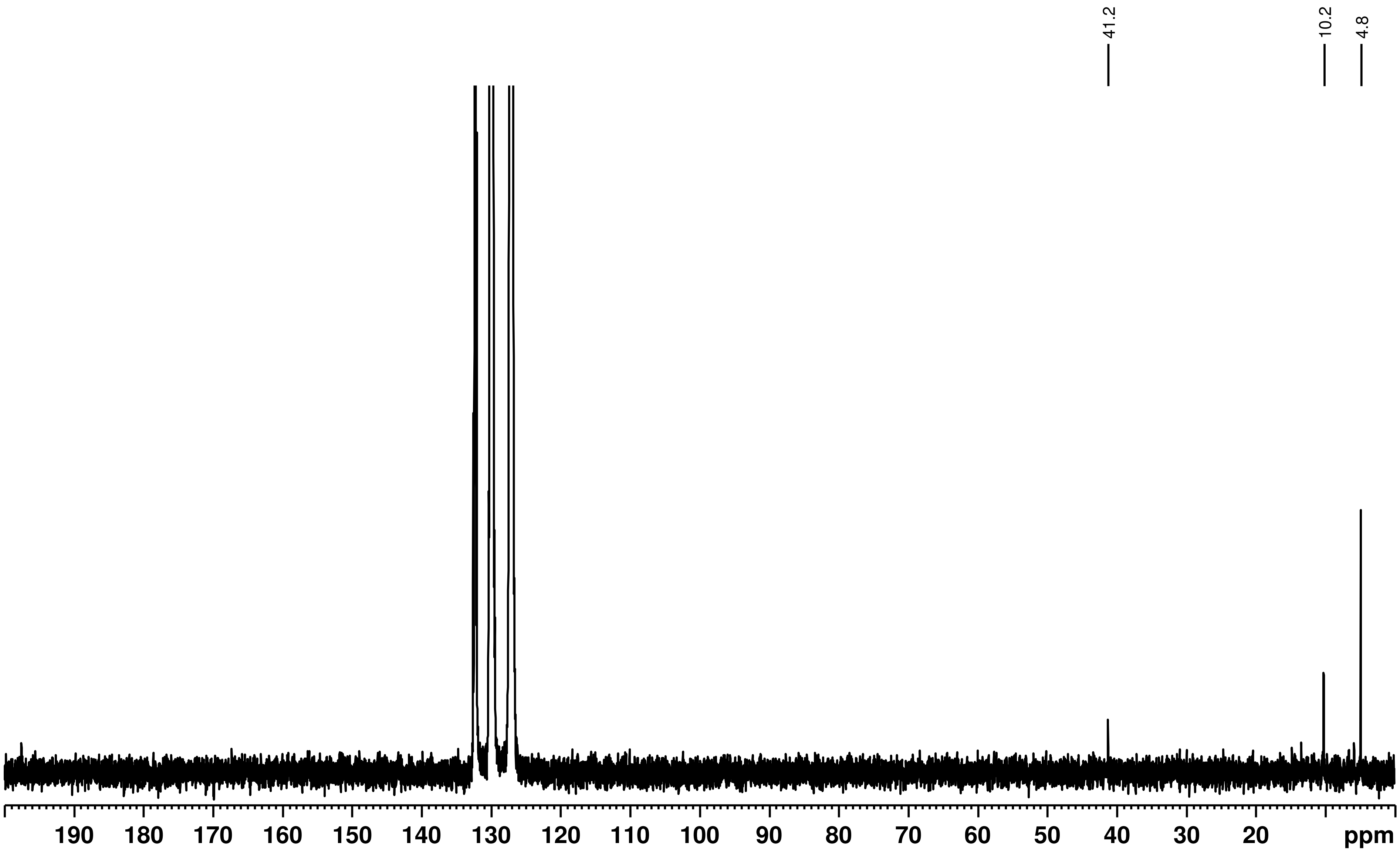

Supplementary Fig. 132. [19]**F NMR** spectrum (471 MHz, 1,2-$C_6D_4Cl_2$, 298 K) of [$Et_2FSi(HCB_{11}H_5Br_6)$] (**8ab**)

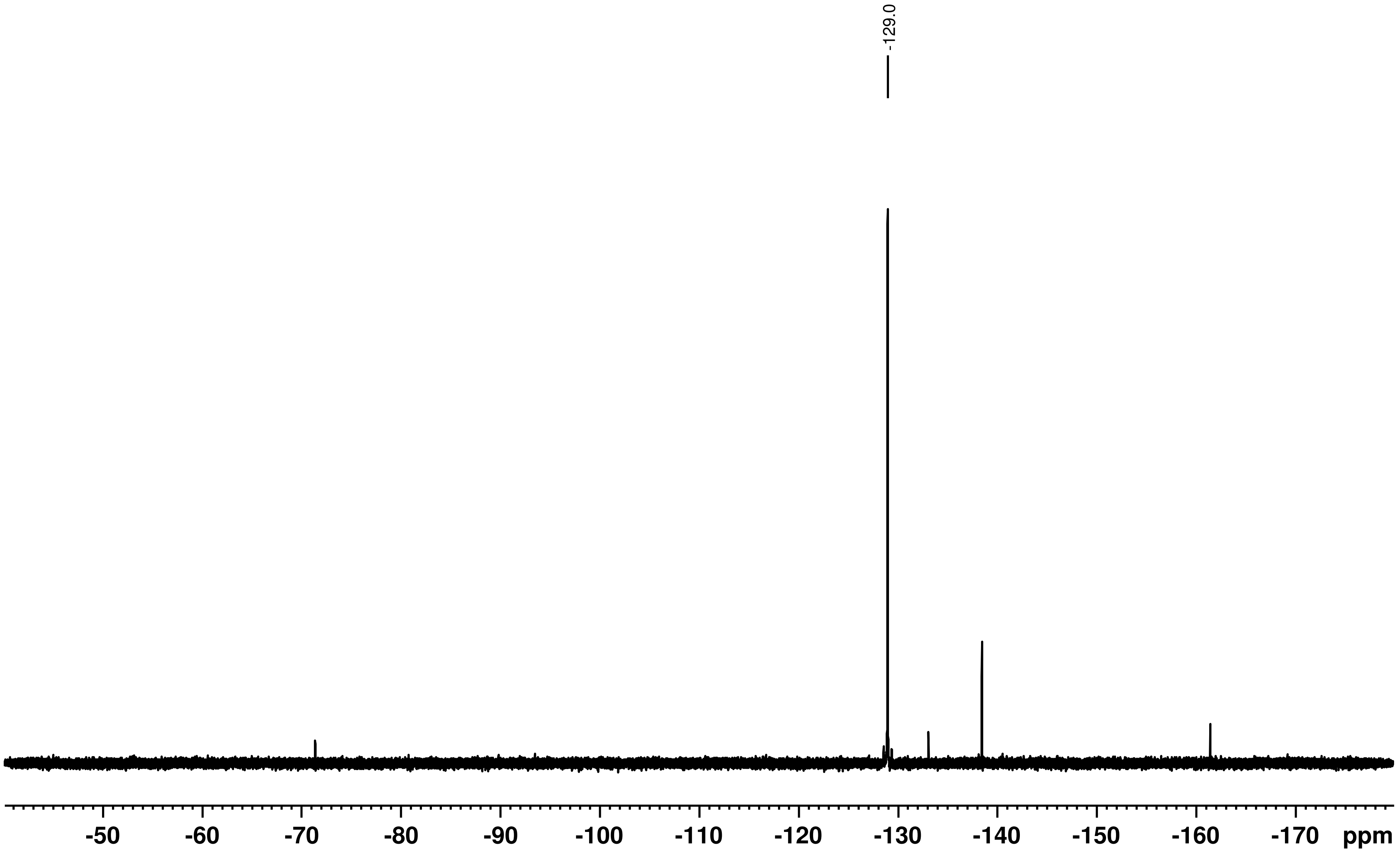

Supplementary Fig. 133. **^{1}H,^{29}Si HMQC NMR** (500/99 MHz, 1,2-$C_6D_4Cl_2$, 298 K, optimized for *J* = 7 Hz) of [$Et_2FSi(HCB_{11}H_5Br_6)$] (**8ab**)

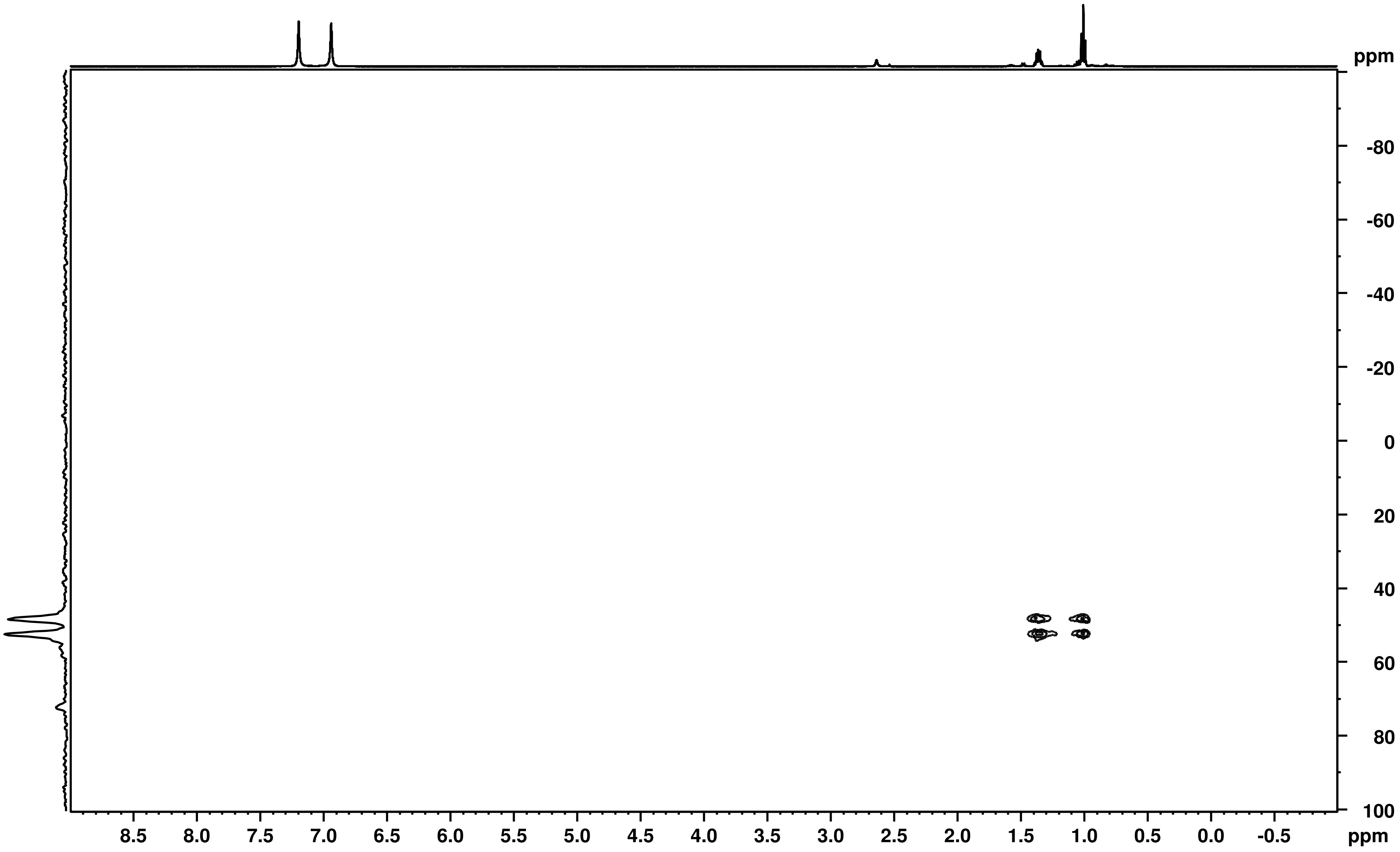

Supplementary Fig. 134. **^{1}H NMR** spectrum (500 MHz, 1,2-$C_6D_4Cl_2$, 298 K) of [*i*$Pr_2FSi(HCB_{11}H_5Br_6)$] (**8ac**)

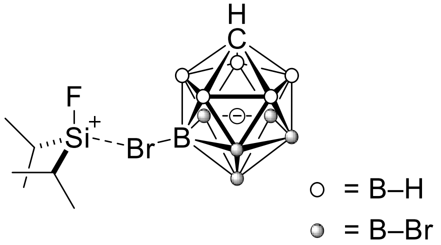

8ac

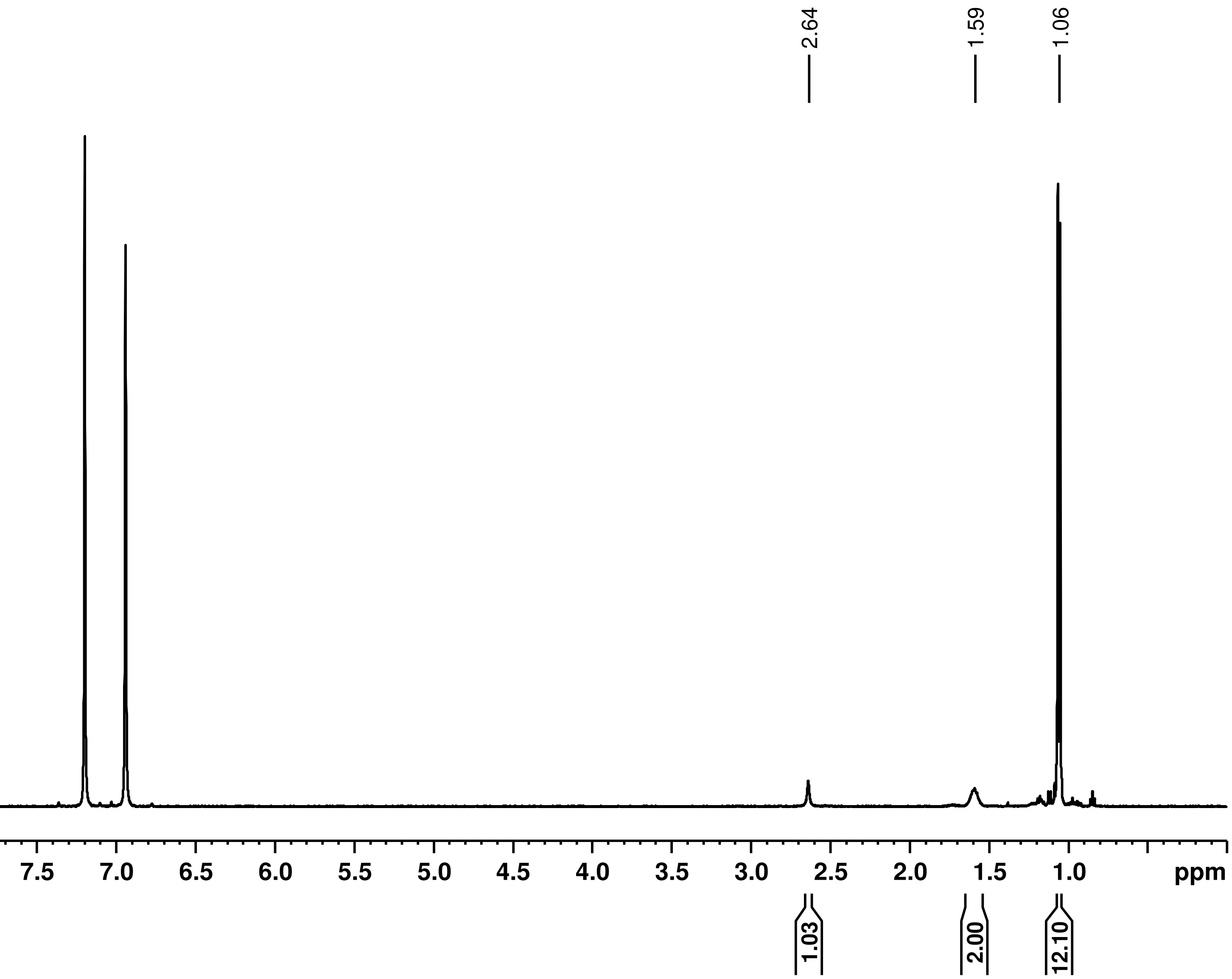

Supplementary Fig. 135. **^{11}B NMR** spectrum (160 MHz, 1,2-$C_6D_4Cl_2$, 298 K) of [*i*$Pr_2FSi(HCB_{11}H_5Br_6)$] (**8ac**)

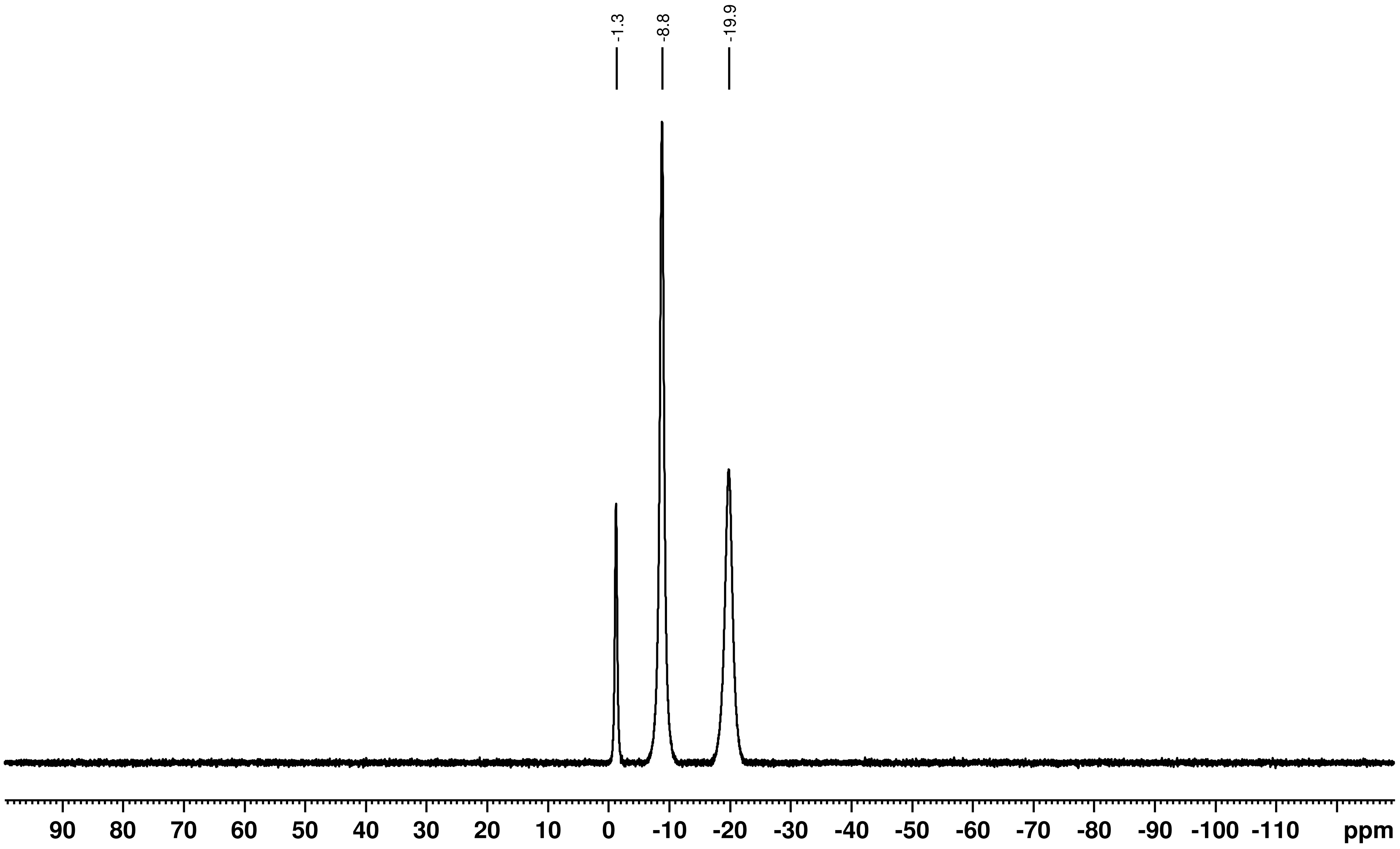

Supplementary Fig. 136. **$^{13}C\{^1H\}$ NMR** spectrum (126 MHz, 1,2-$C_6D_4Cl_2$, 298 K) of [*i*$Pr_2FSi(HCB_{11}H_5Br_6)$] (**8ac**)

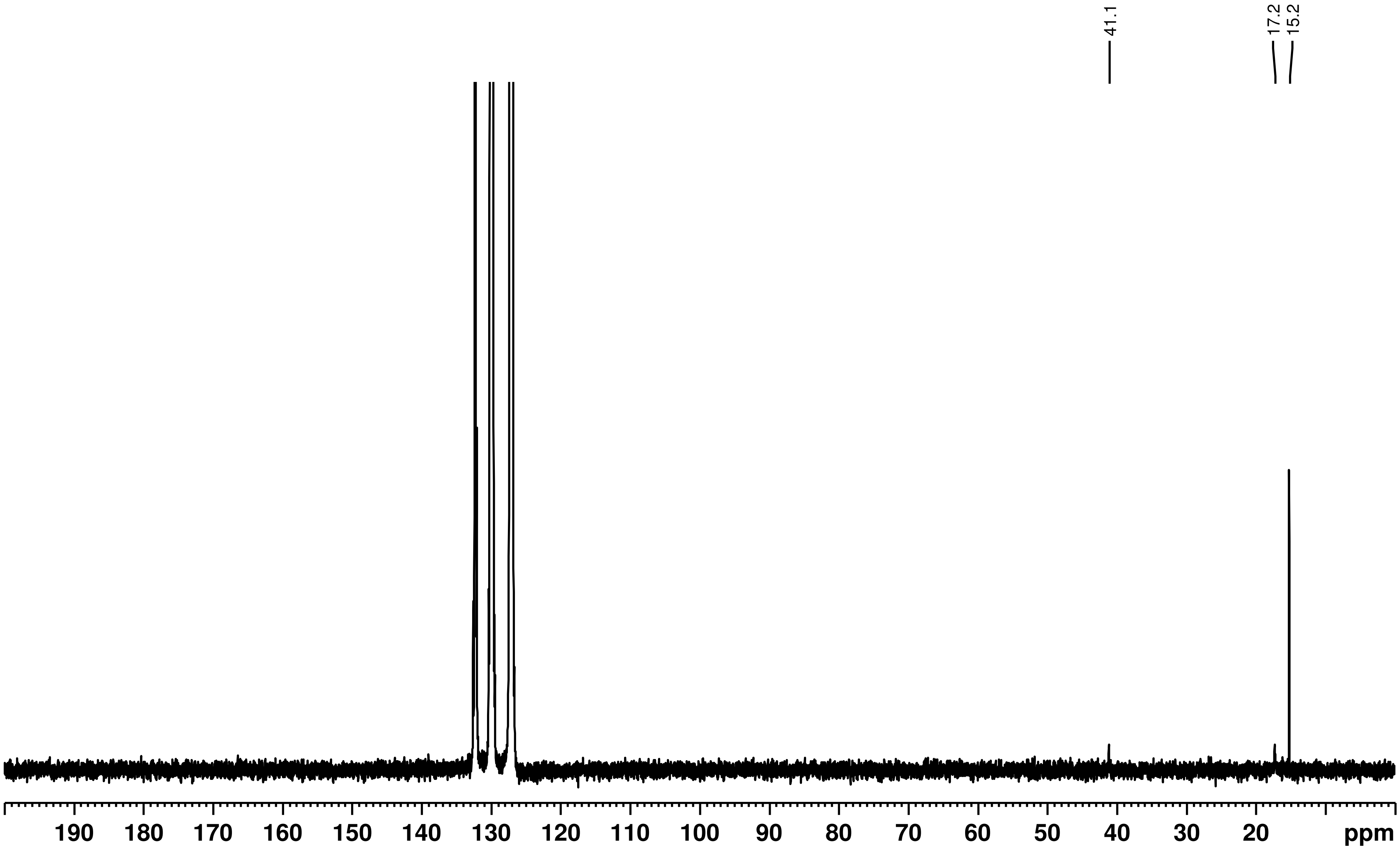

Supplementary Fig. 137. ^{19}**F NMR** spectrum (471 MHz, 1,2-$C_6D_4Cl_2$, 298 K) of [*i*$Pr_2FSi(HCB_{11}H_5Br_6)$] (**8ac**)

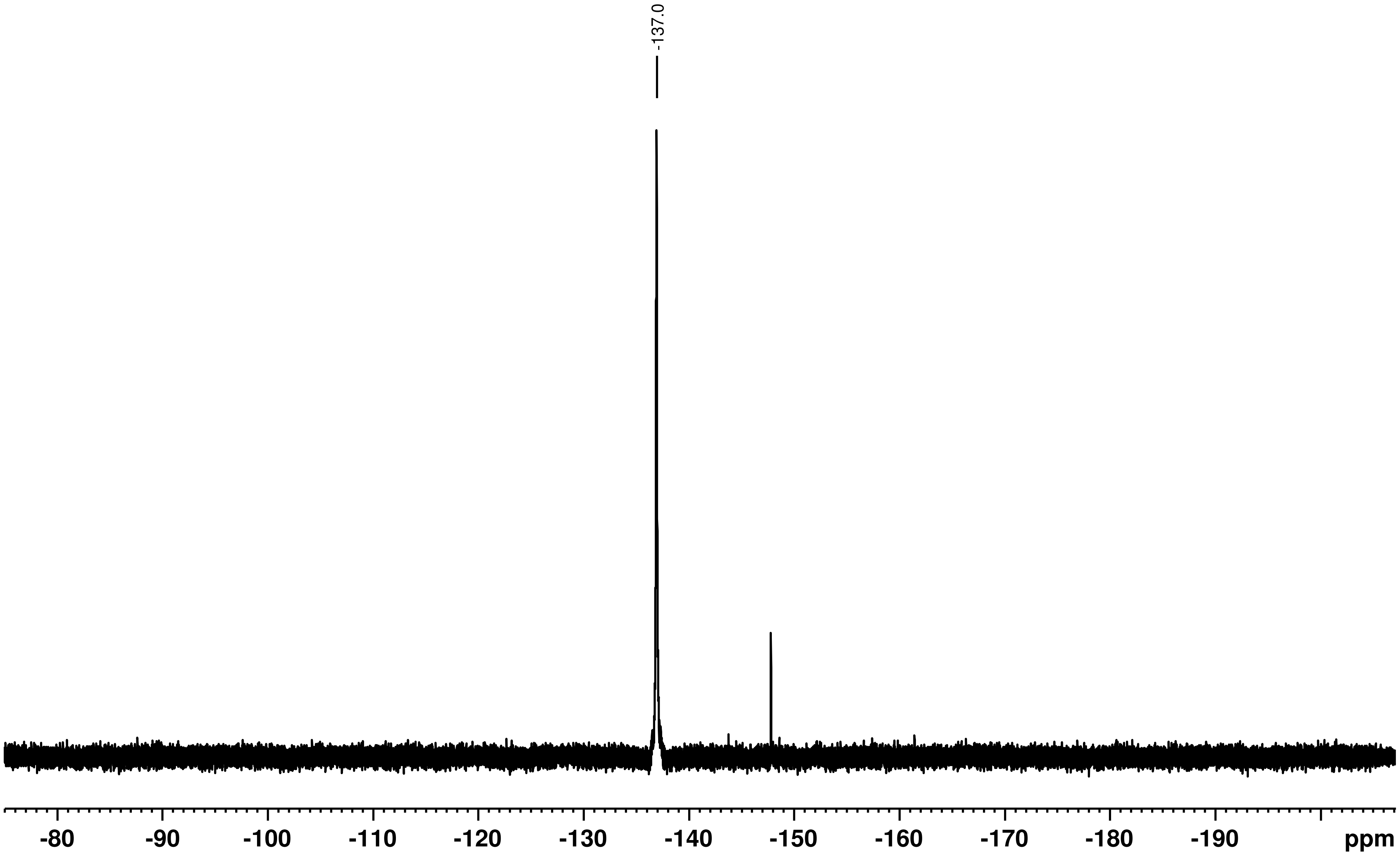

Supplementary Fig. 138. **$^{1}H,^{29}Si$ HMQC NMR** (500/99 MHz, 1,2-$C_6D_4Cl_2$, 298 K, optimized for *J* = 7 Hz) of [*i*$Pr_2FSi(HCB_{11}H_5Br_6)$] (**8ac**)

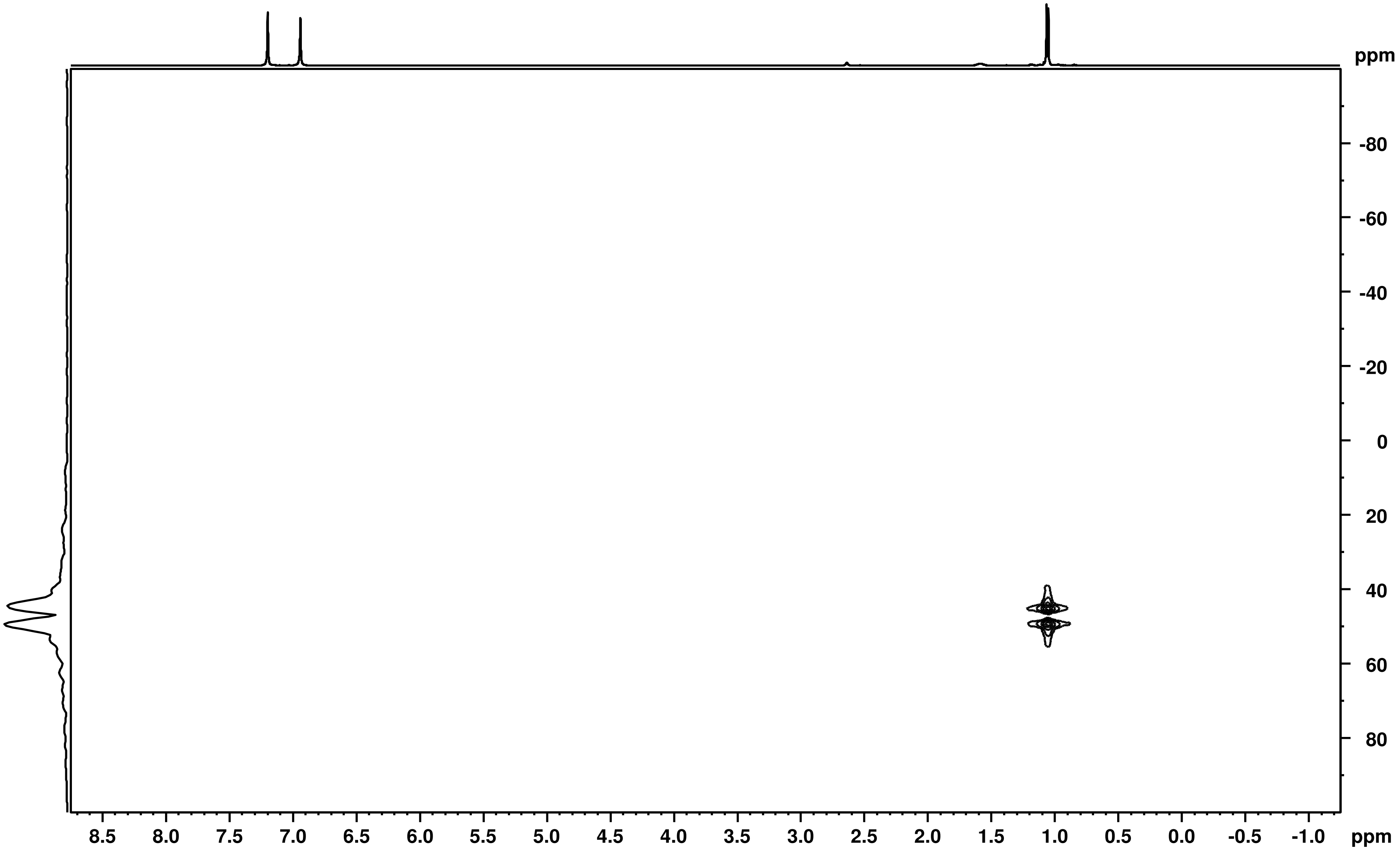

Supplementary Fig. 139. [1]**H NMR** spectrum (500 MHz, 1,2-$C_6D_4Cl_2$, 298 K) of [*t*$Bu_2FSi(HCB_{11}H_5Br_6)$] (**8ad**)

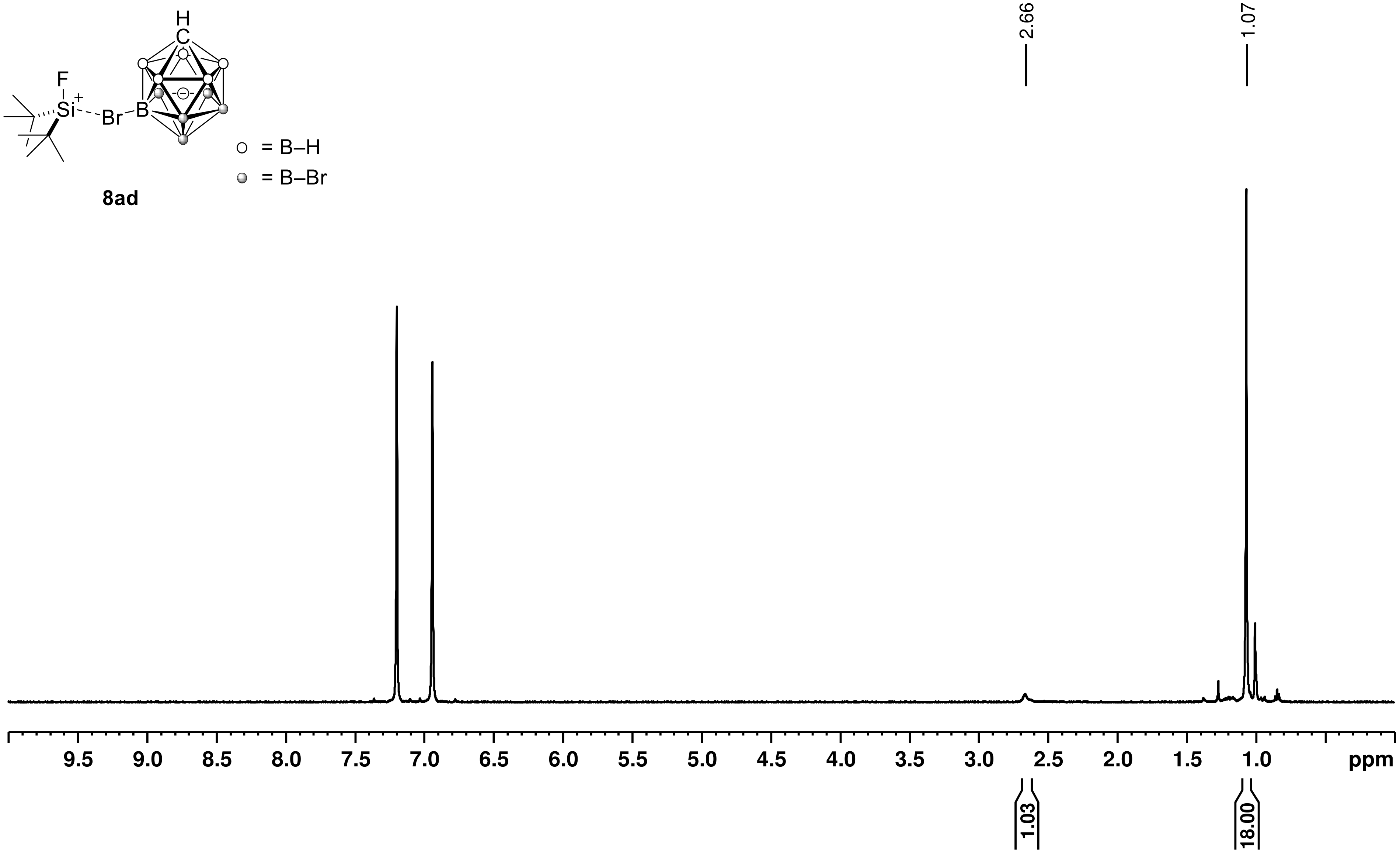

Supplementary Fig. 140. **^{11}B NMR** spectrum (160 MHz, 1,2-$C_6D_4Cl_2$, 298 K) of [tBu$_2$FSi(HCB$_{11}$H$_5$Br$_6$)] (**8ad**) (* anion decomposition)

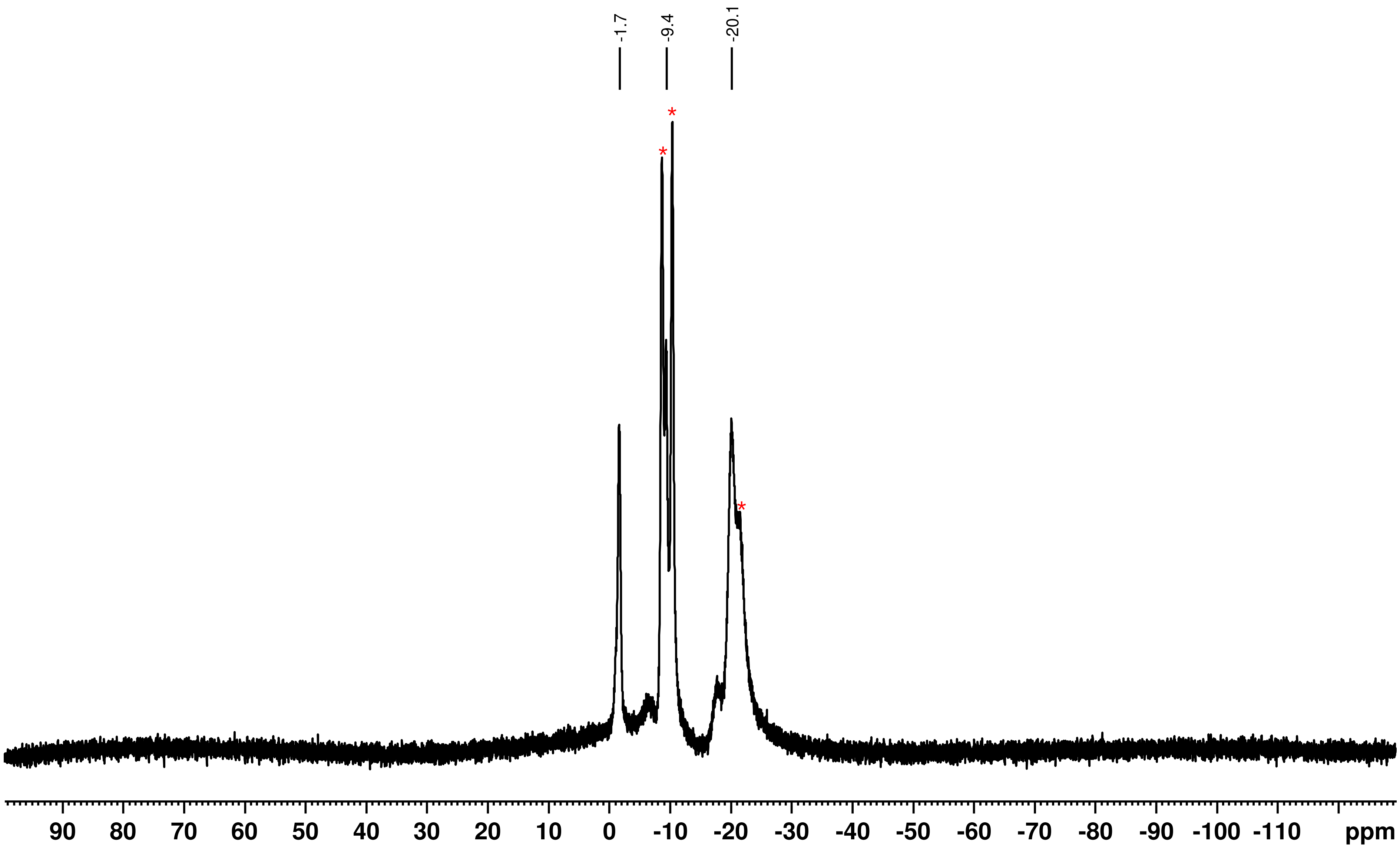

Supplementary Fig. 141. **$^{13}C\{^1H\}$ NMR** spectrum (126 MHz, 1,2-$C_6D_4Cl_2$, 298 K) of [*t*$Bu_2FSi(HCB_{11}H_5Br_6)$] (**8ad**)

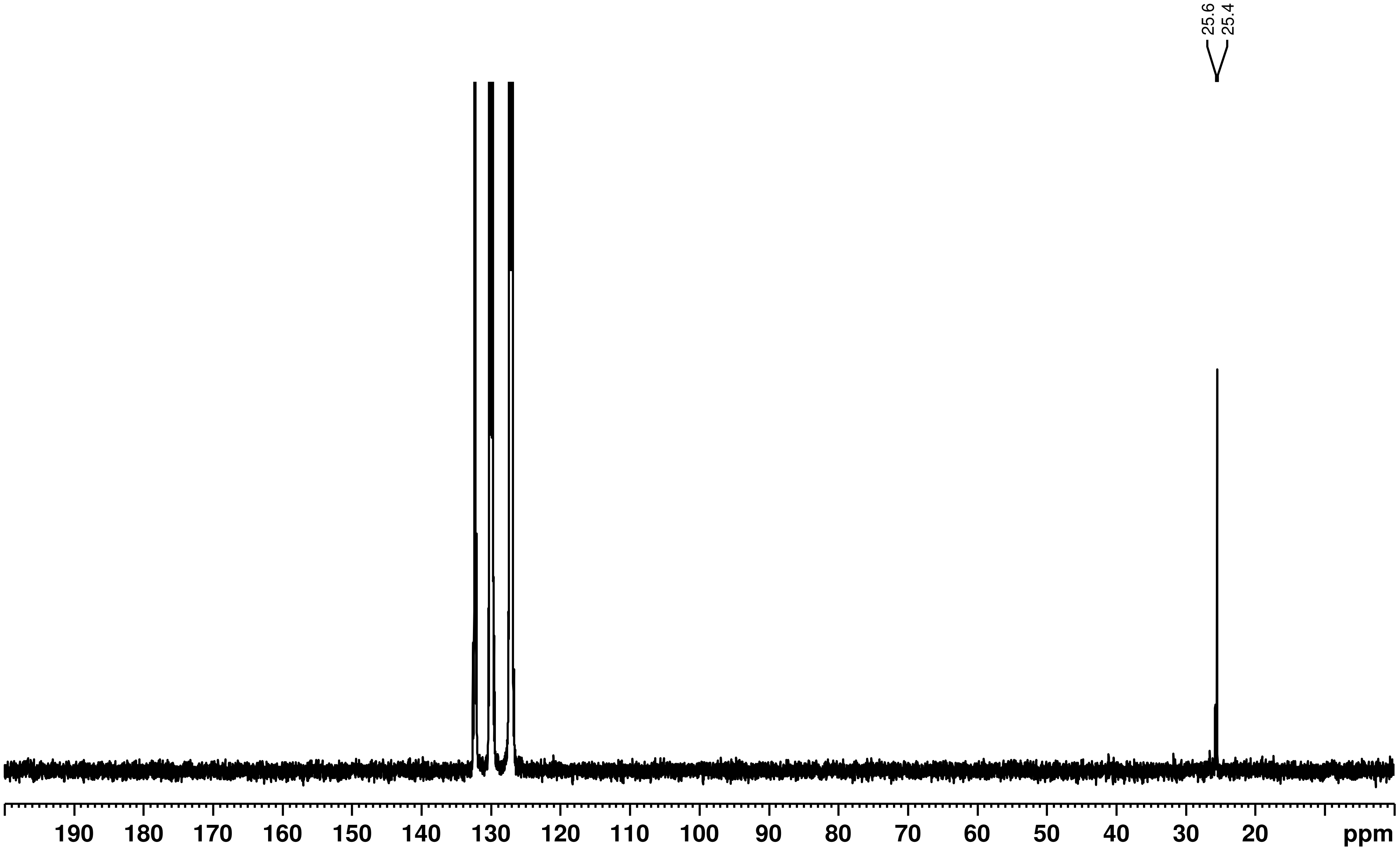

Supplementary Fig. 142. ^{19}**F{^{1}H} NMR** spectrum (471 MHz, 1,2-$C_6D_4Cl_2$, 298 K) of [*t*Bu_2FSi($HCB_{11}H_5Br_6$)] (**8ad**) (* *t*Bu_2SiF_2)

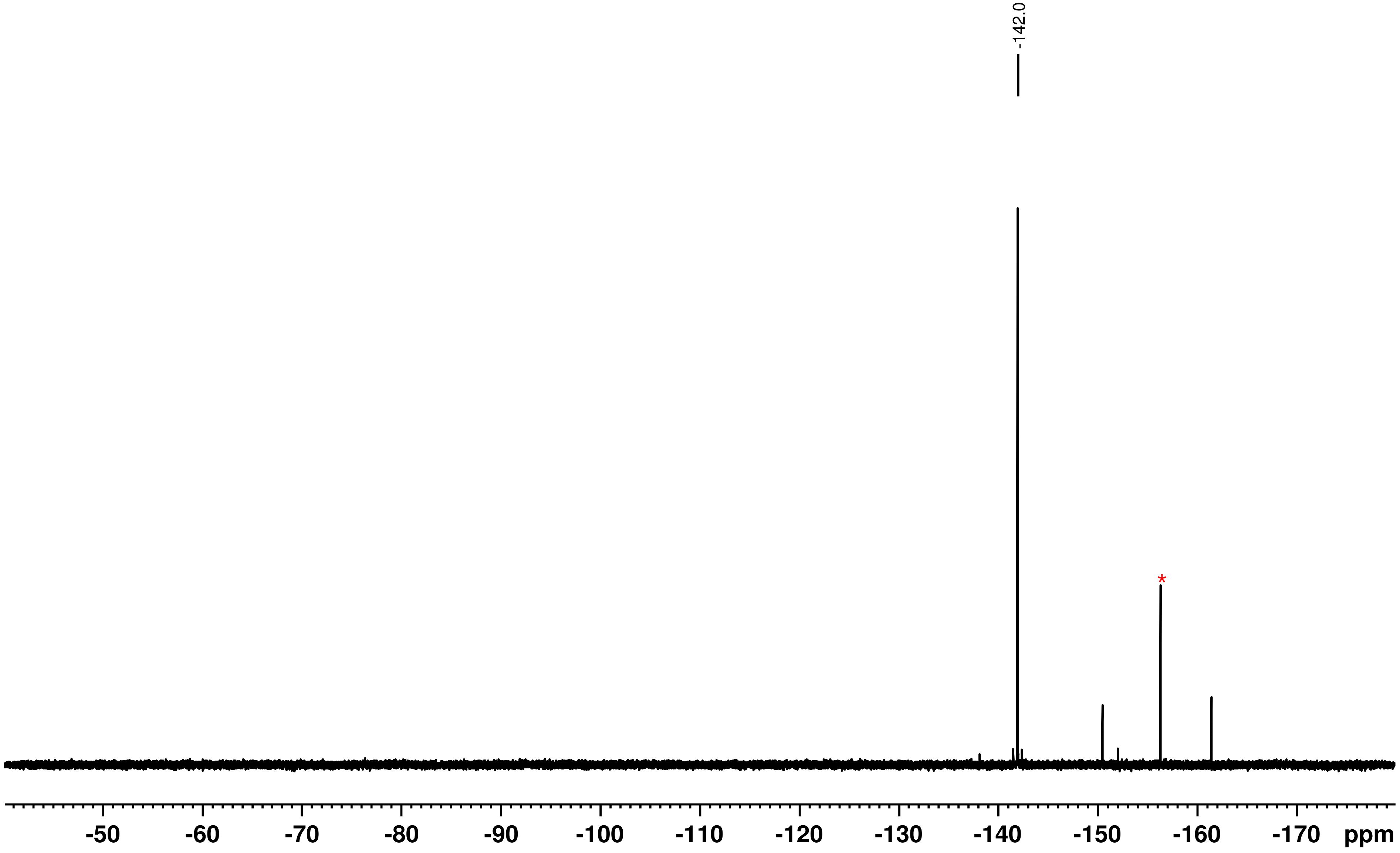

Supplementary Fig. 143. **$^{29}Si\{^{1}H\}$ DEPT NMR** spectrum (99 MHz, 1,2-$C_6D_4Cl_2$, 298 K, optimized for $J_{H,Si}$ = 7 Hz, 13.6°) of [*t*$Bu_2FSi(HCB_{11}H_5Br_6)$] (**8ad**)

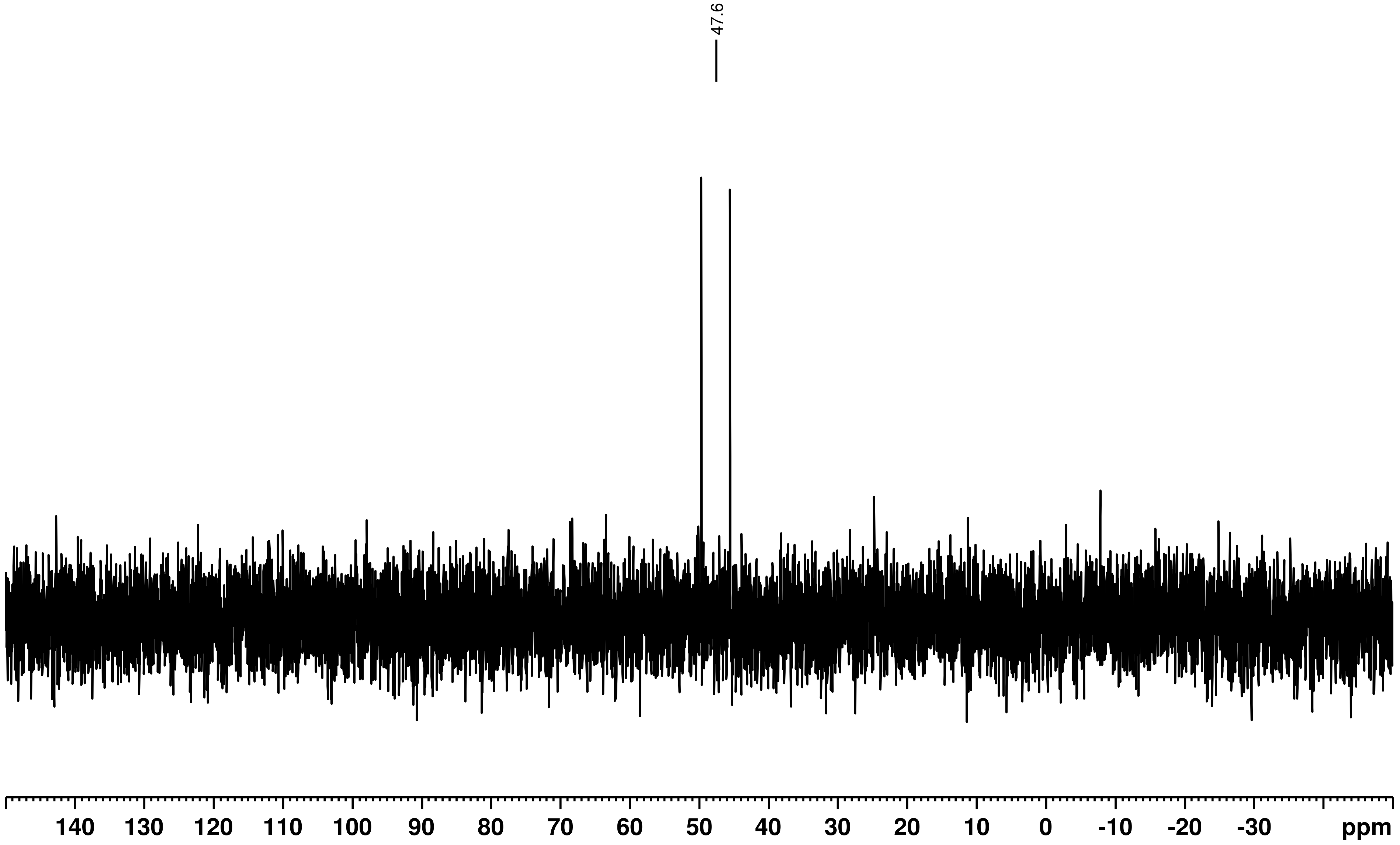

Supplementary Fig. 144. **^{1}H NMR** spectrum (500 MHz, 1,2-$C_6D_4Cl_2$, 298 K) of [$Me_2ClSi(HCB_{11}H_5Br_6)$] (**8ba**)

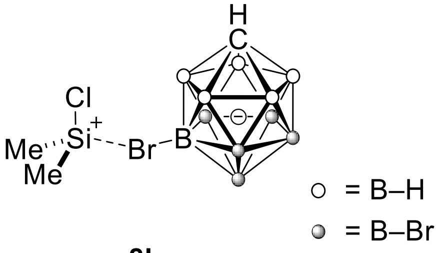

8ba

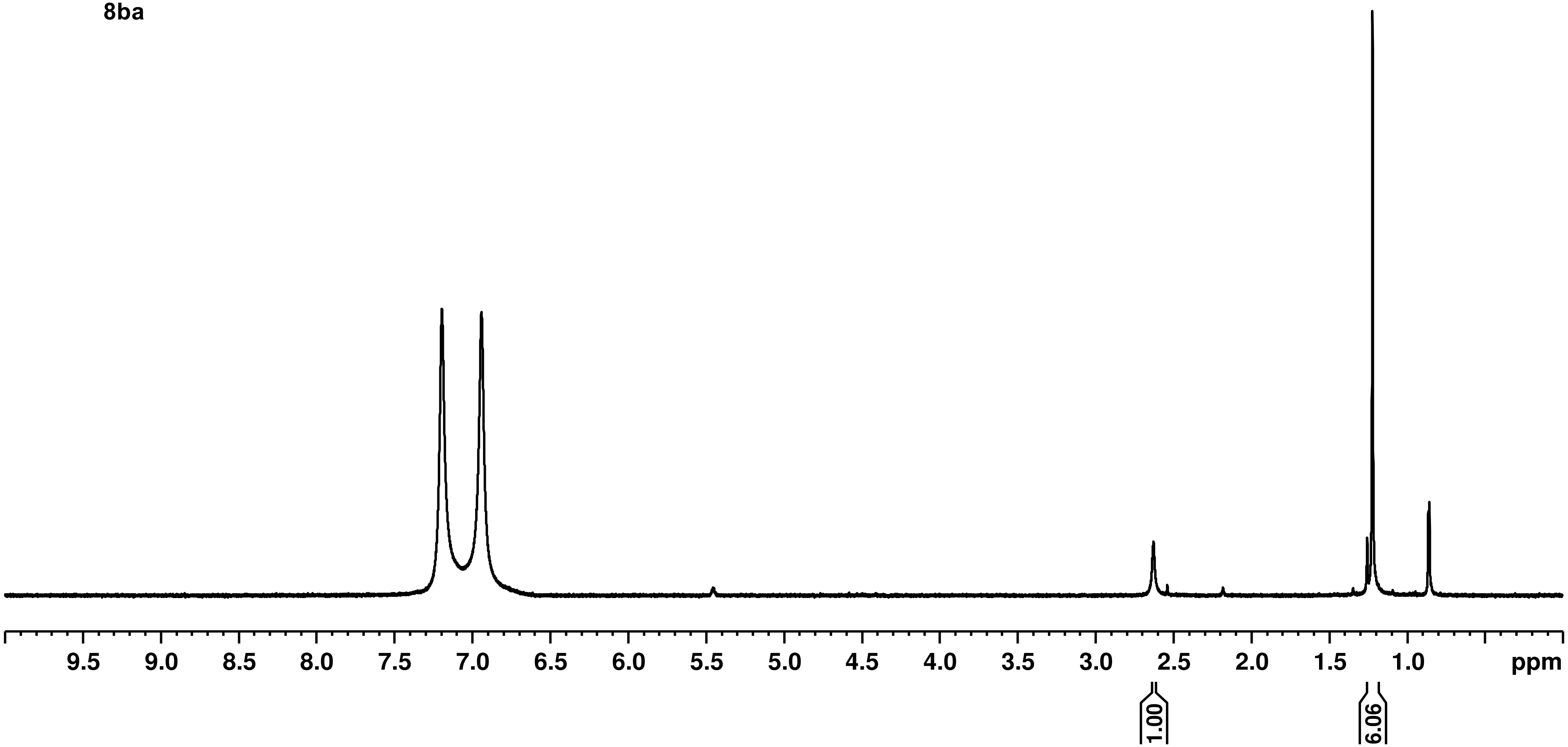

Supplementary Fig. 145. ^{11}**B NMR** spectrum (160 MHz, 1,2-$C_6D_4Cl_2$, 298 K) of [$Me_2ClSi(HCB_{11}H_5Br_6)$] (**8ba**)

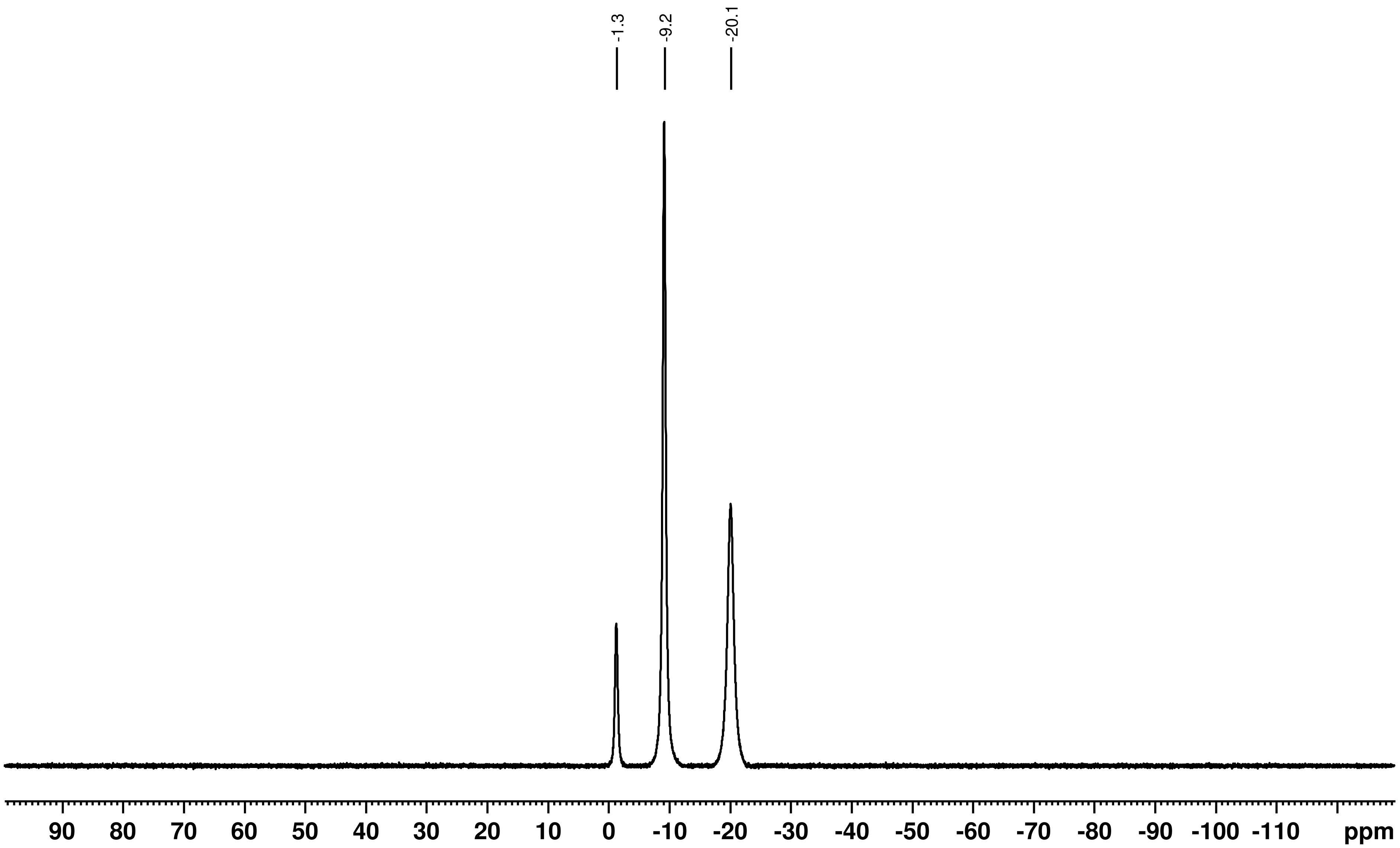

Supplementary Fig. 146. **^{13}C{^{1}H} NMR** spectrum (126 MHz, 1,2-$C_6D_4Cl_2$, 298 K) of [$Me_2ClSi(HCB_{11}H_5Br_6)$] (**8ba**)

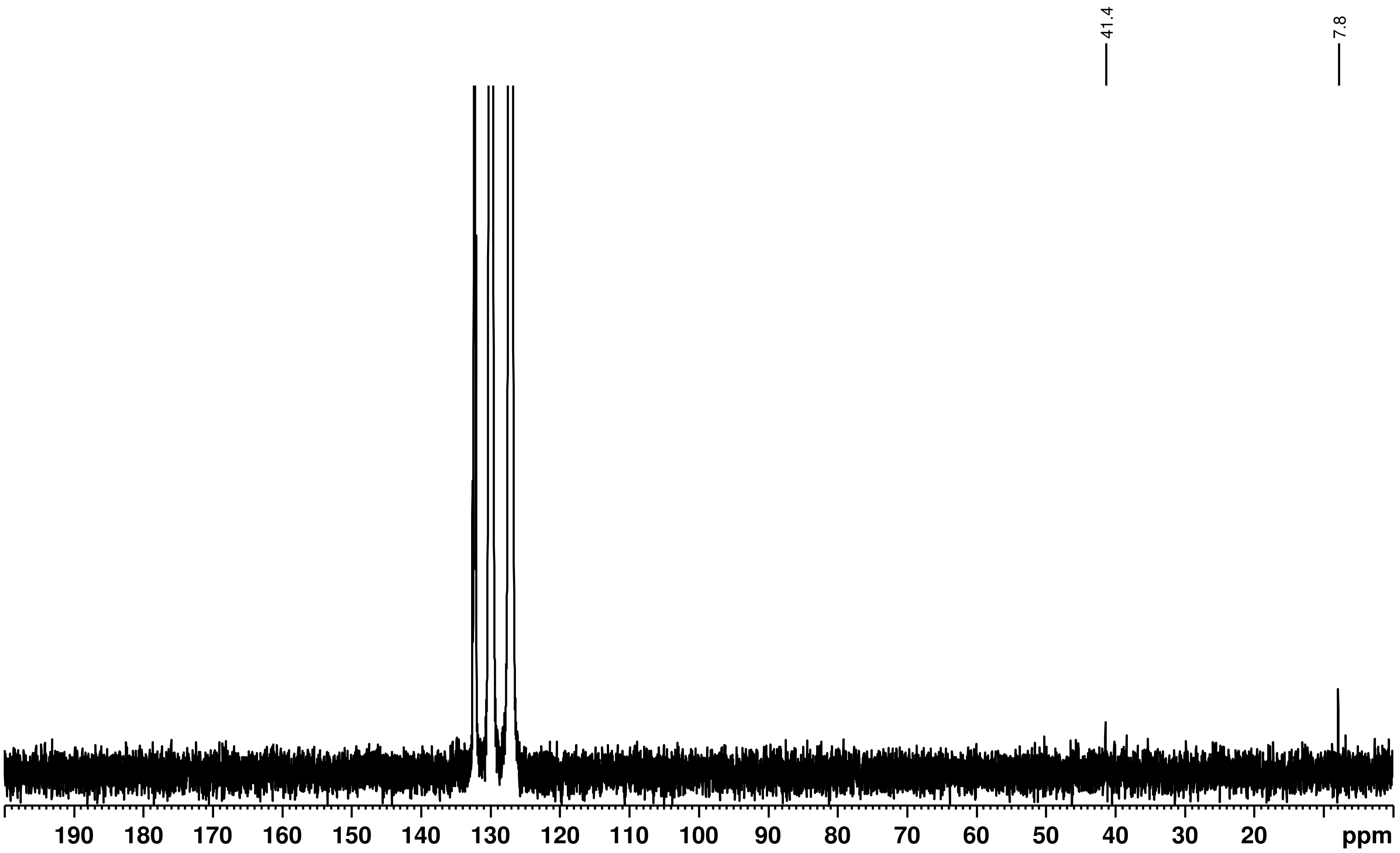

Supplementary Fig. 147. **$^{29}Si\{^1H\}$ DEPT NMR** spectrum (99 MHz, 1,2-$C_6D_4Cl_2$, 298 K, optimized for $J_{H,Si}$ = 7 Hz, 24.1°) of [$Me_2ClSi(HCB_{11}H_5Br_6)$] (**8ba**)

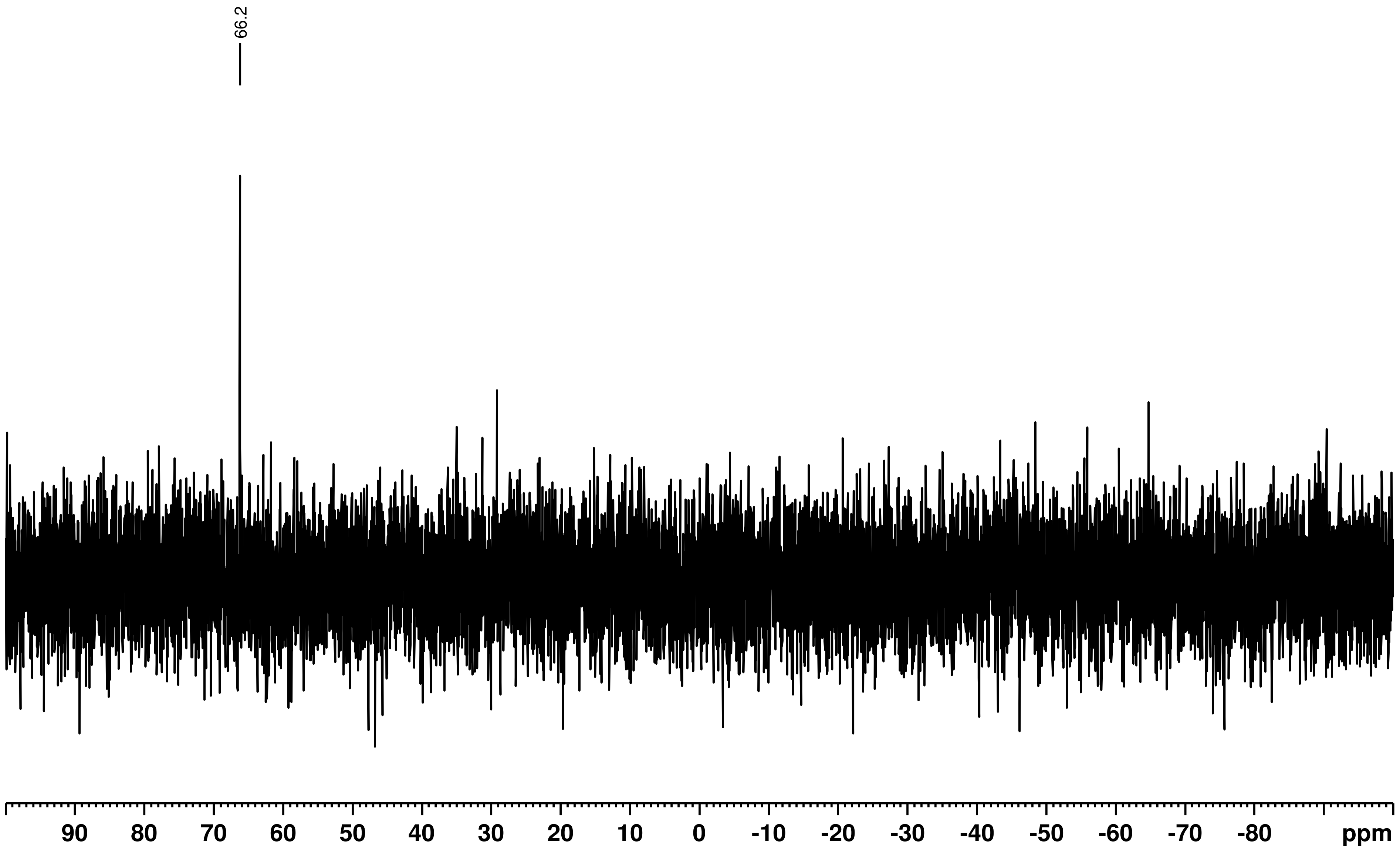

Supplementary Fig. 148. **^{1}H NMR** spectrum (500 MHz, 1,2-$C_6D_4Cl_2$, 298 K) of [$Et_2ClSi(HCB_{11}H_5Br_6)$] (**8bb**)

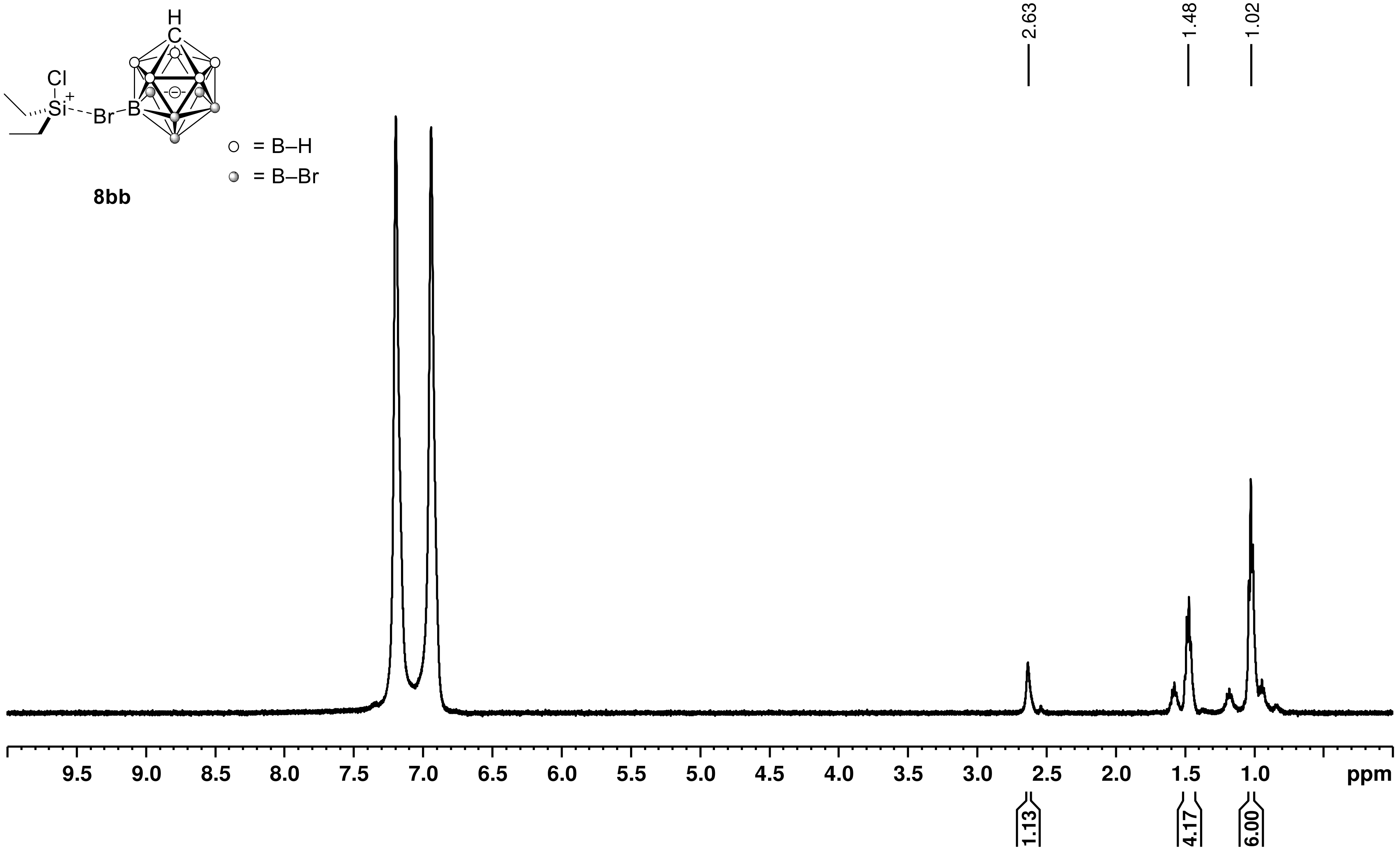

Supplementary Fig. 149. **^{11}B NMR** spectrum (160 MHz, 1,2-$C_6D_4Cl_2$, 298 K) of [$Et_2ClSi(HCB_{11}H_5Br_6)$] (**8bb**)

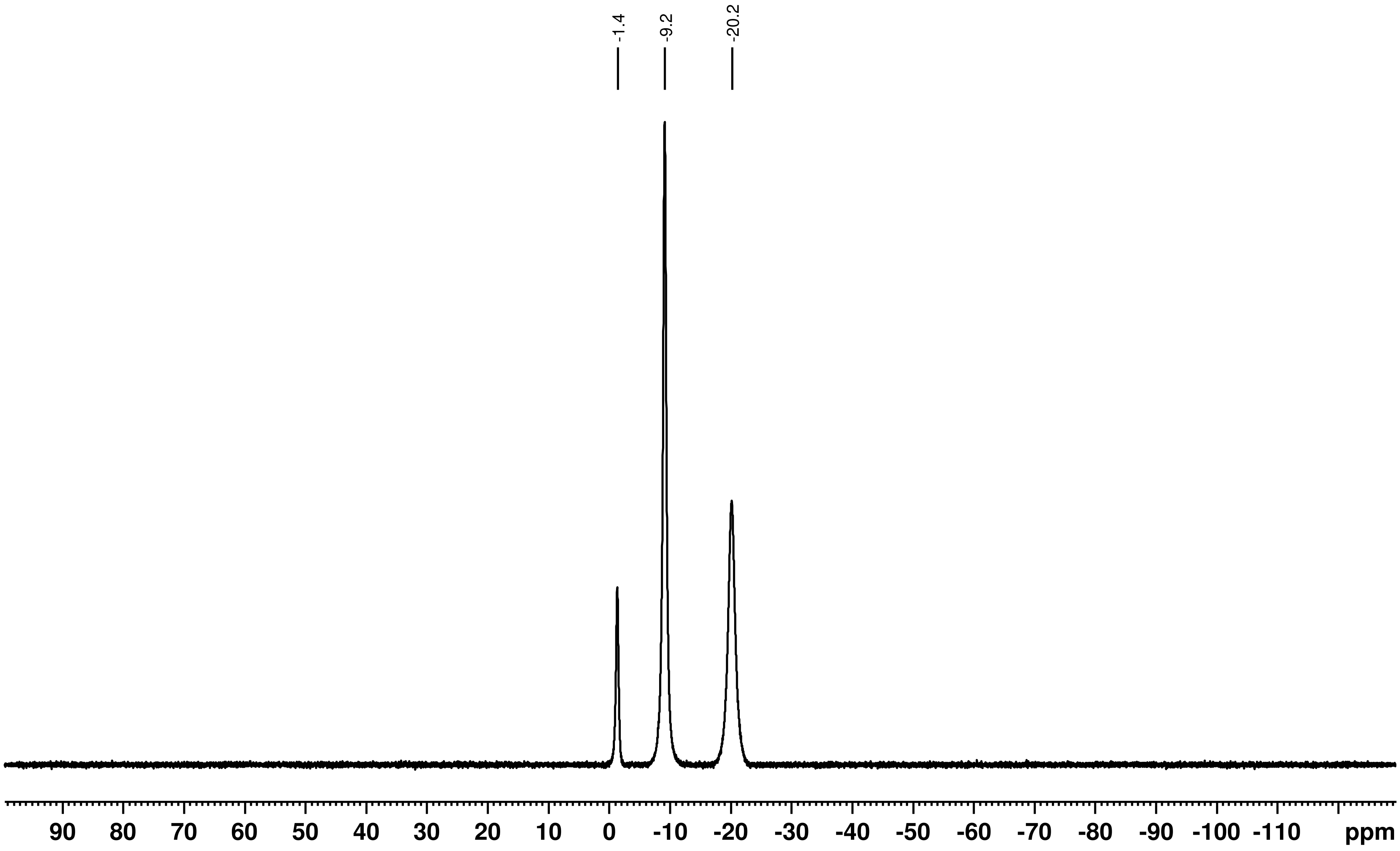

Supplementary Fig. 150. **^{13}C{^{1}H} NMR** spectrum (126 MHz, 1,2-$C_6D_4Cl_2$, 298 K) of [$Et_2ClSi(HCB_{11}H_5Br_6)$] (**8bb**)

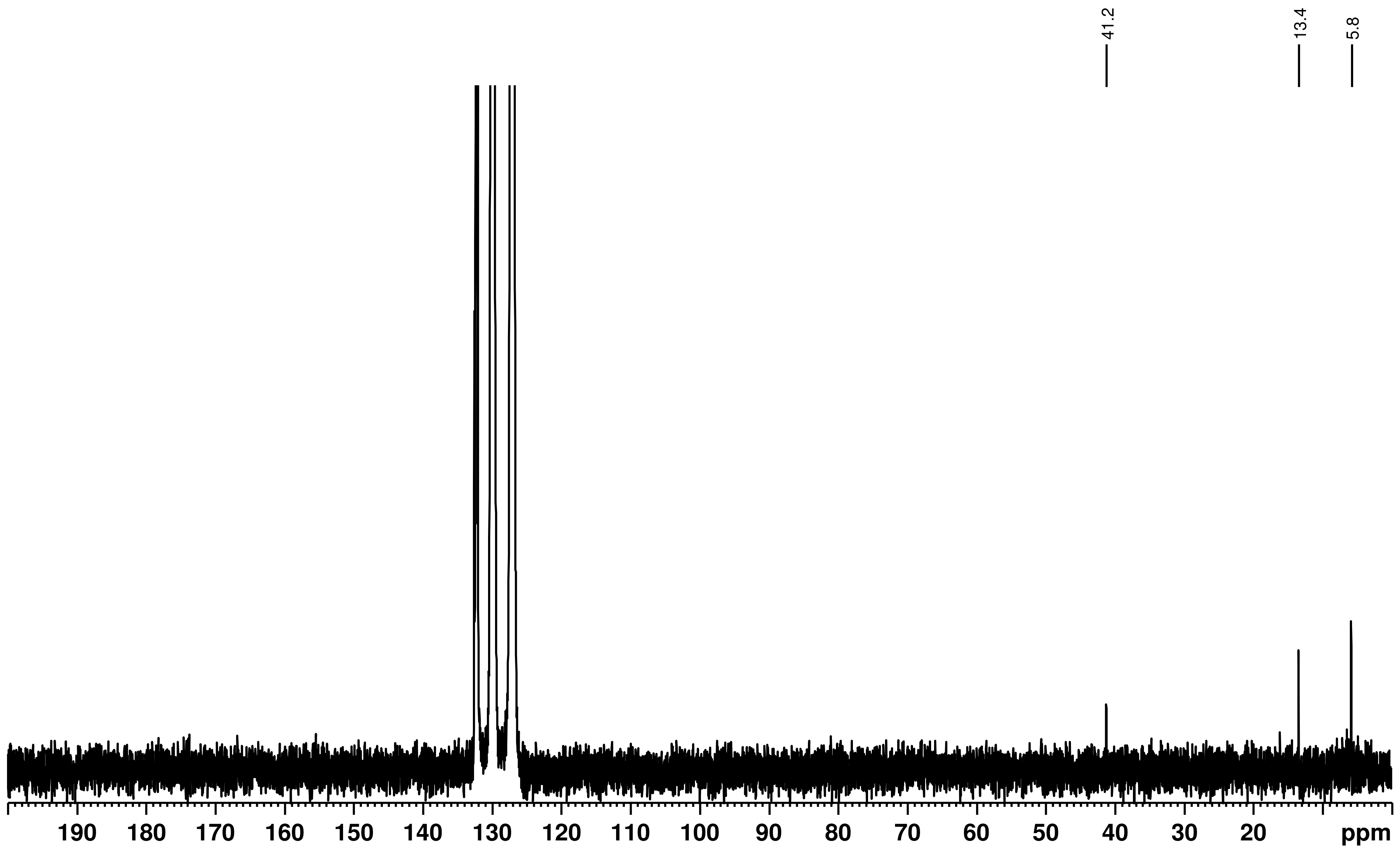

Supplementary Fig. 151. **$^{1}H,^{29}Si$ HMQC NMR** (500/99 MHz, 1,2-$C_6D_4Cl_2$, 298 K, optimized for *J* = 7 Hz) of [$Et_2ClSi(HCB_{11}H_5Br_6)$] (**8bb**)

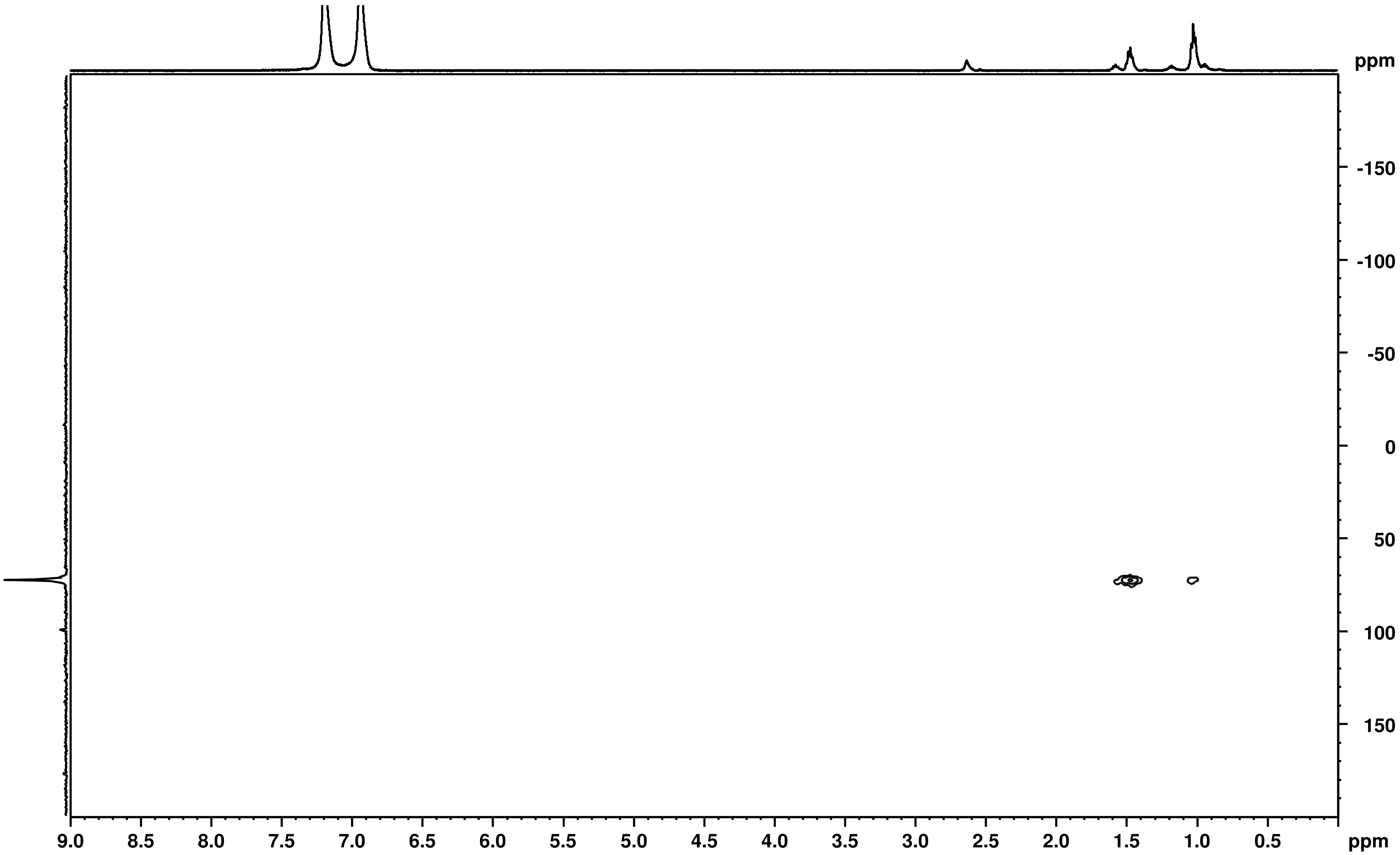

Supplementary Fig. 152. **^{1}H NMR** spectrum (500 MHz, 1,2-$C_6D_4Cl_2$, 298 K) of [*i*$Pr_2ClSi(HCB_{11}H_5Br_6)$] (**8bc**)

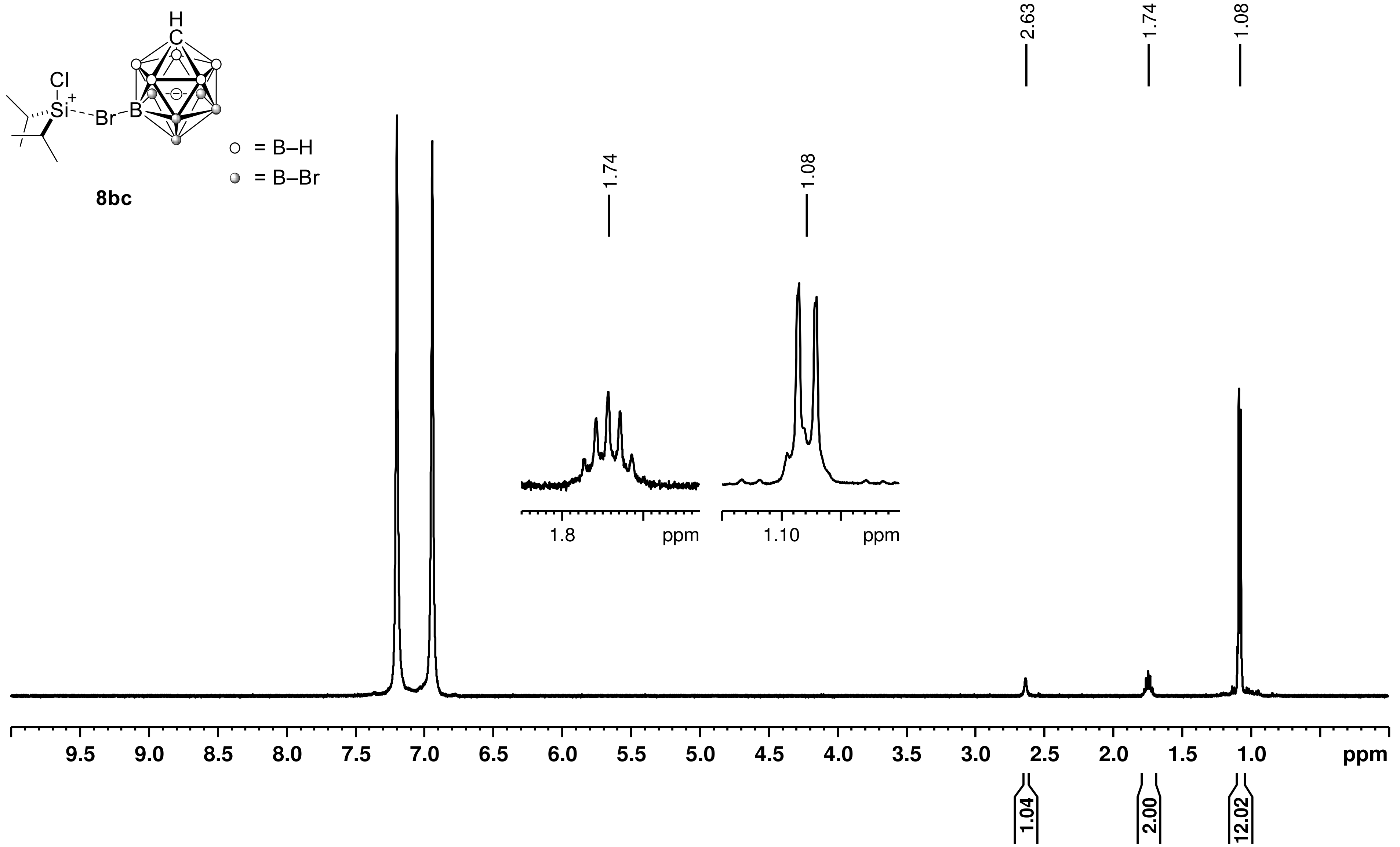

Supplementary Fig. 153. ^{11}**B NMR** spectrum (160 MHz, 1,2-$C_6D_4Cl_2$, 298 K) of [*i*$Pr_2ClSi(HCB_{11}H_5Br_6)$] (**8bc**)

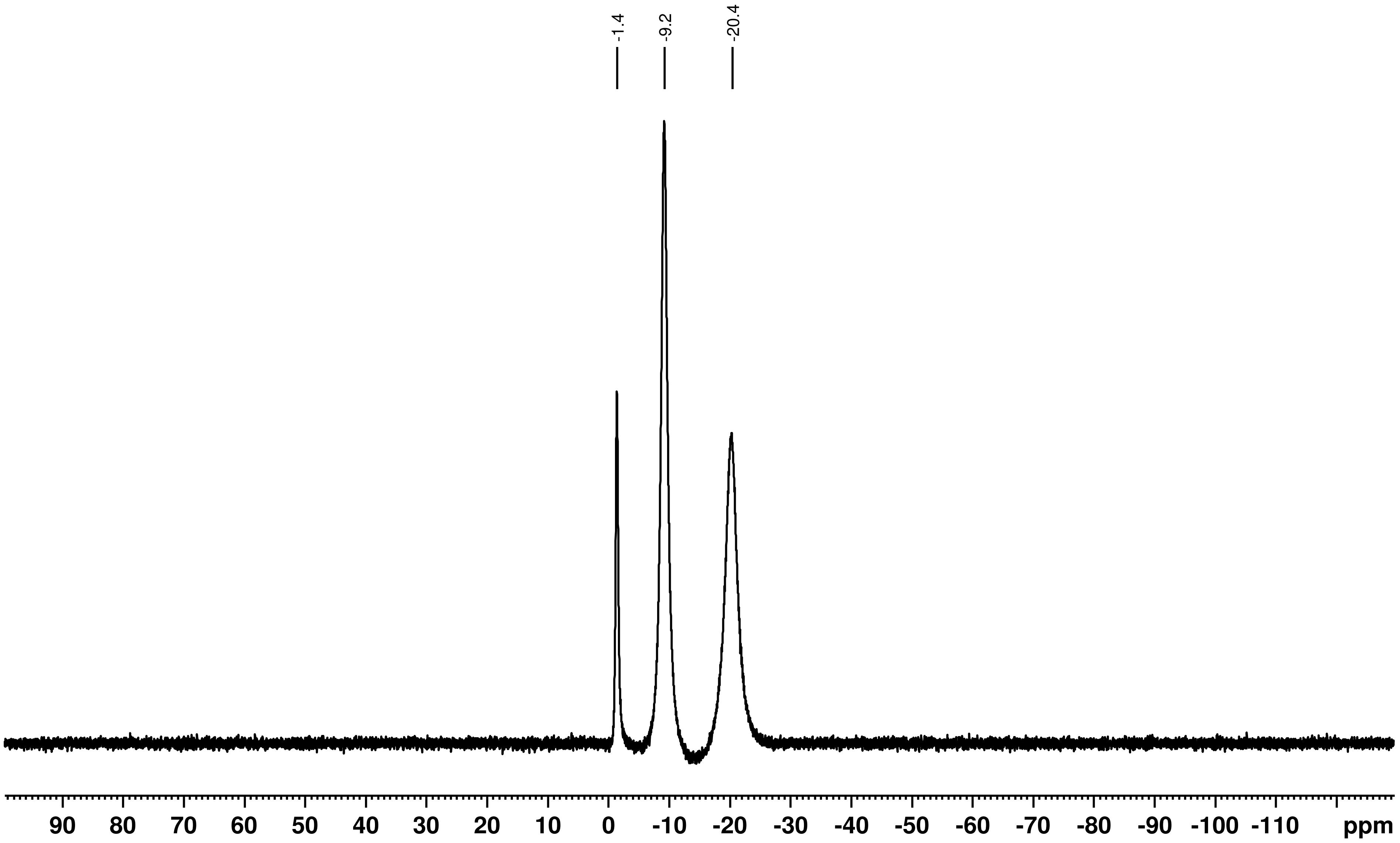

Supplementary Fig. 154. **$^{13}C\{^{1}H\}$ NMR** spectrum (126 MHz, 1,2-$C_6D_4Cl_2$, 298 K) of [*i*$Pr_2ClSi(HCB_{11}H_5Br_6)$] (**8bc**)

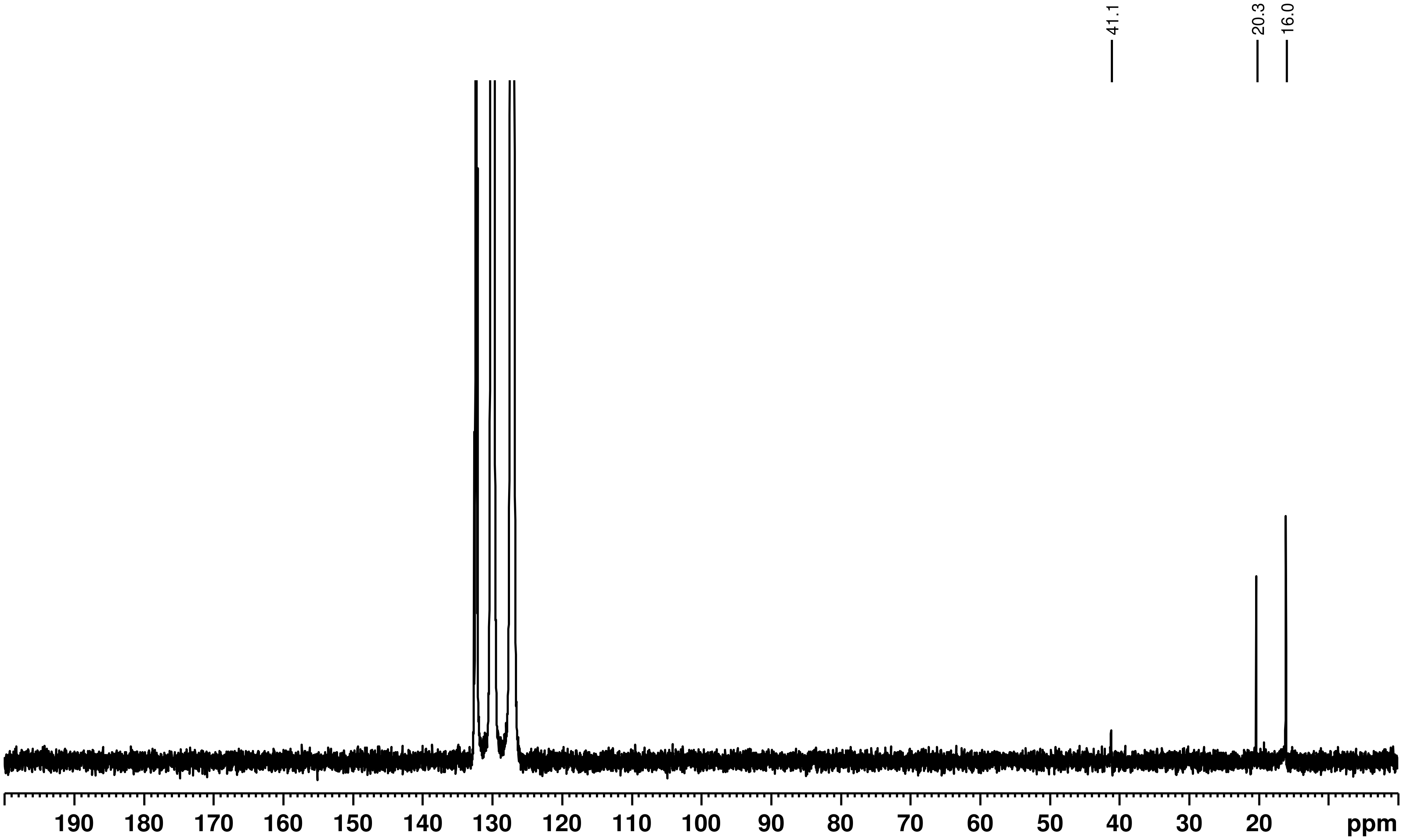

Supplementary Fig. 155. **^{1}H,^{29}Si HMQC NMR** (500/99 MHz, 1,2-$C_6D_4Cl_2$, 298 K, optimized for *J* = 7 Hz) of [*i*$Pr_2ClSi(HCB_{11}H_5Br_6)$] (**8bc**)

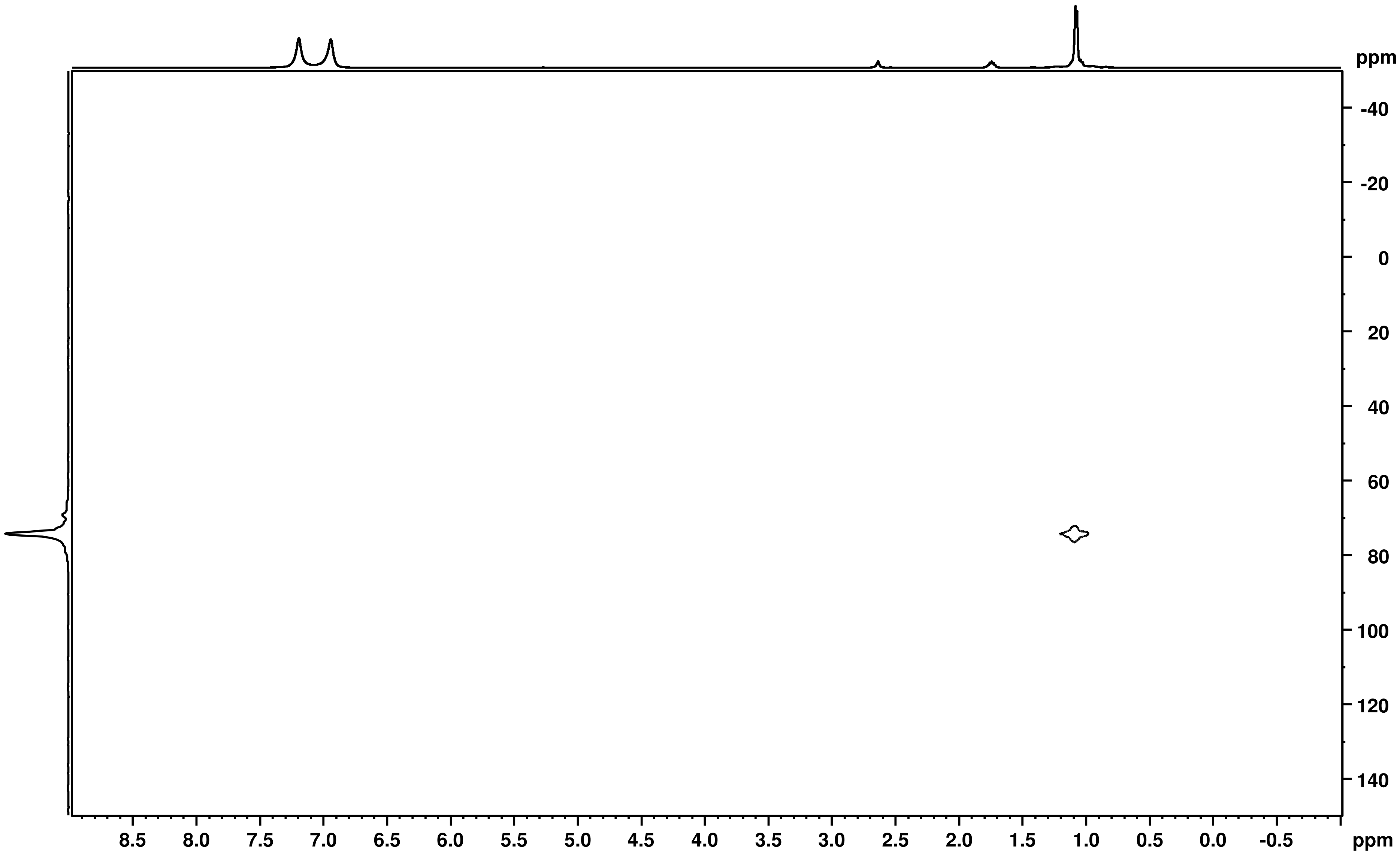

Supplementary Fig. 156. ^{1}**H NMR** spectrum (500 MHz, 1,2-$C_6D_4Cl_2$, 298 K) of [$t$$Bu_2ClSi(HCB_{11}H_5Br_6)$] (**8bd**)

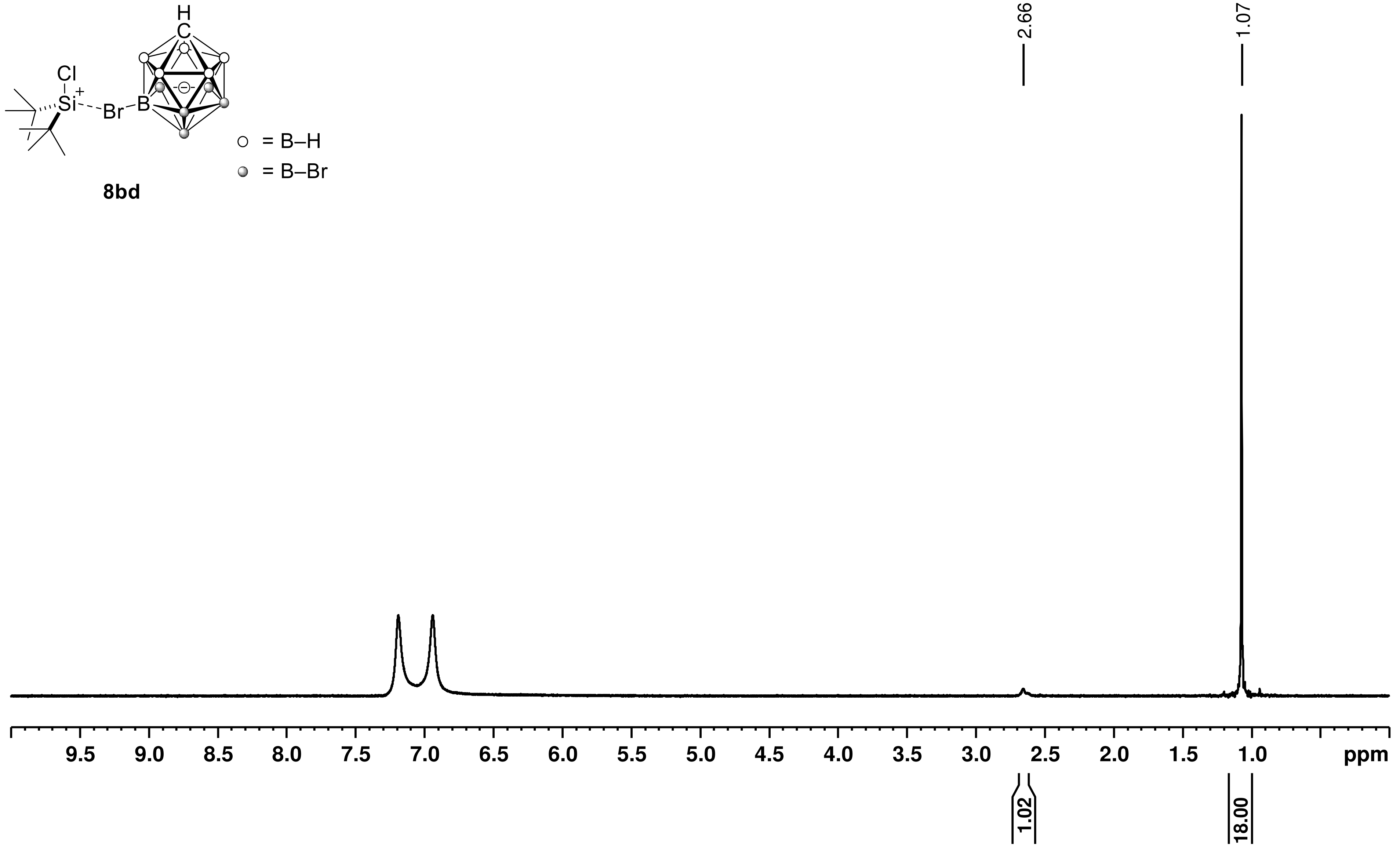

Supplementary Fig. 157. ^{11}**B NMR** spectrum (160 MHz, 1,2-$C_6D_4Cl_2$, 298 K) of [$t$$Bu_2ClSi(HCB_{11}H_5Br_6)$] (**8bd**)

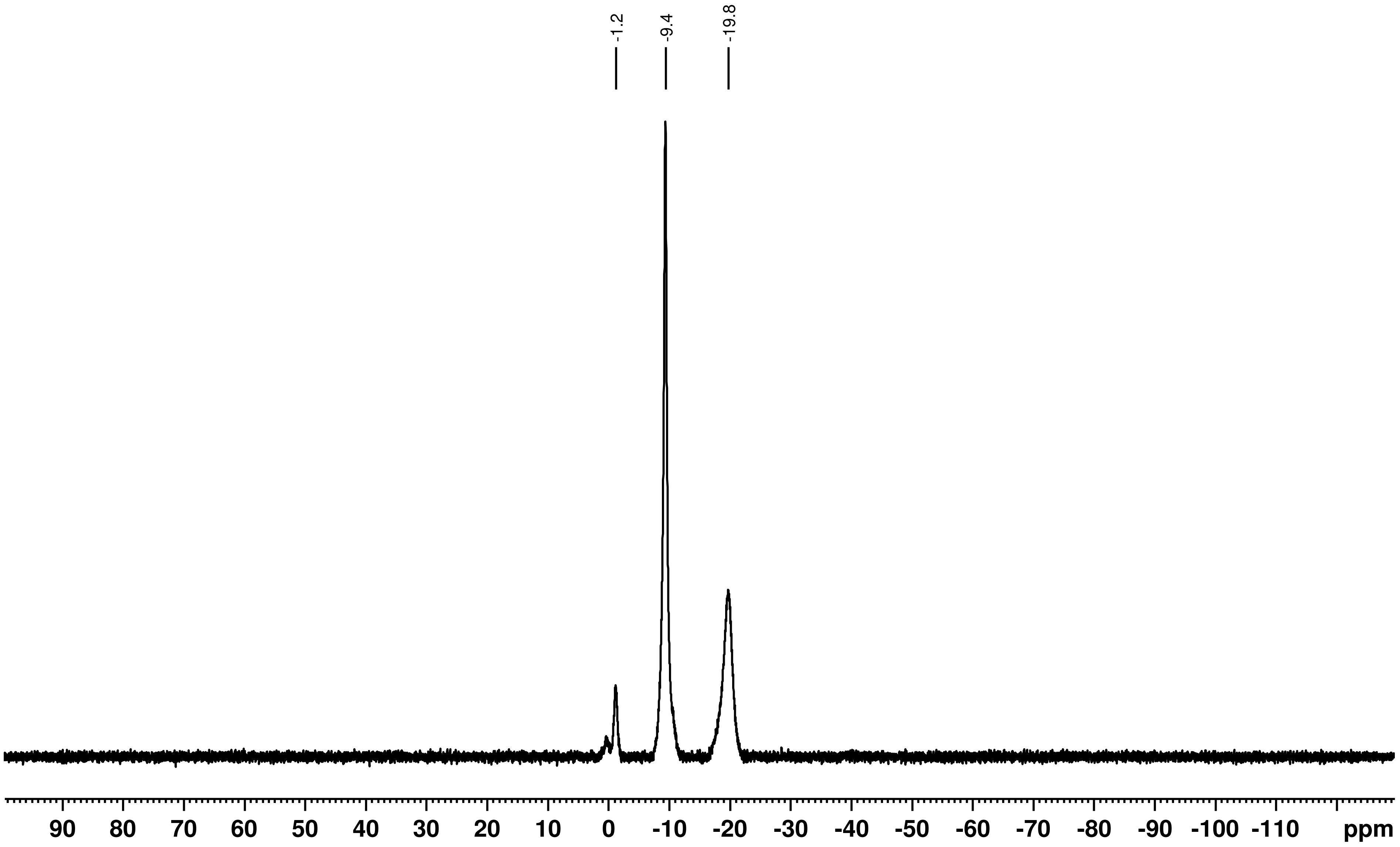

Supplementary Fig. 158. **$^{13}C\{^1H\}$ NMR** spectrum (126 MHz, 1,2-$C_6D_4Cl_2$, 298 K) of [$t$$Bu_2ClSi(HCB_{11}H_5Br_6)$] (**8bd**)

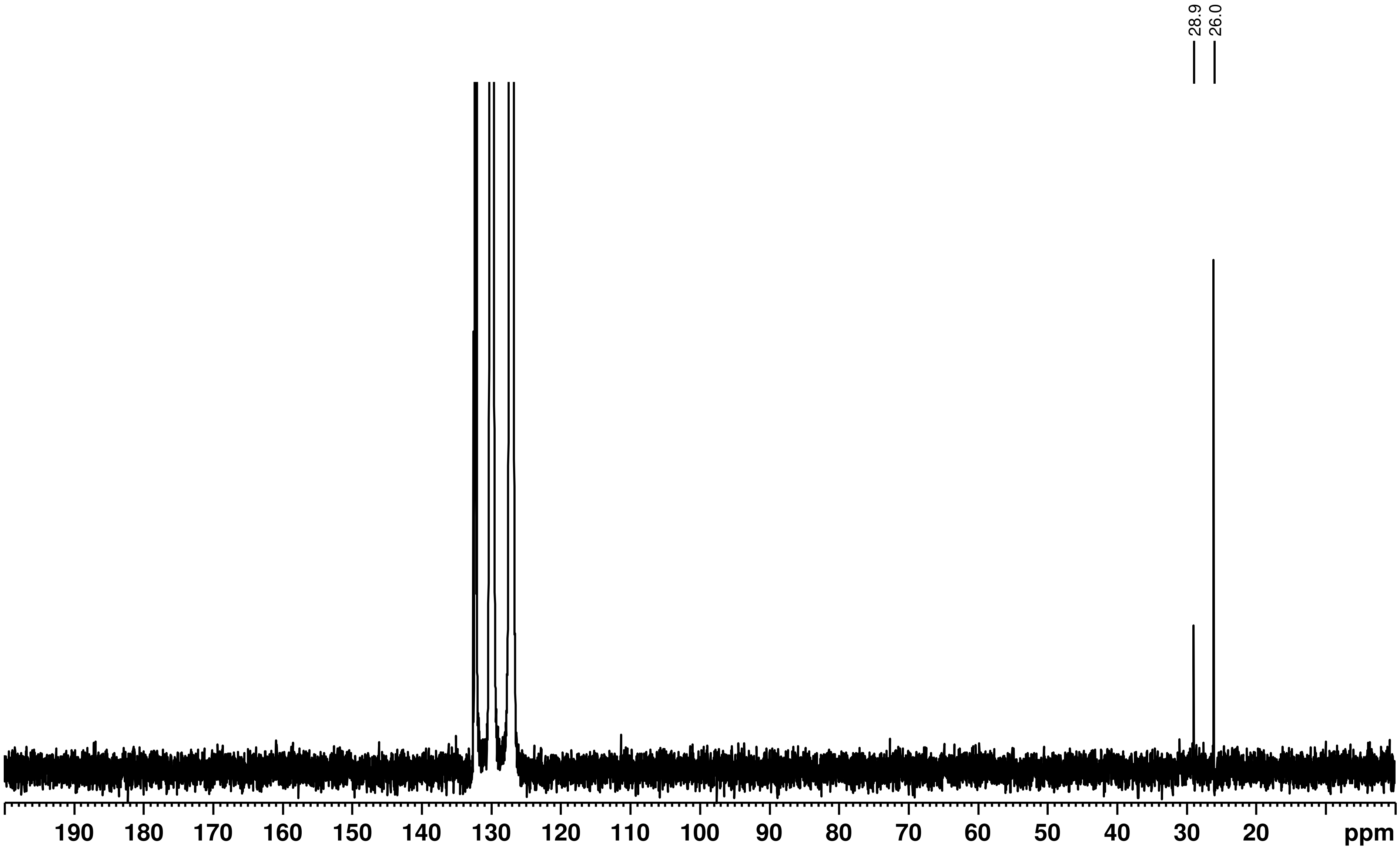

Supplementary Fig. 159. **^{29}Si{^{1}H} DEPT NMR** spectrum (99 MHz, 1,2-$C_6D_4Cl_2$, 298 K, optimized for $J_{H,Si}$ = 7 Hz, 13.6°) of [*t*$Bu_2ClSi(HCB_{11}H_5Br_6)$] (**8bd**)

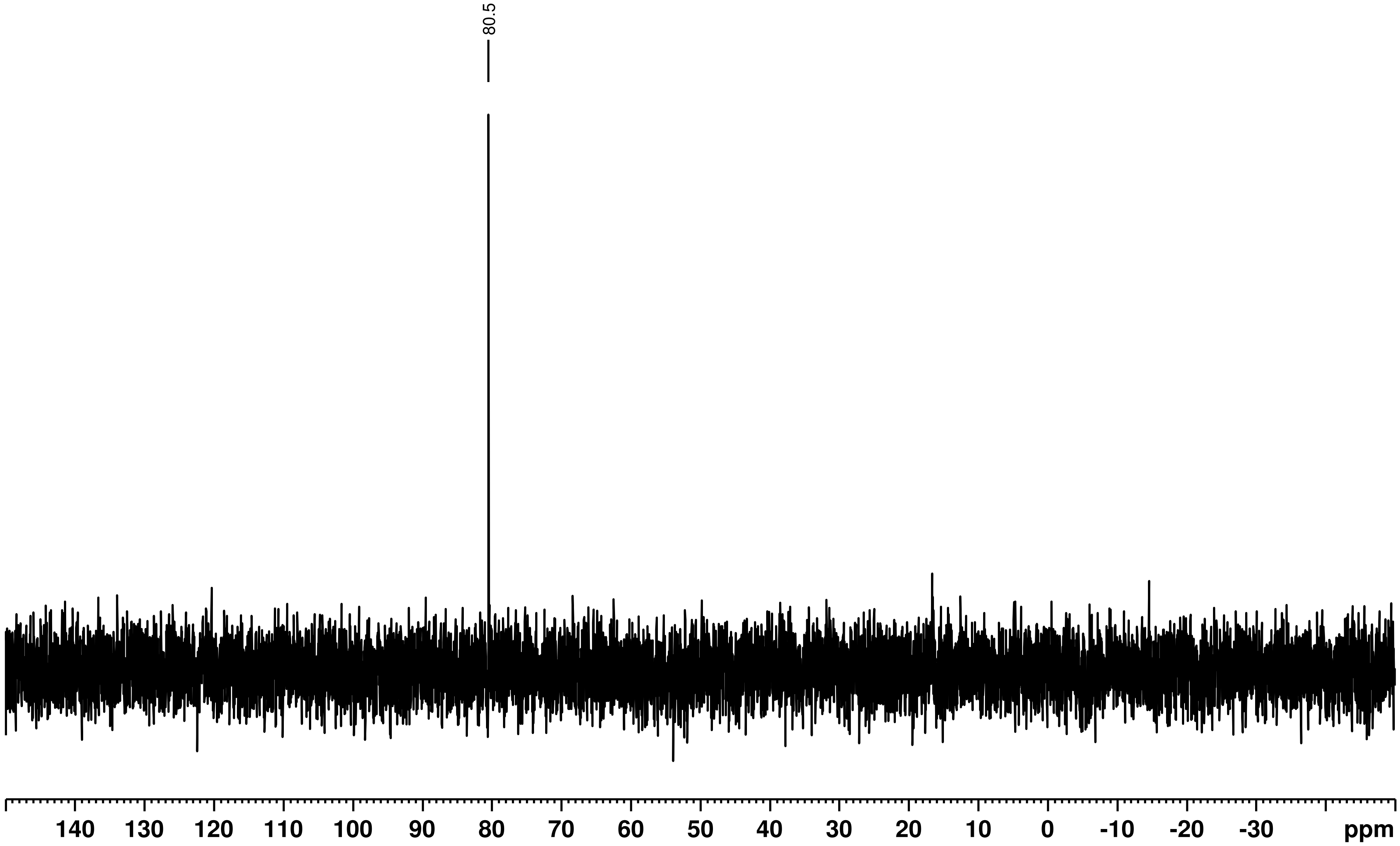

Supplementary Fig. 160. **^{1}H NMR** spectrum (500 MHz, 1,2-$C_6D_4Cl_2$, 298 K) of [$Me_2BrSi(HCB_{11}H_5Br_6)$] (**8ca**)

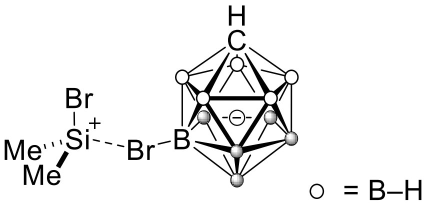

8ca

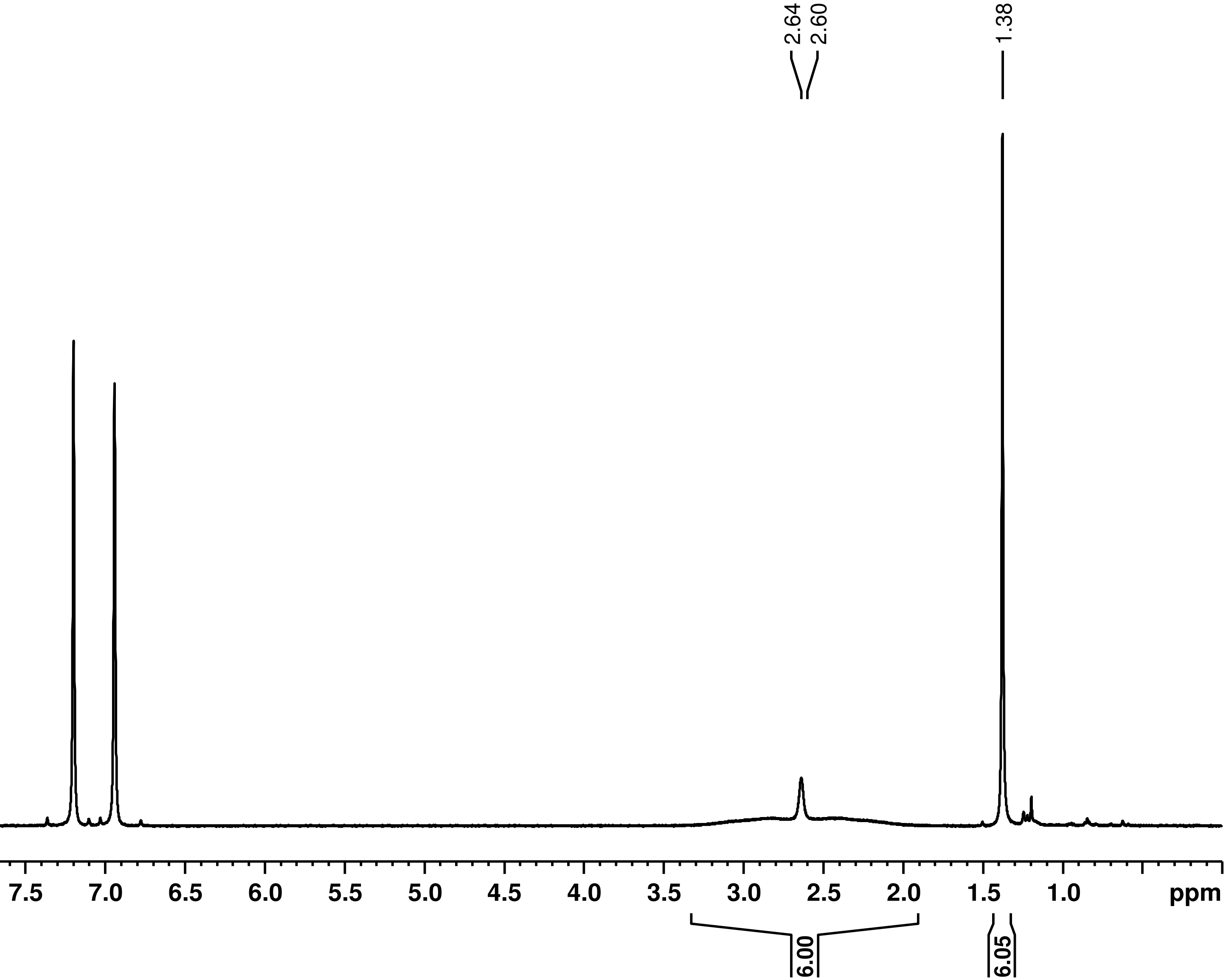

Supplementary Fig. 161. ^{11}**B NMR** spectrum (160 MHz, 1,2-$C_6D_4Cl_2$, 298 K) of [$Me_2BrSi(HCB_{11}H_5Br_6)$] (**8ca**)

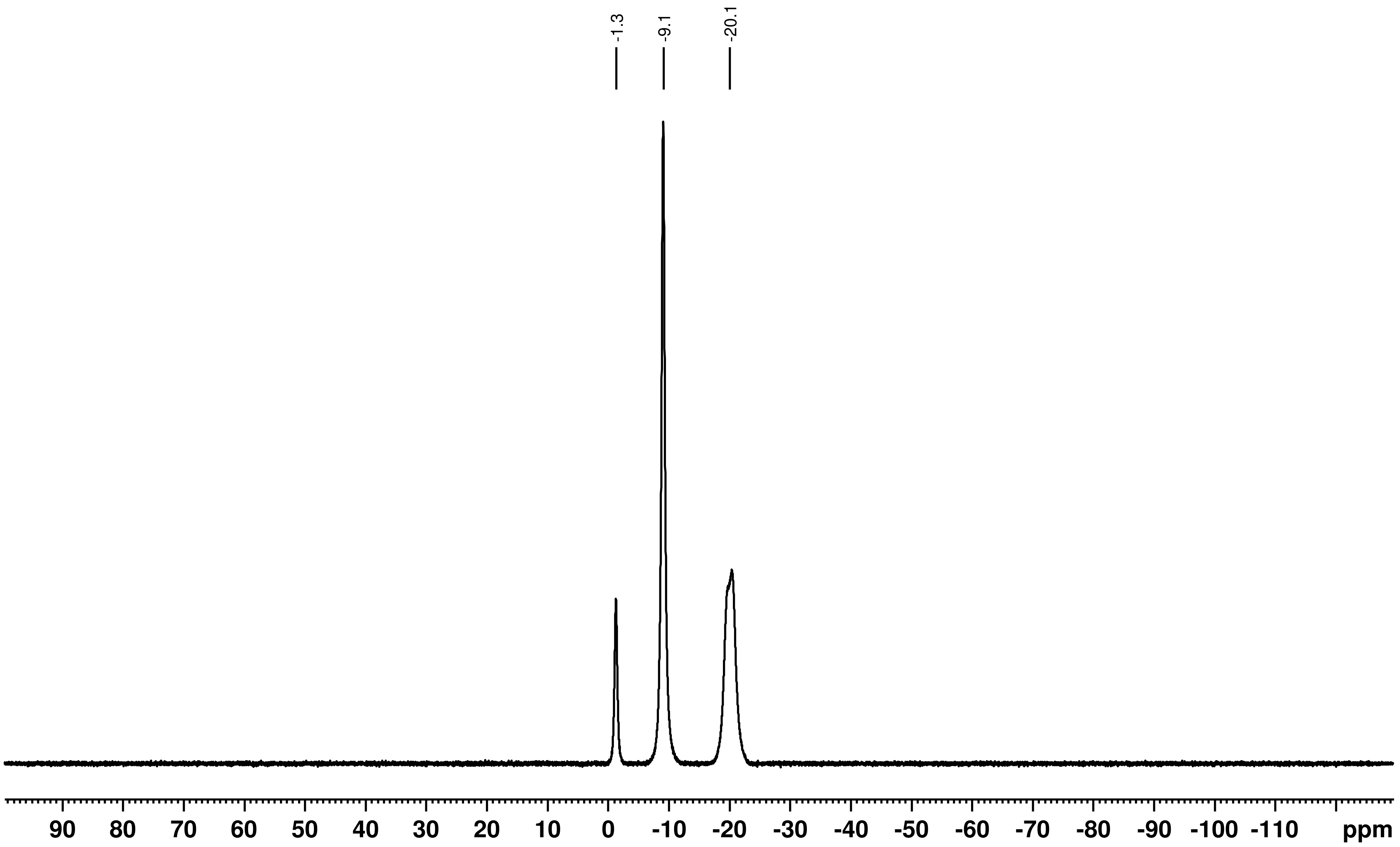

Supplementary Fig. 162. **^{13}C{^{1}H} NMR** spectrum (126 MHz, 1,2-$C_6D_4Cl_2$, 298 K) of [$Me_2BrSi(HCB_{11}H_5Br_6)$] (**8ca**)

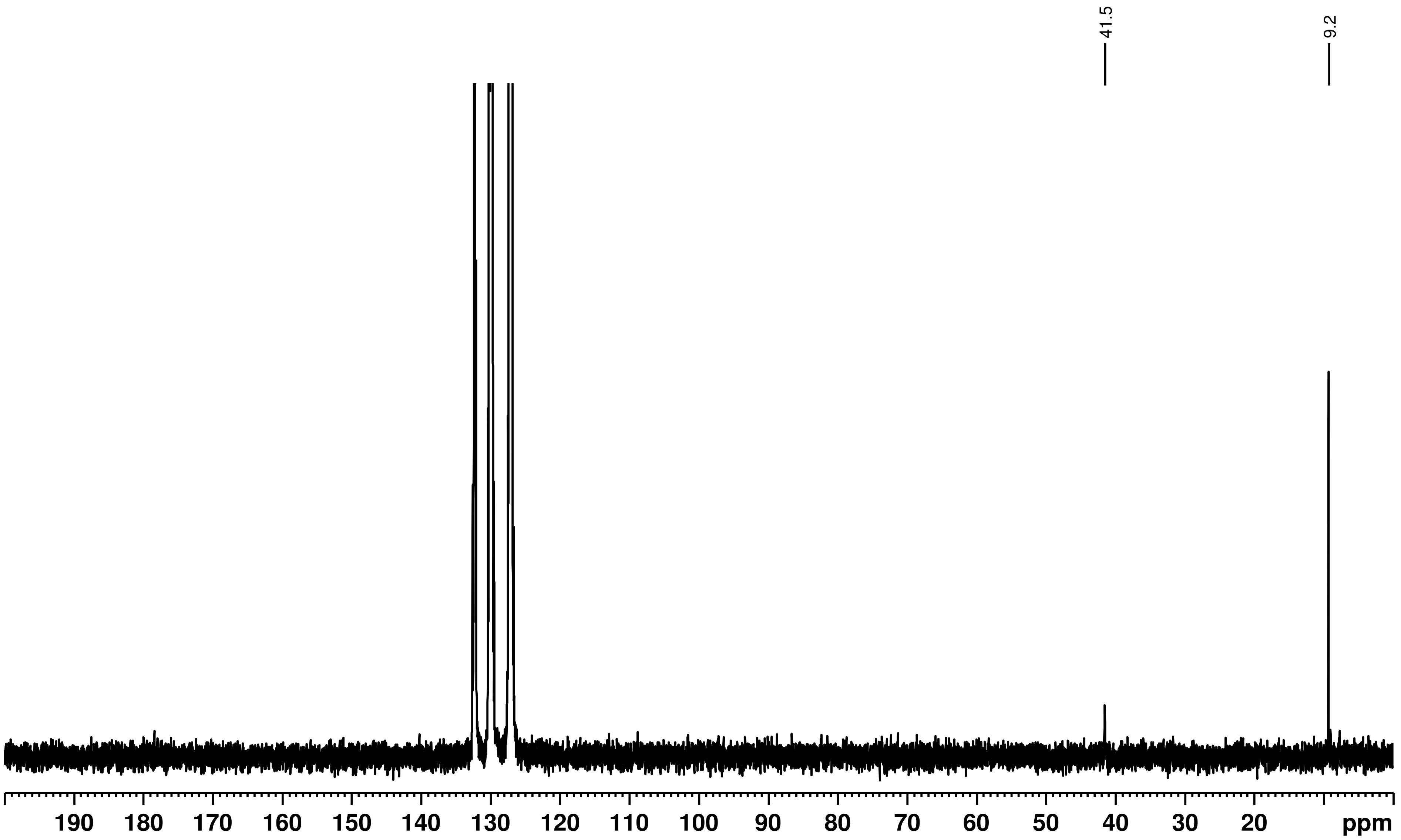

Supplementary Fig. 163. **$^{29}Si\{^1H\}$ DEPT NMR** spectrum (99 MHz, 1,2-$C_6D_4Cl_2$, 298 K, optimized for $J_{H,Si}$ = 7 Hz, 24.1°) of [$Me_2BrSi(HCB_{11}H_5Br_6)$] (**8ca**)

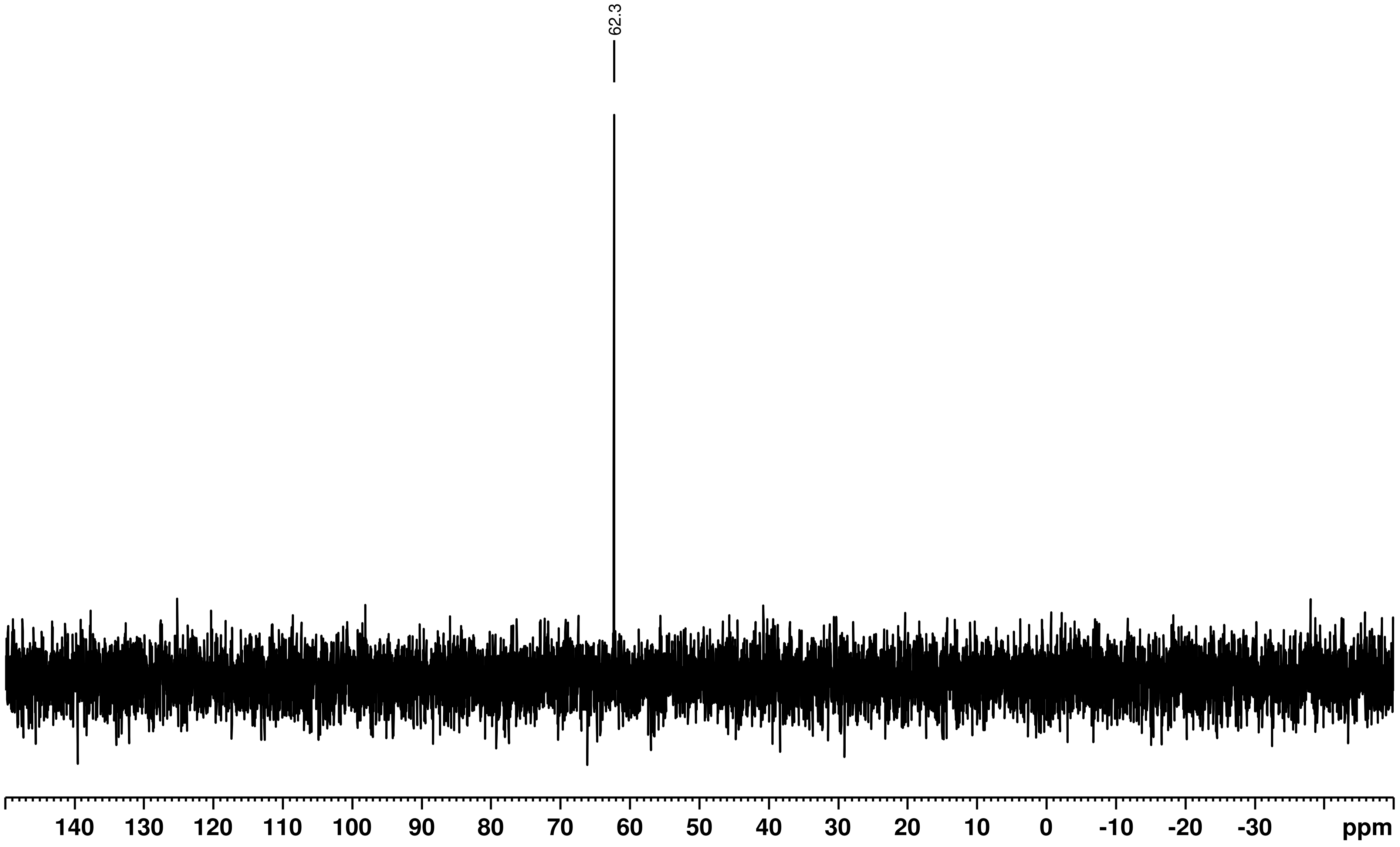

Supplementary Fig. 164. **^{1}H NMR** spectrum (500 MHz, 1,2-$C_6D_4Cl_2$, 298 K) of [$Et_2BrSi(HCB_{11}H_5Br_6)$] (**8cb**)

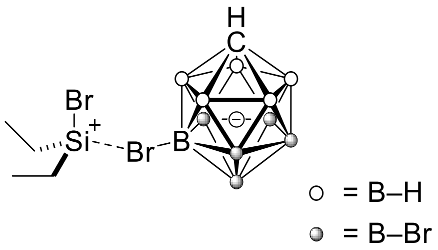

8cb

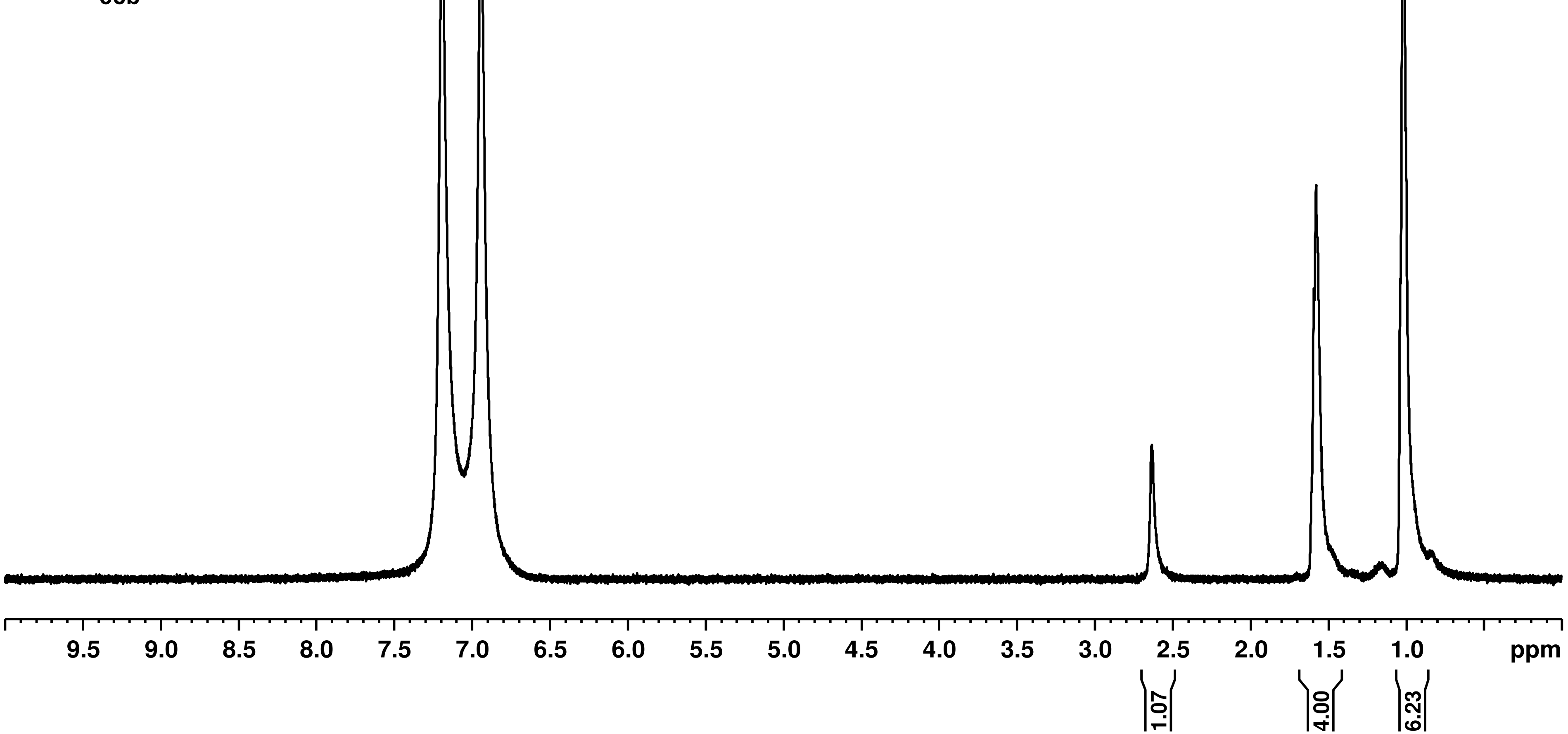

Supplementary Fig. 165. [11]**B NMR** spectrum (160 MHz, 1,2-$C_6D_4Cl_2$, 298 K) of [$Et_2BrSi(HCB_{11}H_5Br_6)$] (**8cb**)

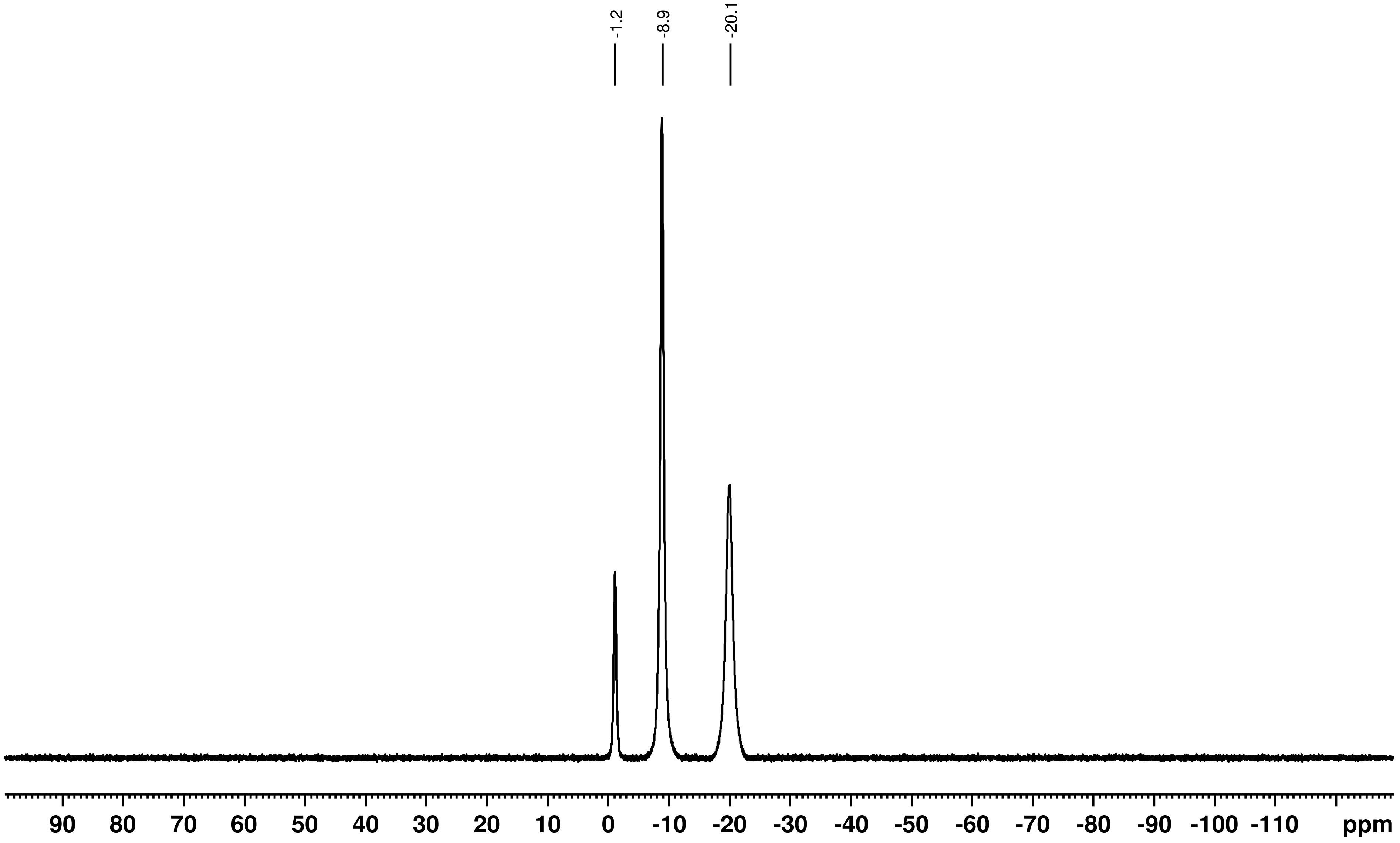

Supplementary Fig. 166. **$^{13}C\{^{1}H\}$ NMR** spectrum (126 MHz, 1,2-$C_6D_4Cl_2$, 298 K) of [$Et_2BrSi(HCB_{11}H_5Br_6)$] (**8cb**)

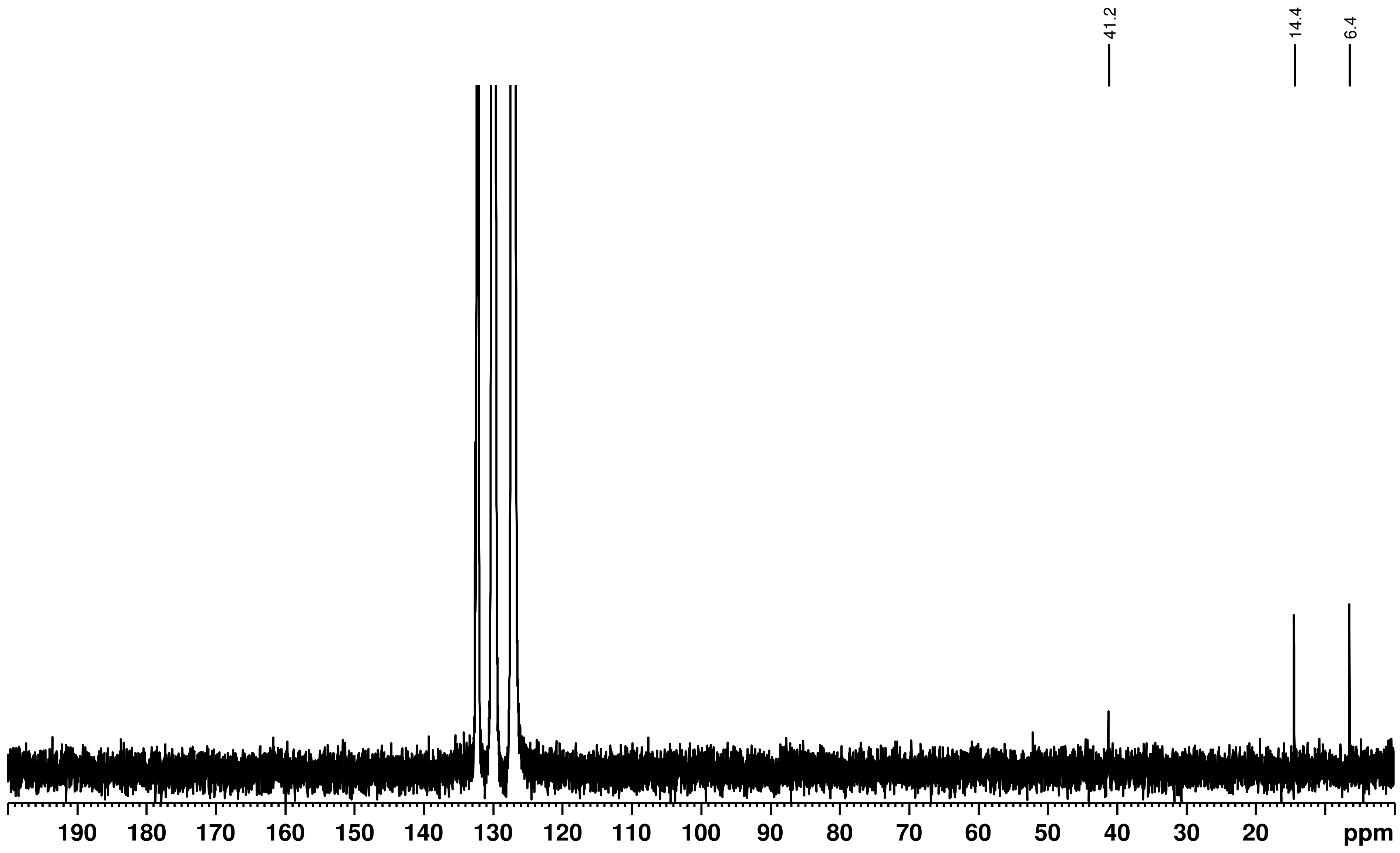

Supplementary Fig. 167. **^{1}H,^{29}Si HMQC NMR** (500/99 MHz, 1,2-$C_6D_4Cl_2$, 298 K, optimized for *J* = 7 Hz) of [$Et_2BrSi(HCB_{11}H_5Br_6)$] (**8cb**)

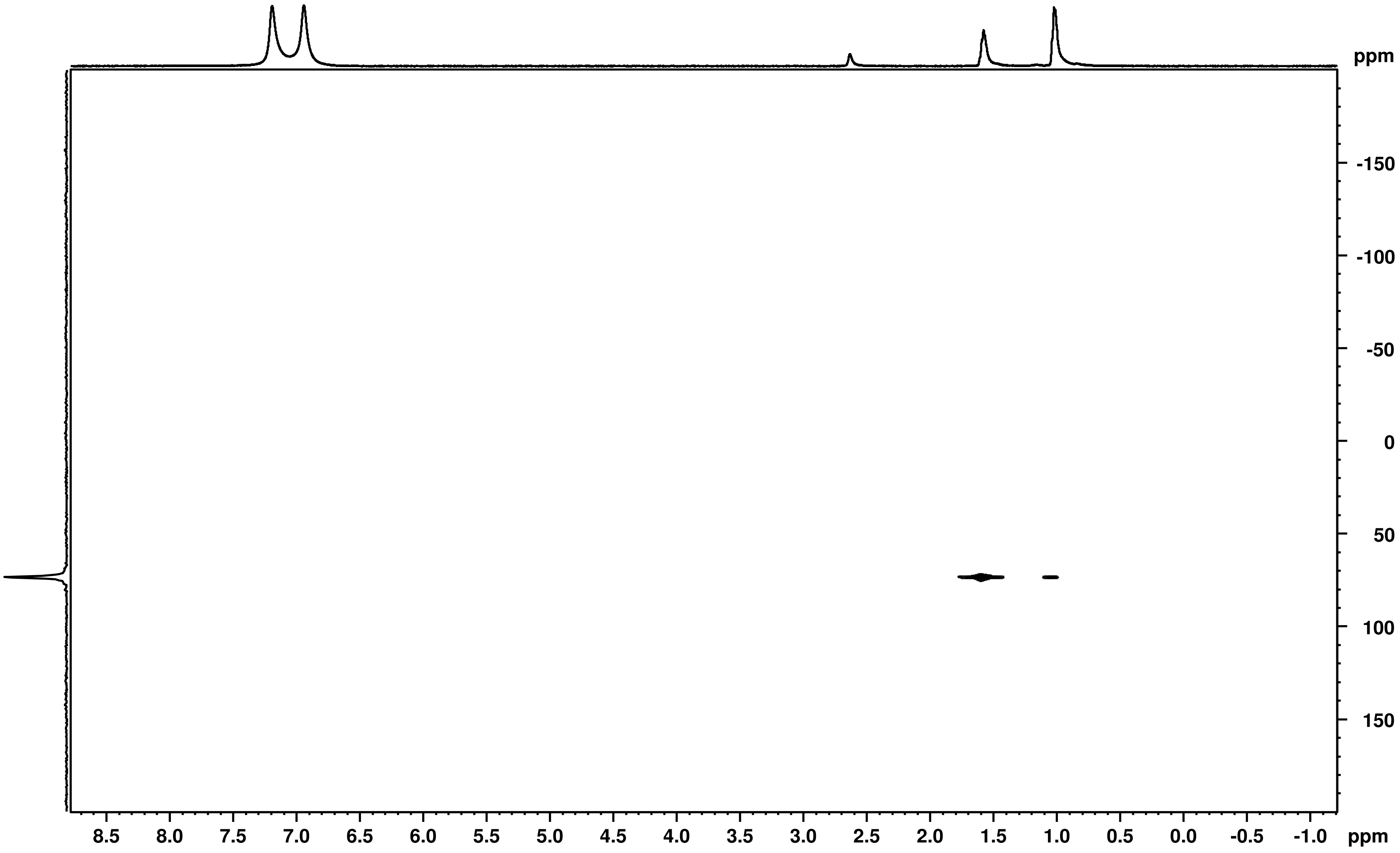

Supplementary Fig. 168. ^{1}**H NMR** spectrum (500 MHz, 1,2-$C_6D_4Cl_2$, 298 K) of [$i$$Pr_2BrSi(HCB_{11}H_5Br_6)$] (**8cc**)

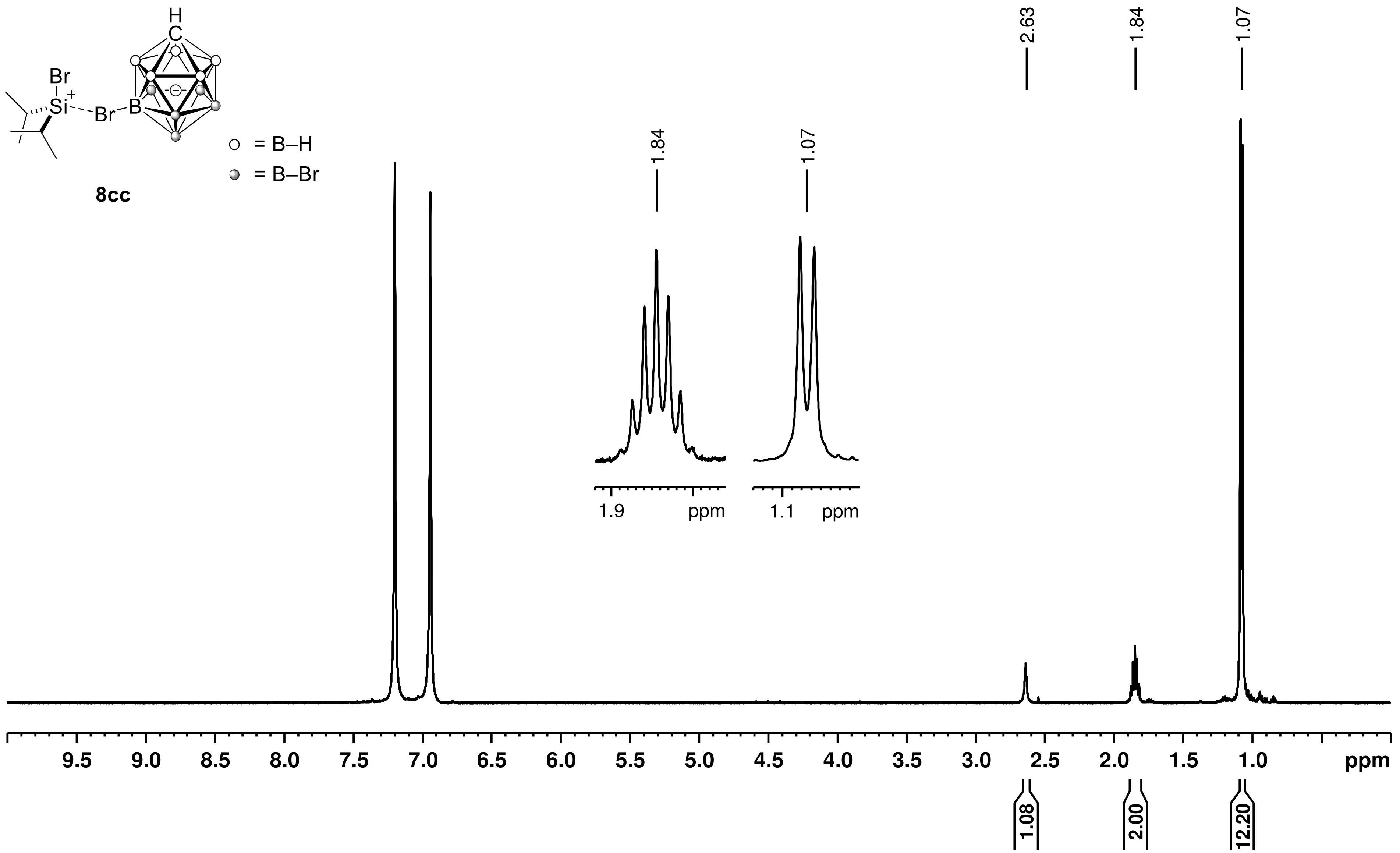

Supplementary Fig. 169. **^{11}B NMR** spectrum (160 MHz, 1,2-$C_6D_4Cl_2$, 298 K) of [*i*$Pr_2BrSi(HCB_{11}H_5Br_6)$] (**8cc**)

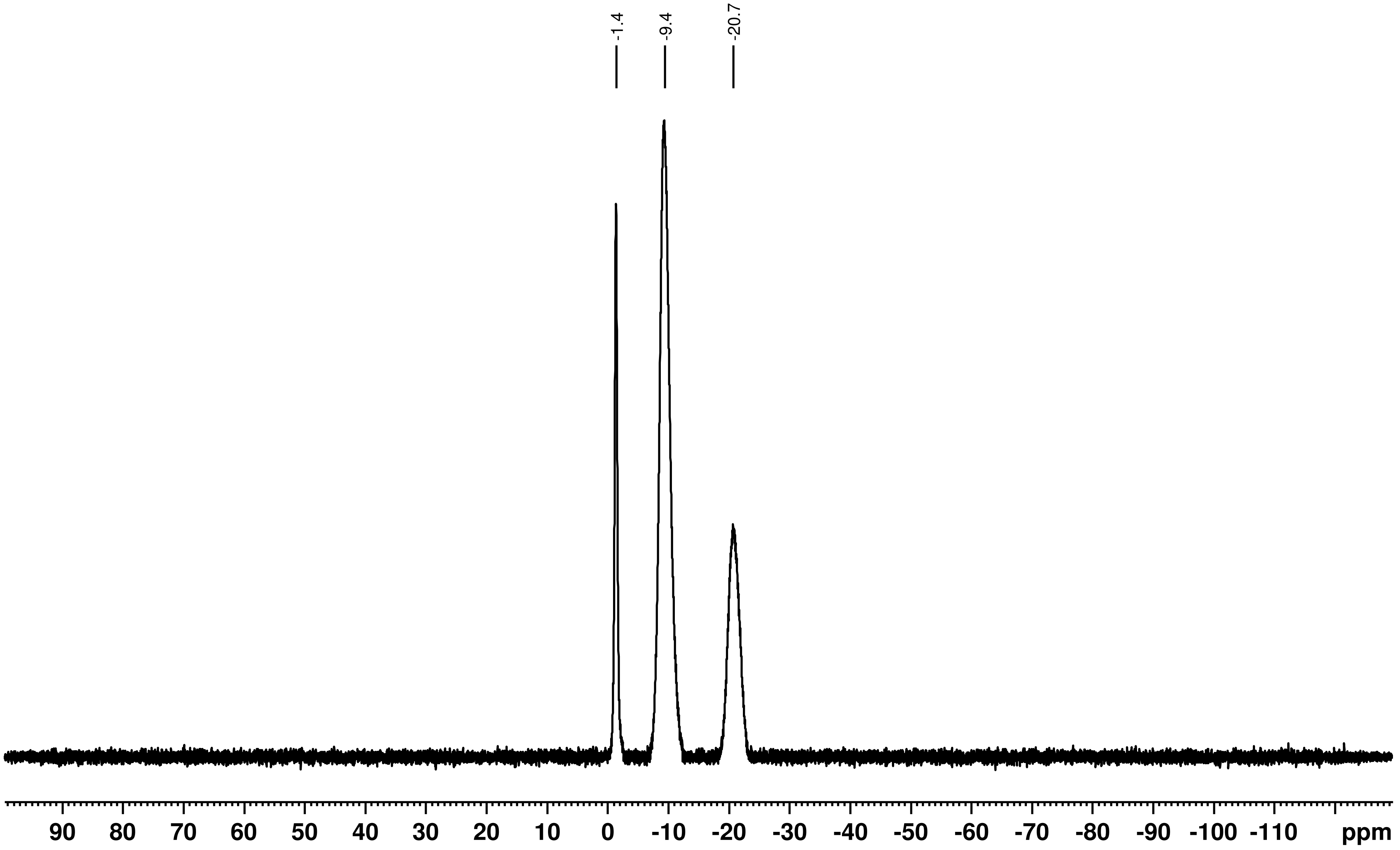

Supplementary Fig. 170. **$^{13}C\{^{1}H\}$ NMR** spectrum (126 MHz, 1,2-$C_6D_4Cl_2$, 298 K) of [*i*$Pr_2BrSi(HCB_{11}H_5Br_6)$] (**8cc**)

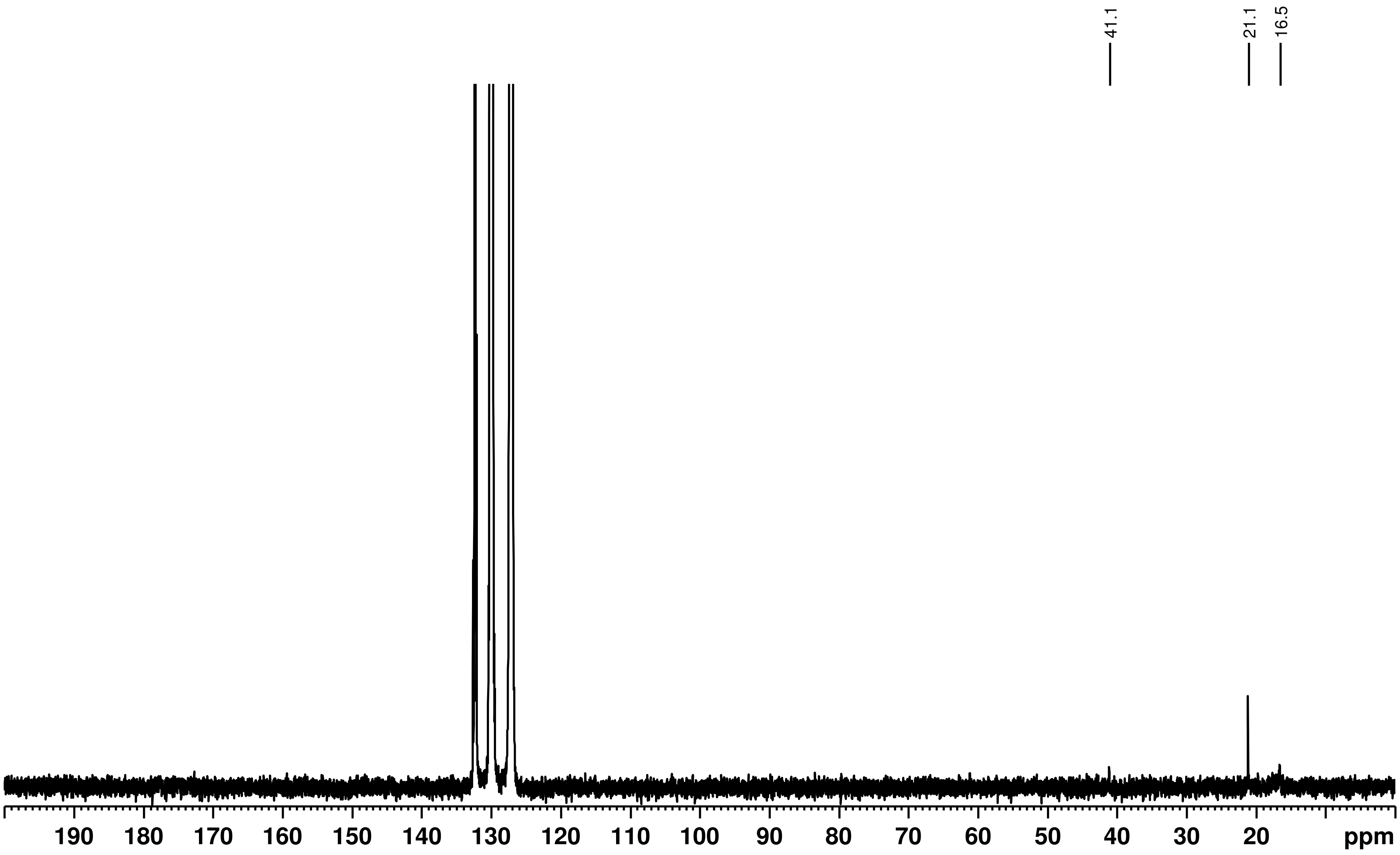

Supplementary Fig. 171. **^{1}H,^{29}Si HMQC NMR** (500/99 MHz, 1,2-$C_6D_4Cl_2$, 298 K, optimized for J = 7 Hz) of [$i$$Pr_2BrSi(HCB_{11}H_5Br_6)$] (**8cc**)

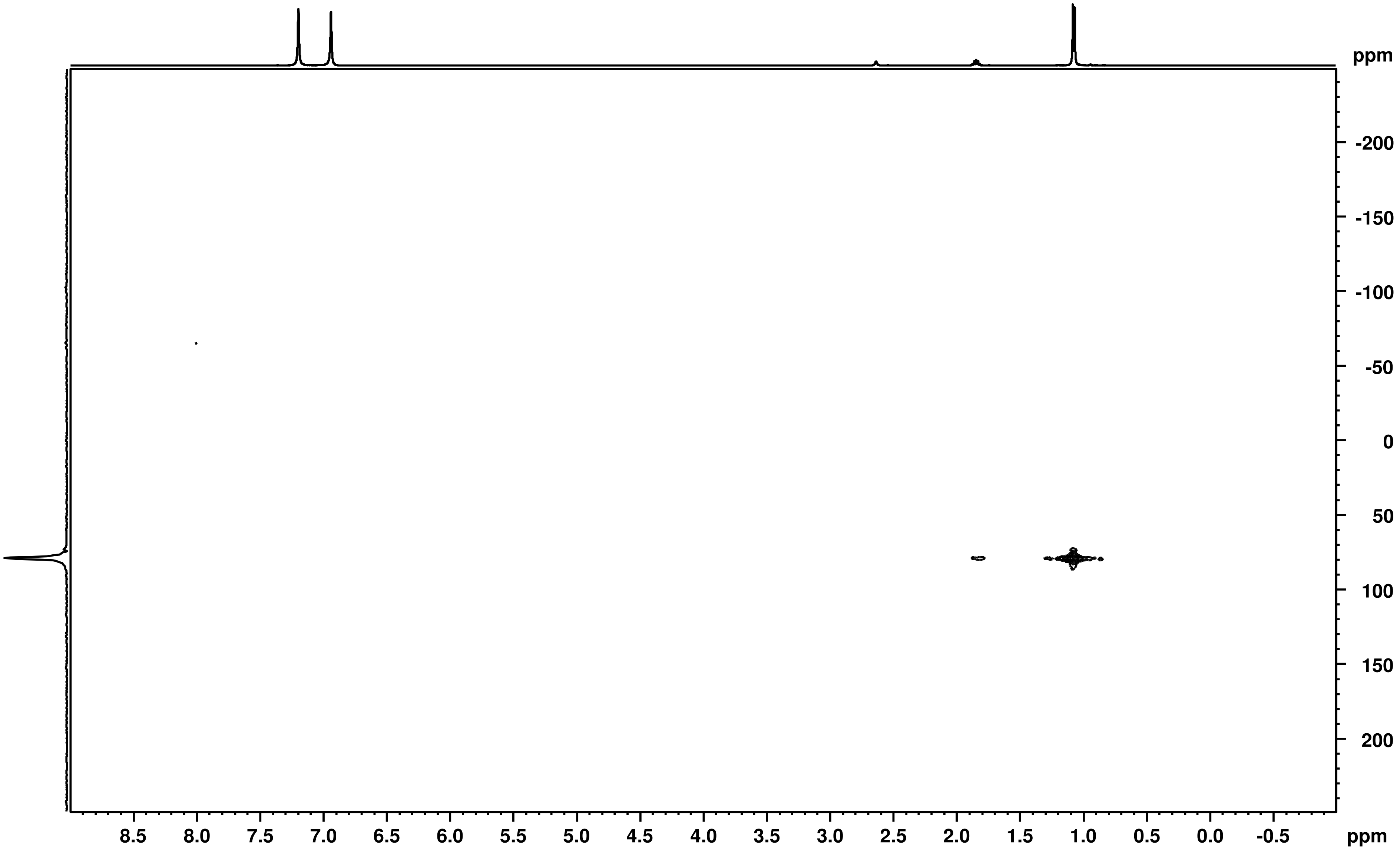

Supplementary Fig. 172. **^{1}H NMR** spectrum (500 MHz, 1,2-$C_6D_4Cl_2$, 298 K) of [$t$$Bu_2BrSi(HCB_{11}H_5Br_6)$] (**8cd**)

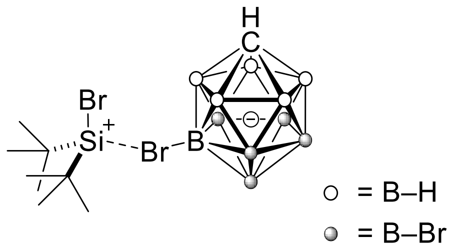

8cd

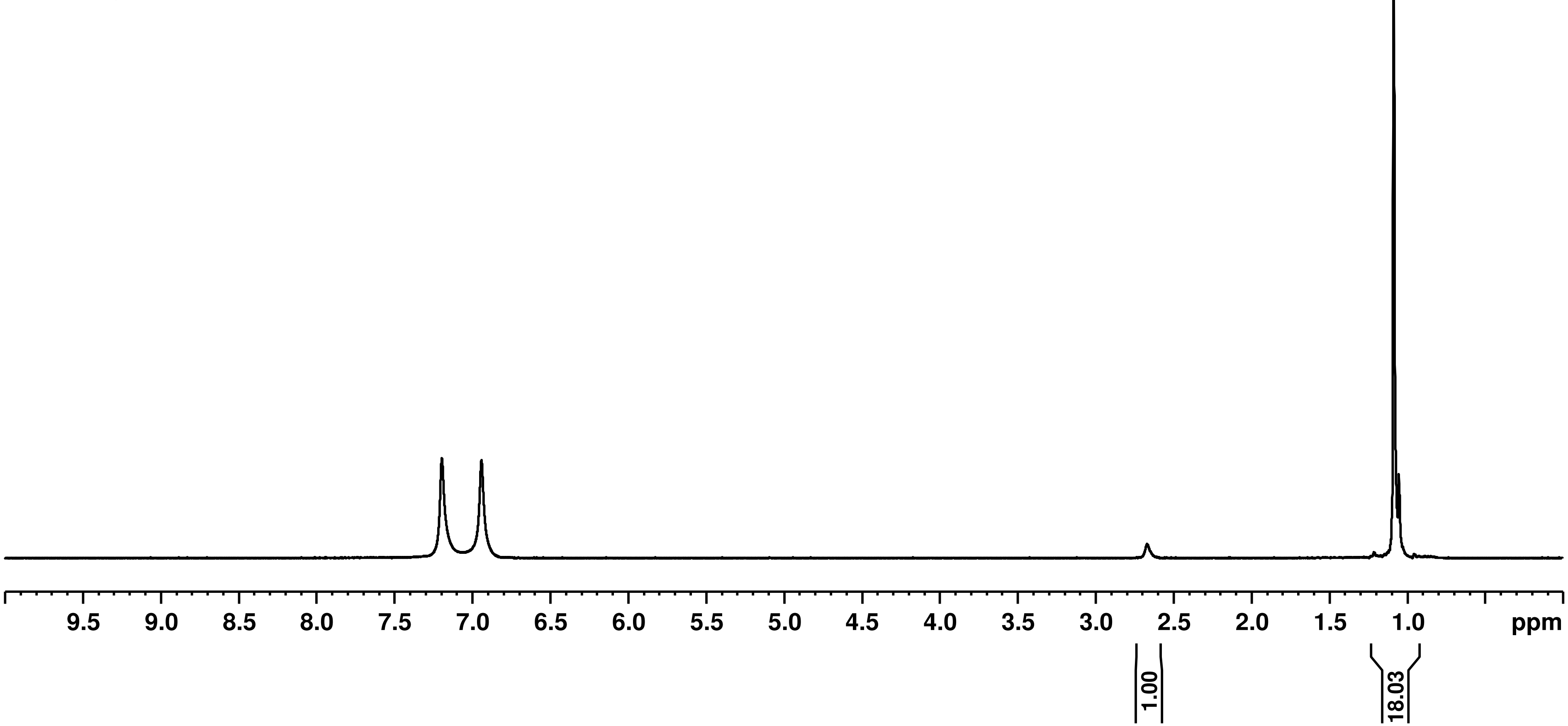

Supplementary Fig. 173. ^{11}**B NMR** spectrum (160 MHz, 1,2-$C_6D_4Cl_2$, 298 K) of [$t$$Bu_2BrSi(HCB_{11}H_5Br_6)$] (**8cd**) (* anion decomposition)

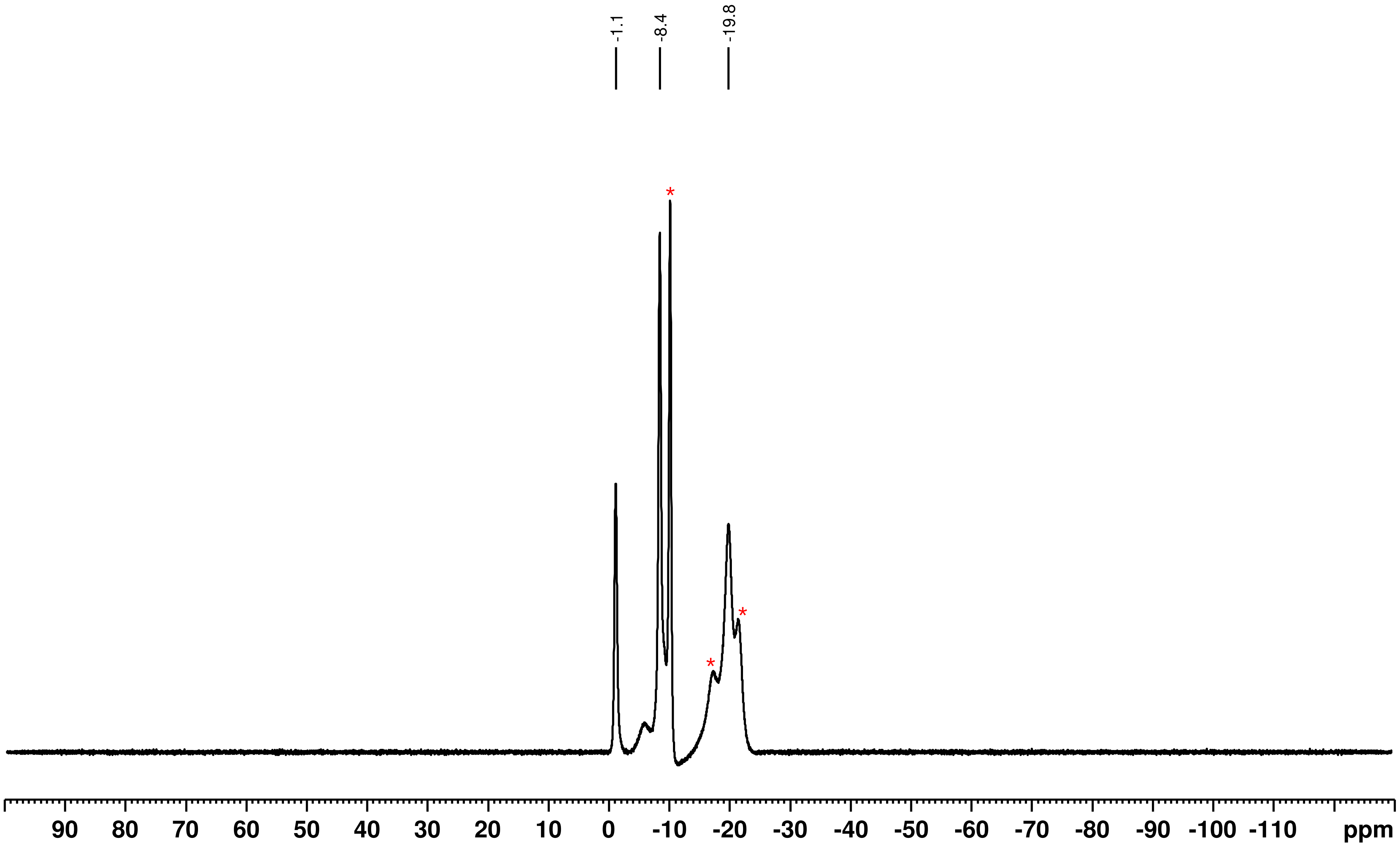

Supplementary Fig. 174. **$^{13}C\{^{1}H\}$ NMR** spectrum (126 MHz, 1,2-$C_6D_4Cl_2$, 298 K) of [$t$$Bu_2BrSi(HCB_{11}H_5Br_6)$] (**8cd**)

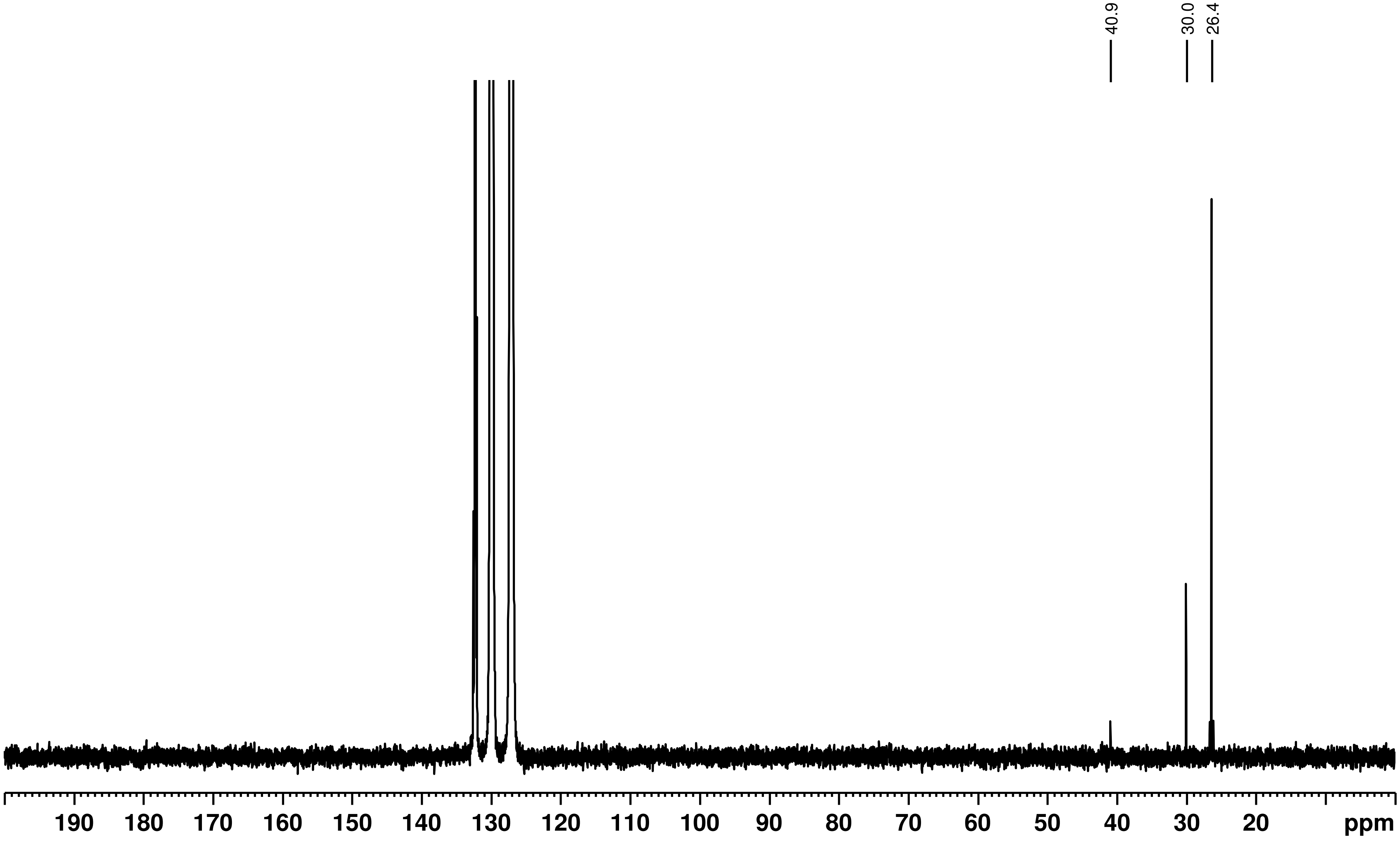

Supplementary Fig. 175. **^{29}Si{^{1}H} DEPT NMR** spectrum (99 MHz, 1,2-$C_6D_4Cl_2$, 298 K, optimized for $J_{H,Si}$ = 7 Hz, 13.6°) of [*t*$Bu_2BrSi(HCB_{11}H_5Br_6)$] (**8cd**)

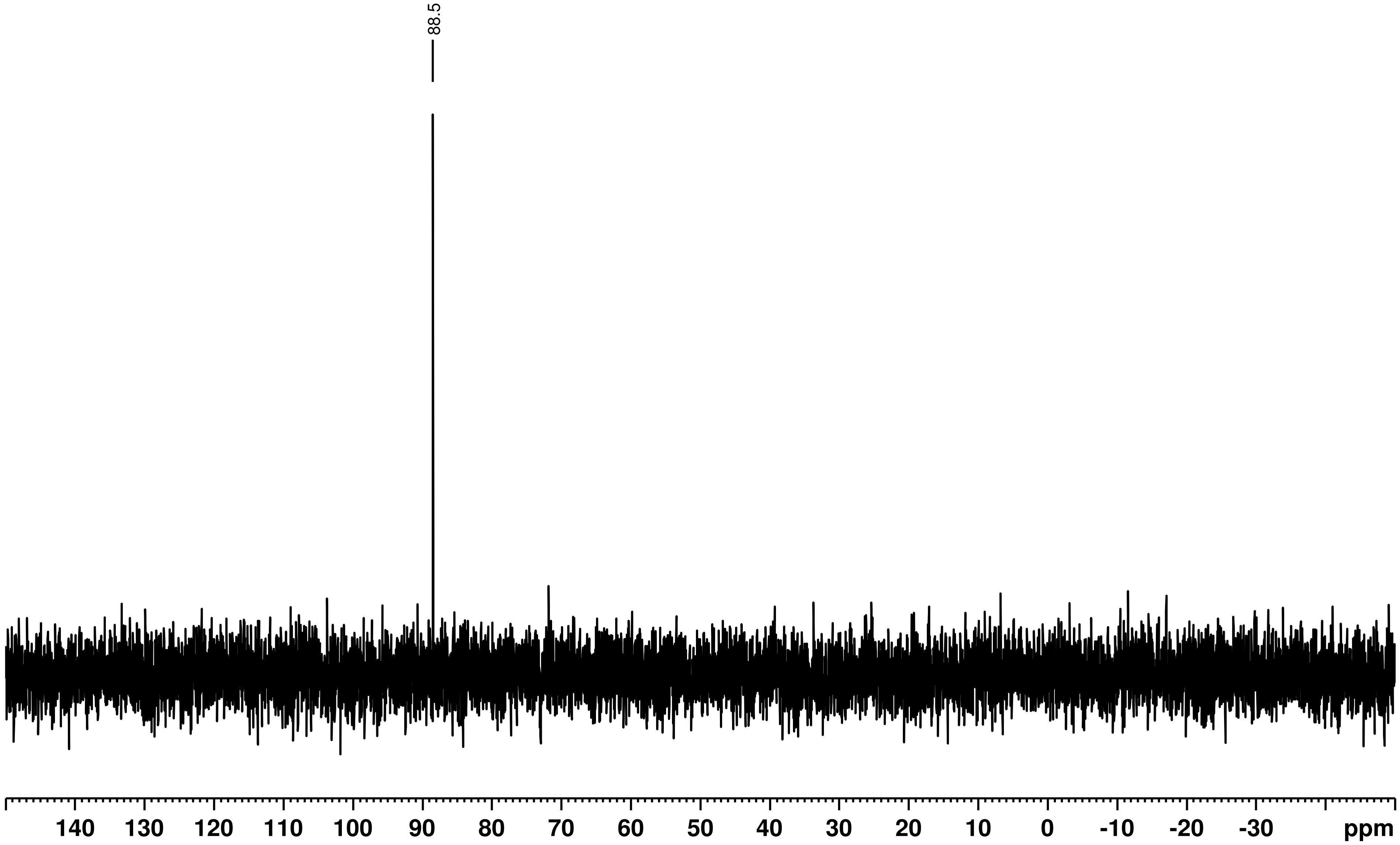

Supplementary Fig. 176. **^{1}H NMR** spectrum (500 MHz, 1,2-$C_6D_4Cl_2$, 298 K) of [$Me_2ISi(HCB_{11}H_5Br_6)$] (**8da**)

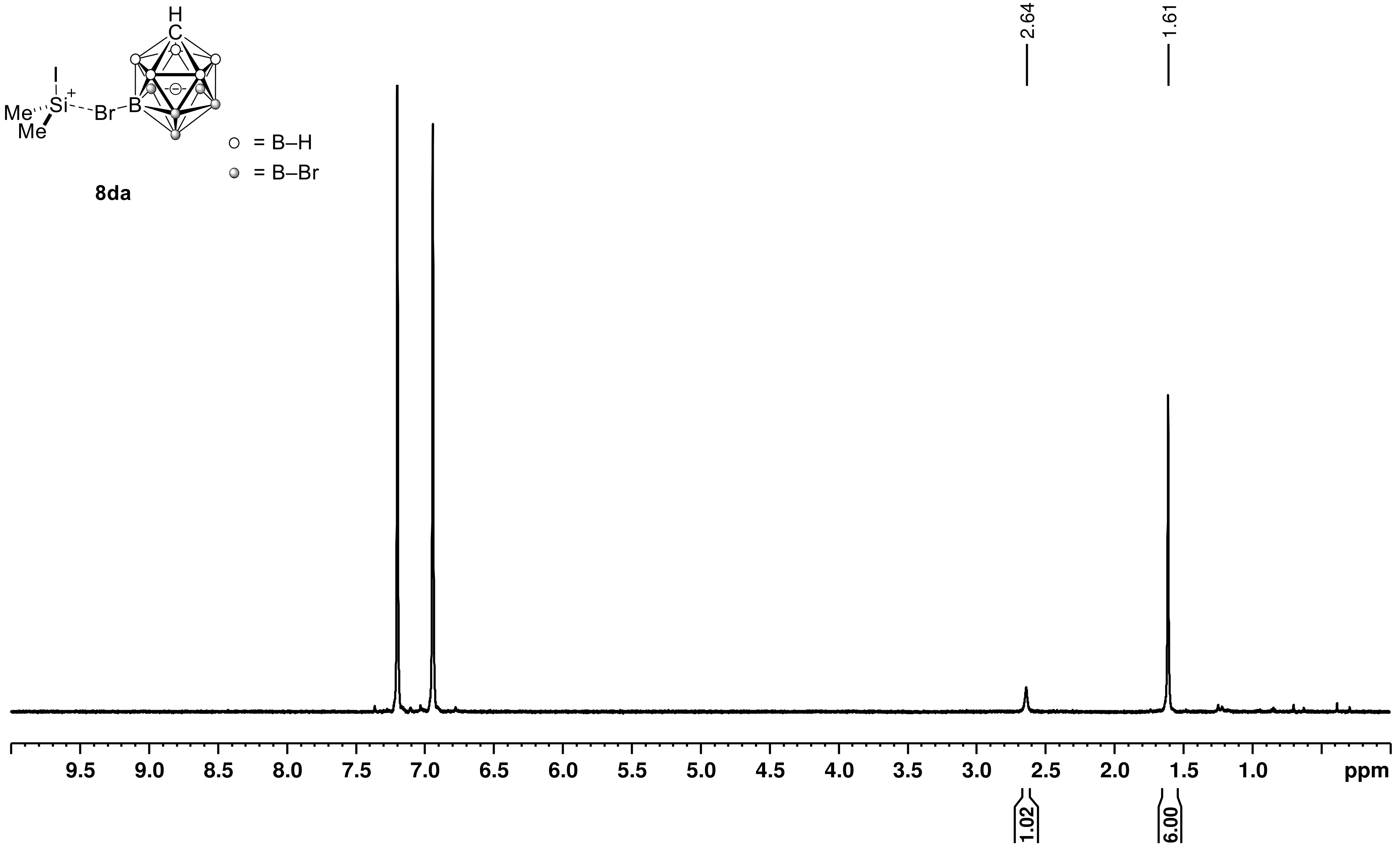

Supplementary Fig. 177. ^{11}**B NMR** spectrum (160 MHz, 1,2-$C_6D_4Cl_2$, 298 K) of [$Me_2ISi(HCB_{11}H_5Br_6)$] (**8da**)

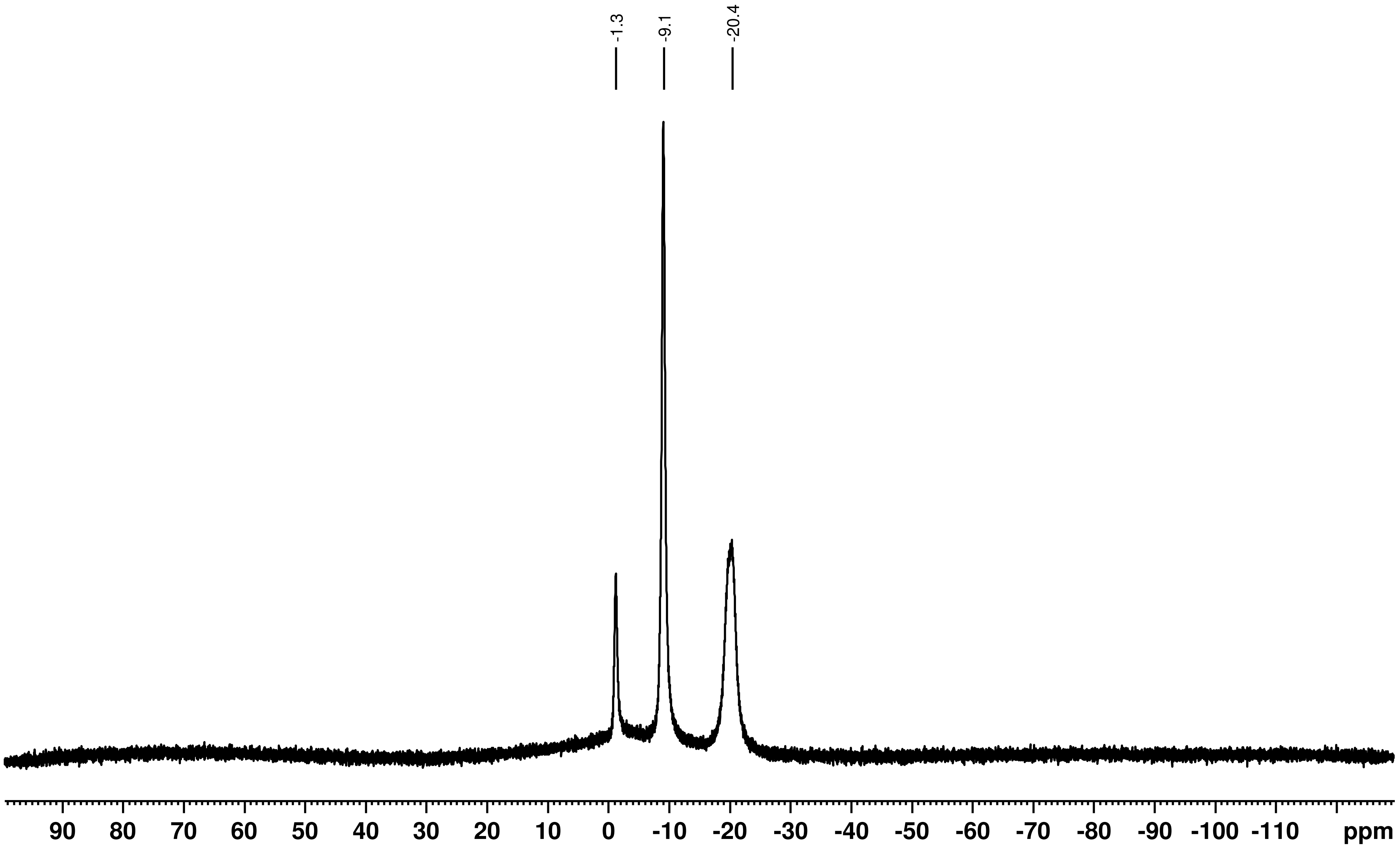

Supplementary Fig. 178. **$^{13}C\{^{1}H\}$ NMR** spectrum (126 MHz, 1,2-$C_6D_4Cl_2$, 298 K) of [$Me_2ISi(HCB_{11}H_5Br_6)$] (**8da**)

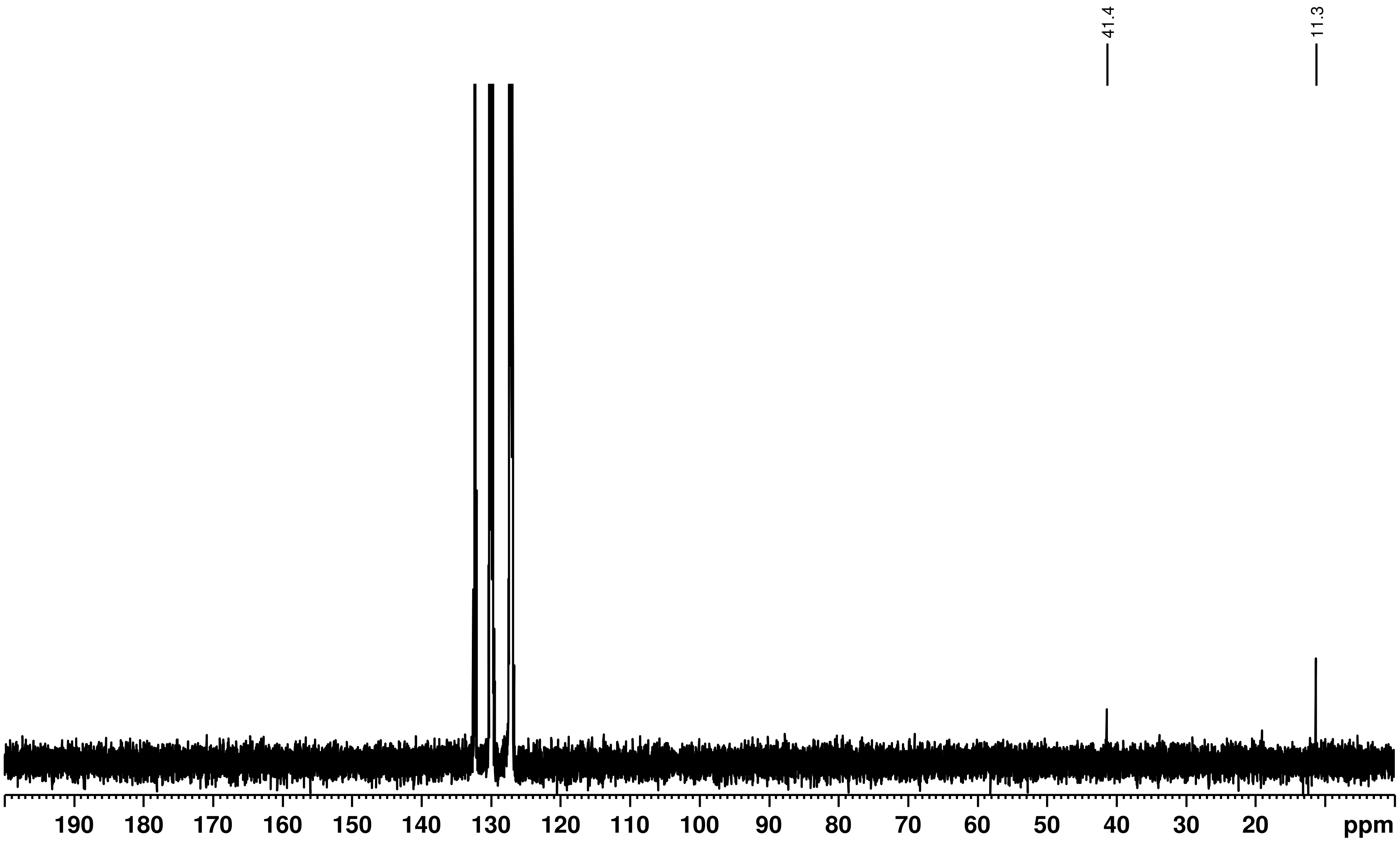

Supplementary Fig. 179. **^{1}H,^{29}Si HMQC NMR** (500/99 MHz, 1,2-$C_6D_4Cl_2$, 298 K, optimized for *J* = 7 Hz) of [$Me_2ISi(HCB_{11}H_5Br_6)$] (**8da**)

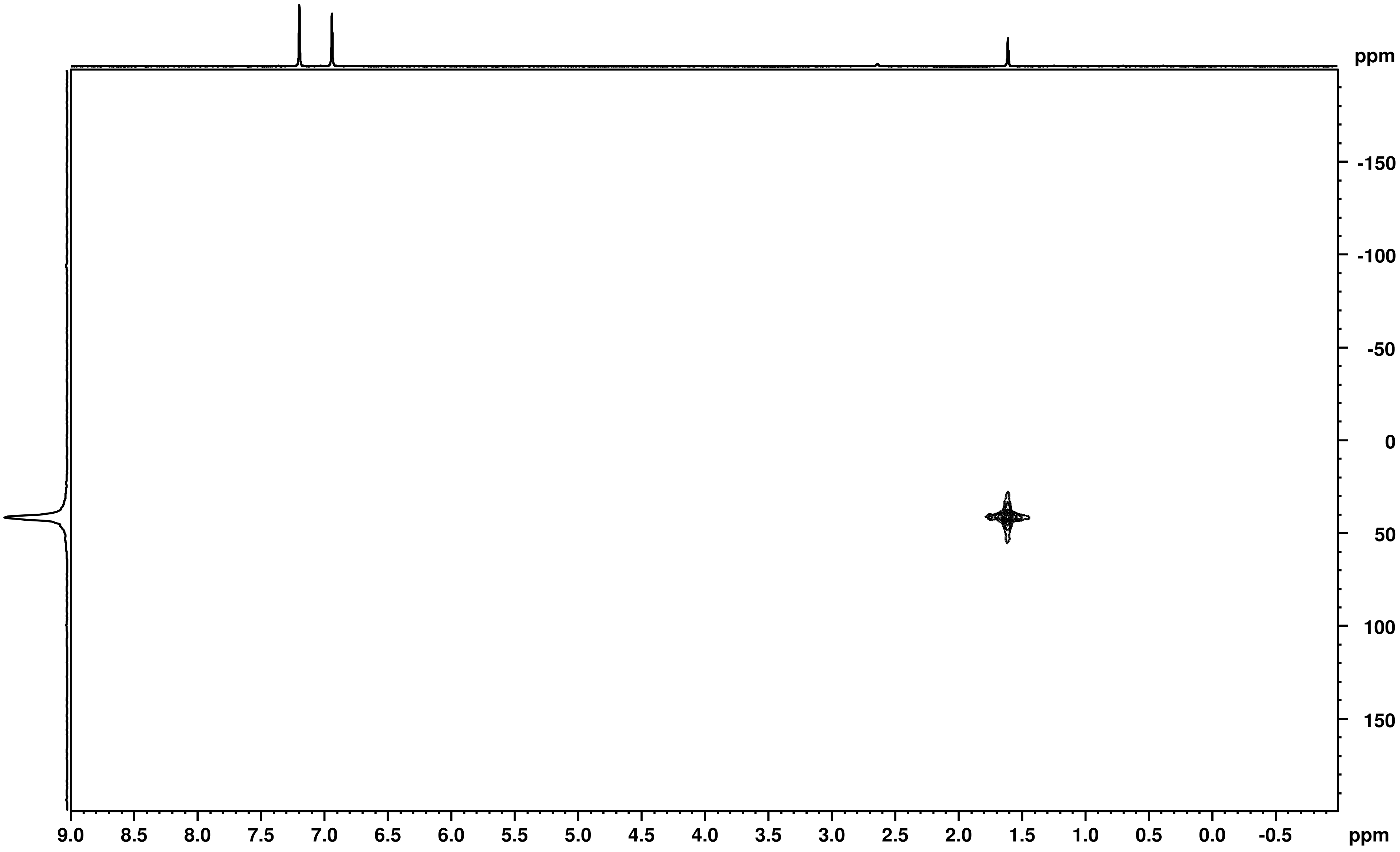

Supplementary Fig. 180. **^{1}H NMR** spectrum (500 MHz, 1,2-$C_6D_4Cl_2$, 298 K) of [$Et_2ISi(HCB_{11}H_5Br_6)$] (**8db**)

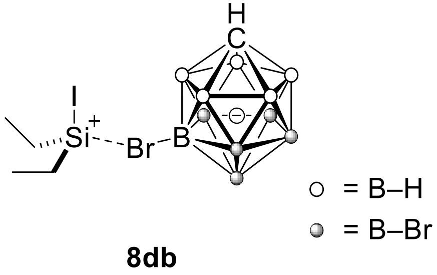

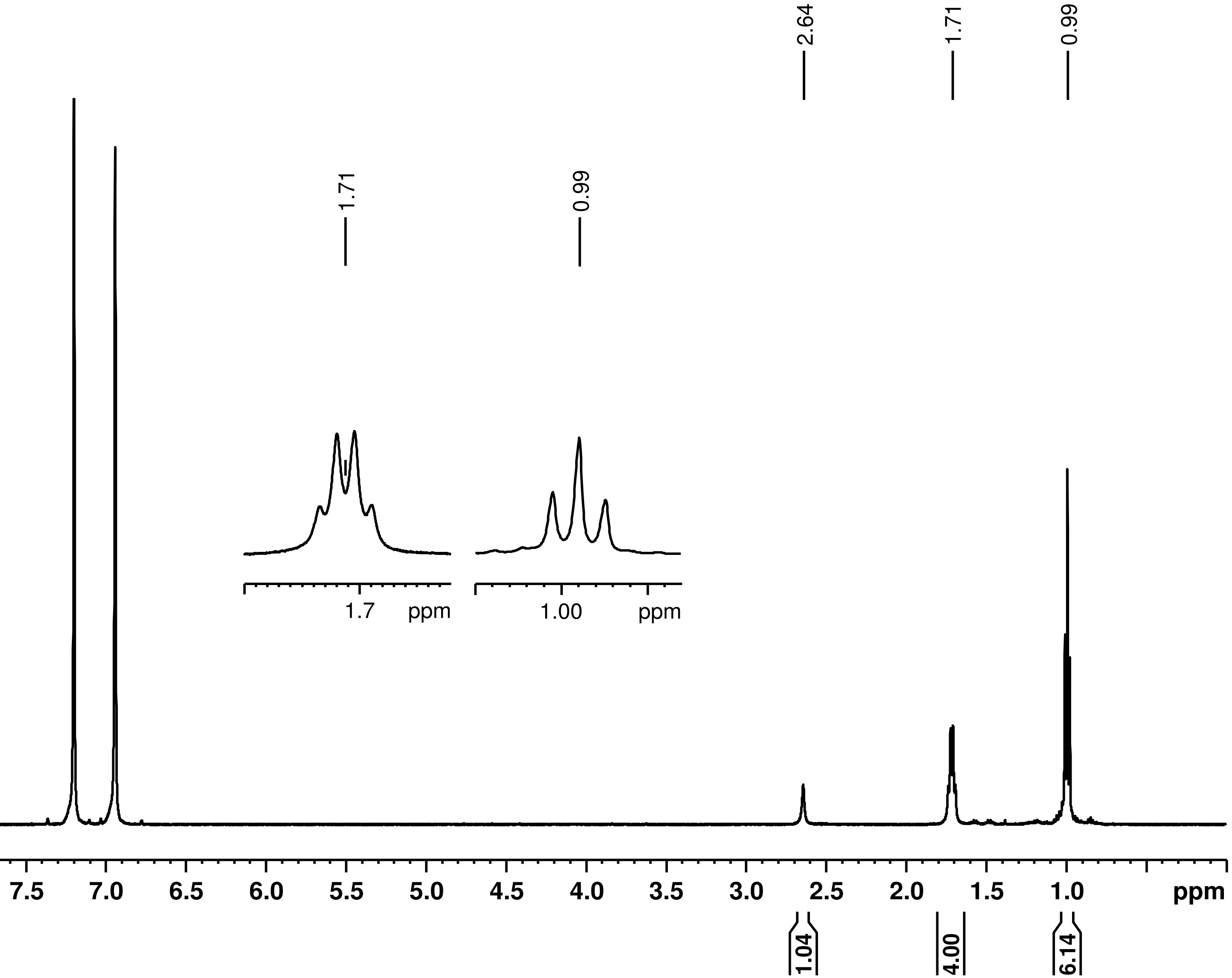

Supplementary Fig. 181. ^{11}**B NMR** spectrum (160 MHz, 1,2-$C_6D_4Cl_2$, 298 K) of [$Et_2ISi(HCB_{11}H_5Br_6)$] (**8db**)

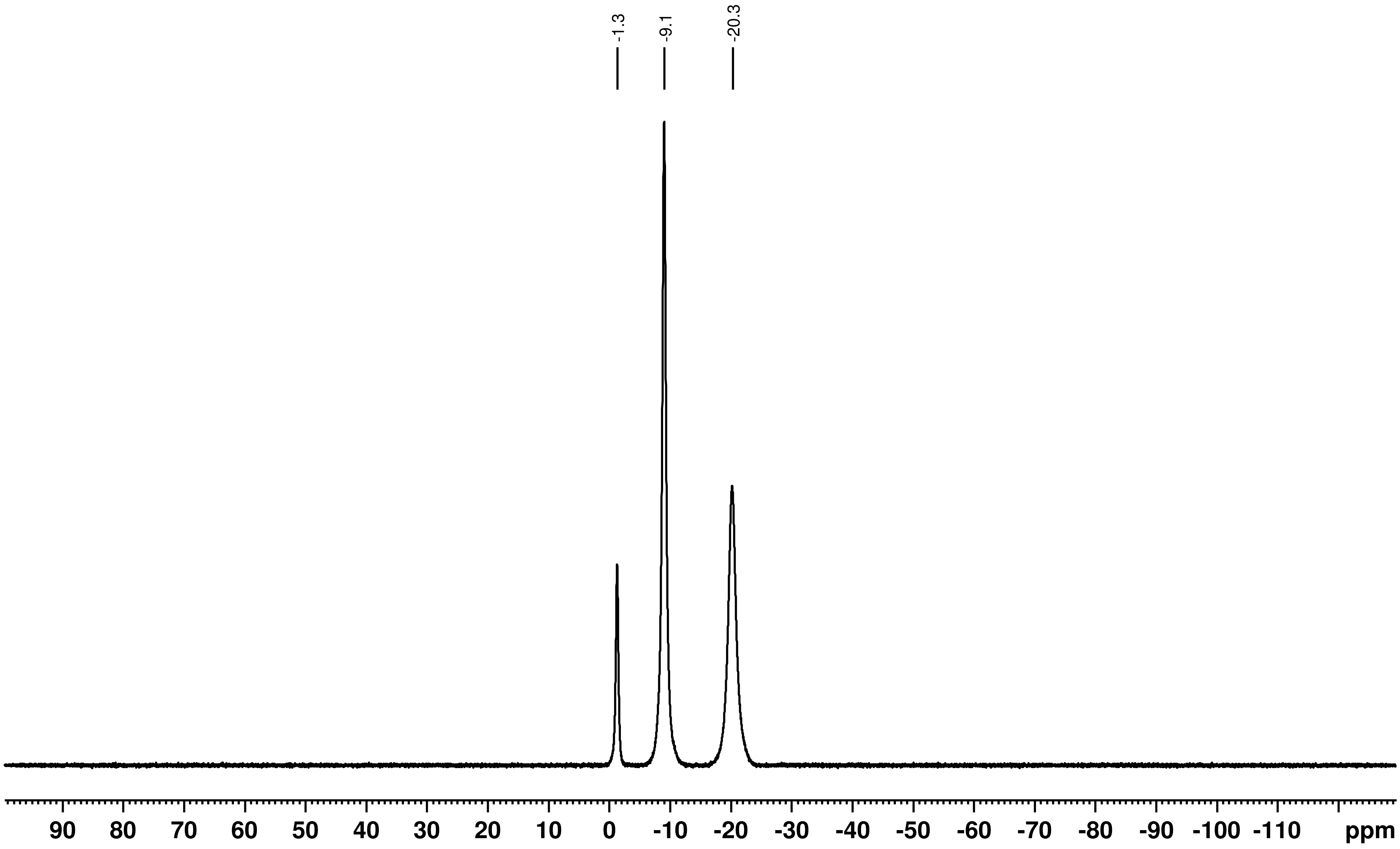

Supplementary Fig. 182. **$^{13}C\{^{1}H\}$ NMR** spectrum (126 MHz, 1,2-$C_6D_4Cl_2$, 298 K) of [$Et_2ISi(HCB_{11}H_5Br_6)$] (**8db**) (* [$Et_2ClSi(HCB_{11}H_5Br_6)$])

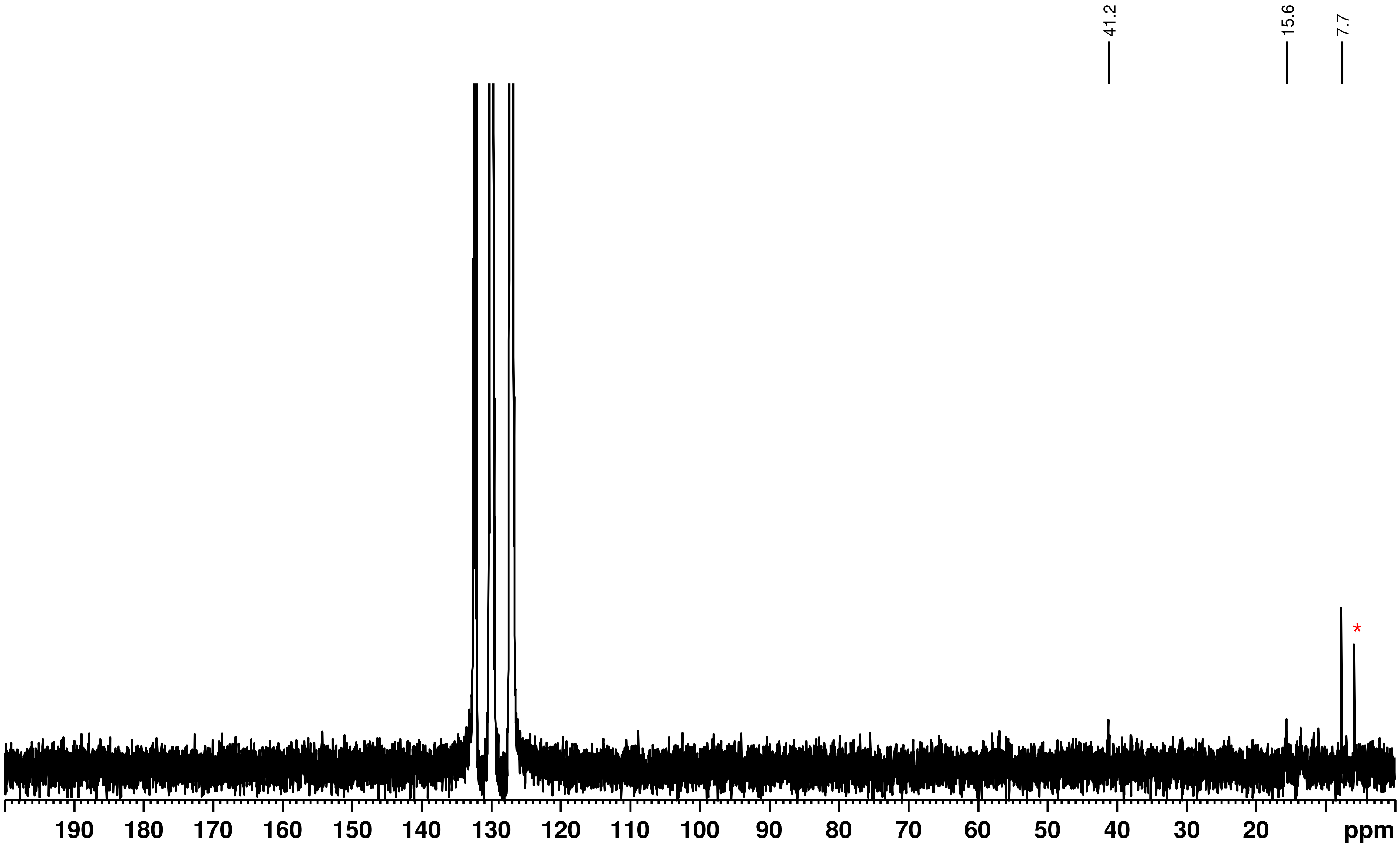

Supplementary Fig. 183. **^{1}H,^{29}Si HMQC NMR** (500/99 MHz, 1,2-$C_6D_4Cl_2$, 298 K, optimized for *J* = 7 Hz) of [$Et_2ISi(HCB_{11}H_5Br_6)$] (**8db**)

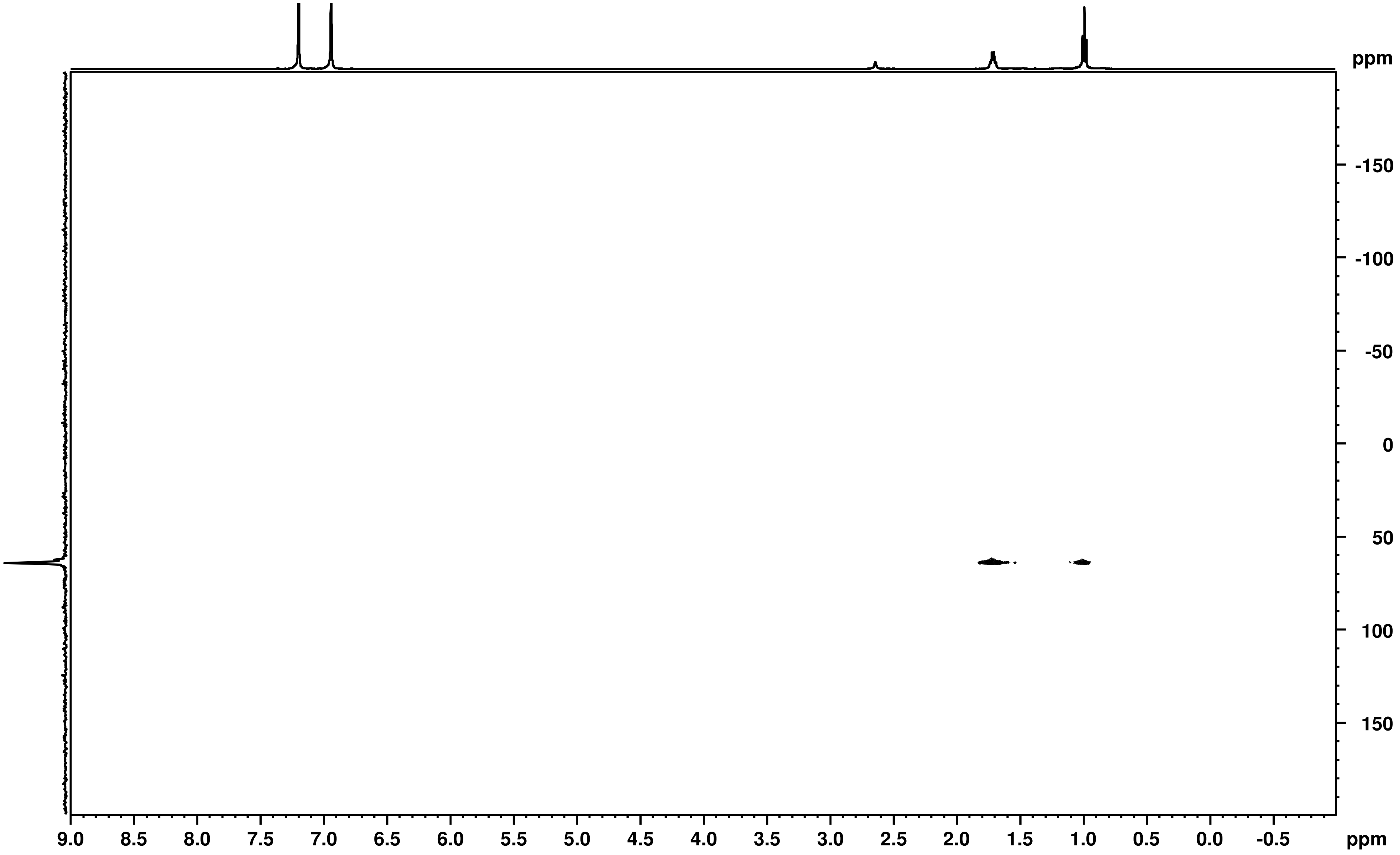

Supplementary Fig. 184. **^{1}H NMR** spectrum (500 MHz, 1,2-$C_6D_4Cl_2$, 298 K) of [*i*Pr_2ISi($HCB_{11}H_5Br_6$)] (**8dc**)

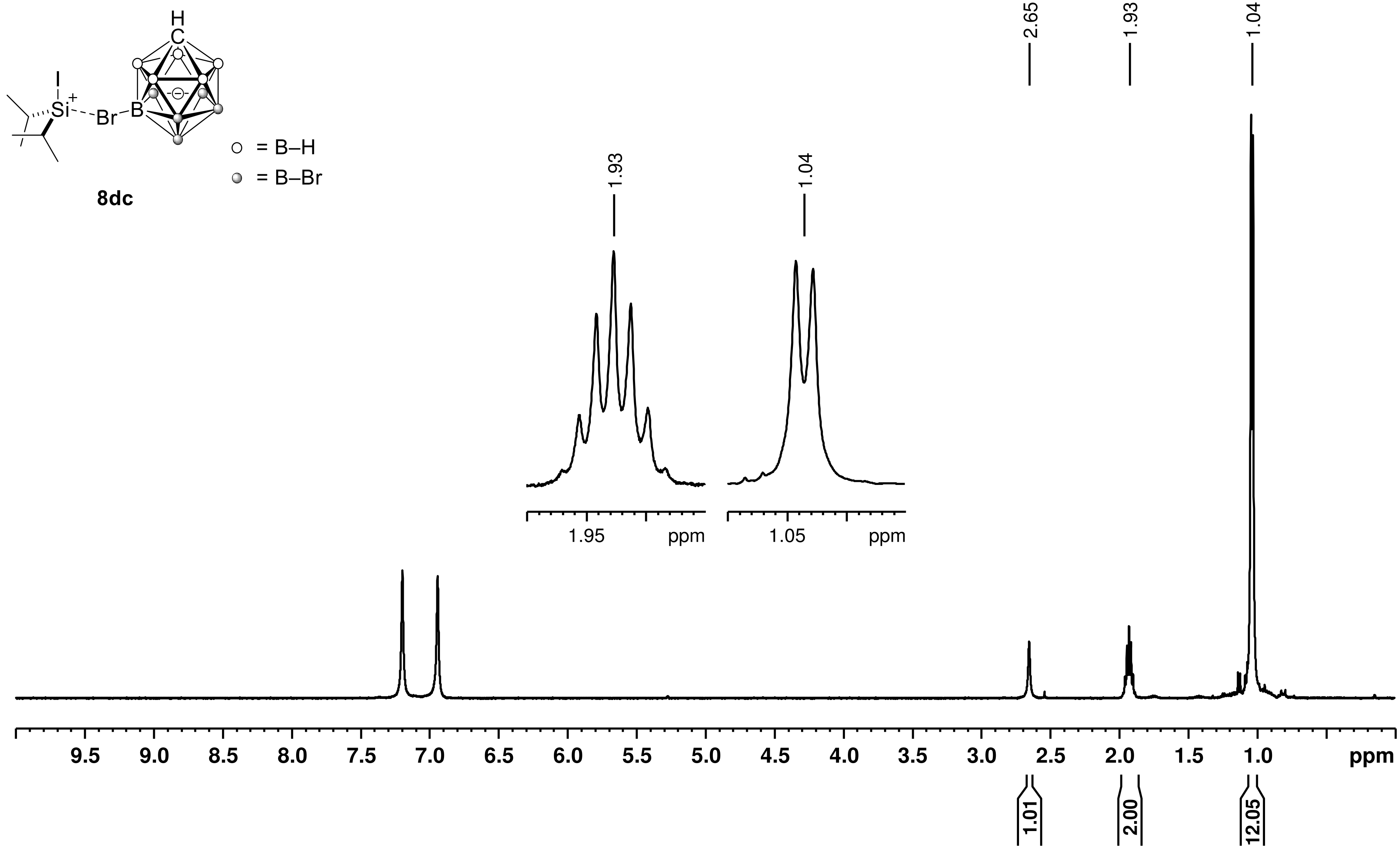

Supplementary Fig. 185. ^{11}**B NMR** spectrum (160 MHz, 1,2-$C_6D_4Cl_2$, 298 K) of [*i*Pr_2ISi($HCB_{11}H_5Br_6$)] (**8dc**)

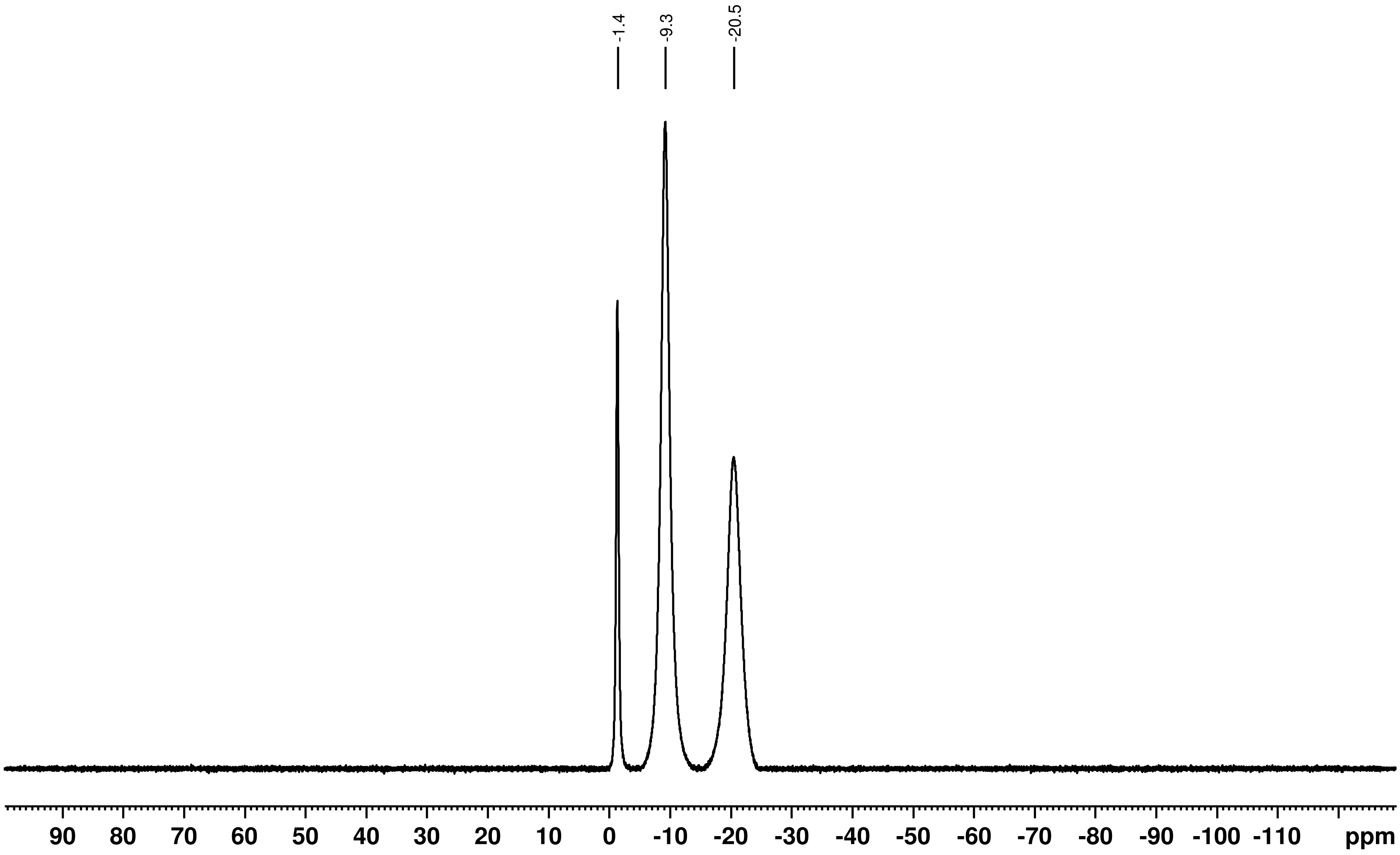

Supplementary Fig. 186. **$^{13}C\{^{1}H\}$ NMR** spectrum (126 MHz, 1,2-$C_6D_4Cl_2$, 298 K) of [*i*$Pr_2ISi(HCB_{11}H_5Br_6)$] (**8dc**)

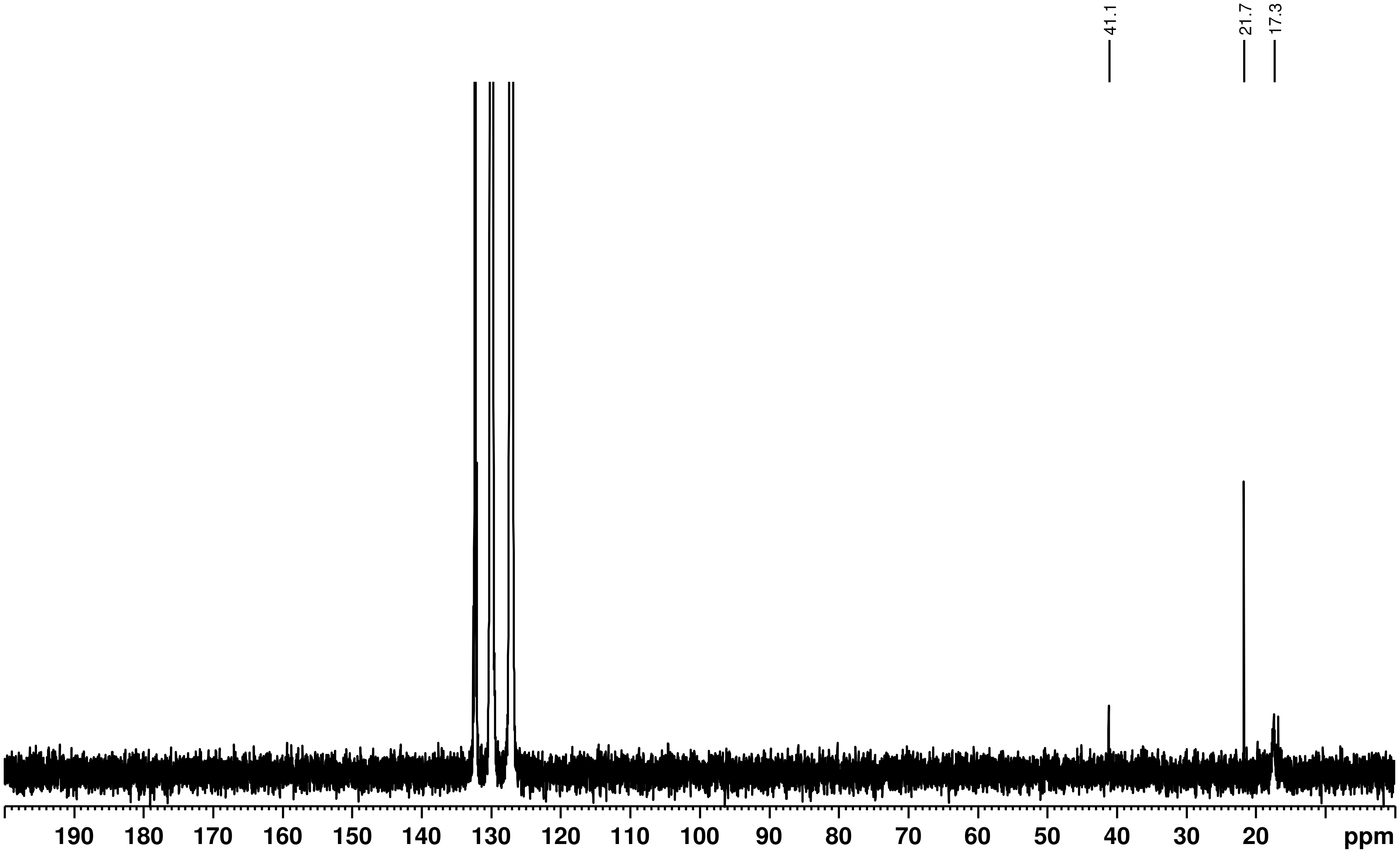

Supplementary Fig. 187. **^{1}H,^{29}Si HMQC NMR** (500/99 MHz, 1,2-$C_6D_4Cl_2$, 298 K, optimized for *J* = 7 Hz) of [*i*$Pr_2ISi(HCB_{11}H_5Br_6)$] (**8dc**)

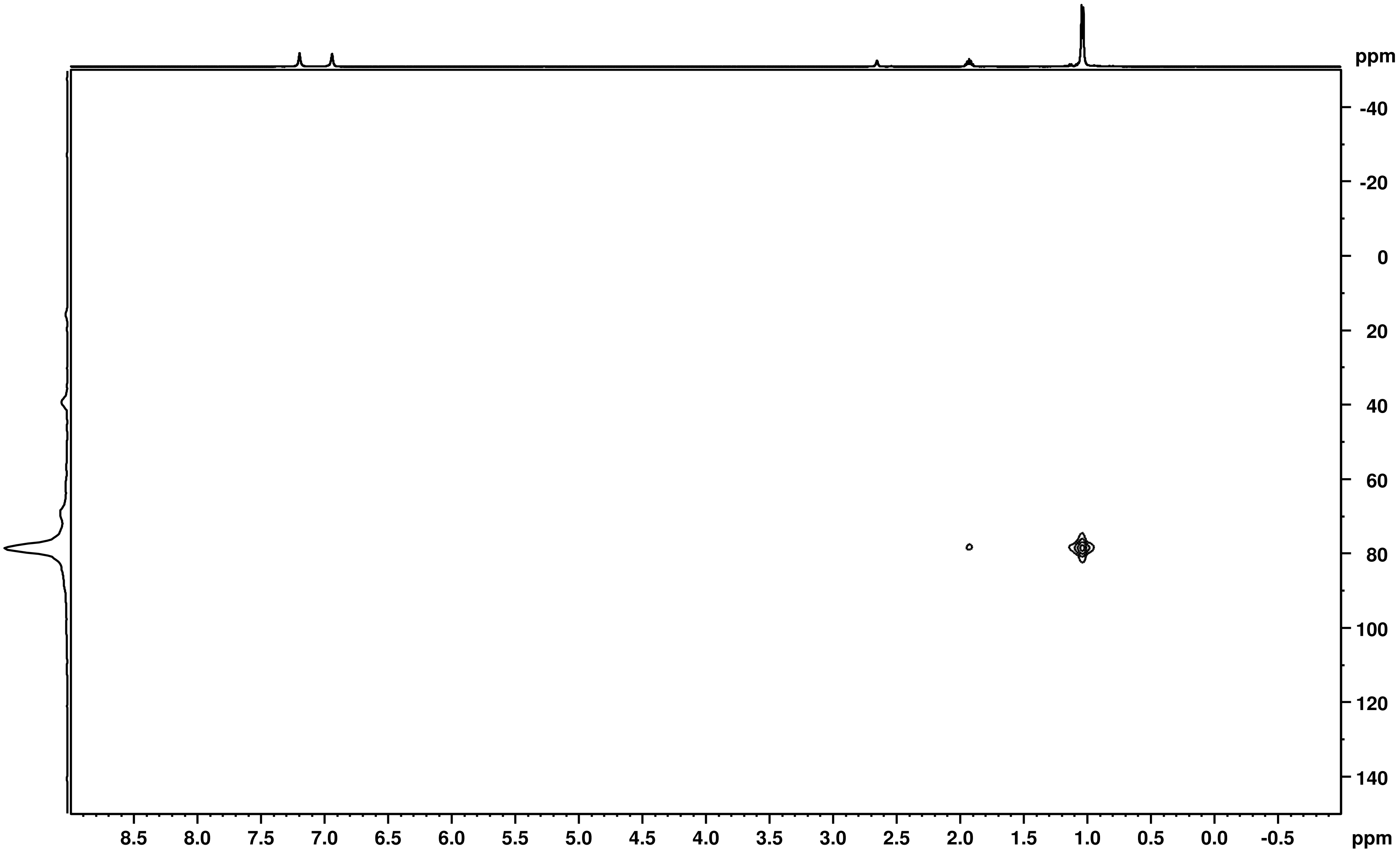

Supplementary Fig. 188. **^{1}H NMR** spectrum (500 MHz, 1,2-$C_6D_4Cl_2$, 298 K) of [*t*$Bu_2ISi(HCB_{11}H_5Br_6)$] (**8dd**)

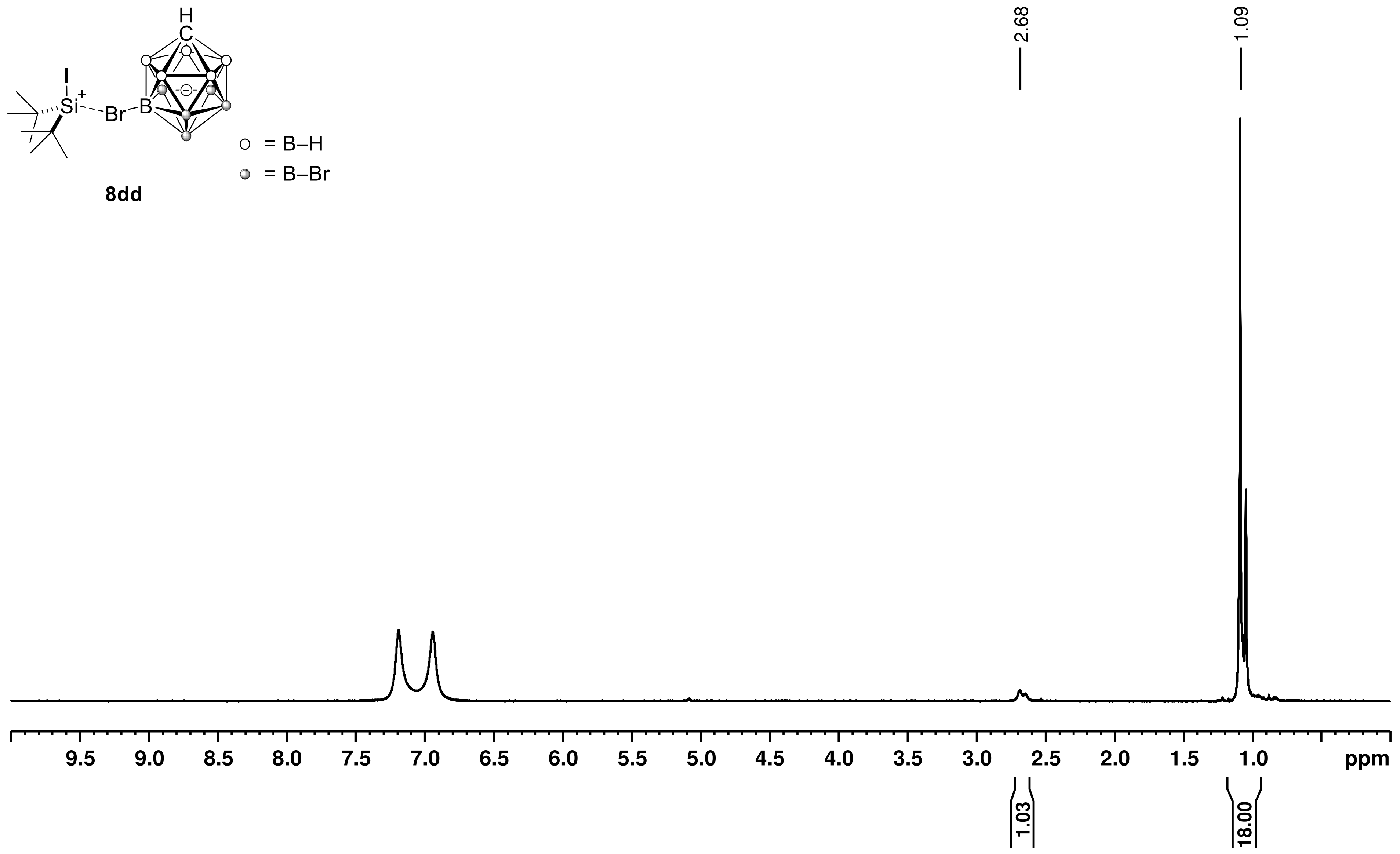

Supplementary Fig. 189. **^{11}B NMR** spectrum (160 MHz, 1,2-$C_6D_4Cl_2$, 298 K) of [*t*Bu_2ISi($HCB_{11}H_5Br_6$)] (**8dd**) (* anion decomposition)

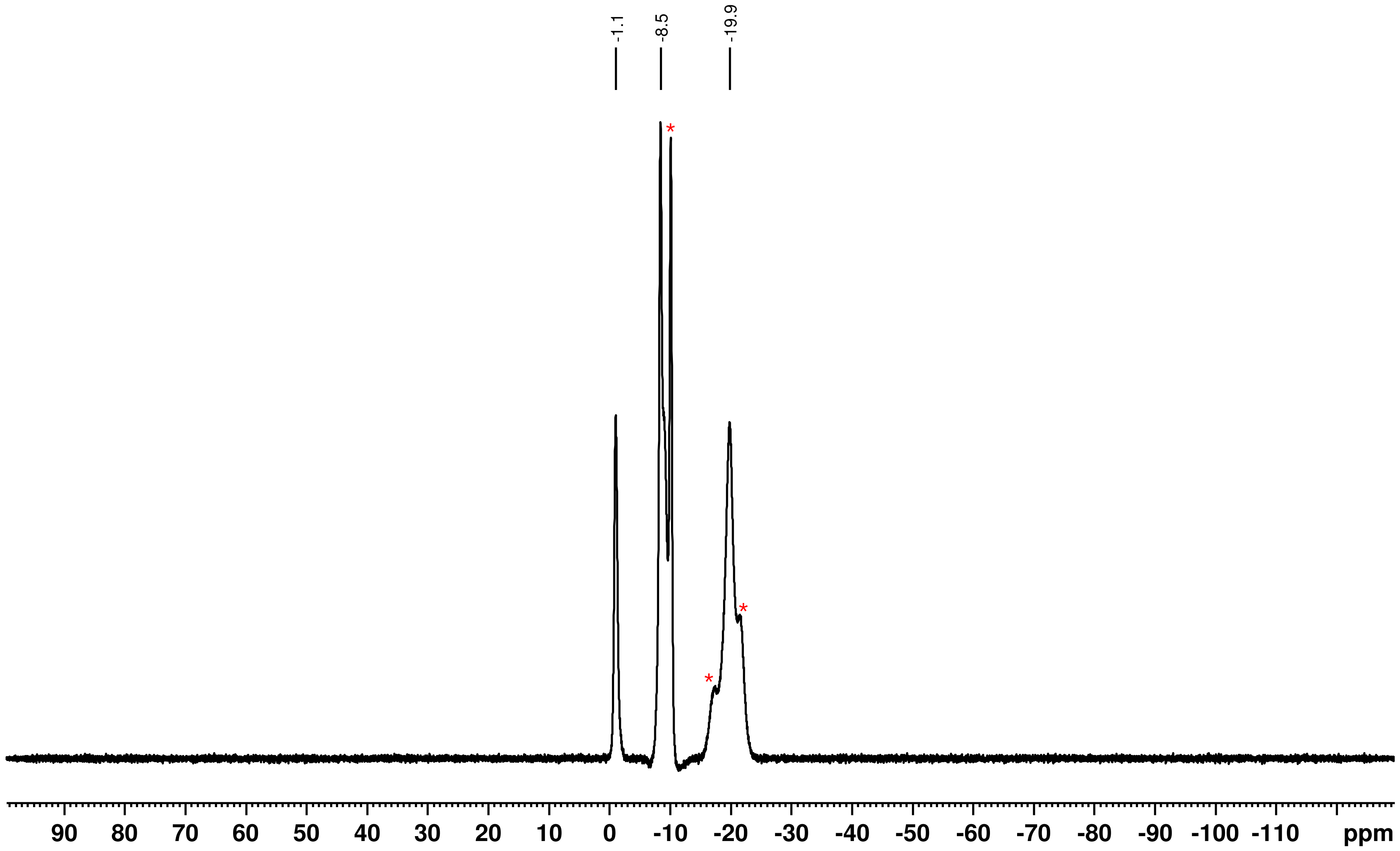

Supplementary Fig. 190. **$^{13}C\{^1H\}$ NMR** spectrum (126 MHz, 1,2-$C_6D_4Cl_2$, 298 K) of [*t*$Bu_2ISi(HCB_{11}H_5Br_6)$] (**8dd**)

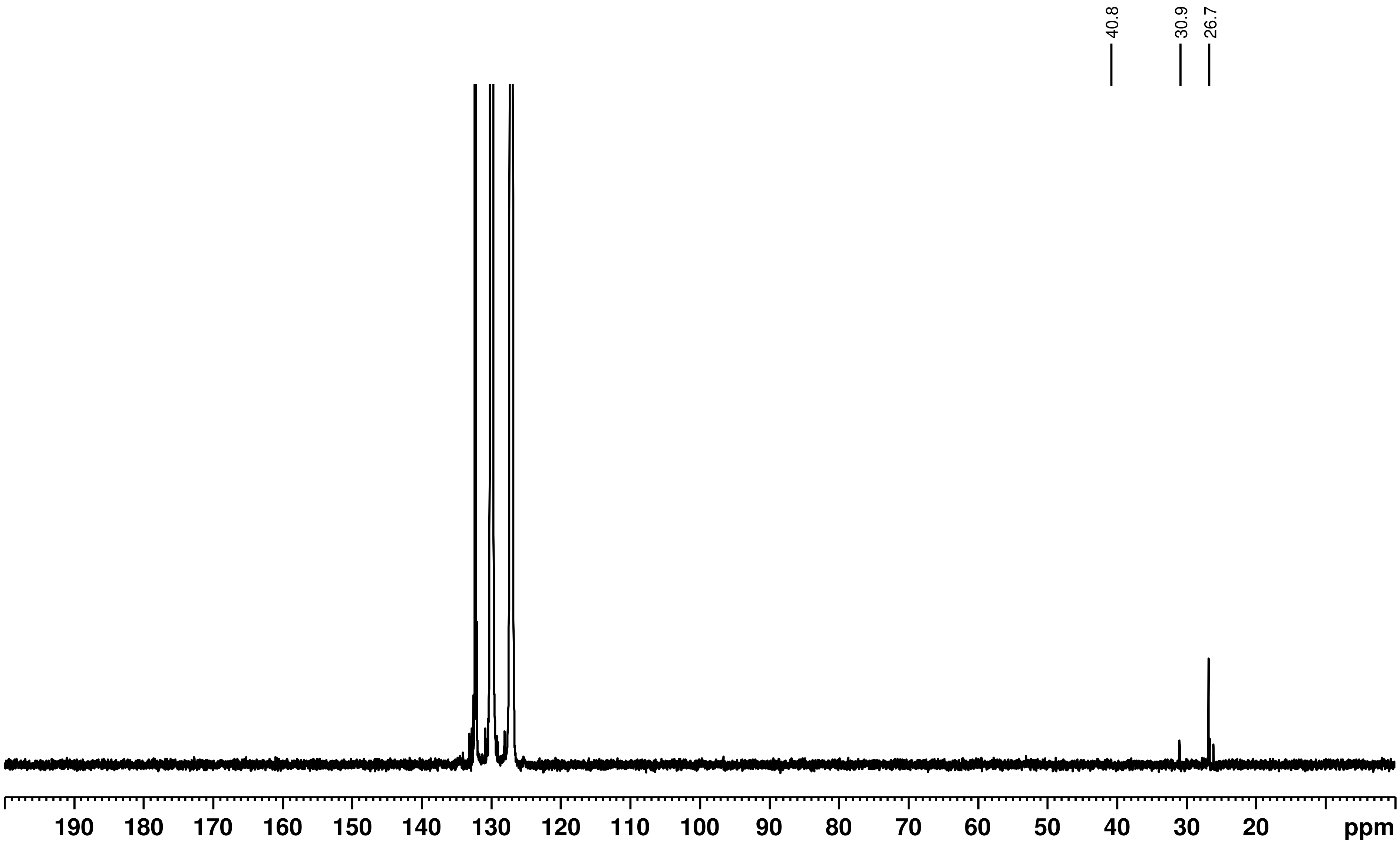

Supplementary Fig. 191. **$^{29}Si\{^{1}H\}$ DEPT NMR** spectrum (99 MHz, 1,2-$C_6D_4Cl_2$, 298 K, optimized for $J_{H,Si}$ = 7 Hz, 13.6°) of [*t*$Bu_2ISi(HCB_{11}H_5Br_6)$] (**8dd**)

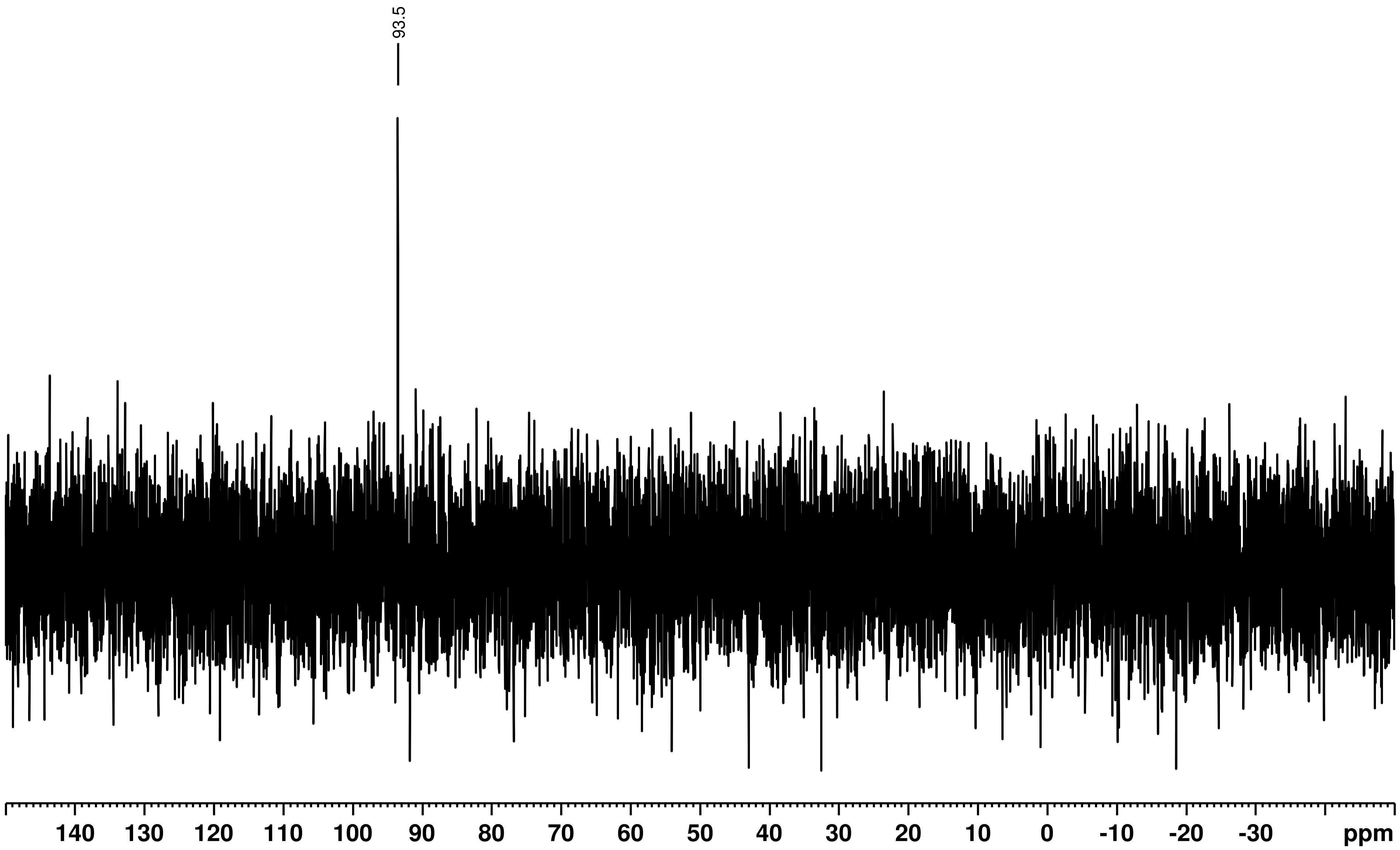

Supplementary Fig. 192. **^{1}H NMR** spectrum (500 MHz, C_6D_6, 298 K) of [iPr$_2$FSi(FBN)]$^+$[HCB$_{11}$H$_5$Br$_6$]$^-$ ([**8ac**(FBN)]$^+$[HCB$_{11}$H$_5$Br$_6$]$^-$) (* [iPr$_2$FSi(HCB$_{11}$H$_5$Br$_6$)])

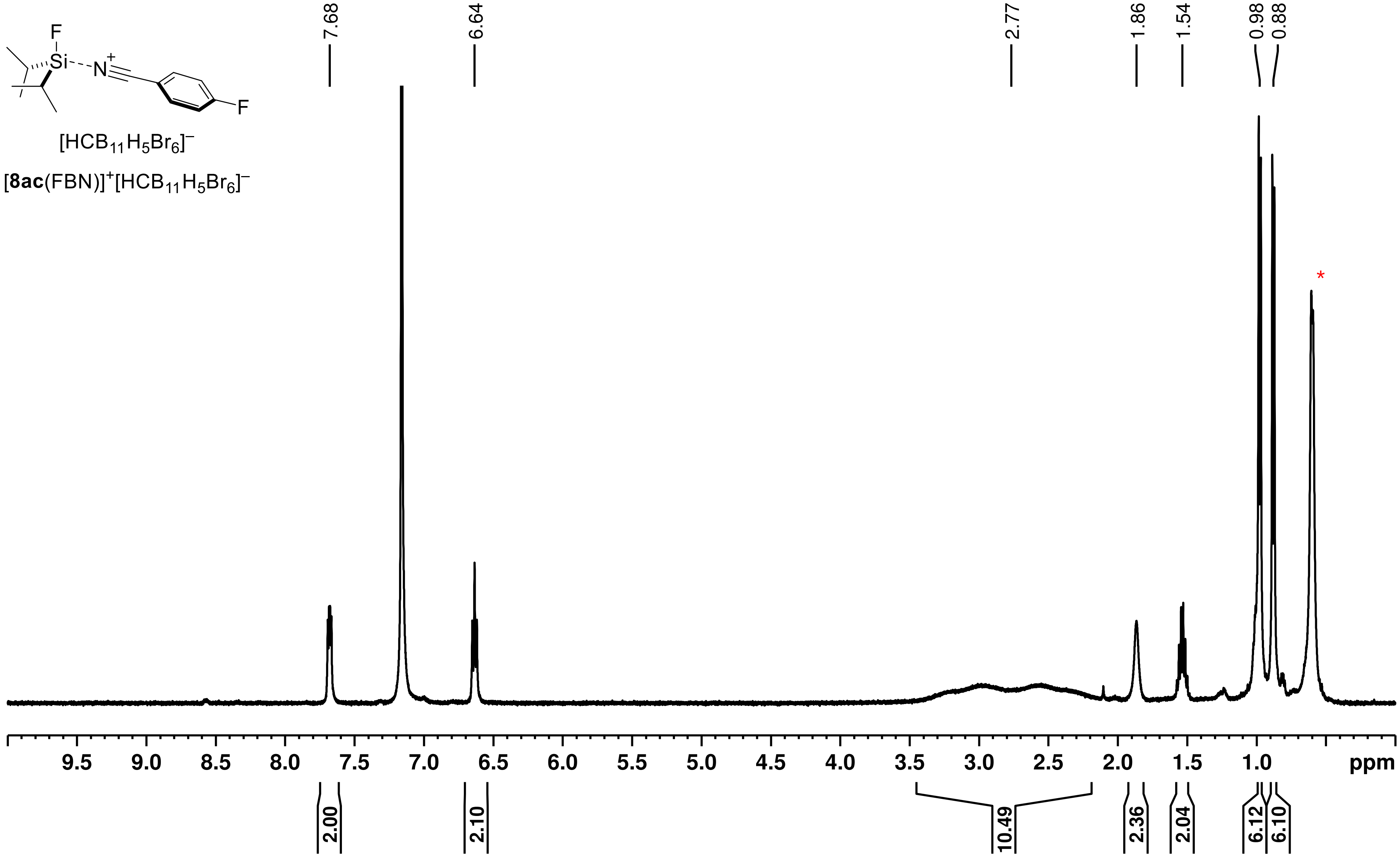

Supplementary Fig. 193. **^{11}B NMR** spectrum (160 MHz, C_6D_6, 298 K) of [*i*Pr_2FSi(FBN)]$^+$[$HCB_{11}H_5Br_6$]$^-$ ([**8ac**(FBN)]$^+$[$HCB_{11}H_5Br_6$]$^-$)

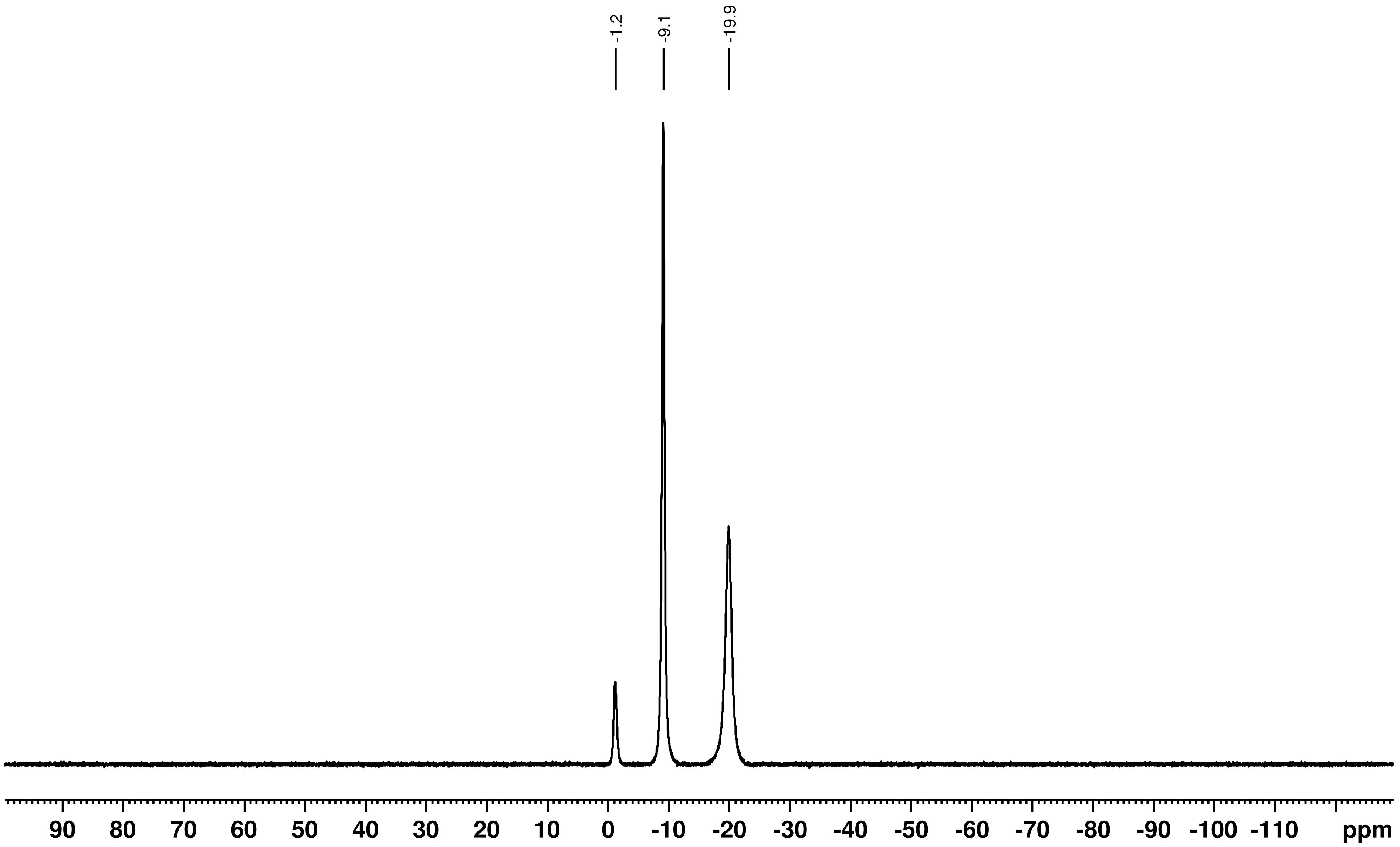

Supplementary Fig. 194. **^{13}C{^{1}H} NMR** spectrum (126 MHz, C_6D_6, 298 K) of [*i*Pr_2FSi(FBN)]$^+$[$HCB_{11}H_5Br_6$]$^-$ ([**8ac**(FBN)]$^+$[$HCB_{11}H_5Br_6$]$^-$) (* [*i*Pr_2FSi($HCB_{11}H_5Br_6$)])

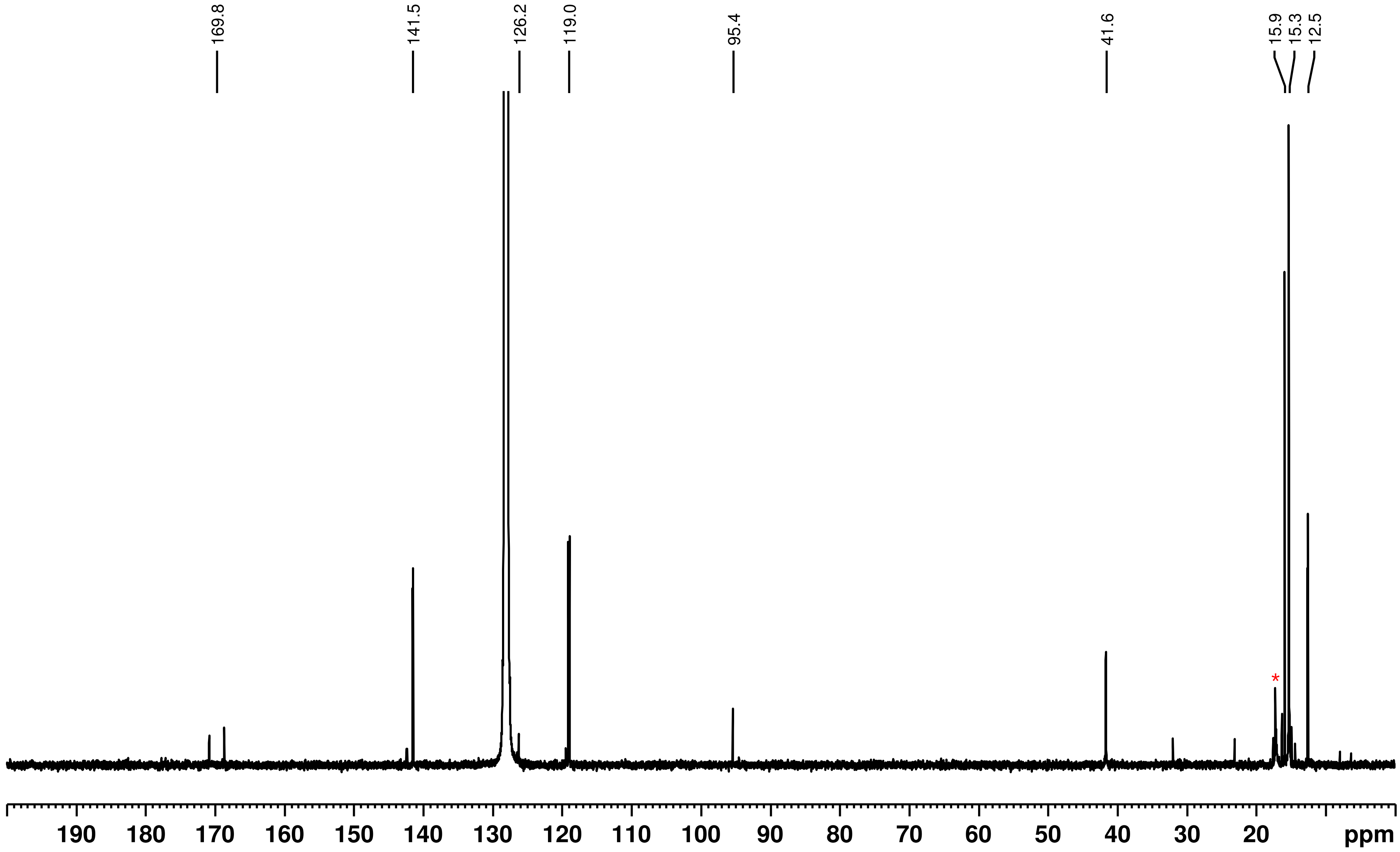

Supplementary Fig. 195. ^{19}**F NMR** spectrum (471 MHz, C_6D_6, 298 K) of [*i*Pr_2FSi(FBN)]$^+$[$HCB_{11}H_5Br_6$]$^-$ ([**8ac**(FBN)]$^+$[$HCB_{11}H_5Br_6$]$^-$) (* [*i*Pr_2FSi($HCB_{11}H_5Br_6$)])

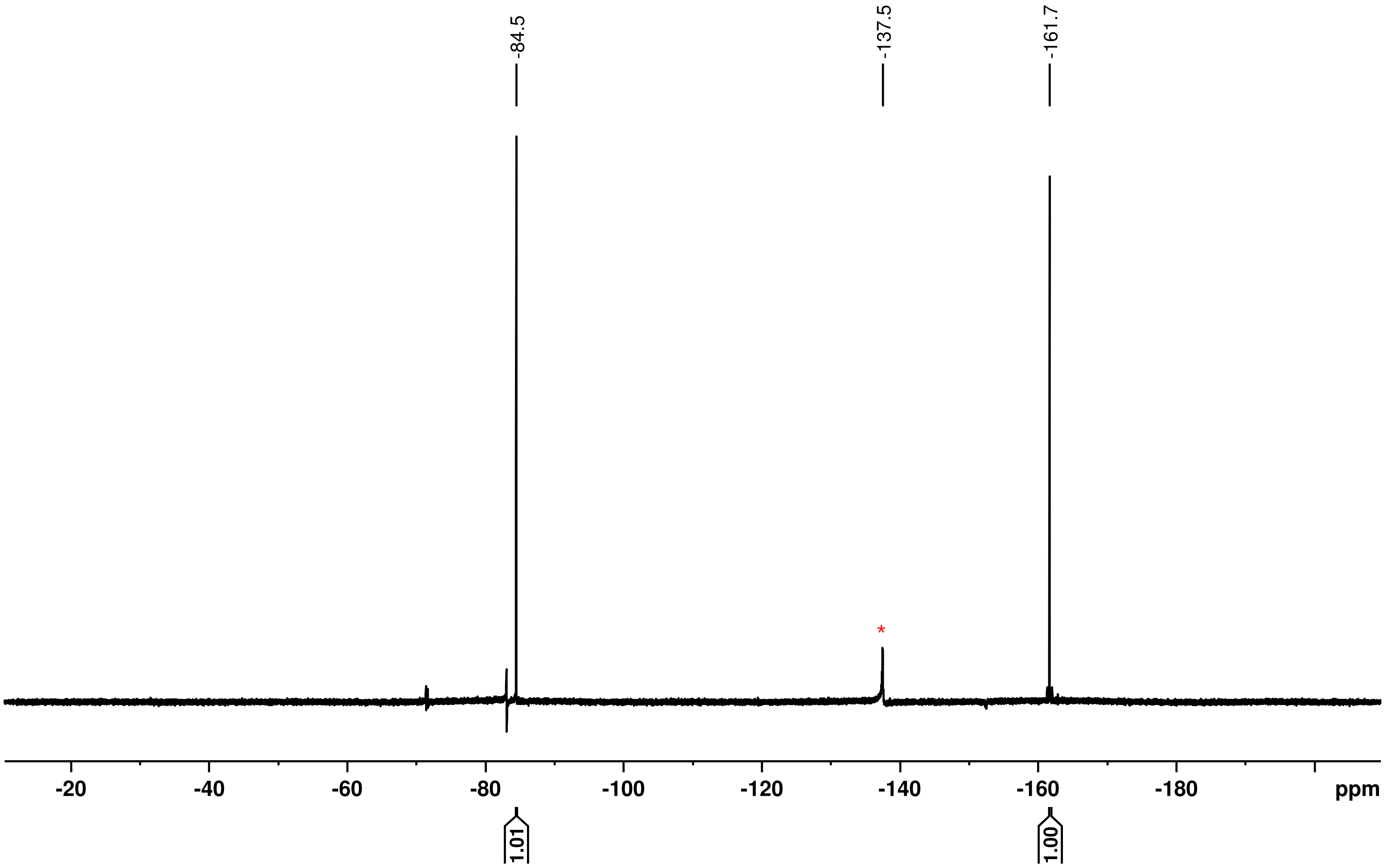

Supplementary Fig. 196. **$^{29}Si\{^1H\}$ DEPT NMR** spectrum (99 MHz, C_6D_6, 298 K, optimized for $J_{H,Si}$ = 7 Hz, 15.5°) of [*i*$Pr_2FSi(FBN)]^+[HCB_{11}H_5Br_6]^-$ ([**8ac**(FBN)]$^+[HCB_{11}H_5Br_6]^-$)

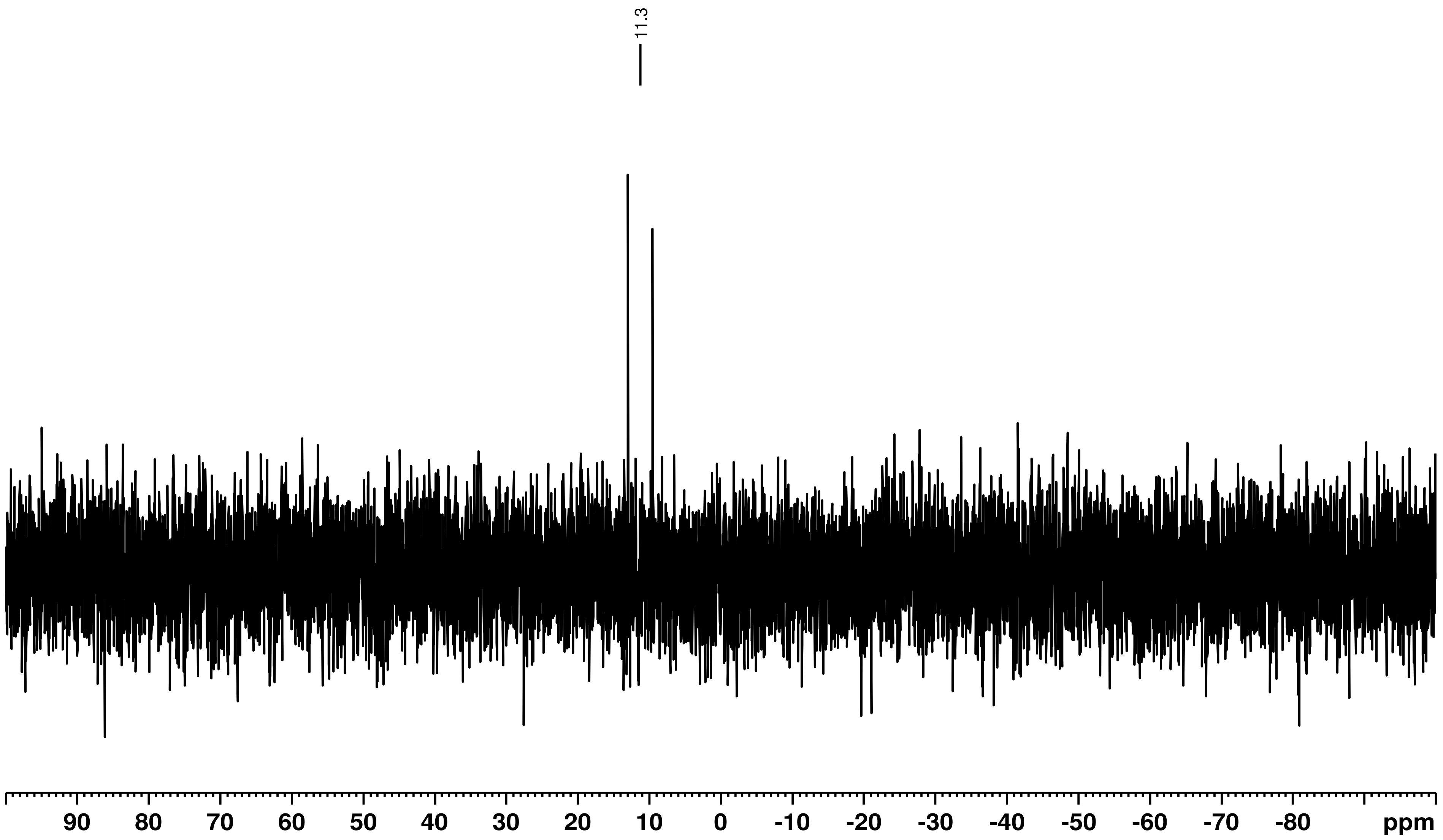

Supplementary Fig. 197. **^{1}H NMR** spectrum (500 MHz, C_6D_6, 298 K) of [*i*Pr_2ClSi(FBN)]$^+$[$HCB_{11}H_5Br_6$]$^-$ ([**8bc**(FBN)]$^+$[$HCB_{11}H_5Br_6$]$^-$) (* [*i*Pr_2ClSi($HCB_{11}H_5Br_6$)])

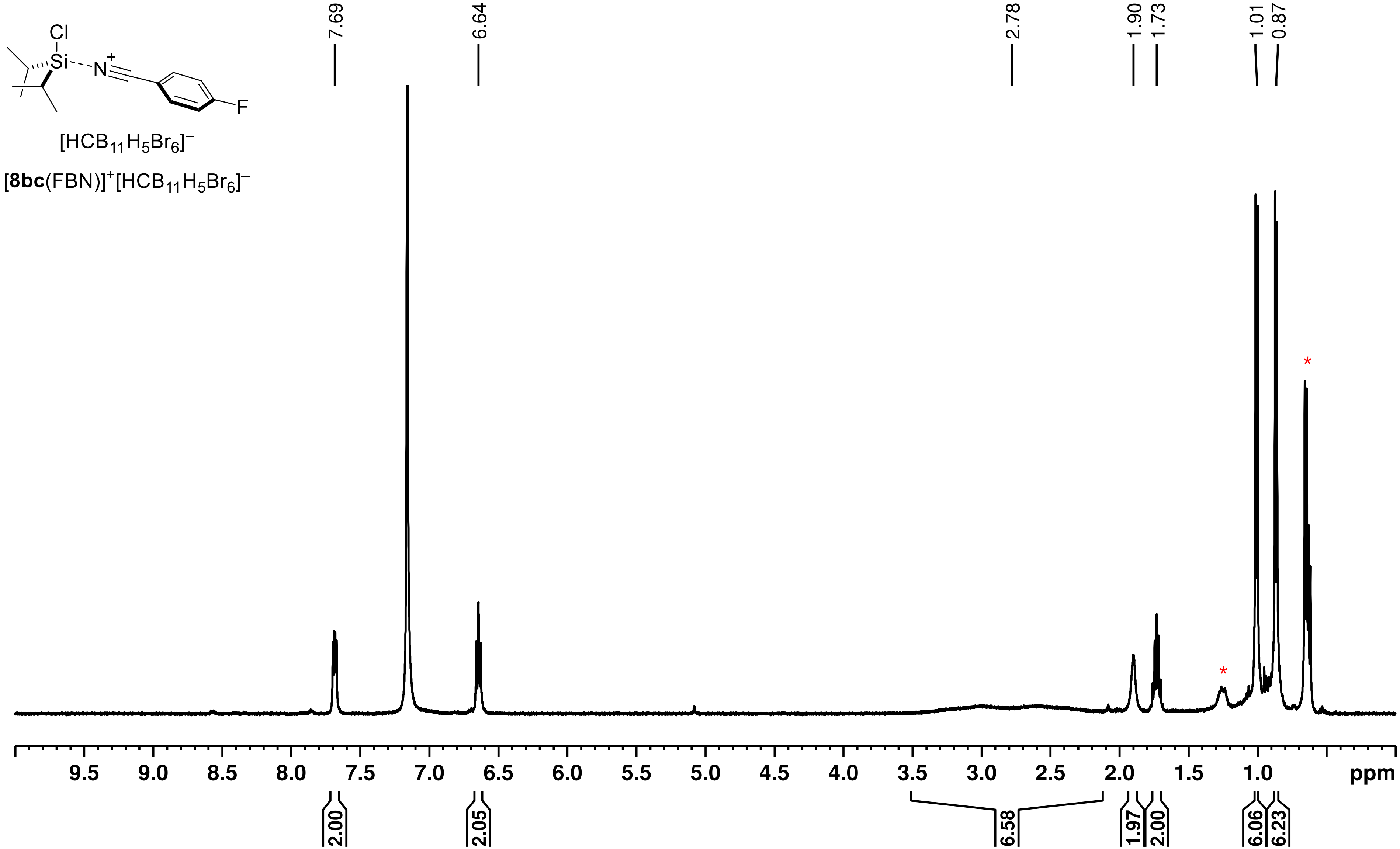

Supplementary Fig. 198. **^{11}B NMR** spectrum (160 MHz, C_6D_6, 298 K) of [*i*$Pr_2ClSi(FBN)]^+[HCB_{11}H_5Br_6]^-$ ([**8bc**(FBN)]$^+[HCB_{11}H_5Br_6]^-$)

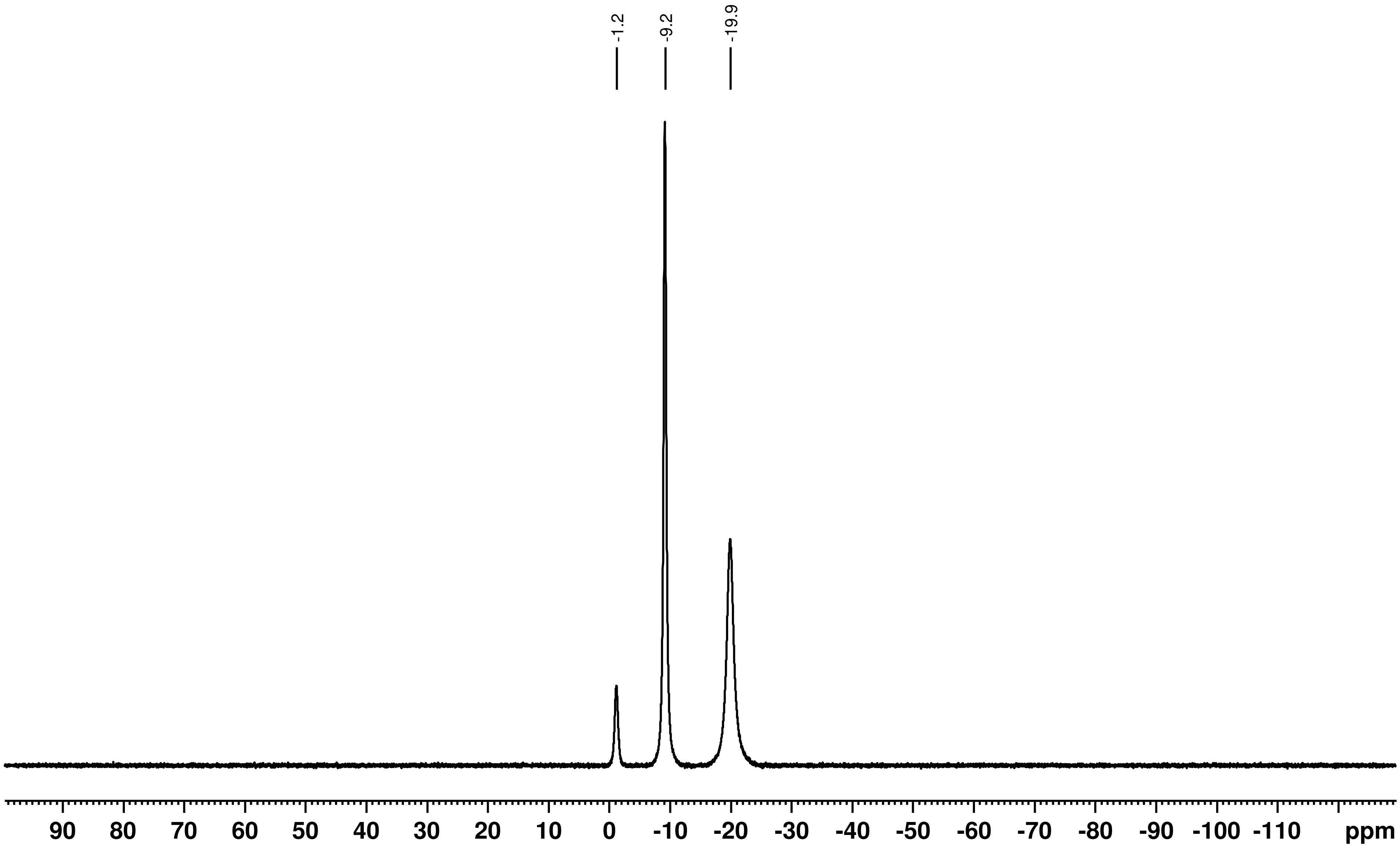

Supplementary Fig. 199. **$^{13}C\{^{1}H\}$ NMR** spectrum (176 MHz, C_6D_6, 298 K) of [*i*Pr_2ClSi(FBN)]$^+$[$HCB_{11}H_5Br_6$]$^-$ ([**8bc**(FBN)]$^+$[$HCB_{11}H_5Br_6$]$^-$) (* [*i*Pr_2ClSi($HCB_{11}H_5Br_6$)])

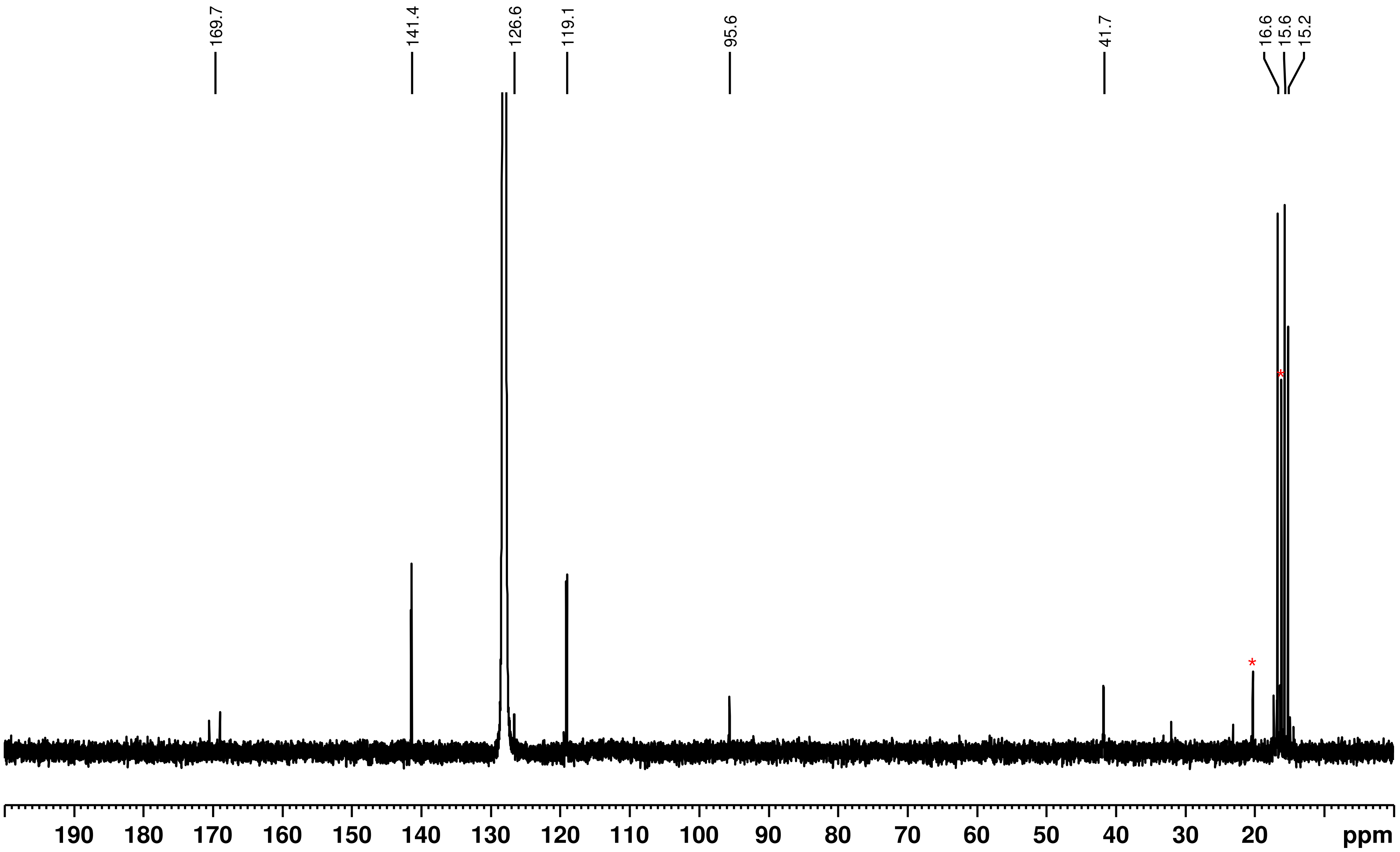

Supplementary Fig. 200. ^{19}**F NMR** spectrum (471 MHz, C_6D_6, 298 K) of [*i*$Pr_2ClSi(FBN)]^+[HCB_{11}H_5Br_6]^-$ ([**8bc**(FBN)]$^+[HCB_{11}H_5Br_6]^-$)

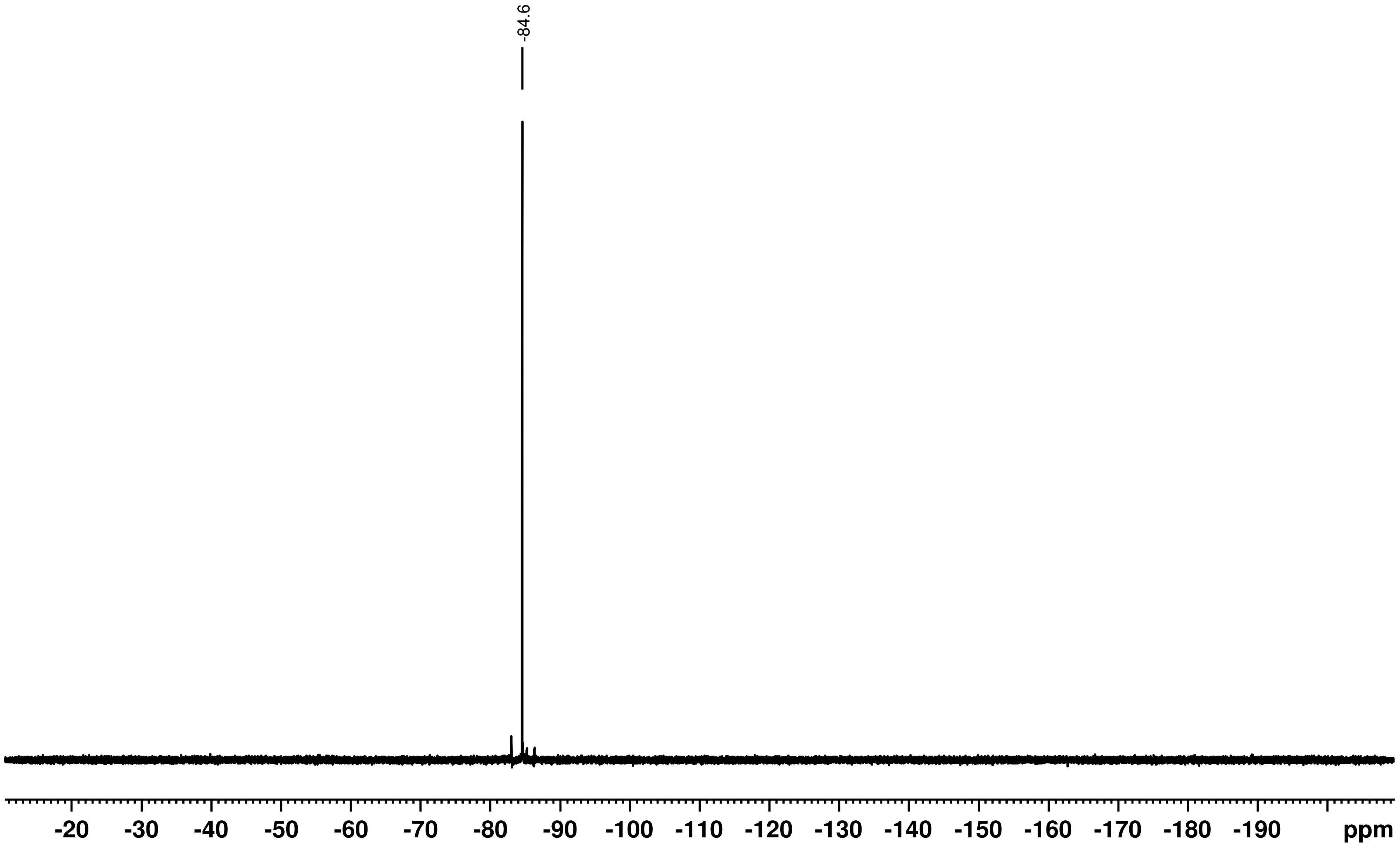

Supplementary Fig. 201. **^{29}Si{^{1}H} DEPT NMR** spectrum (99 MHz, C_6D_6, 298 K, optimized for $J_{H,Si}$ = 7 Hz, 15.5°) of [*i*Pr_2ClSi(FBN)]$^+$[$HCB_{11}H_5Br_6$] ([**8bc**(FBN)]$^+$[$HCB_{11}H_5Br_6$]$^-$)

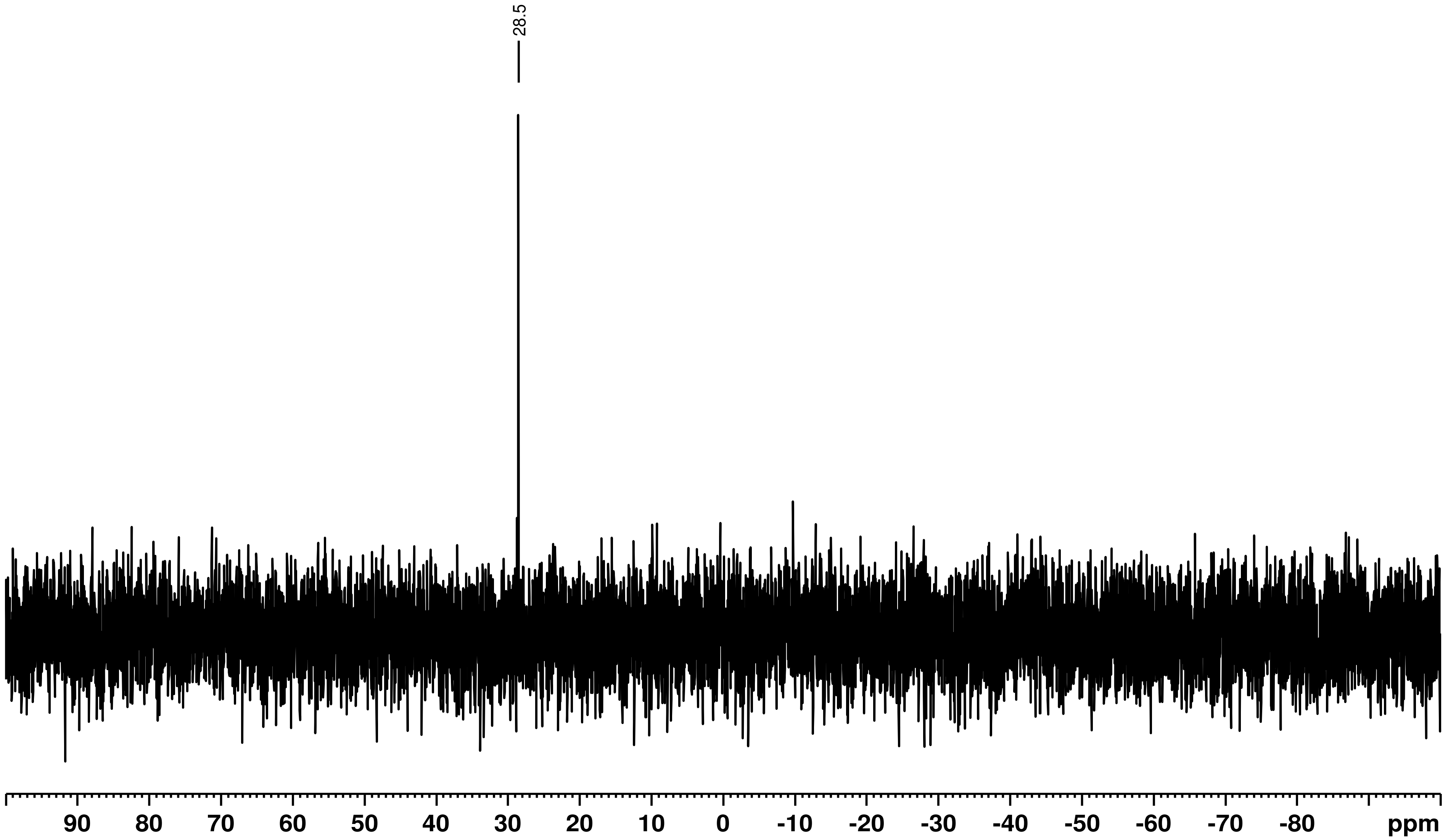

Supplementary Fig. 202. **^{1}H NMR** spectrum (500 MHz, C_6D_6, 298 K) of [*i*Pr_2BrSi(FBN)]$^+$[$HCB_{11}H_5Br_6$]$^-$ ([**8cc**(FBN)]$^+$[$HCB_{11}H_5Br_6$]$^-$) (* [*i*Pr_2BrSi($HCB_{11}H_5Br_6$)])

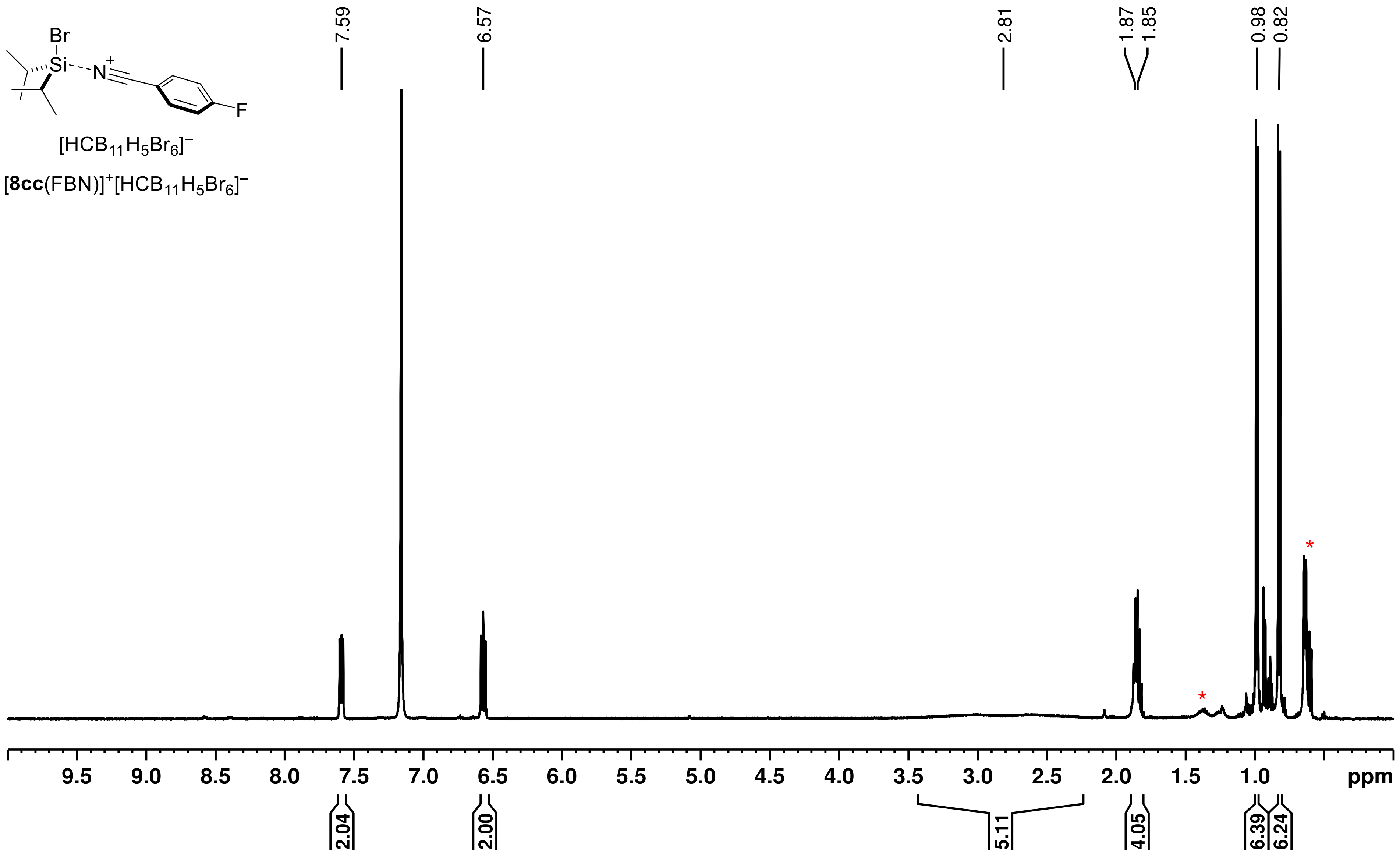

Supplementary Fig. 203. **^{11}B NMR** spectrum (160 MHz, C_6D_6, 298 K) of [*i*Pr_2BrSi(FBN)]$^+$[$HCB_{11}H_5Br_6$]$^-$ ([**8cc**(FBN)]$^+$[$HCB_{11}H_5Br_6$]$^-$)

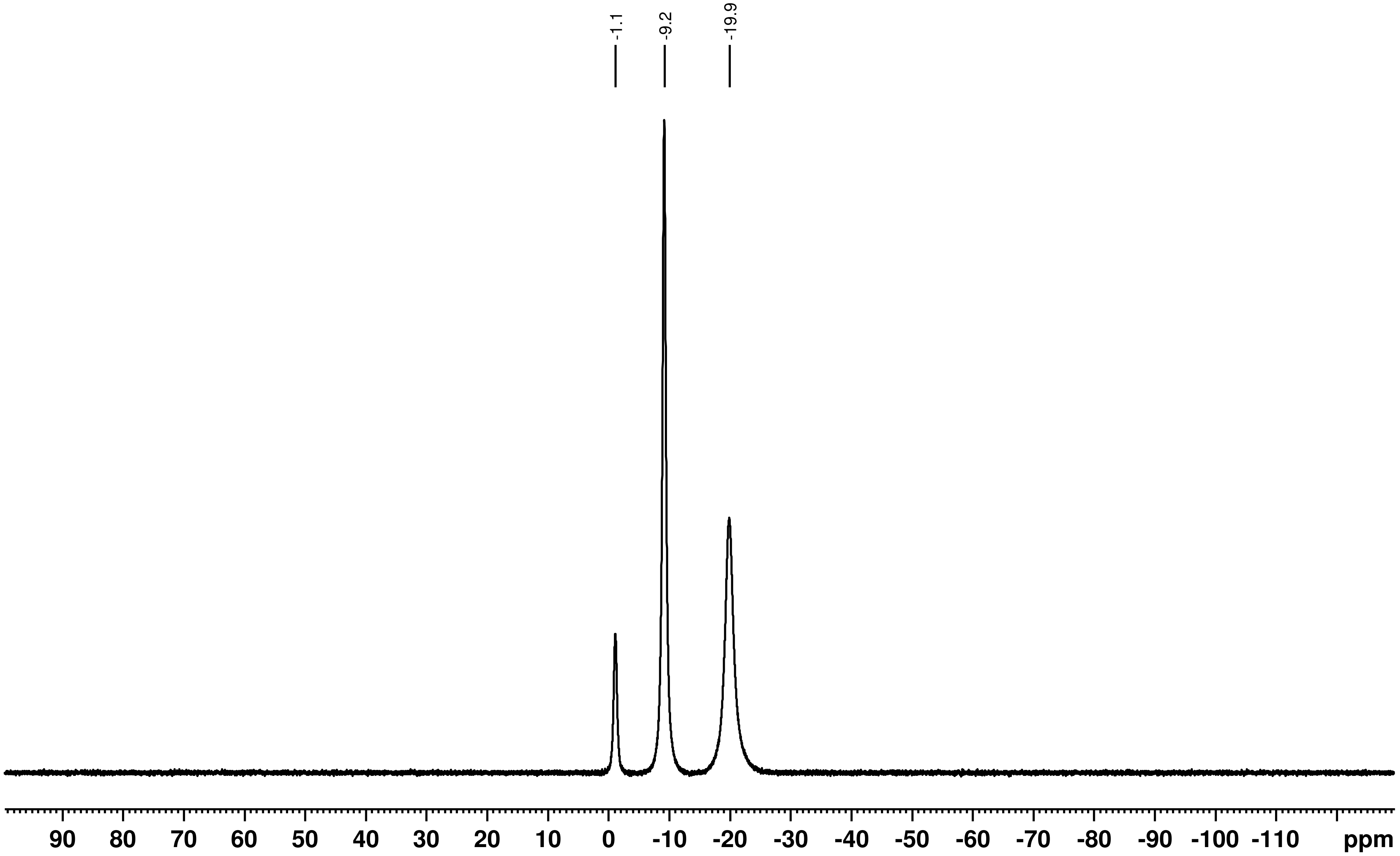

Supplementary Fig. 204. **^{13}C{^{1}H} NMR** spectrum (126 MHz, C_6D_6, 298 K) of [*i*Pr_2BrSi(FBN)]$^+$[$HCB_{11}H_5Br_6$]$^-$ ([**8cc**(FBN)]$^+$[$HCB_{11}H_5Br_6$]$^-$)

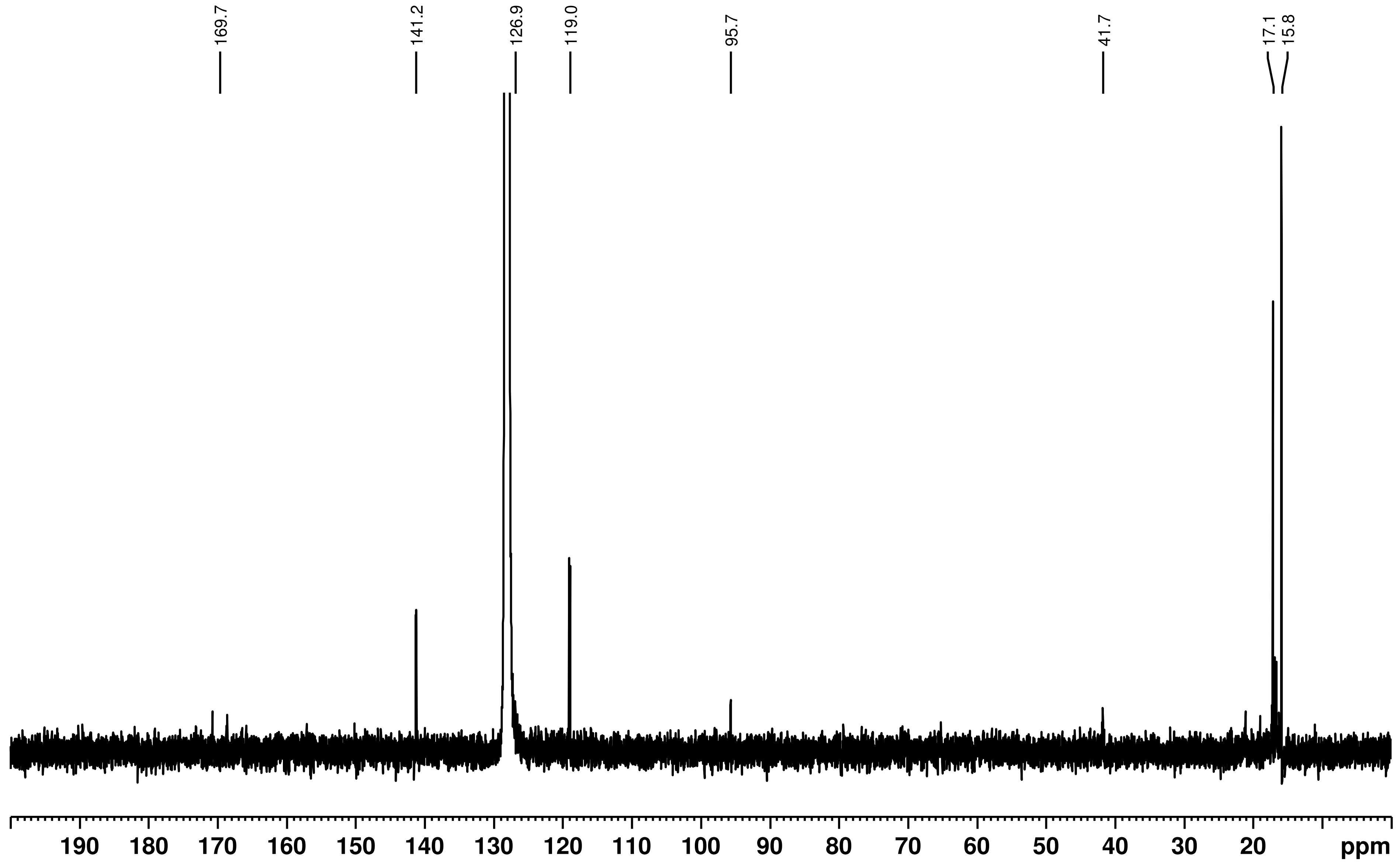

Supplementary Fig. 205. ^{19}**F NMR** spectrum (471 MHz, C_6D_6, 298 K) of [*i*Pr_2BrSi(FBN)]$^+$[$HCB_{11}H_5Br_6$]$^-$ ([**8cc**(FBN)]$^+$[$HCB_{11}H_5Br_6$]$^-$)

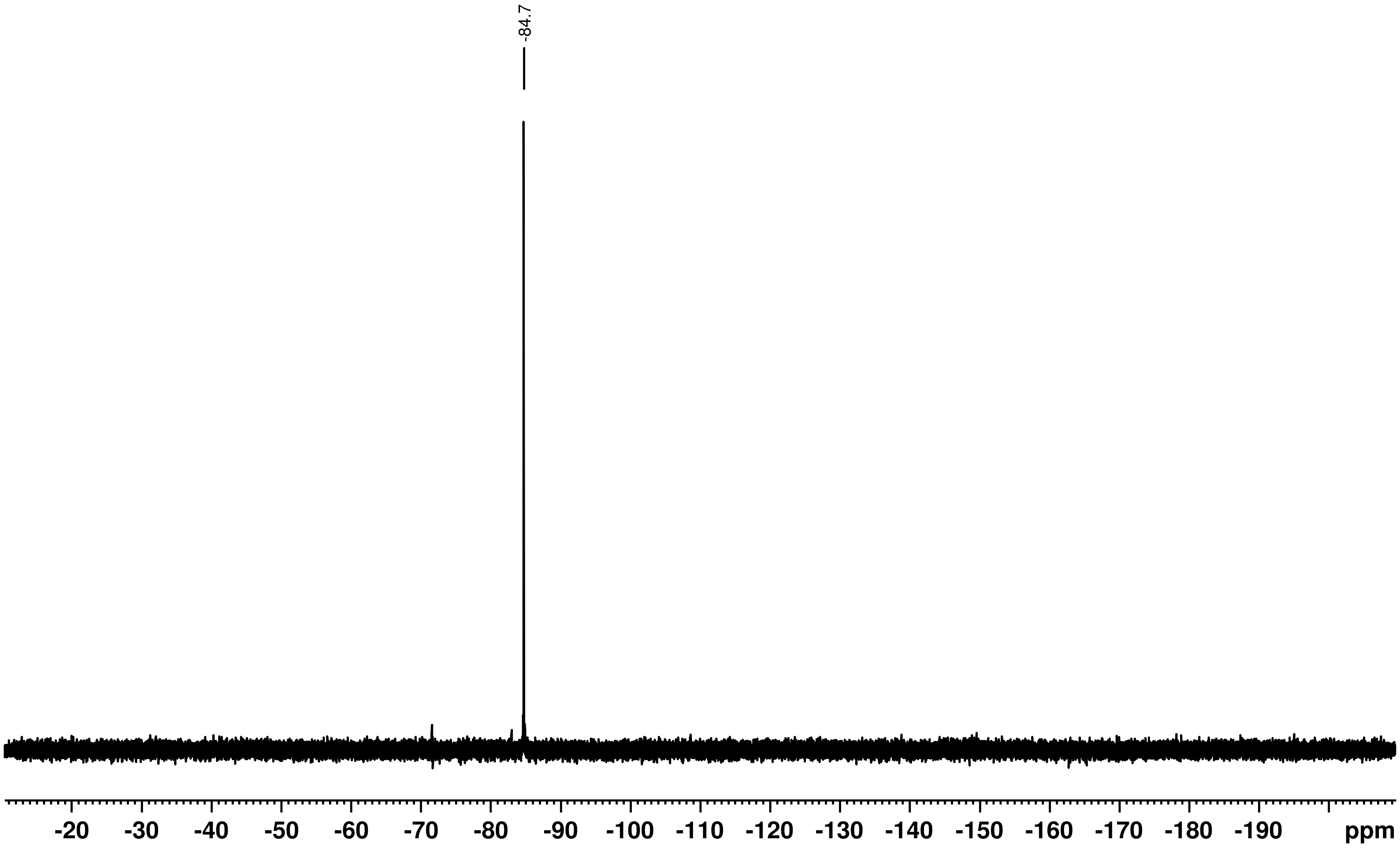

Supplementary Fig. 206. **^{29}Si{^{1}H} DEPT NMR** spectrum (99 MHz, C_6D_6, 298 K, optimized for $J_{H,Si}$ = 7 Hz, 15.5°) of [*i*Pr_2BrSi(FBN)]$^+$[$HCB_{11}H_5Br_6$]$^-$ ([**8cc**(FBN)]$^+$[$HCB_{11}H_5Br_6$]$^-$)

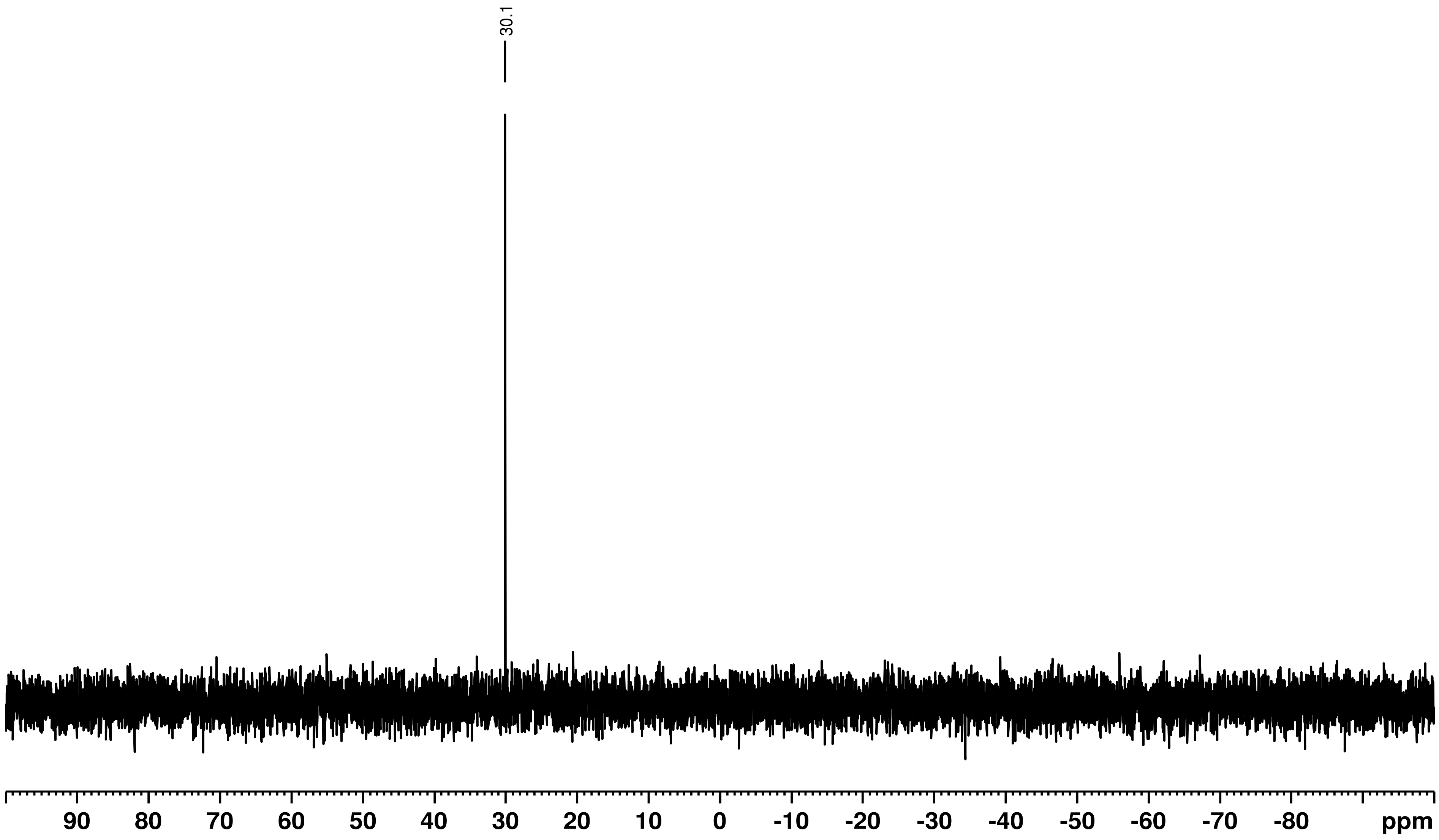

Supplementary Fig. 207. **^{1}H NMR** spectrum (500 MHz, C_6D_6, 298 K) of [*i*Pr_2ISi(FBN)]$^+$[$HCB_{11}H_5Br_6$]$^-$ ([**8dc**(FBN)]$^+$[$HCB_{11}H_5Br_6$]$^-$) (* [*i*Pr_2ISi($HCB_{11}H_5Br_6$)])

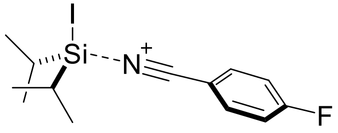

[$HCB_{11}H_5Br_6$]$^-$

[**8dc**(FBN)]$^+$[$HCB_{11}H_5Br_6$]$^-$

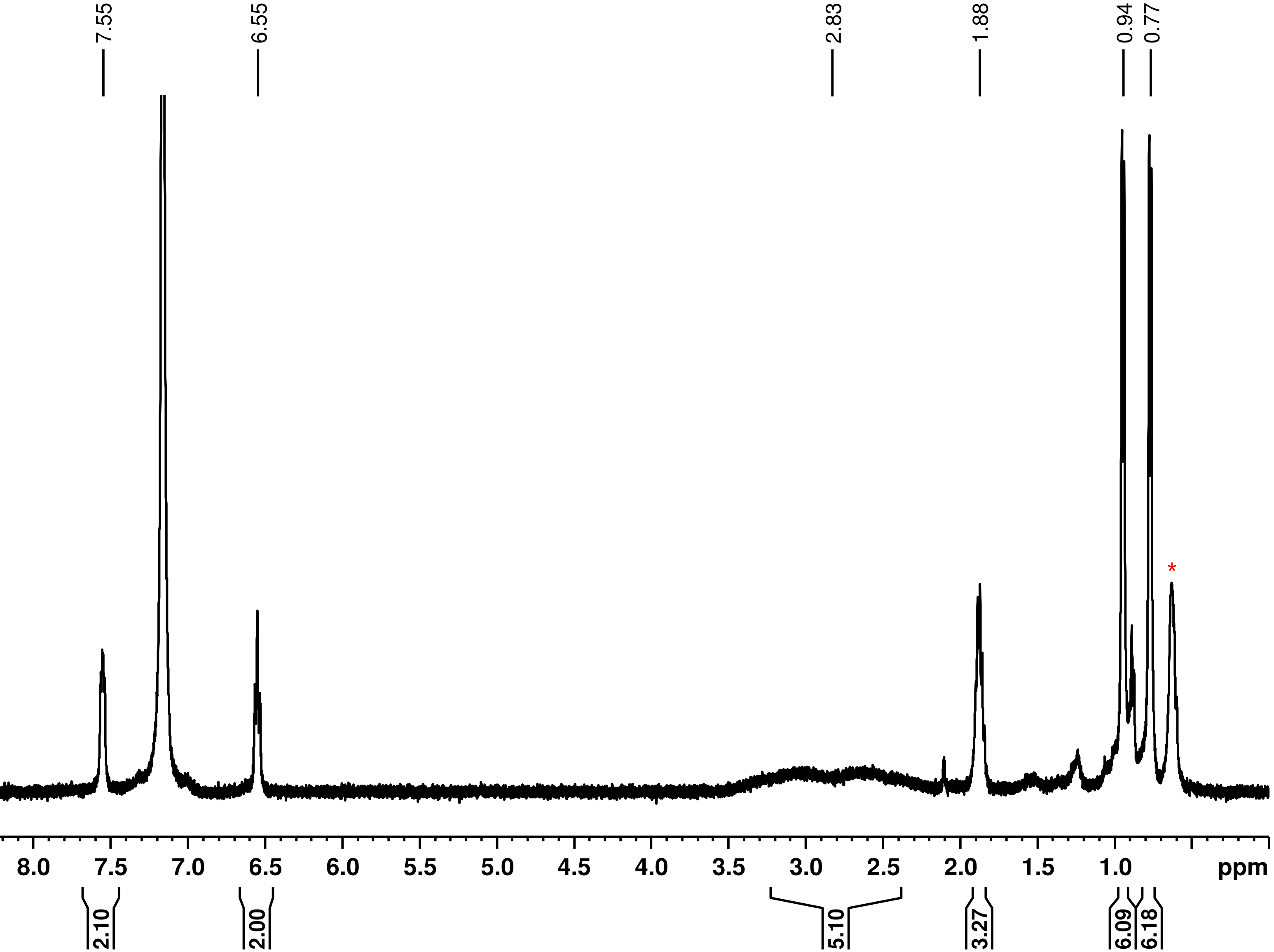

Supplementary Fig. 208. ^{11}**B NMR** spectrum (160 MHz, C_6D_6, 298 K) of [*i*Pr_2ISi(FBN)]$^+$[$HCB_{11}H_5Br_6$]$^-$ ([**8dc**(FBN)]$^+$[$HCB_{11}H_5Br_6$]$^-$)

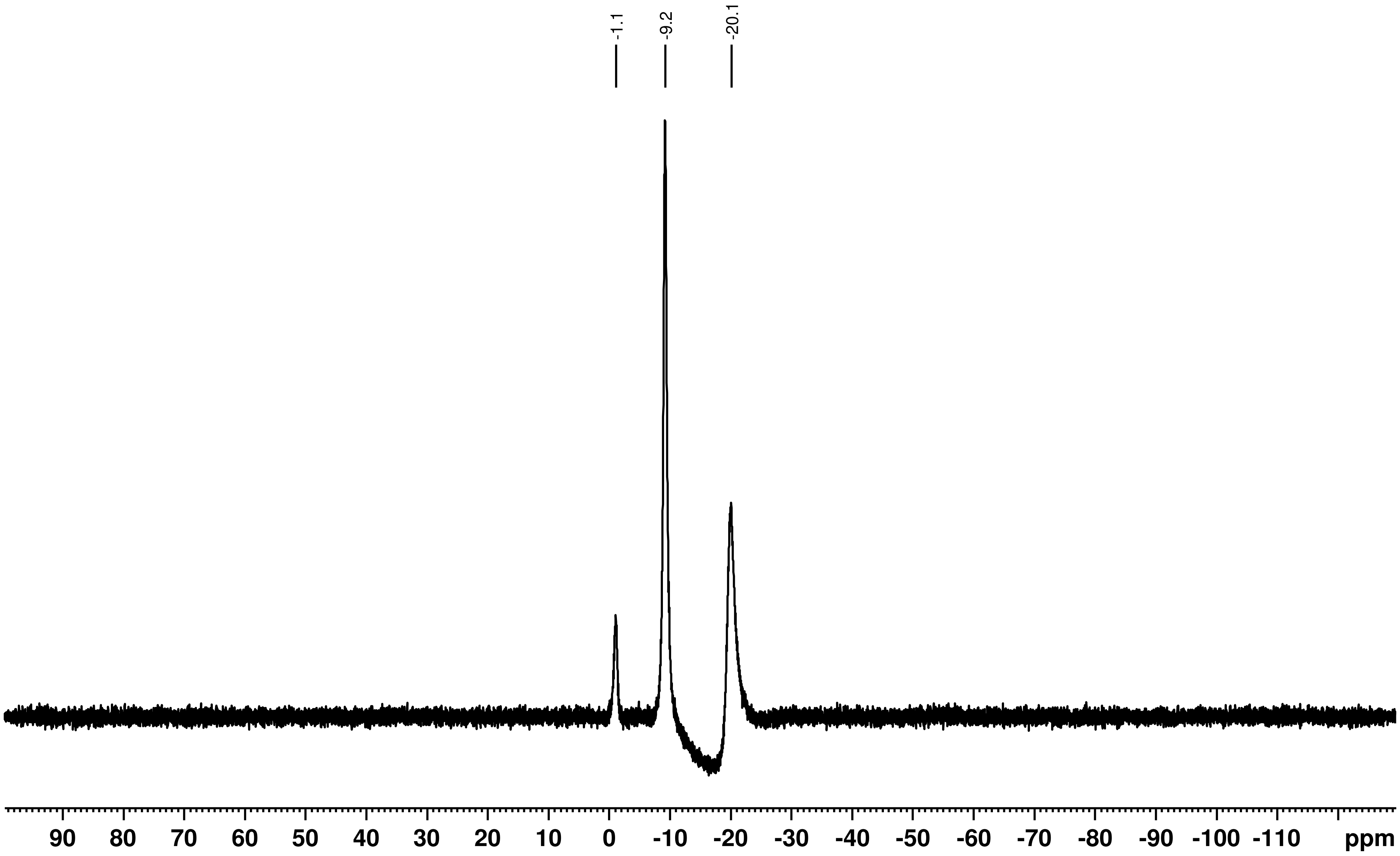

Supplementary Fig. 209. **$^{13}C\{^1H\}$ NMR** spectrum (176 MHz, C_6D_6, 298 K) of [*i*$Pr_2ISi(FBN)]^+[HCB_{11}H_5Br_6]^-$ ([**8dc**(FBN)]$^+[HCB_{11}H_5Br_6]^-$)

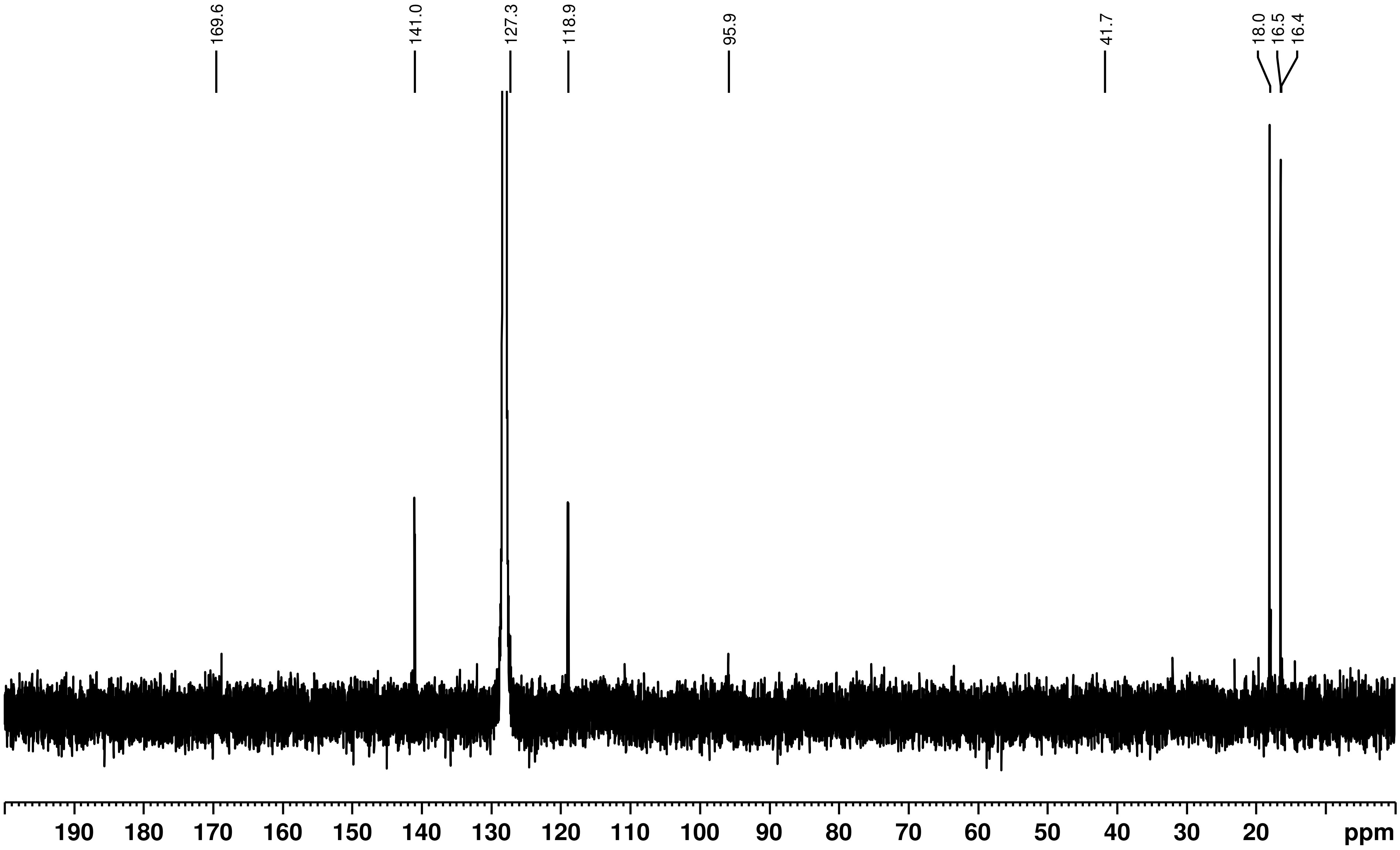

Supplementary Fig. 210. ^{19}**F NMR** spectrum (471 MHz, C_6D_6, 298 K) of [*i*$Pr_2ISi(FBN)]^+[HCB_{11}H_5Br_6]^-$ ([**8dc**$(FBN)]^+[HCB_{11}H_5Br_6]^-$)

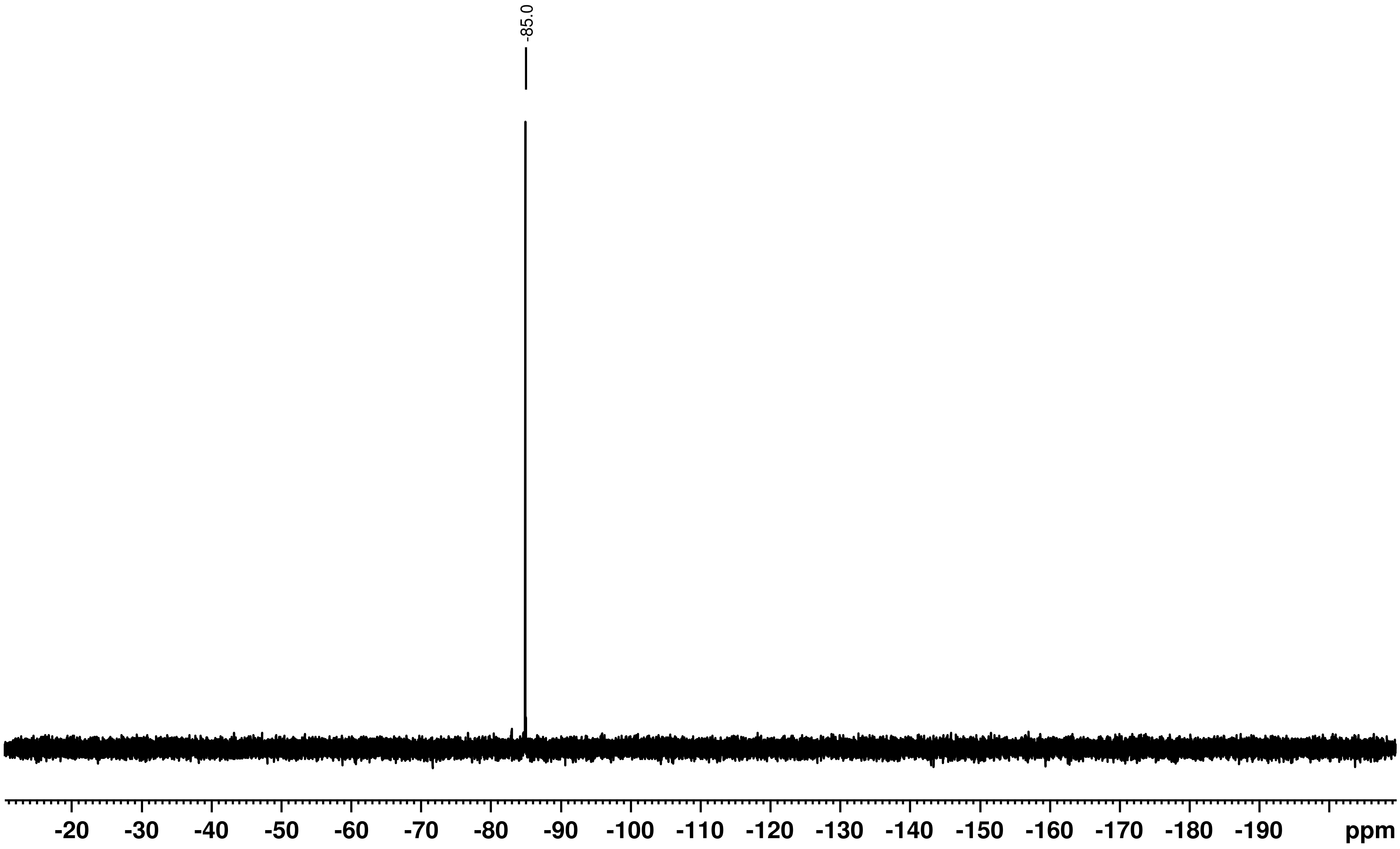

Supplementary Fig. 211. **$^{1}H,^{29}Si$ HMQC NMR** (500/99 MHz, C_6D_6, 298 K, optimized for *J* = 7 Hz) of [*i*Pr_2ISi(FBN)]$^+$[$HCB_{11}H_5Br_6$]$^-$ ([**8dc**(FBN)]$^+$[$HCB_{11}H_5Br_6$]$^-$)

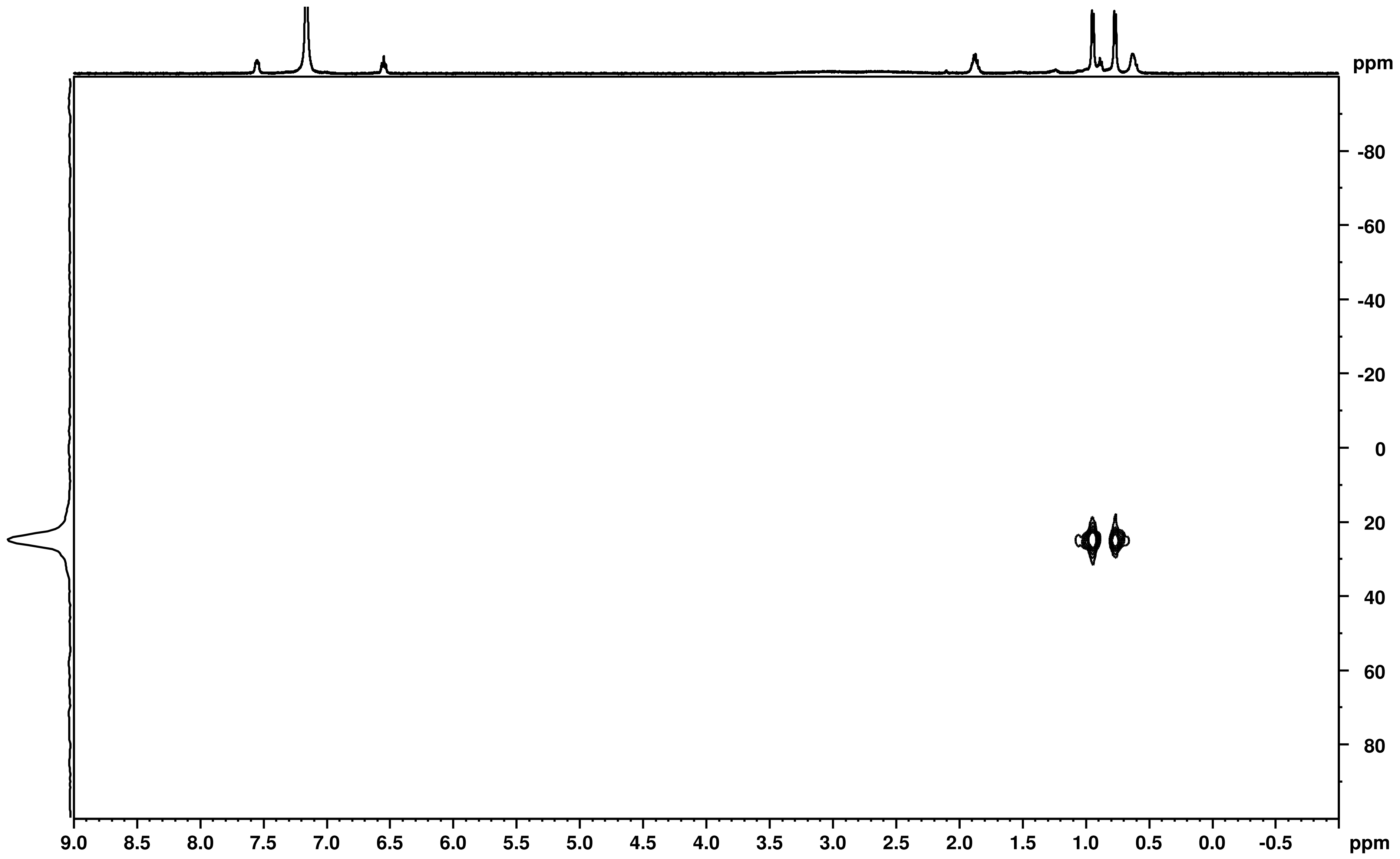

Supplementary Fig. 212. **^{1}H NMR** spectrum (500 MHz, C_6D_6, 298 K) of [*i*Pr_2HSi(FBN)]$^{+}$[$HCB_{11}H_5Br_6$]$^{-}$ ([**5c**(FBN)]$^{+}$[$HCB_{11}H_5Br_6$]$^{-}$) (* [*i*Pr_2HSi($HCB_{11}H_5Br_6$)])

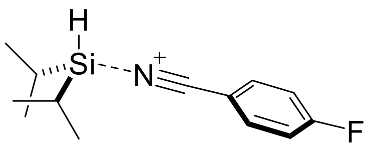

[$HCB_{11}H_5Br_6$]$^{-}$

[**5c**(FBN)]$^{+}$[$HCB_{11}H_5Br_6$]$^{-}$

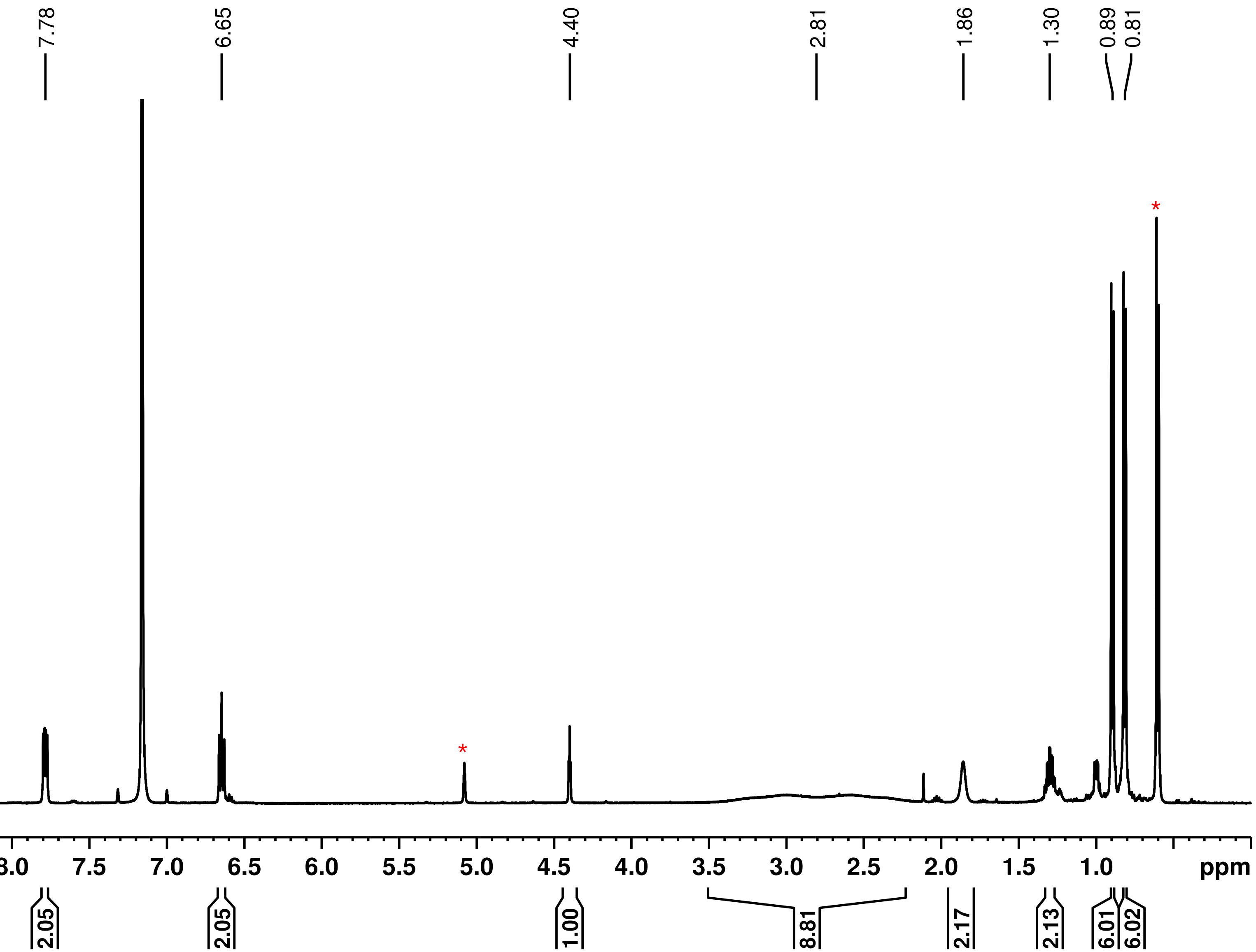

Supplementary Fig. 213. ^{11}**B NMR** spectrum (160 MHz, C_6D_6, 298 K) of [*i*$Pr_2HSi(FBN)]^+[HCB_{11}H_5Br_6]^-$ ([**5c**(FBN)]$^+[HCB_{11}H_5Br_6]^-$)

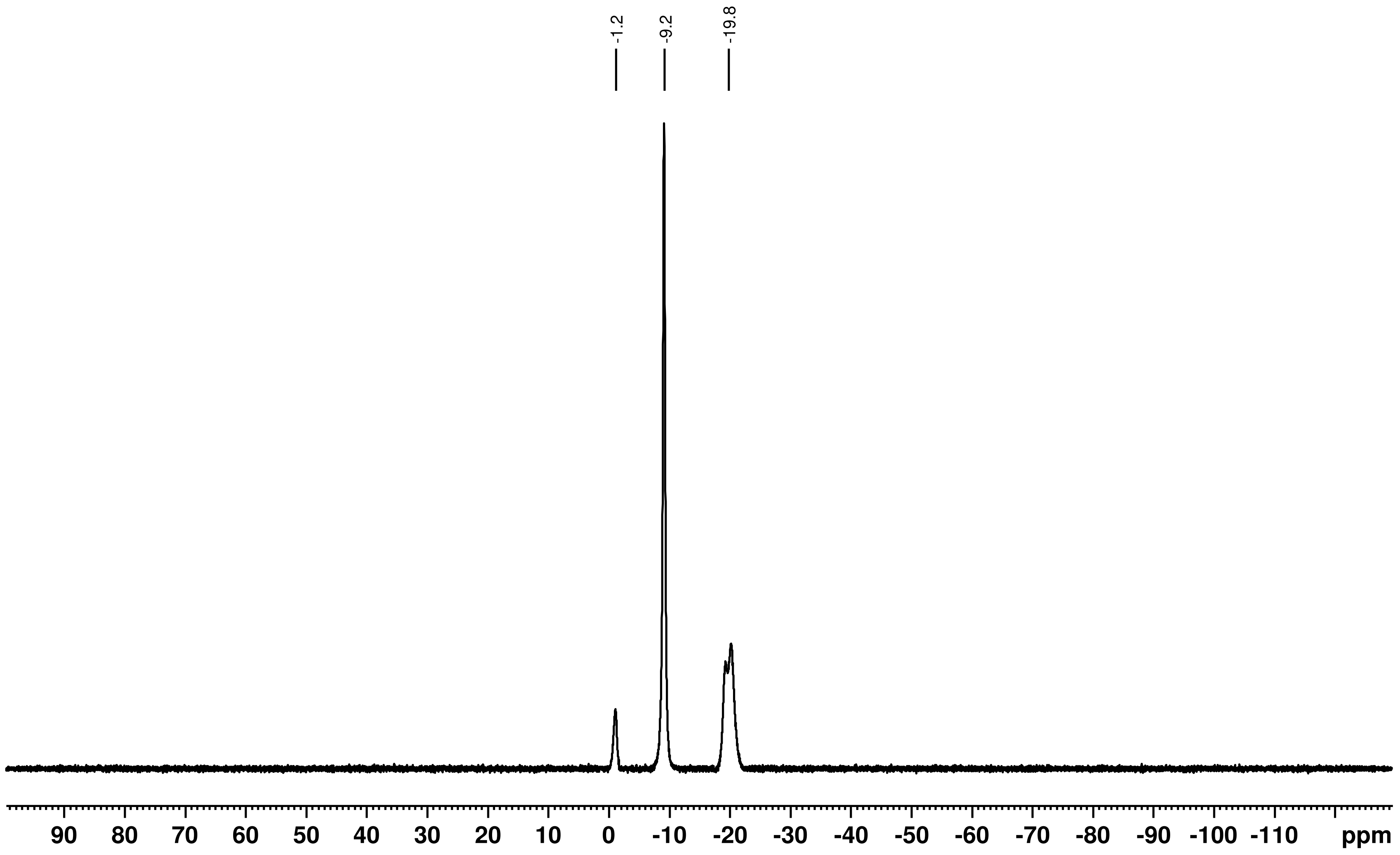

Supplementary Fig. 214. **^{13}C{^{1}H} NMR** spectrum (126 MHz, C_6D_6, 298 K) of [*i*Pr_2HSi(FBN)]$^+$[$HCB_{11}H_5Br_6$]$^-$ ([**5c**(FBN)]$^+$[$HCB_{11}H_5Br_6$]$^-$) (* [*i*Pr_2HSi($HCB_{11}H_5Br_6$)])

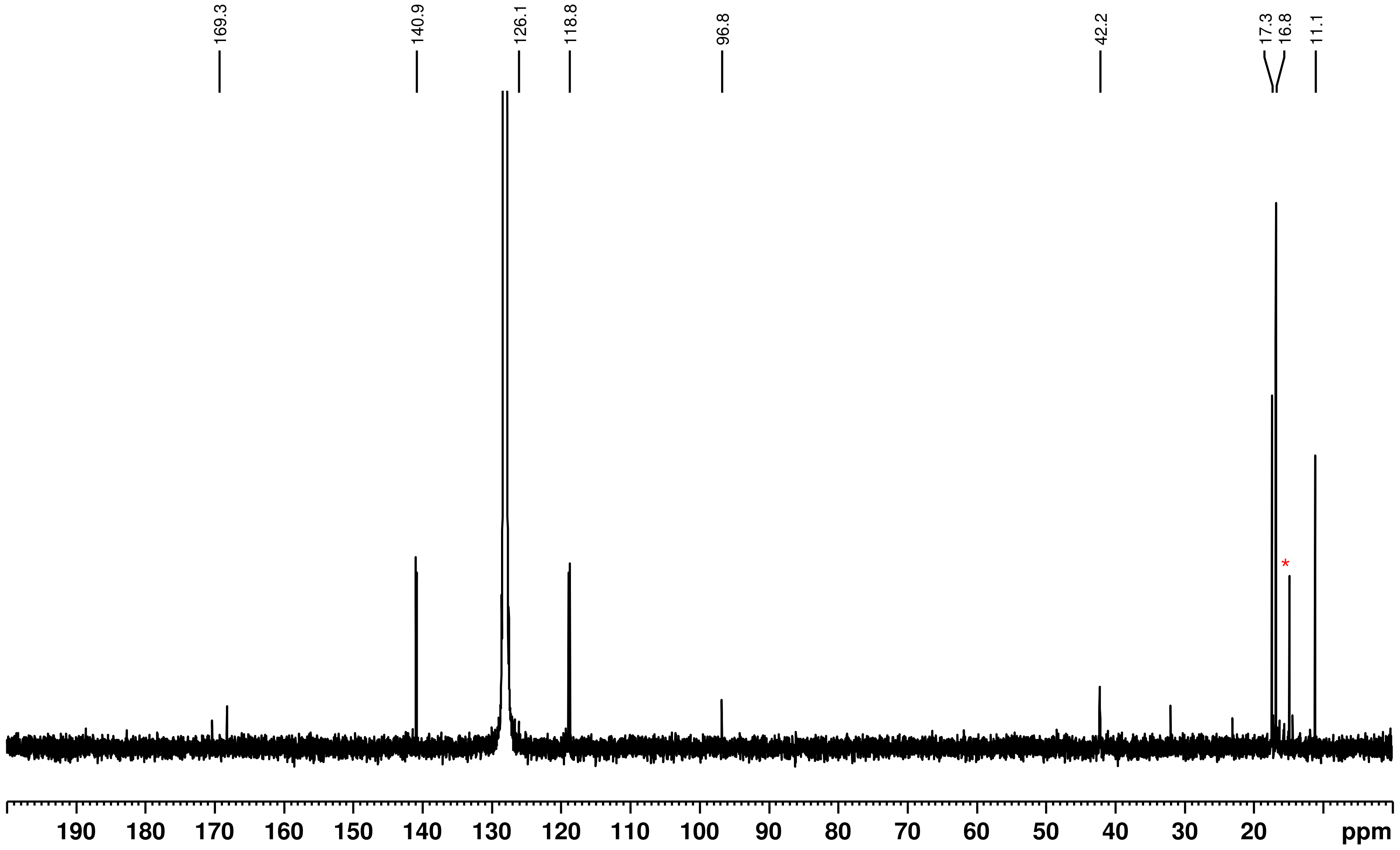

Supplementary Fig. 215. ^{19}**F NMR** spectrum (471 MHz, C_6D_6, 298 K) of [*i*$Pr_2HSi(FBN)]^+[HCB_{11}H_5Br_6]^-$ ([**5c**(FBN)]$^+[HCB_{11}H_5Br_6]^-$)

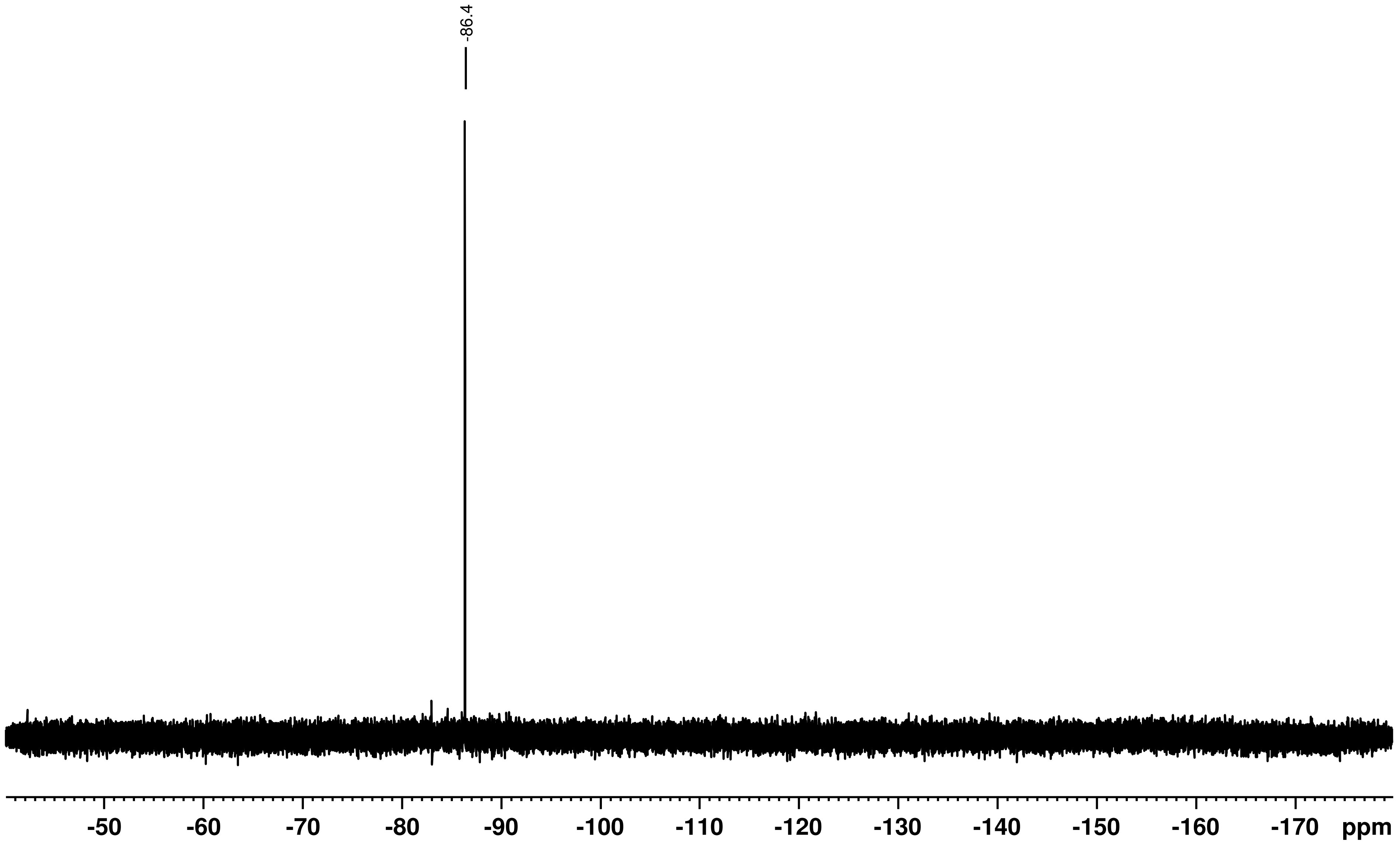

Supplementary Fig. 216. **^{29}Si{^{1}H} DEPT NMR** spectrum (99 MHz, C_6D_6, 298 K, optimized for $J_{H,Si}$ = 7 Hz, 15.5°) of [*i*Pr_2HSi(FBN)]$^+$[$HCB_{11}H_5Br_6$]$^-$ ([**5c**(FBN)]$^+$[$HCB_{11}H_5Br_6$]$^-$) (* [*i*Pr_2HSi($HCB_{11}H_5Br_6$)])

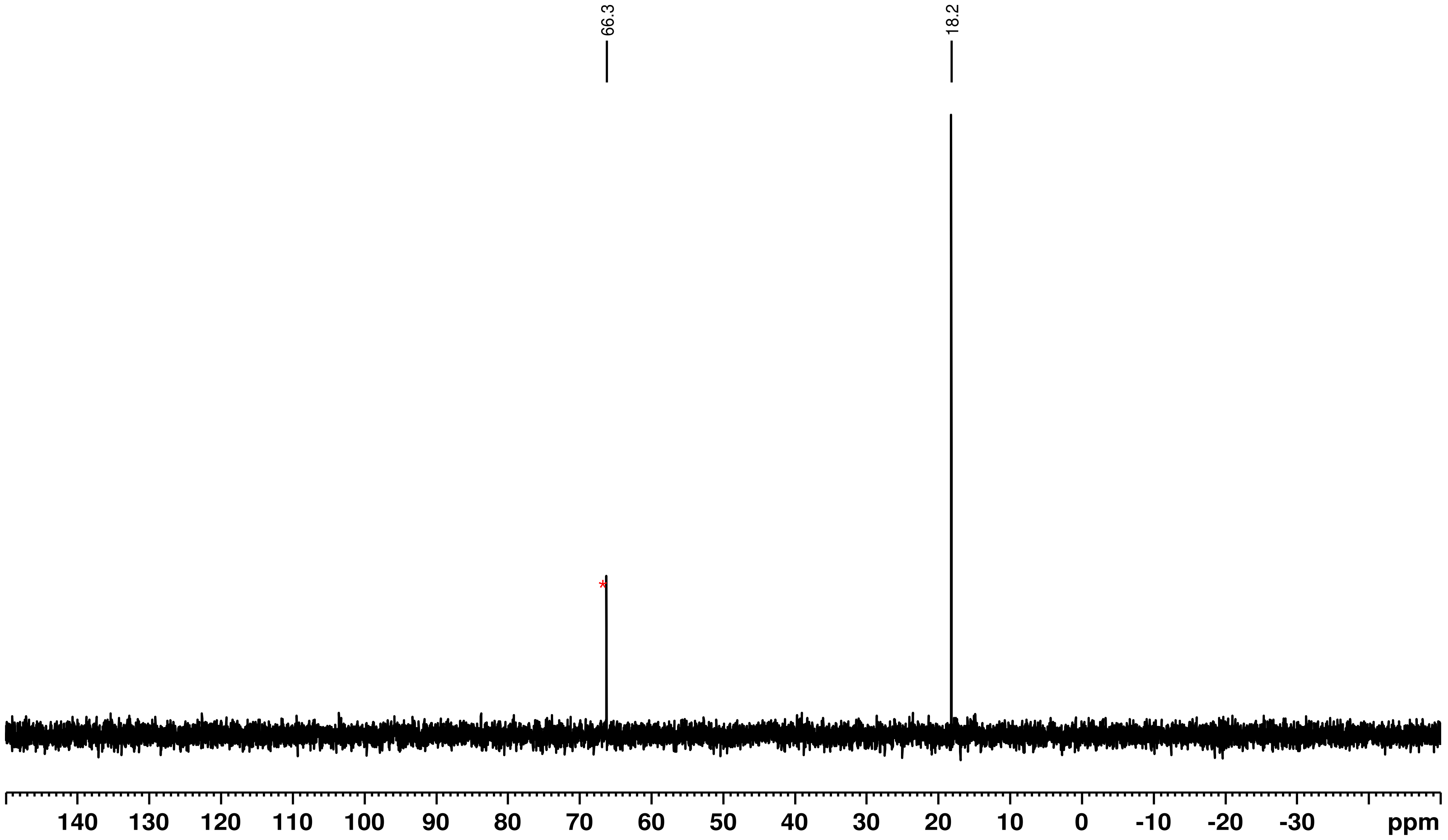

Supplementary Fig. 217. ^{1}**H NMR** spectrum (500 MHz, C_6D_6, 298 K) of $[i\text{Pr}_3\text{Si(FBN)}]^+[\text{HCB}_{11}\text{H}_5\text{Br}_6]^-$ ([**2c**(FBN)]$^+[\text{HCB}_{11}\text{H}_5\text{Br}_6]^-$) (* $[i\text{Pr}_3\text{Si}(\text{HCB}_{11}\text{H}_5\text{Br}_6)]$)

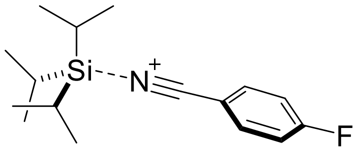

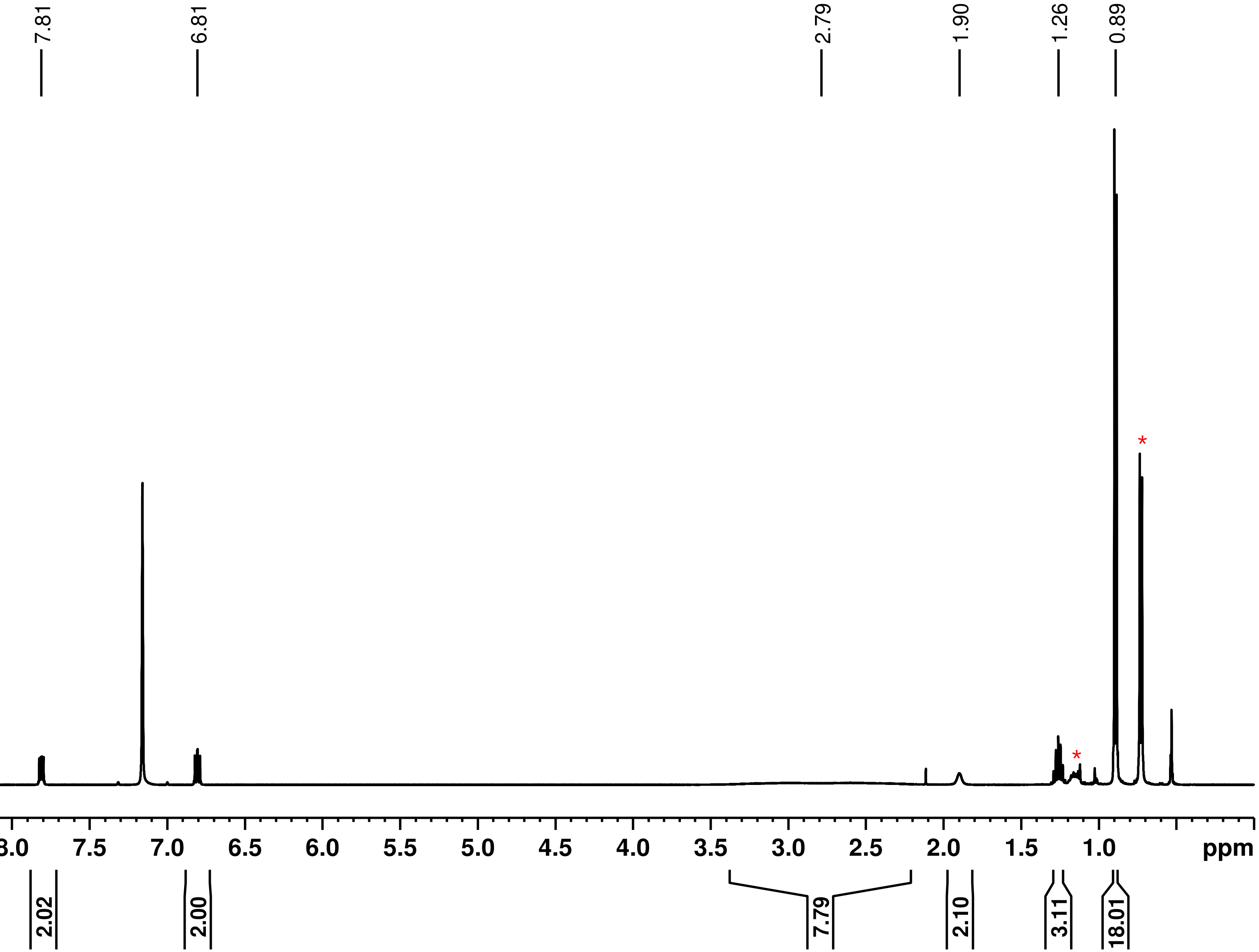

Supplementary Fig. 218. **^{11}B NMR** spectrum (160 MHz, C_6D_6, 298 K) of [$i$$Pr_3$Si(FBN)]$^+$[$HCB_{11}H_5Br_6$]$^-$ ([**2c**(FBN)]$^+$[$HCB_{11}H_5Br_6$]$^-$)

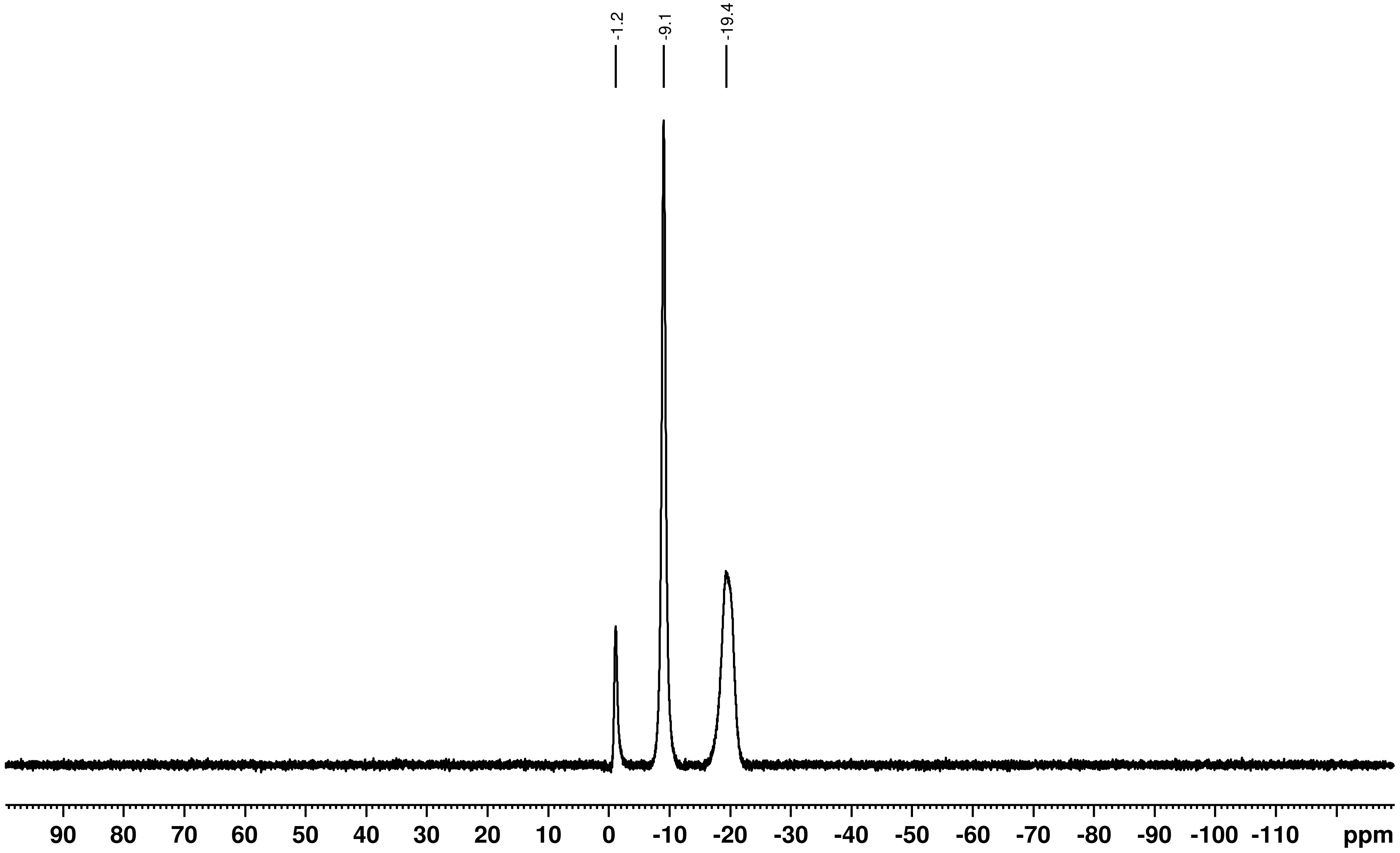

Supplementary Fig. 219. **$^{13}C\{^{1}H\}$ NMR** spectrum (126 MHz, C_6D_6, 298 K) of [*i*Pr_3Si(FBN)]$^+$[$HCB_{11}H_5Br_6$]$^-$ ([**2c**(FBN)]$^+$[$HCB_{11}H_5Br_6$]$^-$) (* [*i*Pr_3Si($HCB_{11}H_5Br_6$)])

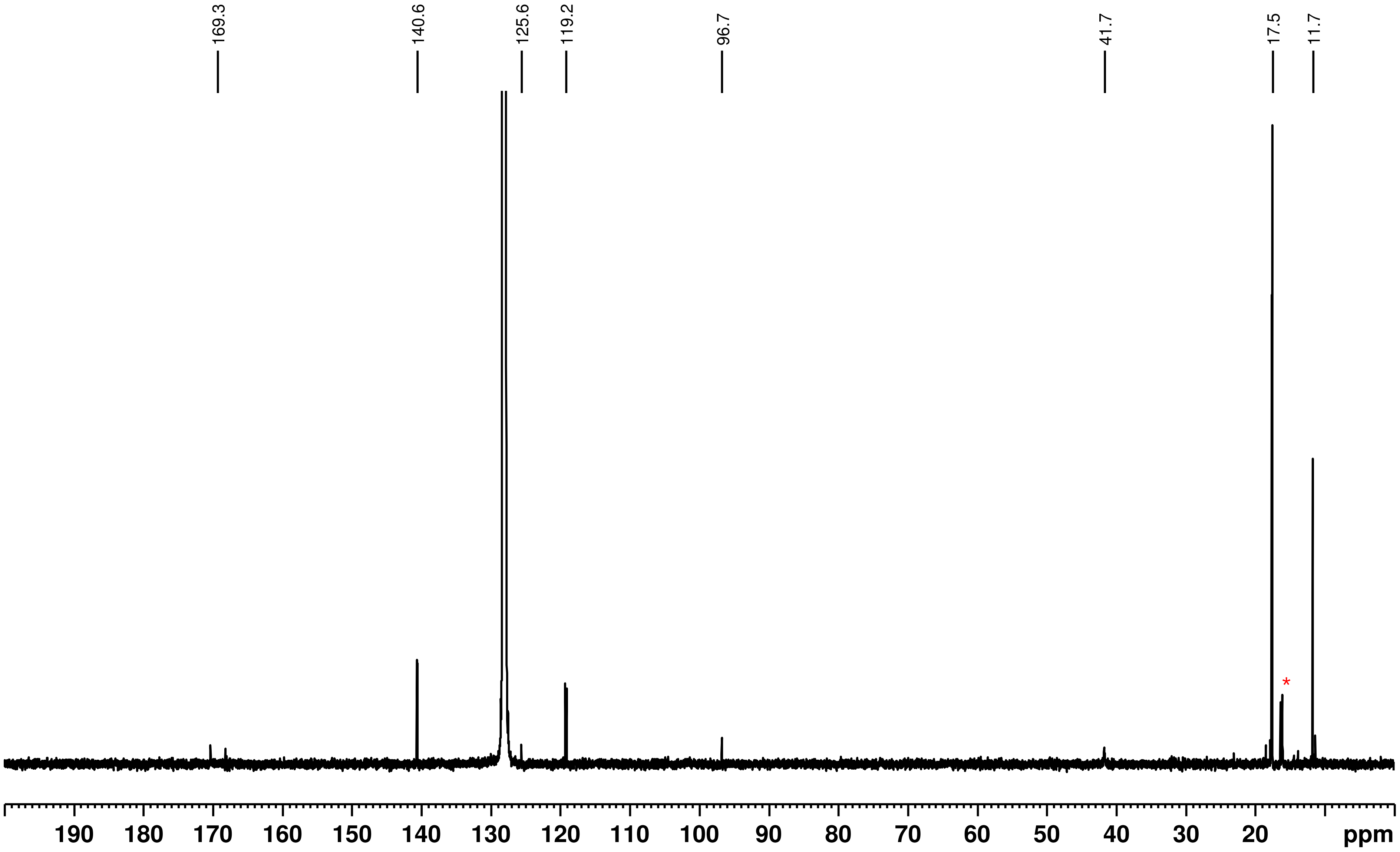

Supplementary Fig. 220. ^{19}**F NMR** spectrum (471 MHz, C_6D_6, 298 K) of [*i*$Pr_3Si(FBN)]^+[HCB_{11}H_5Br_6]^-$ ([**2c**(FBN)]$^+[HCB_{11}H_5Br_6]^-$)

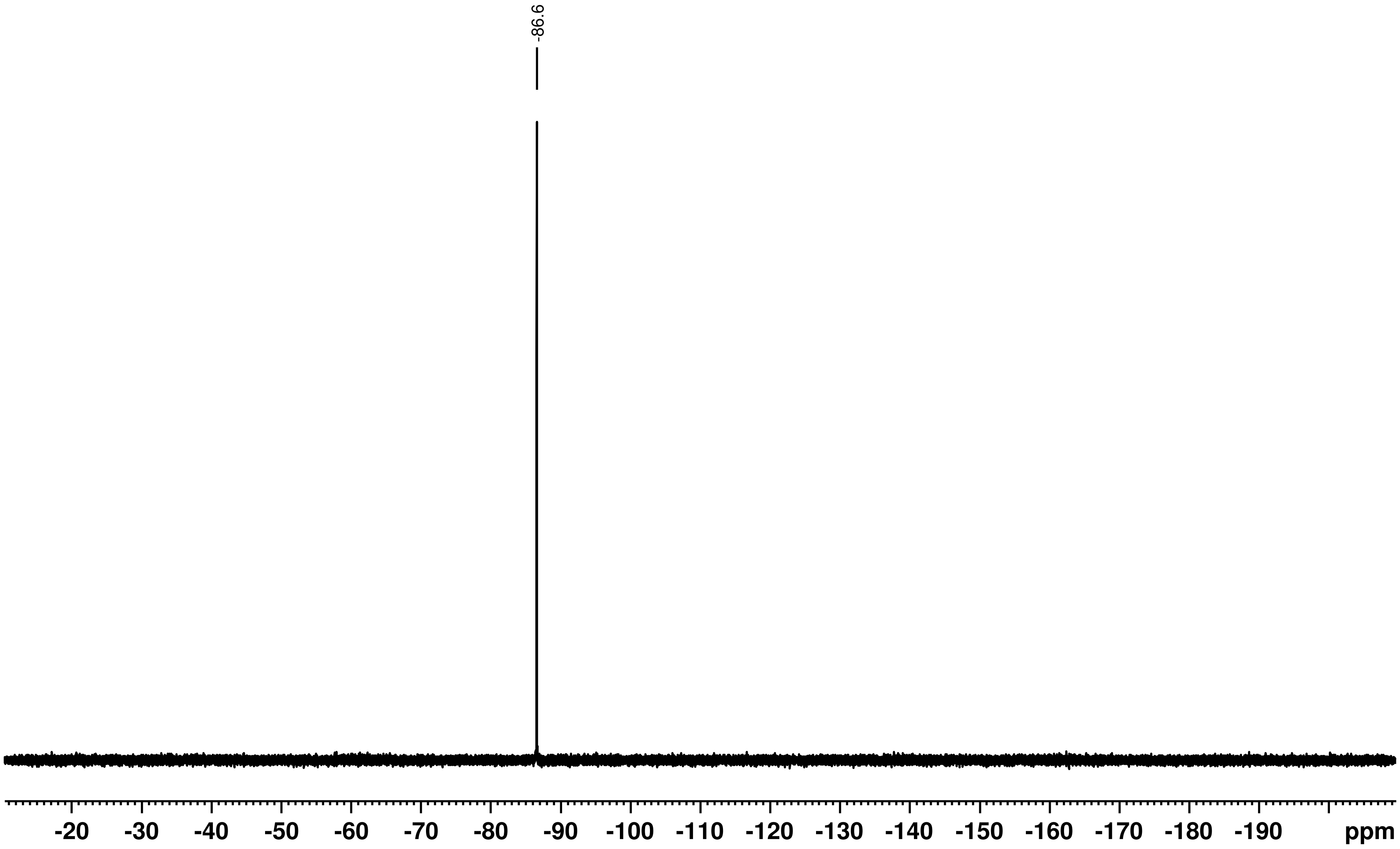

Supplementary Fig. 221. **^{29}Si{^{1}H} DEPT NMR** spectrum (99 MHz, C_6D_6, 298 K, optimized for $J_{H,Si}$ = 7 Hz, 12.6°) of [*i*Pr_3Si(FBN)]$^+$[$HCB_{11}H_5Br_6$]$^-$ ([**2c**(FBN)]$^+$[$HCB_{11}H_5Br_6$]$^-$)

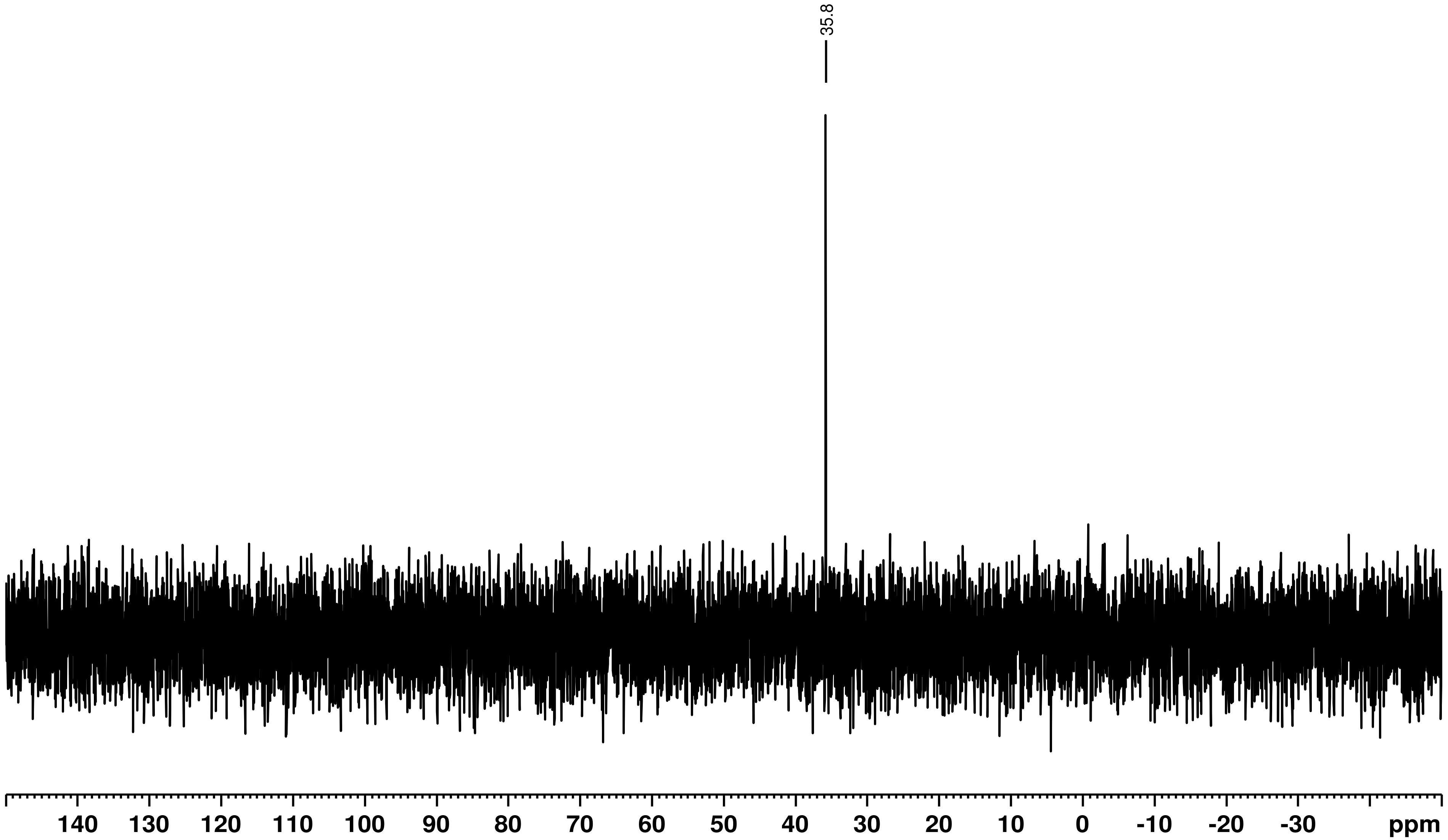

9 References

41 Harris, R. K., Becker, E. D., Cabral de Menezes, S. M., Goodfellow, R., Granger, P. NMR Nomenclature: Nuclear Spin Properties and Conventions for Chemical Shifts: IUPAC Recommendations 2001. *Solid State Nucl. Magn. Reson.* **22**, 458–483 (2002).

42 Agilent Technologies, CrysAlisPro, Data Collection and Processing Software for Agilent X-ray Diffractometers, Yarnton, Oxfordshire, UK, **2012**.

43 Sheldrick, G. M. Phase annealing in *SHELX*-90: Direct methods for larger structures. *Acta Crystallogr. A.* **46**, 467–473 (1990).

44 Sheldrick, G. M. A short history of SHELX. *Acta Crystallogr. A.* **64**, 112–122 (2008).

45 Cambridge Crystallographic Data Centre, Mercury 2024.3.0, Cambridge, UK, **2024**, can be found under https://www.ccdc.cam.ac.uk/solutions/software/mercury/.

46 Omann, L., Qu, Z.-W., Irran, E., Klare, H. F. T., Grimme, S., Oestreich, M. Electrophilic Formylation of Arenes by Silylium Ion Mediated Activation of Carbon Monoxide. *Angew. Chem. Int. Ed.* **57**, 8301–8305 (2018).

48 Bannwarth, C., Ehlert, S., Grimme, S. GFN2-xTB–An Accurate and Broadly Parametrized Self-Consistent Tight-Binding Quantum Chemical Method with Multipole Electrostatics and Density-Dependent Dispersion Contributions *J. Chem. Theory Comput.* **15**, 1652–1671 (2019).

49 Local version derived from TURBOMOLE V7.9 2024, a development of University of Karlsruhe and Forschungszentrum Karlsruhe GmbH, 1989–2007, TURBOMOLE GmbH, since 2007; available from https://www.turbomole.org.

50 Becke, A. D. Density-functional exchange-energy approximation with correct asymptotic behavior *Phys. Rev. A* **38**, 3098–3100 (1988).

51 Perdew, J. P. Density-functional approximation for the correlation energy of the inhomogeneous electron gas *Phys. Rev. B* **33**, 8822–8824 (1986).

52 Perdew, J. P. Erratum: Density-functional approximation for the correlation energy of the inhomogeneous electron gas *Phys. Rev. B* **34**, 7406 (1986).

53 Weigend, F., Ahlrichs, R. Balanced basis sets of split valence, triple zeta valence and quadruple zeta valence quality for H to Rn: Design and assessment of accuracy *Phys. Chem. Chem. Phys.* **7**, 3297–3305 (2005).

54 Caldeweyher, E., Ehlert, S., Hansen, A., Neugebauer, H., Spicher, S., Bannwarth, C., Grimme, S. A generally applicable atomic-charge dependent London dispersion correction *J. Chem. Phys.* **150**, 154122 (2019).

55 Haasler, M., Maier, T. M., Grotjahn, R., Gückel, S., Arbuznikov, A. V., Kaupp, M. A Local Hybrid Functional with Wide Applicability and Good Balance between

(De)Localization and Left–Right Correlation *J. Chem. Theory Comput.* **16**, 5645–5657 (2020).

56 Rappoport, D., Furche, F. Property-optimized Gaussian basis sets for molecular response calculations *J. Chem. Phys.* **133**, 134105 (2010).

57 Sierka, M., Hogekamp, A., Ahlrichs, R. Fast evaluation of the Coulomb potential for electron densities using multipole accelerated resolution of identity approximation *J. Chem. Phys.* **118**, 9136–9148 (2003).

58 Weigend, F. Accurate Coulomb-fitting basis sets for H to Rn *Phys. Chem. Chem. Phys.* **8**, 1057–1065 (2006).

59 Pollak, P., Weigend, F. Segmented Contracted Error-Consistent Basis Sets of Double- and Triple-ζ Valence Quality for One- and Two-Component Relativistic All-Electron Calculations *J. Chem. Theory Comput.* **13**, 3696–3705 (2017).

60 Adamo, C., Barone, V. Toward reliable density functional methods without adjustable parameters: The PBE0 model *J. Chem. Phys.* **110**, 6158–6170 (1999).

61 Boettger, J. C. Approximate two-electron spin-orbit coupling term for density-functional-theory DFT calculations using the Douglas-Kroll-Hess transformation *Phys. Rev. B* **62**, 7809–7815 (2000).

62 Filatov, M., Zou, W., Cremer, D. Spin-orbit coupling calculations with the two-component normalized elimination of the small component method *J. Chem. Phys.* **139**, 014106 (2013).

63 Zou, W., Filatov, M., Cremer, D. Analytical energy gradient for the two-component normalized elimination of the small component method *J. Chem. Phys.* **142**, 214106 (2015).

64 Franzke, Y. J., Middendorf, N., Weigend, F. Efficient implementation of one- and two-component analytical energy gradients in exact two-component theory *J. Chem. Phys.* **148**, 104110 (2018).

65 Visscher, L., Dyall, K. G. Dirac–Fock Atomic Electronic Structure Calculations Using Different Nuclear Charge Distributions *At. Data Nucl. Data Tables* **67**, 207–224 (1997).

66 Repisky, M., Komorovsky, S., Kadek, M., Konecny, L., Ekström, U., Malkin, E., Kaupp, M., Ruud, K., Malkina, O. L., Malkin, V. G. ReSpect: Relativistic spectroscopy DFT program package *J. Chem. Phys.* **152**, 184101 (2020).

67 Jensen, F. Polarization consistent basis sets: Principles *J. Chem. Phys.* **115**, 9113–9125 (2001).

68 Jensen, F. Polarization consistent basis sets. II. Estimating the Kohn–Sham basis set limit *J. Chem. Phys.* **116**, 7372–7379 (2002).

69 Jensen, F., Helgaker, T. Polarization consistent basis sets. V. The elements Si–Cl *J. Chem. Phys.* **121**, 3463–3470 (2004).

70 Dyall, K. G. Relativistic Quadruple-Zeta and Revised Triple-Zeta and Double-Zeta Basis Sets for the 4p, 5p, and 6p Elements *Theor. Chem. Acc.* **115**, 441–447 (2006).

71 Jensen, F. Polarization Consistent Basis Sets. 4: The Elements He, Li, Be, B, Ne, Na, Mg, Al, and Ar *J. Phys. Chem. A* **111**, 11198–11204 (2007).

72 NBO 7.0. E. D. Glendening, J. K. Badenhoop, A. E. Reed, J. E. Carpenter, J. A. Bohmann, C. M. Morales, P. Karafiloglou, C. R. Landis, and F. Weinhold, Theoretical Chemistry Institute, University of Wisconsin, Madison, WI, **2018**.
